# Oxetane Cleavage Pathways in the Excited State: Photochemical Kinetic Resolution as an Approach to Enantiopure Oxetanes

**DOI:** 10.1021/jacs.5c02483

**Published:** 2025-04-14

**Authors:** Niklas Pflaum, Mike Pauls, Ajeet Kumar, Roger Jan Kutta, Patrick Nuernberger, Jürgen Hauer, Christoph Bannwarth, Thorsten Bach

**Affiliations:** † Department Chemie and Catalysis Research Center (CRC), School of Natural Sciences Technische Universität München, D-85747 Garching, Germany; ‡ Institut für Physikalische Chemie RWTH Aachen University, D-52074 Aachen, Germany; § Institut für Physikalische und Theoretische Chemie, Universität Regensburg, Universitätsstr. 31, D-93053 Regensburg, Germany

## Abstract

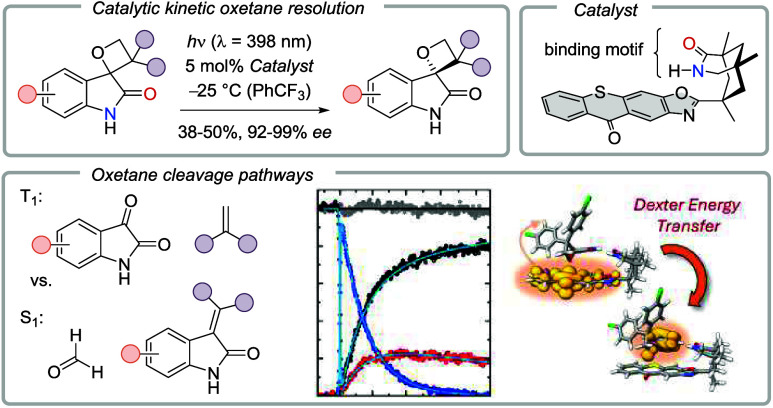

Chiral spirocyclic oxetanes [2-oxo-spiro(3*H*-indole-3,2′-oxetanes)] were subjected to irradiation in the presence of a chiral thioxanthone catalyst (5 mol %) at λ = 398 nm. An efficient kinetic resolution was observed, which led to an enrichment of one oxetane enantiomer as the major enantiomer (15 examples, 37−50% yield, 93−99% *ee*). The minor enantiomer underwent decomposition, and the decomposition products were carefully analyzed. They arise from a photocycloreversion (retro-Paternò–Büchi reaction) into a carbonyl component and an olefin. The cycloreversion offers two cleavage pathways depending on whether a C−O bond scission or a C−C bond scission occurs at the spirocyclic carbon atom. The course of this reaction was elucidated by a suite of mechanistic, spectroscopic, and quantum chemical methods. In the absence of a catalyst, cleavage occurs exclusively by initial C−O bond scission, leading to formaldehyde and a tetrasubstituted olefin as cleavage products. Time-resolved spectroscopy on the femtosecond/picosecond time scale, synthetic experiments, and calculations suggest the reaction to occur from the first excited singlet state (S_1_). In the presence of a sensitizer, triplet states are populated, and the first excited triplet state (T_1_) is responsible for cleavage into an isatin and a 1,1-diarylethene by an initial C−C bond scission. The kinetic resolution is explained by the chiral catalyst recruiting predominantly one enantiomer of the spirocyclic oxindole. A two-point hydrogen-bonding interaction is responsible for the recognition of this enantiomer, as corroborated by NMR titration studies and quantum chemical calculations. Transient absorption studies on the nanosecond/microsecond time scale allowed for observing the quenching of the catalyst triplet by either one of the two oxetane enantiomers with a slight preference for the minor enantiomer. In a competing situation with both enantiomers present, energy transfer to the major enantiomer is suppressed initially by the better-binding minor enantiomer and—as the reaction progresses—by oxindole fragmentation products blocking the binding site of the catalyst.

## Introduction

**Scheme 1:**
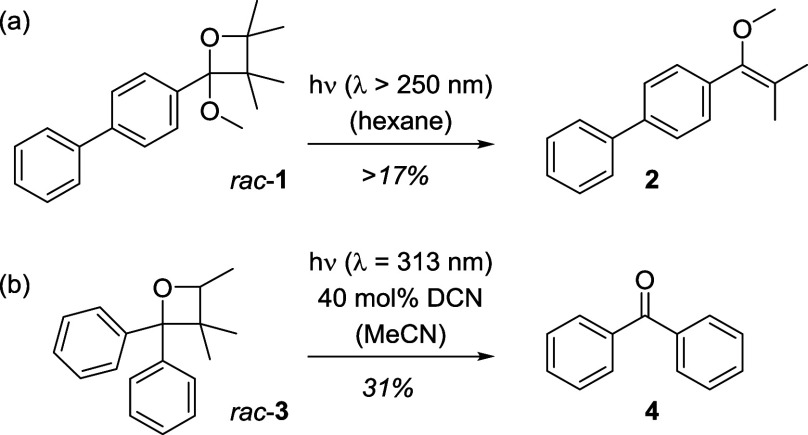
Known Examples of Photochemical Oxetane Cleavage Reactions by Direct Excitation (a)^8^ or Photoinduced Electron Transfer (b) with 1,4-Dicyanonaphthalene (DCN)^9a^ as the Oxidant

Oxetanes (oxacyclobutanes) represent an important and versatile class of heterocyclic compounds.^1^ They occur in several natural products, and they have been identified as equivalents for geminal dimethyl and carbonyl groups in medicinal chemistry.^2^ Due to their ring strain, the C−O bond of oxetanes is labile and can be cleaved by appropriate nucleophiles. Oxetanes, thus, serve as building blocks with latent 1,3-difunctionality, and a range of ring-opening reactions have been described in recent years.^1,3^ Regarding their synthesis, one of the most facile and concise reactions^4^ leading to oxetanes is the [2 + 2] photocycloaddition reaction of carbonyl compounds to olefins, the Paternò–Büchi reaction.^5^ In most cases, the reaction proceeds stepwise on the triplet potential energy surface (PES) via short-lived 1,4-diradical intermediates.^6^ It has been found that the thermal cycloreversion occurs in the opposite sense as the photocycloaddition enabling a formal metathesis by a two-step procedure.^7^ Several studies have addressed the photochemical cycloreversion of oxetanes (Scheme 1).

Cantrell and co-workers observed that the Paternò–Büchi reaction of benzoates and simple olefins such as 2,3-dimethyl-2-butene delivered side products stemming from a cycloreversion.^8^ They showed that the reaction was not thermally induced, but that light was required to trigger the cleavage. Oxetane *rac*-**1** for example was formed from methyl biphenyl-4-carboxylate upon irradiation with a medium-pressure mercury lamp through a Vycor filter but delivered under these conditions also enol ether **2** as its photochemical cleavage product. In a series of papers, Shima and co-workers studied the cycloreversion of oxetanes upon photoinduced electron transfer (PET).^9^ They discovered the reverse of a Paternò–Büchi reaction for oxetane *rac*-**3** if irradiated at λ = 313 nm in the presence of 1,4-dicyanonaphthalene (DCN). Benzophenone (**4**) was isolated together with 2-methyl-2-butene (24%) as products of the photochemical cycloreversion.^9a^ While this process likely proceeds by oxidation of the oxetane, ring cleavage was also detected at a short wavelength (λ = 254 nm) in the presence of a reductant, such as triethylamine.^9b^ Griesbeck and co-workers used a reductive PET to initiate the cleavage of bicyclic oxetanes in a formal carbonyl–alkene metathesis reaction.^10^ The topic of photochemical oxetane cleavage has received extensive attention in the context of DNA repair, specifically concerning oxetane-containing (6−4) DNA lesions.^11^ Miranda and co-workers performed seminal mechanistic work on the cleavage of oxetanes by PET, either employing strong photochemical oxidants or reductants.^12^

**Scheme 2:**
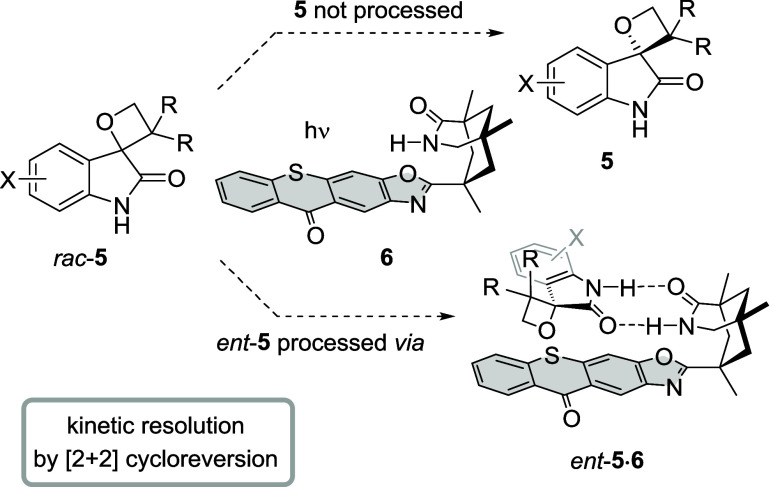
Model for a Possible Kinetic Resolution of Oxetanes *rac*-**5** by Chiral Thioxanthone **6**: [2 + 2] Cycloreversion of Enantiomer *ent*-**5** upon Energy Transfer

Our interest in the cycloreversion of oxetanes was kindled by recent work on the photochemical deracemization^13^ of spirocyclopropyl oxindoles with a chiral thioxanthone. It had been discovered that a C−C bond cleavage was induced upon energy transfer and that one enantiomer was preferentially processed by the catalyst.^14^ We hypothesized that spirocyclic oxetanes **5** would undergo similar bond fission, which would trigger a [2 + 2] cycloreversion.^15^ Catalyst **6** displays a binding motif, which invites coordination to a given lactam and accelerates energy transfer within the respective complex.^16^ Discrimination between enantiomers **5** and *ent*-**5** seemed possible because the bulky substituents R would suffer from Pauli repulsion with the backbone of catalyst **6**, while enantiomer *ent*-**5** was expected to bind without any significant constraints (Scheme 2).

The method promised straightforward, unprecedented access to enantiopure oxetanes. Although an enantioselective Paternò–Büchi reaction has been very recently reported by Yoon and co-workers,^17^ there is no other precedence for the formation of oxetanes with high enantiomeric excess (*ee*) by a catalytic photochemical method.

We have now undertaken a collaborative effort to study the photochemical cleavage of oxetanes *rac*-**5** in detail. We see divergent cleavage pathways for the oxetanes depending on the nature of the excited state and experimentally observe remarkably strict discrimination between the two enantiomers by catalyst **6**. Computational studies support the idea of a photoinduced bond fission after energy transfer, while time-resolved spectroscopy data shed light on the photophysics of the thioxanthone chromophore and cleavage pathways after excitation.

## Results and Discussion

### Synthetic Studies, Kinetic Resolution, and Association Constants

**Scheme 3:**
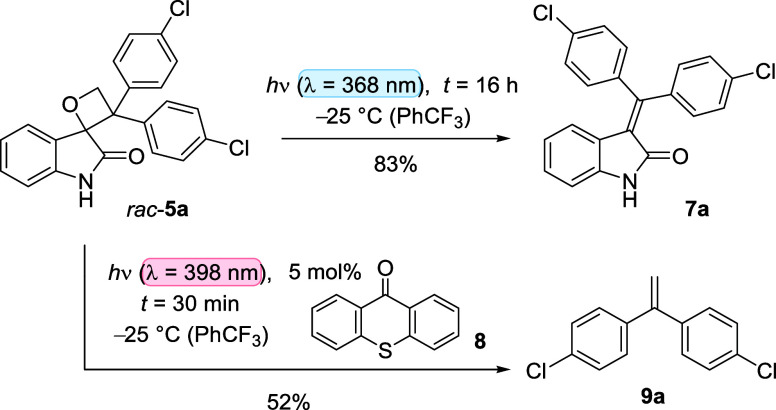
Photochemical Cleavage of Oxetane *rac*-**5a** in the Absence and Presence of Thioxanthen-9-one (**8**) as a Sensitizer

Before turning toward kinetic resolution experiments, we studied a possible cleavage of oxetanes under photolytic conditions. The racemic starting materials *rac*-**5** for our studies were typically prepared by the Paternò–Büchi reaction of *N*-acetylisatin and 1,1-disubstituted olefins followed by deacetylation.^18^ Due to its superior solvation properties and ease of handling, the 4-chlorophenyl-substituted oxetane *rac*-**5a** served as a model compound for many preliminary and mechanistic studies. The compound displays a broad UV/vis absorption centered at λ = 312 nm (ε = 1400 M^−1^ cm^−1^, CH_2_Cl_2_), which stretches to a wavelength of λ = 370 nm. When irradiated at λ = 368 nm in trifluorotoluene at −25 °C, the compound underwent a clean [2 + 2] photocycloreversion that led exclusively to tetrasubstituted olefin **7a** (Scheme 3).

The compound was isolated in 83% yield, while the isolation/detection of formaldehyde was not attempted. The addition of piperylene as a possible triplet quencher^19^ did not suppress the rate of the cleavage reaction. If the same compound was irradiated under otherwise identical conditions at λ = 398 nm, neither cycloreversion nor any other photochemical reaction was observed. The addition of 5 mol % thioxanthen-9-one (**8**) induced a rapid decomposition of the compound at λ = 398 nm, generating exclusively the 1,1-disubstituted olefin **9a** as the cleavage product. After 30 min of irradiation, 52% of compound **9a** and 46% of oxetane *rac*-**5a** were isolated. Isatin was detected but appeared to decompose under the reaction conditions. The observation is in agreement with the fact that isatin is a poor substrate for Paternò–Büchi reactions.^20^ Under sensitized irradiation conditions, there was no indication of the formation of tetrasubstituted olefin **7a**, nor was there any evidence for the formation of olefin **9a** and isatin under direct irradiation conditions (λ = 368 nm). To exclude a cleavage via photochemical oxidation by thioxanthen-9-one, we determined the oxidation potential of oxetane **5a** and attempted its cleavage with a stronger oxidant but thioxanthen-9-one (**8**). For thioxanthone **8**, the redox potential in the excited state was calculated as *E*_1/2_(8*/**8**^**•**−^) = +1.22 V vs SCE (MeCN) from its triplet-state energy *E*_T_ = 274 kJ mol^−1^ (77 K, methylcyclohexane-isopentane)^21^ and its ground-state redox potential *E*_1/2_(**8**/**8**^**•**−^) = −1.62 V vs SCE (MeCN).^22^ The peak potential for the oxidation of compound *rac*-**5a** was measured as *E*_p_ = +1.33 V vs SCE (MeCN). Ruthenium complex Ru(bpz)_3_(PF_6_)_2_ (bpz = 2,2′-bipyrazine) was used as a stronger oxidant, whose excited redox potential has been reported as *E*_1/2_(Ru^II^*/Ru^I^) = +1.45 V vs SCE (MeCN).^23^ Upon irradiation at λ = 455 nm in the presence of 2 mol % Ru(bpz)_3_(PF_6_)_2_ (MeCN/PhCF_3_), the oxetane *rac*-**5a** was quantitatively recovered.

**Scheme 4:**
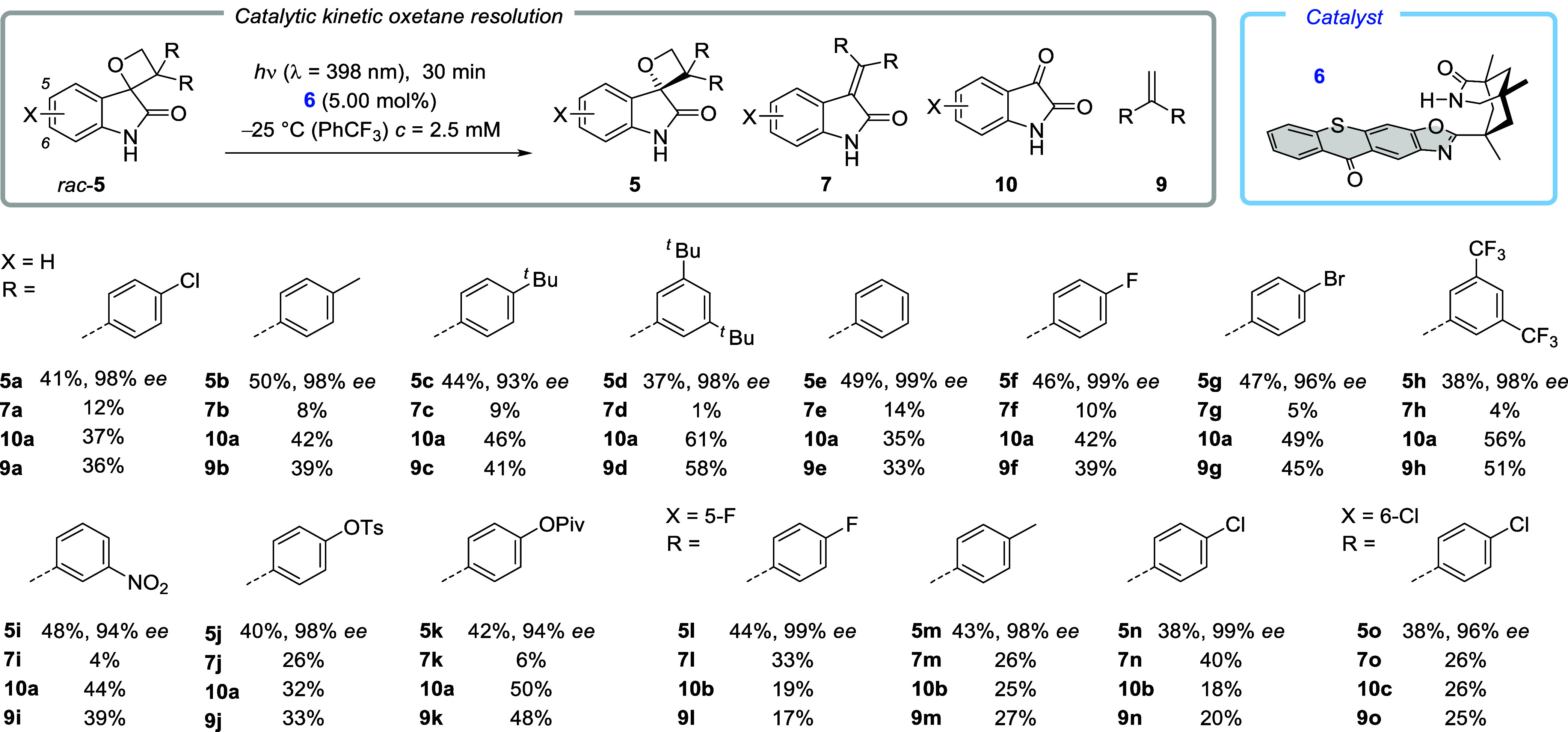
Photochemical Kinetic Resolution of Chiral Oxetanes *rac*-**5** upon Irradiation in the Presence of Chiral Thioxanthone **6**

The preliminary experiments, thus, suggested that cleavage of compound *rac*-**5a** into formaldehyde and olefin **7a** occurs by direct excitation (λ = 368 nm) via a singlet intermediate, while the [2 + 2] photocycloreversion to isatin and 1,1-disubstituted olefin **9a** at λ = 398 nm occurs by triplet energy transfer from thioxanthen-9-one (**8**). The latter observation encouraged us to continue studies toward a projected kinetic resolution employing chiral sensitizer **6**. Optimization experiments commenced by using 5 mol % catalyst **6** in various solvents at different irradiation conditions (see the Supporting Information for an overview on all optimization experiments). It was found that the expected photocycloreversion indeed occurred and that enantioenriched oxetane could be isolated. Trifluorotoluene evolved as the preferred solvent at a wavelength of λ = 398 nm and a reaction temperature of −25 °C. By monitoring the *ee* over time, it became evident that the reaction was complete after less than 30 min. The exclusion of oxygen was important to guarantee reproducibility and consistent enantioselectivities. Lowering the catalyst loading to 2.5 mol % was possible and gave a 95% *ee*, but the results remained slightly inferior to the results recorded with 5 mol % catalyst. Careful analysis of the cleavage products revealed the mass balance of the reaction to be high. Against the background of our preliminary work (Scheme 3), it was surprising, however, that not only isatin (**10a**) and 1,1-disubstituted olefin **9a** were isolated as cleavage products but also tetrasubstituted olefin **7a**. Under optimized conditions, on a 1 mmol scale, oxetane *rac*-**5a** produced 41% of analytically pure oxetane **5a** (98% *ee*) after chromatography. Thioxanthone catalyst **6** was almost quantitatively recovered (98% yield), while fragmentation products isatin (**10a**) and 1,1-disubstituted alkene **9a** were isolated in yields of 37 and 36%, respectively. Tetrasubstituted olefin **7a** was tedious to separate from the oxetane, which led to some loss in the material. It was isolated in 12% yield. At a smaller scale (50 μmol), at which the other reactions were run, the separation was not feasible. Here, the respective enantioenriched oxetane **5** and olefin **7** were isolated as a mixture of two compounds, and the yield of each component was calculated from their relative ratio (^1^H NMR integration). The kinetic resolution protocol turned out to be robust, and a wide array of diphenylspiro-oxetanes was successfully taken into the reaction. Apart from alkyl-substituted compounds **5b**–**5d** and from the unsubstituted 1,2-dihydro-2-oxo-3′,3′-diphenylspiro[3*H*-indole-3,2′-oxetane] (**5e**), functional groups at the phenyl ring included fluoro (**5f**), bromo (**5g**), trifluoromethyl (**5h**), nitro (**5i**), sulfonate (**5j**), Ts = 4-methylphenylsulfonyl), and ester (**5k**, Piv = pivaloyl) groups (Scheme 4). The fragmentation pathway toward isatin (**10a**) and 1,1-disubstituted alkene **9** was clearly preferred. Since the latter compounds are formed by the same cleavage pathway, the average yield for both products should be taken to calculate the combined yield and evaluate the mass balance. For oxetane *rac*-**5a** as an example, the combined product yield is 41% + 12% + 36.5% = 89.5%. The *s* factors for the cycloreversion of two enantiomeric oxetanes *ent*-**5** vs **5** were calculated from the conversion and *ee*.^24^ Since the conversion was in our case determined from the yield of recovered oxetane **5**, any loss in the material during isolation strongly affects the calculation. Thus, *s* factors vary between 14 (for **5o**) and >100 (for **5b** and **5e**, see the Supporting Information for a complete list), and they indicate a significant kinetic preference for processing oxetane *ent*-**5** over **5**. The absolute configuration of the enantiopure oxetane was exemplarily proven for product **5a** by a previously described method^25^ (see the Supporting Information for details). The fragmentation pattern changed somewhat once the benzo part of the indolinone was halogen-substituted (substrates *rac*-**5l** to *rac*-**5o**). The enantioselectivity achieved in the kinetic resolution remained excellent (96−99% *ee*), but the amount of tetrasubstituted olefins **7** increased at the expense of isatins **10** and alkenes **9**. The total combined yields were high (≥90%), underpinning the fact that apart from the photocycloreversion, no other reactions occurred. In general, the kinetic resolution method offers a facile and operationally simple entry to enantiopure oxetanes.

**Figure 1:**
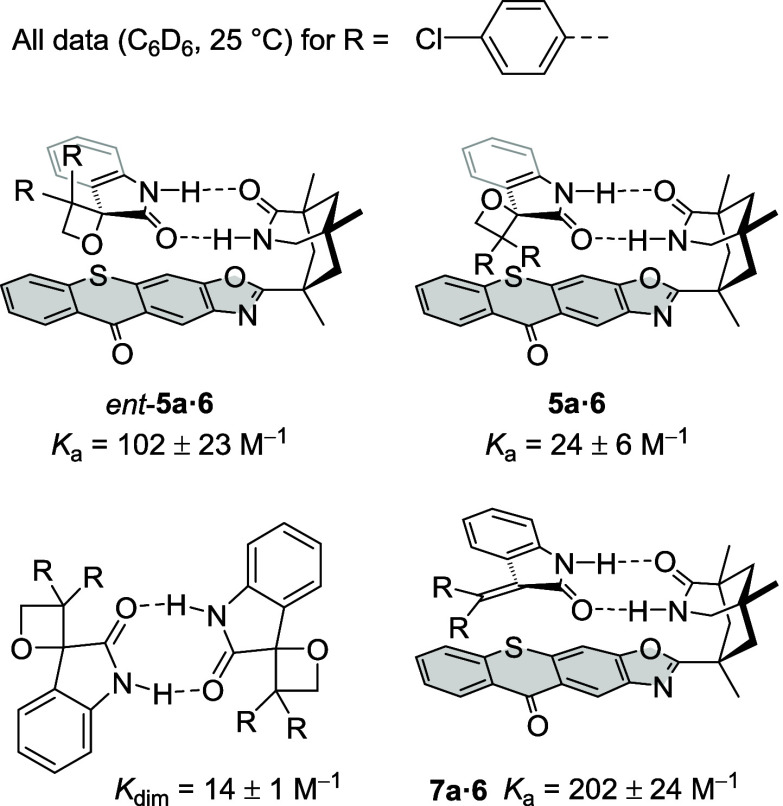
(Top) Complexes of chiral thioxanthone **6** with the two enantiomers of oxetane **5a**: enantiomer **5a** obtained by resolution displays a smaller binding constant than the other enantiomer *ent*-**5a**. (Bottom) Dimerization of two spirocyclic oxetanes *rac*-**5a** and the complex between tetrasubstituted olefin **7a** and catalyst **6**.

It has already been mentioned in the Introduction section that recognition of the catalyst was believed to be key for the success of the reaction. Association constants (*K*_a_) for the two enantiomers *ent*-**5a** and **5a** were measured by NMR titration in deuterated benzene at ambient temperature. The value for *ent*-**5a** (matched) was by a factor of 4 higher than that for **5a** (mismatched), which is very likely due to the increase of steric repulsion in the latter case. The dimerization constant of the substrate (*K*_dim_) was found to be lower than the association constant *K*_a_, while tetrasubstituted olefin **7a** binds well to the catalyst, likely benefiting from an attractive dispersion interaction of the π systems (π stacking). Neither the association constant of isatin (**10a**) to catalyst **6** nor a possible self-association of catalyst **6** could be determined experimentally due to the limited solubility of the components. For the determination of association constants, self-association of the catalyst was assumed to be negligible in line with previous titration studies^26^ of related azabicyclononanones (Figure 1; for further details, see the Supporting Information).

Since the mechanism of the kinetic resolution was not fully understood, further studies were required. In particular, two key questions were to be addressed: (a) Based on the data shown in Figure 1, the association constants seem to be not solely responsible for the discrimination of enantiomers by sensitization from catalyst **6**. Are there significant kinetic differences in the cycloreversion of the two enantiomers or are other factors involved? (b) The occurrence of 1,1-disubstituted alkenes **9** and tetrasubstituted alkenes **7** suggests two divergent cleavage pathways to be operative in the kinetic resolution experiment. Based on the experiments in the achiral series (Scheme 3), the formation of the latter alkene was only observed by direct irradiation, likely (absence of triplet quenching) by a singlet pathway. Is a singlet pathway also involved in the kinetic resolution experiment or is triplet sensitization the exclusive vehicle to induce oxetane cleavage? The raised questions were addressed by transient absorption spectroscopy and quantum chemical methods.

### Transient Absorption and Fluorescence Spectroscopy

**Figure 2:**
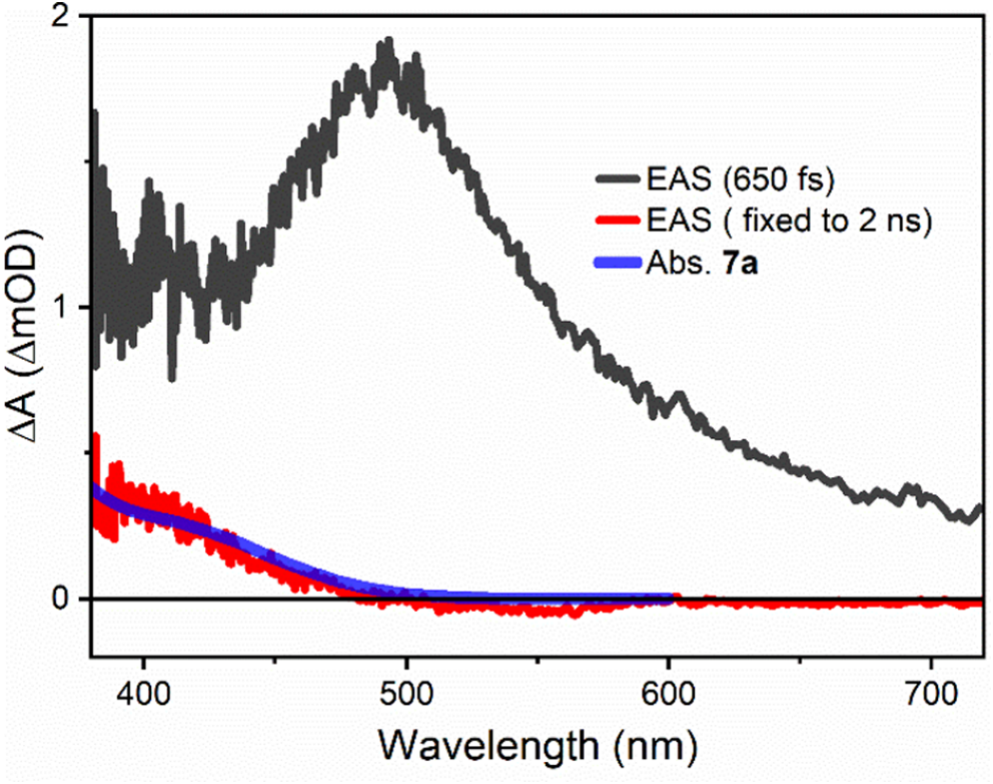
Evolution-associated spectra (EAS) of *rac*-**5a** after 310 nm femtosecond excitation. A comparison of the long-lived species (red full line) with the respective absorption spectrum (transparent full line) assigns this species to photocleavage product **7a**.

Time-resolved spectroscopy was used to describe the ultrafast dynamics of substrate *rac*-**5a**, followed by the investigation of photocatalyst **6** in the absence and presence of *ent*-**5a**. We first examined the photocleavage reaction of oxetane *rac*-**5a** under direct excitation in trifluorotoluene solution (Scheme 3, top; the absorption and excitation spectra can be found in the Supporting Information). The excitation wavelength was selected to match the absorption maximum of the compound at λ_exc_ = 310 nm. Figure 2 shows the global analysis results of the transient absorption measurements of *rac*-**5a** using a sequential deactivation scheme.^27^

An adequate fit of the data requires only two species with the evolution-associated spectra (EAS) shown in black (650 fs lifetime component) and red (fixed to 2 ns) in Figure 2. The short lifetime of the first component supports an assignment to the initially excited singlet state of *rac*-**5a**. The subsequent long-lived species shows weak and mostly positive signals related to photoinduced absorption. A comparison with the absorption spectrum of olefin **7a** allows us to assign the long-lived component to this photoproduct, which is formed directly from the initially excited singlet state of *rac*-**5a**. The result is fully in line with the cycloreversion observed upon direct excitation (Scheme 3) and supports a cleavage on the excited singlet PES.

**Figure 3:**
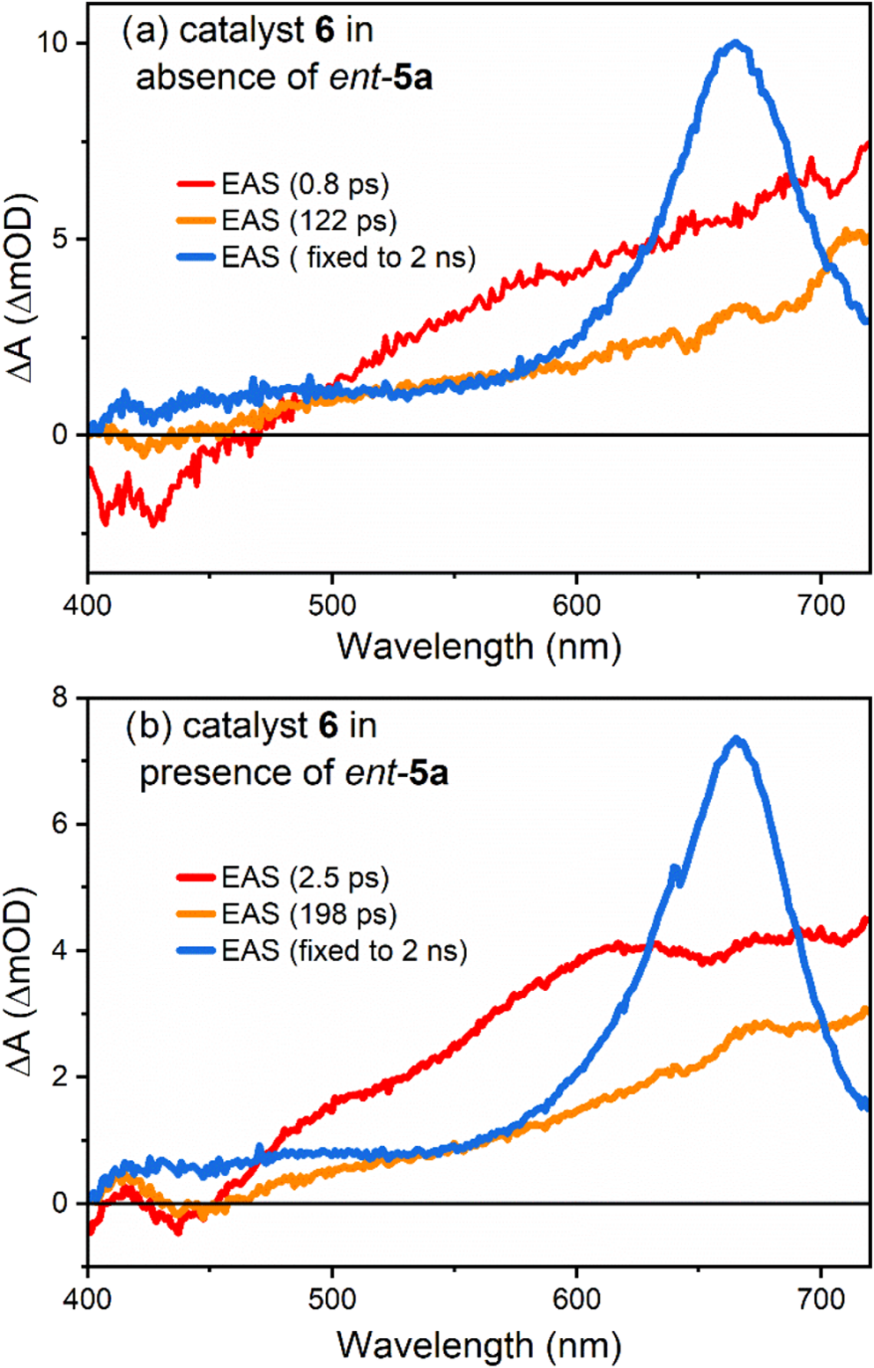
(a) Evolution-associated spectra (EAS) of **6** after 380 nm excitation. The first two components are ascribed to relaxation within (red) and the lifetime of the initially excited singlet state (orange). The long-lived species is the transient signal of the triplet state (blue). (b) Upon the addition of substrate *ent*-**5a**, the number of spectral species for a successful fit remains the same, with largely unchanged spectral signatures. The lifetimes of the initial two species increase.

Time-resolved spectroscopy on the femtosecond time scale (PhCF_3_, λ_exc_ = 380 nm) for chiral thioxanthone **6** showed two short-lived species with lifetimes of 0.8 and 122 ps. Their spectral evolution from broad to more defined structures allows an assignment to cooling (0.8 ps) and lifetime of the initially excited singlet state (122 ps). An alternative interpretation of the initial sub-ps component invokes an ultrafast ππ* → *n*π* excited-state energy transfer as proposed for parent thioxanthen-9-one (**8**).^28^ The long-lived species (blue in Figure 3) is readily assigned to the triplet state.

Upon addition of the chiral substrate *ent*-**5a**, the number of species necessary for a converged fit remains at three. The spectral shapes are largely unaffected by complex formation between **6** and *ent*-**5a** (see colored curves in Figure 3). The lifetimes of the two initial and singlet-associated species are prolonged by substrate addition. In the case of energy transfer from the excited singlet state of **6** toward substrate *ent*-**5a**, we would expect a decrease in lifetimes, as reported previously.^29^ The observed opposite effect could be explained by a change of molecular geometry upon substrate addition, including a blue shift of energetically close thioxanthone triplet states (see Figure S6) that are relevant for intersystem crossing (ISC), and a lack of singlet energy transfer from the catalyst.

The results suggested that the singlet of thioxanthone **6** is not involved in the cycloreversion process. For additional confirmation, we studied a possible fluorescence quenching of the catalyst by the oxetane. Like other thioxanthones,^30^ thioxanthone **6** is fluorescent and displays a broad steady-state emission spectrum centered at λ_em_ = 419 nm upon excitation at λ_exc_ = 375 nm (*c* = 50 μM, PhCF_3_, 25 °C). There was no decrease in fluorescence intensity if the better-binding (matched) enantiomer *ent*-**5a** was added (*c* = 0.5 mM). The signal remained unchanged, indicating that the emissive singlet state was not quenched by oxetane. Time-dependent fluorescence measurements confirmed the results.

**Figure 4:**
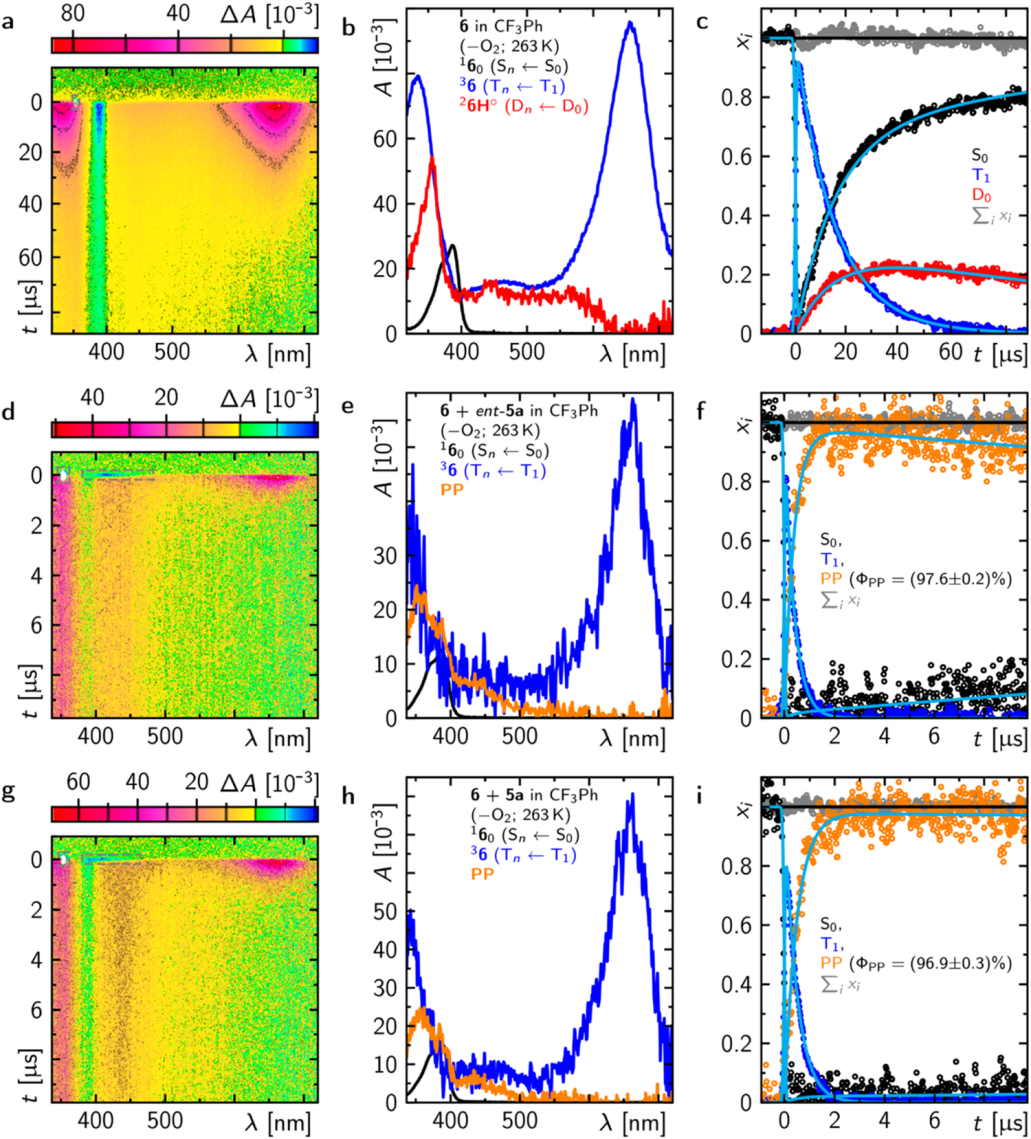
Transient absorption data of **6** (*ca*. 25 μM) in the (a–c) absence or (d–f) presence of either *ent*-**5a** (1.65 mM) or **5a** (1.65 mM) and (g–i) in degassed PhCF_3_ at −10 °C after excitation at 355 nm (*ca*. 3 mJ). (a, d, g) Time-resolved spectra. The gray dashed rectangles indicate spectral areas that were patched during the global fit to the data as described elsewhere.^32^ (b, e, h) Species-associated spectra. (c, f, i) Corresponding mole fraction over time (together with the global fit shown in cyan) that contributes to the data in (a, d, g).

Time-resolved spectroscopy on longer time scales [PhCF_3_, *c*(**6**) ≈ 25 mM, λ_exc_ = 355 nm] in the absence of molecular oxygen at −10 °C revealed that the triplet state of thioxanthone **6** shows some reactivity even in the absence of any additive. The signature of its protonated ketyl radical **6H**(31) was detected, which indicates an intermolecular hydrogen atom transfer from the azabicyclo[3.3.1]nonan-2-one backbone of either a nonexcited or an excited thioxanthone. The lifetime of the thioxanthone triplet was determined to τ_T_1__ = 16 μs. The yield of the radical and its lifetime were determined to Φ_6H°_ = 29% and τ_6H°_ ≈ 147 μs, respectively (Figure 4; PP = photoproducts).

To note, exclusively contributions of HAT are observed in trifluorotoluene, while the HAT occurs only partially in acetonitrile, as evident by an additional rather broad absorption feature at around 650 nm of the thioxanthone radical anion (ketyl radical). Under the same conditions, in the presence of either one of the two enantiomers *ent*-**5a** and **5a** with a concentration of 1.68 mM, the triplet-state lifetime of **6** (τ_T_1__^sub^) is strongly reduced to 386 and 500 ns, and a new persistent (τ_PP_ > 100 μs) signal arises simultaneously with the triplet decay. The spectral features of the signal match the electronic absorption spectra in the UV/vis spectral range of the products. The quantum yields for the photoproduct are determined to 97.6 ± 0.2% and 96.9 ± 0.3% (Φ_PP_ = 1 − τ_T_1__^sub^/τ_T_1__) in the cases of *ent*-**5a** and **5a**, respectively. The electronic absorption spectra, after recording the transient absorption in each case under identical illumination conditions, revealed the formation of oxetane cleavage products with a slightly higher quantum yield in the case of the better-binding (matched) enantiomer *ent*-**5a** (see Figure S13). Since no radical species derived from catalyst **6** are observed, one can conclude that the photoproduct formation proceeds via energy transfer from the triplet of **6** to the substrate. A short-lived triplet diradical intermediate could not be detected, which likely indicates that its rate for further cleavage exceeds the rate of its formation but also means that an electron transfer process cannot be completely ruled out.

Taken together, the transient absorption spectra delivered a clear picture of the decay profile of excited thioxanthone **6** on a time scale from femtoseconds to microseconds. On a femtosecond/picosecond time scale, the singlet species was detected, and its ISC to the triplet state proceeded without any quenching by the oxetanes. The behavior of the long-lived triplet species was monitored on a nanosecond/microsecond time scale. Triplet quenching was confirmed for both oxetanes, which surprisingly appears to occur with almost similar efficiency. Direct excitation of oxetane *rac*-**5a** at λ = 310 nm revealed a rapid decay occurring on the singlet PES.

### Quantum Chemical Calculations on the Cleavage Reactions

In light of the time-resolved spectroscopy experiments, the cause for the enantioselectivity remained to be clarified, as both oxetanes, **5a** and *ent*-**5a**, were shown to be converted to the photoproducts if provided in enantiopure form. Furthermore, the formation of different photoproducts in combination with catalyst **6** needed to be understood. Hence, quantum chemical calculations on thioxanthone **6** and oxetanes **5a** and *ent*-**5a** were performed exemplarily to investigate the ground-state properties and photochemical reaction pathways.

**Figure 5:**
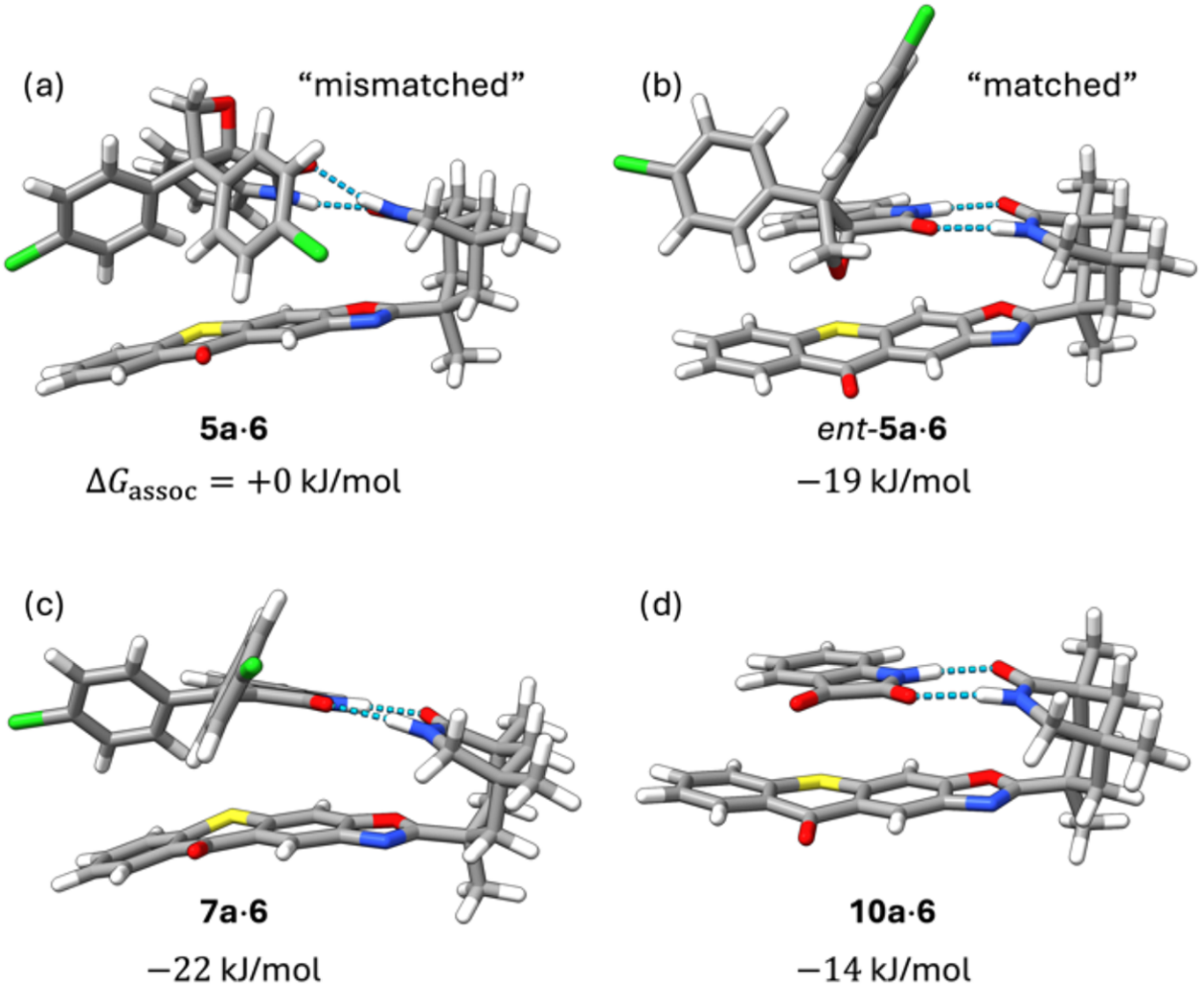
Ground-state geometries of the energetically lowest noncovalent complexes and association free energies Δ*G*_assoc_ of (a) **5a**·**6** and (b) *ent*-**5a**·**6** and photoproduct complexes (c) **7a·6** and (d) **10a**·**6**. The geometries were optimized with the DFT composite method PBEh-3c. Hydrogen-bonding interactions are highlighted as dashed blue lines. All values are given for the formation of one equivalent of the shown complex, starting from the respective thermodynamically favored dimer complexes (see Tables S2 and S3).

We first determined the ground-state geometries and energies of complexes **5a·6** and *ent*-**5a·6** using density functional theory (DFT). Here, the composite method PBEh-3c^33^ was employed to optimize the S_0_ ground-state geometries presented in Figure 5. For both enantiomers, two-point hydrogen bonding to catalyst **6** is feasible. For *ent*-**5a**, we determined a strongly exergonic association free energy of −41 kJ mol^−1^ by means of our computational protocol using wB97X-D4^34,35^/def2-QZVPP^36^//PBEh-3c electronic energies, PBEh-3c^33^ harmonic frequencies for the computation of nuclear zero-point energy and thermostatistical contributions, and solvation corrections computed at the GFN2-xTB/ALPB(DCM)^37^ level of theory (see the Supporting Information for details). In comparison, **5a·6** exhibits an exergonic association energy of −22 kJ mol^−1^. However, self-association of the involved individual compounds competes with the formation of the substrate–catalyst complex and needs to be taken into account. Dimeric species **5a·***ent*-**5a** and **6·6** show association free energies of −24 and −20 kJ mol^−1^, respectively. Dimer **5a·5a** exhibits an association energy of −22 kJ mol^−1^ (see also Table S2). The exergonic self-association allowed us to correct the reported values for catalyst binding by referring to 0.5 equiv of the dimeric species instead of the free substrates. This led to effective association free energies of −19 and +0 kJ mol^−1^ for *ent*-**5a**·**6** and **5a**·**6** shown in Figure 5, respectively. The formation of less favored **5a**·**6** ends up being essentially isoergonic to the respective homodimers, and thus, we expect oxetane *ent*-**5a** to occupy the available catalyst sites, preferably at the beginning of the reaction. We also considered catalyst blocking by the formed decomposition products, particularly by **7a** and isatin (**10a**) (Table S3). Taking into account a potential formation of their dimers, the net association free energies of **7a**·**6** and **10a**·**6** amount to −22 and −14 kJ mol^−1^, respectively (see Figure 5). Consequently, the photoproducts can inhibit the catalyst. Particularly, the formation of the complex with decomposition product **7a** is far more exergonic than the formation of the **5a**·**6** complex (and even slightly more exergonic than *ent*-**5a·6**). Hence, the catalyst will become increasingly occupied with the photoproduct, which is expected to slow down the conversion of *ent*-**5a** and inhibit any significant conversion of the major enantiomer **5a**. The formation of complexes with photoproducts helps to rationalize the observed enantioselectivity in the kinetic resolution, even though Dexter energy transfer within **5a·6** is feasible (Table S5) and conversion of enantiopure **5a** has been observed in time-resolved absorption experiments (vide supra). Due to the different association free energies, we expect the kinetic resolution to occur through favorable formation of *ent*-**5a·6**, while the photocleavage products from *ent*-**5a** prevent significant decomposition of **5a** by blocking the catalyst-binding site (see also Figure 8).

**Figure 6:**
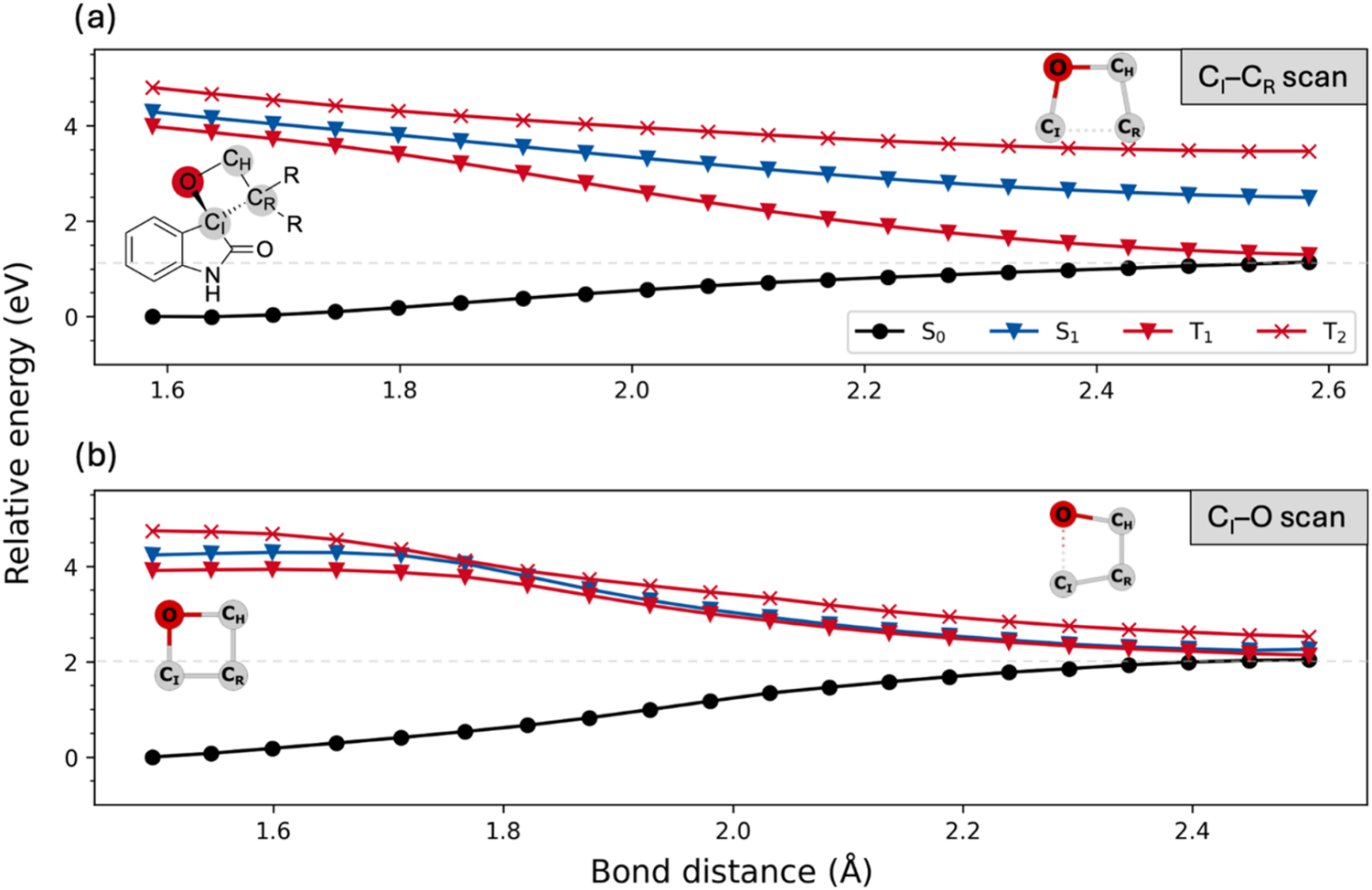
S_0_, S_1_, T_1_, and T_2_ potential energy curves along the C_I_−C_R_ (top) and C_I_−O reaction coordinate (bottom) computed as single points using *hh*-TDA-PBEh-3c. The geometries along this coordinate were generated by constrained optimization with the xtb program^42^ at the GFN2-xTB level of theory using an open-shell configuration (see the Supporting Information for details). Pictograms for the connectivity in the oxetane ring are provided for clarity. We used C_H_, C_I_, and C_R_ to denote the oxetane carbon atoms bound to hydrogen atoms (C_H_), within the isatin core (C_I_), and bound to the substituents R (C_R_).

To probe the excited-state properties of *ent*-**5a·6**, we first computed the vertical excitation energies of *ent*-**5a**, **6**, and the corresponding complexes using density functional theory in conjunction with multireference configuration interaction—DFT/MRCI.^38^ This level of theory was chosen for its high accuracy at still affordable costs for this system size. From these energies (Figure S6), we identify the lowest excited singlet state to be located on the catalyst **6** (Δ*E*_S_1__ = 3.51 eV, vertical excitation), which is expected to be populated upon irradiation with light. At the chosen theory level, the lowest excited singlet state of *ent*-**5a** is significantly higher in energy with Δ*E*_S_1__ = 4.42 eV and thus energetically not accessible for direct excitation at λ = 398 nm (3.12 eV). In alignment with previous work,^39^ we expect that triplet states of **6** can be reached by intersystem crossing (ISC) from S_1_ to energetically proximate triplet states. Under the assumption that Kasha’s rule applies, we first considered the T_1_ state, a state localized on **6**, as a starting point to further investigate the photochemical reaction mechanism via the triplet PES. A Dexter energy transfer^40^ from the localized triplet state on **6** to the triplet state localized on *ent*-**5a** can proceed via T_1_/T_2_ minimum energy conical intersection (MECI) (see Figure S8 and Table S5). For this energy transfer process, an energetic barrier of Δ*E*_MECI_^‡^ = 24 kJ mol^−1^ needs to be overcome according to our calculations (see the Supporting Information for details). It can be expected that this barrier will be passed in the life span of the T_1_ triplet state and that the substrate-localized triplet state, which becomes T_1_ after relaxation, is reached. Transfer to the T_1_ localized on *ent*-**5a** becomes favorable after relaxation of the system, which directly encompasses bond scission of the oxetane ring (C_I_−C_R_ to **5a1** or C_I_−O to **5a2**; see Figure 6 for the used nomenclature). We find that both dissociation pathways can occur in isolated *ent*-**5a** without a significant barrier on the T_1_ but also on the S_1_ state by looking at the PESs along the C_I_−C_R_ and C_I_−O dissociation coordinates. Figure 6 shows the dissociation curves in *ent*-**5a** computed using the hole−hole Tamm-Dancoff approximated density functional theory (*hh*-TDA)^41^ in combination with the PBEh-3c functional (technical details to these scans and further profiles, also taking into account the noncovalent complex geometries, are provided in the Supporting Information).

The top panel of Figure 6 illustrates that on the T_1_ and S_1_ PESs, the ring opening to **5a1** (C_I_−C_R_ opening product) occurs barrierless. The C_I_−O cleavage to **5a2** also occurs via a very small or no energy barrier. A flat region on both states is observed along this coordinate, which is present up to a bond distance of about 1.70 Å. Afterward, the S_1_ and T_1_ energies drop until they become energetically degenerate with each other and also with the S_0_. After crossing to the latter, we expect that relaxation will end up in the formation of a closed-shell species, which is easily achieved by dissociation of formaldehyde from the system. Critically, S_1_/S_0_ degeneracy is not achieved for the C_I_−C_R_ cleavage path but only for T_1_ and S_0_. At bond distances of 2.60 Å, the S_1_ ends up being separated from the S_0_ by 1.35 eV. Furthermore, the S_1_ energy of the C_I_−C_R_ dissociated species is higher by about 0.24 eV compared to the C_I_−O dissociated structure. As a consequence, relaxation to the ground state is less likely for the C_I_−C_R_ pathway, and we expect the excited singlet state of the oxetane to dissociate primarily along the C_I_−O coordinate. The expectation is in full agreement with the experimental observation following direct excitation of oxetane *rac*-**5a** (Scheme 3 and Figure 2). Since the S_0_ and T_1_ states become degenerate in both dissociation channels with the C_I_−C_R_ dissociation product being lower in energy, we expect the latter to be the preferred pathway on the T_1_ surface after Dexter energy transfer has occurred from the lowest triplet state of **6**. The finding is in agreement with the experimental observation that the sensitization with the achiral thioxanthone **8** only leads to the C_I_−C_R_ dissociation products (Scheme 3) and, hence, is enabled through a long-lived triplet state.

**Figure 7:**
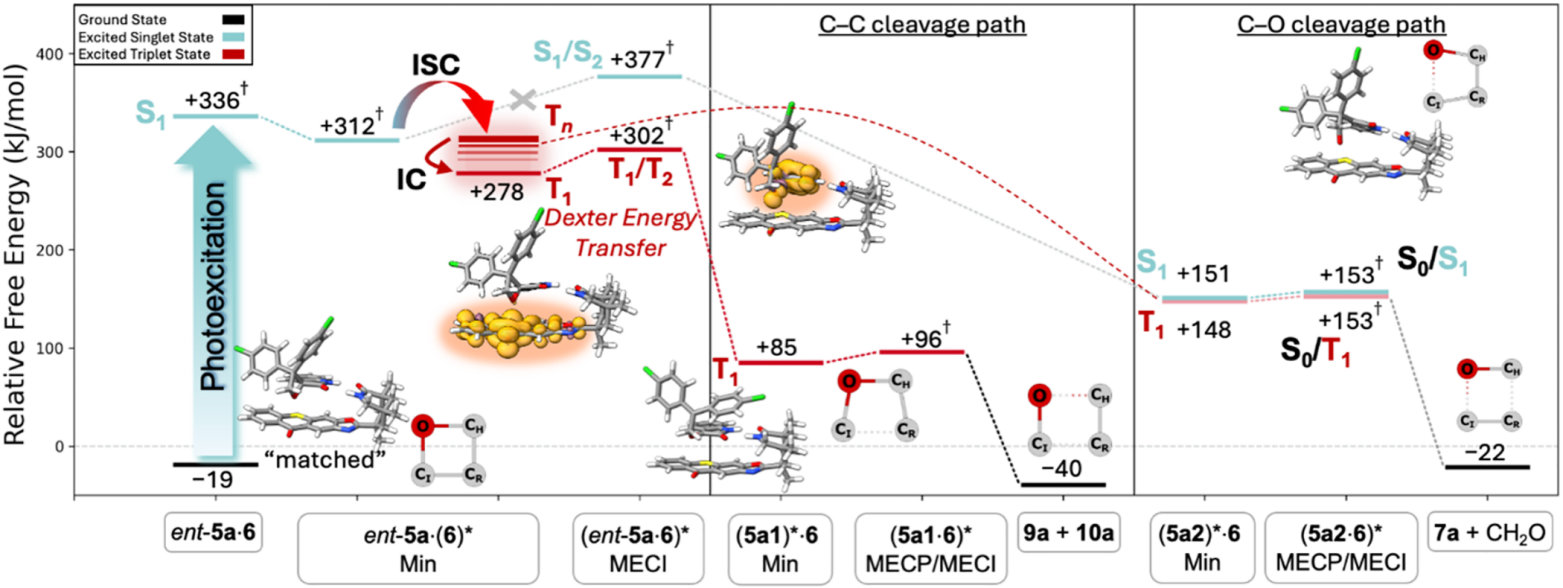
Computed free energy reaction diagram of the photochemical singlet and triplet pathways of *ent*-**5a** in the presence of **6**. Electronic energies are calculated at the wB97X-D4/def2-QZVPP//PBEh-3c level of theory (further details on the theory levels and methods used are provided in the Supporting Information). The connectivity in the oxetane ring is emphasized schematically for the respective reaction steps, and spin densities of the energy transfer process are shown. The asterisk (*) on the labels indicates the position where the excitation is localized. Values marked with a dagger (†) were—due to method-specific technical reasons—determined using an additive scheme including electronic energy differences obtained at other theory levels (see the Supporting Information for details).

The C_I_−O pathway following direct excitation can, thus, be rationalized from the curves along the PES in Figure 6, while the C_I_−C_R_ dissociation appears more likely on the lowest triplet state. The available reaction pathways within complex *ent*-**5a·6** are depicted in Figure 7 and initially involve a Dexter energy transfer process from the lowest thioxanthone triplet state to the lowest oxetane triplet state.

Photoexcitation can occur to the S_1_ state, which according to our DFT/MRCI calculations is a bright thioxanthone-localized ππ* state. We expect complex *ent*-**5a·6** to proceed by ISC to an appropriate triplet state, e.g., the T_3_ state, which is a thioxanthone-localized *n*π*. From there, internal conversion (IC) can occur to the T_1_ state (ππ*) localized on **6**, which in turn enables Dexter energy transfer via the T_1_/T_2_ MECI to the substrate-localized T_1_ state. Bond scission in T_1_ forms either the energetically favorable triplet intermediate (**5a1**)*·6 (Δ*G* = +85 kJ mol^−1^) by C_I_−C_R_ scission or (**5a2**)*·6 (Δ*G* = +148 kJ mol^−1^) by C_I_−O scission. Looking at the energy and the potential energy curves in Figure 6, the energetically lower C_I_−C_R_ scission is expected to be the preferred pathway starting from the thioxanthone T_1_ (ππ*) state. A natural transition orbital (NTO)^43^ analysis of the dominant hole and particle orbitals clearly demonstrates the T_1_ to be localized on the oxetane moiety (Figure S6) after the first bond scission in the oxetane ring. Both ring-opened species can now reach an energetically proximate S_0_/T_1_ minimum energy crossing point (MECP) from where the corresponding cleavage products are obtained. We determine the energy barriers to return to the ground state through the second bond cleavage to be only Δ*E*_MECP_ = +11 kJ mol^−1^ for (**5a1**)*·6 and +5 kJ mol^−1^ in the case of (**5a2**)*·6. It is important to note that we also identified other S_0_/T_1_ MECPs that recover the initial species *ent*-**5a·6** through ring closure. They are close in energy (∼±5 kJ mol^−1^) to the MECPs shown in Figure 7, and thus, a backward reaction seems possible, which would however just recover the oxetane and eventually lead to photocleavage in a subsequent cycle.

For the photocatalytic pathway in *ent*-**5a·6**, it remains to be answered what causes the formation of C_I_−O scission products alongside the C_I_−C_R_ scission product, which is preferred on the T_1_ PES. Due to the high barrier of Δ*E*_MECI_^‡^ = 65 kJ mol^−1^ for the singlet Dexter energy transfer, we expect singlet energy transfer to be unlikely within the lifetime of the lowest excited singlet state localized on thioxanthone. The notion is corroborated by the lack of excited singlet state quenching (Figure 3, vide supra). Given the proximity of catalyst **6** and oxetane *ent*-**5a** in the complex, higher-lying oxetane triplet states may already be involved in the internal conversion process following ISC to the thioxanthone *n*π* triplet state (T_3_). Looking at higher-lying excited states (Figure S6), we find that an oxetane-localized triplet is very close in energy to the thioxanthone S_1_ state (Figures S6 and S7, T_4_, which is 0.11 eV above the thioxanthone ππ* singlet state according to vertical excitation energies from DFT/MRCI). Hence, an internal conversion pathway that involves energy transfer from a higher-lying thioxanthone triplet state directly to a higher oxetane triplet state appears possible. Turning back to the potential energy curves in Figure 6, we can see that the T_2_ state in oxetane has a much flatter PES along the C_I_−C_R_ scission path and separates further from the lower-lying T_1_ state along this path. Hence, the chance of a state crossing between these states is less likely along this coordinate. Different from that, an avoided crossing region for the T_1_ and T_2_ can be detected along the C_I_−O scission path, and both proceed in parallel and energetic proximity toward the C_I_−O scission product. From this, we deduce that the C_I_−O scission products observed in the photocatalytic resolution process in *ent*-**5a·6** do not occur via a singlet pathway but through a pathway that involves higher-lying triplet states. In Figure 7, this is schematically visualized through the ensemble of triplet states and the curved red dashed line. Particularly, the involvement of the T_2_ of oxetane seems likely from the inspection of the PESs shown in Figure 6.

**Figure 8:**
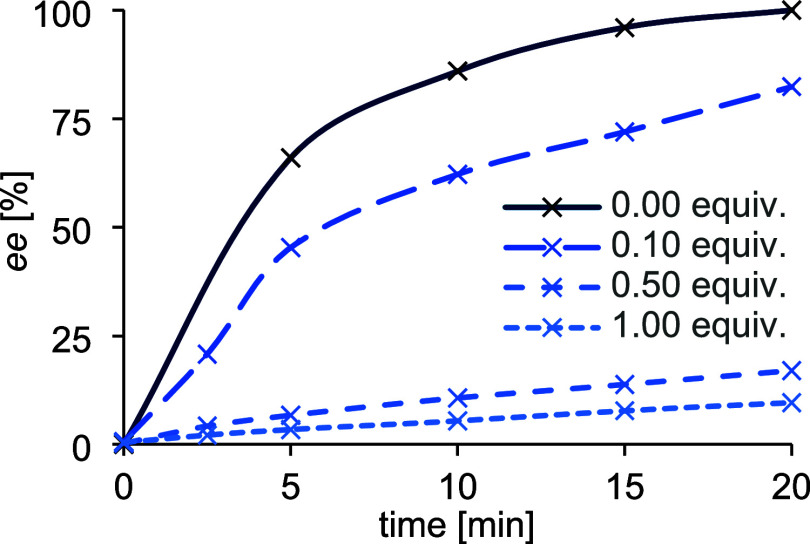
Enantiomeric excess profile of the kinetic resolution *rac*-**5a** → **5a** under standard catalytic reaction conditions (cf. Scheme 4). The oxetane *ee* was recorded as a function of the reaction time. Addition of various amounts of alkene **7a** led to a slower increase of the *ee*, indicating that catalyst **6** is blocked by the olefin and not available for energy transfer.

A final synthetic study was undertaken to underpin the hypothesis of competitive binding to chiral catalyst **6** as key element of the resolution. Computational results indicated a high binding affinity of the cleavage products alkene **7a** and isatin (**10a**). While the latter result could not be experimentally corroborated due to the poor solubility of isatin, the relatively strong association of alkene **7a** was confirmed experimentally by its association constant (cf. Figure 1). Upon cleavage of enantiomer *ent*-**5a**, both isatin (**10a**) and alkene **7a** are formed and potentially block the catalyst, thus avoiding an association of oxetane **5a** and a successful triplet energy transfer. Support for this notion came from the *ee* profile of the kinetic resolution *rac*-**5a** → **5a** in the presence of alkene **7a** (Figure 8).

Under the typical experimental conditions (cf. Scheme 4), the *ee* reached a plateau after 20 min. Addition of various equivalents of alkene **7a** at the very start of the irradiation retarded the reaction, indicating that the olefin inhibits the activity of the catalyst and the latter is not available for energy transfer to the oxetane. Together with the intrinsic preference for the binding of oxetane *ent*-**5a**, the kinetic resolution can be coherently explained.

## Conclusions

In summary, we have been able to decipher the course of a kinetic oxetane resolution facilitated by 5 mol % chiral thioxanthone catalyst **6**. The key step of the resolution is a triplet energy transfer to the oxetane, which induces a [2 + 2] cycloreversion. Substrate enantiomer *ent*-**5** is preferentially processed and delivers fragmentation products resulting from initial C−O (olefins **7** and formaldehyde) or C−C bond scission (1,1-diarylethenes **9** and isatins **10**). The major enantiomer of the resolution, oxetane **5**, is obtained in high *ee*, although transient absorption data suggest that its sensitized cleavage is facile. Evidence has been collected that enantiomer *ent*-**5** displays a higher binding constant to the catalyst and that the binding site of the catalyst is blocked by hydrogen-bonding decomposition products **7** and **10** as the reaction progresses, thus preventing sensitization of the major enantiomer **5**.

Beyond the kinetic resolution, we have identified the cleavage pathways of oxetanes *rac*-**5** and *ent*-**5** depending on the chosen reaction conditions. The compounds fragment from the first excited singlet state (S_1_) into formaldehyde and tetrasubstituted alkenes **7**. The process is extremely fast and occurs without a detectable intermediate by C−O bond scission. It is initiated by direct excitation at a short wavelength (λ_abs_ = 312 nm) in the absence of a sensitizer. In the presence of achiral thioxanthen-9-one (**8**), the high absorption coefficient of the sensitizer (ε > 1000 M^−1^ cm^−1^) in the wavelength region λ = 350−400 nm enforces exclusive excitation of compound **8** even in the presence of oxetanes *rac*-**5**. Triplet energy transfer to the oxetane and internal conversion leads to population of its lowest-lying triplet state T_1_, which was found to cleave exclusively by C−C bond scission. Isatins **10** and 1,1-diarylethenes **9** are the cleavage products. In the environment of chiral thioxanthone **6**, the reactive oxetane isomer *ent*-**5** can undergo a cycloreversion via triplet intermediate T_1_ in full analogy to the achiral case. However, the proximity of the sensitizing unit seems to enable also the population of higher-lying triplet states through which cleavage occurs by initial C−O bond scission.

## Data Availability

The data that support the findings of this study are available in the Supporting Information of this article. Primary research data are openly available in the repository RADAR4Chem at DOI: 10.22000/2nc173tq9xecmy1u.

## Supporting Information Available

Detailed experimental procedures, characterization data for new compounds, NMR titration experiments, time-resolved absorption spectroscopy data, NMR spectra for new compounds, chiral high-performance liquid chromatography (HPLC) traces, coordinates for all computed structures, details and results of the quantum chemical calculations, lowest free energy geometries of the substrate–catalyst species, PES scans for the second bond cleavage, vertical excitation energies, association free energies of the considered photochemical decomposition products, electronic energies of the excited states at the Franck–Condon (FC) point, and electronic energies of the S_0_ and T_1_ states (PDF)
