# Supplementary material for: Oxetane Cleavage Pathways in the Excited State: Photochemical Kinetic Resolution as an Approach to Enantiopure Oxetanes
Source: J Am Chem Soc. 2025 Apr 14;147(16):13893–904. doi: 10.1021/jacs.5c02483 (PMC12022993; doi:10.1021/jacs.5c02483)

Supporting Information

Oxetane Cleavage Pathways in the Excited State: Photochemical Kinetic Resolution as an Approach to Enantiopure Oxetane

Niklas Pflaum,[a] Mike Pauls,[b] Ajeet Kumar,[a] Roger Jan Kutta,[c] Patrick Nuernberger,[c] Jürgen Hauer,[a] Christoph Bannwarth,[b] and Thorsten Bach[a*]

[a] *Department Chemie and Catalysis Research Center (CRC),*

School of Natural Sciences

Technische Universität München,

D-85747 Garching, Germany

[b] *Institut für Physikalische Chemie*

RWTH Aachen University,

D-52074 Aachen, Germany

[c] *Institut für Physikalische und Theoretische Chemie,*

Universität Regensburg

Universitätsstr. 31, D-93053 Regensburg, Germany

Correspondence to: thorsten.bach@tum.de

Table of Contents

1.	General Information	3
2.	Analytical Methods	10
3.	General Synthetic Procedures	12
4.	Computational Studies	16
5.	Transient Absorption Spectroscopy	43
6.	Cyclic Voltammetry	47
7.	Fluorescence Quenching Studies	49
8.	Optimization of the Reaction Conditions	50
9.	Kinetic Profile	51
10.	Selectivity Factors for the Kinetic Resolution	52
11.	Catalyst Inhibition Experiments	53
12.	NMR-Titration Experiments	54
13.	Determination of the Absolute Configuration	63
14.	Analytical Data	66
15.	NMR-Spectra	148
16.	HPLC-Traces	211
17.	Steady-state Absorption Spectra in the UV/Vis Spectral Range	235

1. General Information

All air and moisture sensitive reactions were carried out in flame-dried glassware under overpressure of argon gas using standard *Schlenk* techniques.

All commercially available chemicals were used without further purification, if not stated otherwise. For moisture sensitive reactions, dichloromethane (CH_2Cl_2), diethylether (Et_2O) and tetrahydrofuran (THF) were purified using a *MBraun* MBSPS 800 solvent purification system. The following columns were used:

CH_2Cl_2:	2 × MB-KOL-A type (aluminum oxide)
Et_2O:	1 × MB-KOL-A type (aluminum oxide)
	1 × MB-KOL-M type 2 (3Å molecular sieve)
THF:	2 × MB-KOL-M-type 2 (3Å molecular sieve)

Anhydrous *N*,*N*-dimethylformamide (DMF, supplied over 3Å molecular sieves) was purchased from *Acros Organics*, α,α,α-trifluorotoluene (TFT) from *Sigma Aldrich* (*Merck*) and α,α,α,α′,α′,α′-hexafluoro-*m*-xylene (HFX, 98%) from *TCI Chemicals*.

Technical solvents for column chromatography [dichloromethane (CH_2Cl_2), ethylacetate (EtOAc), methanol (MeOH), acetone (Ac), hexanes (Hex), *n*-pentane (Pen)] were used after simple distillation.

Normal phase flash column chromatography was performed on silica 60 *(Merck*, 230-400 mesh) using the indicated eluent mixtures.

Photochemical reactions at λ_{max} = 425 nm were performed in a standard round-bottom flask under an argon atmosphere using an LED (see detailed setup information below).

Photochemical reactions at λ_{max} = 398 nm were performed in a *Duran* glass tube (see detailed setup information below) under an argon atmosphere using a LED. Reactions that required cooling were performed in a dewar vessel attached to a cryostat.

Solvents used in photochemical reactions were usually degassed by three cycles of freeze-pump-thaw (2.1×10^{-3}mbar) or by sparging the solution with argon while ultrasonicating for 15 minutes.

Datasheet LED065 LUXEON Z 420nm

Basic Information

Type	High-Power-LED	
Description	Luxeon Z 420 nm on a Saber Z5 Base-Plate	
Manufacturer / Supplier	Philips Lumileds / Luxeonstar	
Order number / Date of purch.	n/a / 03/2018	
Internal lot / serial number	2018-03 / LED065	

Specification Manufacturer

Type / size	4 emitters / ca. 1 x 1 mm	
Mechanical specification		
Electrical specification	700 mA, UF 12.2 V	
Wavelength (range, typ.)	420-425 nm, typ. n/a	
Spectral width (FWHM)	n/a	
Datasheet	LuxeonZUV.pdf	

Characterization

Description of measurement	Measured with Ocean-optics USB4000 spectrometer using a calibrated setup (cosine corrector/fibre). The distance between the emitting surface and the surface of the cosine corrector was 20 mm. The LED was operated at 700 mA on a passive heat-sink at approx. 20 °C	
Measured dominant wavelength / Int.	425 nm	13219 µW/mm²nm
Measured spectral width (FWHM)	15 nm	
Integral Reference intensity / range	243039 µW/cm²	350-500 nm

Spectrum

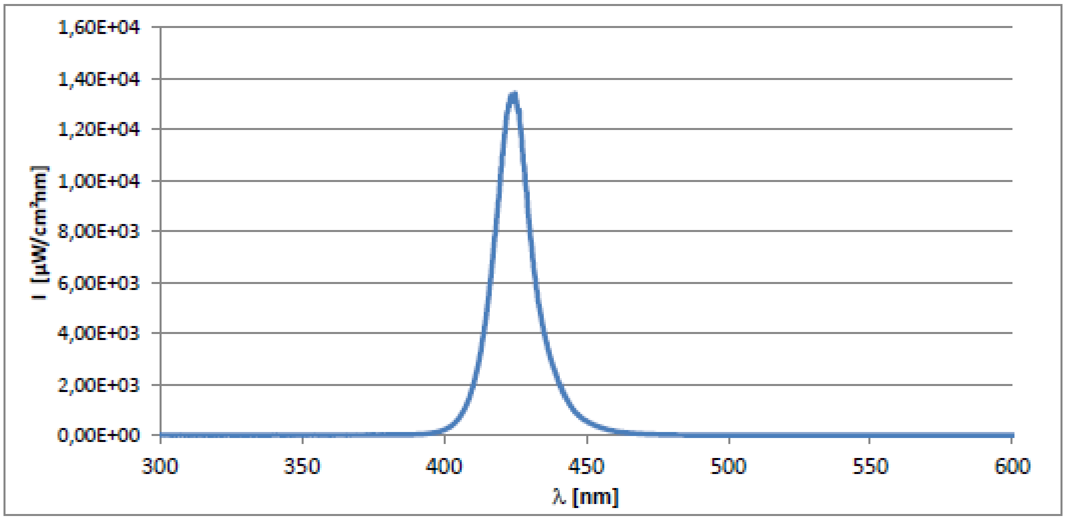

Datasheet LED026 400 / 10 W

**Basic Information**	Ultra-High-Power Violet (400)
Type	High-Power-LED
Description	
Manufacturer / Supplier	LED-Engine Mouser
Order number / Date of purch.	LZ4-40UA00-00U6 / 01/2016
Internal lot / serial number	2016-01 / LED026
**Specification Manufacturer**	
Type / size	quattro emitter / not spec.
Mechanical specification	
Electrical specification	700 mA @15 V
Wavelength (range, typ.)	
Spectral width (FWHM)	
Datasheet	LZ4-00UA-series.pdf
**Characterization**	
Description of measurement	Measured with Ocean-optics USB4000 spectrometer using a calibrated setup (cosine corrector/fibre). The distance between the emitting surface and the surface of the cosine corrector was 20 mm. The LED was operated at 500 mA on a passive heat-sink at approx. 20 °C
Measured wavelength	398 nm
Measured spectral width	15 nm
Integral Reference intensity	317017 µW/cm² (360-480 nm @ 20 mm distance, 4 mmcosine corr.)

Spectrum

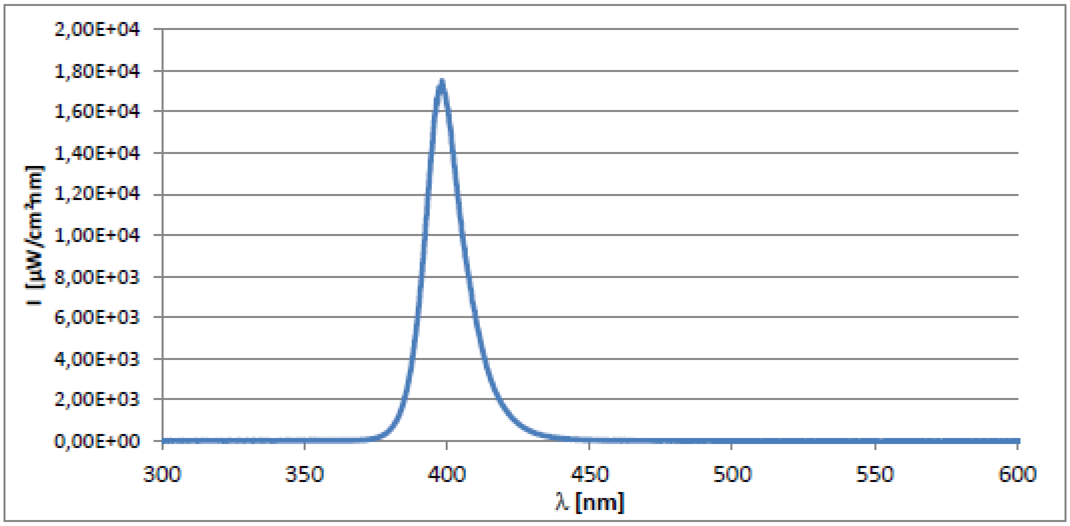

Datasheet LED041 366 / 3W

Basic Information

Basic Information	High-Power UV-A-LED
Type	High-Power-LED
Description	
Manufacturer / Supplier	Mouser
Order number / Date of purch.	LZ1-10UV00-0000 / 06/2016
Internal lot / serial number	2016-01 / LED041

Specification Manufacturer

Type / size	matrix emitter - not spec. / not spec.
Mechanical specification	
Electrical specification	700 mA @3.7 V
Wavelength (range, typ.)	
Spectral width (FWHM)	
Datasheet	LZ1-00UV-series.pdf

Characterization

Description of measurement	Measured with Ocean-optics USB4000 spectrometer using a calibrated setup (cosine corrector/fibre). The distance between the emitting surface and the surface of the cosine corrector was 20 mm. The LED was operated at 700 mA on a passive heat-sink at approx. 20 °C
Measured wavelength	368 nm
Measured spectral width	11 nm
Integral Reference intensity	176704 µW/cm² (350-425 nm @ 20 mm distance, 4 mmcosine corr.)

Spectrum

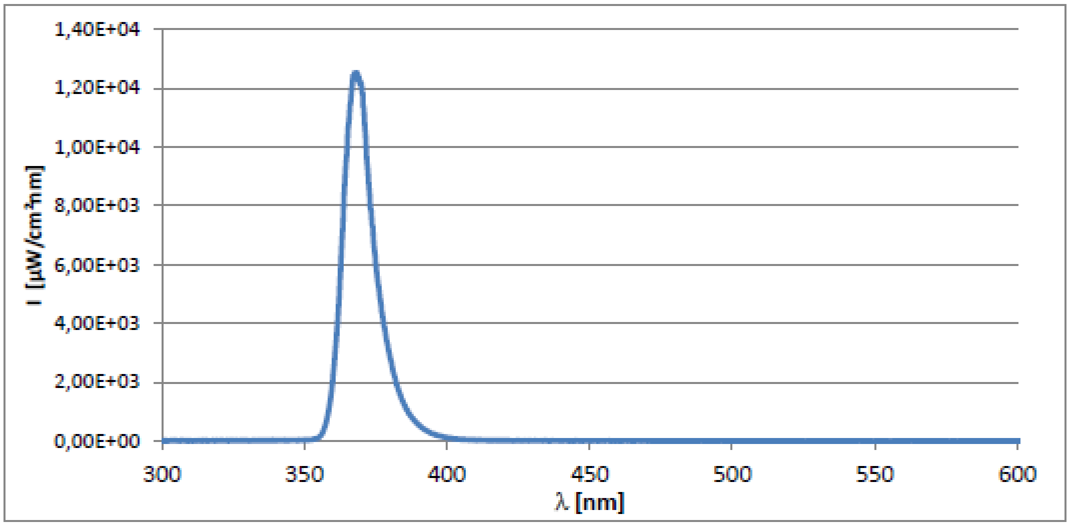

Datasheet FLT022 LZC-420

Basic Information

Type	Fluorescent light tube
Description	Luzchem LZC-420
Manufacturer / Supplier	n/a / Luzchem
Order number / Date of purch.	n/a / 07/2017
Internal lot / serial number	2017-07 / FLT022

Specification Manufacturer

Type / size	T5 tube, G5 socket
Mechanical specification	16 mm diameter, 288 mm length
Electrical specification	8 W
Wavelength (range, typ.)	400 - 440 nm
Spectral width (FWHM)	~ 30 nm
Datasheet	LES-420-016

Characterization

Description of measurement	Measured with Ocean-optics USB4000 spectrometer using a calibrated setup (cosine corrector/fibre). The cosine corrector was placed at 20 mm distance from a single fluorescent tube at half height.	
Measured dominant wavelength / Int.	421 nm	121 µW/mm²nm
Measured spectral width (FWHM)	30 nm	
Integral Reference intensity / range	4142 µW/cm²	350-500 nm

Spectrum

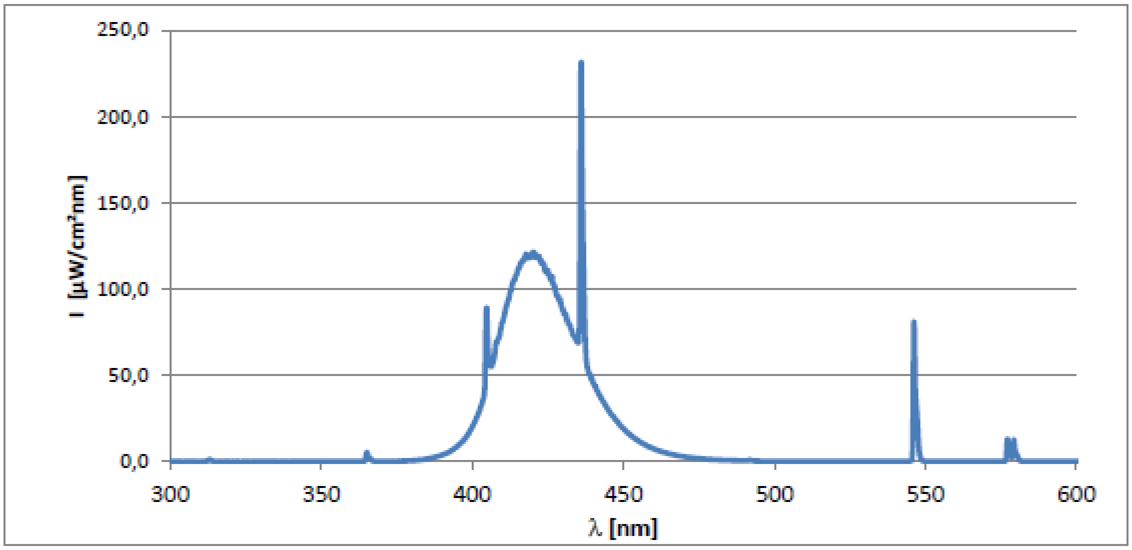

LED – Irradiation setups

For all synthetic irradiation experiments within this publication, two LED setups were used. 1. Setup: For the synthesis of oxetanes by the *Paternò-Büchi* reaction, an LED (λ_{max} = 425 nm) was connected to a glass rod acting as an optical fiber that is attached to any standard laboratory glassware. Since these reactions were carried out at ambient temperature, a water bath was used for keeping a constant temperature.

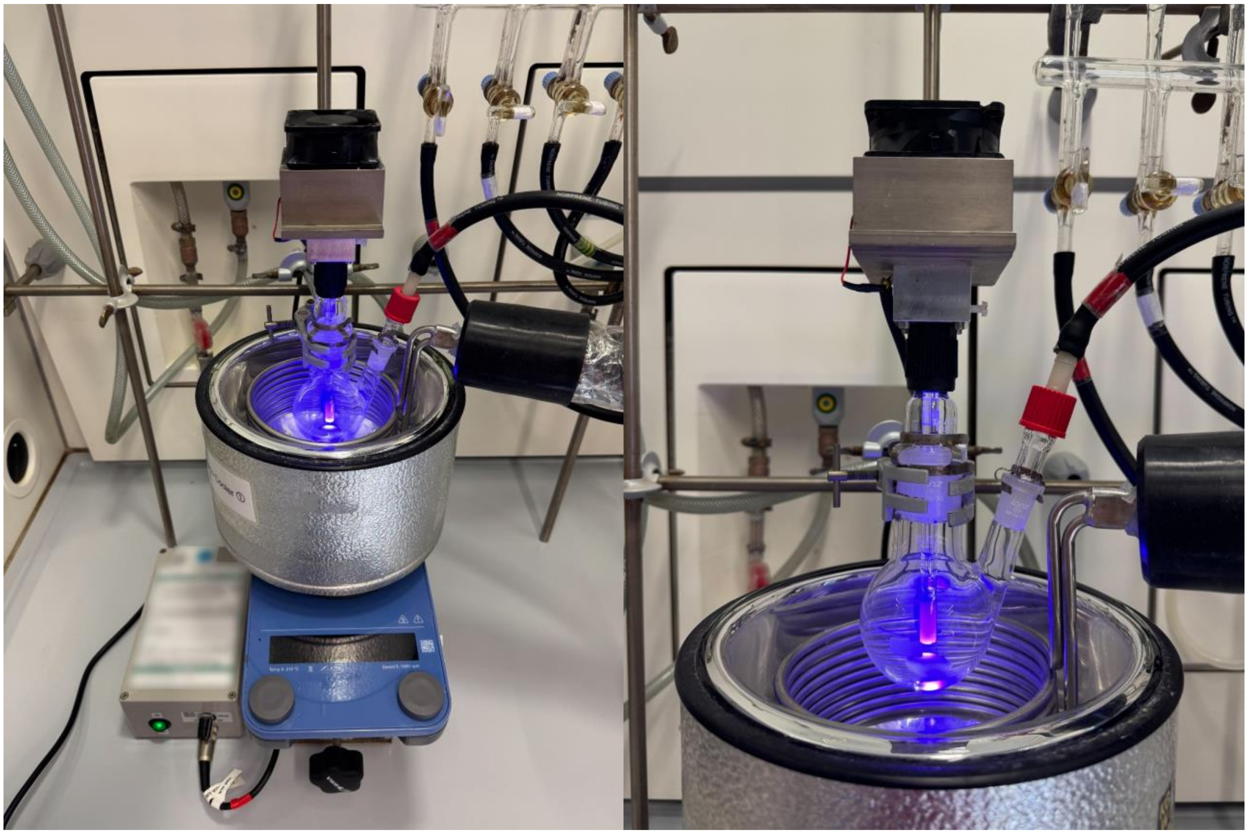

2. Setup: For all kinetic resolution experiments, an LED (λ_{max} = 398 nm) was connected to a glass rod acting as an optical fiber that can be attached to *Schlenk* phototubes. To cool the reaction solution, the tube is immersed in a dewar vessel cooled by an external cryostat.

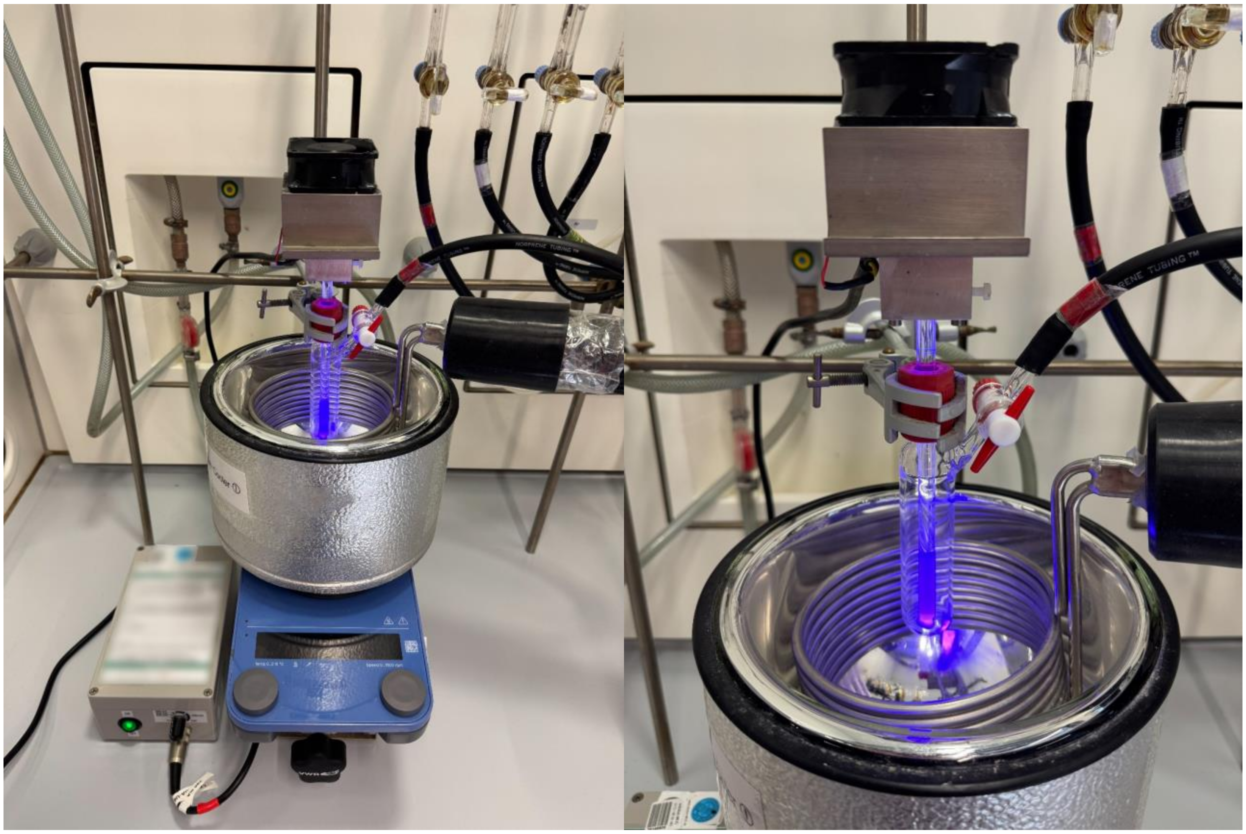

2. Analytical Methods

Thin layer chromatography (TLC) was performed on silica coated glass plates (silica ge 60 F_{254}) with detection by UV-light (λ = 254 nm) or potassium permanganate stain [$KMnO_4$]

Infrared spectra (IR) were recorded on a JASCO IR-4100 or a Perkin Elmer Frontier IR-FTR spectrometer by ATR technique. The signal intensity is assigned using the following abbreviations s (strong), m (medium), w (weak).

Melting points (M.p.) were determined using a *Kofler* ("Thermopan", Fs *Reichert*) apparatus.

Nuclear magnetic resonance (NMR) (^{1}H, ^{13}C, and ^{19}F-NMR) spectra were recorded at room temperature on either a Bruker AVHD-300, AVHD-400, AVHD-500, or a Bruker AV-II-500 equipped with cryo probe head. Chemical shifts of the NMR spectra are reported relative to $CDCl_3$, (^{1}H-NMR: δ = 7.26 ppm, ^{13}C-NMR: δ = 77.16 ppm), benzene-d_6 (^{1}H-NMR: δ = 7.16 ppm, ^{13}C-NMR: δ = 128.39 ppm), or DMSO-d_6 (^{1}H-NMR: δ = 2.50 ppm, ^{13}C-NMR: δ = 39.52 ppm). The data are reported as follows: chemical shift (δ) [multiplicity, coupling constant *J* (Hz), number of protons, nucleus] where multiplicity is defined as: m = multiplet, s = singlet, d = doublet, t = triplet, q = quartet, quint. = quintet, sext. = sextet, sept. = septet, br = broad. Apparent multiplets which occur as a result of coupling constant equality between magnetically non-equivalent protons are marked as virtual (*virt.*).

Mass spectrometry (MS) and high-resolution mass spectrometry (HRMS) were performed by electron spray ionization (ESI) recorded on a *Waters* Thermo Scientific LTQ-FT Ultra or a Synapt XS High Resolution MS.

Specific Rotation was determined using an ADP440+ polarimeter (Fa *Bellingham+Stanley*) and is reported as follows: $[a]_D^T$ (c in g per 100 mL solvent).

High performance liquid chromatography (HPLC): For normal phase HPLC, ChiralPak AD-H (250 x 4.6 mm) or Chiralpak AS-H (250 x 4.6 mm) (*Daicel Chemical Industries*) columns were used. Isocratic elution was performed with *n*-heptane and isopropanol (^{i}PrOH). For reversed-phase HPLC, Chiralcel OD-RH and Ciralcel OJ-RH (*Daicel Chemical Industries*) columns were used as the chiral stationary phase and a mixture of acetonitrile/water was used as mobile phase.

For normal and reversed phase HPLC analytics, a UVD 340 photodiode array detector, a P580 pump and an ASI-100 automated sample injector were used at 20 °C.

Cyclic Voltrammetry (CV) was conducted on a *Metrohm* Autolab PGSTAT204 together with a glassy carbon working electrode (*Palmsens*, IS-3MM-GC-W, d = 3 mm) and platinum wire (*ADVENT*, PT541407) as the counter electrode. As a reference electrode an $AgNO_3$/Ag (*Goodfellow*, AG005150/27, 0.01 M $AgNO_3$ und 0.10 M $[N(^nBu)_4]PF_6$ in MeCN) system was used separated from the analyte by a *Vycor* frit. All measurements were conducted in a five-neck flask under argon atmosphere. All potentials are given against SCE. For reference, ferrocene ($E_{\frac{1}{2}(Fc^+/Fc)}$ = 74 mV in MeCN against $AgNO_3$/Ag) was used as an internal standard to convert the measured potentials to SCE. ($AgNO_3$/Ag to SCE = +298 mV in MeCN)[1]. All samples were prepared in flame dried flasks under argon atmosphere in water free MeCN with an analyte concentration of c = 1.0 mM and $[N(^nBu)_4]PF_6$ as the electrolyte (c = 0.1 M). Prior to measurement, all samples were degassed by three cycles of freeze pump thaw.

References for this section

(1) J. Hofer, F. Pecho, T. Bach, *Synlett* **2023**, *34* (09), 1063-1067.

3. General Synthetic Procedures

General Procedure A (GP-A)

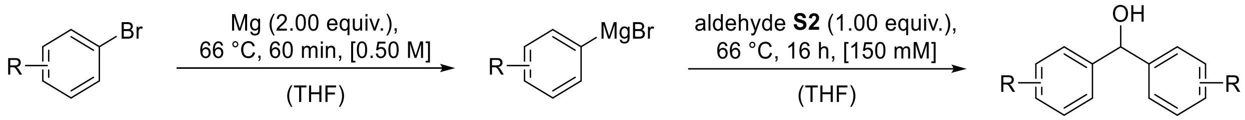

S1
1.50 equiv.

S3

Preparation of Grignard reagent:

Magnesium shavings (2.00 equiv.) were activated with a small grain of iodine and stirred without solvent. After the addition of 5 mL of THF, about 10% of the corresponding aryl bromide was added in one portion to start the reaction. If necessary, the reaction was briefly heated. After successful start of the reaction, the remaining THF (calculated for a final *Grignard* reagent concentration of 0.50 M) was added, followed by the dropwise addition of the corresponding aryl bromide (1.50 equiv.) **S1**. After complete addition, the reaction mixture was refluxed for 60 min and then cooled to room temperature.

Grignard addition:

A solution of the corresponding aldehyde (1.00 equiv.) **S2** in THF [150 mM] is cooled to 0 °C and the freshly prepared *Grignard* reagent (1.50 equiv.) in THF [0.50 M] is added dropwise. After the complete addition, the ice bath was removed and the reaction mixture was refluxed for 16 h. After complete aldehyde conversion, the reaction is quenched by the addition of 1 M HCl. The resulting mixture is extracted with EtOAc (3 × 100 mL per 1.00 g aldehyde) and washed with sat. aq. $NaHCO_3$ solution (3 × 100 mL per 1.00 g aldehyde) and brine. After drying over Na_2SO_4 and filtration, all volatiles were removed under reduced pressure and the corresponding alcohol **S3** was purified by flash column chromatography.

General Procedure B (GP-B)

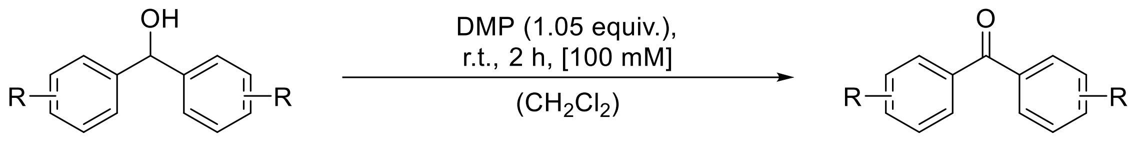

For oxidation, the corresponding alcohol **S3** (1.00 equiv.) was dissolved in CH_2Cl_2 [100 mM] and stirred at room temperature. DMP (1.05 equiv.) was added in one portion and the resulting solution was stirred for 2 h. After complete conversion, water (50 mL per 500 mg alcohol **S3**) was added and the mixture was extracted with CH_2Cl_2 (3 × 100 mL per 500 mg alcohol), washed with brine (100 mL) and dried over Na_2SO_4. After filtration, the solvent was removed under reduced pressure and the crude benzophenone **S4** was purified by flash column chromatography.

General Procedure C (GP-C)

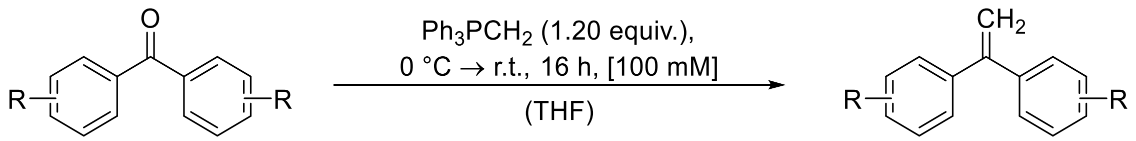

For methylenation of the corresponding benzophenone **S4**, methyltriphenylphosphoniumbromide (1.20 equiv.) was suspended in THF [50.0 mM] and cooled to 0 °C. *n*-BuLi solution (1.20 equiv., 2.50 M in hexanes) was added dropwise at this temperature and the resulting orange solution was stirred at 0 °C for 60 min. Benzophenone **S4** was dissolved in THF [50.0 mM] and subsequently added to the reaction mixture at 0 °C. The reaction mixture was warmed to room temperature and stirred for 16 h. After complete conversion, the reaction was quenched with sat. aq. NH_4Cl solution (50 mL) and extracted with hexanes (5 × 75 mL per 500 mg ketone). The combined organic layers were washed with brine (100 mL), dried over Na_2SO_4 and filtered. Any volatiles were removed under reduced pressure and the crude material was purified by flash column chromatography.

Gerneral Procedure D (GP-D)

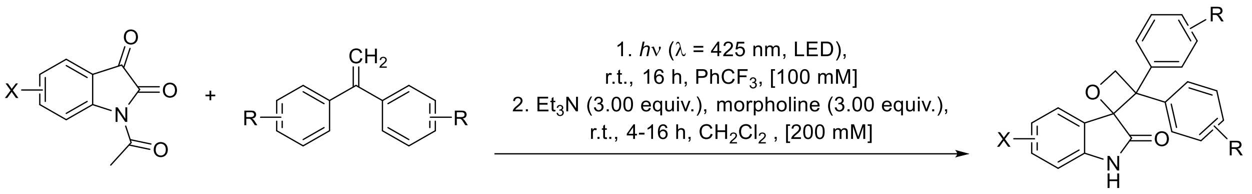

S5 9 *rac*-**5**

[2+2]-Photocycloaddition (PCA) – Paternó-Büchi reaction*:*

For photocycloaddition the corresponding *N*-acetylated isatin **S5** (1.00 equiv.) and olefin **9** (3.00 equiv.) were dissolved in $PhCF_3$ [100 mM] and degassed for 15 min by sparging the reaction mixture with argon while ultrasonication. The bright yellow solution was irradiated with an LED (λ_{max} = 425 nm) using the previously described irradiation setup for [2+2]-PCA reactions until complete conversion of *N*-acetylated isatin **S5**. The solvent was removed under reduced pressure and the crude product was used directly for deprotection.[1]

Acetyl deprotection:

The crude material synthesized by [2+2]-PCA was taken up in CH_2Cl_2 [200 mM] and treated with Et_3N (3.00 equiv.) and morpholine (3.00 equiv.) at room temperature. After stirring for 4-16 h and after complete conversion of the acetyl protected oxetane, all volatiles were removed under reduced pressure and the crude oxetane **5** was purified by flash column chromatography.

General Procedure E (GP-E)

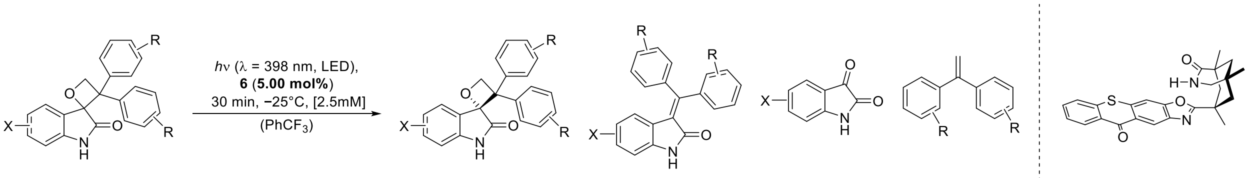

rac-**5** **5** **7** **10** **9** **6**

For the kinetic resolution of spiro oxetane indolones, 50.0 µmol of the corresponding oxetane *rac*-**5** and 2.50 µmol thioxanthone **6** (5.00 mol%, synthesized analogously to literature)[2] were dissolved in TFT [2.50 mM]. The clear solution was degassed (3 × freeze pump thaw) and cooled to −25 °C for 30 min. Using the irradiation setup as described for kinetic resolution experiments (*vide supra*), the reaction was irradiated using an LED (λ_{max} = 398 nm) for 30 min. After irradiation, the solvent was removed under reduced pressure and the crude mixture was directly purified by

flash column chromatography (CH_2Cl_2:Ac = 100:0 → 90:10). Due to similar retention times on column, the enantiomerically enriched oxetane **5** and fragmentation product **7** were isolated as a combined fraction and their ratios were determined by crude ^{1}H-NMR. Olefin **9** and isatin **10** were isolated as pure fractions. To prove synthetic relevance, the photocatalytic kinetic resolution of model substrate *rac*-**5a** was additionally performed on a 1.00 mmol scale. Here, the effort was undertaken to separate the two products **5a** and **7a**. This result turned out to be identical to the small-scale reaction (more detailed information see below).

General Procedure F (GP-F)

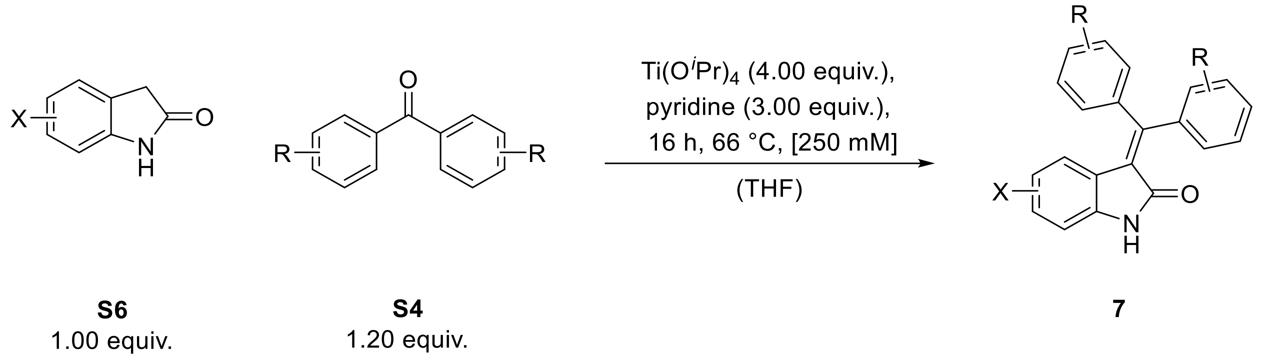

The condensation of reference compounds **7** was performed analogously to literature[3] by dissolving oxindole **S6** (1.00 equiv.) and benzophenone **S4** (1.20 equiv.) in THF [250 mM] at room temperature. Pyridine (3.00 equiv.) and Ti(O^{i}Pr)$_4$ (4.00 equiv.) were added dropwise and a rapid change in color to dark orange was observed. The reaction mixture was heated to 66 °C and stirred for 16 h. After complete conversion, water (100 mL) was added and the mixture was extracted repeatedly with EtOAc until the aqueous layer was colorless. The combined organic phases were dried with brine and Na_2SO_4 and filtered. Any volatiles were removed under reduced pressure and the crude product was purified by flash column chromatography.

References for this section:

(1) J. Xue, Y. Zhang, T. Wu, H.-K. Fun, J.-H. Xu, *J. Chem. Soc., Perkin Trans. 1* **2001**, 183–191.
(2) R. Alonso, T. Bach, *Angew. Chem. Int. Ed.* **2014**, *53*, 4368–4371.
(3) H. J. Lee, J. W. Lim, J. Yu, J. N. Kim, *Tetrahedron Lett.* **2014**, *55*, 1183–1187.

4. Computational Studies

The computational investigation started with the enantiomeric oxetane species **5a**, *ent*-**5a**, and the chiral thioxanthone catalyst **6** whose optimized ground state geometries are illustrated in Figure S1.

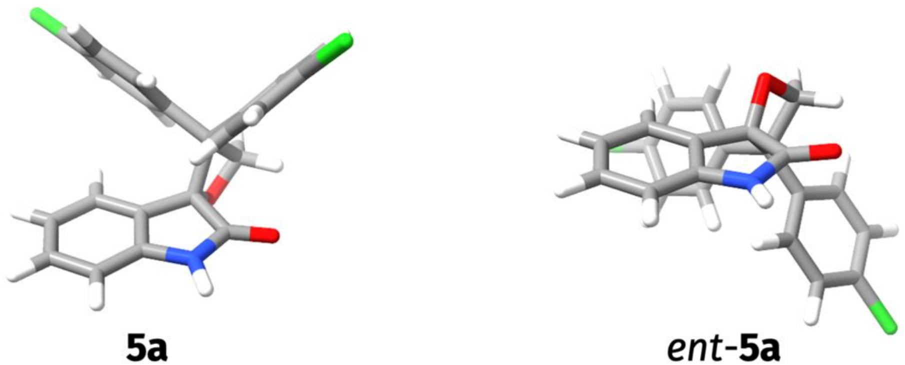

5a

ent-**5a**

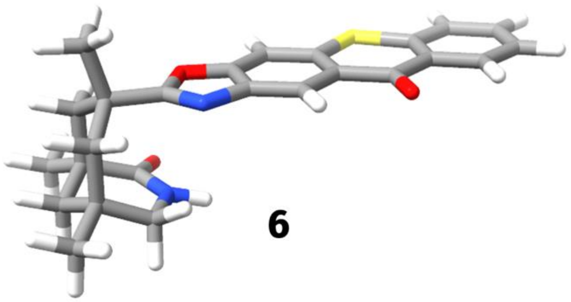

6

Figure S1: S_0 structures of the enantiomeric oxetane substrates **5a**, *ent*-**5a**, and the thioxanthone photocatalyst **6** optimized using the PBEh-3c density functional theory composite method.

The initial geometries were pre-optimized using the semiempirical electronic structure theory method GFN2-xTB.[1] Using the ”low-cost” density functional theory (DFT) method PBEh-3c[2], these geometries are reoptimized. PBEh-3c is a composite method utilizing a modified def2-SVP[3] basis set (def2-mSVP) and a global hybrid functional based on the functional of Perdew-Burke-Ernzerhoff (PBE)[4] with 42% non-local Fock-exchange. A geometrical counter-poise correction (gCP)[5] and the D3 dispersion correction[6,7] are employed such that the basis set superposition error (BSSE) and dispersion interactions are accounted for.

To derive meaningful Gibbs free energies, representative conformer ensembles (CE) for all species considered are generated with the aim of identifying global minima. We perform metadynamics (MTD) simulations using the default settings of the Conformer Rotamer Ensemble Sampling Tool (CREST)[8,9] (version 2.11.3) in combination with GFN2-xTB as implemented in xtb (version 6.5.1).[10]

For the generation of noncovalent complex geometries, we first computed localized molecular orbitals (LMO) for the conformers of the involved species using GFN1-xTB.[11] These LMOs are then used to construct the intermolecular force-field approach xTB-iFF[12], which is used to associate rigid fragments.

All structures obtained in this way are reoptimized with GFN2-xTB in CREST (keyword ”mdopt”). Duplicates and energetically high-lying geometries are removed from the CE with the CREGEN routine in CREST. Here, an energy window of 3 kcal mol^{-1} with respect to the lowest conformer was chosen. The truncated ensemble is again optimized with PBEh-3c. Thermochemical properties are calculated on these geometries as described in the following.

Calculation of Free Energies

The free energy G_i of species *i* may be computed as:

$$G_i = E_{\mathrm{el},i} + G_{\mathrm{TRV},i} + \delta G_{\mathrm{solv},i} \quad \text{(Eq. 1)}$$

Electronic gas phase energies $E_{\mathrm{el},i}$ here refer to the ωB97X-D4[13,14]/def2-QZVPP[3]//PBEh-3c level of theory. The rigid rotor and modified harmonic oscillator (mRRHO)[15] model is used to account for translational, rotational and vibrational (TRV) contributions to the thermal free energy $G_{\mathrm{TRV},i}$ as well as zero point vibrational energy (ZPVE). Harmonic frequencies based on the PBEh-3c geometries (scaled by 0.95[2,16]) are used throughout.

A standard state solvation correction $\delta G_{\mathrm{solv},i}$ for the conversion of 1 mol of gas at 1 bar to a 1 M solution is calculated with GFN2-xTB based on the PBEh-3c geometries. For this step, the analytically linearized Poisson-Boltzmann (ALPB) model[17] for dichloromethane (DCM) is chosen since the dielectric constant is similar to the experimentally used solvent α,α,α-trifluorotoluene, which is not parameterized for the ALPB model. In Table S1, the methods used to assess the thermodynamic quantities are summarized.

Table S1: Employed levels of theory for free energy calculations in this computational study.

Geometry optimizations	PBEh-3c
Electronic energies $E_{\mathrm{el},\,i}$	ωB97X-D4/def2-QZVPP
Nuclear contributions to the free energy $G_{\mathrm{TRV},\,i}$	PBEh-3c harmonic frequencies (scaled with 0.95); particle-in-a-box, rigid rotor and modified harmonic oscillator model to account for translational, rotational and vibrational contributions to the nuclear thermal free energy. Harmonic frequencies below 50 cm^{-1} are handled by the interpolated free rotor–harmonic oscillator model.
Solvation free energy correction $\delta G_{\mathrm{solv},\,i}$	$\delta G_{\mathrm{solv},\,i} = G_{\mathrm{GFN2xTB/ALPB(DCM)},\,i} - E_{\mathrm{GFN2xTB/gas},\,i}$ based on PBEh-3c geometries. This standard state correction is calculated for 1 mol of gas at 1 bar compared to a 1 M solution (keyword 'bar1M' in xtb).

For the excited states (ES) S_1 and T_1, the contributions $G_{\mathrm{TRV},i}$ and $\delta G_{\mathrm{solv},i}$ are described with an open-shell GFN2-xTB configuration (keyword "uhf 2"). The approximated open-shell ES singlet/triplet states are indistinguishable at the GFN2-xTB level of theory, because the GFN2 Hamiltonian does not include spin-discriminating terms.[1,18]

Throughout the computational studies, the presented relative energy differences are based on the conformers showing the lowest Gibbs energy within a CE. The corresponding thermodynamic data is provided below in Table S7. All geometries and relevant input/output files for the computations are provided in the "Computational_Studies" folder within the published repository. Further technical details on the computational methodology are outlined in the following.

Technical Details

- Ground state geometry optimizations are performed with the ORCA program (version 5.0.3)[19] addressing the GPU-accelerated electronic structure code TeraChem[20,21] for energy and gradient computations (keyword "extopt" in ORCA). We use a development version of TeraChem (based on version 1.9.2) featuring, among others, the 3c-composite method PBEh-3c.[22] The self-consistent field (SCF) settings for the TeraChem single-point runs (S_0) are tightened and a mixed precision formalism was used throughout.[23]

- Unrestricted Kohn Sham (UKS-)DFT T_1 calculations were conducted using the ORCA program as standalone.
- Electronic energies (ωB97X-D4/def2-QZVPP//PBEh-3c) are computed with ORCA using the resolution-of-the-identity (RI-J)[24,25] approximation with the auxiliary basis (def2/J)[26] for the evaluation of the Coulomb integrals and the chain of spheres for exchange (COSX)[27] approximation. As quadrature grid for numerical integrations, the 'defgrid3' option is used.

Association Energies

In Table S2, association energies of the oxetane enantiomers to **6** and corresponding dimerization energies at $T = -25$ °C (248.15 K) are presented.

Table S2: Free energy differences (T = 248.15 K) for the association of oxetanes **5a** and *ent*-**5a** to photocatalyst **6** and dimerization energies. Electronic energies are computed at the ωB97X-D4/def2-QZVPP//PBEh-3c theory level.

Species	ΔG (kJ mol^{-1})
Catalyst Association	
**5a** + **6**	−22
ent-**5a** + **6**	−41
2 × **6**	−20
Oxetane Association	
2 × **5a**	−22
ent-**5a** + **5a**	−24

A distinct binding affinity of the oxetane enantiomers **5a** and *ent*-**5a** to form **5a·6** and *ent*-**5a·6** is observed. The dimerization of the oxetane competes with the formation of these species. With −24 kJ mol^{-1}, the association of **5a** and *ent*-**5a** is even larger than the binding of **5a** to **6** (−22 kJ mol^{-1}). However, *ent*-**5a** favorably associates to the catalyst (−41 kJ mol^{-1}). The large binding affinity to form *ent*-**5a·6** surpasses association of both oxetane enantiomers, resulting in an effective association energy of:

$$\Delta G_{\text{eff}} = -41\ \text{kJ mol}^{-1} - \frac{1}{2}(-20\ \text{kJ mol}^{-1} - 24\ \text{kJ mol}^{-1}) = -19\ \text{kJ mol}^{-1}$$

An association free energy of +0 kJ mol^{-1} is found for **5a**. This difference explains to some degree the high enantiomeric excesses (*ee*) obtained in the reaction. The competitive inhibition due to

photoproducts further prevents the conversion of **5a** (see below). In Figure S2, the complex geometries of **5a·6** and *ent*-**5a·6** are presented.

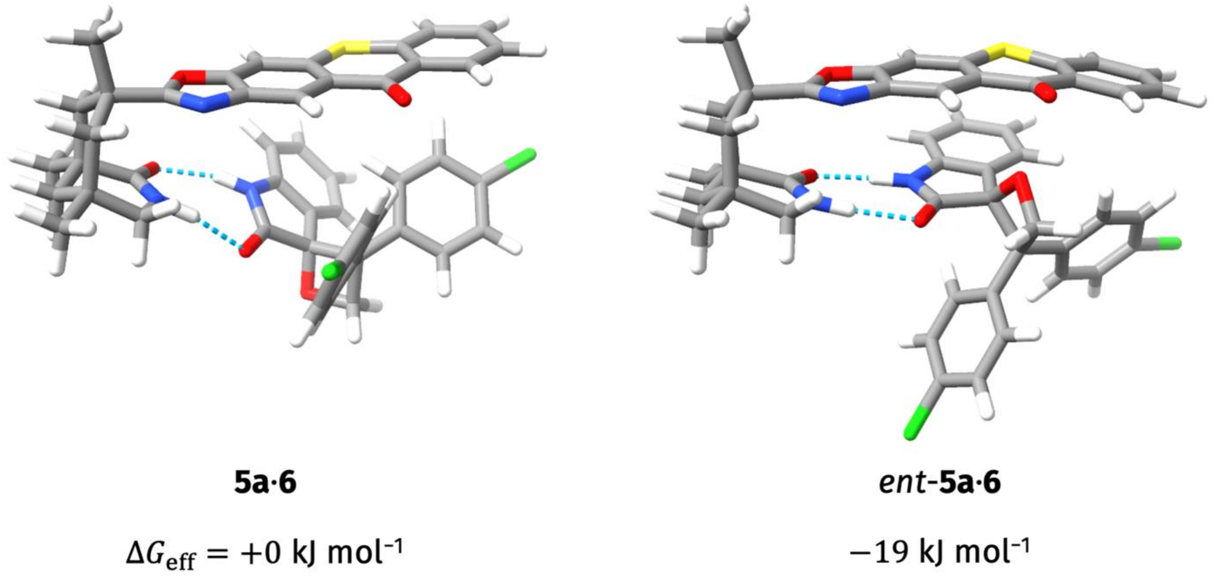

Figure S2: Lowest free energy geometries of the substrate–catalyst species optimized with PBEh-3c. Both oxetane enantiomers coordinate to photocatalyst **6** *via* two hydrogen bonds. The hydrogen bonding interaction (shown with dashed blue lines) to form **5a·6** is sterically less feasible as evident from the computed association free energies (Tab. S2) and the shown ground state geometry. The association free energy relative to the dimers ΔG_{eff} amounts to +0 kJ mol^{-1} (**5a·6**) and −19 kJ mol^{-1} (*ent*-**5a·6**).

Upon irradiation with light, bond scission in the oxetane ring and subsequent decomposition is observed. In Figure S3, the investigated reaction pathways *via* intermediates **5a1** and **5a2** to the respective decomposition products are outlined.

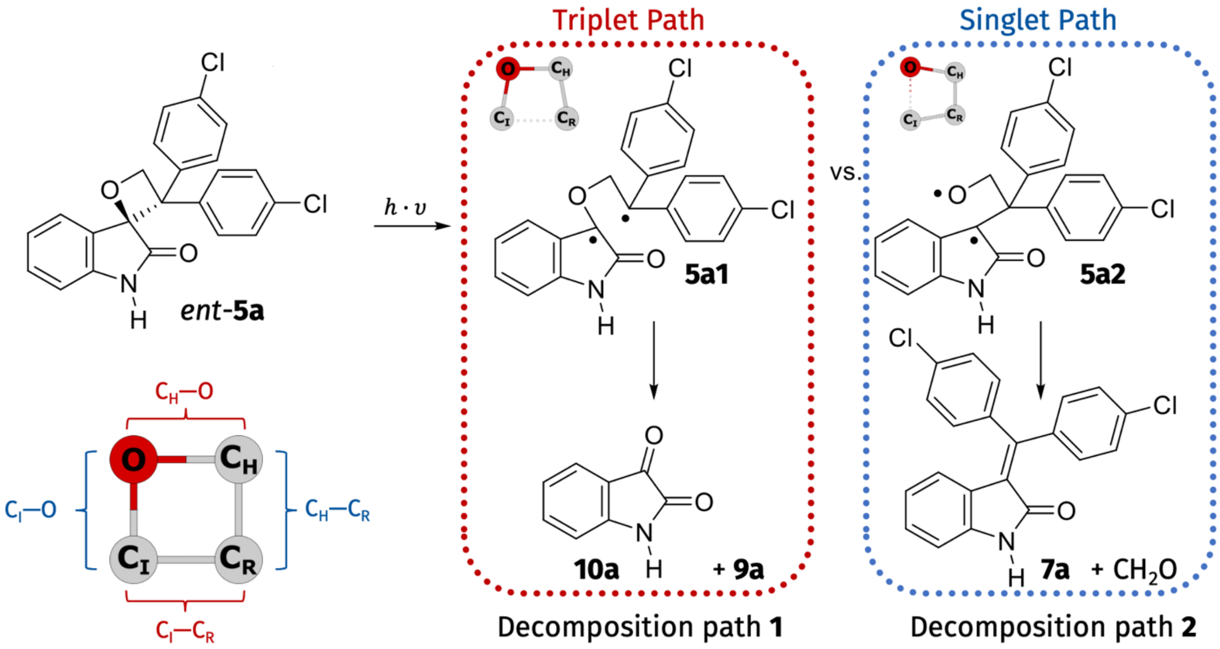

Figure S3: Studied decomposition pathways of *ent*-**5a**. Photochemically induced decomposition of *ent*-**5a** to **10a** and **9a** (red box) can proceed *via* carbon–carbon (C_I—C_R) bond scission to intermediate **5a1**. The tetra-substituted olefin **7a** and CH_2O are formed *via* carbon–oxygen bond scission (C_I—O) to species **5a2** (blue box). Pictograms for the connectivity and nomenclature of the C—C and C—O bonds of the oxetane ring are given. The identified multiplicities for the photochemical pathways are indicated and explained in the following. Triplet pathways of *ent*-**5a** are only feasible in presence of **6** or **8** (see main article), and decomposition path 2 can proceed *via* a triplet as well.

Catalyst Deactivation

Decomposition products may also associate to **6**, inhibiting the photocatalytic decomposition of oxetane. To clarify whether the association of decomposition products (Fig. S3) to **6** is feasible, we computed the corresponding association free energies (Tab. S3). Since dimerization effects are found to be relevant to obtain meaningful association energies, these are also taken into consideration for the decomposition products.

Table S3: Association free energies of the considered photochemical decomposition products (**7a**, **9a**, **10a**, CH_2O) computed using ωB97X-D4/def2-QZVPP//PBEh-3c electronic energies. Dimerization energies are given as well. All values refer to a thermodynamic temperature of 248.15 K.

Species	ΔG (kJ mol^{-1})
Catalyst Association	
**9a** + **6**	−6
**10a** + **6**	−35
**7a** + **6**	−40
CH_2O + **6**	−4
Oxetane Association	
**9a** + **5a**	+0
**10a** + **5a**	−24
**7a** + **5a**	−25
CH_2O + **5a**	−7
Dimerization	
2 × **9a**	+2
2 × **10a**	−22
2 × **7a**	−15
2 × CH_2O	−2

7a (second pathway) and **10a** (first pathway) show similar association energies to the catalyst with $\Delta G = -40$ kJ mol^{-1} and −35 kJ mol^{-1}, respectively. Due to their planarity and the structural motif to form hydrogen bonds with **6**, the complex formation is quite exergonic. The association free energy is similar to *ent*-**5a** (−40 kJ mol^{-1}), where the sterically demanding substituent is oriented away from the thioxanthone moiety of **6**. The complementary decomposition products of the respective pathways, **9a** (−6 kJ mol^{-1}) and formaldehyde (−4 kJ mol^{-1}) without suitable hydrogen bond donor/acceptor sites only show small exergonic association energies.

Taking into consideration dimerization, we can compute effective association energies as described above. For **10a**·**6** we obtain $\Delta G = -35 \text{ kJ mol}^{-1} - \frac{1}{2}(-22 + -20) \text{ kJ mol}^{-1} = -14 \text{ kJ mol}^{-1}$

whereas the free energy equals $\Delta G = -40\ \mathrm{kJ\ mol^{-1}} - \frac{1}{2}(-15 + -20)\ \mathrm{kJ\ mol^{-1}} = -22\ \mathrm{kJ\ mol^{-1}}$ for **7a·6**. These results suggest that inhibition of the catalyst will occur if sufficient amount of **7a** or **10a** are present, since this binding affinity exceeds the value determined for *ent*-**5a** and more so for **5a**.

Potential Energy Surface Scans

As demonstrated in Figure 6 in the main article, potential energy surface (PES) scans were performed along the reaction coordinates of the first bond scission of the cleavage pathways of *ent*-**5a** (Fig. S3). These scans help understanding whether bond scission towards **5a1** and **5a2** can occur *via* a singlet or triplet path, since it can be assumed that one of these channels is favoured due to a lower relative energy with respect to S_0. Furthermore, the PES scans allow to estimate whether the ring opening involves a meaningful energetic barrier.

Throughout this work, partially relaxed scans on the PES (denoted "PES scan" for brevity) were performed at the GFN2-xTB theory level using an open-shell configuration (keyword "--uhf 2" in xtb). The scans begin from the S_0 equilibrium bond distances and a harmonic constraint on the adjacent bond of the other photochemical cleavage pathway is employed. E.g., for the PES scan of C_I—O (see also Fig. S3, we use C_H, C_I, and C_R to denote the oxetane carbon atoms bonded to the (formaldehyde) hydrogens, isatin, and the aromatic substituents, respectively), the C_I—C_R bond is constrained to the S_0 equilibrium bond distance and *vice versa*.

The electronic ground state and low-lying excited singlet (S_1) and triplet (T_1, T_2) states are computed using *hole-hole* Tamm-Dancoff approximated density functional theory (*hh*-TDA).[28,29] We employ the floating occupation molecular orbital (FOMO) scheme for the generation of the reference configuration as presented in Ref. [29], evenly distributing all electrons among all occupied orbitals and the LUMO.

In Figure S4, the PES scan of free **5a1** for the C_H—O and **5a2** C_H—C_R bonds are illustrated. These pose the second bond scission to arrive at the photochemical cleave products of *ent*-**5a**, i.e., **9a** and **10a** (*via* **5a1**, S4 a) or **7a** and CH_2O (*via* **5a2**, S4 b) respectively.

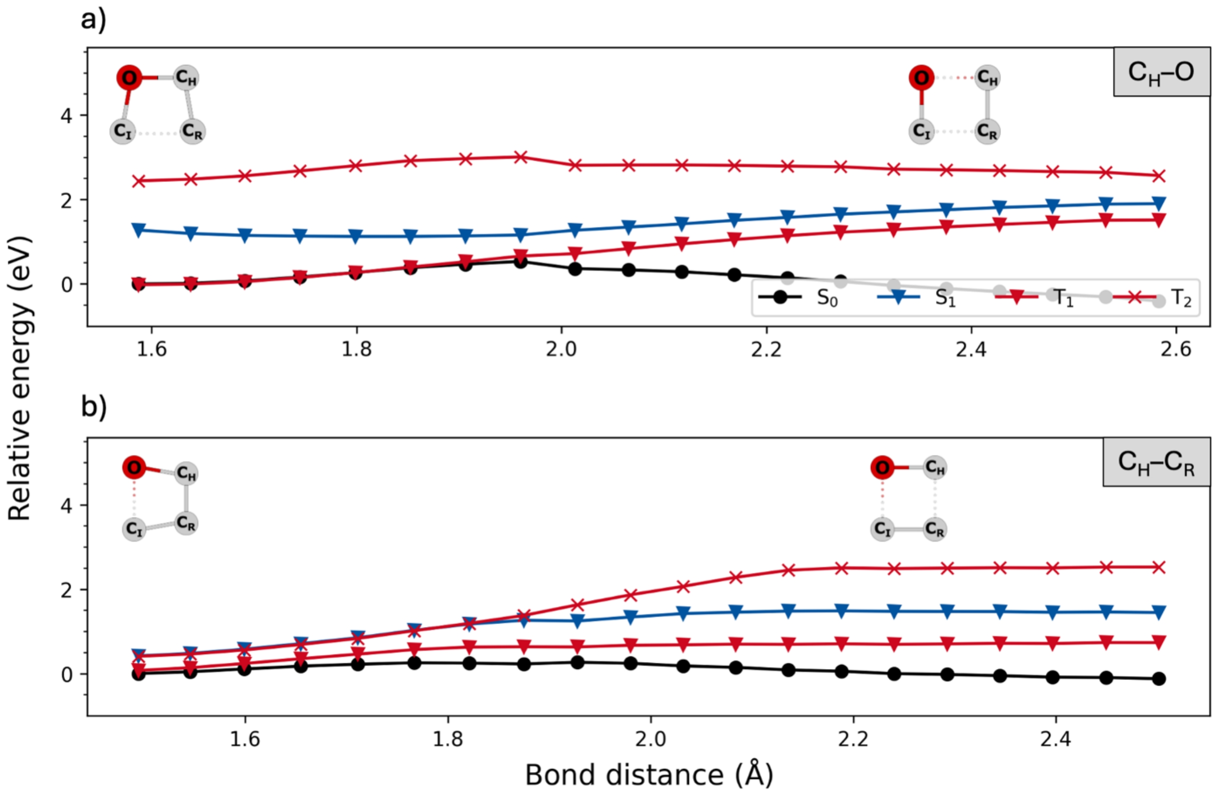

Figure S4: PES scans for the second bond cleavage, i.e., along the C_H—O (a) and C_H—C_R (b) reaction coordinate of free *ent*-**5a**. The geometries were generated using an open-shell GFN2-xTB configuration. The shown electronic energies refer to the FOMO-*hh*-PBEh-3c level of theory. Pictograms illustrate the connectivity in the oxetane ring at different reaction coordinate distances.

The S_0 and T_1 are degenerate in energy along C_H—O up to a bond distance of 1.90 Å. At larger distances, the states become energetically separated again, which marks the point where the photochemical cleavage products are formed on the ground state.

To further understand the small barrier to the first bond cleavage in *ent*-**5a**, we conducted the same PES scan (see Figure 6 in the main article) with the full noncovalent complex geometry *ent*-**5a**·**6** (Figure S5). This allows us to evaluate whether the PESs along this reaction coordinate is similar in free *ent*-**5a** and in presence of **6**.

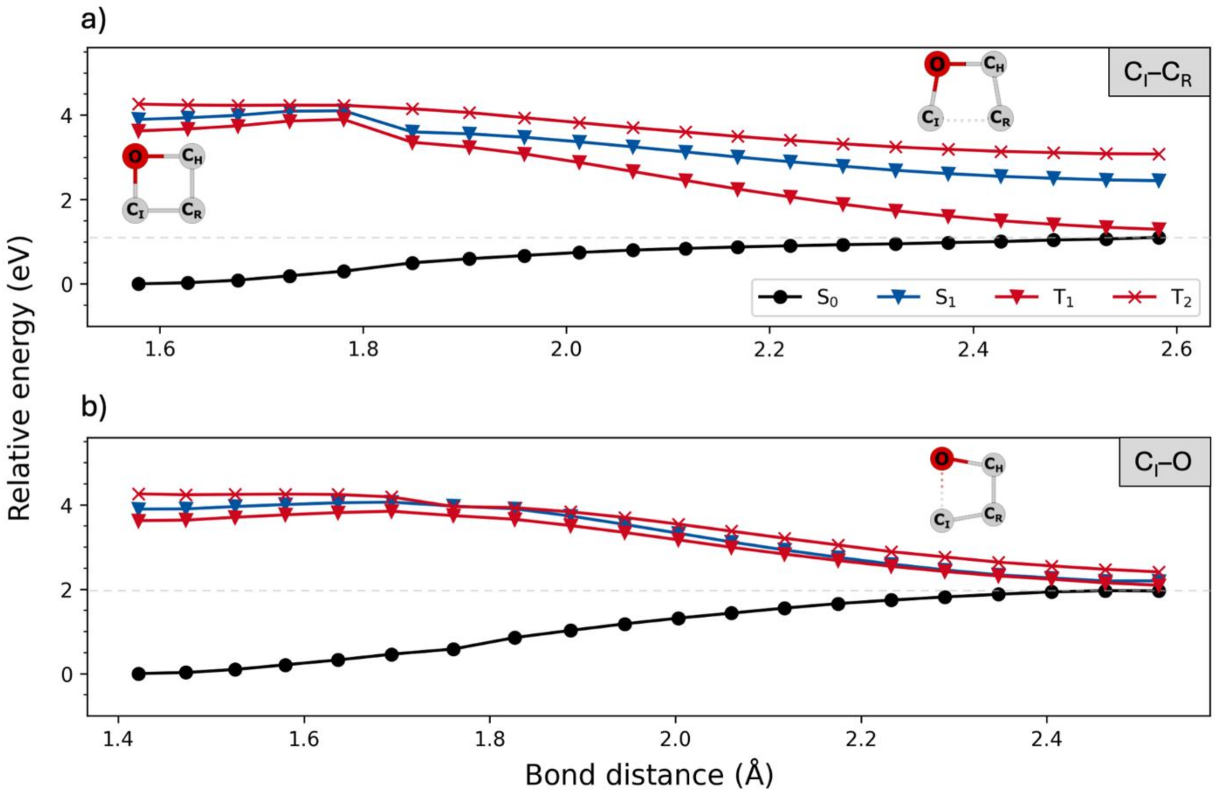

Figure S5: PES scan along the C_I—C_R (top) and C_I—O (bottom) reaction coordinate of *ent*-**5a·6**. The geometries were generated using an open-shell GFN2-xTB configuration. The shown electronic energies refer to the FOMO-*hh*-PBEh-3c level of theory. Pictograms illustrate the connectivity in the oxetane ring at different reaction coordinate distances. For the C_I—O scan, an additional constraint between the thioxanthone sheet and the oxygen atom of the oxetane ring was applied. A dashed line is drawn on the S_0 energy level at 2.55 Å to ease comparison between a) and b).

As can be seen, the PES scans inside the complex and inside the free oxetane are comparable. Similar to what was observed for free *ent*-**5a**, the cleave path along C_I—O features a small energetic barrier to arrive at **5a1·6**. A "kink" in the potential curves of all states is to be noted at around 1.65 Å. This may be a result of the additional constraint applied for the calculation but does not affect the qualitative description of a near barrierless ring opening along this path. One can clearly identify the S_1, T_1 as well as the T_2 states to be almost degenerate along the potential curve. The T_1 is almost degenerate with the S_0 at about 2.50 Å. For the ring opening along C_I—C_R, we find a slight increase in energy up to a distance of $d(\mathrm{C_I} - \mathrm{C_R})$ = 1.70 Å, which is different from the situation found for *ent*-**5a**. For the latter (Fig. 6, main article), the T_1 drops in energy at 1.65 Å and becomes degenerate with the S_0 at 2.60 Å. This observation is in line with the observation of

decomposition along both pathways for *ent*-**5a·6** depending if the sensitized or direct excitation situation is considered.

Photoexcitation and Energy Transfer

In this section, we outline the photophysics that precede the photochemical decomposition of *ent*-**5a**. For this purpose, we computed vertical excitation energies (VEEs) using DFT in conjunction with multireference configuration interaction (MRCI) method — DFT/MRCI.[30,31] Throughout, the R2018 Hamiltonian[32] is employed. A configuration selection threshold of 1.0 E_h and the standard parametrization of the Hamiltonian (abbreviated as “standard settings” in the following) are used. The anchor configuration is computed with Turbomole (version 7.6)[33,34] at the BHLYP[35]/def2-SV(P)[3] level of theory. We use the command line tool cefine[36] to generate all required input files. The resolution of the identity for Coulomb integrals (RI-J and RI-C) is employed[24,37] with corresponding auxiliary basis sets.[26] The initial configuration interaction (CI) reference space is generated from a (4,4) active space restricted to single and double excitations. MOs with energies ϵ outside the interval $-3.0\ E_h < \epsilon < 2.0\ E_h$ of the BHLYP/def2-SV(P) anchor configuration are kept frozen for the MRCI. Figure S6 contains the three (four) lowest singlet (triplet) VEEs computed for the *ent*-**5a**, **6** and the corresponding noncovalent complexes.

Compared to the experimental irradiation wavelength of λ = 398 nm (3.12 eV), direct excitation of **5a** seems unlikely. As such, we compute VEEs for **6** and the full complexes **5a·6** and *ent*-**5a·6** (Fig. S6)

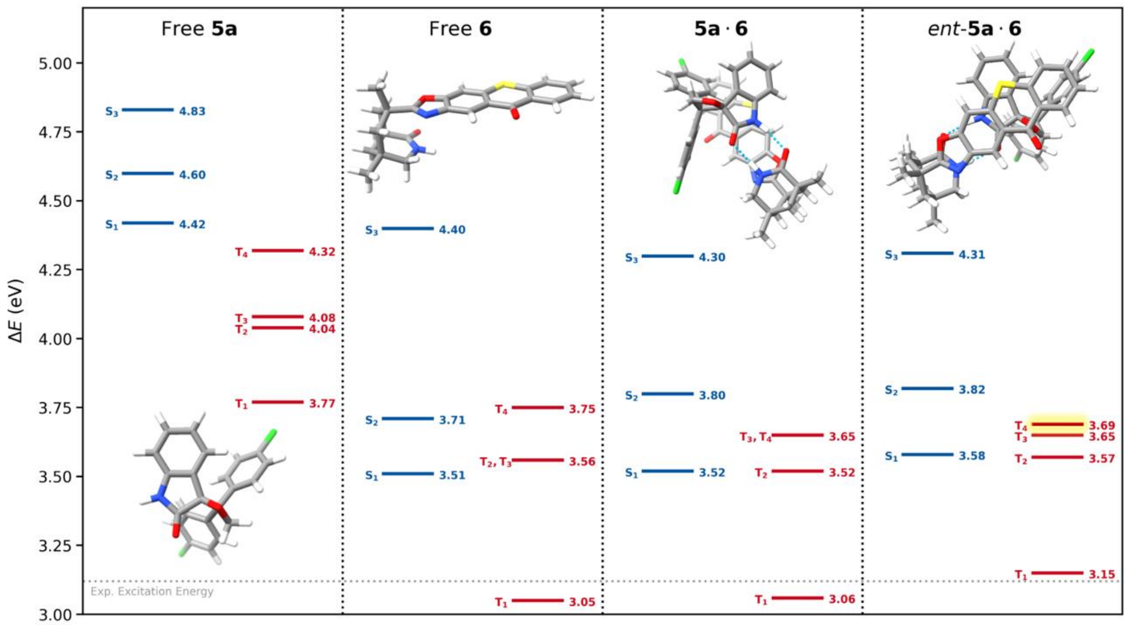

Figure S6: Vertical excitation energies of **5a** (first column from left), **6** (second column), complex **5a·6** (third column) and *ent*-**5a·6** (fourth column) computed with DFT/MRCI (R2018, standard settings) using a BHLYP/def2-SV(P) reference configuration. The experimental excitation energy of 398 nm (3.12 eV) is drawn for comparison. The oxetane-localized triplet state of *ent*-**5a·6** is highlighted in yellow (see also Fig. S7 below for the corresponding MOs).

Direct excitation of *ent*-**5a** at λ = 366 nm (3.39 eV) leads to a photochemical conversion of the oxetane. Comparing to the computed VEEs of *ent*-**5a**, we seem to overestimate its S_1 energy which amounts to 4.42 eV at the chosen theory level.

The energy of the lowest excited singlet state of *ent*-**5a** (4.42 eV) is considerably higher in energy than that of **6** (3.51 eV). Compared to the experimental irradiation wavelength (λ_{exp} = 398 nm, 3.12 eV), likely only the S_1 state of the catalyst is accessible at the chosen reaction conditions. For the photochemical reaction to occur, either electron or energy transfer would be required to stimulate cycloreversion in *ent*-**5a**. Given that the low-lying states inside the complex can already be identified in the free species (see MOs in Figure S7), we consider the energy transfer to be the more likely option compared to an electron transfer.

Nonetheless, we find the quality of the VEEs determined using DFT/MRCI to be satisfactorily considering that no excited state solvation effects, nor any vibrational effects of the systems in solution were included in our computational protocol.

For isolated **6**, we determined the lower-lying singlet state energys to be 3.51 eV (S_1) and 3.71 eV (S_2) respectively. Again, taking into consideration the experimental irradiation wavelength of 398 nm (3.12 eV), we still overestimate the relative energy of the singlet states that could be populated upon irradiation with light.

The MOs that are associated with the largest contribution to the excited triplet states are depicted in Figure S7. Based on these MOs, we can clarify that the T_4 in *ent*-**5a·6** is localized on the oxetane. T_1 and T_2 correspond to $\pi\pi^*$ and T_3 to $n\pi^*$ excitations of **6**, correspondingly. The idea that intersystem crossing (ISC) from the populated singlet state(s) to the energetically proximate triplet states is supported from the fact that a small energetic gap is present based on the VEEs. It is possible that ISC leads to a triplet state higher than T_1 and that the T_1 is reached *via* rapid internal conversion (IC).

The cycloreversion of *ent*-**5a** should be driven from a substrate-localized state, which can be reached through a Dexter-type energy transfer from **6** to *ent*-**5a** (see below). This energy transfer process seems to be the main channel for the photochemical conversion to proceed, which is why we aimed on modelling this step of the photochemical mechanism in more detail.

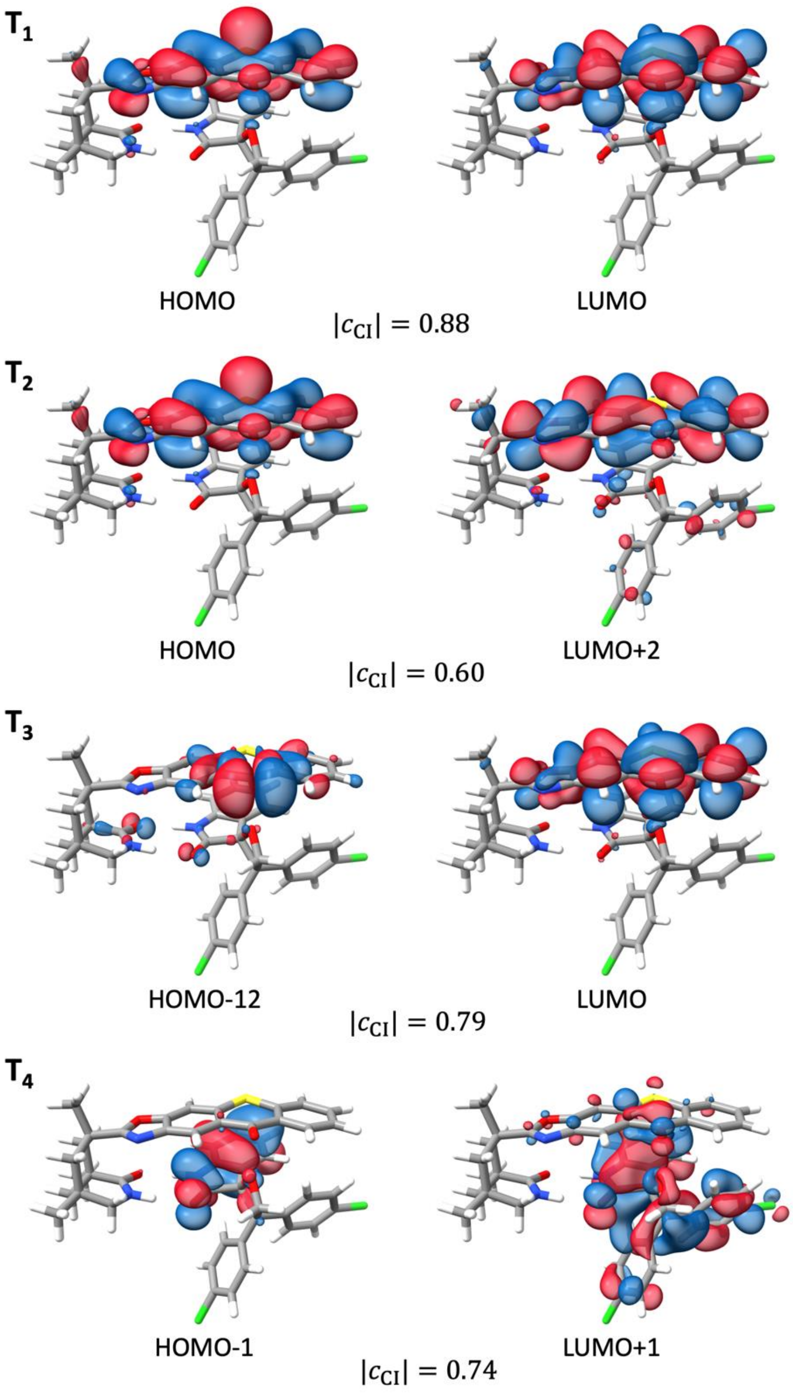

Figure S7: BHLYP/def2-SV(P) molecular orbitals (MOs) that correspond to the leading configurations of the T_1—T_4 triplet states in *ent*-**5a**·**6** as identified with DFT/MRCI(R2018) using standard settings. The absolute value of the expansion coefficients is given, and the MOs are labeled relative to the highest occupied (HOMO) and lowest unoccupied molecular orbital (LUMO). The contour value to plot the isosurfaces was set to ± 0.02 consistently.

To improve the estimate for the vertical excitation energy to the thioxanthone-localized S_1 and the corresponding minimum of *ent*-**5a·6**, we performed state-specific calculations using ΔSCF. Available with ORCA (version 6.0.0), the maximum overlap method (MOM)[38] procedure was used to compute the S_1 at the PBEh-3c level of theory. An open-shell determinant where an electron is promoted from the HOMO to the LUMO was used, and the MO coefficients from an unrestricted T_1 computation served as guess for the ΔSCF calculation. The choice of this configuration is based on the dominant configuration identified from our DFT/MRCI calculation (see also Fig. S7). For the SCF, adjusted convergence settings were used.

In the same way, we were able to optimize to the S_1 minimum geometry. The corresponding electronic energies are provided in Table S4 below.

Table S4: Electronic energies of the S_1 at the Franck-Condon (FC) point and the minimum (Min) geometry for *ent*-**5a·6** computed using MOM-based ΔSCF with PBEh-3c. The adiabatic S_0 (RKS-PBEh-3c), T_1 energies (UKS-PBEh-3c), and relative electronic energy ΔE_{rel} are given for comparison.

State	E_{el} (E_h)	ΔE_{rel} (eV)
S_0	−3667.022601	0.00
T_1 (Min)	−3666.912543	2.99
S_1 (FC)	−3666.894612	3.48
S_1 (Min)	−3666.903914	3.23

The S_1 VEE of *ent*-**5a·6** computed using ΔSCF amounts to 3.48 eV and is lower than that determined using DFT/MRCI (3.58 eV). This is in resonance with the fact that lower experimental irradiation energies facilitate the photochemical conversion. The relaxed S_1 minimum energy amounts to 3.23 eV. Compared to the adiabatic T_1 energy (UKS-PBEh-3c) at 2.99 eV, the energy gap S_1—T_1 with 0.24 eV already seems sufficiently small for ISC but is potentially even smaller for higher-lying triplet states.

Identification of Conical Intersections and Crossing Points

To simulate the energy transfer process in the *ent*-**5a·6*** complex, points of degeneracies of different potential energy surfaces (PESs) are identified. In this work, the minimum energy conical intersections (MECIs) between the T_1 and T_2 state are computed with floating occupation molecular orbital-complete active space configuration interaction (FOMO-CASCI)[39–41] as implemented in TeraChem. Throughout, a (4,4) active space is used where four electrons are

evenly smeared out within the active four orbitals during the SCF iterations (constant smearing). Grimme's D3 dispersion correction with Becke-Johnson damping D3(BJ)[7] and a def2-SV(P)[3] are used. Analytical nonadiabatic coupling (NAC) vectors are available for this method and used in the optimization of the MECIs.[20]

For the computation of minimum energy crossing points (MECPs), i.e. crossing points of PESs of electronic states with different spin multiplicity, a development version of CREST (based on version 3.0.1) is used. This version includes the recently proposed algorithm to compute MECPs based on a bias potential that takes into account the energy of the crossing states.[42] In this routine, only nuclear gradients and energies of the involved states are required to optimize towards the MECP. In principle, this procedure allows to compute MECPs at various theory levels, since only energies and nuclear gradients of the involved states need to be provided to CREST ("generic" runmode). For the purpose of studying spin-forbidden non-radiative transitions in this work, we compute S_0/T_1 MECPs using UKS-DFT with PBEh-3c in TeraChem. This is done with the aim of achieving a consistent description of the energy profile matching theory levels of the remaining parts of the computational protocol.

Modelling of Dexter-type Energy Transfer

From the excited S_1 state of **6**, intersystem crossing (ISC) is expected to occur, leading to triplet states being populated. The Dexter transfer to the oxetane T_1 state can be modelled *via* a T_2/T_1 minimum energy conical intersection (MECI) within the complex. This is relevant to describe the photochemical conversion on the T_1 PES. The computed T_1/T_2 MECI (FOMO-CASCI(4,4)-D3(BJ)/def2-SV(P)) and the corresponding natural transition orbitals (NTOs)[43] at the MECI geometry are shown in Figure S8 for complex *ent*-**5a·6**.

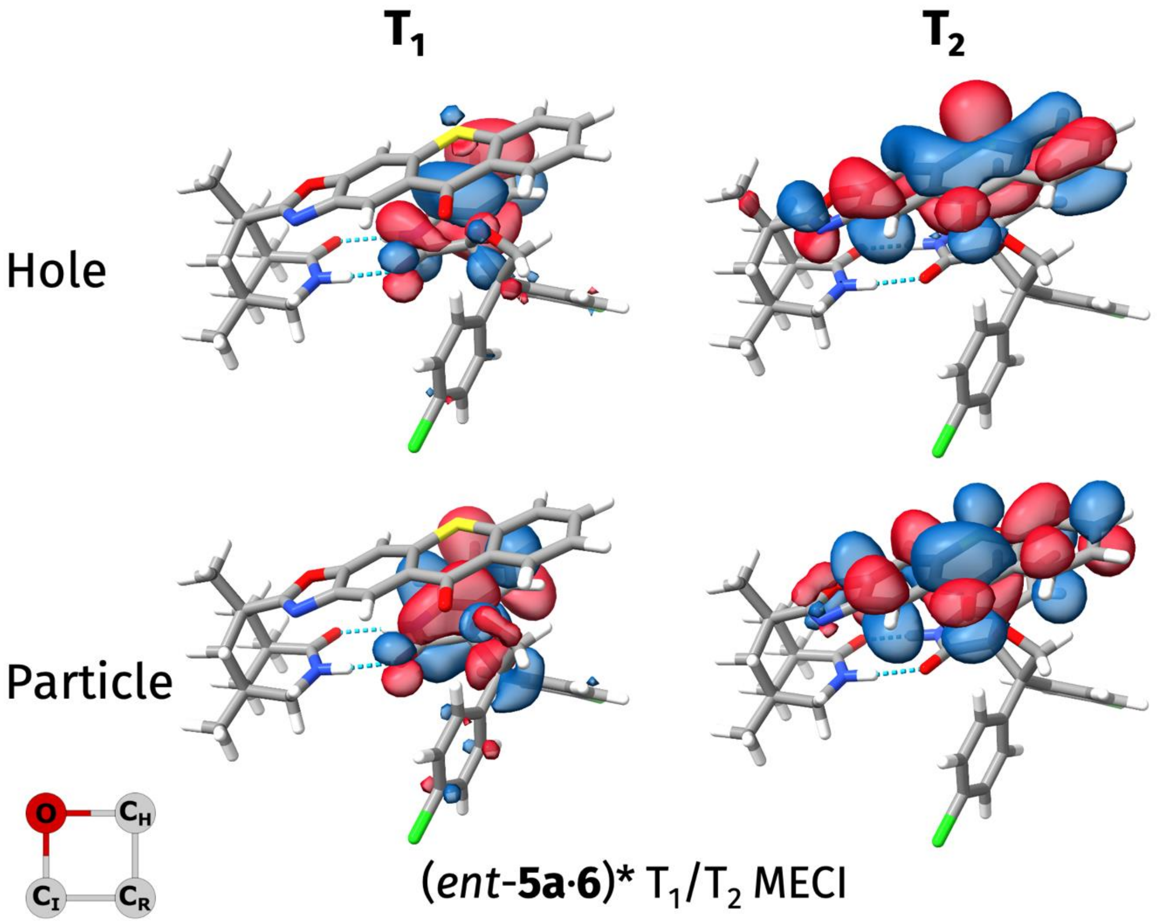

Figure S8: FOMO-CASCI(4,4)-D3(BJ)/def2-SV(P) dominant hole (top) and particle (bottom) NTOs computed at the T_1/T_2 MECI geometry of complex *ent*-**5a·6**. The cartoon of the oxetan moiety (bottom left) is given to make clear that the oxetane ring is intact at this geometry.

Clearly, the states involved in the Dexter-type transfer are states with electronic excitation localized either on **6** or on *ent*-**5a**. The dominant hole and particle NTOs of the T_1 state are localized on *ent*-**5a** and those of the T_2 state on **6** correspondingly (note that the order at the MECI is arbitrary). At the MECIs of complex **5a·6** (not shown here), similar NTOs constitute the respective states, which underlines the similarity of the MECIs for both complexes (also in relative energy, cf. Table S5 below).

The energy transfer can be resolved further looking at the NTOs of species (**5a***)·**6** and (**5a2***)·**6** (Figure S9) respectively. The asterisk indicates the excited moiety, where the NTOs are localized. We obtain these geometries optimizing the T_1 state based on a complex geometry with intermediates **5a1** or **5a2** associated to **6**.

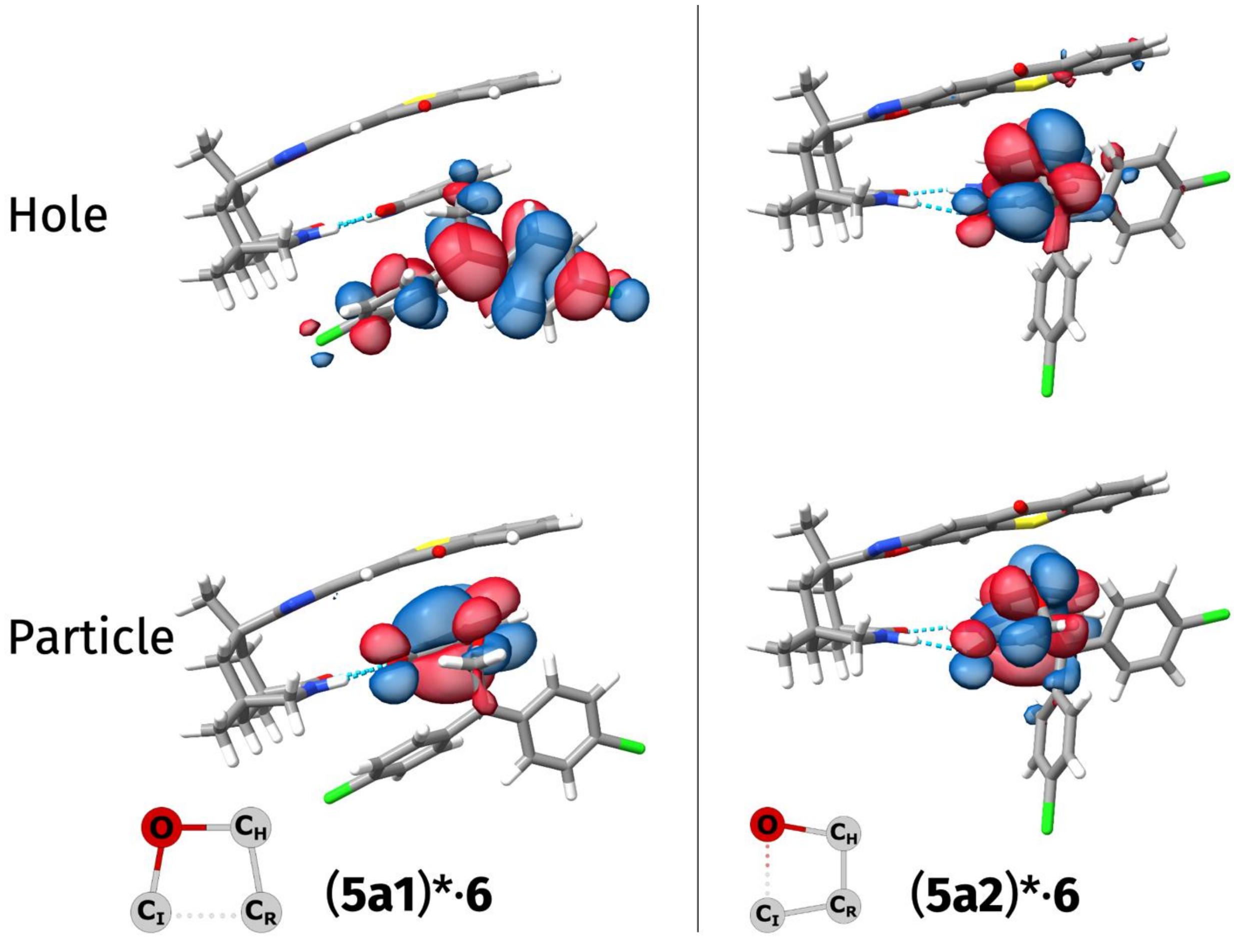

Figure S9: FOMO-CASCI(4,4)-D3(BJ)/def2-SV(P) dominant hole and particle NTOs of the T1 state for (**5a1***)·**6** (left) and (**5a2***)·**6** (right) after Dexter energy transfer from **6** to *ent*-**5a**. The respective connectivity in the oxetane ring is illustrated schematically.

A scission of C_I—C_R (**5a1**) or C_I—O (**5a2**) allows to compute a localized T_1 state, where the NTOs are located on the substrate. We suspect that the energy transfer process *via* the T_1/T_2 MECI causes the first bond scission in the oxetane ring towards (**5a1***)·**6** or (**5a2***)·**6**.

The energy barrier to the T_1/T_2 MECI $\Delta E^{‡}$ is estimated from the electronic energy difference relative to the T_1 minimum $E_{\mathrm{Min,T1}}$ before energy transfer, that is, relative to *ent*-**5a·6***.

$$\Delta E^{‡} = \frac{E_{\mathrm{MECI,T1}} + E_{\mathrm{MECI,T2}}}{2} - E_{\mathrm{Min,T1}} \qquad \text{(Eq. 2)}$$

The arithmetic mean of the quasi-degenerate triplet states (T_1 and T_2) $E_{\mathrm{MECI},i}$ is taken. Data of the energy transfer process from the catalyst to the oxetane species leading to intermediates (**5a1***)·**6** and (**5a2***)·**6** are provided in Table S5.

Table S5: Electronic energies $E_{\mathrm{el},i}$ of the triplet states (T_1 and T_2) at the T_1 minimum and T_1/T_2 MECI of *ent*-**5a·6** computed using FOMO-CASCI(4,4)-D3(BJ)/def2-SV(P). A barrier to the MECI $\Delta E^{\ddagger}$ is computed using Eq. 2. Free energies (using ωB97X-D4/def2-QZVPP//PBEh-3c electronic energies) are given for comparison (see Eq. 1 for contributions and Tab. S1 for involved methods). ΔG_{ET} corresponds to the free energy difference of the Dexter energy transfer process involving bond scission to (**5a1***)·**6** or (**5a2***)·**6** respectively. The asterisk indicates where the triplet state is localized. Electronic energies of the energy transfer process of **5a·6** are given for comparison.

	ent-**5a·6*** T_1	(*ent*-**5a·6**)* T_1/T_2 MECI	**5a1*·6** T_1	**5a2*·6** T_1
FOMO-CASCI(4,4)-D3(BJ)/def2-SV(P)				
$E_{\mathrm{el,T1}}$ (E_h)	−3655.310288	−3655.301003	−3655.395971	−3655.362870
$E_{\mathrm{el,T2}}$ (E_h)	−3655.269228	−3655.301003	−3655.252893	−3655.211864
$\Delta E_{\mathrm{el,T1}}$ (eV)	1.11	0.00	3.86	4.08
$\Delta E^{\ddagger}$ (kJ mol^{-1})	-	+24	-	-
ωB97X-D4/def2-QZVPP//PBEh-3c				
$E_{\mathrm{el,T1}}$ (E_h)	−3675.567032	-	−3675.644799	−3675.614861
G_{T1} (E_h)	−3674.949671	-	−3675.023204	−3674.999359
ΔG_{ET} (kJ mol^{-1})	-	-	−193	−130

	**5a·6*** T_1	(**5a·6**)* T_1/T_2 MECI	**5a1*·6** T_1	**5a2*·6** T_1
FOMO-CASCI(4,4)-D3(BJ)/def2-SV(P)				
$E_{\mathrm{el,T1}}$ (E_h)	−3655.307853	−3655.299825	-	-
$E_{\mathrm{el,T2}}$ (E_h)	−3655.273103	−3655.299808	-	-
$\Delta E_{\mathrm{el,T1}}$ (eV)	0.94	0.00	-	-
$\Delta E^{\ddagger}$ (kJ mol^{-1})		+21	-	-

Intermediates (**5a1***)·**6** and (**5a2***)·**6** are more stable than the corresponding structure with closed oxetane ring. This aligns with the idea that the cycloreversion is thermodynamically feasible. Since both intermediates are lower in energy, competing decomposition towards **9a** and **10a** on the one hand, and **7a** and CH_2O on the other is expected. (**5a1***)·**6** is the thermodynamically favored product of the first bond scission. As alluded to above, the energetic barrier to the T_1/T_2 MECI in

5a·6 is similar with $\Delta E^{\ddagger} = +21$ kJ mol^{-1} compared to *ent*-**5a·6** (+24 kJ mol^{-1}) at the chosen theory level. These findings suggest that not the excited state energy barrier but rather the distinct binding affinity to the photosensitizer and competitive inhibition of the catalyst primarily defines the observed favoured reactivity of *ent*-**5a** *via* the triplet PES.

Singlet Energy Transfer and Relaxation to the Ground State

Analogous to the T_1/T_2 MECI, a S_1/S_2 MECI was computed using FOMO-CASCI(4,4)-D3(BJ)/def2-SV(P). The electronic energies and corresponding NTOs of the involved states are shown in Table S6 and Figure S10 below.

Table S6: Electronic energies of the singlet states (S_0—S_2) at the S_1 minimum and S_0/S_1, S_1/S_2 MECIs of *ent*-**5a·6**. The S_1/S_2 MECI computed using FOMO-CASCI(4,4)-D3(BJ)/def2-SV(P) and shown in the upper part. The S_0/S_1 MECI data is shown in the bottom part and corresponds to the FOMO-*hh*-PBEh-3c level of theory. A barrier to the respective MECI is computed using Eq. 2. The asterisk indicates where the singlet state is localized.

	ent-**5a·6*** S_1	(*ent*-**5a·6**)* S_1/S_2 MECI
FOMO-CASCI(4,4)-D3(BJ)/def2-SV(P)		
$E_{\mathrm{el,S1}}$ (E_h)	−3655.265885	−3655.241121
$E_{\mathrm{el,S2}}$ (E_h)	−3655.185227	−3655.241121
$\Delta E_{\mathrm{el,S1}}$ (eV)	2.18	0.00
$\Delta E^{\ddagger}$ (kJ mol^{-1})	-	+65
	(**5a2***)·**6** S_1	(**5a2·6**)* S_0/S_1 MECI
FOMO-*hh*-PBEh-3c		
$E_{\mathrm{el,S0}}$ (E_h)	−3667.09365084	−3667.0928421919
$E_{\mathrm{el,S1}}$ (E_h)	−3667.09272289	−3667.0928404281
$\Delta E_{\mathrm{el,S1}}$ (eV)	0.03	0.00
$\Delta E^{\ddagger}$ (kJ mol^{-1})	-	+2

A possible singlet pathway with energy transfer *via* S_1/S_2 MECI was modelled for *ent*-**5a·6**. This was done to evaluate whether the photo-induced decomposition may proceed in an analogous fashion to the triplet pathway. Similar to the T_1/T_2 MECI (Fig. S8), the dominant hole and particle NTOs at the S_1/S_2 MECI are localized states (see. Fig. S10 below).

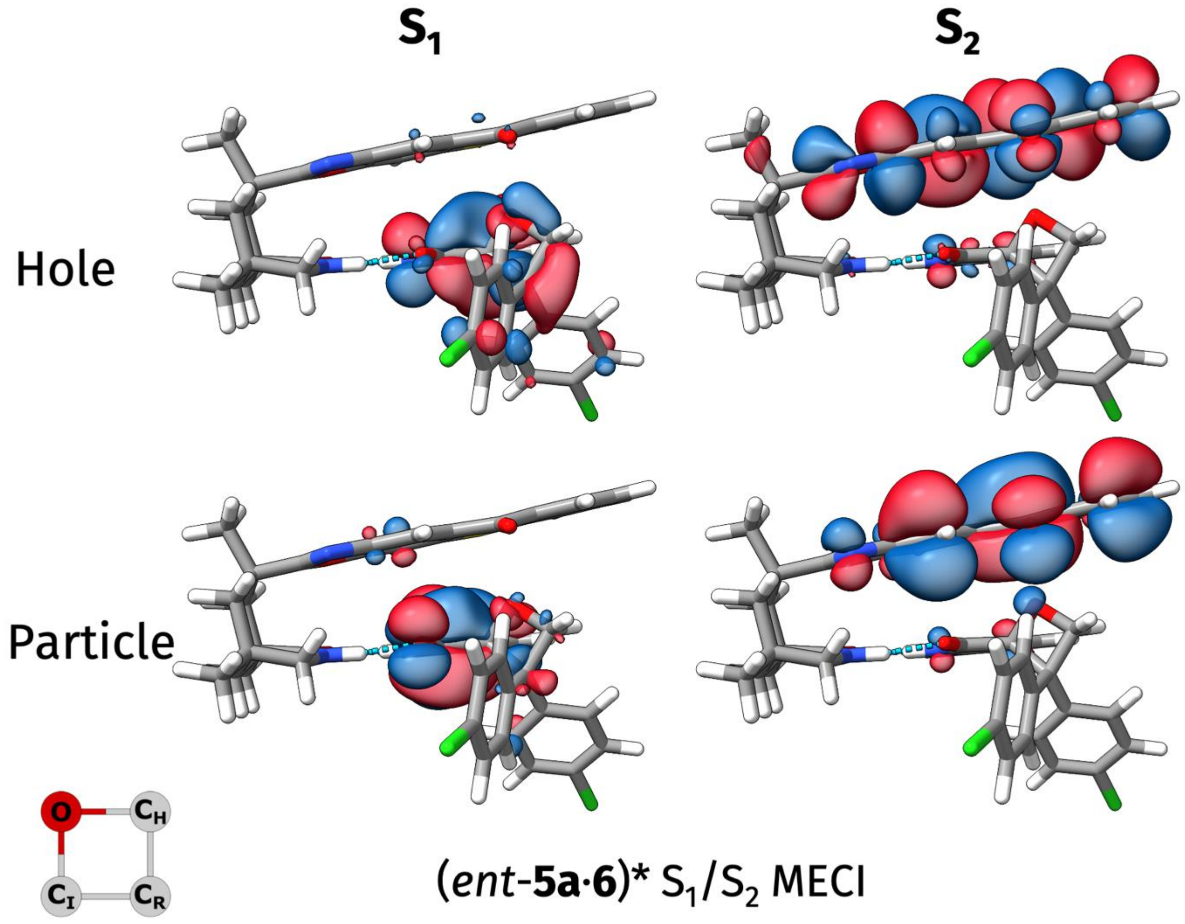

Figure S10: FOMO-CASCI(4,4)-D3(BJ)/def2-SV(P) dominant hole (top) and particle (bottom) NTOs computed at the S_1/S_2 MECI geometry of complex *ent*-**5a·6**. The cartoon of the oxetan moiety (bottom left) is given to make clear that the oxetane ring is intact at this geometry.

However, the computed barrier to the S_1/S_2 MECI is considerably higher in energy with $\Delta E^{\ddagger} = +65\ \mathrm{kJ\ mol^{-1}}$ compared to $\Delta E^{\ddagger} = +24\ \mathrm{kJ\ mol^{-1}}$ *via* T_1/T_2. It is thus unlikely that this barrier is passed in the lifetime of a singlet excited state, which is why we argue that singlet energy transfer is unfeasible.

After C_I—O bond scission, the decomposition to **7a** and CH_2O can, in principle, proceed without a significant energetic barrier (see Tab. S6). We modelled these steps using broken-symmetry

(BS-)DFT to derive Gibbs free energies and, again, adding electronic energy barriers (*hh*-PBEh-3c) on top.

From (**5a2***)·**6**, the electronic ground state can be reached *via* a S_0/S_1 MECI (see Tab. S6) that shows a negligible barrier of only +2 kJ mol^{-1}. Consequently, dissociation of CH_2O is feasible after the initial C_I—O bond scission in the oxetane ring occurred, which is in line with the direct irradiation experiments. From the given energy difference between the S_0 and S_1 states at the S_1 minimum, $\Delta E_{\mathrm{el,S1}}$, one recognizes the states' near-degeneracy that was also identified from the PES scans in Figure 5.

Last, we turn to the relaxation channel to the ground state from the T_1 minima of (**5a1***)·**6** and (**5a2***)·**6** respectively. As stated above, the derivative coupling vector-free MECP routine in the CREST program was used for this purpose. The S_0 and T_1 PESs were described at the UKS-PBEh-3c level of theory, such that an electronic energy barrier with respect to the T_1 minima can be computed. As was done for MECIs, the electronic energy barrier was added on top of the free energies (denoted with † in Figure 7 in the main article). The corresponding data is summarized in Table S7.

Table S7: Electronic energies of the S_0 and T_1 states computed at the UKS-PBEh-3c level of theory for the T_1 minima and S_0/T_1 MECPs of 5a1*·6 and 5a2*·6. The electronic energy barrier to the MECP $\boldsymbol{\Delta E_{\mathrm{MECP}}}$ has been computed in analogy to Eq. 2, averaging over the involved degenerate states at the MECP and relative to the T_1 minimum.

	(**5a1***)·**6** T_1	(**5a1***)·**6** S_0/T_1 MECP	(**5a2***)·**6** T_1	(**5a2***)·**6** S_0/T_1 MECP
$E_{\mathrm{el,S0}}$ (E_h)	-	−3666.987190	-	−3666.955618
$E_{\mathrm{el,T1}}$ (E_h)	−3666.991401	−3666.987257	−3666.957411	−3666.955316
$\Delta E_{\mathrm{S0/T1}}$ (eV)	-	0.00	-	0.01
$\Delta E^{\ddagger}$ (kJ mol^{-1})	+11	-	+5	-

The computed barriers to the S_0/T_1 MECPs reflect that, after the first bond scission to (**5a1***)·**6** (C_I—C_R) or (**5a2***)·**6** (C_I—O), the photochemical decomposition products can form passing negligibly small barriers. For (**5a1***)·**6**, the electronic energy barrier to the S_0/T_1 MECP amounts to +11 kJ mol^{-1}, while it is smaller (+5 kJ mol^{-1}) for (**5a2***)·**6**.

Gibbs Free Energy Contributions

Table S8: Free energy contributions for the species investigated in this study, divided into isolated (monomeric) and associated (dimeric) species. The values refer to the theory level outlined in Table S1. [a]: Values have been computed using ΔSCF with PBEh-3c instead. BS: Broken symmetry.

Species	State	E_{el} (E_h)	G_{TRV} (E_h)	δG_{solv} (E_h)	G (E_h)
Monomeric Species					
(*ent*-)**5a**	S_0	−1973.926191	0.271575	−0.031745	−1973.686361
**6**	S_0	−1701.718516	0.398044	−0.040307	−1701.360779
**7a**	S_0	−1859.328021	0.239328	−0.030026	−1859.118719
CH_2O	S_0	−114.585470	0.008935	−0.000392	−114.576927
**9a**	S_0	−1460.449159	0.159378	−0.017816	−1460.307597
**10a**	S_0	−513.466218	0.087512	−0.015664	−513.394371
Dimeric Species					
**5a**·**5a**	S_0	−3947.884501	0.565754	−0.061451	−3947.380198
**5a**·*ent*-**5a**	S_0	−3947.885237	0.565122	−0.061702	−3947.381816
**5a**·**6**	S_0	−3675.679452	0.692508	−0.068680	−3675.055624
ent-**5a**·**6**	S_0	−3675.687306	0.692493	−0.067907	−3675.062719
	S_1[a]	−3666.903914	-	-	-
	T_1	−3675.567032	0.687465	−0.070104	−3674.949671
**7a**·**5a**	S_0	−3833.287692	0.533302	−0.060165	−3832.814555
CH_2O·**5a**	S_0	−2088.525803	0.295627	−0.035810	−2088.265986
**9a**·**5a**	S_0	−3434.395316	0.451853	−0.050580	−3433.994043
**10a**·**5a**	S_0	−2487.423373	0.379756	−0.046056	−2487.089673
**6**·**6**	S_0	−3403.471495	0.818362	−0.076142	−3402.729276
**7a**·**6**	S_0	−3561.088777	0.660285	−0.066133	−3560.494626
CH_2O·**6**	S_0	−1816.320349	0.423399	−0.042305	−1815.939255
**9a**·**6**	S_0	−3162.194814	0.578633	−0.054632	−3161.670813
**10a**·**6**	S_0	−2215.223626	0.506718	−0.051407	−2214.768316
**7a**·**7a**	S_0	−3718.685797	0.500233	−0.057670	−3718.243234
CH_2O·CH_2O	S_0	−229.174843	0.026996	−0.006896	−229.154743
**9a**·**9a**	S_0	−2920.915347	0.338499	−0.037707	−2920.614555
**10a**·**10a**	S_0	−1026.959118	0.193397	−0.031483	−1026.797205
**5a1**·**6**	T_1	−3675.644799	0.688844	−0.067249	−3675.023204
**5a2**·**6**	S_0 (BS)	−3675.613944	0.687071	−0.071180	−3674.998052
	T_1	−3675.614861	0.687067	−0.071565	−3674.999359

References for this section

(1) Bannwarth, C.; Ehlert, S.; Grimme, S. GFN2-xTB—An Accurate and Broadly Parametrized Self-Consistent Tight-Binding Quantum Chemical Method with Multipole Electrostatics and Density-Dependent Dispersion Contributions. *J. Chem. Theory Comput.* **2019**, *15* (3), 1652–1671.

(2) Grimme, S.; Brandenburg, J. G.; Bannwarth, C.; Hansen, A. Consistent Structures and Interactions by Density Functional Theory with Small Atomic Orbital Basis Sets. *J. Chem. Phys.* **2015**, *143*, 054107.

(3) Weigend, F.; Ahlrichs, R. Balanced Basis Sets of Split Valence, Triple Zeta Valence and Quadruple Zeta Valence Quality for H to Rn: Design and Assessment of Accuracy. *Phys. Chem. Chem. Phys.* **2005**, *7*, 3297–3305.

(4) Perdew, J. P.; Burke, K.; Ernzerhof, M. Generalized Gradient Approximation Made Simple. *Phys. Rev. Lett.* **1996**, *77*, 3865–3868.

(5) Kruse, H.; Grimme, S. A Geometrical Correction for the Inter- and Intra-Molecular Basis Set Superposition Error in Hartree-Fock and Density Functional Theory Calculations for Large Systems. *J. Chem. Phys.* **2012**, *136* (15), 154101.

(6) Grimme, S.; Antony, J.; Ehrlich, S.; Krieg, H. A Consistent and Accurate Ab Initio Parametrization of Density Functional Dispersion Correction (DFT-D) for the 94 Elements H-Pu. *J. Chem. Phys.* **2010**, *132*, 154104.

(7) Grimme, S.; Ehrlich, S.; Goerigk, L. Effect of the Damping Function in Dispersion Corrected Density Functional Theory. *J. Comput. Chem.* **2011**, *32*, 1456–1465.

(8) Pracht, P.; Bohle, F.; Grimme, S. Automated Exploration of the Low-Energy Chemical Space with Fast Quantum Chemical Methods. *Phys. Chem. Chem. Phys.* **2020**, *22* (14), 7169–7192.

(9) Conformer-Rotamer Ensemble Sampling Tool (CREST). https://github.com/grimme-lab/crest.

(10) Semiempirical Extended Tight-Binding Program Package. https://github.com/grimme-lab/xtb.

(11) Grimme, S.; Bannwarth, C.; Shushkov, P. A Robust and Accurate Tight-Binding Quantum Chemical Method for Structures, Vibrational Frequencies, and Noncovalent Interactions of Large Molecular Systems Parametrized for All Spd-Block Elements (Z=1-86). *J. Chem. Theory Comput.* **2017**, *13*, 1989–2009.

(12) Grimme, S.; Bannwarth, C.; Caldeweyher, E.; Pisarek, J.; Hansen, A. A General Intermolecular Force Field Based on Tight-Binding Quantum Chemical Calculations. *J. Chem. Phys.* **2017**, *147*, 161708.

(13) Najibi, A.; Goerigk, L. DFT-D4 Counterparts of Leading Meta-Generalized-Gradient Approximation and Hybrid Density Functionals for Energetics and Geometries. *J. Comput. Chem.* **2020**, *41* (30), 2562–2572.

(14) Caldeweyher, E.; Ehlert, S.; Hansen, A.; Neugebauer, H.; Spicher, S.; Bannwarth, C.; Grimme, S. A Generally Applicable Atomic-Charge Dependent London Dispersion Correction. *J. Chem. Phys.* **2019**, *150* (15), 154122.

(15) Grimme, S. Supramolecular Binding Thermodynamics by Dispersion Corrected Density Functional Theory. *Chem. Eur. J.* **2012**, *18* (32), 9955–9964.

(16) Spicher, S.; Grimme, S. Efficient Computation of Free Energy Contributions for Association Reactions of Large Molecules. *J. Phys. Chem. Lett.* **2020**, *11* (16), 6606–6611.

(17) Ehlert, S.; Stahn, M.; Spicher, S.; Grimme, S. Robust and Efficient Implicit Solvation Model for Fast Semiempirical Methods. *J. Chem. Theory Comput.* **2021**, *17* (7), 4250–4261.

(18) Bannwarth, C.; Caldeweyher, E.; Ehlert, S.; Hansen, A.; Pracht, P.; Seibert, J.; Spicher, S.; Grimme, S. Extended Tight-Binding Quantum Chemistry Methods. *WIREs Comput. Mol. Sci.* **2021**, *11* (2), e1493.

(19) Neese, F. The ORCA Program System. *WIREs Comput. Mol. Sci.* **2012**, *2*, 73–78.

(20) Seritan, S.; Bannwarth, C.; Fales, B. S.; Hohenstein, E. G.; Isborn, C. M.; Kokkila-Schumacher, S. I. L.; Li, X.; Liu, F.; Luehr, N.; Snyder Jr., J. W.; Song, C.; Titov, A. V.; Ufimtsev, I. S.; Wang, L.-P.; Martínez, T. J. TeraChem: A Graphical Processing Unit-Accelerated Electronic Structure Package for Large-Scale Ab Initio Molecular Dynamics. *WIREs Comput. Mol. Sci.* **2021**, *11* (2), e1494.

(21) Seritan, S.; Bannwarth, C.; Fales, B. S.; Hohenstein, E. G.; Kokkila-Schumacher, S. I. L.; Luehr, N.; Snyder, J. W.; Song, C.; Titov, A. V.; Ufimtsev, I. S.; Martínez, T. J. TeraChem: Accelerating Electronic Structure and Ab Initio Molecular Dynamics with Graphical Processing Units. *J. Chem. Phys.* **2020**, *152* (22), 224110.

(22) Steinbach, P.; Bannwarth, C. Combining Low-Cost Electronic Structure Theory and Low-Cost Parallel Computing Architecture. *Phys. Chem. Chem. Phys.* **2024**, *26* (23), 16567–16578.

(23) Luehr, N.; Ufimtsev, I. S.; Martinez, T. J. Dynamic Precision for Electron Repulsion Integral Evaluation on Graphical Processing Units (Gpus). *J. Chem. Theory Comput.* **2011**, *7*, 949–954.

(24) Vahtras, O.; Almlöf, J.; Feyereisen, M. W. Integral Approximations for LCAO-SCF Calculations. *Chem. Phys. Lett.* **1993**, *213* (5), 514–518.

(25) Neese, F. An Improvement of the Resolution of the Identity Approximation for the Formation of the Coulomb Matrix. *J. Comput. Chem.* **2003**, *24* (14), 1740–1747.

(26) Weigend, F. Accurate Coulomb-Fitting Basis Sets for H to Rn. *Phys. Chem. Chem. Phys.* **2006**, *8*, 1057–1065.

(27) Neese, F.; Wennmohs, F.; Hansen, A.; Becker, U. Efficient, Approximate and Parallel Hartree–Fock and Hybrid DFT Calculations. A 'Chain-of-Spheres' Algorithm for the Hartree–Fock Exchange. *Chem. Phys.* **2009**, *356* (1), 98–109.

(28) Bannwarth, C.; Yu, J. K.; Hohenstein, E. G.; Martínez, T. J. Hole–Hole Tamm–Dancoff-Approximated Density Functional Theory: A Highly Efficient Electronic Structure Method Incorporating Dynamic and Static Correlation. *J. Chem. Phys.* **2020**, *153* (2), 024110.

(29) Yu, J. K.; Bannwarth, C.; Hohenstein, E. G.; Martínez, T. J. Ab Initio Nonadiabatic Molecular Dynamics with Hole–Hole Tamm–Dancoff Approximated Density Functional Theory. *J. Chem. Theory Comput.* **2020**, *16* (9), 5499–5511.

(30) Grimme, S.; Waletzke, M. A Combination of Kohn–Sham Density Functional Theory and Multi-Reference Configuration Interaction Methods. *J. Chem. Phys.* **1999**, *111* (13), 5645–5655.

(31) Marian, C. M.; Heil, A.; Kleinschmidt, M. The DFT/MRCI Method. *WIREs Comput. Mol. Sci.* **2019**, *9* (2), e1394.

(32) Heil, A.; Kleinschmidt, M.; Marian, C. M. On the Performance of DFT/MRCI Hamiltonians for Electronic Excitations in Transition Metal Complexes: The Role of the Damping Function. *J. Chem. Phys.* **2018**, *149* (16), 164106.

(33) TURBOMOLE V7.6 2020, a development of University of Karlsruhe and Forschungszentrum Karlsruhe GmbH, 1989-2007, TURBOMOLE GmbH, since 2007; available from http://www.turbomole.com.

(34) Furche, F.; Ahlrichs, R.; Hättig, C.; Klopper, W.; Sierka, M.; Weigend, F. Turbomole. *WIREs Comput. Mol. Sci.* **2014**, *4*, 91–100.

(35) Becke, A. D. A New Mixing of Hartree–Fock and Local Density-Functional Theories. *J. Chem. Phys.* **1993**, *98*, 1372–1377.

(36) commandline define. https://github.com/grimme-lab/cefine.

(37) Von Arnim, M.; Ahlrichs, R. Performance of Parallel TURBOMOLE for Density Functional Calculations. *J. Comput. Chem.* **1998**, *19* (15), 1746–1757.

(38) Gilbert, A. T. B.; Besley, N. A.; Gill, P. M. W. Self-Consistent Field Calculations of Excited States Using the Maximum Overlap Method (MOM). *J. Phys. Chem. A* **2008**, *112* (50), 13164–13171.

(39) Slavíček, P.; Martínez, T. J. Ab Initio Floating Occupation Molecular Orbital-Complete Active Space Configuration Interaction: An Efficient Approximation to CASSCF. *J. Chem. Phys.* **2010**, *132* (23), 234102.

(40) Hollas, D.; Šištík, L.; Hohenstein, E. G.; Martínez, T. J.; Slavíček, P. Nonadiabatic Ab Initio Molecular Dynamics with the Floating Occupation Molecular Orbital-Complete Active Space Configuration Interaction Method. *J. Chem. Theory Comput.* **2018**, *14* (1), 339–350.

(41) Hohenstein, E. G.; Bouduban, M. E. F.; Song, C.; Luehr, N.; Ufimtsev, I. S.; Martínez, T. J. Analytic First Derivatives of Floating Occupation Molecular Orbital-Complete Active Space Configuration Interaction on Graphical Processing Units. *J. Chem. Phys.* **2015**, *143* (1), 014111.

(42) Pracht, P.; Bannwarth, C. Finding Excited-State Minimum Energy Crossing Points on a Budget: Non-Self-Consistent Tight-Binding Methods. *J. Phys. Chem. Lett.* **2023**, *14* (19), 4440–4448.

(43) Martin, R. L. Natural Transition Orbitals. *J. Chem. Phys.* **2003**, *118* (11), 4775–4777.

5. Transient Absorption Spectroscopy

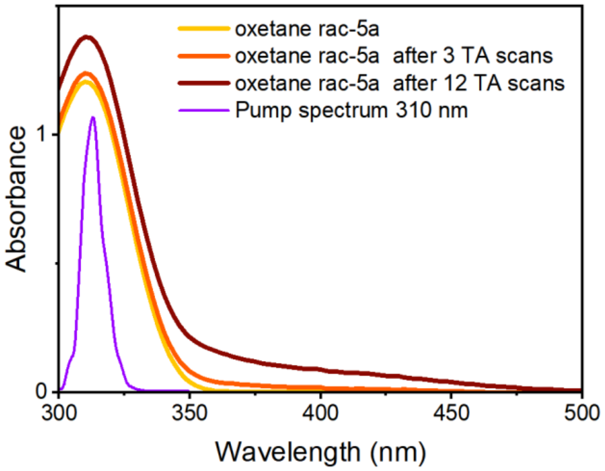

Figure S11: Absorption spectra of oxetane *rac*-**5a** after increasing number of TA scans. The pump pulse spectrum is also shown to illustrate the spectral overlap in terms of illumination conditions.

The absorption spectrum of oxetane *rac*-**5a** dissolved in chloroform was measured before and after direct excitation in the transient absorption (TA) experiment. The spectrum was examined after 3 and 12 scans, with each scan taking 4 minutes. After three scans, the absorption spectrum of rac-**5a** began to change, indicating the formation of photoproducts.

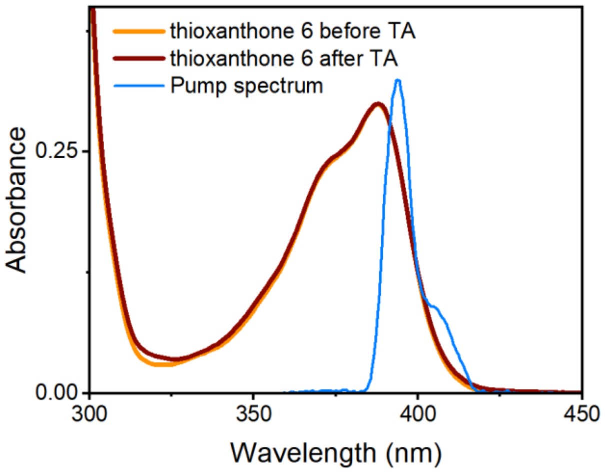

Figure S12: Absorption spectra of thioxanthone **6** before and after TA. The pump pulse spectrum is also shown to illustrate the spectral overlap in terms of illumination conditions.

The absorption spectrum of **6** dissolved in $PhCF_3$ was measured before and after the transient absorption experiment. A 1 mm flow cell was used and the optical density of **6** was 0.3. The sample was excited using 380 nm pump pulses, which target the red edge of absorption of **6**. For both TA measurements the pump energy was kept below 30 nJ. During the TA measurements, the cuvette was continuously moved perpendicularly to the laser beam path to prevent the excitation of the same pool of molecules multiple times. More details about the TA setup can be found elsewhere.[1]

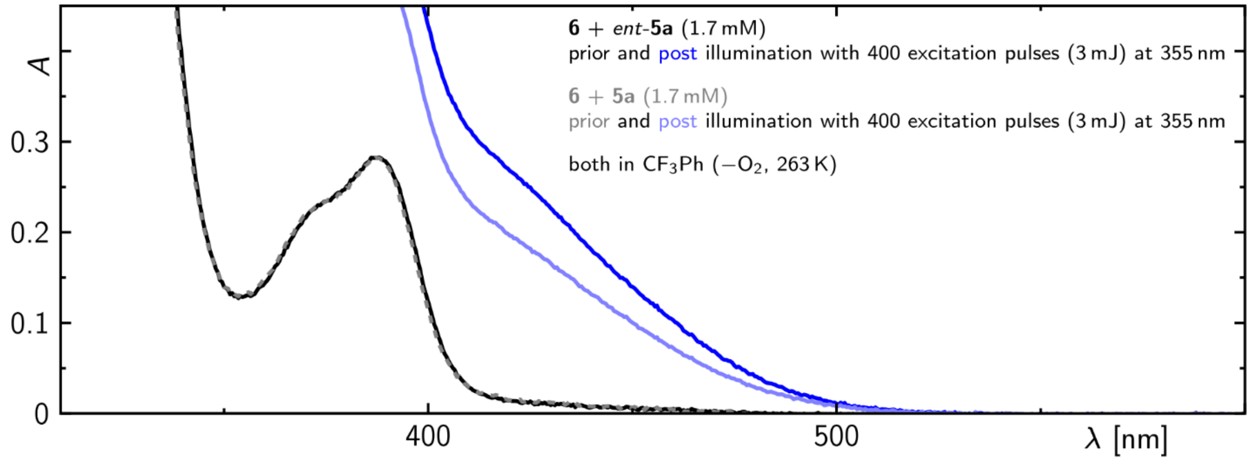

Figure S13: Absorption spectra of **6** in degassed (5 cycles of freeze-pump thaw at a pressure of $5 \cdot 10^{-5}$ mbar) $PhCF_3$ in the presence of either *ent*-**5a** or **5a** (each with c = 1.7 mM) prior and post the recording of the transient absorption on the μs time range at 263 K. This corresponds to an identical illumination of each sample with 400 laser pulses at 355 nm (3 mJ/ pulse). The sample volume of 3 mL was stirred during the recordings.

Time resolved fluorescence lifetime measurements for **6**, **5a** + **6**, and *ent*-**5**a + **6** were performed using an Edinburgh Instruments FS5 spectrofluorometer equipped with a 150 W CW ozone-free xenon arc lamp, Czerny-Turner design monochromators, an R928P photomultiplier emission detector, a TCSPC module, and the MCS (phosphorescence) option. More details about the instrument can be found in the literature. [2]

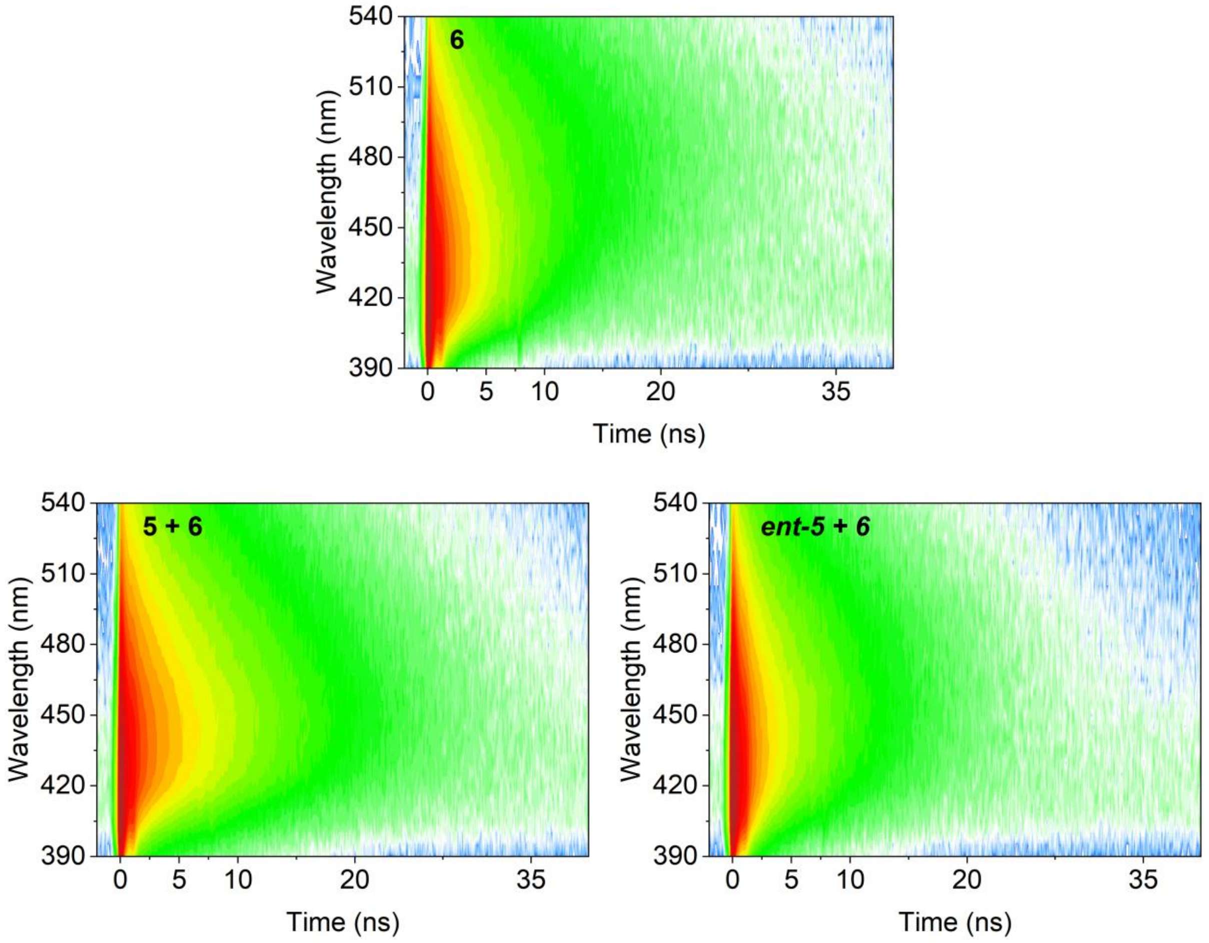

Figure S14: Fluorescence lifetime measurements for **6**, **5a** + **6** and *ent*-**5a** + **6** in $PhCF_3$. (c(**6**) = 125 μM), c(**5a**) = c(*ent*-**5a**) = 2.5 mM).

The sample was placed in a 1 mm cuvette, and fluorescence was collected in reflective mode. A picosecond diode laser with an excitation wavelength of λ_{max} = 375 nm was used to excite the sample, and fluorescence was collected in the range of 390 – 540 nm, with the slit kept at 2 nm. Fluorescence lifetime kinetic traces were fitted using the EasyTau 2 system software from *PICOQUANT*. The results from the fit are shown in the table below.

Table S9: Fluorescence lifetime kinetic traces for **6**, **5a** + **6** and *ent*-**5a** + **6**.

**Sample**	**τ_1 (ns)**	**τ_2 (ns)**
**6**	0.68 ± 0.01	5.04 ± 0.09
**5a** + **6**	0.65 ± 0.07	5.36 ± 0.06
ent-**5a** + **6**	0.59 ±0.08	4.14 ±0.35

References for this section

(1) A. Kumar, P. Malevich, L. Mewes, S. Wu, J. P. Barham, J. Hauer, *J. Chem. Phys.* **2023**, *158*, 144201.

(2) P. M. Stanley, C. Thomas, E. Thyrhaug, A. Urstoeger, M. Schuster, J. Hauer, B. Rieger, J. Warnan, R. A. Fischer, ACS Catal. **2021**, *11*, 871–882.

6. Cyclic Voltammetry

The oxidation potential of model substrate *rac*-**5a** was determined using the standard method and sample preparation (*vide supra*):

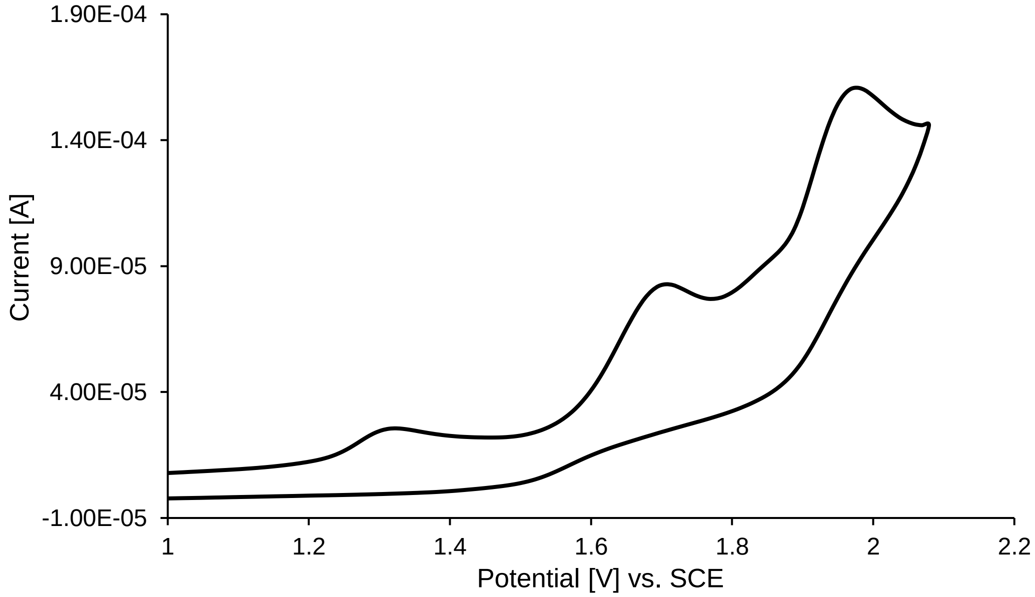

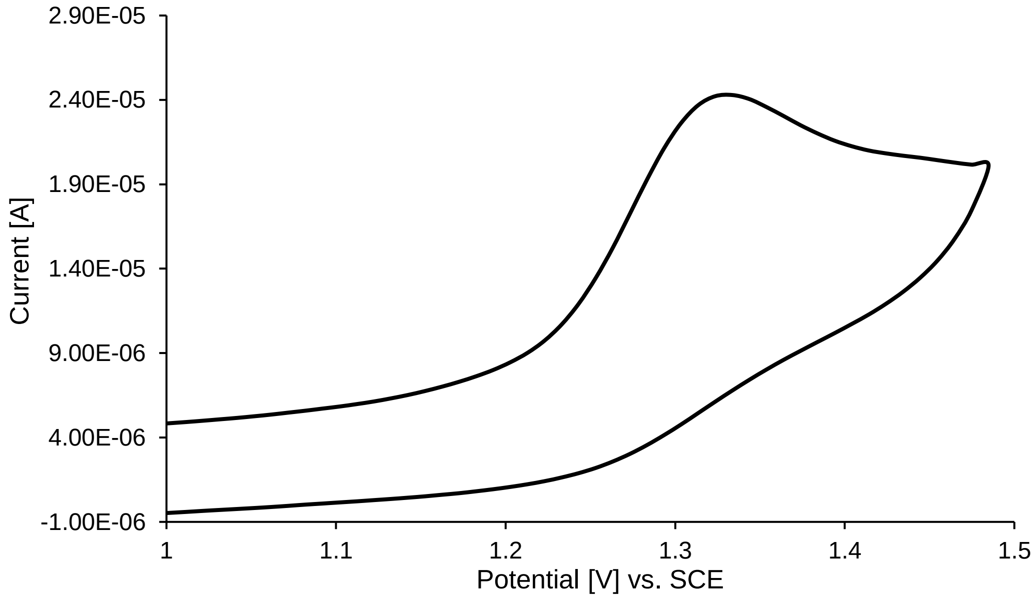

Figure S15: Cyclic voltammograms of *rac*-**5a** (c = 1.0 mM) and $N(^{n}Bu)_4PF_6$ (c = 0.1 M) in MeCN under argon and degassed at 25 °C; 100 mV/s scan rate.

The irreversible oxidation of oxetane *rac*-**5a** can be expected with an $E_{P/2}$ = 1.27 V. Comparison to literature known ground state redox potentials of thioxanthone **8** [3] and the subsequent calculation of excited state redox potentials, reveal that the cleavage of oxetane *via* electron transfer appears to be disfavored. [1,2]

Ground state redox potentials of TXT **8**:

- $E_{\frac{1}{2}(TXT/TXT^-)}$ = –1.62 V (vs. SCE in MeCN)
- $E_{\frac{1}{2}(TXT^-/\mathrm{TXT})}$ = +1.69 V (vs. SCE in MeCN)

Calculated excited state redox potentials of TXT (E_T = 274 kJ/mol)[2]:

- $E_{\frac{1}{2}(TXT^+/TXT^*)}$ = –1.15 V (vs. SCE in MeCN)
- $E_{\frac{1}{2}(TXT^*/TXT^-)}$ = +1.22 V (vs. SCE in MeCN)

References for this section

(1) J. Hofer, F. Pecho, T. Bach, *Synlett* **2023**, *34*, 1063–1067.

(2) N. F. Nikitas, P. L. Gkizis, C. G. Kokotos, *Org. Biomol. Chem.* **2021**, *19*, 5237–5253.

(3) H. J. Timpe, K. P. Kronfeld, *J. Photochem. Photobiol., A* **1989**, *46*, 253−267.

7. Fluorescence Quenching Studies

In order to exclude the involvement of the excited singlet state of thioxanthone **6** in the kinetic resolution of oxetanes, a study of the catalyst's possible fluorescence quenching was investigated. Consequently, a steady-state emission spectrum of thioxanthone **6** was recorded (λ_{em} = 419 nm). Subsequent to the addition of 10.0 equiv. of the better binding matched enantiomer *ent*-**5a**, no decrease in the intensity of thioxanthone **6** fluorescence was observed. This absence of quenching indicates that the excited singlet state of thioxanthone **6** is not involved in the kinetic resolution of oxetanes.

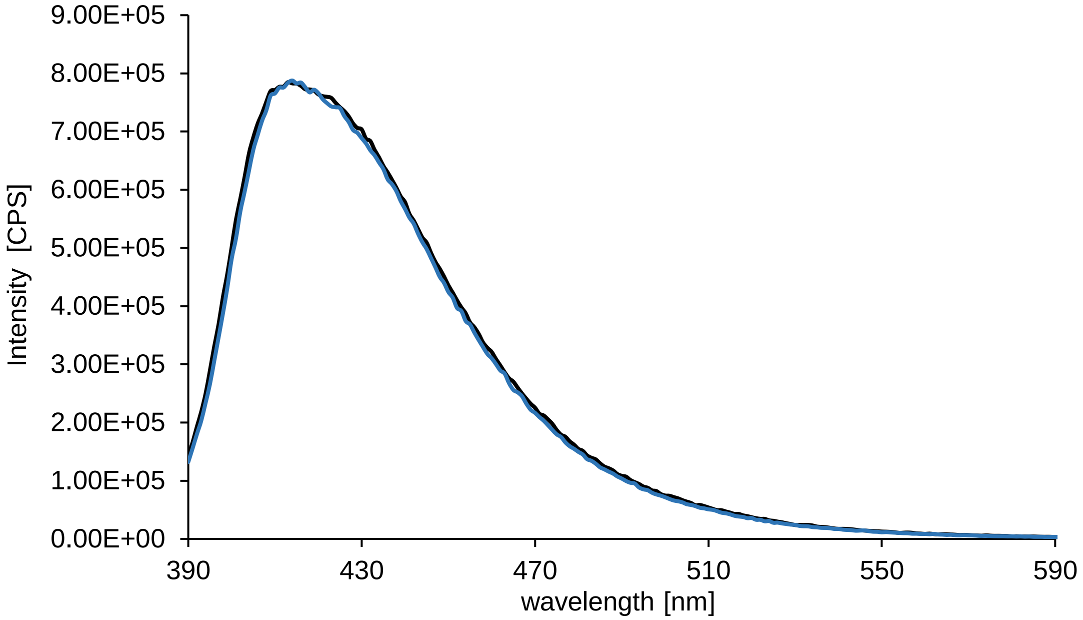

Figure S16: Fluorescence quenching study; steady state emission of thioxanthone **6** (c = 50 µM) in $PhCF_3$ at 25 °C excited at λ = 375nm in the absence (black) or presence of the matched oxetane *ent*-**5a** (0.50 mM, 10.0 equiv.) in $PhCF_3$ at 25 °C (blue).

8. Optimization of the Reaction Conditions

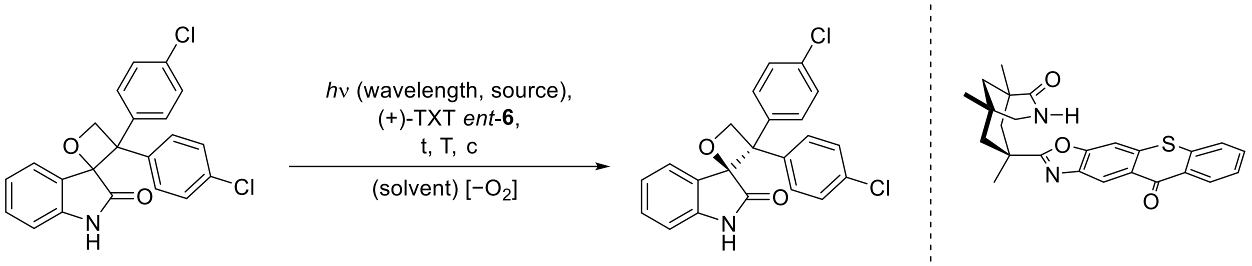

rac-**5a** *ent*-**5a** (+)-TXT *ent*-**6**

Table S10: Optimization of reaction conditions for the enantioselective kinetic resolution of *rac*-**5a** with *ent*-**6**.

#[a]	solvent	T [°C]	*ent*-**6** [mol%]	c [mM]	source	wavelength [nm]	preparation[b]	*ee* [%][c]
1	$PhCF_3$	−25	5.0	2.5	LED	398	A	96
2	*CH_2Cl_2*	−25	5.0	2.5	LED	398	A	75
3	*MeCN*	−25	5.0	2.5	LED	398	A	21
4	*PhH*	*rt*	5.0	2.5	LED	398	A	n.d.
5	*DCE*	−25	5.0	2.5	LED	398	A	79
6	$PhCF_3$	*rt*	5.0	2.5	LED	398	A	93
7	$PhCF_3$	*0*	5.0	2.5	LED	398	A	96
8	$PhCF_3$	−25	*2.5*	2.5	LED	398	A	95
9	$PhCF_3$	−25	*10*	2.5	LED	398	A	97
10	$PhCF_3$	−25	5.0	*5*	LED	398	B	99
11	$PhCF_3$	−25	5.0	*10*	LED	398	B	94
12	$PhCF_3$	−25	5.0	2.5	*LED*	*425*	B	31
13	$PhCF_3$	−25	5.0	2.5	*reactor*	*420*	B	23
14	$PhCF_3$	−25	5.0	2.5	LED	398	B	99
15	$PhCF_3$	−25	5.0	2.5	LED	398	−	88

[a]All reactions were carried out on a 12.5 µmol scale regarding *rac*-**5a** and irradiated for 30 min. [b] Method A: the solution was degassed for 15 min by ultrasonication while sparging the solution with argon gas. Method B: the solution was degassed by a freeze pump thaw (FPT) cycle repeated three times. [c] the enantiomeric excess (*ee*) was calculated from the ratios of (**5a** and *ent*-**5a**) determined by chiral HPLC.

9. Kinetic Profile

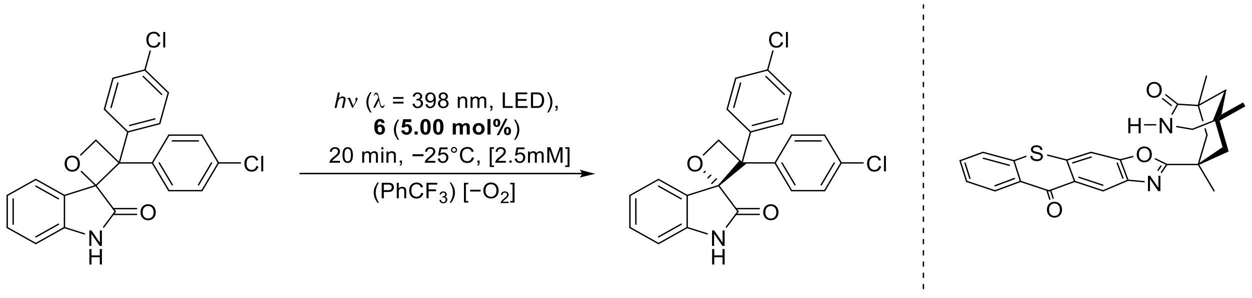

In order to gain a better understanding of the time profile, a kinetic study was conducted to ascertain the complete depletion of one enantiomer. Consequently, the kinetic resolution of *rac*-**5a** was investigated at a 50.0 µmol scale under optimized conditions. The enantiomeric excess was plotted against time as follows:

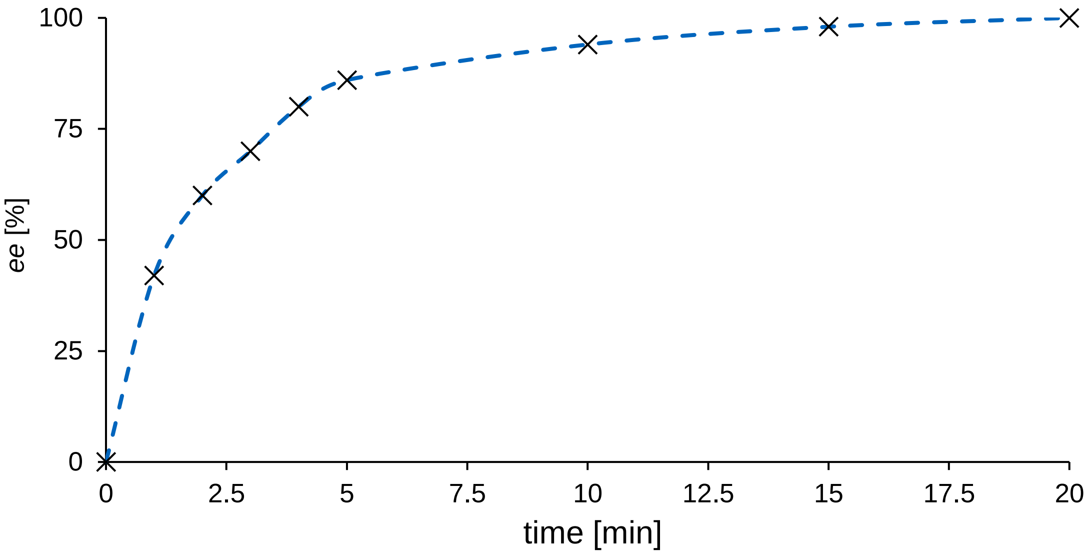

Figure S17: Profile of the enantiomeric excess against time for the kinetic resolution of *rac*-**5a** under optimized reaction conditions.

Despite the fact that complete depletion of *ent*-**5a** is attained after 20 min, a 30-minute irradiation time was selected to ensure that the irradiation conditions are suitable for all substrates.

10. Selectivity Factors for the Kinetic Resolution

Selectivity factors (*s*) were calculated with the conversion (C) and enantiomeric excess (*ee*) obtained in the kinetic resolution experiments for each substrate. Within the boundaries of measurement accuracy of the determined yield (isolated yield) and enantiomeric excess (determined by HPLC), the *s* factors were calculated by:

$$s = \frac{\ln[(1-C)*(1-ee)]}{\ln[(1-C)*(1+ee)]} \quad \text{(Eq. 3)}$$

Since the accuracy of given *s* factors decreases for raising values, *s* factors greater than 100 are given as >100, *s* factors between 40 and 100 are given to the nearest 10 and *s* factors smaller than 40 are reported to the nearest integer.[1]

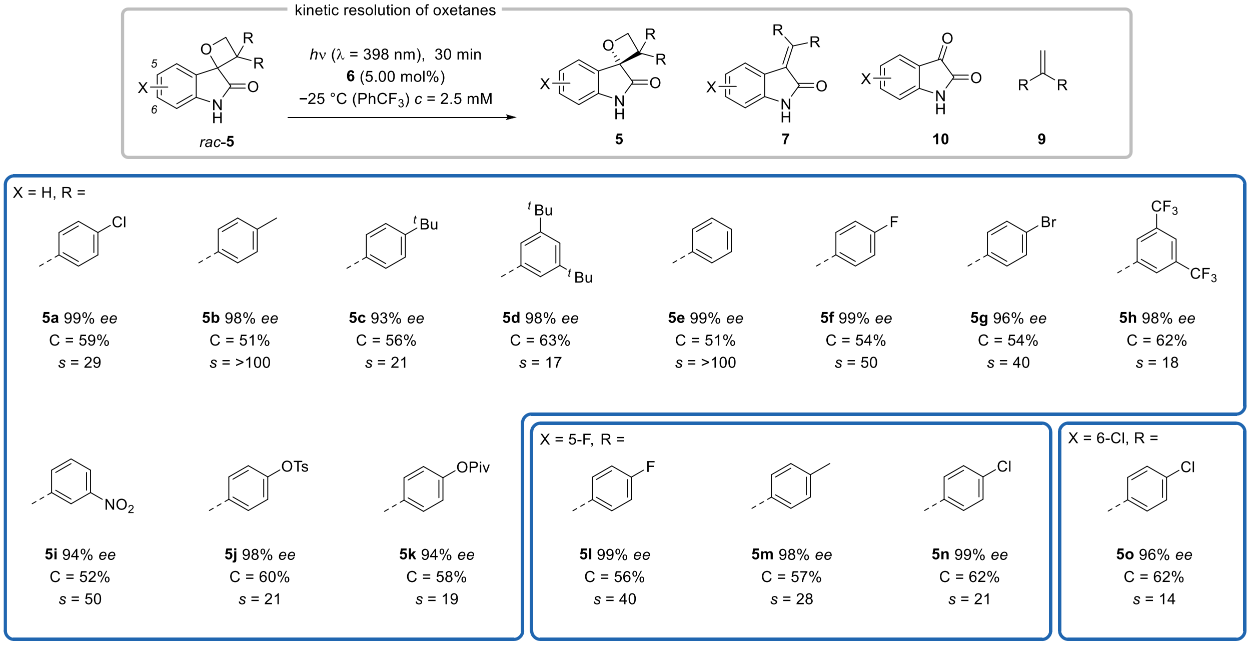

Figure S18: Substrate overview for the kinetic resolution with enantiomeric excess (*ee*), conversion (C), and selectivity factor (*S*).

References for this section:

(1) M. D. Greenhalgh, J. E. Taylor, A. D. Smith, *Tetrahedron* **2018**, *74*, 5554–5560.

11. Catalyst Inhibition Experiments

The photocatalytic kinetic resolution of *rac*-**5** using thioxanthone **6** yields enantioenriched oxetane **5** as well as olefinic fragmentation products **7** and isatin **10**. Since these products are capable of binding to thioxanthone **6** the hypothesis of a selective catalyst inhibition which facilitates the discrimination of **5** was investigated in greater detail.

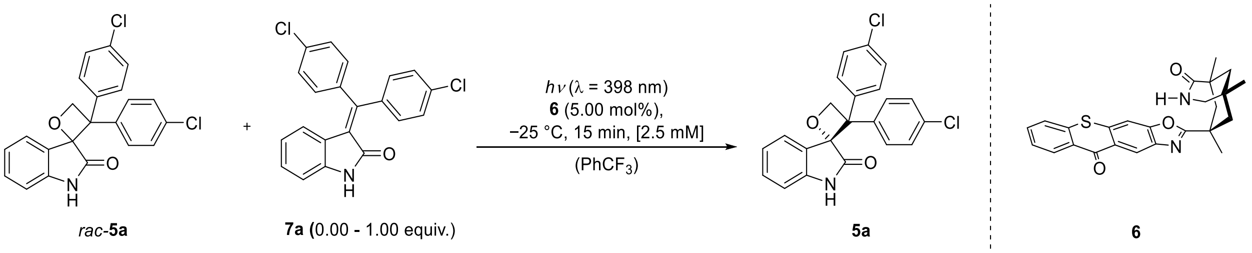

Consequently, the profile of the enantiomeric excess against time of the kinetic resolution of *rac*-**5a** under standard reaction conditions was investigated with rising equivalents of olefin **7a** added already at the beginning of the reaction. Following *ee* vs. time profiles were obtained:

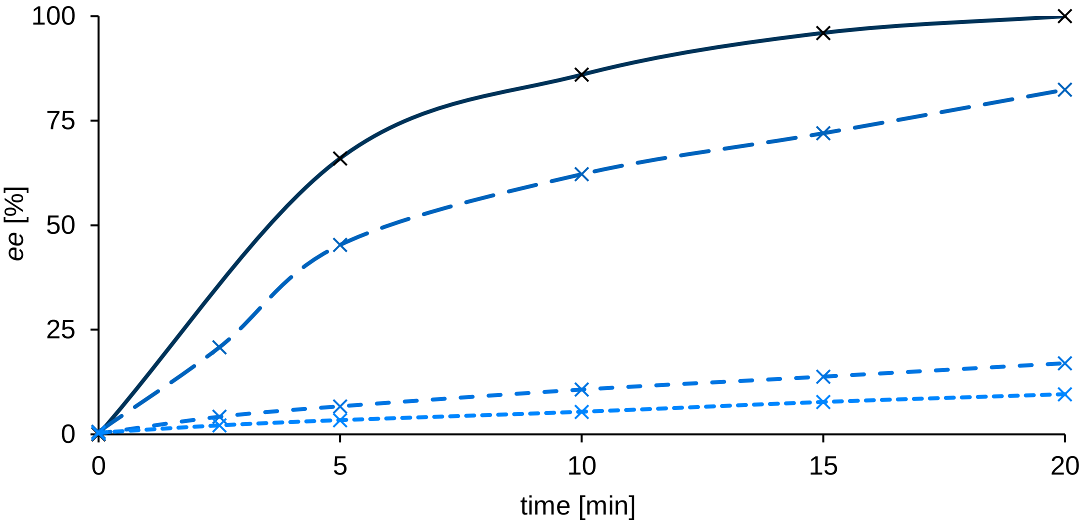

Figure S19: Profiles for the kinetic resolution of *rac*-**5a** under optimized reaction conditions with rising amounts of **7a** (—×— = 0.00 equiv.; —×— = 0.10 equiv.; - × - = 0.50 equiv.; --×-- = 1.00 equiv.) added from the start of the reaction.

12. NMR-Titration Experiments

For a comprehensive overview of the theoretical background of NMR titrations, along with the practical implementation for determining dimerization (K_{dim}) and association (K_a) constants, please refer to the supplementary information of our previous study.[1] Based on its properties regarding chiral HPLC and solvation properties, substrate *rac*-**5a** was selected as a model substrate. The corresponding enantiomers were separated on a semi-preparative HPLC.

Determination of the dimerization constant K_{dim} for oxetane 5a.

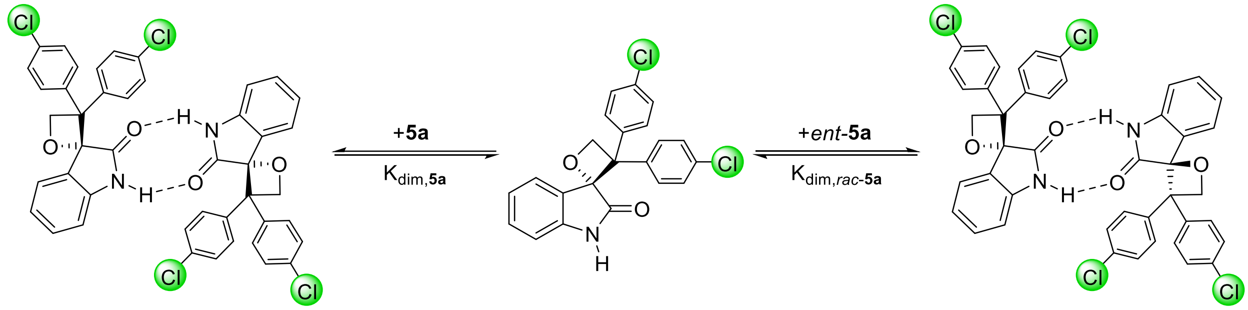

[5a]₂ **5a** **[5a • ent-5a]**

The equilibrium constants were determined by a dilution series with subsequent monitoring of the NH-proton by ^{1}H-NMR. The chemical shift varies between the free monomer and the dimeric form, which is formed through hydrogen bonding and is highly dependent on the substrate concentration. It is noteworthy that three distinct dimers are possible, namely [**5a**]$_2$, [*ent*-**5a**]$_2$, and [**5a**·*ent*-**5a**]. While all three dimers can form in solution, only two of these dimers have their dimerization constants quantifiable.

- dimerization of the same enantiomers [**5a**]$_2$ or [*ent*-**5a**]$_2$ with $K_{dim,\mathbf{5a}} = K_{dim,\mathit{ent}\text{-}\mathbf{5a}}$
- dimerization of the opposite enantiomers [**5a** · *ent*-**5a**] with $K_{dim,\mathit{rac}\text{-}\mathbf{5a}}$

Both of these dimerization constants were determined as followed:

Given that the calculation of the dimerization constant necessitates data obtained at substrate concentrations within the range of 0.1 K_{dim}^{-1} to 10 K_{dim}^{-1} to obtain reliable dimerization constants, a preliminary estimation of the anticipated dimerization constant was conducted. This estimation

was derived from the analysis of previous case studies done within our research group.[2,3] For compound *rac*-**5a** a dimerization constant of 20 L/mol was used to calculate the dilution series. A total of 15 samples of *rac*-**5a** and **5a** were prepared with concentrations ranging from $2.06 \cdot 10^{-3}$ mol/L to $2.06 \cdot 10^{-1}$ mol/L. All samples were measured in benzene-d_6 at a constant temperature of 298 K. The concentration dependent chemical shifts for both titrations are depicted below:

Entry	[*rac*-**5a**]$_0$ [mol/L]	δ_{NH} [ppm]
1	2.060E-03	5.886
2	2.884E-03	5.940
3	4.017E-03	6.061
4	5.408E-03	6.162
5	7.725E-03	6.334
6	1.082E-02	6.498
7	1.494E-02	-*
8	2.060E-02	6925
9	2.884E-02	7.164
10	4.017E-02	7.431
11	5.665E-02	7.697
12	7.725E-02	7.919
13	1.082E-01	8.166
14	1.494E-01	8.388
15	2.060E-01	8.588

*signals cannot be assigned due to overlaps

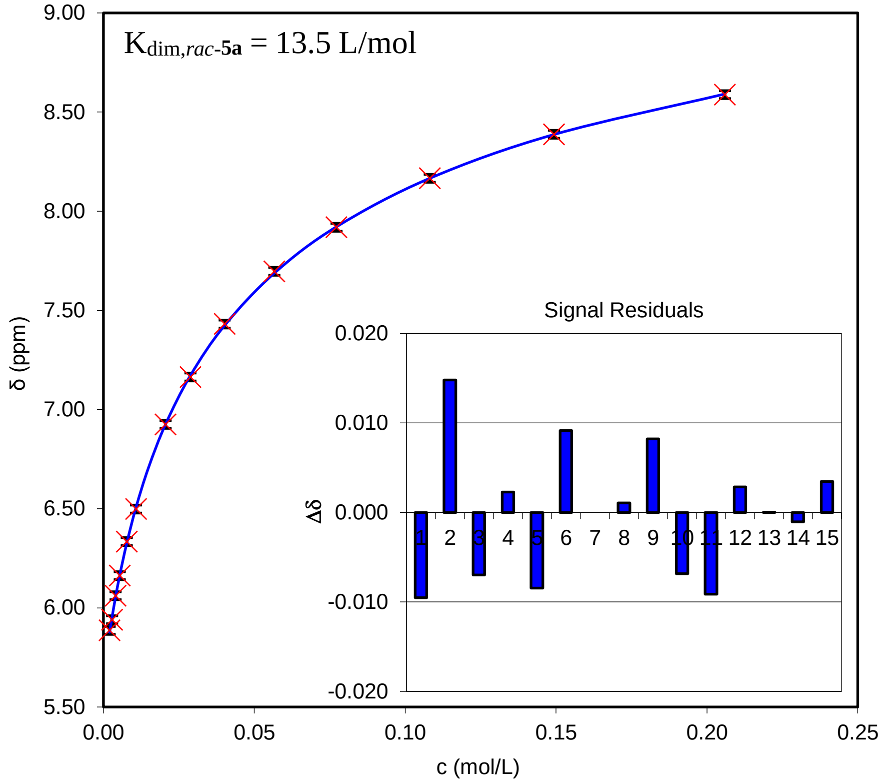

Entry	[**5a**]$_0$ [mol/L]	δ_{NH} [ppm]
1	2.100E-03	6.010
2	2.940E-03	6.106
3	4.095E-03	6.209
4	5.513E-03	6.325
5	7.875E-03	6.490
6	1.103E-02	6.679
7	1.523E-02	6.882
8	2.100E-02	7.123
9	2.940E-02	7.360
10	4.095E-02	7.617
11	5.775E-02	7.882
12	7.875E-02	8.111
13	1.103E-01	8.349
14	1.523E-01	8.563
15	2.100E-01	8.753

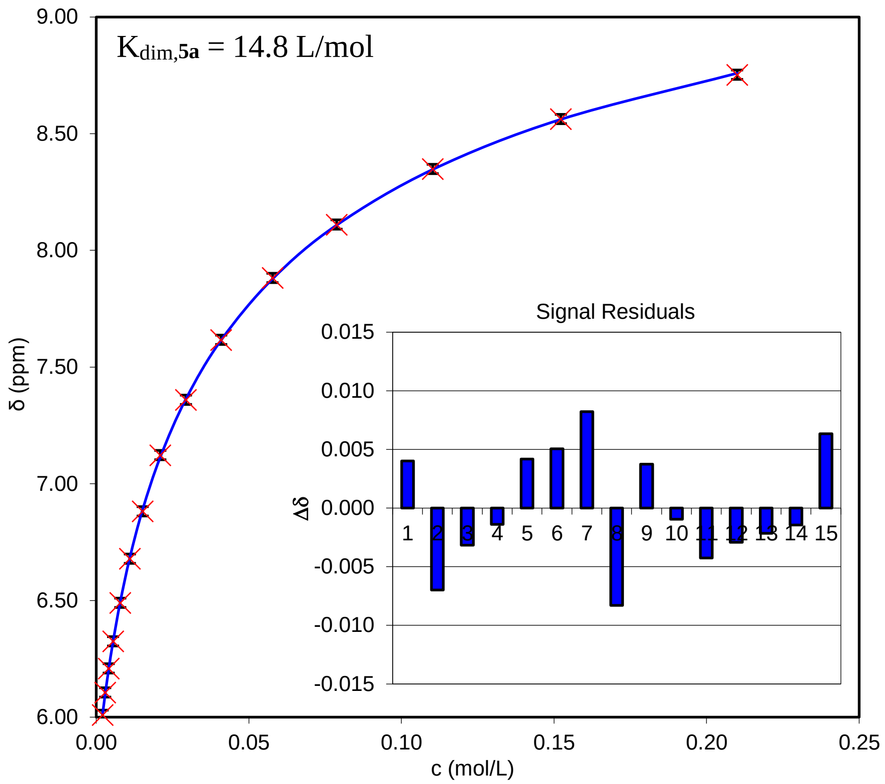

For both titrations, a best fit curve was determined on the basis of the described model with defined boundary conditions using non-linear regression. The following dimerization constants were found:

- $K_{dim,\mathit{rac}\text{-}\mathbf{5a}}$ = 13.5. L/mol
- $K_{dim,\mathbf{5a}}$ = 14.8 L/mol

Both titrations confirm that within the limits of measurement accuracy the boundary condition of $K_{dim,\mathit{rac}\text{-}\mathbf{5a}} = K_{dim,\mathbf{5a}}$ is valid.

The robustness of the best fit curve was confirmed by *Monte-Carlo* analysis.[1,4] 1000 datasets were calculated in which each individual data point of the original dataset deviates randomly with a normal distribution, using the standard deviation observed in the original dataset, around the best fit values (σ = 0.008 ppm, *rac*-**5a**; σ = 0.006 ppm, **5a**). Based on this, dimerization constants were

obtained for each set of calculated titrations. Subsequently, a mean, the standard deviation and a 95% confidence interval were calculated as shown:

Table S10: Robustness data from *Monte-Carlo* analysis.

#	Mean [L/mol]	σ [L/mol]	95% CI [L/mol]
rac-**5a**	13.5	0.2	13.5 ± 0.5
**5a**	14.8	0.2	14.8 ± 0.3

Based on this preliminary work, a value of K_{dim} = 13.5 L/mol was used for the determination of the association constants.

Determination of the association constants K_a between 5a and ent-5a with chiral thioxanthone 6

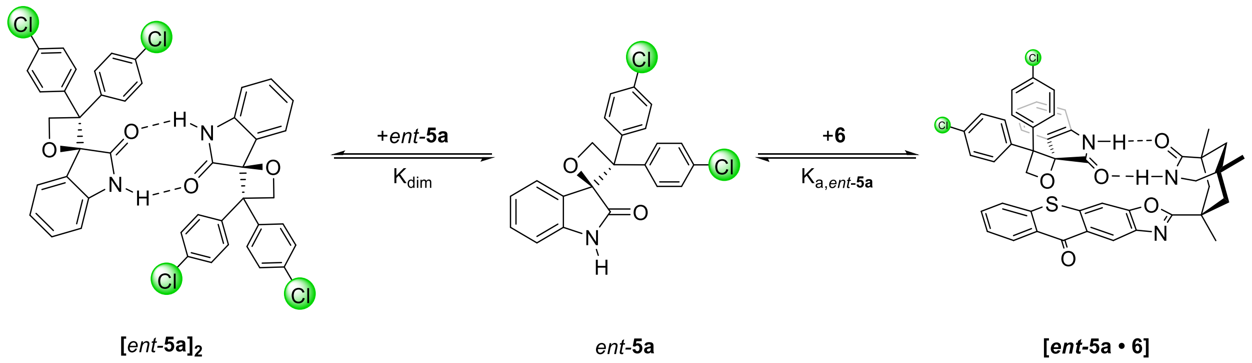

Since the investigated thioxanthone **6** possesses a lactam two-point hydrogen bonding site, the association constants K_a between the catalyst **6** and the single enantiomers **5a** and *ent*-**5a** can be determined as already described for the dimerization studies. Therefore, the concentration of thioxanthone catalyst **6** was kept constant at $4.00 \cdot 10^{-4}$ mol/L and its proton NH chemical shift was monitored upon varying the concentrations of **5a** and *ent*-**5a** (0.00 mol/L – $9.00 \cdot 10^{-2}$ mol/L) for two different sets of titrations. The concentration dependent chemical shifts for both titrations are depicted below:

Entry	[*ent*-**5a**]$_0$ [mol/L]	δ_{NH} [ppm]
1	0.00E+00	3.938
2	9.00E-04	4.402
3	1.62E-03	4.704
4	2.81E-03	5.094
5	5.06E-03	5.559
6	9.00E-03	6.001
7	1.37E-02	-*
8	3.15E-02	-*
9	5.06E-02	7.702
10	9.00E-02	8.189

*signals cannot be assigned due to overlaps

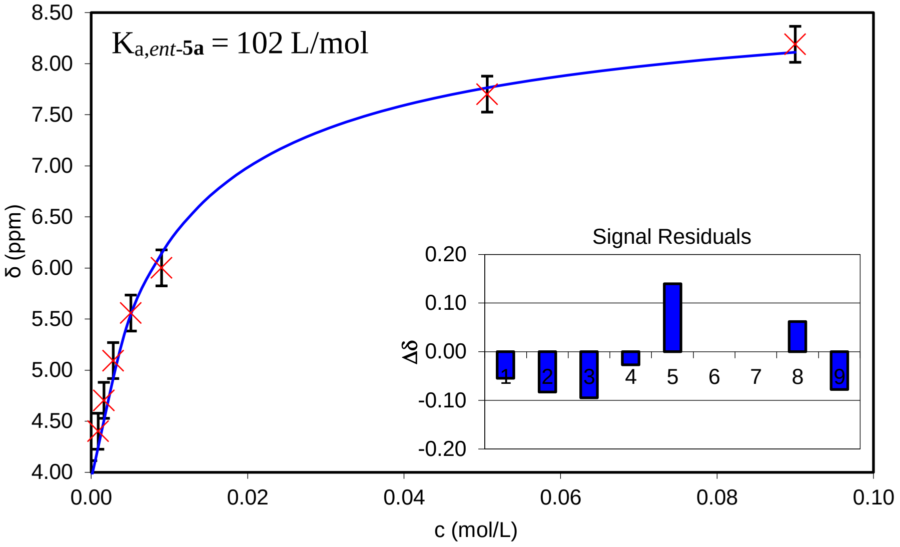

Entry	[**5a**]$_0$ [mol/L]	δ_{NH} [ppm]
1	0.00E+00	3.950
2	9.00E-04	3.968
3	1.62E-03	3.990
4	2.81E-03	4.026
5	5.06E-03	4.080
6	9.00E-03	4.159
7	1.62E-02	4.262
8	2.81E-02	4.416
9	5.06E-02	4.555
10	9.00E-02	4.661

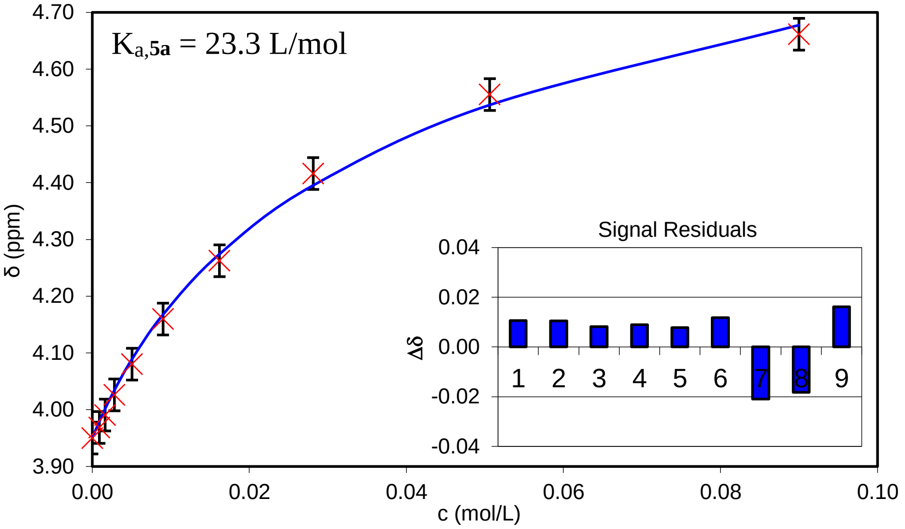

For both titrations, a best fit curve was determined on the basis of the described model with defined boundary conditions using non-linear regression. The following association constants were determined:

- $K_{a,\textit{ent}\text{-}\mathbf{5a}}$ = 102 L/mol
- $K_{a,\mathbf{5a}}$ = 23.3 L/mol

The robustness of the best fit curve was confirmed by *Monte-Carlo* analysis,[1,4] by calculating 1000 datasets in which each individual data point deviates randomly with a normal distribution

using the standard deviation observed in the corresponding dataset around the best fit values (σ = 0.088 ppm, *rac*-**5a**; σ = 0.014 ppm, **5a**). Based on this, association constants were obtained for each set of calculated titrations. Subsequently, a mean, the standard deviation and a 95% confidence interval can be calculated as shown:

Table S11: Robustness data from *Monte-Carlo* analysis.

#	Mean [L/mol]	σ [L/mol]	95% CI [L/mol]
ent-**5a**	101	8.10	101 ± 22.6
**5a**	23.4	0.094	23.4 ± 5.7

Determination of the dimerization constant K_{dim} for olefin 7a.

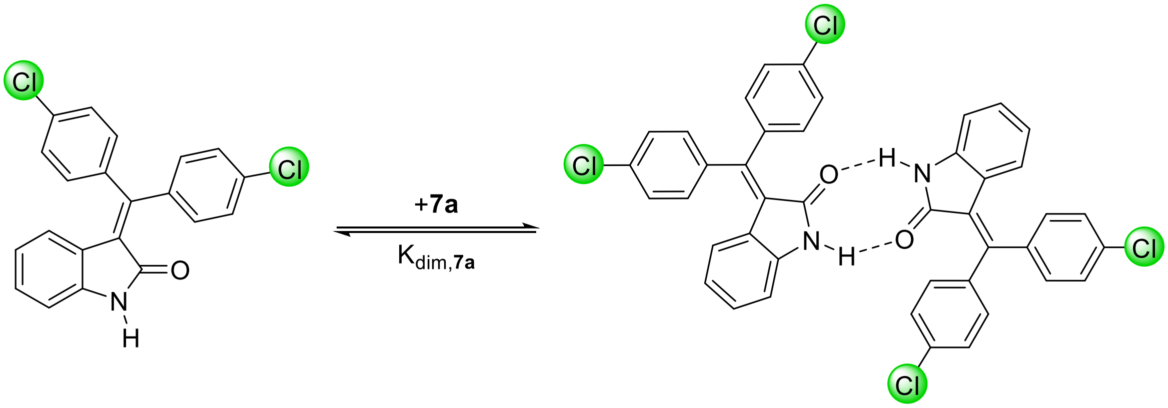

[7a] **[7a]$_2$**

Since olefin **7a** is not chiral, it forms only one possible dimer **[7a]**$_2$ in solution and therefore only one dimerization constant needs to be determined. For compound **7a** a dimerization constant of 15 L/mol was suggested to calculate the dilution series and prepare 15 samples of **7a** with concentrations ranging from $1.20 \cdot 10^{-3}$ mol/L to $1.20 \cdot 10^{-1}$ mol/L. All samples were measured in benzene-d_6 at a constant temperature of 298 K. The concentration dependent chemical shifts for the titration are depicted below:

Entry	[**7a**]$_0$ [mol/L]	δ_{NH} [ppm]
1	1.20E-03	6.362
2	1.68E-03	6.475
3	2.34E-03	6.605
4	3.15E-03	6.744
5	4.50E-03	6.962
6	6.30E-03	7.205
7	8.70E-03	7.463
8	1.20E-02	7.723
9	1.68E-02	8.021
10	2.34E-02	8.326
11	3.30E-02	8.621
12	4.50E-02	8.890
13	6.30E-02	9.148
14	8.70E-02	9.379
15	1.20E-01	9.579

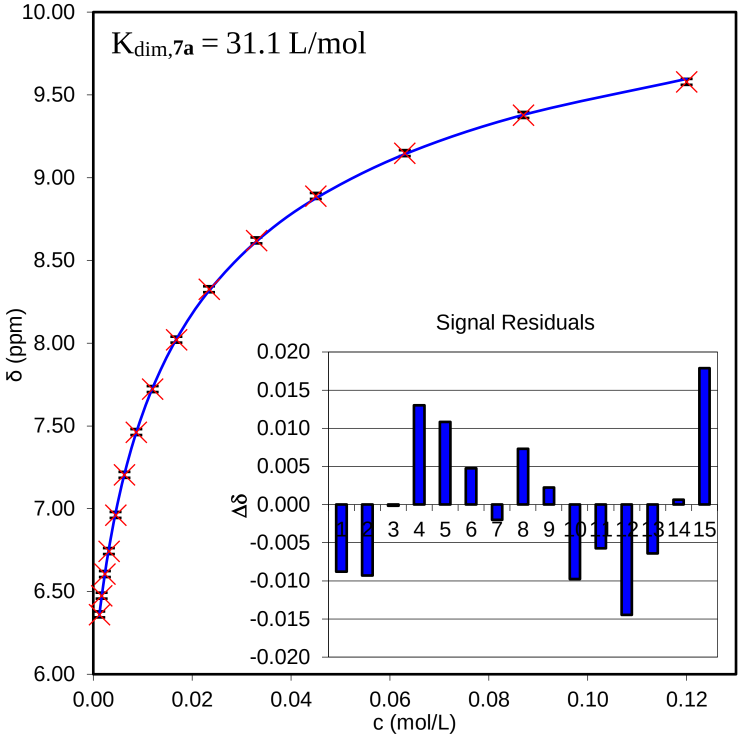

For the titration a best fit curve was determined on the basis of the described model with defined boundary conditions using non-linear regression. For olefin **7a** the following dimerization constant was found:

- $K_{dim,\mathbf{7a}}$ = 31.1 L/mol

The robustness of the best fit curve was confirmed by *Monte-Carlo* analysis,[1,4] by calculating 1000 datasets in which every individual data point deviates randomly with a normal distribution using the standard deviation observed in the corresponding dataset around the best fit values (σ = 0.009 ppm, **7a**). Based on this, dimerization constants were obtained for each set of calculated titrations. Subsequently, a mean, the standard deviation and a 95% confidence interval can be calculated as shown:

Table S12: Robustness data from *Monte-Carlo* analysis.

#	Mean [L/mol]	σ [L/mol]	95% CI [L/mol]
**7a**	31.1	0.5	31.1 ± 1.2

Determination of the association constant K_a between 7a with chiral thioxanthone 6

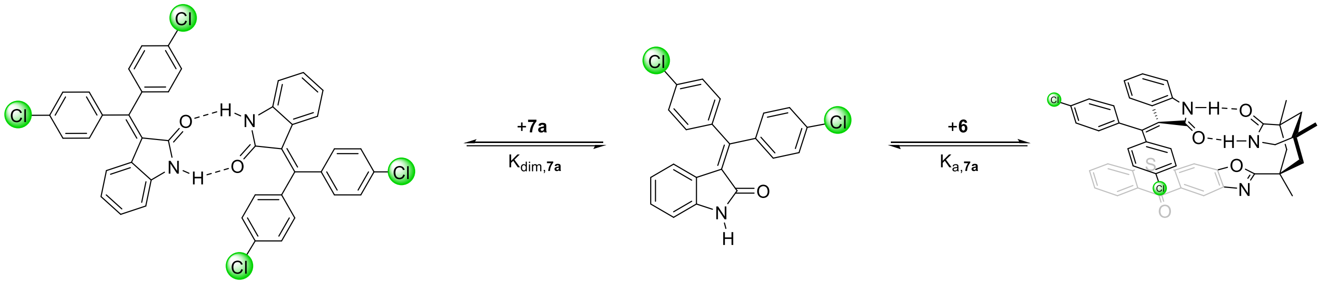

Analog to the previous titrations for determining the association constants $K_{a,\mathbf{5a}}$ and $K_{a,\mathit{ent}\text{-}\mathbf{5a}}$, thioxanthone **6** was titrated against various concentrations of olefin **7a**. Therefore, the concentration of thioxanthone catalyst **6** was kept constant at $4.00 \cdot 10^{-4}$ mol/L and its proton NH chemical shift was monitored upon varying the concentrations of **7a** (0.00 mol/L – $1.20 \cdot 10^{-1}$ mol/L). The concentration dependent chemical shifts are depicted below:

Entry	[**7a**]$_0$ [mol/L]	δ_{NH} [ppm]
1	0.00E+00	3.949
2	1.20E-03	5.071
3	2.16E-03	5.623
4	3.75E-03	6.179
5	6.75E-03	6.891
6	1.20E-02	-*
7	2.16E-02	8.128
8	3.75E-02	8.502
9	6.75E-02	8.918
10	1.20E-01	9.252

*signals cannot be assigned due to overlaps

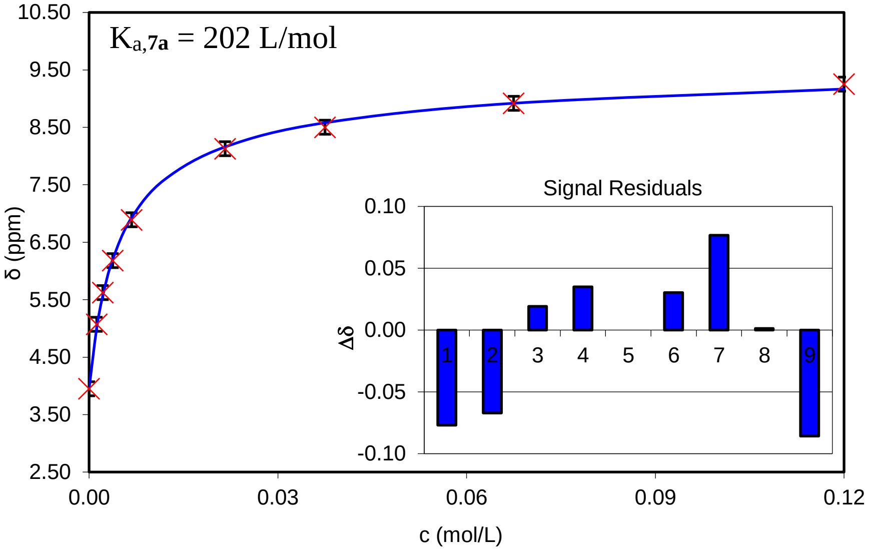

For both titrations, a best fit curve was determined on the basis of the described model with defined boundary conditions using non-linear regression. Following association constants were found:

- $K_{a,\mathbf{7a}}$ = 202 L/mol

The robustness of the best fit curve was confirmed by *Monte-Carlo* analysis,[1,4] by calculating 1000 datasets in which every individual data point deviates randomly with a normal distribution using the standard deviation observed in the corresponding dataset around the best fit values (σ = 0.061 ppm, **7a**). Based on this, association constants were obtained for each set of calculated titrations. Subsequently a mean, the standard deviation and a 95% confidence interval can be calculated as followed:

Table S13: Robustness data from *Monte-Carlo* analysis.

#	Mean [L/mol]	σ [L/mol]	95% CI [L/mol]
**7a**	196	9.2	196 ± 23.7

References for this section:

(1) A. Bakowski, M. Dressel, A. Bauer, T. Bach, *Org. Biomol. Chem.* **2011**, *9*, 3516–3529.

(2) T. Kratz, P. Steinbach, S. Breitenlechner, G. Storch, C. Bannwarth, T. Bach, *J. Am. Chem. Soc.* **2022**, *144*, 10133–10138.

(3) X. Li, R. J. Kutta, C. Jandl, A. Bauer, P. Nuernberger, T. Bach, *Angew. Chem. Int. Ed.* **2020**, *59*, 21640–21647.

(4) J. S. Alper, R. I. Gelb, *J. Phys. Chem.* **1990**, *94*, 4747–4751.

13. Determination of the Absolute Configuration

Since oxetanes **5** constantly precipitated from solution as amorphous solids during crystallization attempts, it proved unfeasible for this class of compounds to crystalize an enantiomerically pure sample for the determination of the absolute configuration *via* single crystal XRD. Therefore, a chiral complexing agent was utilized in a qualitative ^{1}H-NMR titration study to ascertain the configuration of the selected model substrate **5a**. The method is based on a chiral 7-substituted 3-azabicyclo[3.3.1]nonan-2-one which was previously used in our group as a chiral complexing agent to induce enantioselectivity in numerous reactions.[1,2] For detailed information on the method of assigning the absolute configuration with such a complexing agent it is referred to a previous publication from our group.[1] Since the 7-substituted 3-azabicyclo-[3.3.1]nonan-2-one **S7** possesses a two point hydrogen binding site, it is capable of binding oxetane *ent*-**5a** in a similar fashion as thioxanthone **6**.

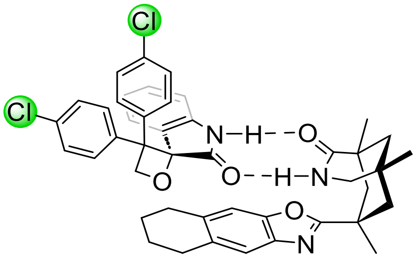

[*ent*-**5a** • **S7]**

For two molecules of the same enantiomer of **S7**, the sterically demanding shields prevent a close approach of the two lactam binding sites and therefore no self-dimerization is observed for such homochiral pairs [**S7**]$_2$ and [*ent*-**S7**]$_2$. In contrast, the heterochiral pair [**S7** · *ent*-**S7**] is ideally suited for dimerization by two hydrogen bonds. Consequently, the NH proton shows a significant change in chemical shift in contrast to the homochiral pair. This behavior is utilized for the determination of the absolute configuration of **5a**. By artificially generating the pseudo-homochiral pair [**5a** · **S7**] and pseudo-heterochiral pair [**5a** · *ent*-**S7**] the letter should show the expected change in chemical shift, whereas no significant change in chemical shift is expected for [**5a** · **S7**].

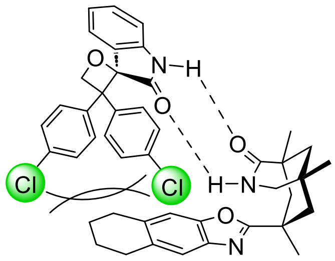

[5a] + **[S7]**
pseudo-homochiral

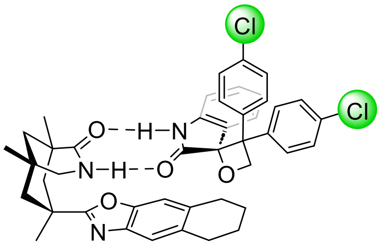

[5a • *ent*-**S7]**
pseudo-heterochiral

For the NMR experiments, two samples of **5a** are prepared in benzene-d_6. To each sample 0.50 equiv. of a single enantiomer of the chiral complexing agent **S7** / *ent*-**S7** is added and a ^{1}H-NMR spectrum is measured. This procedure is repeated for two more times until every sample contains 1.50 equiv. of the respective chiral complexing agent. For the pseudo-homochiral pair following NMR overlay was generated:

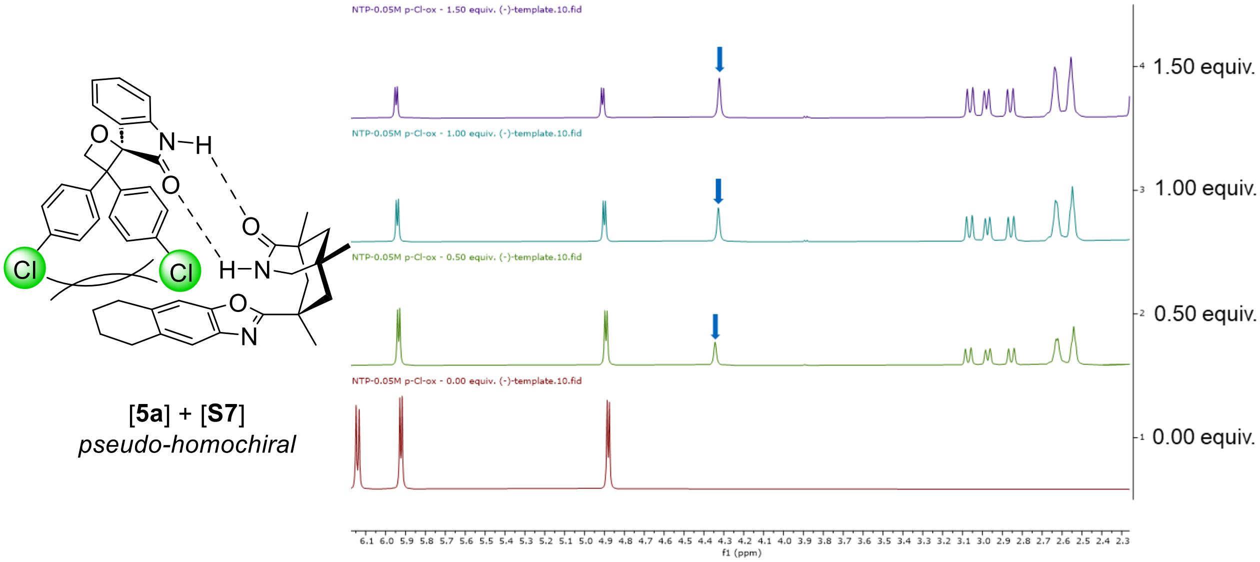

[5a] + **[S7]**
pseudo-homochiral

The blue arrows indicate the corresponding NH signal of chiral complexing agent **S7** for raising concentrations of **S7**. As expected, no change in chemical shift is observed for the pseudo-homochiral pair as no binding occurs between substrate **5a** and chiral complexing agent **S7**. Only free **S7** is in solution. For the pseudo-heterochiral NMR experiment following overlay was generated:

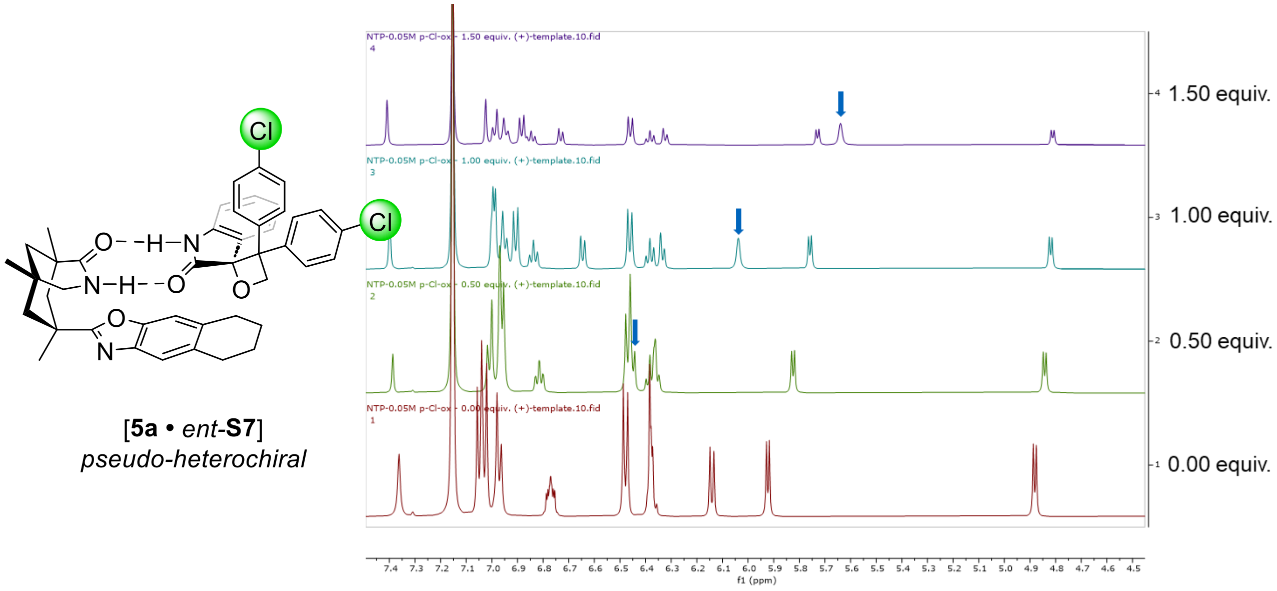

[**5a** • *ent*-**S7**]
pseudo-heterochiral

The blue arrows indicate the corresponding NH signal of chiral complexing agent *ent*-**S7** for raising concentrations in steps of 0.50 equiv.. As expected for the pseudo-heterochiral pair an overall upfield shift for the corresponding NH signal is observed. For sub stoichiometric amounts of *ent*-**S7** the majority of the chiral complexing agent is bound to **5a**. Consequently, its signal is shifted strongly downfield in comparison to free *ent*-**S7**. By further addition of *ent*-**S7**, the equilibrium between bound and free *ent*-**S7** is shifted towards an increased amount of free *ent*-**S7**. Therefore, the signal is starting to shift upfield again. With the literature known absolute configuration of **S7** and *ent*-**S7** the stereocenter at C3 position of **5a** can be set as followed:

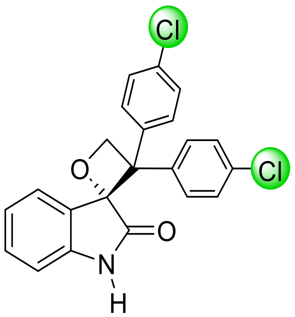

References for this section:

(1) A. Bauer, T. Bach, *Tetrahedron: Asymmetry* **2004**, *15*, 3799–3803.

(2) M. de Robichon, T. Kratz, F. Beyer, J. Zuber, C. Merten, T. Bach, *J. Am. Chem. Soc.* **2023**, *145*, 24466–24470.

14. Analytical Data

Substrate Synthesis:

3,5-Di-*tert*-butylbenzaldehyde (S2)

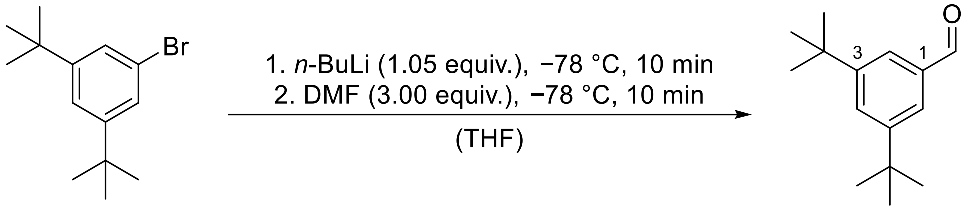

S2
$C_{15}H_{22}O$
M = 218.34g/mol

For formylation 5.00 g of aryl bromide (18.6 mmol, 1.00 equiv.) are dissolved in 100 mL THF and cooled to −78 °C. 7.80 mL of a solution of *n*-BuLi in hexanes [2.50 mM] (19.5 mmol, 1.05 equiv.) were added dropwise to the stirred solution. After the complete addition, the reaction mixture was stirred for 10 min and 4.31 mL DMF (55.7 mmol, 3.00 equiv.) were added in one portion. After stirring for further 10 min at −78 °C, 10 mL of water were added and the mixture was warmed to room temperature. The crude mixture was further diluted with water and extracted with EtOAc (3 × 150 mL). The combined organic layers were washed with brine, dried over Na_2SO_4 and filtered. Any volatiles were removed under reduced pressure and the crude product was purified by flash column chromatography. 3.95 g of aldehyde **S2** (18.1 mmol) were obtained as a white solid (97%).

Analytical data match those reported in literature.[4]

TLC: 0.61 (Hex:EtOAc = 9:1) [UV], [$KMnO_4$]

M.p.: 95 °C

^{1}H-NMR (500 MHz, DMSO-d_6, 300 K) δ [ppm] = 10.00 (s, 1H, CHO), 7.75 (s, 3H, CH-2 / CH-4), 1.32 (s, 18H, CH_3).

^{13}C-NMR (125 MHz, DMSO-d_6, 300 K) δ [ppm] = 193.7 (CHO), 151.5 (2C, C3), 136.1 (C1), 128.6 (C4), 123.7 (2C, C2), 34.7 [2C, $C(CH_3)_3$], 31.1 [6C, $C(CH_3)_3$].

Bis(4-(*tert*-butyl)phenyl)methanol (S3a)

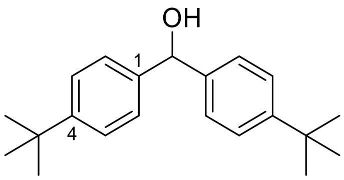

S3a
$C_{21}H_{28}O$
M = 296.45 g/mol

Alcohol **S3a** was synthesized according to **GP-A** from commercially available 4-(*tert*-butyl)benzaldehyde (3.50 g, 21.6 mmol, 1.00 equiv.). After purification 6.02 g **S3a** (20.3 mmol, 94%) were obtained as a white solid.

Analytical data match those reported in literature.[5]

TLC: 0.70 (Hex:EtOAc = 8:2) [UV], [$KMnO_4$]

M.p.: 111 °C

^{1}H-NMR (500 MHz, $CDCl_3$, 300 K) δ [ppm] = 7.39 – 7.35 (m, 4H, CH-3) 7.35 – 7.31 (m, 4H, CH-2), 5.81 (d, ^3J = 3.4 Hz, 1H, C*H*OH), 2.17 (d, ^3J = 3.4 Hz, 1H, CHO*H*), 1.32 (s, 18H, CH_3).

^{13}C-NMR (125 MHz, DMSO-d_6, 300K) δ [ppm] = 148.9 (2C, C4), 143.0 (2C, C1), 125.9 (4C, C2), 124.8 (4C, C3), 74.0 (1C, COH), 34.1 [2C, $C(CH_3)_3$], 31.2 [6C, $C(CH_3)_3$].

Bis(3,5-di-*tert*-butylphenyl)methanol (S3b)

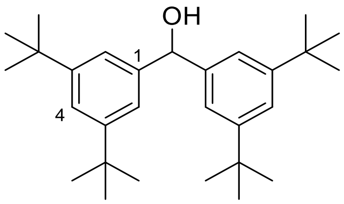

S3b

$C_{29}H_{44}O$

M = 408.67 g/mol

Alcohol **S3b** was synthesized according to **GP-A** from aldehyde **S2** (3.90 g, 17.9 mmol, 1.00 equiv.). After purification 4.11 g **S3b** (10.1 mmol, 56%) were obtained as a white solid.

Analytical data match those reported in literature.[6]

TLC: 0.85 (Hex:EtOAc = 8:2) [UV], [$KMnO_4$]

M.p.: 147 °C

^{1}H-NMR (500 MHz, $CDCl_3$, 300 K) δ [ppm] = 7.34 (t, ^4J = 1.9 Hz, 2H, CH-4) 7.27 (d, ^4J = 1.9 Hz, 4H, CH-2), 5.85 (d, ^3J = 3.7 Hz, 1H, C*H*OH), 2.23 (d, ^3J = 3.7 Hz, 1H, CHO*H*), 1.32 (s, 36H, CH_3).

^{13}C-NMR (125 MHz, DMSO-d_6, 300 K) δ [ppm] = 149.5 (4C, C3), 145.0 (2C, C1), 120.4 (4C, C2), 119.9 (2C, C4), 75.5 (1C, COH), 34.5 [4C, *C*(CH_3)$_3$], 31.3 [12C, C(*C*H_3)$_3$].

Bis(4-(*tert*-butyl)phenyl)methanone (S4a)

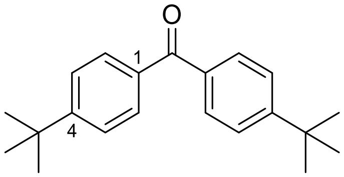

S4a

$C_{21}H_{26}O$

M = 294.44 g/mol

Benzophenone **S4a** was synthesized by oxidation according to **GP-B** from alcohol **S3a** (6.00 g, 20.2 mmol, 1.00 equiv.). After purification 3.98 g **S4a** (13.5 mmol, 67%) were obtained as a white solid.

Analytical data match those reported in literature.[5]

TLC: 0.93 (Hex:EtOAc = 8:2) [UV], [$KMnO_4$]

M.p.: 120 °C

^{1}H-NMR (500 MHz, DMSO-d_6, 300 K) δ [ppm] = 7.70 – 7.66 (m, 4H, CH-3) 7.60 – 7.55 (m, 4H, CH-2), 1.32 (s, 18H, CH_3).

^{13}C-NMR (125 MHz, DMSO-d_6, 300 K) δ [ppm] = 195.1 (CO), 155.6 (2C, C4), 134.6 (2C, C1), 129.7 (4C, C2), 125.4 (4C, C3), 34.9 [2C, $C(CH_3)_3$], 30.9 [6C, $C(CH_3)_3$].

Bis(3,5-di-*tert*-butylphenyl)methanone (S4b)

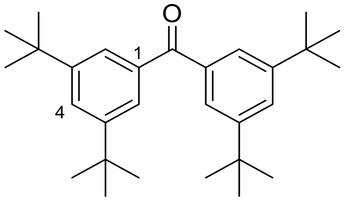

S4b
$C_{29}H_{42}O$
M = 406.65 g/mol

Benzophenone **S4b** was synthesized by oxidation according to **GP-B** from alcohol **S3b** (4.00 g, 9.79 mmol, 1.00 equiv.). After purification 2.04 g **S4b** (5.02 mmol, 51%) were obtained as a white solid.

Analytical data match those reported in literature.[6]

TLC: 0.90 (Hex:EtOAc = 8:2) [UV], [$KMnO_4$]

M.p.: 151 °C

^{1}H-NMR (500 MHz, $CDCl_3$, 300 K) δ [ppm] = 7.67 (d, ^4J = 1.9 Hz, 4H, CH-2) 7.65 (t, ^4J = 1.9 Hz, 2H, CH-4), 1.35 (s, 36H, CH_3).

^{13}C-NMR (125 MHz, $CDCl_3$, 300 K) δ [ppm] = 198.1 (CO), 150.8 (4C, C3), 137.4 (2C, C1), 126.5 (2C, C4), 124.8 (4C, C2), 35.2 [4C, $C(CH_3)_3$], 31.6 [12C, $C(CH_3)_3$].

Carbonylbis(4,1-phenylene) bis(2,2-dimethylpropanoate) (S4c)

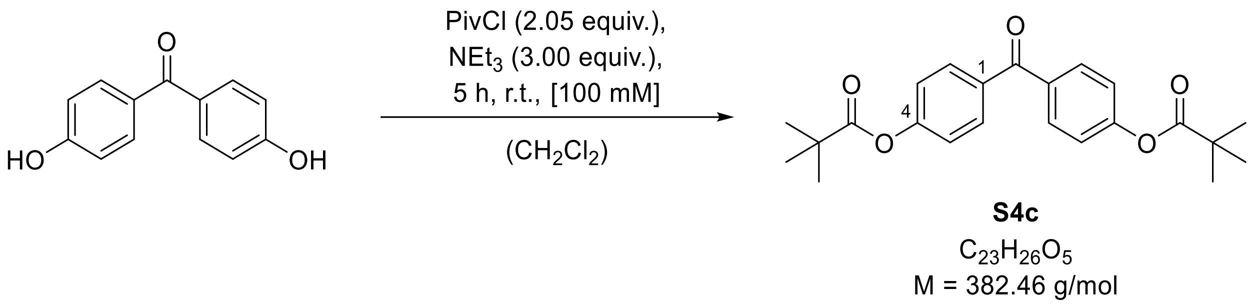

For the double pivaloyl protection, 2.00 g of bis(4-hydroxyphenyl)methanone (9.34 mmol, 1.00 equiv.) were suspended in 90 mL CH_2Cl_2 and cooled to 0 °C. 2.35 mL PivCl (19.1 mmol, 2.05 equiv.) and 3.90 mL triethylamine (28.0 mmol, 3.00 equiv.) were added dropwise to the cooled suspension. After complete addition the reaction mixture became a cloudy solution. After 5 h and complete conversion of the ketone, the reaction was diluted with 1N aq. NaOH solution until the cloudy precipitate was dissolved completely. Layers were separated and the aqueous phase was extracted with CH_2Cl_2 (3 × 50 mL). The combined organic layers were dried with brine and Na_2SO_4 and filtered. Any volatiles were removed under reduced pressure and the crude product was purified by flash column chromatography. This yielded 3.22 g of **S4c** (8.42 mmol, 90%) as a white solid.

Analytical data match those reported in literature.[7]

TLC: 0.75 (Hex:EtOAc = 8:2) [UV], [$KMnO_4$]

M.p.: 171 °C

^{1}H-NMR (500 MHz, DMSO-*d*$_6$, 300 K) δ [ppm] = 7.83 – 7.79 (m, 4H, CH-2) 7.33 – 7.29 (m, 4H, CH-3), 1.33 (s, 18H, CH_3).

^{13}C-NMR (125 MHz, DMSO-*d*$_6$, 300 K) δ [ppm] = 193.8 (CO), 176.1 (2C, *C*OO^{t}Bu), 154.1 (2C, C4), 134.5 (2C, C1), 131.4 (4C, C2), 122.1 (4C, C3), 38.7 [2C, *C*(CH_3)$_3$], 26.7 [6C, C(*C*H_3)$_3$].

Carbonylbis(4,1-phenylene) bis(4-methylbenzenesulfonate) (S4d)

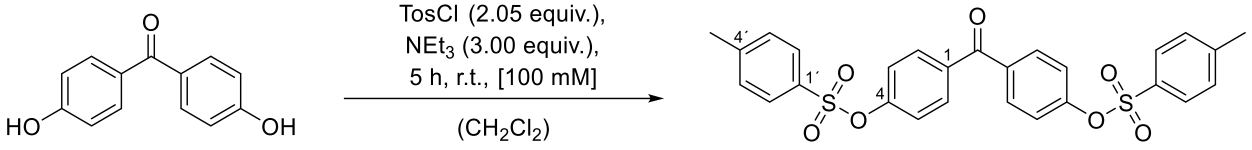

S4d
$C_{27}H_{22}O_7S_2$
M = 522.59 g/mol

For the double tosyl protection 2.00 g of bis(4-hydroxyphenyl)methanone (9.34 mmol, 1.00 equiv.) were suspended in 90 mL CH_2Cl_2 and cooled to 0 °C. 3.65 g TosCl (19.1 mmol, 2.05 equiv.) and 3.90 mL triethylamine (28.0 mmol, 3.00 equiv.) were added dropwise to the cooled suspension. After complete addition the reaction mixture became a cloudy solution. After 5 h and complete conversion of the ketone, the reaction was diluted with 1N aq. NaOH solution until the cloudy precipitate was fully dissolved. Layers were separated and the aqueous phase was extracted with CH_2Cl_2 (3 × 50 mL). The combined organic layers were dried with brine and Na_2SO_4 and filtered. Any volatiles were removed under reduced pressure and the crude product was purified by flash column chromatography. This yielded 4.05 g of **S4d** (7.75 mmol, 83%) as an off white solid.

TLC: 0.25 (Hex:EtOAc = 8:2) [UV], [$KMnO_4$]

M.p.: 175 °C

^{1}H-NMR (500 MHz, DMSO-d_6, 300 K) δ [ppm] = 7.82 – 7.76 (m, 4H, CH-2´) 7.75 – 7.71 (m, 4H, CH-2), 7.51 – 7.46 (m, 4H, CH-3´), 7.24 – 7.20 (m, 4H, CH-3) 2.42 (s, 6H, CH_3).

^{13}C-NMR (125 MHz, DMSO-d_6, 300 K) δ [ppm] = 193.4 (CO), 152.0 (2C, C4), 146.2 (2C, C4´), 135.5 (2C, C1´), 131.8 (4C, C2), 131.2 (2C, C1), 130.4 (4C, C2´), 128.3 (4C, C3´), 122.3 (4C, C3), 21.2 (2C, CH_3).

HRMS (ESI+) *m/z*: $[M+H]^+$ Calculated: 523.0880; Found: 523.0870

IR (ATR): $\tilde{\nu}$ [cm^{-1}] = 3444 (w, C-H_{arom}), 3313 (w, C-$H_{arom.}$), 2982 (w, C-$H_{aliph.}$), 1664 (s, C=O), 1595 (s), 1706 (s, C=O), 1362 (s, SO_2), 1175 (s, SO_2), 1090 (s), 858 (s), 722 (s).

4,4'-(Ethene-1,1-diyl)bis(chlorobenzene) (9a)

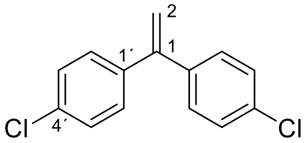

9a
$C_{14}H_{10}Cl_2$
M = 249.13 g/mol

9a was synthesized according to **GP-C** from commercially available bis(4-chlorophenyl)methanone (5.00 g, 19.9 mmol, 1.00 equiv.). After purification 4.71 g of **9a** (18.9 mmol, 95%) were obtained as a white solid.

Analytical data match those reported in literature.[8]

TLC: 0.68 (Hex) [UV], [$KMnO_4$]

M.p.: 83 °C

^{1}H-NMR (500 MHz, DMSO-d_6, 300 K) δ [ppm] = 7.46 – 7.42 (m, 4H, CH-3´) 7.33 – 7.28 (m, 4H, CH-2´), 5.55 (s, 2H, CH_2).

^{13}C-NMR (125 MHz, DMSO-d_6, 300 K) δ [ppm] = 146.8 (C1), 139.2 (2C, C4`), 132.8 (2C, C1´), 129.7 (4C, C2´), 128.5 (4C, C3´), 116.2 (C2).

4,4'-(Ethene-1,1-diyl)bis(methylbenzene) (9b)

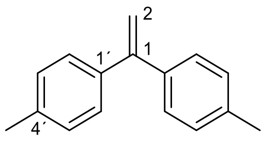

9b
$C_{16}H_{16}$
M = 208.30 g/mol

9b was synthesized according to **GP-C** from commercially available di-*p*-tolylmethanone (5.00 g, 23.8 mmol, 1.00 equiv.). After purification 4.91 g of **9b** (23.6 mmol, 99%) were obtained as a white solid.

Analytical data match those reported in literature.[9]

TLC: 0.53 (Hex) [UV], [$KMnO_4$]

M.p.: 75 °C

^{1}H-NMR (500 MHz, $CDCl_3$, 300 K) δ [ppm] = 7.27 – 7.23 (m, 4H, CH-2´) 7.17 – 7.13 (m, 4H, CH-3´), 5.39 (s, 2H, CH_2), 2.38 (s, 6H, CH_3).

^{13}C-NMR (125 MHz, $CDCl_3$, 300 K) δ [ppm] = 149.9 (C1), 138.9 (2C, C1`), 137.6 (2C, C4´), 129.0 (4C, C2´), 128.3 (4C, C3´), 113.2 (C2), 21.3 (2C, CH_3).

4,4'-(Ethene-1,1-diyl)bis(*tert*-butylbenzene) (9c)

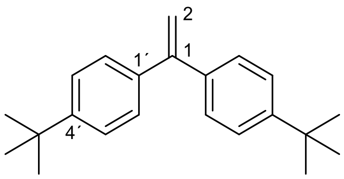

9c

$C_{22}H_{28}$

M = 292.47 g/mol

9c was synthesized according to **GP-C** from **S4a** (4.00 g, 13.6 mmol, 1.00 equiv.). After purification 3.94 g of **9c** (13.5 mmol, 99%) were obtained as a white solid.

Analytical data match those reported in literature.[10]

TLC: 0.50 (Hex) [UV], [$KMnO_4$]

M.p.: 103 °C

^{1}H-NMR (500 MHz, $CDCl_3$, 300 K) δ [ppm] = 7.37 – 7.33 (m, 4H, CH-2´) 7.31 – 7.28 (m, 4H, CH-3´), 5.41 (s, 2H, CH_2), 1.84 (s, 18H, CH_3).

^{13}C-NMR (125 MHz, $CDCl_3$, 300 K) δ [ppm] = 150.8 (2C, C4´), 149.7 (C1), 138.8 (2C, C1`), 128.1 (4C, C2´), 125.1 (4C, C3´), 113.4 (C2), 34.7 [2C, *C*(CH_3)$_3$], 31.5 [6C, C(*C*H_3)$_3$].

5,5'-(Ethene-1,1-diyl)bis(1,3-di-*tert*-butylbenzene) (9d)

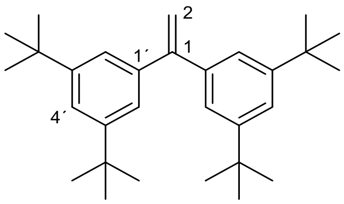

9d
$C_{30}H_{44}$
M = 404.68 g/mol

9d was synthesized according to **GP-C** from **S4b** (2.00 g, 4.92 mmol, 1.00 equiv.). After purification 1.82 g of **9d** (4.50 mmol, 91%) were obtained as colorless crystals.

Analytical data match those reported in literature.[11]

TLC: 0.50 (Hex) [UV], [$KMnO_4$]

M.p.: 92 °C

^{1}H-NMR (500 MHz, C_6D_6, 300 K) δ [ppm] = 7.59 – 7.54 (m, 6H, CH-2´/CH-4´) 5.68 (s, 2H, CH_2), 1.28 (s, 36H, CH_3).

^{13}C-NMR (125 MHz, C_6D_6, 300 K) δ [ppm] = 152.5 (C1), 150.7 (4C, C3´), 141.6 (2C, C1`), 123.5 (2C, C4´), 122.0 (4C, C2´), 113.3 (C2), 35.0 [4C, *C*(CH_3)$_3$], 31.7 [12C, C(*C*H_3)$_3$].

4,4'-(Ethene-1,1-diyl)bis(fluorobenzene) (9f)

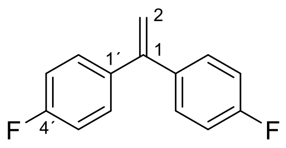

9f

$C_{14}H_{10}F_2$

M = 216.23 g/mol

9f was synthesized according to **GP-C** from commercially available bis(4-fluorophenyl)methanone (5.00 g, 22.9 mmol, 1.00 equiv.). After purification 4.56 g of **9f** (21.1 mmol, 92%) were obtained as a white solid.

Analytical data match those reported in literature.[12]

TLC: 0.61 (Hex) [UV], [$KMnO_4$]

M.p.: 54 °C

^{1}H-NMR (500 MHz, $CDCl_3$, 300 K) δ [ppm] = 7.31 – 7.25 (m, 4H, CH-3´) 7.05 – 7.00 (m, 4H, CH-2´), 5.39 (s, 2H, CH_2).

^{13}C-NMR (125 MHz, $CDCl_3$, 300 K) δ [ppm] = 162.7 (d, $^1J_{CF}$ = 247.0 Hz, 2C, C4´), 148.2 (C1), 137.5 (d, $^4J_{CF}$ = 3.0 Hz, 2C, C1´), 130.0 (d, $^3J_{CF}$ = 8.0 Hz, 4C, C2´), 115.3 (d, $^2J_{CF}$ = 21.6 Hz, 4C, C3´), 114.3 (C2).

^{19}F-NMR (470 MHz, $CDCl_3$, 300 K) δ [ppm] = –114.4

4,4'-(Ethene-1,1-diyl)bis(bromobenzene) (9g)

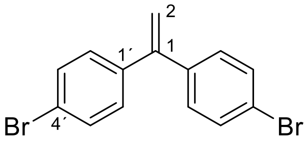

9g
$C_{14}H_{10}Br_2$
M = 338.04 g/mol

9g was synthesized according to **GP-C** from commercially available bis(4-bromophenyl)methanone (5.00 g, 14.7 mmol, 1.00 equiv.). After purification 4.48 g of **9g** (13.3 mmol, 90%) were obtained as a white solid.

Analytical data matched those reported in literature.[13]

TLC: 0.64 (Hex) [UV], [$KMnO_4$]

M.p.: 87 °C

^{1}H-NMR (500 MHz, DMSO-d_6, 300 K) δ [ppm] = 7.59 – 7.54 (m, 4H, CH-3´) 7.25 – 7.21 (m, 4H, CH-2´), 5.55 (s, 2H, CH_2).

^{13}C-NMR (125 MHz, DMSO-d_6, 300 K) δ [ppm] = 146.9 (C1), 139.5 (2C, C4´), 131.4 (4C, C3´), 130.0 (4C, C2´), 121.4 (2C, C1´), 116.2 (C2).

5,5'-(Ethene-1,1-diyl)bis(1,3-bis(trifluoromethyl)benzene) (9h)

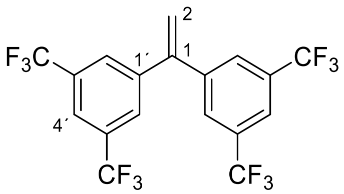

9h

$C_{18}H_8F_{12}$

M = 452.24 g/mol

9h was synthesized according to **GP-C** from commercially available bis(3,5-bis(trifluoromethyl)phenyl)methanone (5.00 g, 11.0 mmol, 1.00 equiv.). After purification 2.88 g of **9h** (6.37 mmol, 58%) were obtained as white crystals.

Analytical data matched those reported in literature.[14]

TLC: 0.70 (Hex) [UV], [$KMnO_4$]

M.p.: 88 °C

^{1}H-NMR (500 MHz, C_6D_6, 300 K) δ [ppm] = 7.66 (s, 2H, CH-4´) 7.40 (s, 4H, CH-2´), 4.89 (s, 2H, CH_2).

^{13}C-NMR (125 MHz, C_6D_6, 300 K) δ [ppm] = 144.5 (C1), 141.9 (2C, C1´), 132.3 (q, $^2J_{CF}$ = 33.6 Hz, 4C, C3´), 128.6 (4C, C2´), 123.6 (q, $^1J_{CF}$ = 273 Hz, 4C, CF_3), 122.3 (h, $^3J_{CF}$ = 4.0 Hz, 2C, C4´), 120.5 (C2).

^{19}F-NMR (470 MHz, C_6D_6, 300 K) δ [ppm] = –62.9.

3,3'-(Ethene-1,1-diyl)bis(nitrobenzene) (9i)

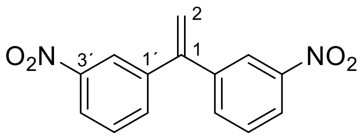

9i
$C_{14}H_{10}N_2O_4$
M = 270.24 g/mol

9i was synthesized according to **GP-C** from commercially available bis(3-nitrophenyl)methanone (5.00 g, 18.4 mmol, 1.00 equiv.). After purification 3.12 g of **9i** (11.6 mmol, 63%) were obtained as an off-white solid.

Analytical data matched those reported in literature.[15]

TLC: 0.85 (CH_2Cl_2:Pen = 7:3) [UV], [$KMnO_4$]

M.p.: 96 °C

^{1}H-NMR (500 MHz, $CDCl_3$, 300 K) δ [ppm] = 8.25 – 8.21 (m, 2H, CH-4´) 8.21 – 8.17 (m, 2H, CH-2´), 7.63 (*virt.* dt ,^3J = 7.7 Hz, $^4J \approx {}^4J$ = 1.4 Hz, 2H, CH-6´), 5.73 (s, 2H, CH_2).

^{13}C-NMR (125 MHz, $CDCl_3$, 300 K) δ [ppm] = 148.6 (C1), 146.3 (2C, C3´), 142.0 (2C, C4´), 134.1 (2C, C6´), 129.8 (2C, C5´), 123.4 (2C, C1´), 123.0 (2C, C2´), 119.0 (C2).

Ethene-1,1-diylbis(4,1-phenylene) bis(4-methylbenzenesulfonate) (9j)

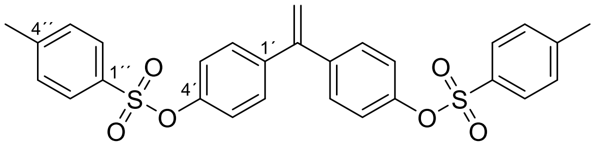

9j
$C_{28}H_{24}O_6S_2$
M = 520.61 g/mol

9j was synthesized according to **GP-C** from **S4d** (4.00 g, 7.65 mmol, 1.00 equiv.). After purification 2.88 g of **9j** (5.53 mmol, 72%) were obtained as a white solid.

TLC: 0.65 (CH_2Cl_2:Pen = 7:3) [UV], [$KMnO_4$]

M.p.: 162 °C

^{1}H-NMR (500 MHz, DMSO-d_6, 300 K) δ [ppm] = 7.78 – 7.74 (m, 4H, CH-2´´), 7.49 – 7.45 (m, 4H, CH-3´´), 7.26 – 7.22 (m, 4H, CH-2´), 7.04 – 7.00 (m, 4H, CH-3´), 5.50 (s, 2H, CH_2), 2.41 (s, 6H, CH_3).

^{13}C-NMR (125 MHz, DMSO-d_6, 300 K) δ [ppm] = 149.1 (2C, C4´), 146.3 (C1), 146.4 (2C, C4´´), 139.8 (2C, C1´), 131.9 (2C, C1´´), 130.8 (4C, C3´´), 129.8 (4C, C2´), 128.7 (4C, C2´´), 122.6 (4C, C3´), 117.2 (C2), 21.6 (2C, CH_3).

HRMS (ESI+) *m/z*: $[M+H]^+$ Calculated: 521.1087; Found: 521.1070

IR (ATR): $\tilde{\nu}$ [cm^{-1}] = 3093 (w, C-H_{arom}), 3041 (w, C-$H_{arom.}$), 2962 (w, C-$H_{aliph.}$), 1597 (m), 1499 (s), 1370 (s, SO_2), 1196 (s, SO_2), 1151 (s), 813 (s), 717 (s).

Ethene-1,1-diylbis(4,1-phenylene) bis(2,2-dimethylpropanoate) (9k)

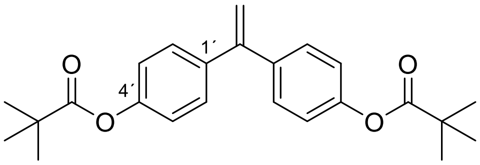

9k
$C_{24}H_{28}O_4$
M = 380.48 g/mol

9k was synthesized according to **GP-C** from **S4c** (3.00 g, 7.84 mmol, 1.00 equiv.). After purification 1.05 g of **9k** (2.76 mmol, 35%) were obtained as a white solid.

TLC: 0.85 (CH_2Cl_2:Pen = 7:3) [UV], [$KMnO_4$]

M.p.: 153 °C

^{1}H-NMR (500 MHz, $CDCl_3$, 300 K) δ [ppm] = 7.36 – 7.32 (m, 4H, CH-2´), 7.04 – 7.01 (m, 4H, CH-3´), 5.43 (s, 2H, CH_2), 1.37 (s, 18H, CH_3).

^{13}C-NMR (125 MHz, $CDCl_3$, 300 K) δ [ppm] = 177.3 (2C, CO), 150.9 (2C, C4´), 148.5 (C1), 138.9 (2C, C1´), 129.4 (4C, C2´), 121.4 (4C, C3´), 114.7 (C2), 39.2 [2C, *C*(CH_3)$_3$], 27.3 [6C, C(*C*H_3)$_3$].

HRMS (ESI+) *m/z*: $[M+H]^+$ Calculated: 381.2060; Found: 381.2069

IR (ATR): $\tilde{\nu}$ [cm^{-1}] = 3481 (w, C-H_{arom}), 3041 (w, C-$H_{arom.}$), 2972 (m, CO.), 2907 (w), 1747 (s, C=O), 1504 (m), 1479 (m), 1275 (m),1196 (m), 1112 (s, C-O-C), 895 (s), 757 (m).

1-Acetylindoline-2,3-dione (S5a)

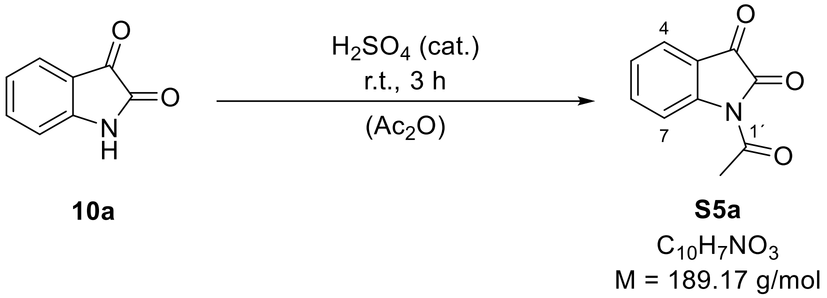

For *N*-acetylation 5.00 g isatin (34.0 mmol, 1.00 equiv.) were suspended in 10 mL acetic anhydride and two drops of conc. H_2SO_4 and were stirred at r.t. for 3 h. The bright yellow suspension is poured on ice and extracted with EtOAc (3 × 100ml). The combined organic layers were washed with sat. aq. $NaHCO_3$ solution, dried over brine and Na_2SO_4 and filtered. Excess solvent was removed under reduced pressure and the crude product was recrystallized from EtOAc:heptane = 1:9. 5.81 g of 1-acetylindoline-2,3-dione (30.7 mmol) were obtained as a bright yellow needles (90%).

Analytical data match those reported in literature.[1]

TLC: 0.75 (Hex:EtOAc = 6:4) [UV], [$KMnO_4$]

M.p.: 153 °C

^{1}H-NMR (500 MHz, $CDCl_3$, 300 K) δ [ppm] = 8.40 (d, ^3J = 7.5 Hz, 1H, CH-7), 7.77 (d, ^3J = 7.5 Hz, 1H, CH-4), 7.71 (*virt.* td, $^3J \approx {}^3J$ = 7.5 Hz, ^4J = 1.5 Hz, 1H, CH-6), 7,33 (*virt.* td, $^3J \approx {}^3J$ = 7.5 Hz, ^4J = 1.5 Hz, 1H, CH-6), 2.72 (s, 3H, CH_3).

^{13}C-NMR (125 MHz, $CDCl_3$, 300 K) δ [ppm] = 180.2 (C3), 169.9 (C1′), 158.1 (C2), 148.7 (C7a), 139.1 (C6), 126.3 (C5), 125.4 (C4), 119.3 (C3a), 118.4 (C7), 26.6 (CH_3).

IR (ATR): $\tilde{\nu}$ [cm^{-1}] = 3555, (w, C-H_{arom}), 3488 (w, C-$H_{arom.}$), 2940 (w, C-$H_{aliph.}$), 1781 (s, C=O), 1746 (s, C=O), 1706 (s, C=O), 1607 (m, C=C), 1459 (m), 1306 (m), 1152 (m), 979 (m), 764 (s).

1-Acetyl-5-fluoroindoline-2,3-dione (S5b)

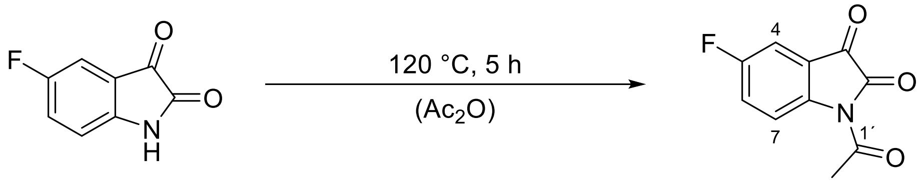

10b

S5b
$C_{10}H_6FNO_3$
M = 207.16 g/mol

For *N*-acetylation 5-fluoroisatin (1.00 g, 6.06 mmol, 1.00 equiv.) was suspended in 5 mL acetic anhydride and heated to 120 °C for 5 h. The clear, dark red solution is poured on ice and the precipitated solid extracted with EtOAc (3 × 100ml). The combined organic layers were washed with sat. aq. $NaHCO_3$ solution, dried over brine and Na_2SO_4 and filtered. Excess solvent was removed under reduced pressure and the crude product was purified by column chromatography (Hex:EtOAc = 8:2) and subsequently recrystallized (EtOAc:heptane = 0.5:9.5). 1.02 g of **S5b** (4.92 mmol) were obtained as a yellow solid (81%).

Analytical data match those reported in literature.[3]

TLC: 0.40 (Hex:EtOAc = 6:4) [UV], [$KMnO_4$]

M.p.: 150 °C

^{1}H-NMR (400 MHz, $CDCl_3$, 300 K) δ [ppm] = 8.48 – 8.40 (m, 1H, CH-7), 7.48 – 7.39 (m, 2H, CH-4 / CH-6), 2.71 (s, 3H, CH_3).

^{13}C-NMR (100 MHz, $CDCl_3$, 300 K) δ [ppm] = 179.7 (d, $^4J_{CF}$ = 2.5 Hz, C3), 169.6 (C1´), 160.3 (d, $^1J_{CF}$ = 250 Hz, C5), 157.6 (C2), 145.0 (d, $^4J_{CF}$ = 2.4 Hz, C7a), 125.7 (d, $^2J_{CF}$ = 23.4 Hz, C6), 120.4 (d, $^3J_{CF}$ = 7.8 Hz, C3a), 120.4 (d, $^3J_{CF}$ = 6.9 Hz, C7), 111.7 (d, $^2J_{CF}$ = 24.3 Hz, C3), 26.6 (CH_3).

^{19}F-NMR (376 MHz, CDCl3, 300 K) δ [ppm] = −113.8.

IR (ATR): $\tilde{\nu}$ [cm^{-1}] = 3549, (w, C-H$_{arom}$), 3404 (w, C-H$_{arom.}$), 3148 (m), 2947 (w, C-H$_{aliph.}$), 1779 (s, C=O), 1751 (s, C=O), 1703 (s, C=O), 1474 (s, C=C), 1297 (m), 1146 (m), 1038 (m), 893 (m), 809 (s).

1**-Acetyl-6-chloroindoline-2,3-dione (S5c)**

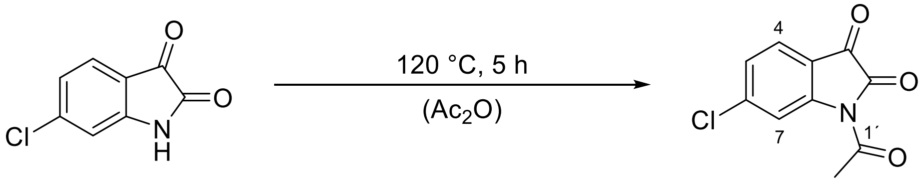

10c

S5c
$C_{10}H_6ClNO_3$
M = 223.61 g/mol

For *N*-acetylation 6-cloroisatin (1.00 g, 5.51 mmol, 1.00 equiv.) was suspended in 5 mL acetic anhydride and heated to 120 °C for 5 h. The clear, dark orange solution is poured on ice and the precipitated solid extracted with EtOAc (3 × 100ml). The combined organic layers were washed with sat. aq. $NaHCO_3$ solution, dried over brine and Na_2SO_4 and filtered. Excess solvent was removed under reduced pressure and the crude product was purified by column chromatography (Hex:EtOAc = 7:3) and subsequently recrystallized (EtOAc:heptane = 1:9). 0.92 g of **S5c** (4.11 mmol) were obtained as a yellow solid (74%).

Analytical data match those reported in literature.[2]

TLC: 0.32 (Hex:EtOAc = 6:4) [UV], [$KMnO_4$]

M.p.: 170 °C

^{1}H-NMR (500 MHz, $CDCl_3$, 300 K) δ [ppm] = 8.49 (d, ^{4}J = 1.8 Hz, 1H, CH-7), 7.73 (d, ^{3}J = 8.1 Hz, 1H, CH-4), 7.33 (dd, ^{3}J = 8.1 Hz, ^{4}J = 1.8 Hz, 1H, CH-5), 2.74 (s, 3H, CH_3).

^{13}C-NMR (125 MHz, $CDCl_3$, 300 K) δ [ppm] = 178.9 (C3), 169.2 (C1´), 157.8 (C2), 149.2 (C6), 145.7 (C7a), 126.9 (C5), 126.4 (C4), 119.1 (C7), 117.6 (C3a), 26.6 (CH_3).

IR (ATR): $\tilde{\nu}$ [cm^{-1}] = 3539, (w, C-H_{arom}), 3416 (w, C-$H_{arom.}$), 3139 (m), 2941 (w, C-$H_{aliph.}$), 1788 (m, C=O), 1745 (s, C=O), 1711 (s, C=O), 1576 (m, C=C), 1325 (m), 1156 (m), 1099 (m), 886 (m), 708 (s).

3',3'-Bis(4-chlorophenyl)spiro[indoline-3,2'-oxetan]-2-one (*rac*-5a)

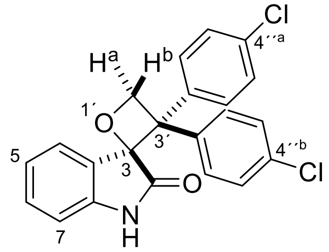

rac-**5a**
$C_{22}H_{15}Cl_2NO_2$
M = 396.27 g/mol

Substrate *rac*-**5a** was synthesized according to **GP-D** from **S5a** (300 mg, 1.59 mmol, 1.00 equiv.) and 1.19 g of **9a** (4.76 mmol, 3.00 equiv.). After deprotection and purification 515 mg of *rac*-**5a** (1.30 mmol, 82%) were obtained as a white amorphous solid.

TLC: 0.48 (CH_2Cl_2:Ac = 97.5:2.5) [UV], [$KMnO_4$]

M.p.: decomp. under normal conditions

^{1}H-NMR (500 MHz, DMSO-d_6, 300 K) δ [ppm] = 10.59 (s, 1H, NH), 7.43 – 7.35 (m, 4H, CH-3´´^{b}/CH-3´´^{a}), 7.34 – 7.29 (m, 2H, CH-2´´^{a}), 7.23 (*virt.* td, $^{3}J \approx {}^{3}J$ = 7.7 Hz, ^{4}J = 1.3 Hz, 1H, CH-6), 6.99 – 6.94 (m, 2H, CH-2´´^{b}), 6.85 (d, ^{3}J = 7.7 Hz, CH-7), 6.67 (*virt.* td, $^{3}J \approx {}^{3}J$ = 7.7 Hz, ^{4}J = 1.3 Hz, 1H, CH-5), 6.15 (d, ^{3}J = 7.7 Hz, 1H, CH-4), 5.66 (d, ^{2}J = 6.1 Hz, 1H, CH^{b}), 5.43 (d, ^{2}J = 6.1 Hz, 1H, CH^{a}).

^{13}C-NMR (125 MHz, DMSO-d_6, 300 K) δ [ppm] = 176.9 (CO), 142.6 (C7a), 141.9 (C4´´^{a}), 138.6 (C4´´^{b}), 132.1 (C1´´^{b}), 131.3 (C6), 130.1 (2C, C2´´^{b}), 128.4 (2C, C3´´^{a}), 128.3 (2C, C3´´^{b}), 128.0 (2C, C2´´^{a}), 126.8 (C4), 124.7 (C3a), 121.3 (C5), 110.3 (C7), 90.7 (C3), 76.3 (C2´), 59.9 (C3´).

HRMS (ESI+) *m/z*: $[M+H]^+$ Calculated: 396.0553; Found: 396.0562

IR (ATR): $\tilde{\nu}$ [cm^{-1}] = 3416 (w, C-H_{arom}), 3241 (w, C-$H_{arom.}$), 2954 (w, C-$H_{alip.}$), 2856 (w), 1720 (s, C=O), 1612 (m), 1466 (m), 1325 (m), 1093 (m, C-O-C), 949 (m), 929 (m), 754 (s).

Chiral HPLC: AD-H 250 × 4.6 mm, *n*-Hep/^{i}PrOH = 90/10, 1 mL/min, λ = 210 nm, t_R = 17.7 min (**5a**), 45.8 (*ent*-**5a**).

3',3'-Di-*p*-tolylspiro[indoline-3,2'-oxetan]-2-one (*rac*-5b)

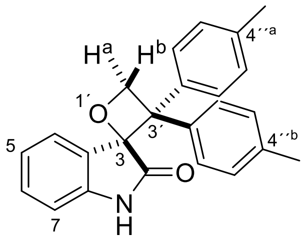

rac-**5b**
$C_{24}H_{21}NO_2$
M = 355.44 g/mol

Substrate *rac*-**5b** was synthesized according to **GP-D** from **S5a** (300 mg, 1.59 mmol, 1.00 equiv.) and 991 mg of **9b** (4.76 mmol, 3.00 equiv.). After deprotection and purification 412 mg of *rac*-**5b** (1.16 mmol, 73%) were obtained as a white amorphous solid.

TLC: 0.35 (CH_2Cl_2:Ac = 97.5:2.5) [UV], [$KMnO_4$]

M.p.: decomp. under normal conditions

^{1}H-NMR (500 MHz, DMSO-d_6, 300 K) δ [ppm] = 10.50 (s, 1H, NH), 7.21 – 7.16 (m, 3H, CH-6/CH-2´´^{a}), 7.14 – 7.10 (m, 2H, CH-3´´^{a}), 7.10 – 7.07 (m, 2H, CH-3´´^{b}), 6.82 (d, ^3J = 7.6 Hz, CH-7), 6.79 – 6.75 (m, 2H, CH-2´´^{b}), 6.59 (*virt.* t, $^3J \approx {}^3J$ = 7.6 Hz, 1H, CH-5), 6.09 (d, ^3J = 7.6 Hz, 1H, CH-4), 5.65 (d, ^2J = 5.6 Hz, 1H, CH^b), 5.39 (d, ^2J = 5.6 Hz, 1H, CH^a), 2.26 (s, 3H, CH_3^a), 2.24 (s, 3H, CH_3^b).

^{13}C-NMR (125 MHz, DMSO-d_6, 300 K) δ [ppm] = 177.5 (CO), 142.6 (C7a), 140.7 (C4´´^{b}), 137.3 (C4´´^{a}), 136.1 (C1´´^{a}), 135.3 (C1´´^{b}), 131.0 (C6), 128.8 (2C, C3´´^{b}), 128.8 (2C, C3´´^{a}), 128.3 (2C, C2´´^{a}), 126.9 (C4), 125.9 (2C, C2´´^{b}), 125.2 (C3a), 121.1 (C5), 110.0 (C7), 90.9 (C3), 76.6 (C2´), 60.2 (C3´), 20.6 (CH_3^b), 20.6 (CH_3^a).

HRMS (ESI+) *m/z*: $[M+H]^+$ Calculated: 356.1645; Found: 356.1648

IR (ATR): $\tilde{\nu}$ [cm^{-1}] = 3348 (m, C-H_{arom}), 3058 (w, C-$H_{arom.}$), 2979 (m, CH_2), 2911 (w), 1732 (s, C=O), 1702 (s), 1468 (m), 1405 (m), 1092 (s, C-O-C), 1012 (s), 801 (m), 751 (s).

Chiral HPLC: OD-RH 250 × 4.6 mm, H_2O/MeCN = 80/20 → 0/100, 1 mL/min, λ = 210 nm, t_R = 20.9 min (**5b**), 21.9 (*ent*-**5b**).

3',3'-Bis(4-(*tert*-butyl)phenyl)spiro[indoline-3,2'-oxetan]-2-one (*rac*-5c)

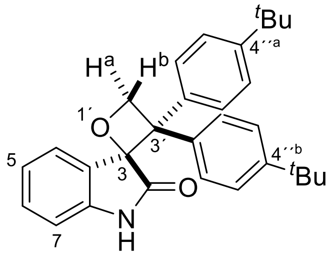

rac-**5c**
$C_{30}H_{33}NO_2$
M = 439.60 g/mol

Substrate *rac*-**5c** was synthesized according to **GP-D** from **S5a** (300 mg, 1.59 mmol, 1.00 equiv.) and 1.39 g of **9c** (4.76 mmol, 3.00 equiv.). After deprotection and purification 495 mg of *rac*-**5c** (1.13 mmol, 71%) were obtained as a white amorphous solid.

TLC: 0.45 (CH_2Cl_2:Ac = 97.5:2.5) [UV], [$KMnO_4$]

M.p.: decomp. under normal conditions

^{1}H-NMR (500 MHz, DMSO-d_6, 300 K) δ [ppm] = 10.53 (s, 1H, NH), 7.35 – 7.28 (m, 4H, CH-3´´^{a}/CH-3´´^{b}), 7.24 – 7.19 (m, 2H, CH-2´´^{a}), 7.18 (*virt.* td, $^3J \approx {}^3J$ = 7.6 Hz, ^4J = 1.2 Hz, 1H, CH-6), 6.84 – 6.79 (m, 3H, CH-7/CH-2´´^{b}), 6.51 (*virt.* td, $^3J \approx {}^3J$ = 7.6 Hz, ^4J = 1.2 Hz, 1H, CH-5), 5.91 (d, ^3J = 7.6 Hz, 1H, CH-4), 5.65 (d, ^2J = 5.6 Hz, 1H, CH^b), 5.44 (d, ^2J = 5.6 Hz, 1H, CH^a), 1.25 (s, 9H, CH_3^a), 1.24 (s, 9H, CH_3^b).

^{13}C-NMR (125 MHz, DMSO-d_6, 300 K) δ [ppm] = 177.5 (CO), 149.6 (C4´´^{a}), 148.3 (C4´´^{b}), 142.5 (C7a), 140.6 (C1´´^{b}), 137.0 (C1´´^{a}), 131.0 (C6), 128.0 (2C, C2´´^{a}), 125.6 (2C, C2´´^{b}), 125.2 (C3a), 125.0 (2C, C3´´^{a}), 124.9 (2C, C3´´^{b}), 120.9 (C5), 109.9 (C7), 91.1 (C3), 76.2 (C2´), 60.2 (C3´), 34.2 [$C(CH_3)_3^b$], 34.2 [$C(CH_3)_3^a$], 31.2 [$C(CH_3)_3^a$], 31.1 [$C(CH_3)_3^b$].

HRMS (ESI+) *m/z*: $[M+H]^+$ Calculated: 440.2584; Found: 440.2584

IR (ATR): $\tilde{\nu}$ [cm^{-1}] = 3250 (br, NH), 3094 (w, C-H_{arom}), 3057 (w, C-$H_{arom.}$), 2961 (m, $CH_{2.}$), 2903 (w), 1724 (s, C=O), 1620 (s), 1466 (m), 1201 (m), 1109 (m, C-O-C), 930 (m), 751 (s).

Chiral HPLC: AD-H 250 × 4.6 mm, *n*-Hep/^{i}PrOH = 90/10, 1 mL/min, λ = 210 nm, t_R = 5.8 min (**5c**), 24.2 (*ent*-**5c**).

3',3'-Bis(3,5-di-*tert*-butylphenyl)spiro[indoline-3,2'-oxetan]-2-one (*rac*-5d)

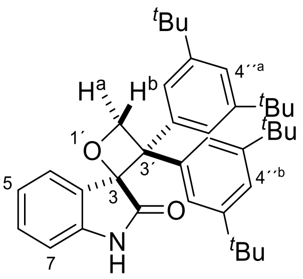

rac-**5d**
$C_{38}H_{49}NO_2$
M = 551.82 g/mol

Substrate *rac*-**5d** was synthesized according to **GP-D** from **S5a** (300 mg, 1.59 mmol, 1.00 equiv.) and 1.93 g of **9d** (4.76 mmol, 3.00 equiv.). After deprotection and purification 540 mg of *rac*-**5d** (0.98 mmol, 62%) were obtained as a white amorphous solid.

TLC: 0.51 (CH_2Cl_2:Ac = 97.5:2.5) [UV], [$KMnO_4$]

M.p.: decomp. under normal conditions

^{1}H-NMR (500 MHz, DMSO-d_6, 300 K) δ [ppm] = 10.49 (s, 1H, NH), 7.22 – 7.17 (m, 2H, CH-6/CH-4´´^{a}), 7.16 (t, ^4J = 1.8 Hz, 1H, CH-4´´^{b}), 7.07 (br, 2H, CH-2´´^{a}), 6.86 (d, ^3J = 7.6 Hz, 1H, CH-7), 6.81 (d, ^4J = 1.8 Hz, 2H, CH-2´´^{b}), 6.58 (*virt.* td, $^3J \approx {}^3J$ = 7.6 Hz, ^4J = 1.0 Hz, 1H, CH-5), 6.01 (dd, ^3J = 7.6 Hz, ^4J = 1.0 Hz, 1H, CH-4), 5.67 (d, ^2J = 6.0 Hz, 1H, CH^b), 5.56 (d, ^2J = 6.0 Hz, 1H, CH^a), 1.21 (s, 18H, $CH_3{}^b$), 1.14 (s, 18H, $CH_3{}^a$).

^{13}C-NMR (125 MHz, DMSO-d_6, 300 K) δ [ppm] = 177.7 (CO), 149.6 (2C, C3´´^{b}), 149.5 (2C, C3´´^{a}), 143.7 (C7a), 142.4 (C1´´^{b}), 139.7 (C1´´^{a}), 130.7 (C6), 127.2 (C4) 126.0 (C4´´^{a}), 122.3 (2C, C2´´^{a}), 122.1 (C3a) 121.0 (C5), 120.0 (2C, C2´´^{b}), 119.3 (C4´´b), 109.7 (C7), 92.1 (C3), 76.4 (C2´), 61.7 (C3´), 34.5 [2C, $C(CH_3)_3{}^a$ /$C(CH_3)_3{}^b$], 31.2 [$C(CH_3)_3{}^a$], 31.1 [$C(CH_3)_3{}^b$].

HRMS (ESI+) *m/z*: $[M+H]^+$ Calculated: 552.3836; Found: 552.3837

IR (ATR): $\tilde{\nu}$ [cm^{-1}] = 3206 (br, NH), 3138 (w, C-H_{arom}), 2962 (m, $CH_{2.}$), 2903 (w), 1732 (s, C=O), 1621 (s), 1597 (m), 1468 (m), 1248 (m), 1100 (m, C-O-C), 751 (s).

Chiral HPLC: OD-RH 250 × 4.6 mm, H_2O/MeCN = 80/20 → 0/100, 1 mL/min, λ = 210 nm, t_R = 23.9 min (**5d**), 24.6 (*ent*-**5d**).

3',3'-Diphenylspiro[indoline-3,2'-oxetan]-2-one (*rac*-5e)

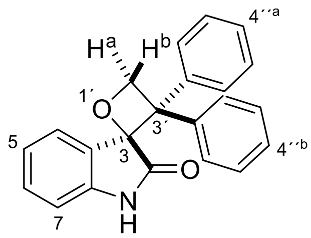

rac-**5e**
$C_{22}H_{17}NO_2$
M = 327.38 g/mol

Substrate *rac*-**5e** was synthesized according to **GP-D** from **S5a** (300 mg, 1.59 mmol, 1.00 equiv.) and 0.86 g of commercially available **9e** (4.76 mmol, 3.00 equiv.). After deprotection and purification 355 mg of *rac*-**5e** (1.08 mmol, 68%) were obtained as an off white amorphous solid.

TLC: 0.47 (CH_2Cl_2:Ac = 97.5:2.5) [UV], [$KMnO_4$]

M.p.: decomp. under normal conditions

^{1}H-NMR (500 MHz, DMSO-d_6, 300 K) δ [ppm] = 10.56 (s, 1H, NH), 7.36 – 7.26 (m, 7H, CH-2´´^{a}/CH-3´´^{a}/CH-3´´^{b}/CH-4´´^{a}), 7.22 – 7.17 (m, 2H, CH-4´´^{b}/CH-6), 6.94 – 6.90 (m, 2H, CH-2´´^{b}), 6.83 (d, ^3J = 7.3 Hz, 1H, CH-7), 6.57 (*virt.* td, $^3J \approx {}^3J$ = 7.6 Hz, ^4J = 1.1 Hz, 1H, CH-5), 6.00 (d, ^3J = 7.6 Hz, 1H, CH-4), 5.69 (d, ^2J = 5.9 Hz, 1H, CH^b), 5.48 (d, ^2J = 5.9 Hz, 1H, CH^a).

^{13}**C-NMR** (75 MHz, DMSO-d_6, 300 K) δ [ppm] = 177.4 (CO), 143.5 (C7a), 142.6 (C1´´^{a}), 139.9 (C1´´^{b}), 131.1 (C6), 128.4 (2C, C2´´^{a}), 128.3 (2C, C3´´^{a*}), 128.2 (2C, C3´´^{b*}), 127.3 (C4´´^{a**}), 126.8 (C4´´^{b**}), 126.4 (C4), 126.1 (2C, C2´´^{b}), 125.1 (C3a), 121.1 (C5), 110.0 (C7), 91.0 (C3), 76.4 (C2´), 60.8 (C3´).

Signals marked with * and ** could not be assigned and can be interchanged.

HRMS (ESI+) *m/z*: $[M+H]^+$ Calculated: 328.1332; Found: 328.1333

IR (ATR): $\tilde{\nu}$ [cm^{-1}] = 3348 (br, NH), 3058 (w, C-H$_{arom}$), 3032 (w, C-H$_{arom.}$), 2980 (w, $CH_{2.}$), 2909 (w), 1732 (s, C=O), 1702 (s), 1621 (m), 1468 (s), 1092 (m, C-O-C), 1012 (s), 801 (m), 752 (s).

Chiral HPLC: AD-H 250 × 4.6 mm, *n*-Hep/^{i}PrOH = 90/10, 1 mL/min, λ = 210 nm, t_R = 16.4 min (**5e**), 43.7 (*ent*-**5e**).

3',3'-Bis(4-fluorophenyl)spiro[indoline-3,2'-oxetan]-2-one (*rac*-5f)

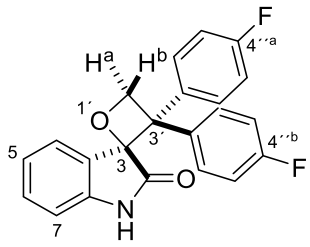

rac-**5f**
$C_{22}H_{15}F_2NO_2$
M = 363.36 g/mol

Substrate *rac*-**5f** was synthesized according to **GP-D** from **S5a** (300 mg, 1.59 mmol, 1.00 equiv.) and 1.03 g of **9f** (4.76 mmol, 3.00 equiv.). After deprotection and purification 509 mg of *rac*-**5f** (1.40 mmol, 88%) were obtained as a white amorphous solid.

TLC: 0.35 (CH_2Cl_2:Ac = 97.5:2.5) [UV], [$KMnO_4$]

M.p.: decomp. under normal conditions

^{1}H-NMR (300 MHz, DMSO-d_6, 300 K) δ [ppm] = 10.66 (s, 1H, NH), 7.42 – 7.33 (m, 2H, CH-2´´^{a*}/CH-2´´^{b**}), 7.24 (*virt.* td, $^3J \approx {}^3J$ = 7.7 Hz, ^4J = 1.4 Hz, 1H, CH-6), 7.18 – 7.03 (m, 4H, CH-3´´^{a***}/CH-3´´^{b****}/CH-5´´^{b****}/CH-6´´^{b**}), 6.87 (d, ^3J = 7.7 Hz, 1H, CH-7), 6.85–6.78 (m, 2H, CH-5´´^{a***}/CH-6´´^{a*}), 6.66 (*virt.* td, $^3J \approx {}^3J$ = 7.7 Hz, ^4J = 1.4 Hz, 1H, CH-5), 6.10 (dd, ^3J = 7.7 Hz, ^4J = 1.4 Hz 1H, CH-4), 5.65 (d, ^2J = 6.2 Hz, 1H, CH^b), 5.47 (d, ^2J = 6.2 Hz, 1H, CH^a).

Signals marked with *, **, *** and **** could not be assigned and can be interchanged.

^{13}C-NMR (125 MHz, DMSO-d_6, 300 K) δ [ppm] = 177.4 (CO), 162.5 (d, $^1J_{CF}$ = 244.0 Hz, C4´´^{a*}), 162.4 (d, $^1J_{CF}$ = 244.0 Hz, C4´´^{b*}), 146.0 (d, $^3J_{CF}$ = 7.1 Hz, C6´´^{a**}),143.1 (C7a), 142.8 (d, $^3J_{CF}$ = 7.1 Hz, C6´´^{b**}), 131.9 (C6), 130 (d, $^3J_{CF}$ = 8.0 Hz, C2´´^{a**}), 130.8 (d, $^3J_{CF}$ = 8.0 Hz, C2´´^{b**}), 127.2 (C4), 125.1 (C3a), 125.0 (d, $^4J_{CF}$ = 2.8 Hz, C1´´^{a***}), d, $^4J_{CF}$ = 2.8 Hz, C1´´^{b***}), 121.8 (C5), 115.6 (d, $^2J_{CF}$ = 22.3 Hz, C5´´^{a****}), 114.8 (d, $^2J_{CF}$ = 20.8 Hz, C5´´^{b****}), 114.1 (d, $^4J_{CF}$ = 20.8 Hz, C3´´^{b****}), 113.6 (d, $^2J_{CF}$ = 22.3 Hz, C3´´^{a****}) 110.8 (C7), 91.2 (C3), 76.7 (C2´), 60.8 (C3´).

Signals marked with *, **, *** and **** could not be assigned and can be interchanged.

^{19}F-NMR (376 MHz, DMSO-d_6, 300 K) δ [ppm] = –112.5.

HRMS (ESI+) *m/z*: [M+H]$^+$ Calculated: 364.1144; Found: 364.1147

IR (ATR): $\tilde{\nu}$ [cm^{-1}] = 3249 (br, NH), 2981 (w, $CH_{2.}$), 2903 (w), 1724 (s, C=O), 1617 (m), 1587 (m), 1488 (s), 1197 (m, C-O-C), 784 (w), 749 (s), 701 (m).

Chiral HPLC: AD-H 250 × 4.6 mm, *n*-Hep/^{i}PrOH = 70/30, 1 mL/min, λ = 210 nm, t_R = 7.0 min (**5f**), 9.3 (*ent*-**5f**).

3',3'-Bis(4-bromophenyl)spiro[indoline-3,2'-oxetan]-2-one (*rac*-5g)

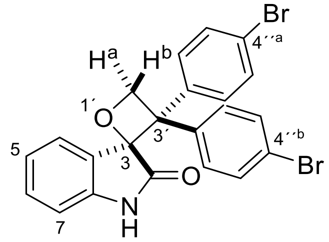

rac-**5g**
$C_{22}H_{15}Br_2NO_2$
M = 485.18 g/mol

Substrate *rac*-**5g** was synthesized according to **GP-D** from **S5a** (300 mg, 1.59 mmol, 1.00 equiv.) and 1.61 g of **9g** (4.76 mmol, 3.00 equiv.). After deprotection and purification 676 mg of *rac*-**5g** (1.39 mmol, 88%) were obtained as a yellowish amorphous solid.

TLC: 0.43 (CH_2Cl_2:Ac = 97.5:2.5) [UV], [$KMnO_4$]

M.p.: decomp. under normal conditions

^{1}H-NMR (500 MHz, DMSO-d_6, 300 K) δ [ppm] = 10.62 (s, 1H, NH), 7.56 – 7.52 (m, 2H, CH-3´´^{a}), 7.52 – 7.48 (m, 2H, CH-3´´^{b}), 7.28 – 7.20 (m, 3H, CH-6/CH-2´´^{a}), 6.91 – 6.88 (m, 2H, CH-2´´^{b}), 6.85 (d, ^3J = 7.7 Hz, CH-7), 6.67 (*virt.* td, $^3J \approx {}^3J$ = 7.7 Hz, ^4J = 1.1 Hz, 1H, CH-5), 6.15 (dd, ^3J = 7.7 Hz, ^4J = 1.1 Hz, 1H, CH-4), 5.63 (d, ^2J = 6.2 Hz, 1H, CH^b), 5.43 (d, ^2J = 6.2 Hz, 1H, CH^a).

^{13}C-NMR (125 MHz, DMSO-d_6, 300 K) δ [ppm] = 177.1 (CO), 142.7 (C4´´^{b}), 142.4 (C7a), 139.0 (C4´´^{a}), 131.5 (C6), 131.4 (2C, C3´´^{b}), 131.4 (2C, C3´´^{a}), 130.6 (2C, C2´´^{a}), 128.4 (2C, C2´´^{b}), 126.9 (C4), 124.8 (C3a), 121.5 (C5), 120.9 (C1´´^{a}), 119.9 (C1´´^{b}), 110.4 (C7), 90.7 (C3), 76.3 (C2´), 60.0 (C3´).

HRMS (ESI+) *m/z*: $[M+H]^+$ Calculated: 483.9542; Found: 483.9534

IR (ATR): $\tilde{\nu}$ [cm^{-1}] = 3248 (w, C-H_{arom}), 3094 (w, C-$H_{arom.}$), 3027 (w, C-$H_{arom.}$), 2958 (w, C-$H_{alip.}$), 2854 (w), 1722 (s, C=O), 1619 (m), 1487 (s), 1199 (m), 1007 (m, C-O-C), 927 (m), 751 (s).

Chiral HPLC: AD-H 250 × 4.6 mm, *n*-Hep/^{i}PrOH = 70/30, 1 mL/min, λ = 210 nm, t_R = 7.3 min (**5g**), 19.0 (*ent*-**5g**).

3',3'-Bis(3,5-bis(trifluoromethyl)phenyl)spiro[indoline-3,2'-oxetan]-2-one (*rac*-5h)

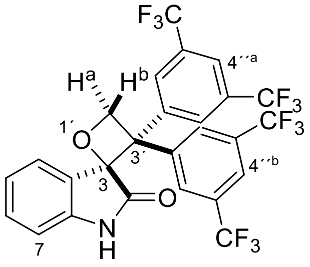

rac-**5h**
$C_{26}H_{13}F_{12}NO_2$
M = 599.38 g/mol

Substrate *rac*-**5h** was synthesized according to **GP-D** from **S5a** (300 mg, 1.59 mmol, 1.00 equiv.) and 2.15 g of **9h** (4.76 mmol, 3.00 equiv.). After deprotection and purification 879 mg of *rac*-**5h** (1.47 mmol, 92%) were obtained as an off white amorphous solid.

TLC: 0.59 (CH_2Cl_2:Ac = 97.5:2.5) [UV], [$KMnO_4$]

M.p.: decomp. under normal conditions

^{1}H-NMR (500 MHz, DMSO-d_6, 300 K) δ [ppm] = 10.89 (s, 1H, NH), 8-06 (s, 2H, CH-4´´^{a}), 8.07 (s, 2H, CH-4´´^{b}), 7.94 (s, 2H, CH-2´´^{b}), 7.86 (br, 2H, CH-2´´^{a}), 7.31 (*virt.* t, $^3J \approx {}^3J$ = 7.6 Hz, 1H, CH-7), 6.96 (d, ^3J = 7.6 Hz, 1H, CH-6), 6.72 (*virt.* t, $^3J \approx {}^3J$ = 7.6 Hz, 1H, CH-5) 5.79 (d, ^2J = 7.3 Hz, 1H, CH^a), 5.67 (d, ^2J = 7.3 Hz, 1H, CH^b).

^{13}C-NMR (125 MHz, DMSO-d_6, 300 K) δ [ppm] = 176.0 (CO), 145.6 (C1´´^{b*}), 142.7 (C7a), 142.3 (C1´´^{a*}), 131.8 (C6), 130.6 (q, $^2J_{CF}$ = 32.1 Hz, 2C, C3´´^{a**}), 130.3 (q, $^2J_{CF}$ = 32.1 Hz, 2C, C3´´^{b**}) 129.0 (C2´´^{a}), 127 (C4), 127.3 (C2´´^{b}), 124.6 (C3a), 122.9 (q, $^1J_{CF}$ = 273.5 Hz, 2C, CF_3^{b***}), 122.6 (q, $^1J_{CF}$ = 273.5 Hz, 2C, CF_3^{a***}) 121.7 (C4´´^{a}), 121.6 (C5), 121.3(C4´´^{b}), 110.8 (C7), 91.0 (C3), 76.2 (C2´), 60.3 (C3´).

Signals marked with *, **and *** could not be assigned and can be interchanged.

^{19}F-NMR (376 MHz, DMSO-d_6, 300 K) δ [ppm] = –60.3, –61.4.

HRMS (ESI+) *m/z*: $[M+H]^+$ Calculated: 600.0827; Found: 600.0837

IR (ATR): $\tilde{\nu}$ [cm^{-1}] = 3154 (w, C-H_{arom}), 3097 (w, C-$H_{arom.}$), 3043 (w, C-$H_{arom.}$), 2982 (w, C-$H_{alip.}$), 2842 (w), 1733 (s, C=O), 1624 (m), 1471 (m), 1375 (m), 1276 (s), 1086 (m, C-O-C), 746 (m), 746 (s), 682 (m).

Chiral HPLC: OD-RH 250 × 4.6 mm, H_2O/MeCN = 80/20 → 0/100, 1 mL/min, λ = 210 nm, t_R = 19.4 min (**5h**), 20.0 (*ent*-**5h**).

3',3'-Bis(3-nitrophenyl)spiro[indoline-3,2'-oxetan]-2-one (*rac*-5i)

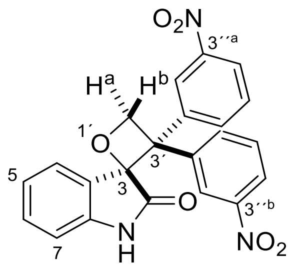

rac-**5i**
$C_{22}H_{15}N_3O_6$
M = 417.38 g/mol

Substrate *rac*-**5i** was synthesized according to **GP-D** from **S5a** (300 mg, 1.59 mmol, 1.00 equiv.) and 1.29 g of **9i** (4.76 mmol, 3.00 equiv.). After deprotection and purification 522 mg of *rac*-**5i** (1.25 mmol, 79%) were obtained as a white amorphous solid.

TLC: 0.30 (CH_2Cl_2:Ac = 97.5:2.5) [UV], [$KMnO_4$]

M.p.: decomp. under normal conditions

^{1}H-NMR (500 MHz, DMSO-d_6, 300 K) δ [ppm] = 10.81 (s, 1H, NH), 8.23 (s, 1H, CH-2´´^{a}), 8.20 (ddd, ^3J = 8.0 Hz, ^4J = 2.0 Hz, ^4J = 1.1 Hz, 1H, CH-4´´^{a}), 8.14 (ddd, ^3J = 8.3 Hz, ^4J = 2.3 Hz, ^4J = 1.0 Hz, 1H, CH-4´´^{b}), 7.95 (*virt.* t, $^4J \approx {}^4J$ = 2.3 Hz, 1H, CH-2´´^{b}), 7.67 (*virt.* t, $^3J \approx {}^3J$ = 8.3 Hz, 1H, CH-5´´^{b}), 7.64 (br, 1H, CH-6´´^{a}), 7.61 (*virt.* t, $^3J \approx {}^3J$ = 8.0 Hz, 1H, CH-5´´^{a}) 7.55 (ddd, ^3J = 8.3 Hz, ^4J = 2.3 Hz, ^4J = 1.0 Hz, 1H, CH-6´´^{b}), 7.27 (*virt.* td, $^3J \approx {}^3J$ = 7.7 Hz, ^4J = 1.3 Hz, 1H, CH-6) 6.93 (d, ^3J = 7.7 Hz, 1H, CH-7), 6.67 (*virt.* td, $^3J \approx {}^3J$ = 7.7 Hz, ^4J = 1.3 Hz, 1H, CH-5), 6.15 (d, ^3J = 7.6 Hz, 1H, CH-4), 5.74 (d, ^2J = 6.7 Hz, 1H, CH^b), 5.68 (d, ^2J = 6.7 Hz, 1H, CH^a).

^{13}**C-NMR** (125 MHz, DMSO-d_6, 300 K) δ [ppm] = 176.5 (CO), 147.9 (C3´´^{b}*), 147.9 (C3´´^{a}*), 144.3 (C1´´^{b}), 142.6 (C7a), 141.1 (C1´´^{a}), 135.5 (C6´´^{b}), 131.8 (C6), 130.4 (C5´´^{b}) 130.2 (C5´´^{a}), 127.1 (C4), 124.3 (C2´´^{a}) 122.8 (C4´´^{a}), 122.4 (C3a), 122.2 (C4´´^{b}), 121.7 (C5), 121.0 (C2´´^{b}), 110.6 (C7), 90.8 (C3), 76.2 (C2´), 60.3 (C3´).

Signals marked with * could not be assigned and can be interchanged.

HRMS (ESI+) *m/z*: $[M+H]^+$ Calculated: 418.1034; Found: 418.1035

IR (ATR): $\tilde{\nu}$ [cm^{-1}] = 3231 (w, NH), 3095 (w, C-$H_{arom.}$), 3008 (w, C-$H_{arom.}$), 2873 (w, C-$H_{alip.}$), 2798 (w), 1736 (m, C=O), 1620 (m), 1523 (s, N=O), 1352 (s, (N=O), 1328 (m), 1092 (w), 761 (m), 728 (s).

Chiral HPLC: OD-RH 250 × 4.6 mm, H_2O/MeCN = 80/20 → 0/100, 1 mL/min, λ = 210 nm, t_R = 18.1 min (**5i**), 19.3 (*ent*-**5i**).

(2-Oxospiro[indoline-3,2'-oxetane]-3',3'-diyl)-bis(4,1-phenylene) bis(4-methylbenzene-sulfonate) (*rac*-5j)

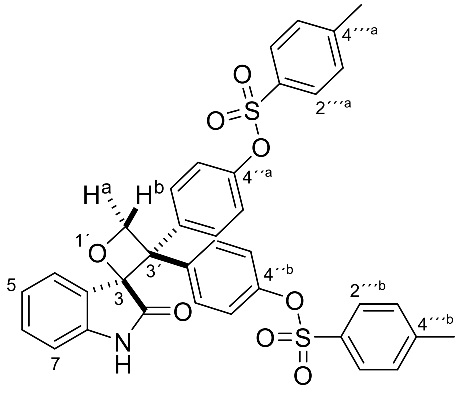

rac-**5j**
$C_{36}H_{29}NO_8S_2$
M = 667.75 g/mol

Substrate *rac*-**5j** was synthesized according to **GP-D** from **S5a** (300 mg, 1.59 mmol, 1.00 equiv.) and 2.48 g of **9j** (4.76 mmol, 3.00 equiv.). After deprotection and purification 831 mg of *rac*-**5j** (1.24 mmol, 78%) were obtained as a white amorphous solid.

TLC: 0.28 (CH_2Cl_2:Ac = 97.5:2.5) [UV], [$KMnO_4$]

M.p.: decomp. under normal conditions

^{1}**H-NMR** (500 MHz, DMSO-d_6, 300 K) δ [ppm] = 10.81 (s, 1H, NH), 7.78 – 7.48, (m, 2H, CH-2´´´^{a}), 7.69 – 7.66 (m, 2H, CH-2´´´^{b}), 7.51 – 7.48 (m, 2H, CH-3´´´^{a}), 7.45 – 7.41 (m, 2H, CH-3´´´^{b}), 7.30 – 7.21 (m, 3H, CH-2´´^{a}/CH-6), 7.02 – 6.98 (m, 2H, CH-3´´^{a}), 6.98 – 6.94 (m, 2H, CH-3´´^{b}), 6.93 – 6.89 (m, 2H, CH-2´´^{b}), 6.82 (d, ^{3}J = 7.7 Hz, 1H, CH-7), 6.60 (*virt.* t, $^{3}J \approx {}^{3}J$ = 7.7 Hz, 1H, CH-5) 5.86 (d, ^{3}J = 7.7 Hz, 1H, CH-4), 5.59 (d, ^{2}J = 6.2 Hz, 1H, CH^{b}), 5.37 (d, ^{2}J = 6.2 Hz, 1H, CH^{a}).

^{13}**C-NMR** (125 MHz, DMSO-d_6, 300 K) δ [ppm] = 176.9 (CO), 148.1 (C4´´^{a}), 147.5 (C4´´^{b}), 146.0 (C1´´´^{a}*), 145.9 (C1´´´^{b}*), 142.7 (C7a), 142.0 (C1´´^{b}), 138.6 (C1´´^{a}), 131.4 (C6), 131.4 (C4´´´^{a}) 131.3 (C4´´´^{b}), 130.3 (2C, C3´´´^{a}), 130.2 (2C, C3´´´^{b}), 130.0 (2C, C2´´^{a}), 128.3 (2C, C2´´´^{a}) 128.2 (2C, C2´´´^{b}), 127.8 (2C, C2´´^{b}), 126.5 (C4) 124.5 (C3a), 122.1 (2C, C3´´^{b}), 122.0 (C3´´^{a}), 121.2 (C5), 110.4 (C7), 90.6 (C3), 76.2 (C2´), 60.0 (C3´), 21.2 (CH_3^{a}**), 21.2 (CH_3^{b}**).

Signals marked with * and ** could not be assigned and can be interchanged.

HRMS (ESI+) *m/z*: $[M+H]^+$ Calculated: 668.1407; Found: 668.1400

IR (ATR): $\tilde{\nu}$ [cm^{-1}] = 3253 (w, NH), 3068 (w, C-$H_{arom.}$), 2971 (w, C-$H_{alip.}$), 2923 (w, C-$H_{alip.}$), 1731 (m, C=O), 1619 (m), 1597 (s), 1368 (s, (S=O), 1177 (m), 1153 (s), 750 (m), 662 (s).

Chiral HPLC: OD-RH 250 × 4.6 mm, H_2O/MeCN = 80/20 → 0/100, 1 mL/min, λ = 210 nm, t_R = 24.5 min (**5j**), 26.3 (*ent*-**5j**).

2-Oxospiro[indoline-3,2'-oxetane]-3',3'-diyl)bis(4,1-phenylene) bis(2,2-dimethylpropanoate (*rac*-5k)

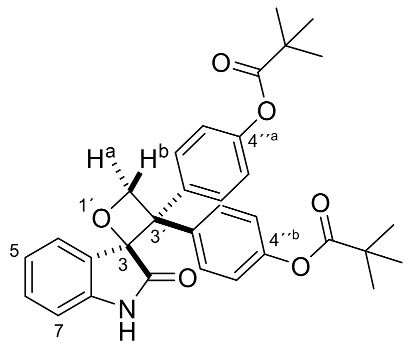

rac-**5k**
$C_{32}H_{33}NO_6$
M = 527.62 g/mol

Substrate *rac*-**5k** was synthesized according to **GP-D** from **S5a** (300 mg, 1.59 mmol, 1.00 equiv.) and 1.81 g of **9k** (4.76 mmol, 3.00 equiv.). After deprotection and purification 0.748 mg of *rac*-**5k** (1.42 mmol, 89%) were obtained as a white amorphous solid.

TLC: 0.33 (CH_2Cl_2:Ac = 97.5:2.5) [UV], [$KMnO_4$]

M.p.: decomp. under normal conditions

^{1}H-NMR (500 MHz, DMSO-d_6, 300 K) δ [ppm] = 10.59 (s, 1H, NH), 7.10 – 7.03, (m, 2H, CH-3´´^{a}), 7.21 (*virt.* td, $^3J \approx {}^3J$ = 7.7 Hz, ^4J = 1.1 Hz, 1H, CH-6), 7.10 – 7.03 (m, 4H, CH-3´´^{b}/CH-2´´^{a}), 6.99 – 6.94 (m, 2H, CH-2´´^{b}), 6.84 (d, ^3J = 7.7 Hz, CH-7), 6.62 (*virt.* td, $^3J \approx {}^3J$ = 7.7 Hz, ^4J = 1.1 Hz, 1H, CH-6), 6.09 (d, ^3J = 7.7 Hz, 1H, CH-4), 5.69 (d, ^2J = 6.7 Hz, 1H, CH^b), 5.46 (d, ^2J = 6.7 Hz, 1H, CH^a), 1.29 (s, 3H, $CH_3{}^b$), 1.28 (s, 3H, $CH_3{}^a$).

^{13}C-NMR (125 MHz, DMSO-d_6, 300 K) δ [ppm] = 177.2 (CONH), 176.3 (O*C*O^{t}Bu^{a}*), 176.2 (O*C*O^{t}Bu^{b}*), 149.8 (C4´´^{a}), 149.1 (C4´´^{b}), 142.7 (C1´´^{b}), 137.3 (C1´´^{a}), 131.3 (C6), 129.5 (2C, C3´´^{a}), 127.2 (2C, C2´´^{b}), 126.9 (C4), 124.9 (C3a), 121.6 (2C, C3´´^{b}), 121.6 (C2´´^{a}), 121.3 (C5), 110.2 (C7), 90.9 (C3), 76.2 (C2´), 60.0 (C3´), 38.6 (*C*(CH_3)$_3$^{b}), 38.6 [*C*(CH_3)$_3$^{a}], 26.8 [C(*C*H_3)$_3$^{a}**], 26.8 [C(*C*H_3)$_3$^{b}**)].

Signals marked with * and ** could not be assigned and can be interchanged.

HRMS (ESI+) *m/z*: $[M+H]^+$ Calculated: 528.2381; Found: 528.2385

IR (ATR): $\tilde{\nu}$ [cm^{-1}] = 3169 (w, NH), 3035 (w, C-H$_{arom.}$), 2970 (w, CH$_{2.}$), 2932 (w, C-H$_{alip.}$), 1743 (s, C=O), 1722 (s, C=O), 1619 (m), 1468 (m), 1202 (m), 1108 (s), 748 (s).

Chiral HPLC: AD-H 250 × 4.6 mm, *n*-Hep/^{i}PrOH = 90/10, 1 mL/min, λ = 210 nm, t_R = 12 7 min (**5k**), 24.3 (*ent*-**5k**).

5-Fluoro-3',3'-bis(4-fluorophenyl)spiro[indoline-3,2'-oxetan]-2-one (*rac*-5l)

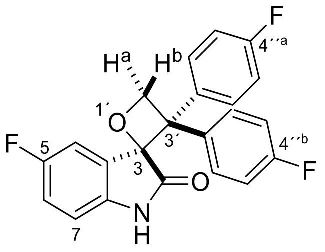

rac-**5l**
$C_{22}H_{14}F_3NO_2$
M = 381.35 g/mol

Substrate *rac*-**5l** was synthesized according to **GP-D** from **S5c** (300 mg, 1.59 mmol, 1.00 equiv.) and 939 mg of **9f** (4.76 mmol, 3.00 equiv.). After deprotection and purification 497 mg of *rac*-**5k** (1.30 mmol, 90%) were obtained as a white amorphous solid.

TLC: 0.34 (CH_2Cl_2:Ac = 97.5:2.5) [UV], [$KMnO_4$]

M.p.: decomp. under normal conditions

^{1}H-NMR (500 MHz, DMSO-d_6, 300 K) δ [ppm] = 10.66 (s, 1H, NH), 7.43 – 7.30, (m, 2H, CH-2´´^{a}), 7.23 – 7.19 (m, 2H, CH-3´´^{a}), 7.17 – 7.13 (m, 2H, CH-3´´^{b}), 7.10 (*virt.* td, $^3J_{HH} \approx {}^3J_{HF}$ = 8.8 Hz, $^4J_{HH}$ = 2.8 Hz, 1H, CH-6), 6.99 – 6.94 (m, 2H, CH-2´´^{b}), 6.85 (dd, $^3J_{HH}$ = 8.8 Hz, $^4J_{HF}$ = 4.5, 1H, CH-7), 5.79 (dd, $^3J_{HF}$ = 8.8 Hz, $^4J_{HH}$ = 2.8 Hz, 1H, CH-4), 5.65 (d, ^2J = 6.1 Hz, 1H, CH^b), 5.46 (d, ^2J = 6.1 Hz, 1H, CH^a).

^{13}C-NMR (125 MHz, DMSO-d_6, 300 K) δ [ppm] = 177.0 (CO), 161.5 (d, $^1J_{CF}$ = 246.7 Hz, C4´´^{b}), 159.8 (d, $^1J_{CF}$ = 246.7 Hz, C4´´^{a}), 157.7 (d, $^1J_{CF}$ = 237.4 Hz, C5), 139.0 (d, $^4J_{CF}$ = 3.5 Hz, C7a), 138.8 (d, $^4J_{CF}$ = 3.0 Hz, C1´´^{b}), 135.7 (d, $^4J_{CF}$ = 3.0 Hz, C1´´^{a}), 130.5 (d, $^3J_{CF}$ = 8.0 Hz, 2C, C2´´^{a}), 128.1 (d, $^3J_{CF}$ = 8.0 Hz, 2C, C2´´^{b}), 126.3 (d, $^3J_{CF}$ = 7.5 Hz, C3a), 117.8 (d, $^2J_{CF}$ = 23.1 Hz, C6), 115.4 (d, $^2J_{CF}$ = 23.8 Hz, 2C, C3´´^{a}*), 115.4 (d, $^2J_{CF}$ = 23.8 Hz, 2C, C3´´^{b}*), 114.0 (d, $^2J_{CF}$ = 25.4 Hz, CH-4), 111.2 (d, $^3J_{CF}$ = 7.5 Hz, C7), 90.6 (C3), 76.8 (C2´), 59.9 (C3´).

Signals marked with * could not be assigned and can be interchanged.

^{19}F-NMR (470 MHz, DMSO-d_6, 300 K) δ [ppm] = –115.0, –115.9, –121.7.

HRMS (ESI+) *m/z*: $[M+H]^+$ Calculated: 382.1049; Found: 382.1058

IR (ATR): $\tilde{\nu}$ [cm^{-1}] = 3237 (w, NH), 3083 (w, C-H$_{arom.}$), 2981 (w, CH$_{2.}$), 2907 (w, C-H$_{alip.}$), 1721 (s, C=O), 1603 (m), 1509 (s), 1476 (s), 1226 (m), 1167 (m), 824 (s), 748 (s).

Chiral HPLC: AD-H 250 × 4.6 mm, *n*-Hep/^{i}PrOH = 90/10, 1 mL/min, λ = 210 nm, t_R = 17.1 min (**5l**), 42.3 (*ent*-**5l**).

5-Fluoro-3',3'-di-*p*-tolylspiro[indoline-3,2'-oxetan]-2-one (*rac*-5m)

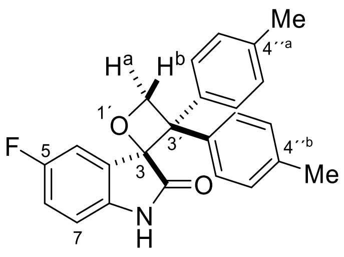

rac-**5m**
$C_{24}H_{20}FNO_2$
M = 373.43 g/mol

Substrate *rac*-**5m** was synthesized according to **GP-D** from **S5c** (300 mg, 1.59 mmol, 1.00 equiv.) and 905 mg of **9b** (4.76 mmol, 3.00 equiv.). After deprotection and purification 500 mg of *rac*-**5m** (1.34 mmol, 92%) were obtained as a white amorphous solid.

TLC: 0.37 (CH_2Cl_2:Ac = 97.5:2.5) [UV], [$KMnO_4$]

M.p.: decomp. under normal conditions

^{1}H-NMR (500 MHz, DMSO-*d*$_6$, 300 K) δ [ppm] = 10.55 (s, 1H, NH), 7.25 – 7.15, (m, 4H, CH-2´´^{a}/CH-3´´^{a}), 7.11 – 7.08 (m, 2H, CH-3´´^{b}), 7.05 (*virt.* td, $^{3}J_{HH} \approx {}^{3}J_{HF}$ = 8.8 Hz, $^{4}J_{HH}$ = 2.7 Hz, 1H, CH-6), 6.82 (dd, $^{3}J_{HH}$ = 8.8 Hz, $^{4}J_{HF}$ = 4.5 Hz, 1H, CH-7),6.79 – 6.74 (dd, $^{3}J_{HF}$ = 8.8 Hz, $^{4}J_{HH}$ = 2.7 Hz, 1H, CH-4), 5.64 (d, ^{2}J = 5.8 Hz, 1H, CH^{a}), 5.42 (d, ^{2}J = 5.8 Hz, 1H, CH^{b}), 2.28 (s, 3H, CH_3^{b}), .2.24 (s, 3H, CH_3^{a}).

^{13}C-NMR (125 MHz, DMSO-*d*$_6$, 300 K) δ [ppm] = 177.3 (CO), 175 (d, $^{1}J_{CF}$ = 236.4 Hz, C5), 140.2 (C1´´^{b}), 138.8 (d, 4J_{CF} = 1.8 Hz, C7a), 136.9 (C4´´^{a}), 136.6 (C1´´^{a}), 135.4 (C4´´b), 128.9 (2C, C3´´^{a}), 128.9 (2C, C3´´^{b}), 128.2 (2C, C2´´^{a}), 126.6 (d, ^{3}$_{CF}$ = 8.0 Hz, C3a), 125.9 (2C, C2´´^{b}) 117.8 (d, $^{2}J_{CF}$ = 23.6 Hz, C6), 114.3 (d, $^{2}J_{CF}$ = 25.1 Hz, C4), 110.9 (d, $^{3}J_{CF}$ = 7.5 Hz, C7), 90.8 (C3), 76.8 (C2´), 60.3 (C3´), 20.6 (CH_3^{b}), 20.5 (CH_3^{a}).

^{19}F-NMR (470 MHz, DMSO-*d*$_6$, 300 K) δ [ppm] = –121.9.

HRMS (ESI+) *m/z*: $[M+H]^+$ Calculated: 374.1551; Found: 374.1562

IR (ATR): $\tilde{\nu}$ [cm^{-1}] = 3237 (w, NH), 3024 (w, C-$H_{arom.}$), 2958 (w, C-$H_{alip.}$), 2922 (m, $CH_{2.}$), 1721 (s, C=O), 1475 (m), 1274 (m), 1170 (m), 812 (s), 765 (s), 749 (s).

Chiral HPLC: AD-H 250 × 4.6 mm, *n*-Hep/^{i}PrOH = 90/10, 1 mL/min, λ = 210 nm, t_R = 14.5 min (**5m**), 23.6 (*ent*-**5m**).

3',3'-Bis(4-chlorophenyl)-5-fluorospiro[indoline-3,2'-oxetan]-2-one (*rac*-5n)

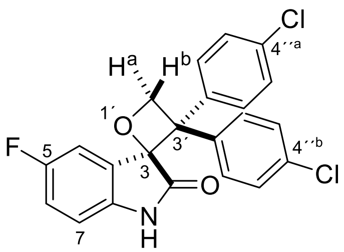

rac-**5n**
$C_{22}H_{14}Cl_2FNO_2$
M = 414.26 g/mol

Substrate *rac*-**5n** was synthesized according to **GP-D** from **S5c** (300 mg, 1.59 mmol, 1.00 equiv.) and 1.08 g of **9a** (4.76 mmol, 3.00 equiv.). After deprotection and purification 500 mg of *rac*-**5m** (1.34 mmol, 92%) were obtained as a white amorphous solid.

TLC: 0.37 (CH_2Cl_2:Ac = 97.5:2.5) [UV], [$KMnO_4$]

M.p.: decomp. under normal conditions

^{1}**H-NMR** (500 MHz, DMSO-d_6, 300 K) δ [ppm] = 10.69 (s, 1H, NH), 7.49 – 7.42, (m, 4H, CH-3´´^{a}), 7.40 – 7.30 (m, 2H, CH-3´´^{b}), 7.36 – 7.27 (m, 2H, CH-2´´^{a}), 7.10 (*virt.* td, $^3J_{HH}$ ≈ $^3J_{HF}$ = 8.8 Hz, $^4J_{HH}$ = 2.8 Hz, 1H, CH-6), 7.00 – 7.94 (m, 2H, CH-2´´^{b}), 6.86 (dd, $^3J_{HH}$ = 8.8 Hz, $^4J_{HF}$ = 4.5 Hz, 1H, CH-7), 5.86 (dd, $^3J_{HF}$ = 8.8 Hz, $^4J_{HH}$ = 2.8 Hz, 1H, CH-4), 5.64 (d, ^2J = 6.2 Hz, 1H, CH^a), 5.46 (d, ^2J = 6.2 Hz, 1H, CH^b).

^{13}**C-NMR** (125 MHz, DMSO-d_6, 300 K) δ [ppm] = 176.9 (CO), 157 (d, $^1J_{CF}$ = 236.9 Hz, C5), 141.5 (C4´´^{b}), 138.8 (C4´´^{a}), 138.2 (d, $^4J_{CF}$ = 1.5 Hz, C7a), 132.4 (C1´´b), 131.4 (C1´´^{a}), 130.2 (2C, C2´´^{a}), 128.5 (2C, C3´´^{a}*), 128.5 (2C, C3´´^{b}*) 128.1 (2C, C2´´^{b}), 126.1 (d, ^{3}CF = 8.0 Hz, C3a), 117.9 (d, $^2J_{CF}$ = 25.1 Hz, C6), 114.1 (d, $^2J_{CF}$ = 25.1 Hz, C4), 111.3 (d, $^3J_{CF}$ = 8.0 Hz, C7), 90.6 (C3), 76.6 (C2´), 60.0 (C3´).

^{19}**F-NMR** (470 MHz, DMSO-d_6, 300 K) δ [ppm] = –119.4.

HRMS (ESI+) *m/z*: [M+H]$^+$ Calculated: 414.0458; Found: 414.0468

IR (ATR): $\tilde{\nu}$ [cm^{-1}] = 3240 (w, NH), 2981 (w, C-H$_{alip.}$), 2905 (w, CH$_{2.}$), 1724 (s, C=O), 1475 (m), 1273 (m), 1170 (m), 1093 (m, C-O-C), 817 (s), 794 (s), 752 (s).

Chiral HPLC: AD-H 250 × 4.6 mm, *n*-Hep/^{i}PrOH = 70/30, 1 mL/min, λ = 210 nm, t_R = 6.9 min (**5n**), 17.4 (*ent*-**5n**).

6-Chloro-3',3'-bis(4-chlorophenyl)spiro[indoline-3,2'-oxetan]-2-one (*rac*-5o)

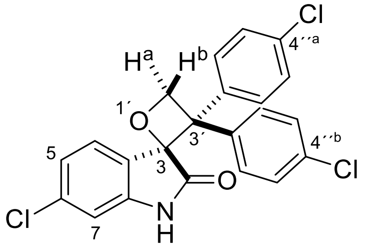

rac-**5o**
$C_{22}H_{14}Cl_3NO_2$
M = 430.71 g/mol

Substrate *rac*-**5o** was synthesized according to **GP-D** from **S5c** (300 mg, 1.59 mmol, 1.00 equiv.) and 1.08 g of **9a** (4.76 mmol, 3.00 equiv.). After deprotection and purification 500 mg of *rac*-**5m** (1.34 mmol, 92%) were obtained as a white amorphous solid.

TLC: 0.50 (CH_2Cl_2:Ac = 97.5:2.5) [UV], [$KMnO_4$]

M.p.: decomp. under normal conditions

^{1}H-NMR (500 MHz, DMSO-d_6, 300 K) δ [ppm] = 10.81 (s, 1H, NH), 7.45 – 7.40, (m, 2H, CH-3´´^{a}), 7.38 – 7.35 (m, 2H, CH-3´´^{b}), 7.35 – 7.29 (m, 2H, CH-2´´^{a}), 6.99 – 6.95 (m, 2H, CH-2´´^{b}), 6.88 (d, ^4J = 2.0 Hz, 1H, CH-7), 6.77 (dd, ^3J = 8.1 Hz, ^4J = 2.0 Hz, 1H, CH-5), 6.11 (d, ^3J = 8.1 Hz, 1H, CH-4), 5.63 (d, ^2J = 6.2 Hz, 1H, CH^b), 5.45 (d, ^2J = 6.2 Hz, 1H, CH^a).

^{13}C-NMR (125 MHz, DMSO-d_6, 300 K) δ [ppm] = 176.9 (CO), 144.2 (C7a), 141.7 (C4´´^{b}), 138.3 (C4´´^{a}), 135.8 (C6), 132.2 (C1´´^{b}), 131.4 (C1´´^{a}), 130.2 (C2´´^{a}), 128.5 (4C, C3´´^{b}/C3´´^{a}), 128.2 (C4), 128.1 (C2´´^{b}), 123.5 (C3a), 121.2 (C5), 110.6 (C7), 90.2 (C3), 76.4 (C2´), 60.0 (C3´).

HRMS (ESI+) *m/z*: $[M+H]^+$ Calculated: 430.0163; Found: 430.0149.

IR (ATR): $\tilde{\nu}$ [cm^{-1}] = 3240 (w, NH), 2981 (w, C-H$_{alip.}$), 2905 (w, CH$_{2.}$), 1724 (s, C=O), 1475 (m), 1273 (m), 1170 (m), 1093 (m, C-O-C), 817 (s), 794 (s), 752 (s).

Chiral HPLC: AD-H 250 × 4.6 mm, *n*-Hep/^{i}PrOH = 90/10, 1 mL/min, λ = 210 nm, t_R = 15.9 min (**5o**), 45.9 min (*ent*-**5o**).

3-(Bis(4-chlorophenyl)methylene)indolin-2-one (7a)

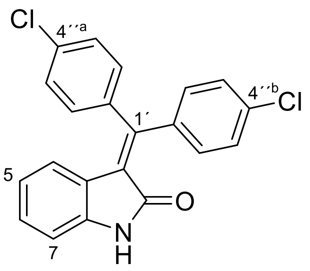

7a
$C_{21}H_{13}Cl_2NO$
M = 366.24 g/mol

Olefin **7a** was synthesized according to **GP-F** from oxindole **S6a** (200 mg, 1.50 mmol, 1.00 equiv.) together with bis(4-chlorophenyl)methanone (453 mg, 1.80 mmol, 1.20 equiv.). After purification 451 mg **7a** (1.23 mmol, 82%) were obtained as a bright yellow solid.

TLC: 0.48 (CH_2Cl_2:Ac = 97.5:2.5) [UV], [$KMnO_4$]

M.p.: 241 °C decomp.

^{1}H-NMR (500 MHz, DMSO-d_6, 300 K) δ [ppm] = 10.54 (s, 1H, NH), 7.60 – 7.51, (m, 2H, CH-3´´^{b}), 7.42 – 7.36 (m, 2H, CH-3´´^{a}), 7.36 – 7.31 (m, 2H, CH-2´´^{b}), 7.31 – 7.25 (m, 2H, CH-2´´^{a}), 7.11 (*virt.* td, $^3J \approx {}^3J$ = 7.7 Hz, ^4J = 1.2 Hz, 1H, CH-6), 6.79 (d, ^3J = 7.7 Hz. 1H, CH-7), 6.64 (*virt.* td, $^3J \approx {}^3J$ = 7.6 Hz, ^4J = 1.2 Hz, 1H, CH-5), 6.23 (d, ^3J = 7.7 Hz, 1H, CH-4).

^{13}C-NMR (125 MHz, DMSO-d_6, 300 K) δ [ppm] = 168.6 (CO), 151.7 (C1´), 141.1 (C7a), 139.4 (C4´´^{b}), 137.9 (C4´´^{a}), 135.8 (C1´´^{b}), 135.7 (C1´´^{a}), 132.1 (2C, C3´´^{a}), 131.3 (2C, C3´´^{b}), 129.5 (2C, C2´´^{b}), 128.3 (2C, C2´´^{a}), 125.6 (C3a), 123.6 (C3), 123.3 (C4), 121.6 (C5), 110.1 (C7).

HRMS (ESI+) *m/z*: $[M+H]^+$ Calculated: 366.0447; Found: 366.0446

IR (ATR): $\tilde{\nu}$ [cm^{-1}] = 3203 (w, NH), 3065 (w, $CH_{arom.}$) 3028 (w, $CH_{arom.}$), 2885 (w, C-$H_{alip.}$), 2690 (w, C-H_{alip}), 1693 (s, C=O), 1586 (m), 1465 (s), 1331 (m), 1088 (m), 1015 ms), 790 (m), 752 (s), 747 (s).

Chiral HPLC: AD-H 250 × 4.6 mm, *n*-Hep/^{i}PrOH = 90/10, 1 mL/min, λ = 210 nm, t_R = 20.5 min (**7a**).

3-(Di-*p*-tolylmethylene)indolin-2-one (7b)

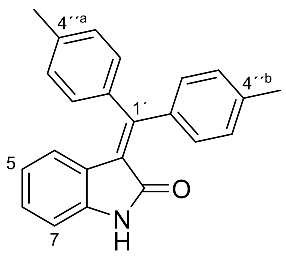

7b
$C_{23}H_{19}NO$
M = 325.4 g/mol

Olefin **7b** was synthesized according to **GP-F** from oxindole **S6a** (200 mg, 1.50 mmol, 1.00 equiv.) together with di-*p*-tolylmethanone (379 mg, 1.80 mmol, 1.20 equiv.). After purification 440 mg **7b** (1.35 mmol, 90%) were obtained as a bright yellow solid.

TLC: 0.35 (CH_2Cl_2:Ac = 97.5:2.5) [UV], [$KMnO_4$]

M.p.: 244 °C

^{1}H-NMR (500 MHz, $CDCl_3$, 300 K) δ [ppm] = 8.80 (s, 1H, NH), 7.29 – 7.26, (m, 2H, CH-2´´^{a}), 7.25 – 7.20 (m, 4H, CH-2´´^{b}/CH-3´´^{b}), 7.19 – 7.15 (m, 2H, CH-3´´^{a}), 7.05 (*virt.* td, $^3J \approx {}^3J$ = 7.7 Hz, ^4J = 1.3 Hz, 1H, CH-6), 6.67 – 6.62 (m, 2H, CH-7/CH-5), 6.47 (d, ^3J = 7.7 Hz, 1H, CH-4), 2.45 (s, 3H, CH_3^b), 2.40 (s, 3H, CH_3^a).

^{13}C-NMR (125 MHz, $CDCl_3$, 300 K) δ [ppm] = 168.9 (CO), 155.7 (C1´), 140.6 (C7a), 139.7 (C4´´^{a}), 139.6 (C4´´^{b}), 138.7 (C1´´^{b}), 137.2 (C1´´^{a}), 130.9 (2C, C2´´^{a}), 129.9 (2C, C2´´^{b}), 129.6 (2C, C3´´^{b}), 128.6 (2C, C3´´^{a}), 128.4 (C6), 124.6 (C3a), 124.0 (C3), 123.1 (C4), 121.1 (C5), 109.7 (C7), 21.7 (CH_3^{a*}), 21.6 (CH_3^{b*}).

HRMS (ESI+) *m/z*: $[M+H]^+$ Calculated: 326.1539; Found: 326.1555

IR (ATR): $\tilde{\nu}$ [cm^{-1}] = 3189 (w, NH), 3068 (w, $CH_{arom.}$) 3024 (w, $CH_{arom.}$), 2945 (w, C-$H_{alip.}$), 2867 (w, C-H_{alip}), 1689 (s, C=O), 1582 (m), 1463 (s), 1180 (m), 745 (s), 704 (m), 678 (m).

Chiral HPLC: OD-RH 250 × 4.6 mm, H_2O/MeCN = 80/20 → 0/100, 1 mL/min, λ = 210 nm, t_R = 21.1 min (**7b**).

3-(Bis(4-(*tert*-butyl)phenyl)methylene)indolin-2-one (7c)

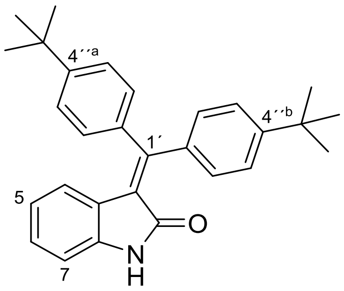

7c
$C_{29}H_{31}NO$
M = 409.57 g/mol

Olefin **7c** was synthesized according to **GP-F** from oxindole **S6a** (200 mg, 1.50 mmol, 1.00 equiv.) together with benzophenone **S4a** (531 mg, 1.80 mmol, 1.20 equiv.). After purification 424 mg **7c** (1.28 mmol, 85%) were obtained as a bright yellow solid.

TLC: 0.45 (CH_2Cl_2:Ac = 97.5:2.5) [UV], [$KMnO_4$]

M.p.: 283 °C

^{1}**H-NMR** (500 MHz, $CDCl_3$, 300 K) δ [ppm] = 8.39 (s, 1H, NH), 7.45 – 7.41, (m, 2H, CH-3´´^{a}), 7.39 – 7.35 (m, 2H, CH-3´´^{b}), 7.33 – 7.29 (m, 2H, CH-2´´^{b}), 7.25 – 7.23 (m, 2H, CH-2´´^{a}), 7.06 (*virt.* td, $^3J \approx {}^3J$ = 7.7 Hz, ^4J = 1.1 Hz, 1H, CH-6), 6.71 (d, ^3J = 7.7 Hz, 1H, CH-7), 6.64 (*virt.* td, $^3J \approx {}^3J$ = 7.7 Hz, ^4J = 1.1 Hz, 1H, CH-5), 6.38 (d, ^3J = 7.7 Hz, 1H, CH-4), 1.38 (s, 9H, $CH_3{}^{b*}$), 1.35 (s, 9H, $CH_3{}^{a*}$).

Signals marked with * could not be assigned and can be interchanged.

^{13}**C-NMR** (125 MHz, $CDCl_3$, 300 K) δ [ppm] = 168.8 (CO), 156.1 (C1´), 152.8 (C4´´^{a}), 152.6 (C4´´^{b}), 140.4 (C7a) 138.6 (2C, C1´´^{a}), 136.9 (2C, C1´´^{b}), 130.7 (2C, C2´´^{b}), 129.6 (2C, C2´´^{a}), 128.3 (C6), 125.8 (2C, C3´´^{a}), 124.8 (C3a), 124.7 (2C, C3´´^{b}), 123.9 (C3), 123.2 (C4), 121.2 (C5), 109.5 (C7), 35.0, [$C(CH_3)_3{}^{a*}$], 34.9 [$C(CH_3)_3{}^{b*}$], 31.5 [3C, $C(CH_3)_3{}^{a**}$], 31.4 [3C, $C(CH_3)_3{}^{b**}$].

Signals marked with * and ** could not be assigned and can be interchanged.

HRMS (ESI+) *m/z*: $[M+H]^+$ Calculated: 410.2478; Found: 410.2484

IR (ATR): $\tilde{\nu}$ [cm^{-1}] = 3267 (w, NH), 3058 (w, $CH_{arom.}$) 3027 (w, $CH_{arom.}$), 2961 (m, C-$H_{alip.}$), 2867 (w, C-H_{alip}), 1698 (s, C=O), 1465 (m), 1206 (m), 818 (m), 746 (s), 660 (m).

Chiral HPLC: AD-H 250 × 4.6 mm, n-Hep/^{i}PrOH = 90/10, 1 mL/min, λ = 210 nm, t_R = 7.0 min (**7c**).

3-(Bis(3,5-di-*tert*-butylphenyl)methylene)indolin-2-one (7d)

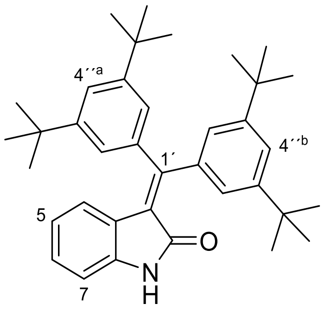

7d

$C_{37}H_{47}NO$

M = 521.79 g/mol

Olefin **7d** was synthesized according to **GP-F** from oxindole **S6a** (200 mg, 1.50 mmol, 1.00 equiv.) together with benzophenone **S4b** (733 mg, 1.80 mmol, 1.20 equiv.). After purification 711 mg **7d** (1.28 mmol, 85%) were obtained as a bright yellow solid.

TLC: 0.51 (CH_2Cl_2:Ac = 97.5:2.5) [UV], [$KMnO_4$]

M.p.: 250 °C decomp.

^{1}H-NMR (500 MHz, $CDCl_3$, 300 K) δ [ppm] = 8.63 (s, 1H, NH), 7.48 (t, ^4J = 1.8 Hz, 1H, CH-4´´^{a}), 7.43 (t, ^4J = 1.8 Hz, 1H, CH-4´´^{b}) 7.25 (d, ^4J = 1.8 Hz, 2H, CH-2´´^{b}), 7.14 (d, ^4J = 1.8 Hz, 2H, Ch-2´´^{a}), 7.02 (*virt.* td, $^3J \approx {}^3J$ = 7.7 Hz, ^4J = 1.2 Hz, 1H, CH-6), 6.71 (d, ^3J = 7.7 Hz, 1H, CH-7), 6.59 (*virt.* td, $^3J \approx {}^3J$ = 7.7 Hz, ^4J = 1.0 Hz, 1H, CH-5), 6.34 (d, ^3J = 7.7 Hz, 1H, CH-4), 1.29 (s, 18H, $CH_3{}^b$), 1.28 (s, 18H, $CH_3{}^a$).

^{13}C-NMR (125 MHz, $CDCl_3$, 300 K) δ [ppm] = 169.1 (CO), 158.4 (C1´), 151.1 (2C, C3´´^{a}), 149.2 (2C, C3´´^{b}), 141.0 (C1´´^{a}) 140.3 (C7a), 138.5 (C1´´^{b}), 128.1 (C6), 126.3 (2C, C2´´^{b}), 125.2 (C3a), 124.9 (2C, C2´´^{a}), 123.6 (C3), 123.5 (C4), 123.5 (C4´´^{b}), 122.7 (C4´´^{a}), 121.0 (C5), 109.3 (C7), 35.1 [2C, *C*(CH_3)$_3$^{a}*], 35.0 [2C, *C*(CH_3)$_3$^{b}*], 31.6 [6C, C(*C*H_3)$_3$^{b}], 31.4 [6C, C(*C*H_3)$_3$^{a}].

Signals marked with * could not be assigned and can be interchanged.

HRMS (ESI+) *m/z*: $[M+H]^+$ Calculated: 522.3730; Found: 522.3740

IR (ATR): $\tilde{\nu}$ [cm^{-1}] = 3169 (w, NH), 3069 (w, $CH_{arom.}$) 3027 (w, $CH_{arom.}$), 2962 (m, C-$H_{alip.}$), 1687 (s, C=O), 1467 (m), 1246 (m), 1195 (m), 879 (w), 747 (s), 716 (m).

Chiral HPLC: OD-RH 250 × 4.6 mm, H_2O/MeCN = 80/20 → 0/100, 1 mL/min, λ = 210 nm, t_R = 26.5 min (**7d**).

3-(Diphenylmethylene)indolin-2-one (7e)

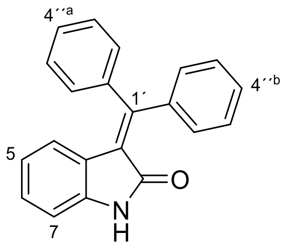

7e
$C_{21}H_{15}NO$
M = 297.36 g/mol

Olefin **7e** was synthesized according to **GP-F** from oxindole **S6a** (200 mg, 1.50 mmol, 1.00 equiv.) together with benzophenone (328 mg, 1.80 mmol, 1.20 equiv.). After purification 341 mg **7e** (1.14 mmol, 76%) were obtained as a bright yellow solid.

TLC: 0.47 (CH_2Cl_2:Ac = 97.5:2.5) [UV], [$KMnO_4$]

M.p.: 260 °C decomp.

^{1}H-NMR (500 MHz, DMSO-d_6, 300 K) δ [ppm] = 10.48 (s, 1H, NH), 7.23 – 7.46 (m, 3H, CH^{Ar}), 7.34 – 7.25 (m, 7H, CH^{Ar}), 7.08 (*virt.* t, $^3J \approx {}^3J$ = 7.8 Hz, 1H, CH-6), 6.71 (d, ^3J = 7.8 Hz, 1H, CH-7), 6.57 (*virt.* t, $^3J \approx {}^3J$ = 7.8 Hz, 1H, CH-5), 6.34 (d, ^3J = 7.8 Hz, 1H, CH-4).

^{13}C-NMR (125 MHz, DMSO-d_6, 300 K) δ [ppm] = 167.1 (CO), 152.8 (C1´), 142.0 (C1´´^{a}), 141.4 (C7a), 139.8 (C1´´^{b}), 129.7 (2C, C2´´^{a}*), 129.2 (2C, C2´´^{b}*), 129.1 (C4´´^{a}**), 129.0 (C4´´^{b}**), 128.5 (C6), 128.4 (2C, C3´´^{a}***) 127.7 (2C, C3´´^{b}***), 124.8 (3a), 123.3 (C4), 122.7 (C5), 120.5 (C3), 109.4 (C7).

Signals marked with *, ** and *** could not be assigned and can be interchanged.

HRMS (ESI+) *m/z*: $[M+H]^+$ Calculated: 298.1226; Found: 298.1237

IR (ATR): $\tilde{\nu}$ [cm^{-1}] = 3168 (w), 3058 (w, CH$_{arom.}$) 3023 (w, CH$_{arom.}$), 2832 (m, C-H$_{alip.}$), 1694 (s, C=O), 1587 (m), 1463 (m), 1159 (m), 1026 (m), 879 (w), 745 (s), 693 (m).

Chiral HPLC: AD-H 250 × 4.6 mm, *n*-Hep/^{i}PrOH = 90/10, 1 mL/min, λ = 210 nm, t_R = 20.1 min (**7e**).

3-(Bis(4-fluorophenyl)methylene)indolin-2-one (7f)

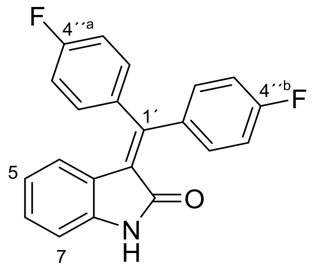

7f
$C_{21}H_{13}F_2NO$
M = 333.34 g/mol

Olefin **7f** was synthesized according to **GP-F** from oxindole **S6a** (200 mg, 1.50 mmol, 1.00 equiv.) together with bis(4-fluorophenyl)methanone (393 mg, 1.80 mmol, 1.20 equiv.). After purification 405 mg **7f** (1.21 mmol, 81%) were obtained as a bright yellow solid.

TLC: 0.35 (CH_2Cl_2:Ac = 97.5:2.5) [UV], [$KMnO_4$]

M.p.: 212 °C

^{1}H-NMR (500 MHz, DMSO-d_6, 300 K) δ [ppm] = 10.52 (s, 1H, NH), 7.36 – 7.29 (m, 6H, CH-2´´^{a}, CH-2´´^{b}, CH-3´´^{b}), 7.19 – 7.13 (m, 2H, CH-3´´^{a}), 7.10 (*virt.* t, $^3J \approx {}^3J$ = 7.7 Hz, 1H, CH-6), 6.77 (d, ^3J = 7.7 Hz, 1H, CH-7), 6.63 (*virt.* t, $^3J \approx {}^3J$ = 7.7 Hz, 1H, CH-5), 6.15 (d, ^3J = 7.7 Hz, 1H, CH-4).

^{13}C-NMR (125 MHz, DMSO-d_6, 300 K) δ [ppm] = 167.5 (CO), 163.2 (d, $^1J_{CF}$ = 240 Hz, C4´´^{b}), 162.9 (d, $^1J_{CF}$ = 240, C4´´^{a}), 150.7 (C1´), 142.5 (C7a), 137.9 (d, $^4J_{CF}$ = 3.5 Hz, C1´´^{b}), 136.4 (d, $^4J_{CF}$ = 3.5 Hz, C1´´^{a}), 131.8 (d, $^3J_{CF}$ = 8.5 Hz, 2C, C2´´^{a}), 131.7 (d, $^3J_{CF}$ = 8.5 Hz, 2C, C2´´^{b}), 129.6 (C6), 125.7 (C3a), 123.6 (C3), 123.0 (C4), 121.1 (C5), 116.9 (d, $^2J_{CF}$ = 21.6 Hz, 2C, C3´´^{b}), 115.2 (d, $^2J_{CF}$ = 21.6 Hz, 2C, C3´´^{a}), 110.0 (C7).

^{19}F-NMR (470 MHz, DMSO-d_6, 300 K) δ [ppm] = –110.7, –111.0.

HRMS (ESI+) *m/z*: $[M+H]^+$ Calculated: 334.1038; Found: 334.1048

IR (ATR): $\tilde{\nu}$ [cm^{-1}] = 3164 (w), 3042 (w, $CH_{arom.}$) 3027 (w, $CH_{arom.}$), 2885 (m, C-$H_{alip.}$), 1686 (s, C=O), 1600 (m), 1501 (m), 1219 (m), 1153 (m), 815 (w), 803 (m), 748 (s).

Chiral HPLC: AD-H 250 × 4.6 mm, *n*-Hep/^{i}PrOH = 70/30, 1 mL/min, λ = 210 nm, t_R = 8.1 min (**7f**).

3-(Bis(4-bromophenyl)methylene)indolin-2-one (7g)

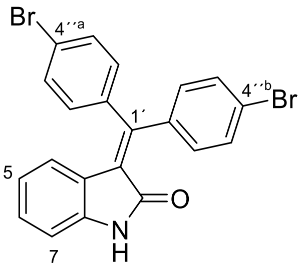

7g

$C_{21}H_{13}Br_2NO$

M = 455.15 g/mol

Olefin **7g** was synthesized according to **GP-F** from oxindole **S6a** (200 mg, 1.50 mmol, 1.00 equiv.) together with bis(4-bromophenyl)methanone (613 mg, 1.80 mmol, 1.20 equiv.). After purification 635 mg **7g** (1.40 mmol, 93%) were obtained as a bright orange solid.

TLC: 0.43 (CH_2Cl_2:Ac = 97.5:2.5) [UV], [$KMnO_4$]

M.p.: 259 °C

^{1}H-NMR (500 MHz, DMSO-d_6, 300 K) δ [ppm] = 10.56 (s, 1H, NH), 7.72 – 7.68 (m, 2H, CH-3´´^{a}), 7.54 – 7.50 (m, 2H, CH-3´´^{b}), 7.29 – 7.25 (m, 2H, CH-2´´^{b}), 7.24 – 7.19 (m, 2H, CH-2´´^{a}), 7.12 (*virt.* td, $^3J \approx {}^3J$ = 7.7 Hz, ^4J = 1.2 Hz, 1H, CH-6), 6.78 (d, ^3J = 7.7 Hz, 1H, CH-7), 6.66 (*virt.* td, $^3J \approx {}^3J$ = 7.7 Hz, ^4J = 1.2 Hz, 1H, CH-5), 6.23 (d, ^3J = 7.7 Hz, 1H, CH-4).

^{13}C-NMR (125 MHz, DMSO-d_6, 300 K) δ [ppm] = 167.0 (CO), 149.5 (C1´), 142.2 (C7a), 139.9 (C4´´^{a}), 138.7 (C4´´^{b}), 132.3 (C2´´^{a}), 131.9 (C2´´^{b}), 131.0 (C3´´^{b}), 130.8 (C3´´^{a}), 129.5 (C6), 125.6 (C3a), 122.8 (C3), 122.7 (2C, C1´´^{a}/C1´´^{b}), 122.2 (C4), 120.8 (C5), 109.7 (C7).

HRMS (ESI+) *m/z*: $[M+H]^+$ Calculated: 453.9437; Found: 453.9433

IR (ATR): $\tilde{\nu}$ [cm^{-1}] = 3166 (w), 3068 (w, $CH_{arom.}$) 3027 (w, $CH_{arom.}$), 2960 (w, C-$H_{alip.}$), 2832 (w, C-$H_{alip.}$), 2691 (w, C-$H_{alip.}$), 1688 (s, C=O), 1614 (m), 1582 (m), 1463 (m), 1292 (m), 1262 (m), 1069 (m), 1011 (m), 821 (m), 734 (s).

Chiral HPLC: AD-H 250 × 4.6 mm, *n*-Hep/^{i}PrOH = 70/30, 1 mL/min, λ = 210 nm, t_R = 9.8 min (**7g**).

3-(Bis(3,5-bis(trifluoromethyl)phenyl)methylene)indolin-2-one (7h)

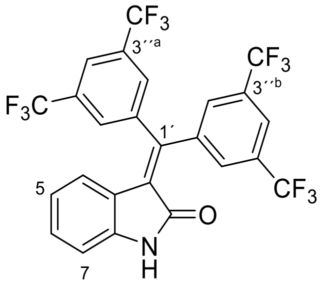

7h
$C_{25}H_{11}F_{12}NO$
M = 569.06 g/mol

Olefin **7h** was synthesized according to **GP-F** from oxindole **S6a** (200 mg, 1.50 mmol, 1.00 equiv.) together with commercially available bis(3,5-bis(trifluoromethyl)phenyl)methanone (613 mg, 1.80 mmol, 1.20 equiv.). After purification 739 mg **7h** (1.30 mmol, 86%) were obtained as a bright red solid.

TLC: 0.59 (CH_2Cl_2:Ac = 97.5:2.5) [UV], [$KMnO_4$]

M.p.: 251 °C decomp.

^{1}H-NMR (500 MHz, DMSO-d_6, 300 K) δ [ppm] = 10.67 (s, 1H, NH), 8.34 (s, 2H, CH-2´´^{a}), 8.22 (s, 1H, CH-4´´^{a}), 8.19 (s, 2H, CH-2´´^{b}), 8.04 (s, 1H, CH-4´´^{b}), 7.18 (*virt.* t, $^3J \approx {^3J}$ = 7.7 Hz, 1H, CH-6), 6.85 (d, ^3J = 7.7 Hz, 1H, CH-7), 6.66 (*virt.* t, $^3J \approx {^3J}$ = 7.7 Hz, 1H, CH-5), 5.95 (d, ^3J = 7.7 Hz, 1H, CH-4).

^{13}C-NMR (125 MHz, $CDCl_3$, 300 K) δ [ppm] = 167.0 (CO), 145.2 (C1´), 141.6 (C1´´^{a})*, 141.6 (C1´´^{b})*, 139.9 (C7a), 133.3 (q, $^{2}J_{CF}$ = 34 Hz, 2C, C3´´^{a}), 131.6 (q, $^{2}J_{CF}$ = 34 Hz, 2C, C3´´^{b}), 131.4 (C6), 130.4 (q, $^{3}J_{CF}$ = 3.0 Hz, 2C, C2´´^{b}), 129.9 (q, $^{3}J_{CF}$ = 3.0 Hz, 2C, C2´´^{a}), 128.7 (C3), 123.8 (sept., $^{3}J_{CF}$ = 4.0 Hz, C4´´^{a}), 123.5 (sept., $^{3}J_{CF}$ = 4.1 Hz, C4´´^{b}), 123.5 (C4), 123.1 (q., $^{1}J_{CF}$ = 273 Hz, 2C, CF_3^{a}), 119.6 (q, $^{1}J_{CF}$ = 273 Hz, CF_3^{b}), 122.1 (C5), 121.8 (C3a), 110.5 (C7).

Signals marked with * could not be assigned and can be interchanged.

^{19}F-NMR (470 MHz, DMSO-d_6, 300 K) δ [ppm] = –61.1, –61.1.

HRMS (ESI+) *m/z*: $[M+H]^+$ Calculated: 570.0722; Found: 570.0728

IR (ATR): $\tilde{\nu}$ [cm^{-1}] = 3184 (w, $CH_{arom.}$), 3033 (w, $CH_{arom.}$) 3011 (w, $CH_{arom.}$), 2902 (w, C-$H_{alip.}$), 1702 (s, C=O), 1619 (m, C=C), 1371 (m), 1275 (s), 1123 (s), 899 (m), 745 (m), 681 (s).

Chiral HPLC: OD-RH 250 × 4.6 mm, H_2O/MeCN = 80/20 → 0/100, 1 mL/min, λ = 210 nm, t_R = 20.1 min (**7h**).

3-(Bis(3-nitrophenyl)methylene)indolin-2-one (7i)

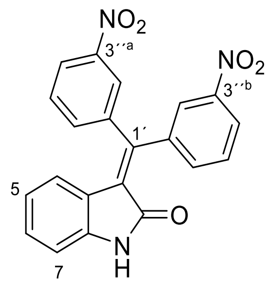

7i
$C_{21}H_{13}N_3O_5$
M = 387.09 g/mol

Olefin **7i** was synthesized according to **GP-F** from oxindole **S6a** (200 mg, 1.50 mmol, 1.00 equiv.) together with commercially available bis(3-nitrophenyl)methanone (491 mg, 1.80 mmol, 1.20 equiv.). After purification 255 mg **7i** (0.66 mmol, 44%) were obtained as a bright orange solid.

TLC: 0.30 (CH_2Cl_2:Ac = 97.5:2.5) [UV], [$KMnO_4$]

M.p.: 272 °C

^{1}H-NMR (500 MHz, DMSO-d_6, 300 K) δ [ppm] = 10.64 (s, 1H, NH), 8.35 (ddd, ^3J = 8.2 Hz, ^4J = 2.1 Hz, ^4J = 1.2 Hz, 1H, CH-4´´^{a}), 3.31 (t, ^4J = 2.1 Hz, 1H, CH-2´´^{a}), 8.28 (t, ^4J = 2.2 Hz, 1H, CH-2´´^{b}), 8.21 (ddd, ^3J = 8.2 Hz, ^4J = 2.2 Hz, ^4J = 1.1 Hz, 1H, CH-4´´^{b}), 7.93 (dt, ^3J = 7.7 Hz, ^4J = 1.2 Hz. 1H, CH-6´´^{a}), 7.85 – 7.79 (m, 2H, CH-5´´^{a}/CH-6´´^{b}), 7.66 (*virt.* t, $^3J \approx {}^3J$ = 8.2 Hz, 1H, CH-5´´^{b}), 7.17 (*virt.* td, $^3J \approx {}^3J$ = 7.7 Hz, ^4J = 1.2 Hz, 1H, CH-6), 6.82 (d, ^3J = 7.7 Hz, 1H, CH-7), 6.65 (*virt.* td, $^3J \approx {}^3J$ = 7.7 Hz, ^4J = 1.2 Hz, 1H, CH-5), 6.16 (d, 1H, CH-4).

^{13}C-NMR (125 MHz, DMSO-d_6, 300 K) δ [ppm] = 166.9 (CO), 148.5 (C3´´^{a}), 147.5 (C3´´^{b}), 145.9 (C1´), 142.8 (C7a), 141.5 (C1´´^{a}), 140.8 (C1´´^{b}), 136.2 (C6´´^{b}), 135.5 (C6´´^{a}), 131.3 (C5´´^{a}), 130.4 (C6), 129.7 (C5´´^{b}), 127.5 (C3), 124.5 (C2´´^{b}), 124.2 (C4´´^{a}), 123.5 (C2´´^{a}), 123.3 (C4´´^{b}), 123.2 (C4), 122.1 (C3a), 121.2 (C5), 110.0 (C7).

HRMS (ESI+) *m/z*: $[M+H]^+$ Calculated: 388.0928; Found: 388.0930

IR (ATR): $\tilde{\nu}$ [cm^{-1}] = 3268 (w, NH), 3079 (w, $CH_{arom.}$), 3005 (w, $CH_{arom.}$), 2990 (w, C-$H_{alip.}$), 1704 (m, C=O), 1670 (m), 1522 (s, N=O), 1344 (s), 1332 (m), 750 (s), 671 (m).

Chiral HPLC: OD-RH 250 × 4.6 mm, H_2O/MeCN = 80/20 → 0/100, 1 mL/min, λ = 210 nm, t_R = 19.7 min (**7i**).

((2-Oxoindolin-3-ylidene)methylene)bis(4,1-phenylene) bis(4-methylbenzenesulfonate) 7j

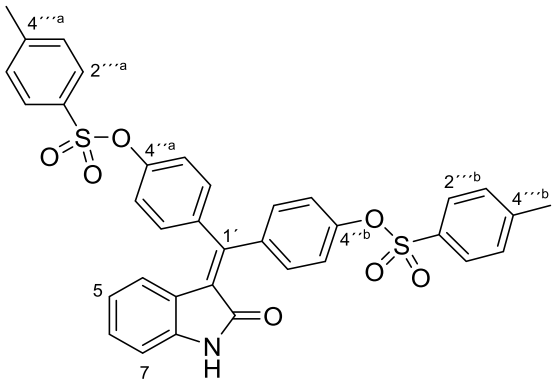

7j
$C_{35}H_{27}NO_7S_2$
M = 637.12 g/mol

Olefin **7j** was synthesized according to **GP-F** from oxindole **S6a** (200 mg, 1.50 mmol, 1.00 equiv.) together with Ketone **S4d** (942 mg, 1.80 mmol, 1.20 equiv.). After purification 627 mg **7j** (0.98 mmol, 65%) were obtained as a bright yellow solid.

^{1}H-NMR (500 MHz, DMSO-d_6, 300 K) δ [ppm] = 10.51 (s, 1H, NH), 7.80 – 7.73(m, 4H, CH-2´´´^{a}/CH-2´´´^{b}), 7.51 – 7.44 (m, 4H, CH-3´´´^{a}/CH-3´´´^{b}), 7.30 – 7.23 (m, 4H, CH-3´´^{a}/ CH-3´´^{b}), 7.16 – 7.10 (m, 4H, CH-2´´^{a}/CH-6), 7.00 – 6.97 (m, 2H, CH-2´´^{b}), 6.77 (d, ^3J = 7.7 Hz), 6.61 (*virt.* td, $^3J \approx {}^3J$ = 7.7 Hz, ^4J = 1.1 Hz, 1H, CH-5), 5.85 (d, ^3J = 7.7 Hz, 1H CH-4).

^{13}**C-NMR** (125 MHz, DMSO-d_6, 300 K) δ [ppm] = 166.8 (CO), 149.3 (C4´´^{b}), 149.1 (C1´), 149.0 (C4´´^{a}), 146.1 (C4´´´^{a})*,145.9 (C4´´´^{b})*, 142.3 (C1´´´^{a})**, 139.9 (C1´´^{a}), 138.4 (C1´´^{b}), 131.6 (C1´´´^{b})**, 131.4 (2C, C3´´^{a}), 131.0 (C7a), 130.3 (2C, C3´´^{b}), 130.3 (2C, C3´´´^{a}), 130.3 (2C, C3´´´^{b}), 129.6 (C6), 128.4 (2C, C2´´´^{b})***, 128.4 (2C, C2´´´^{a})***, 125.2 (C3a), 123.2 (2C, C2´´^{a}), 122.7 (C3), 122.6 (C4), 121.3 (2C, C2´´^{b}), 120.5 (C5), 109.7 (C7), 21.2 (CH_3^b)****, 21.2 (CH_3^a)****.

Signals marked with *, **, ***, **** could not be assigned and can be interchanged.

HRMS (ESI+) *m/z*: $[M+H]^+$ Calculated: 638.1302; Found: 638.1294

IR (ATR): $\tilde{\nu}$ [cm^{-1}] = 3194 (w, NH), 3066 (w, $CH_{arom.}$), 3006 (w, $CH_{arom.}$), 2925 (w, C-$H_{alip.}$), 1697 (m, C=O), 1596 (m), 1497 (m), 1370 (s, S=O), 1175 (s), 1149 (s), 1091 (s), 745 (s).

Chiral HPLC: OD-RH 250 × 4.6 mm, H_2O/MeCN = 80/20 → 0/100, 1 mL/min, λ = 210 nm, t_R = 24.6 min (**7j**).

((2-Oxoindolin-3-ylidene)methylene)bis(4,1-phenylene) bis(2,2-dimethylpropanoate) (7k)

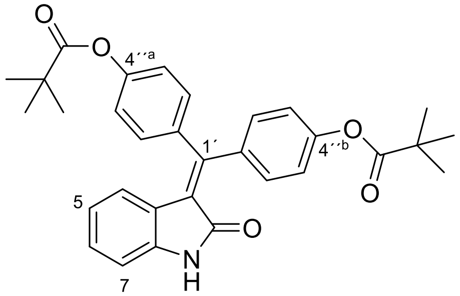

7k
$C_{31}H_{31}NO_5$
M = 497.22 g/mol

Since the synthesis *via* **GP-F** failed for this substrate, **7k** was synthesized photochemically by direct irradiation (λ = 368 nm, LED). 25.0 mg of oxetane *rac*-**5k** (47.4 µmmol, 1.00 equiv.) were dissolved in 19 mL of TFT and degassed (3 × freeze pump thaw). The clear solution was cooled to –25 °C and irradiated for 16 h. After complete conversion, all volatiles were removed under reduced pressure and the crude material was purified by flash column chromatography. 18.6 mg of **7k** (37.4 µmol, 79%) were isolated as a bright yellow solid.

^{1}H-NMR (500 MHz, DMSO-d_6, 300 K) δ [ppm] = 10.53 (s, 1H, NH), 7.36 – 7.30 (m, 4H, CH-3´´^{a}/CH-3´´^{b}), 7.27 – 7.22 (m, 2H, CH-2´´^{a}), 7.12 – 7.06 (m, 3H, CH-2´´^{a}/CH-6), 6.78 (d, ^3J = 7.7 Hz, 1H, CH-7), 6.62 (*virt.* td, $^3J \approx {}^3J$ = 7.7 Hz, ^4J = 1.1 Hz, 1H, CH-5), 6.19 (d, ^3J = 7.7 Hz, 1H, CH-4), 1.32 (s, 9H, $CH_3{}^a$)*, 1.31 (s, 9H, $CH_3{}^b$)*.

Signals marked with * could not be assigned and can be interchanged.

^{13}C-NMR (125 MHz, DMSO-d_6, 300 K) δ [ppm] = 176.7 (O*C*O^{t}Bu^a)*, 176.6 (O*C*O^{t}Bu^b)*, 167.5 (CONH), 151.7 (C4´´^{a})**, 151.5 (C4´´^{b})**, 151.3 (C1´), 142.5 (C7a), 138.0 (C1´´^{a}), 137.4 (C1´´^{b}), 131.8 (2C, C3´´^{b}), 130.6 (2C, C3´´^{a}), 129.6 (C6), 125.6 (C3a), 123.7 (C3), 123.1 (2C, C2´´^{a}), 123.0 (2C, C2´´^{b}), 121.0 (C5), 110.0 (C7), 39.1 [*C*$(CH_3)_3{}^a$]***, 39.1 [*C*$(CH_3)_3{}^b$]***, 27.2 [6C, C(*C*$H_3)_3$].

Signals marked with *, **, *** could not be assigned and can be interchanged.

HRMS (ESI+) *m/z*: $[M+H]^+$ Calculated: 498.2275; Found: 498.2283

IR (ATR): $\tilde{\nu}$ [cm^{-1}] = 3169 (w, NH), 3092 (w, $CH_{arom.}$), 3035 (w, $CH_{arom.}$), 2970 (w, C-$H_{alip.}$), 1743 (s, C=O), 1722 (s, C=O), 1624 (m), 1510 (m), 1468 (m), 1202 (m), 1112 (s), 748 (s).

Chiral HPLC: AD-H 250 × 4.6 mm, *n*-Hep/^{i}PrOH = 90/10, 1 mL/min, λ = 210 nm, t_R = 10.5 min (**7k**).

3-(Bis(4-fluorophenyl)methylene)-5-fluoroindolin-2-one (7l)

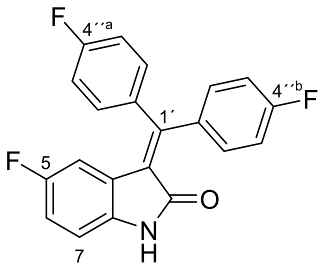

7l
$C_{21}H_{12}F_3NO$
M = 351.33 g/mol

Olefin **7l** was synthesized according to **GP-F** from 5-fluoroindolin-2-one **S6b** (200 mg, 1.32 mmol, 1.00 equiv.) together with commercially available bis(4-fluorophenyl)methanone (346 mg, 1.59 mmol, 1.20 equiv.). After purification 339 mg **7l** (965 μmol, 73%) were obtained as a red solid.

TLC: 0.34 (CH_2Cl_2:Ac = 97.5:2.5) [UV], [$KMnO_4$]

M.p.: 232 °C

^{1}H-NMR (500 MHz, $CDCl_3$, 300 K) δ [ppm] = 8.99 (s, 1H, NH), 7.36 – 7.27 (m, 4H, CH-2´´^{a}/CH-2´´^{b}), 7.19 – 7.14 (m, 2H, CH-3´´^{a}), 7.08 – 7.03 (m, 2H, CH-3´´^{b}), 6.79 (*virt.* td, $^3J_{HH}$ ≈ $^3J_{HF}$ = 8.7 Hz, $^4J_{HH}$ = 2.5 Hz, 1H, CH-6), 6.51 (dd, $^3J_{HH}$ = 8.7 Hz, $^4J_{HF}$ = 4.5Hz, 1H, CH-7), 6.11 (dd, $^3J_{HF}$ = 8.7 Hz, $^4J_{HH}$ = 2.5 Hz, 1H, CH-4).

^{13}**C-NMR** (125 MHz, $CDCl_3$, 300 K) δ [ppm] = 168.8 (CO), 164.2 (d, $^1J_{CF}$ = 250 Hz, C4´´^{a})*, 163.5 (d, $^1J_{CF}$ = 250 Hz, C4´´^{b})*, 158.2 (d, $^1J_{CF}$ = 237 Hz, C5), 154.1 (C1´). 136.9 (d, $^4J_{CF}$ = 2.0 Hz, C7a), 136.6 (d, $^4J_{CF}$ = 3.4 Hz, C1´´^{a}), 135.2 (d, $^4J_{CF}$ = 3.0 Hz, C1´´^{b}), 133.1 (d, $^3J_{CF}$ = 8.5 Hz, 2C, C2´´^{b}), 131.9 (d, $^3J_{CF}$ = 8.5 Hz, 2C, C2´´^{a}), 125.0 (d, $^3J_{CF}$ = 8.5 Hz, C3a), 124.8 (d, $^4J_{CF}$ = 3.0 Hz, C3), 116.6 (d, $^2J_{CF}$ = 21.6 Hz, 2C, C3´´^{a}), 115.5 (d, $^2J_{CF}$ = 24.1 Hz, 2C, C3´´^{b}), 115.2 (116.6 (d, $^2J_{CF}$ = 22.1 Hz, C6), 110.5 (d, $^2J_{CF}$ = 18.6 Hz, C4), 110.3 (C7).

Signals marked with * could not be assigned and can be interchanged.

^{19}**F-NMR** (470 MHz, $CDCl_3$, 300 K) δ [ppm] = –109.8, –110.3, –121.3.

HRMS (ESI+) *m/z*: $[M+H]^+$ Calculated: 352.0944; Found: 352.0949

IR (ATR): $\tilde{\nu}$ [cm^{-1}] = 3217 (w, NH.), 3086 (w, $CH_{arom.}$) 3006 (w, $CH_{arom.}$), 2990 (w, C-$H_{alip.}$), 2868 (w), 1693 (s, C=O), 1597 (m), 1504 (s, C=C), 1477 (s), 1228 (m), 1157 (m), 1095 (m), 837 (m), 749 (s).

Chiral HPLC: AD-H 250 × 4.6 mm, *n*-Hep/^{i}PrOH = 90/10, 1 mL/min, λ = 210 nm, t_R = 11.9 min (**7l**).

3-(Di-*p*-tolylmethylene)-5-fluoroindolin-2-one (7m)

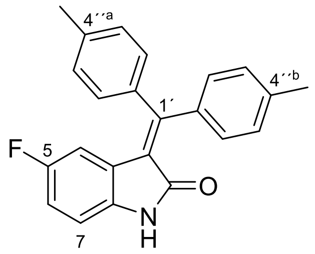

7m
$C_{23}H_{18}FNO$
M = 343.40 g/mol

Olefin **7m** was synthesized according to **GP-F** from 5-fluoroindolin-2-one **S6b** (200 mg, 1.32 mmol, 1.00 equiv.) together with commercially available di-*p*-tolylmethanone (334 mg, 1.59 mmol, 1.20 equiv.). After purification 405 mg **7m** (1.18 mmol, 89%) were obtained as an orange solid.

TLC: 0.37 (CH_2Cl_2:Ac = 97.5:2.5) [UV], [$KMnO_4$]

M.p.: 292 °C decomp.

^{1}H-NMR (500 MHz, $CDCl_3$, 300 K) δ [ppm] = 8.08 (s, 1H, NH), 7.26 – 7.22 (m, 4H, CH-3´´^{a}/CH-3´´^{b}), 7.19 – 7.14 (m, 4H, CH-2´´^{a}/CH-2´´^{b}), 6.77 (*virt.* td, $^3J_{HH} \approx {}^3J_{HF}$ = 8.8 Hz, $^4J_{HH}$ = 2.6 Hz, 1H, CH-6), 6.60 (dd, $^3J_{HH}$ = 8.8 Hz, $^4J_{HF}$ = 4.5 Hz, 1H, CH-7), 6.16 (dd, $^3J_{HF}$ = 8.8 Hz, $^4J_{HH}$ = 2.6 Hz, 1H, CH-4), 2.44 (s, 3H, $CH_3{}^a$)*, 2.38 (s, 3H, $CH_3{}^b$)*.

Signals marked with * could not be assigned and can be interchanged.

^{13}C-NMR (125 MHz, $CDCl_3$, 300 K) δ [ppm] = 168.5 (CO), 157.8 (d, $^1J_{CF}$ = 236 Hz, C5), 157.4 (C1´), 140.1 (C4´´^{a})*, 140.1 (C4´´^{b})*, 138.0 (C1´´^{a})**, 136.7 (C1´´^{b})**, 136.2 (d, $^4J_{CF}$ = 2.0 Hz, C7a), 130.3 (2C, C3´´^{a})***, 129.7 (2C, C3´´^{b})***, 129.7 (2C, C2´´^{a})****, 128.6 (2C, C2´´^{b})****, 125.7 (d, $^3J_{CF}$ = 9.0 Hz, C3a), 123.4 (d, $^4J_{CF}$ = 3.0 Hz, C3), 114.6 (d, $^2J_{CF}$ = 24.6 Hz, C6), 110.4 (d, $^2J_{CF}$ = 26.1 Hz, C4), 109.6 (d, $^3J_{CF}$ = 8.0 Hz), 21.6 ($CH_3{}^a$)*****, 21.6 ($CH_3{}^b$)*****.

Signals marked with *, **, ***, **** and ***** could not be assigned and can be interchanged.

^{19}F-NMR (470 MHz, DMSO-d_6, 300 K) δ [ppm] = –122.9.

HRMS (ESI+) *m/z*: $[M+H]^+$ Calculated: 344.1445; Found: 344.1442

IR (ATR): $\tilde{\nu}$ [cm^{-1}] = 3189 (w, NH), 3127 (w, $CH_{arom.}$), 3070 (w, $CH_{arom.}$), 2948 (w, C-$H_{alip.}$), 1688 (s, C=O) 1578 (m), 1466 (s), 1290 (m), 1261 (m), 802 (m), 750 (s).

Chiral HPLC: AD-H 250 × 4.6 mm, *n*-Hep/^{i}PrOH = 90/10, 1 mL/min, λ = 210 nm, t_R = 10.1 min (**7m**).

3-(Bis(4-chlorophenyl)methylene)-5-fluoroindolin-2-one (7n)

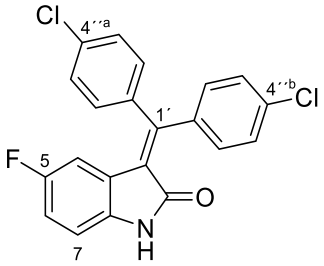

7n
$C_{21}H_{12}Cl_2FNO$
M = 383.03 g/mol

Olefin **7n** was synthesized according to **GP-F** from 5-fluoroindolin-2-one **S6b** (200 mg, 1.32 mmol, 1.00 equiv.) together with commercially available bis(4-chlorophenyl)methanone (399 mg, 1.59 mmol, 1.20 equiv.). After purification 325 mg **7n** (846µmol, 64%) were obtained as a yellow solid.

TLC: 0.40 (CH_2Cl_2:Ac = 97.5:2.5) [UV], [$KMnO_4$]

M.p.: 243 °C decomp.

^{1}**H-NMR** (500 MHz, DMSO-d_6, 300 K) δ [ppm] = 10.58 (s, 1H, NH), 7.63 – 7.59 (m, 2H, CH-3´´^{a}), 7.42 – 7.39 (m, 2H, CH-3´´^{b}), 7.38 – 7.34 (m, 2H, CH-2´´^{a}), 7.33 – 7.29 (m, 2H, CH-2´´^{b}), 6.99 (*virt.* td, $^{3}J_{HH} \approx {}^{3}J_{HF}$ = 9.5 Hz, $^{4}J_{HH}$ = 2.5 Hz, 1H, CH-6), 6.77 (dd, $^{3}J_{HH}$ = 9.5 Hz, $^{4}J_{HF}$ = 4.7 Hz, 1H, CH-6), 5.84 (dd, $^{3}J_{HF}$ = 9.5 Hz, $^{4}J_{HH}$ = 2.5 Hz, 1H, CH-4).

^{13}**C-NMR** (125 MHz, $CDCl_3$, 300 K) δ [ppm] = 167.8 (CO), 158.1 (d, $^{1}J_{CF}$ = 238 Hz, C5), 153.4 (C1´), 138.6 (C4´´^{a}), 137.3 (C4´´^{b}), 136.5 (C7a), 136.3 (C1´´^{a}), 136.0 (C1´´^{b}), 132.0 (2C, C3´´^{a}), 131.0 (2C, C3´´^{b}), 129.6 (C2´´^{a}), 128.3 (C2´´^{b}), 124.7 (d, $^{4}J_{CF}$ = 3.0 Hz, C3), 124.6 (d, $^{3}J_{CF}$ = 9.0 Hz, C3a), 115.8 (d, $^{2}J_{CF}$ = 24.6 Hz, C6), 110.7 (d, $^{2}J_{CF}$ = 26.6 Hz, C4), 110.0 (d, $^{3}J_{CF}$ = 9.0 Hz, C7).

^{19}**F-NMR** (470 MHz, DMSO-d_6, 300 K) δ [ppm] = –122.5.

HRMS (ESI+) *m/z*: $[M+H]^+$ Calculated: 384.0353; Found: 384.0356

IR (ATR): $\tilde{\nu}$ [cm^{-1}] = 3186 (w, NH), 3087 (w, $CH_{arom.}$), 3006 (w, $CH_{arom.}$), 2990 (w, C-$H_{alip.}$), 2863 (w, C-$H_{alip.}$) 1694 (s, C=O) 1587 (m), 1476 (s), 1089 (s), 1013 (m), 764 (s), 785 (s), 750 (s).

Chiral HPLC: AD-H 250 × 4.6 mm, *n*-Hep/^{i}PrOH = 70/30, 1 mL/min, λ = 210 nm, t_R = 6.61 min (**7n**).

3-(Bis(4-chlorophenyl)methylene)-5-chloroindolin-2-one (7o)

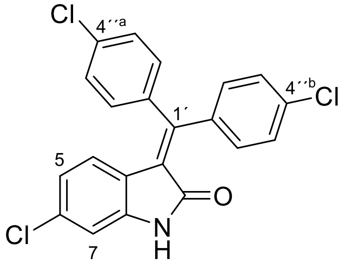

7o
$C_{21}H_{12}Cl_3NO$
M = 400.68 g/mol

Olefin **7o** was synthesized according to **GP-F** from 6-chloroindolin-2-one **S6c** (200 mg, 1.19 mmol, 1.00 equiv.) together with commercially available bis(4-chlorophenyl)methanone (360 mg, 1.43 mmol, 1.20 equiv.). After purification 445 mg **7o** (1.11mmol, 64%) were obtained as a bright yellow solid.

TLC: 0.50 (CH_2Cl_2:Ac = 97.5:2.5) [UV], [$KMnO_4$]

M.p.: 275 °C decomp.

^{1}H-NMR (500 MHz, DMSO-d_6, 300 K) δ [ppm] = 10.70 (s, 1H, NH), 7.59 – 7.55 (m, 2H, CH-3´´^{a}), 7.42 – 7.38 (m, 2H, CH-3´´^{b}), 7.35 – 7.32 (m, 2H, CH-2´´^{a}), 7.30 – 7.27 (m, 2H, CH-2´´^{b}), 6.80 (d, ^4J = 2.0 Hz, 1H, CH-7), 6.75 (dd, ^3J = 8.3 Hz, 1H, CH-5), 6.22 (d, ^3J = 8.2 Hz, 1H, CH-4).

^{13}C-NMR (125 MHz, $CDCl_3$, 300 K) δ [ppm] = 168.3 (CO), 152.6 (C1´), 141.7 (C7a), 139.1 (C4´´^{a}), 137.5 (C4´´^{b}), 136.1 (C1´´^{a}), 136.1 (C1´´^{b}), 132.1 (2C, C2´´^{b}), 131.3 (2C, C2´´^{a}), 129.6 (C3´´^{a}), 128.4 (C3´´^{b}), 124.4 (C3), 124.2 (C4), 121.1 (C3a), 121.7 (C5), 110.4 (C7).

HRMS (ESI+) *m/z*: $[M+H]^+$ Calculated: 400.0057; Found: 400.0063

IR (ATR): $\tilde{\nu}$ [cm^{-1}] = 3178 (w, NH), 3120 (w, $CH_{arom.}$), 3047 (w, $CH_{arom.}$), 2982 (w, C-$H_{alip.}$), 2870 (w, C-$H_{alip.}$) 1692 (s, C=O) 1590 (s), 1487 (m), 1433 (m), 1266 (m), 1087 (s), 811 (s), 765 (s), 741 (s).

Chiral HPLC: AD-H 250 × 4.6 mm, *n*-Hep/^{i}PrOH = 90/10, 1 mL/min, λ = 210 nm, t_R = 22.9 min (**7o**).

Photocatalytic Kinetic Resolution Experiments:

(*R*)-3',3'-Bis(4-chlorophenyl)spiro[indoline-3,2'-oxetan]-2-one (5a)

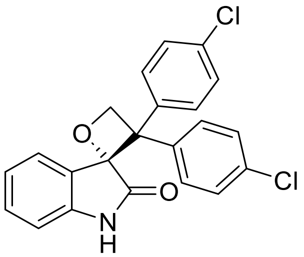

5a
$C_{22}H_{15}Cl_2NO_2$
M = 396.27 g/mol

According to **GP-E** the enantiomerically enriched oxetane **5a** was synthesized by photocatalytic kinetic resolution of *rac*-**5a** (19.8 mg, 50.0 µmol, 1.00 equiv.) by thioxanthone catalyst **6** (1.08 mg, 2.50 µmol, 5.00 mol%) in 20 mL TFT (c = 2.50 mM). After irradiation for 30 min at –25 °C with a blue LED (λ_{max} = 398 nm). The product mixture was purified using flash column chromatography (CH_2Cl_2:Ac = 100:0 → 90:10). This yields oxetane **5a** (8.20 mg, 20.7 µmol, 41%, 99% *ee*, *s* = 29), fragmentation product **7a** (3.18 mg, 8.68 µmol, 17%), isatin **10a** (2.80 mg, 18.9 µmol, 38%) and olefin **9a** (4.80 mg, 19.3 µmol, 39%).

<u>1.00 mmol scale:</u>

To prove synthetic relevance of this kinetic resolution, the reaction was scaled up and carried out on a 1.00 mmol scale. Therefore, oxetane *rac*-**5a** (400 mg, 1.01 mmol, 1.00 equiv.) and thioxanthone catalyst **6** (21.8 mg, 50.5 µmol, 5.00 mol%) were dissolved in 404 mL of TFT in a 500 mL round bottom flask and degassed by sparging the solution with argon while ultrasonication in cold water. The reaction mixture was irradiated using the same LED (λ_{max} = 398 nm) as described above in **GP-E**. The clear reaction solution was cooled to –25 °C and irradiated for 18 h. All volatiles were removed under reduced pressure and the crude material was purified by column chromatography (CH_2Cl_2:Ac = 100:0 → 90:10). After separation of olefin **9a** and isatin **10a** the mixed fractions of **5a** and **7a** were combined and an automated flash column chromatography with a flatter gradient over 20 column volumes (CV) was performed. This yielded the enantiopure oxetane **5a** (165 mg, 416 µmol, 41%, 98% *ee*, *s* = 24) as a white powder,

fragmentation product **7a** (46.1 mg, 126 µmol, 12%) as a bright yellow solid, isatin **10a** (55.0 mg, 373 µmol, 37%) as a red powder and olefin **9a** (91.2 mg, 366 µmol, 36%) as a colorless solid. For this large-scale reaction, the effort was undertaken to reisolate enantiopure thioxanthone **6** (21.3 mg, 49.5 µmol, 98%)
All analytic data matched those for the previously described compounds.

Optical Rotation: $[\alpha]_D^{25}$ = 10 (c = 1.0, $CHCl_3$) [99% *ee*].

Chiral HPLC: AD-H 250 × 4.6 mm, *n*-Hep/^{i}PrOH = 90/10, 1 mL/min, λ = 210 nm, t_R = 17.7 min (major, **5a**), 45.8 (minor, *ent*-**5a**).

(*R*)-3',3'-Di-*p*-tolylspiro[indoline-3,2'-oxetan]-2-one (5b)

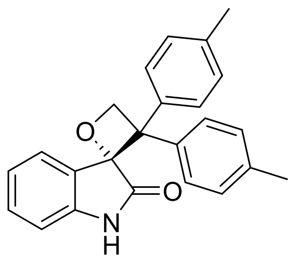

5b
$C_{24}H_{21}NO_2$
M = 355.44 g/mol

According to **GP-E** the enantiomerically enriched oxetane **5b** was synthesized by photocatalytic kinetic resolution of *rac*-**5b** (17.8 mg, 50.0 µmol, 1.00 equiv.) by thioxanthone catalyst **6** (1.08 mg, 2.50 µmol, 5.00 mol%) in 20 mL TFT (c = 2.50 mM). After irradiation for 30 min at –25 °C with an LED (λ_{max} = 398 nm). The product mixture was purified using flash column chromatography (CH_2Cl_2:Ac = 100:0 → 90:10). This yields oxetane **5b** (8.82 mg, 24.8 µmol, 50%, 98% *ee*, *s* > 100), fragmentation product **7b** (1.28 mg, 3.93 µmol, 8%), isatin **10a** (3.06 mg, 20.8 µmol, 42%) and olefin **9b** (4.09 mg, 19.4 µmol, 39%).

Optical Rotation: $[\alpha]_D^{25}$ = 4 (c = 1.0, $CHCl_3$) [98% *ee*].

Chiral HPLC: OD-RH 250 × 4.6 mm, H_2O/MeCN = 80/20 → 0/100, 1 mL/min, λ = 210 nm, t_R = 20.9 min (major, **5b**), 21.9 (minor, *ent*-**5b**).

(*R*)-3',3'-Bis(4-(*tert*-butyl)phenyl)spiro[indoline-3,2'-oxetan]-2-one (5c)

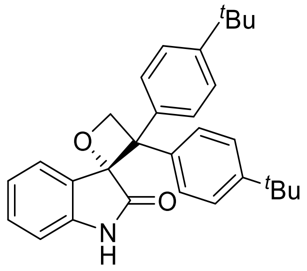

5c
$C_{30}H_{33}NO_2$
M = 439.60 g/mol

According to **GP-E** the enantiomerically enriched oxetane **5c** was synthesized by photocatalytic kinetic resolution of *rac*-**5c** (22.0 mg, 50.0 µmol, 1.00 equiv.) by thioxanthone catalyst **6** (1.08 mg, 2.50 µmol, 5.00 mol%) in 20 mL TFT (c = 2.50 mM). After irradiation for 30 min at –25 °C with an LED (λ_{max} = 398 nm). The product mixture was purified using flash column chromatography (CH_2Cl_2:Ac = 100:0 → 90:10). This yields oxetane **5c** (9.62 mg, 21.9 µmol, 44%, 93% *ee*, *s* = 21), fragmentation product **7c** (1.86 mg, 4.54 µmol, 9%), isatin **10a** (3.40 mg, 23.1 µmol, 46%) and olefin **9c** (6.05 mg, 20.7 µmol, 41%).

Optical Rotation: $[a]_D^{25}$ = 18 (c = 1.0, $CHCl_3$) [93% *ee*].

Chiral HPLC: AD-H 250 × 4.6 mm, *n*-Hep/^{i}PrOH = 90/10, 1 mL/min, λ = 210 nm, t_R = 5.8 min (major, **5c**), 24.2 (minor, *ent*-**5c**).

(*R*)-3',3'-Bis(3,5-di-*tert*-butylphenyl)spiro[indoline-3,2'-oxetan]-2-one (5d)

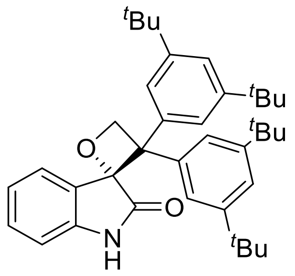

5d
$C_{38}H_{49}NO_2$
M = 551.82 g/mol

According to **GP-E** the enantiomerically enriched oxetane **5d** was synthesized by photocatalytic kinetic resolution of *rac*-**5d** (27.6 mg, 50.0 µmol, 1.00 equiv.) by thioxanthone catalyst **6** (1.08 mg, 2.50 µmol, 5.00 mol%) in 20 mL TFT (c = 2.50 mM). After irradiation for 30 min at –25 °C with an LED (λ_{max} = 398 nm). The product mixture was purified using flash column chromatography (CH_2Cl_2:Ac = 100:0 → 90:10). This yields oxetane **5d** (10.2 mg, 18.5 µmol, 37%, 98% *ee*, *s* = 17), fragmentation product **7d** (0.29 mg, 0.15 µmol, 1%), isatin **10a** (4.50 mg, 30.6 µmol, 61%) and olefin **9d** (11.8 mg, 29.1 µmol, 58%).

Optical Rotation: $[\alpha]_D^{25}$ = 24 (c = 1.0, $CHCl_3$) [98% *ee*].

Chiral HPLC: OD-RH 250 × 4.6 mm, H_2O/MeCN = 80/20 → 0/100, 1 mL/min, λ = 210 nm, t_R = 23.9 min (major, **5d**), 24.6 (minor, *ent*-**5d**).

(*R*)-3',3'-Diphenylspiro[indoline-3,2'-oxetan]-2-one (5e)

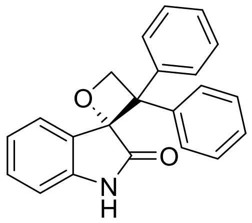

5e
$C_{22}H_{17}NO_2$
M = 327.38 g/mol

According to **GP-E** the enantiomerically enriched oxetane **5e** was synthesized by photocatalytic kinetic resolution of *rac*-**5e** (16.4 mg, 50.0 µmol, 1.00 equiv.) by thioxanthone catalyst **6** (1.08 mg, 2.50 µmol, 5.00 mol%) in 20 mL TFT (c = 2.50 mM). After irradiation for 30 min at –25 °C with an LED (λ_{max} = 398 nm). The product mixture was purified using flash column chromatography (CH_2Cl_2:Ac = 100:0 → 90:10). This yields oxetane **5e** (8.02 mg, 24.5 µmol, 49%, 99% *ee*, *s* > 100), fragmentation product **7e** (2.03 mg, 6.83 µmol, 14%), isatin **10a** (2.59 mg, 17.6 µmol, 35%) and olefin **9e** (2.95 mg, 16.4 µmol, 33%).

Optical Rotation: $[\alpha]_D^{25}$ = 8 (c = 1.0, $CHCl_3$) [99% *ee*].

Chiral HPLC: AD-H 250 × 4.6 mm, *n*-Hep/^{i}PrOH = 90/10, 1 mL/min, λ = 210 nm, t_R = 16.4 min (major, **5e**), 43.7 (minor, *ent*-**5e**).

(*R*)-3',3'-Bis(4-fluorophenyl)spiro[indoline-3,2'-oxetan]-2-one (5f)

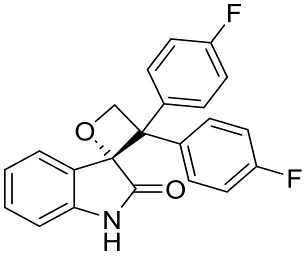

5f
$C_{22}H_{15}F_2NO_2$
M = 363.36 g/mol

According to **GP-E** the enantiomerically enriched oxetane **5f** was synthesized by photocatalytic kinetic resolution of *rac*-**5f** (18.2 mg, 50.0 µmol, 1.00 equiv.) by thioxanthone catalyst **6** (1.08 mg, 2.50 µmol, 5.00 mol%) in 20 mL TFT (c = 2.50 mM). After irradiation for 30 min at –25 °C with an LED (λ_{max} = 398 nm). The product mixture was purified using flash column chromatography (CH_2Cl_2:Ac = 100:0 → 90:10). This yields oxetane **5f** (8.32 mg, 22.9 µmol, 46%, 99% *ee*, *s* = 50), fragmentation product **7f** (1.67 mg, 5.01 µmol, 10%), isatin **10a** (3.06 mg, 20.8 µmol, 42%) and olefin **9f** (4.25 mg, 19.6 µmol, 39%).

Optical Rotation: $[\alpha]_D^{25}$ = 8 (c = 1.0, $CHCl_3$) [99% *ee*].

Chiral HPLC: AD-H 250 × 4.6 mm, *n*-Hep/^{i}PrOH = 70/30, 1 mL/min, λ = 210 nm, t_R = 7.0 min (major, **5f**), 9.3 (minor, *ent*-**5f**).

(*R*)-3',3'-Bis(4-bromophenyl)spiro[indoline-3,2'-oxetan]-2-one (5g)

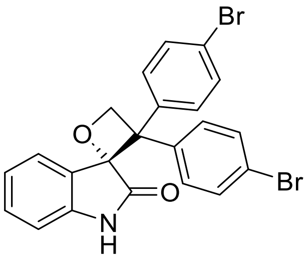

5g
$C_{22}H_{15}Br_2NO_2$
M = 485.18 g/mol

According to **GP-E** the enantiomerically enriched oxetane **5g** was synthesized by photocatalytic kinetic resolution of *rac*-**5g** (24.3 mg, 50.0 µmol, 1.00 equiv.) by thioxanthone catalyst **6** (1.08 mg, 2.50 µmol, 5.00 mol%) in 20 mL TFT (c = 2.50 mM). After irradiation for 30 min at –25 °C with an LED (λ_{max} = 398 nm). The product mixture was purified using flash column chromatography (CH_2Cl_2:Ac = 100:0 → 90:10). This yields oxetane **5g** (11.3 mg, 23.3 µmol, 47%, 96% *ee*, *s* = 40), fragmentation product **7f** (1.15 mg, 2.53 µmol, 5%), isatin **10a** (3.60 mg, 24.5 µmol, 49%) and olefin **9f** (7.58 mg, 22.4 µmol, 45%).

Optical Rotation: $[a]_D^{25}$ = –14 (c = 1.0, $CHCl_3$) [96% *ee*].

Chiral HPLC: AD-H 250 × 4.6 mm, *n*-Hep/^{i}PrOH = 70/30, 1 mL/min, λ = 210 nm, t_R = 7.3 min (major, **5g**), 19.0 (minor, *ent*-**5g**).

(*R*)-3',3'-Bis(3,5-bis(trifluoromethyl)phenyl)spiro[indoline-3,2'-oxetan]-2-one (5h)

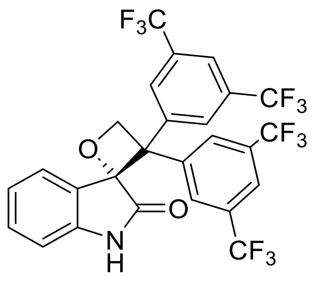

5h

$C_{26}H_{13}F_{12}NO_2$

M = 599.38 g/mol

According to **GP-E** the enantiomerically enriched oxetane **5h** was synthesized by photocatalytic kinetic resolution of *rac*-**5h** (30.0 mg, 50.0 µmol, 1.00 equiv.) by thioxanthone catalyst **6** (1.08 mg, 2.50 µmol, 5.00 mol%) in 20 mL TFT (c = 2.50 mM). After irradiation for 30 min at –25 °C with an LED (λ_{max} = 398 nm). The product mixture was purified using flash column chromatography (CH_2Cl_2:Ac = 100:0 → 90:10). This yields oxetane **5h** (11.4 mg, 19.0 µmol, 38%, 98% *ee*, *s* = 18), fragmentation product **7h** (1.19 mg, 2.09 µmol, 4%), isatin **10a** (4.10 mg, 27.9 µmol, 56%) and olefin **9h** (11.5 mg, 25.5 µmol, 51%).

Optical Rotation: $[a]_D^{25}$ = –6 (c = 1.0, $CHCl_3$) [98% *ee*].

Chiral HPLC: OD-RH 250 × 4.6 mm, H_2O/MeCN = 80/20 → 0/100, 1 mL/min, λ = 210 nm, t_R = 19.4 min (major, **5h**), 20.0 (minor, *ent*-**5h**).

(*R*)-3',3'-Bis(3-nitrophenyl)spiro[indoline-3,2'-oxetan]-2-one (5i)

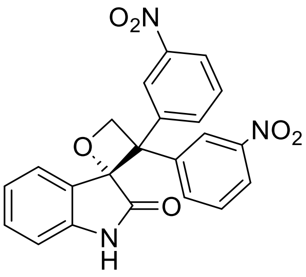

5i
$C_{22}H_{15}N_3O_6$
M = 417.38 g/mol

According to **GP-E** the enantiomerically enriched oxetane **5i** was synthesized by photocatalytic kinetic resolution of *rac*-**5i** (20.9 mg, 50.0 µmol, 1.00 equiv.) by thioxanthone catalyst **6** (1.08 mg, 2.50 µmol, 5.00 mol%) in 20 mL TFT (c = 2.50 mM). After irradiation for 30 min at –25 °C with an LED (λ_{max} = 398 nm). The product mixture was purified using flash column chromatography (CH_2Cl_2:Ac = 100:0 → 90:10). This yields oxetane **5i** (10.1 mg, 24.3 µmol, 48%, 94% *ee*, *s* = 50), fragmentation product **7i** (0.84 mg, 2.17 µmol, 4%), isatin **10a** (3.25 mg, 22.1 µmol, 44%) and olefin **9i** (5.31 mg, 19.7 µmol, 39%).

Optical Rotation: $[\alpha]_D^{25}$ = 34 (c = 1.0, $CHCl_3$) [94% *ee*].

Chiral HPLC: OD-RH 250 × 4.6 mm, H_2O/MeCN = 80/20 → 0/100, 1 mL/min, λ = 210 nm, t_R = 18.1 min (major, **5i**), 19.3 (minor, *ent*-**5i**).

(*R*)-(2-Oxospiro[indoline-3,2'-oxetane]-3',3'-diyl)bis(4,1-phenylene) bis(4-methylbenzenesulfonate) (5j)

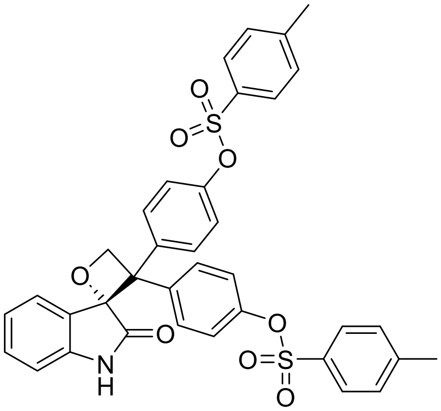

5j
$C_{36}H_{29}NO_8S_2$
M = 667.75 g/mol

According to **GP-E** the enantiomerically enriched oxetane **5j** was synthesized by photocatalytic kinetic resolution of *rac*-**5j** (33.4 mg, 50.0 µmol, 1.00 equiv.) by thioxanthone catalyst **6** (1.08 mg, 2.50 µmol, 5.00 mol%) in 20 mL TFT (c = 2.50 mM). After irradiation for 30 min at –25 °C with an LED (λ_{max} = 398 nm). The product mixture was purified using flash column chromatography (CH_2Cl_2:Ac = 100:0 → 90:10). This yields oxetane **5j** (13.5 mg, 20.1 µmol, 40%, 98% *ee*, *s* = 21), fragmentation product **7j** (8.35 mg, 13.1 µmol, 26%), isatin **10a** (2.41 mg, 16.4 µmol, 32%) and olefin **9j** (8.58 mg, 16.5 µmol, 33%).

Optical Rotation: $[\alpha]_D^{25}$ = 36 (c = 1.0, $CHCl_3$) [98% *ee*].

Chiral HPLC: OD-RH 250 × 4.6 mm, H_2O/MeCN = 80/20 → 0/100, 1 mL/min, λ = 210 nm, t_R = 24.5 min (major, **5j**), 26.3 (minor, *ent*-**5j**).

(*R*)-(2-Oxospiro[indoline-3,2'-oxetane]-3',3'-diyl)bis(4,1-phenylene) bis(2,2-dimethyl-propanoate) (5k)

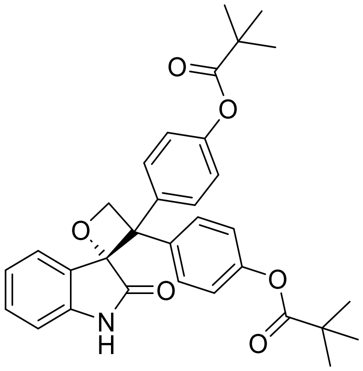

5k
$C_{32}H_{33}NO_6$
M = 527.62 g/mol

According to **GP-E** the enantiomerically enriched oxetane **5k** was synthesized by photocatalytic kinetic resolution of *rac*-**5k** (26.4 mg, 50.0 µmol, 1.00 equiv.) by thioxanthone catalyst **6** (1.08 mg, 2.50 µmol, 5.00 mol%) in 20 mL TFT (c = 2.50 mM). After irradiation for 30 min at –25 °C with an LED (λ_{max} = 398 nm). The product mixture was purified using flash column chromatography (CH_2Cl_2:Ac = 100:0 → 90:10). This yields oxetane **5k** (11.2 mg, 21.3 µmol, 42%, 94% *ee*, *s* = 19), fragmentation product **7k** (1.58 mg, 3.18 µmol, 6%), isatin **10a** (3.70 mg, 25.2 µmol, 50%) and olefin **9k** (9.11 mg, 23.9 µmol, 48%).

Optical Rotation: $[a]_D^{25}$ = –18 (c = 1.0, $CHCl_3$) [94% *ee*].

Chiral HPLC: AD-H 250 × 4.6 mm, *n*-Hep/^{i}PrOH = 90/10, 1 mL/min, λ = 210 nm, t_R = 12 7 min (major, **5j**), 24.3 (minor, *ent*-**5j**).

(*R*)-5-Fluoro-3',3'-bis(4-fluorophenyl)spiro[indoline-3,2'-oxetan]-2-one (5l)

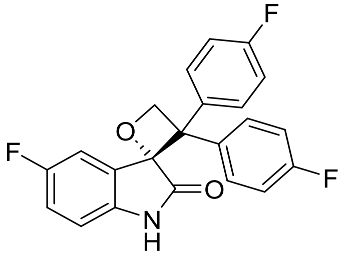

5l

$C_{22}H_{14}F_3NO_2$

M = 381.35 g/mol

According to **GP-E** the enantiomerically enriched oxetane **5l** was synthesized by photocatalytic kinetic resolution of *rac*-**5l** (19.1 mg, 50.0 µmol, 1.00 equiv.) by thioxanthone catalyst **6** (1.08 mg, 2.50 µmol, 5.00 mol%) in 20 mL TFT (c = 2.50 mM). After irradiation for 30 min at –25 °C with an LED (λ_{max} = 398 nm). The product mixture was purified using flash column chromatography (CH_2Cl_2:Ac = 100:0 → 90:10). This yields oxetane **5l** (8.42 mg, 22.1 µmol, 44%, 99% *ee*, *s* = 40), fragmentation product **7l** (5.84 mg, 16.6 µmol, 33%), isatin **10b** (1.60 mg, 9.69 µmol, 19%) and olefin **9l** (1.85 mg, 8.56 µmol, 17%).

Optical Rotation: $[a]_D^{25}$ = 20 (c = 1.0, $CHCl_3$) [99% *ee*].

Chiral HPLC: AD-H 250 × 4.6 mm, *n*-Hep/^{i}PrOH = 90/10, 1 mL/min, λ = 210 nm, t_R = 16.9 min (major, **5l**), 42.4 (minor, *ent*-**5l**).

(*R*)-5-Fluoro-3',3'-di-*p*-tolylspiro[indoline-3,2'-oxetan]-2-one (5m)

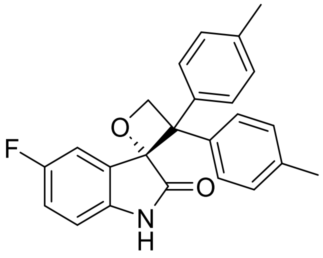

5m

$C_{24}H_{20}FNO_2$

M = 373.43 g/mol

According to **GP-E** the enantiomerically enriched oxetane **5m** was synthesized by photocatalytic kinetic resolution of *rac*-**5m** (18.7 mg, 50.0 µmol, 1.00 equiv.) by thioxanthone catalyst **6** (1.08 mg, 2.50 µmol, 5.00 mol%) in 20 mL TFT (c = 2.50 mM). After irradiation for 30 min at –25 °C with an LED (λ_{max} = 398 nm). The product mixture was purified using flash column chromatography (CH_2Cl_2:Ac = 100:0 → 90:10). This yields oxetane **5m** (7.96 mg, 21.3 µmol, 43%, 98% *ee, s* = 28), fragmentation product **7l** (4.54 mg, 13.2 µmol, 26%), isatin **10b** (2.04 mg, 12.4 µmol, 25%) and olefin **9m** (2.81 mg, 13.5 µmol, 27%).

Optical Rotation: $[a]_D^{25}$ = 36 (c = 1.0, $CHCl_3$) [98% *ee*].

Chiral HPLC: AD-H 250 × 4.6 mm, *n*-Hep/^{i}PrOH = 90/10, 1 mL/min, λ = 210 nm, t_R = 14.3 min (major, **5m**), 23.5 (minor, *ent*-**5m**).

(*R*)-3',3'-Bis(4-chlorophenyl)-5-fluorospiro[indoline-3,2'-oxetan]-2-one (5n)

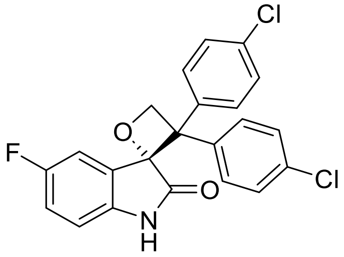

5n
$C_{24}H_{20}Cl_2FNO_2$
M = 414.26 g/mol

According to **GP-E** the enantiomerically enriched oxetane **5n** was synthesized by photocatalytic kinetic resolution of *rac*-**5n** (20.7 mg, 50.0 µmol, 1.00 equiv.) by thioxanthone catalyst **6** (1.08 mg, 2.50 µmol, 5.00 mol%) in 20 mL TFT (c = 2.50 mM). After irradiation for 30 min at –25 °C with an LED (λ_{max} = 398 nm). The product mixture was purified using flash column chromatography (CH_2Cl_2:Ac = 100:0 → 90:10). This yields oxetane **5n** (7.84 mg, 18.9 µmol, 38%, 99% *ee*, *s* = 21), fragmentation product **7n** (7.71 mg, 20.1 µmol, 40%), isatin **10b** (1.45 mg, 8.78 µmol, 18%) and olefin **9n** (2.51 mg, 10.1 µmol, 20%).

Optical Rotation: $[a]_D^{25}$ = 16 (c = 1.0, $CHCl_3$) [99% *ee*].

Chiral HPLC: AD-H 250 × 4.6 mm, *n*-Hep/^{i}PrOH = 70/30, 1 mL/min, λ = 210 nm, t_R = 6.9 min (major, **5n**), 17.5 (minor, *ent*-**5n**).

(*R*)-6-Chloro-3',3'-bis(4-chlorophenyl)spiro[indoline-3,2'-oxetan]-2-one (5o)

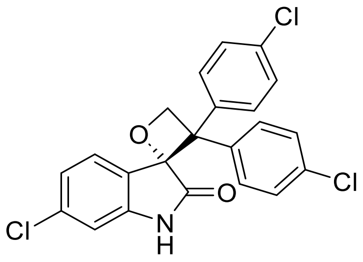

5o
$C_{22}H_{14}Cl_3NO_2$
M = 430.71 g/mol

According to **GP-E** the enantiomerically enriched oxetane **5o** was synthesized by photocatalytic kinetic resolution of *rac*-**5o** (21.5 mg, 50.0 µmol, 1.00 equiv.) by thioxanthone catalyst **6** (1.08 mg, 2.50 µmol, 5.00 mol%) in 20 mL TFT (c = 2.50 mM). After irradiation for 30 min at –25 °C with a blue LED (λ_{max} = 398 nm). The product mixture was purified using flash column chromatography (CH_2Cl_2:Ac = 100:0 → 90:10) This yields oxetane **5o** (8.08 mg, 18.8 µmol, 38%, 96% *ee, s* = 14), fragmentation product **7o** (5.26 mg, 13.1 µmol, 26%), isatin **10c** (2.34 mg, 12.9 µmol, 26%) and olefin **9o** (3.05 mg, 12.2 µmol, 25%).

Optical Rotation: $[a]_D^{25}$ = 10 (c = 1.0, $CHCl_3$) [96% *ee*].

Chiral HPLC: AD-H 250 × 4.6 mm, *n*-Hep/^{i}PrOH = 90/10, 1 mL/min, λ = 210 nm, t_R = 15.9 min (major, **5o**), 45.9 min (minor, *ent*-**5o**).

References for this section

(1) A. El-Faham, Z. Al Marhoon, A. Abdel-Megeed, F. Albericio, *Molecules* **2013**, *18*, 14747–14759.

(2) Holt *et al.*, *J. Chem. Soc.*, **1958**, 1217–1222.

(3) L. D. Azevedo, M. M. Bastos, F. C. Vasconcelos, L. V. B. Hoelz, F. P. S. Junior, R. F. Dantas, A. C. M. de Almeida, A. P. de Oliveira, L. C. Gomes, R. C. Maia et al., *Med Chem Res* **2017**, *26*, 2929–2941.

(4) C. Ebner, C. A. Müller, C. Markert, A. Pfaltz, *J. Am. Chem. Soc.* **2011**, *133*, 4710–4713.

(5) M. Gruber, K. Padberg, J. Min, A. R. Waterloo, F. Hampel, H. Maid, T. Ameri, C. J. Brabec, R. R. Tykwinski, *Chem. Eur. J.* **2017**, *23*, 17829–17835.

(6) J. A. Januszewski, D. Wendinger, C. D. Methfessel, F. Hampel, R. R. Tykwinski, *Angew. Chem. Int. Ed.* **2013**, *52*, 1817–1821.

(7) C. M. Abdelmalek, Z. Hu, T. Kronenberger, J. Küblbeck, F. J. M. Kinnen, S. S. Hesse, A. Malik, M. Kudolo, R. Niess, M. Gehringer et al., *J. Med. Chem.* **2022**, *65*, 4616–4632.

(8) C. Barrett, V. Krishnamurti, X. Ispizua-Rodriguez, Z. Zhu, C. J. Koch, G. K. Surya Prakash, *Org. Lett.* **2022**, *24*, 5417–5421.

(9) L. Cardinale, M. O. Konev, A. Jacobi von Wangelin, *Chem. Eur. J.* **2020**, *26*, 8239–8243.

(10) C. Huang, R.-N. Ci, J. Qiao, X.-Z. Wang, K. Feng, B. Chen, C.-H. Tung, L.-Z. Wu, *Angew. Chem. Int. Ed.* **2021**, *60*, 11779–11783.

(11) Z.-B. Luo, Y.-W. Wang, Y. Peng, *Org. Biomol. Chem.* **2020**, *18*, 2054–2057.

(12) S. Zhang, Z. Shen, H. Jian, *J. Org. Chem.* **2020**, *85*, 6143–6150.

(13) M. Takuya, I. Takayuki, K. Noboru, I. Hiizu, *J. Am. Chem. Soc.* **1992**, *114*, 9952–9959.

(14) A. J. Boutland, A. Carroll, C. Alvarez Lamsfus, A. Stasch, L. Maron, C. Jones, *J. Am. Chem. Soc.* **2017**, *139*, 18190–18193.

(15) C. Ling, M. Minato, P. M. Lahti, H. van Willigen, *J. Am. Chem. Soc.* **1992**, *114*, 9959–9969.

15. NMR-Spectra

3,5-Di-*tert*-butylbenzaldehyde (S2)

^{1}H-NMR (500 MHz, DMSO-d_6, 300K)

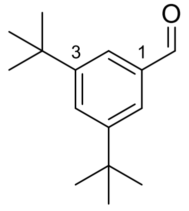

S2

$C_{15}H_{22}O$

M = 218.34g/mol

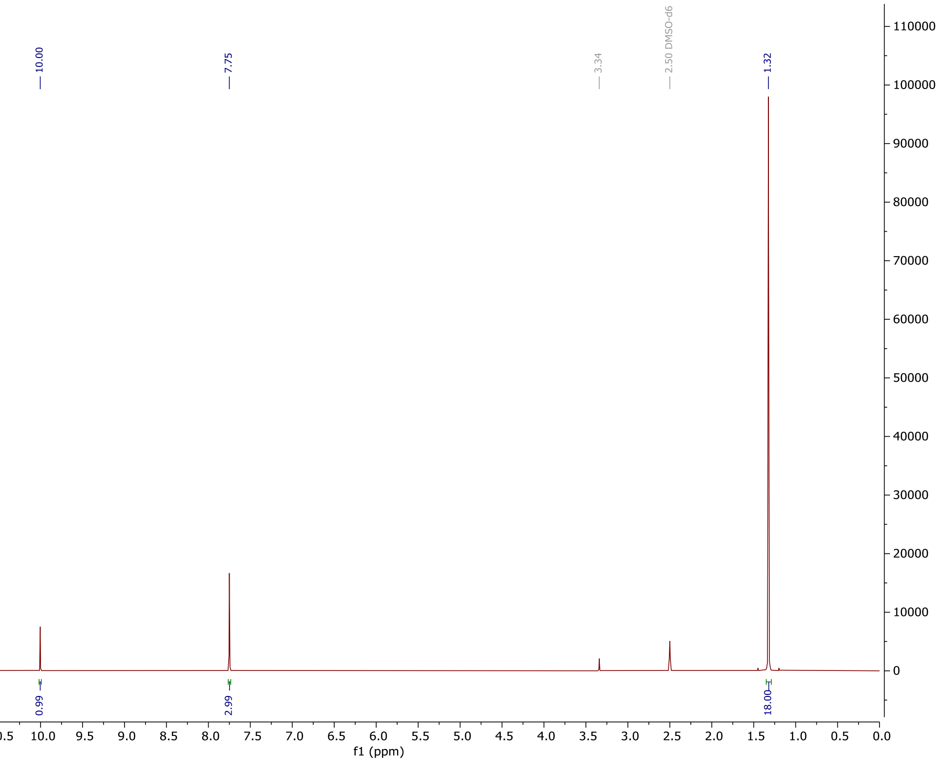

^{13}C-NMR (125 MHz, DMSO-d_6, 300K)

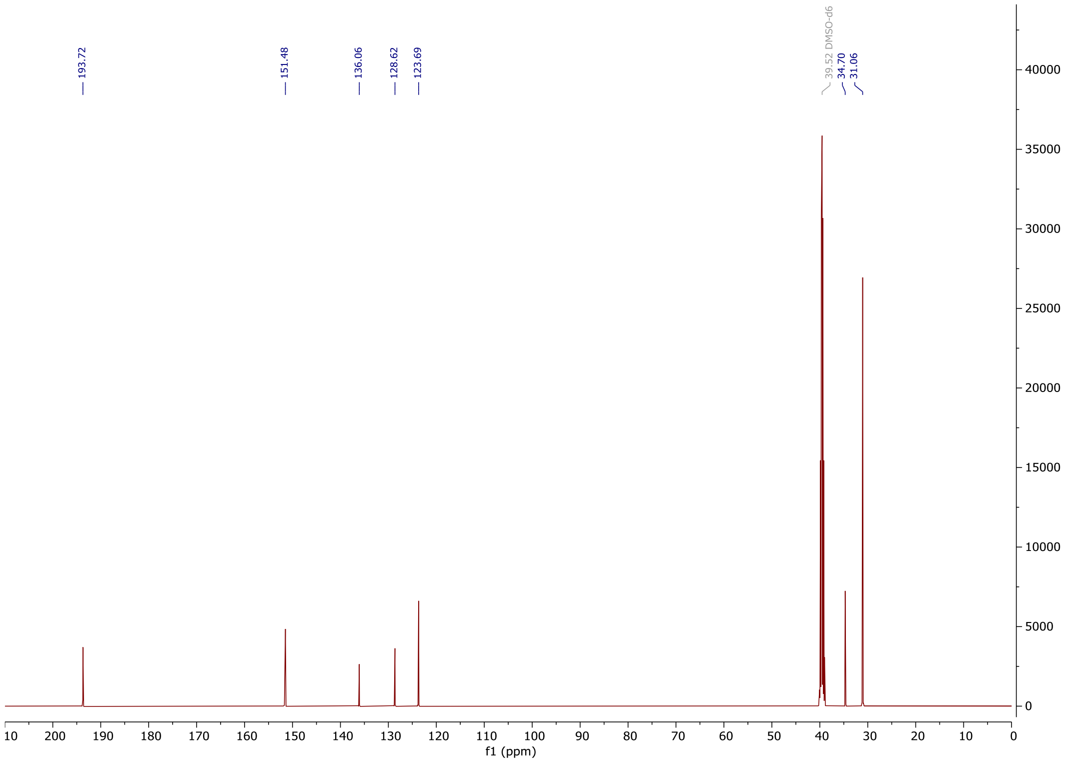

Bis(4-(*tert*-butyl)phenyl)methanol (S3a)

^{1}H-NMR (500 MHz, $CDCl_3$, 300 K)

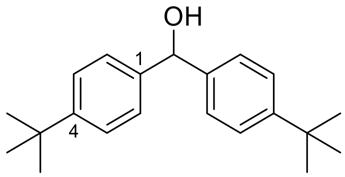

S3a

$C_{21}H_{28}O$

M = 296.45 g/mol

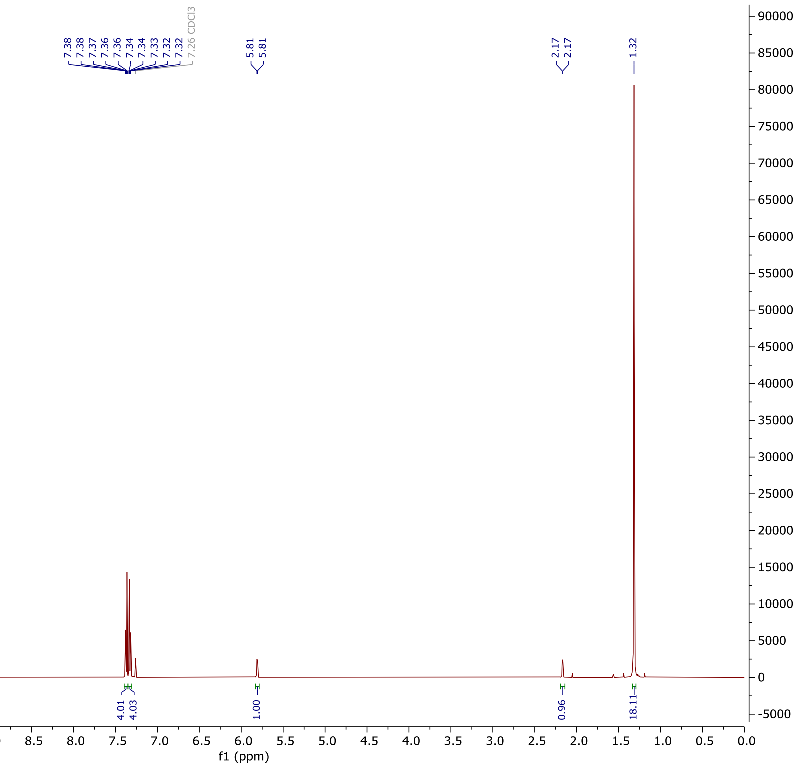

^{13}C-NMR (125 MHz, DMSO-d_6, 300K)

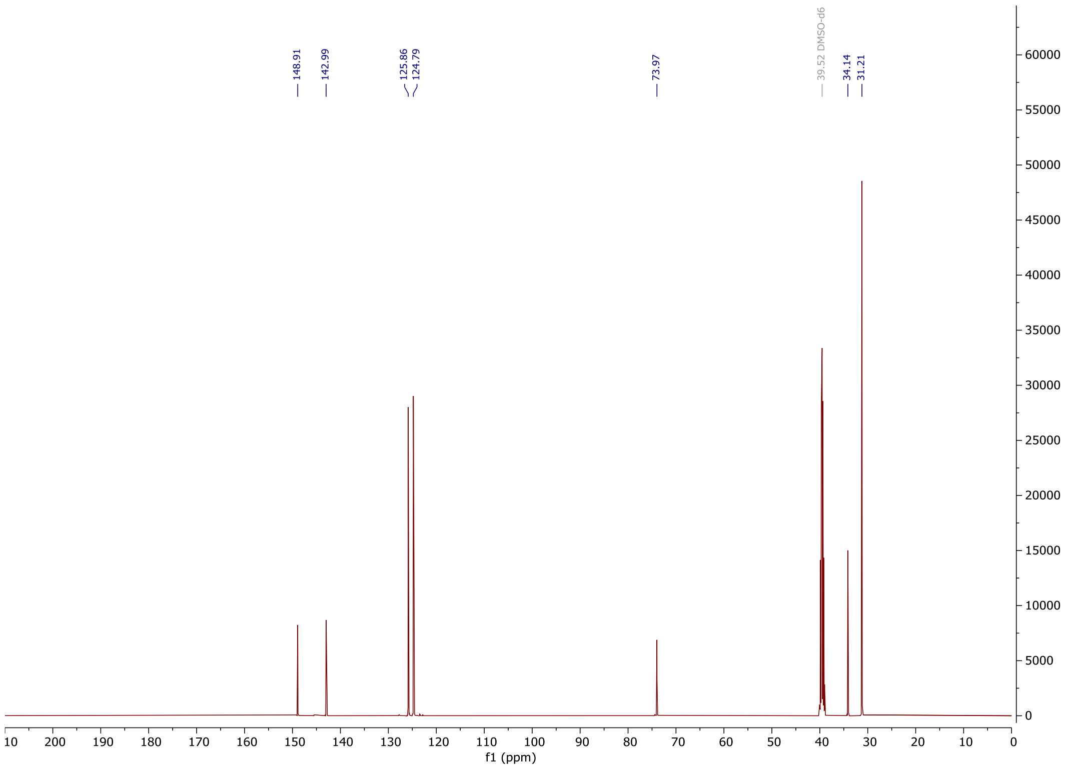

Bis(3,5-di-*tert*-butylphenyl)methanol (S3b)

^{1}H-NMR (500 MHz, $CDCl_3$, 300 K)

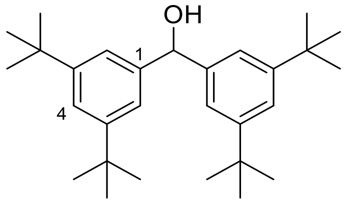

S3b

$C_{29}H_{44}O$

M = 408.67 g/mol

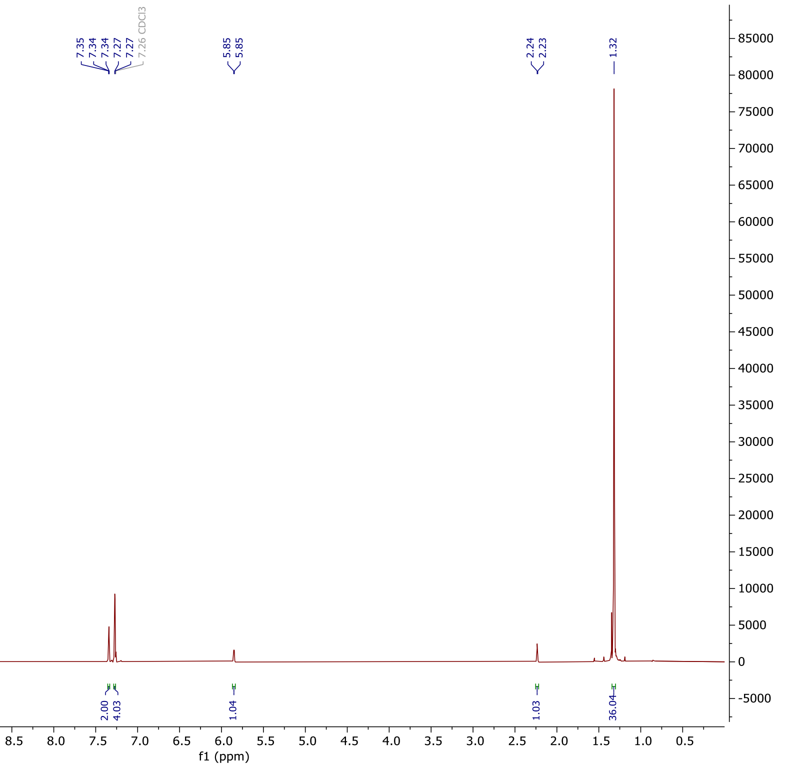

^{13}C-NMR (125 MHz, DMSO-d_6, 300 K)

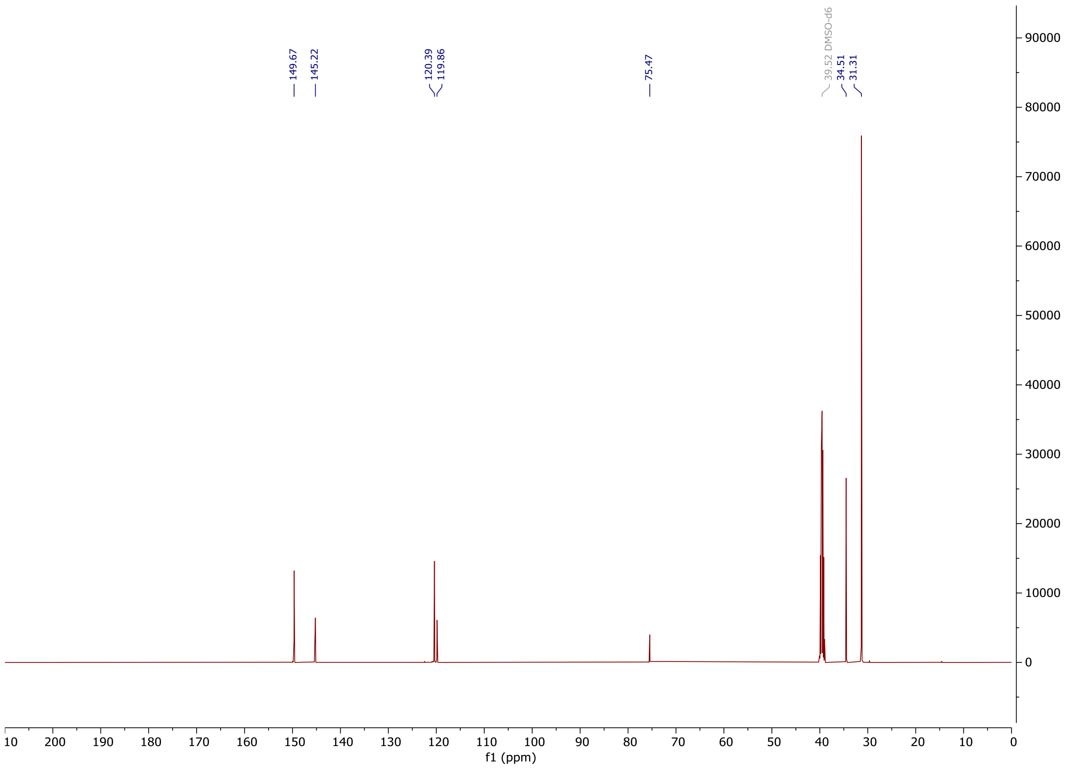

Bis(4-(*tert*-butyl)phenyl)methanone (S4a)

^{1}H-NMR (500 MHz, DMSO-d_6, 300 K)

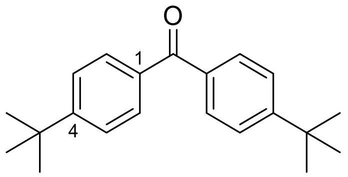

S4a

$C_{21}H_{26}O$

M = 294.44 g/mol

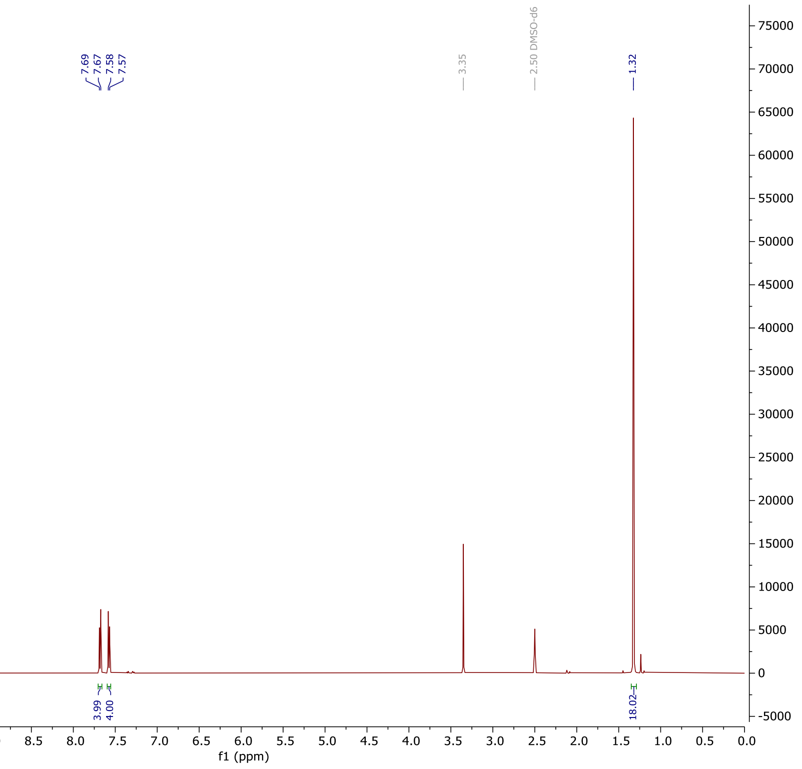

^{13}C-NMR (125 MHz, DMSO-d_6, 300 K)

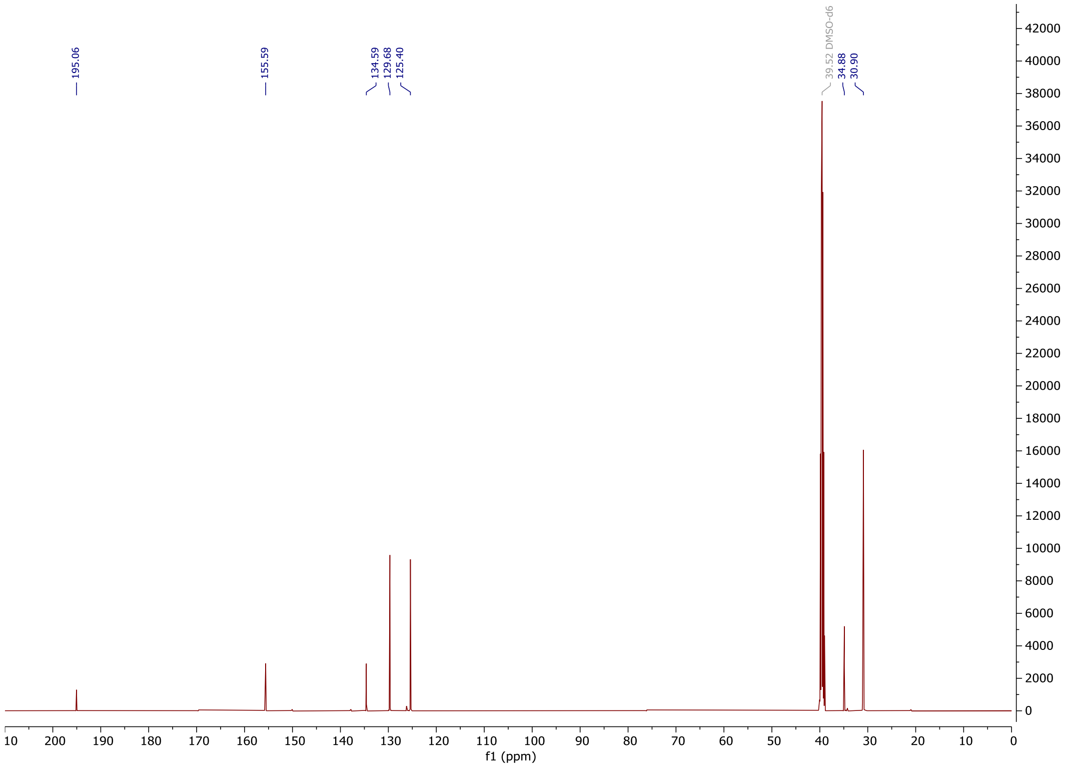

Bis(3,5-di-*tert*-butylphenyl)methanone (S4b)

^{1}H-NMR (500 MHz, $CDCl_3$, 300 K)

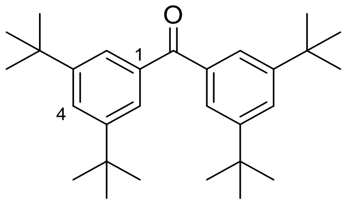

S4b

$C_{29}H_{42}O$

M = 406.65 g/mol

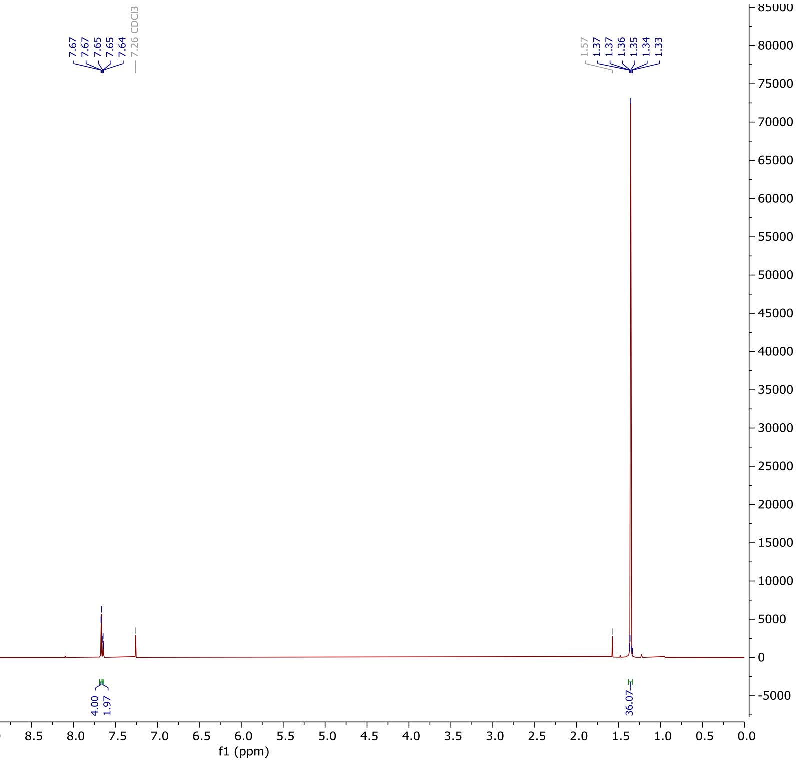

^{13}C-NMR (125 MHz, $CDCl_3$, 300 K)

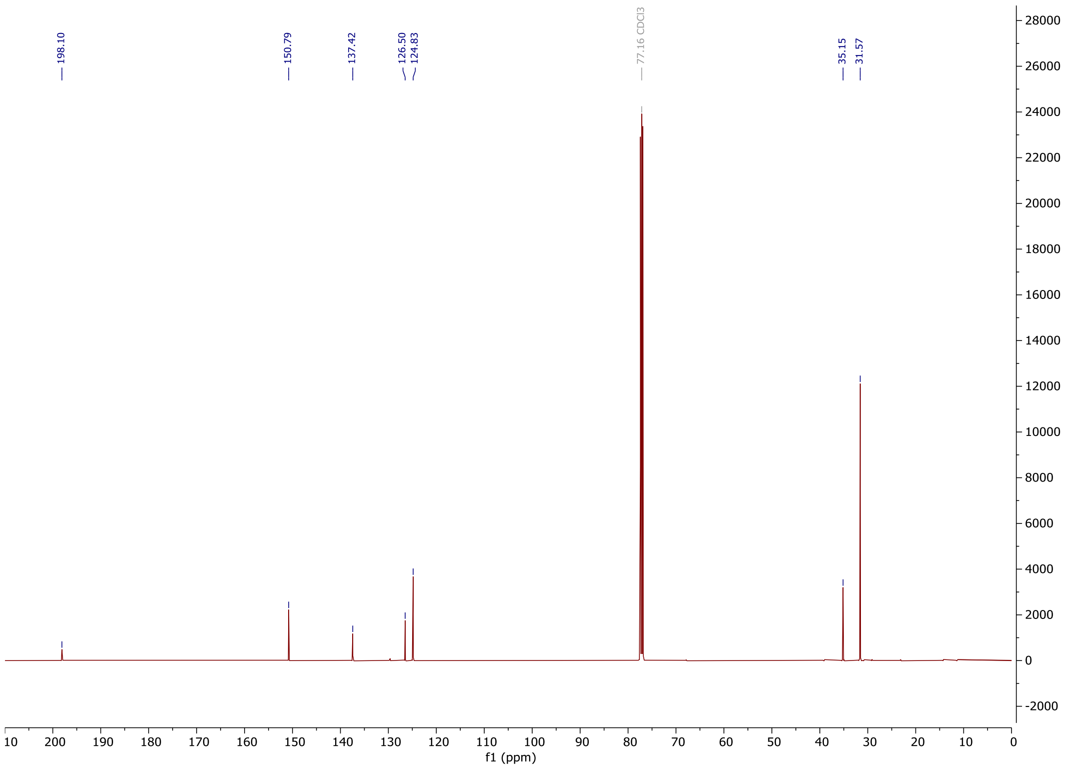

198.10
150.79
137.42
126.50
124.83
77.16 CDCl3
35.15
31.57
f1 (ppm)

Carbonylbis(4,1-phenylene) bis(2,2-dimethylpropanoate) (S4c)

^{1}H-NMR (500 MHz, DMSO-d_6, 300 K)

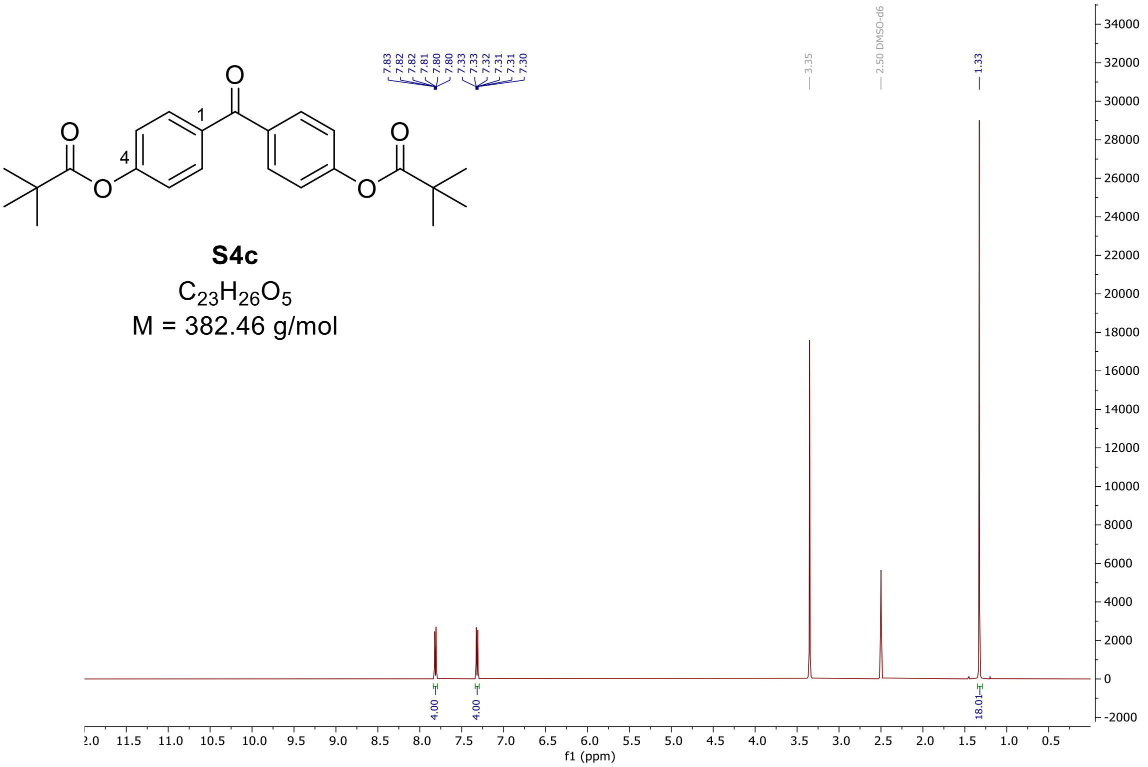

S4c

$C_{23}H_{26}O_5$

M = 382.46 g/mol

^{13}C-NMR (125 MHz, DMSO-d_6, 300 K)

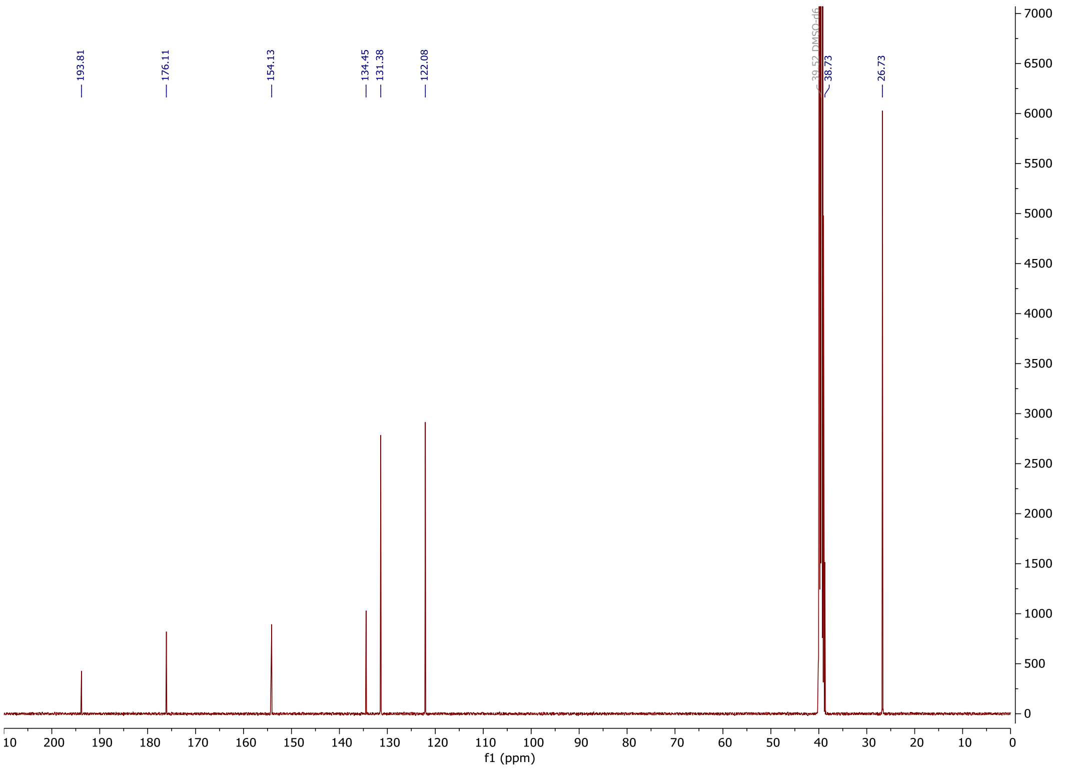

Carbonylbis(4,1-phenylene) bis(4-methylbenzenesulfonate) (S4d)

^{1}H-NMR (500 MHz, DMSO-d_6, 300 K)

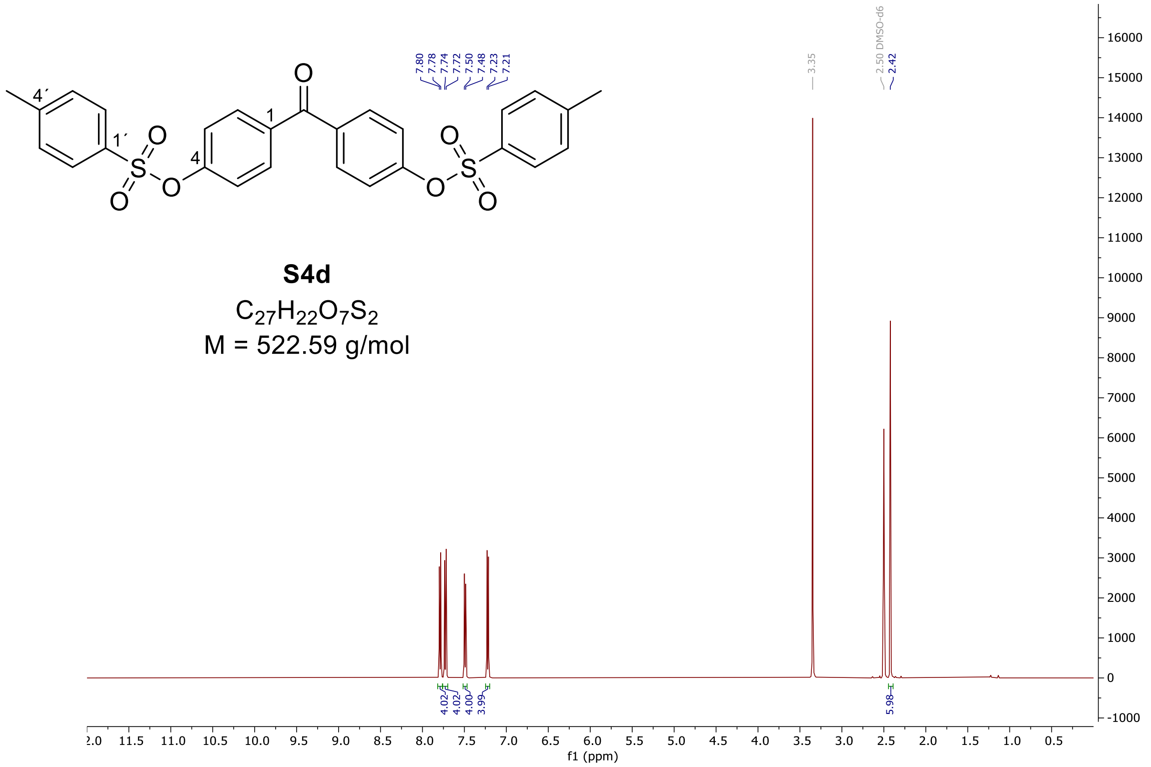

^{13}C-NMR (125 MHz, DMSO-d_6, 300 K)

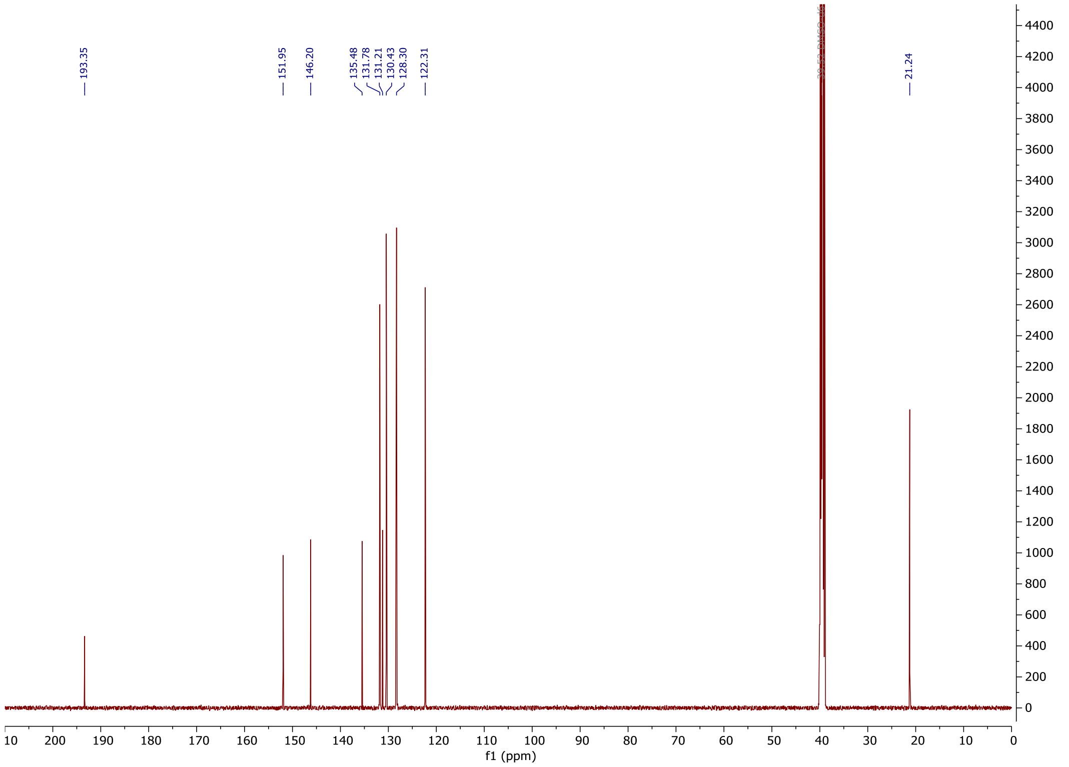

4,4'-(Ethene-1,1-diyl)bis(chlorobenzene) (9a)

^{1}H-NMR (500 MHz, DMSO-d_6, 300 K)

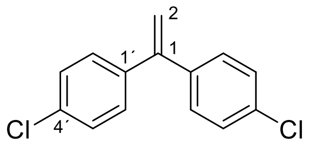

9a

$C_{14}H_{10}Cl_2$

M = 249.13 g/mol

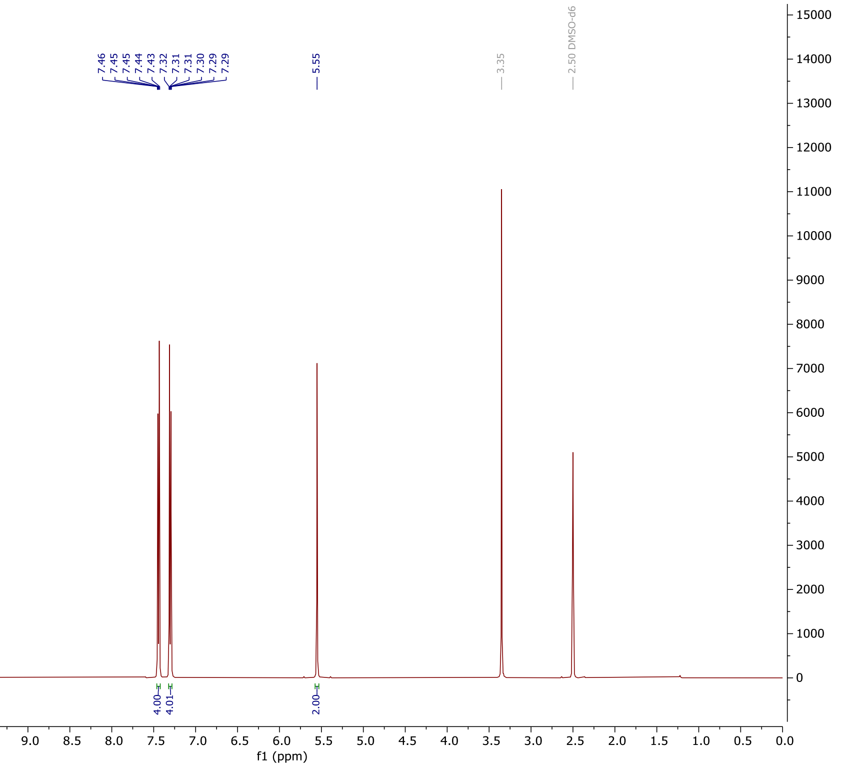

^{13}C-NMR (125 MHz, DMSO-d_6, 300 K)

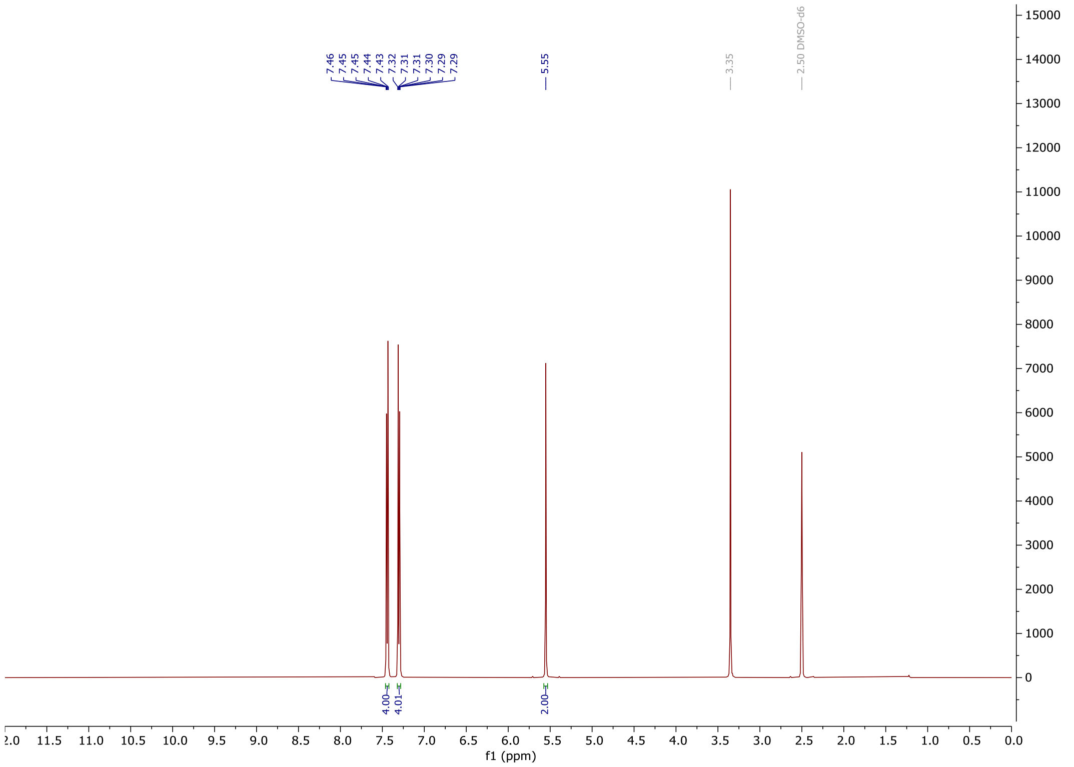

4,4'-(Ethene-1,1-diyl)bis(methylbenzene) (9b)

^{1}H-NMR (500 MHz, $CDCl_3$, 300 K)

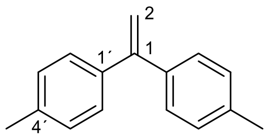

9b

$C_{16}H_{16}$

M = 208.30 g/mol

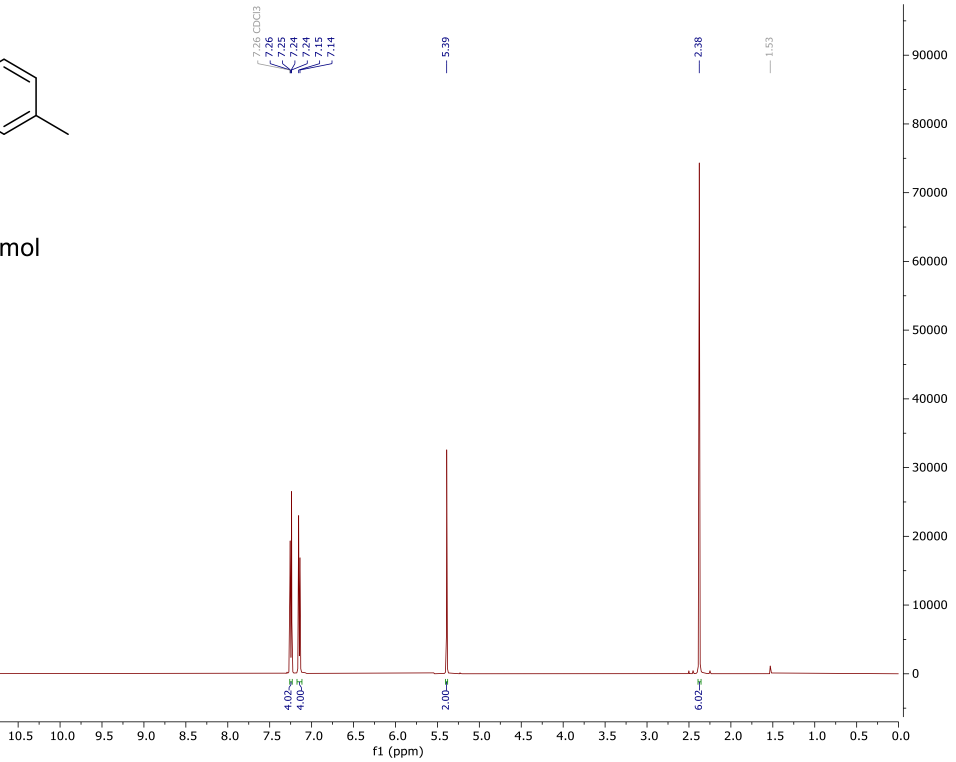

^{13}C-NMR (125 MHz, $CDCl_3$, 300 K)

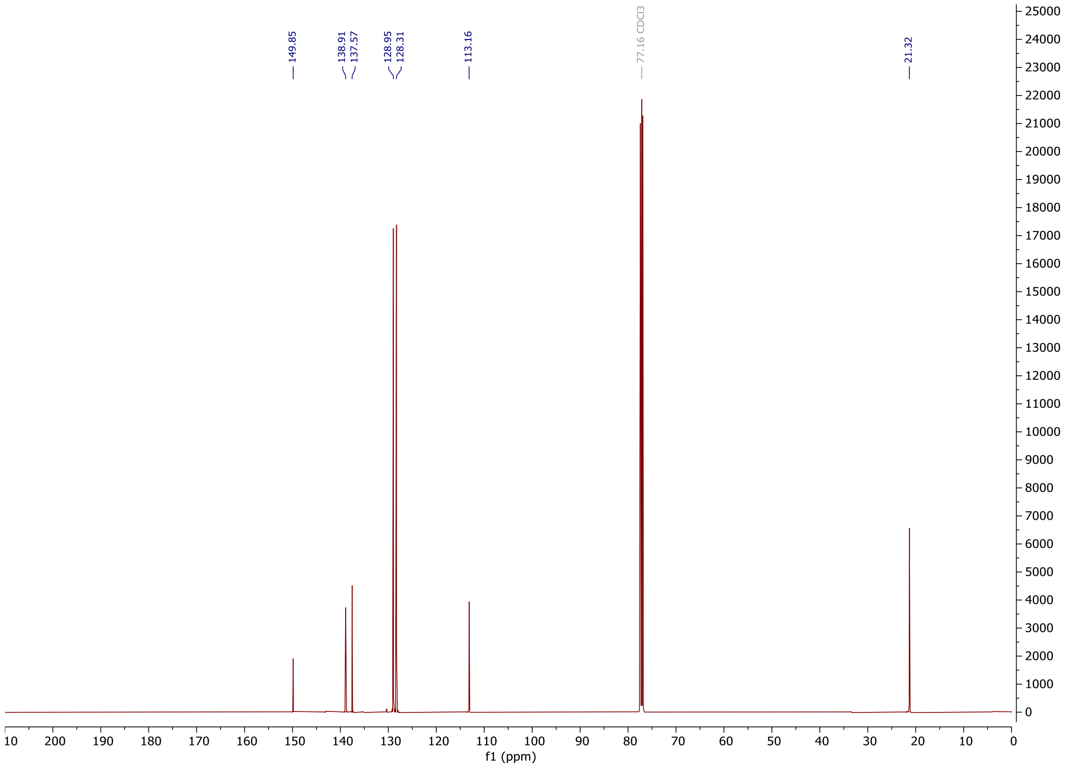

4,4'-(Ethene-1,1-diyl)bis(*tert*-butylbenzene) (9c)

^{1}H-NMR (500 MHz, $CDCl_3$, 300 K)

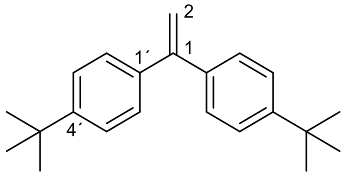

9c

$C_{22}H_{28}$

M = 292.47 g/mol

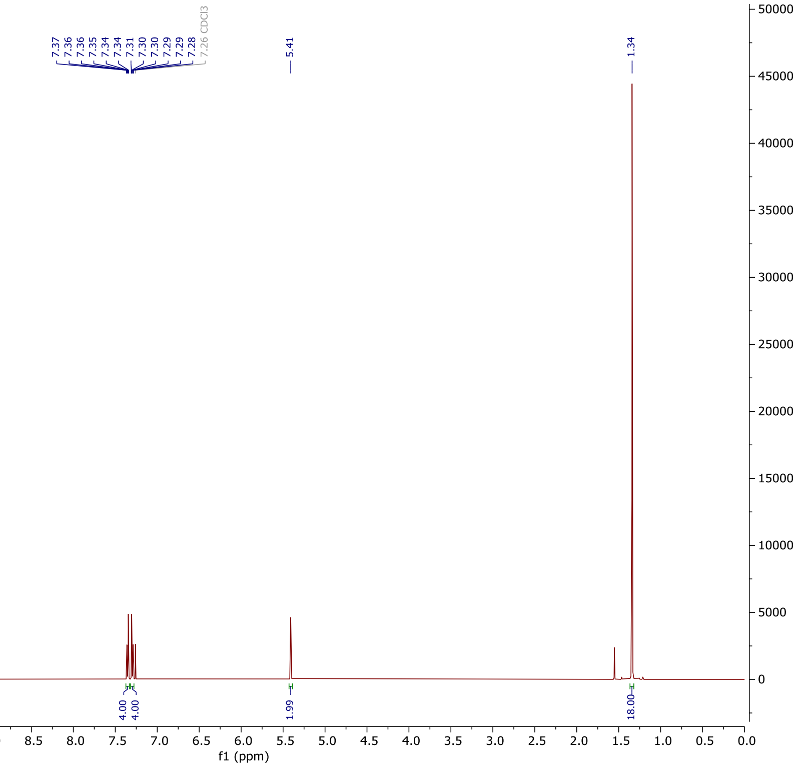

^{13}C-NMR (125 MHz, $CDCl_3$, 300 K)

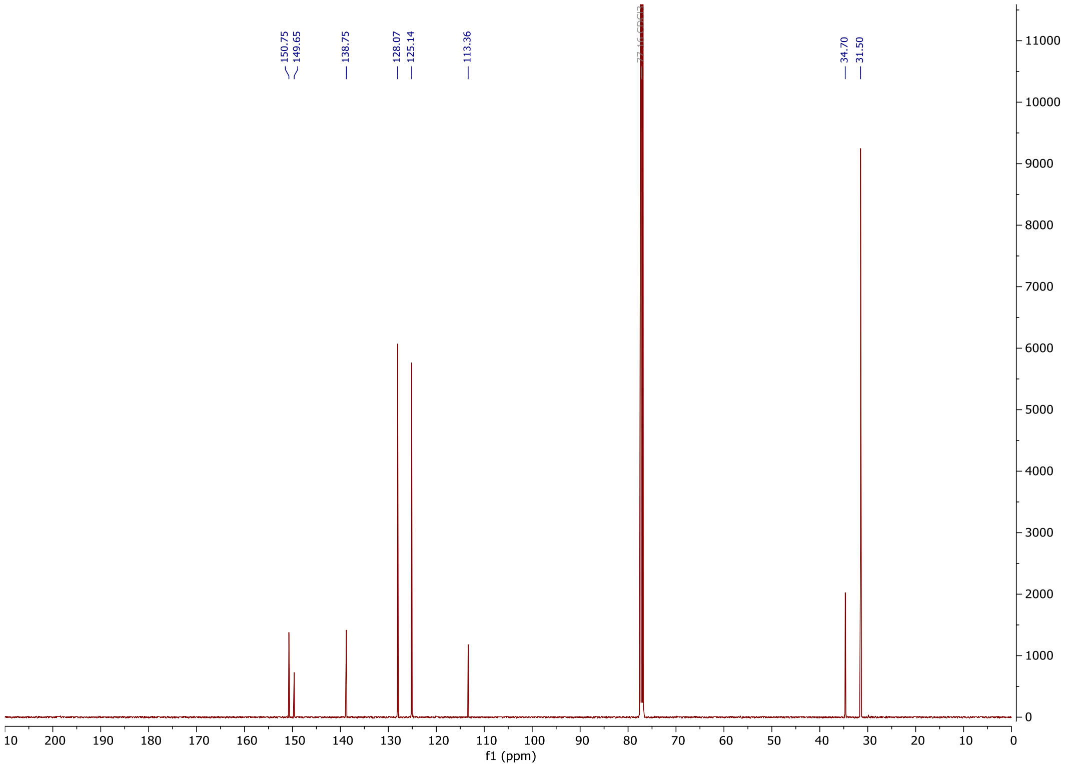

5,5'-(Ethene-1,1-diyl)bis(1,3-di-*tert*-butylbenzene) (9d)

^{1}H-NMR (500 MHz, C_6D_6, 300 K)

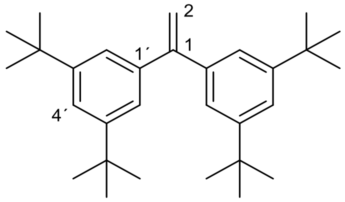

9d
$C_{30}H_{44}$
M = 404.68 g/mol

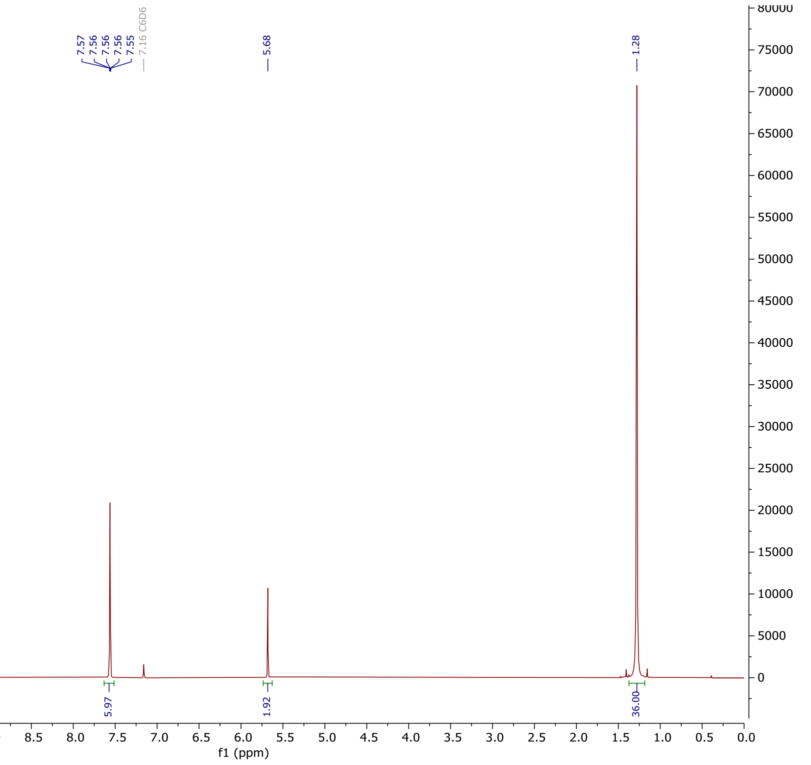

^{13}C-NMR (125 MHz, C_6D_6, 300 K)

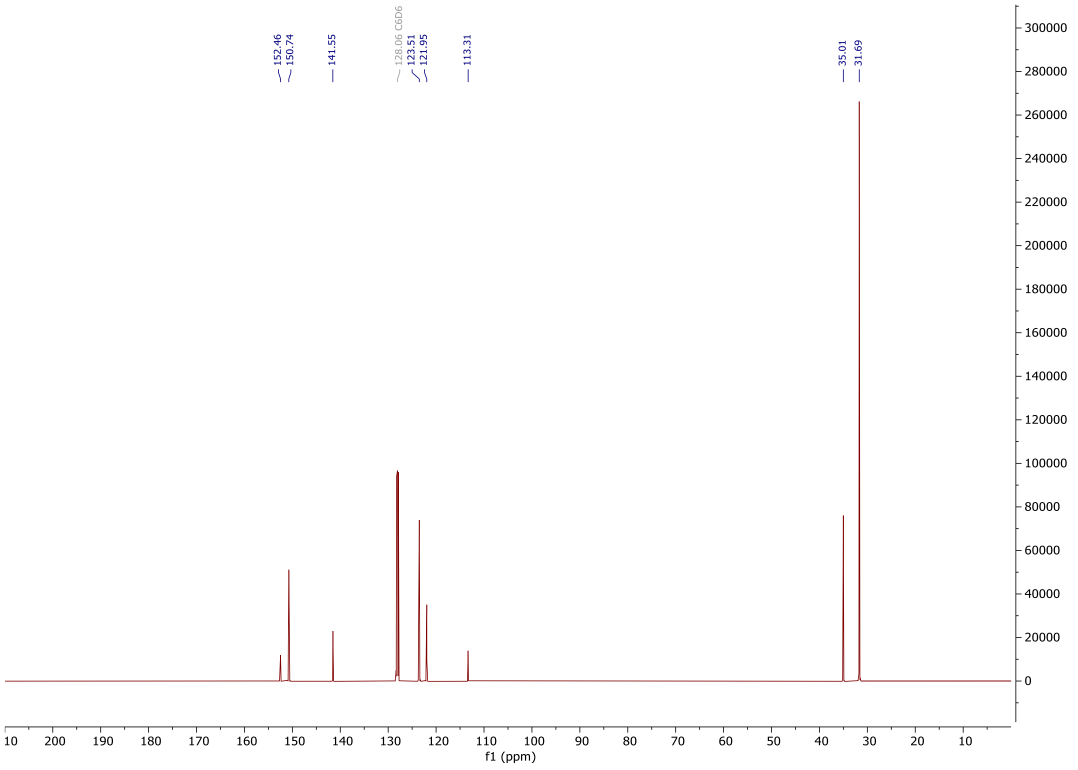

4,4'-(Ethene-1,1-diyl)bis(fluorobenzene) (9f)

^{1}H-NMR (500 MHz, $CDCl_3$, 300 K)

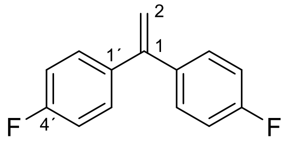

9f

$C_{14}H_{10}F_2$

M = 216.23 g/mol

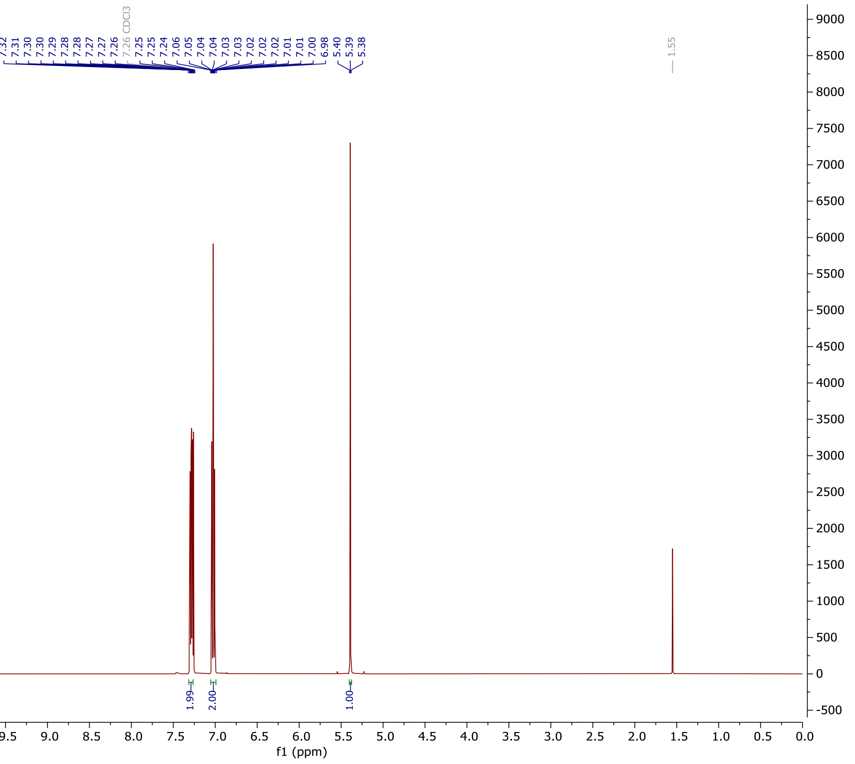

^{13}C-NMR (125 MHz, $CDCl_3$, 300 K)

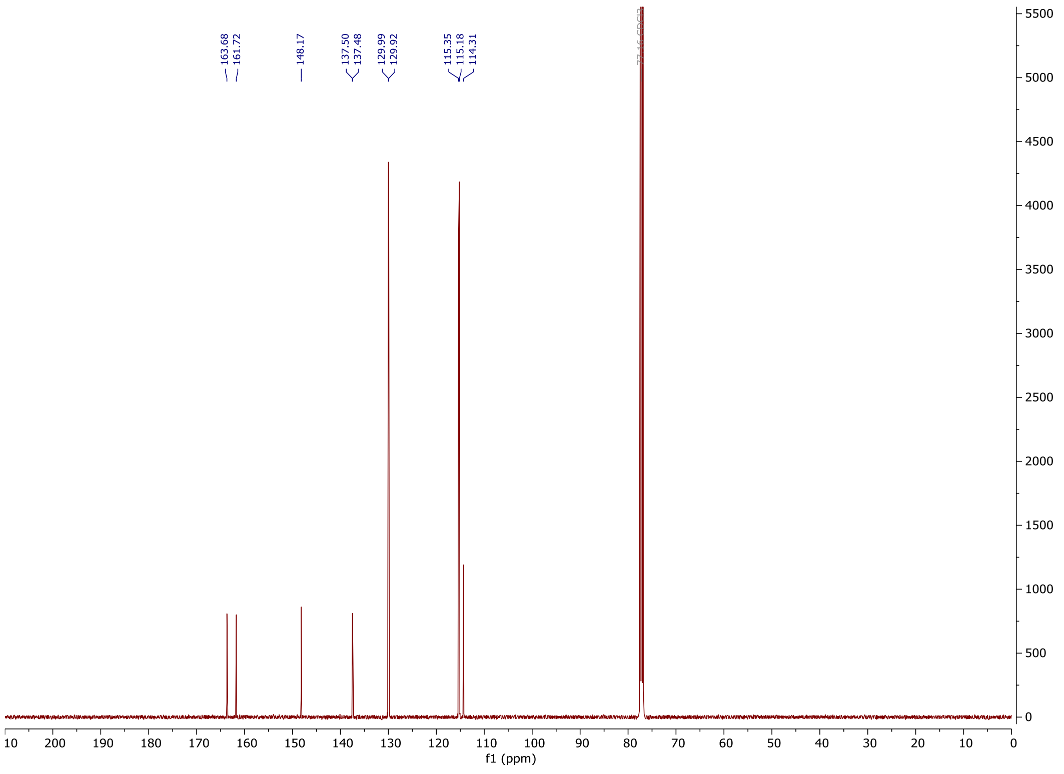

^{19}F-NMR (470 MHz, $CDCl_3$, 300 K)

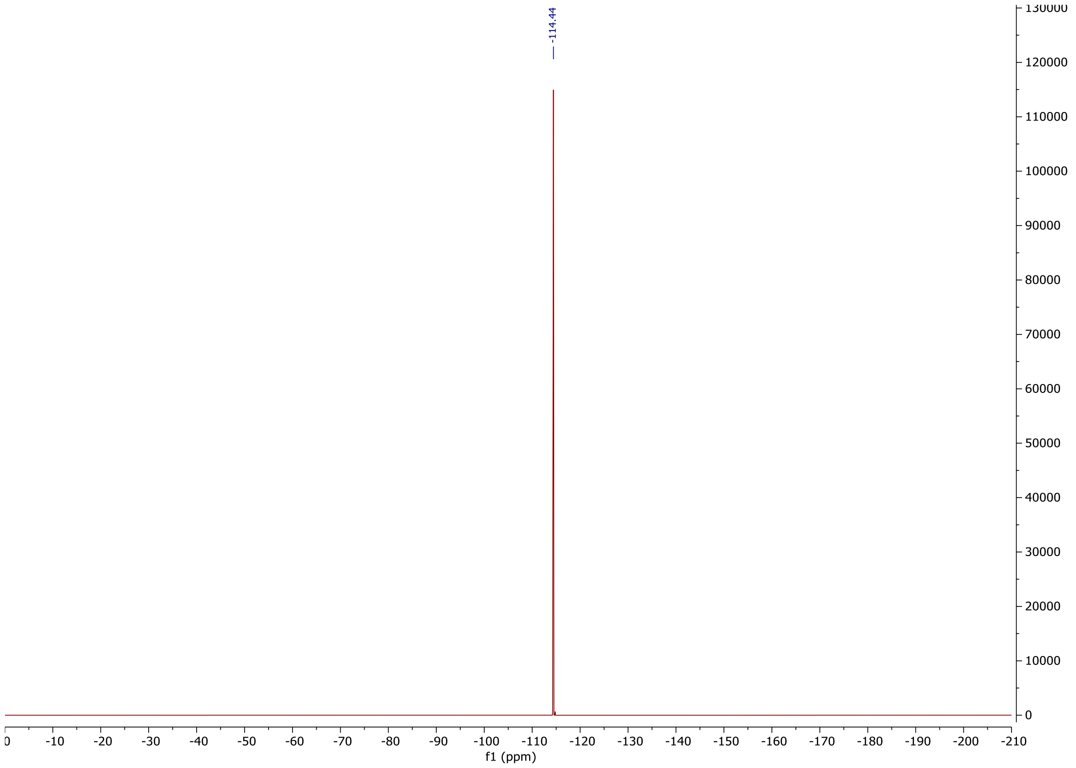

4,4'-(Ethene-1,1-diyl)bis(bromobenzene) (9g)

^{1}H-NMR (500 MHz, DMSO-d_6, 300 K)

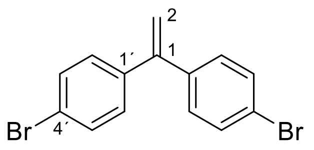

9g
$C_{14}H_{10}Br_2$
M = 338.04 g/mol

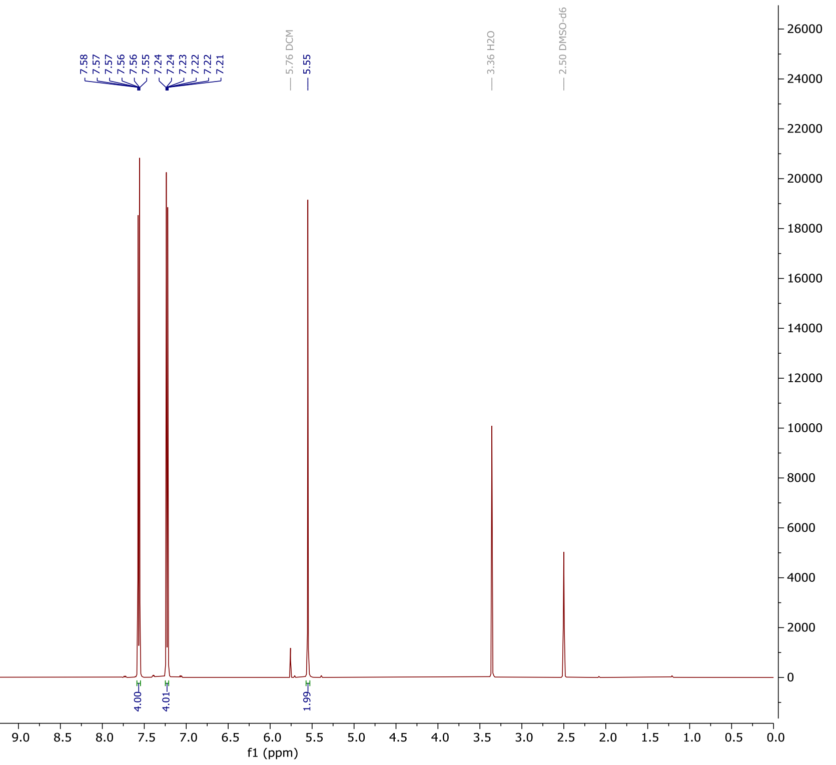

^{13}C-NMR (125 MHz, DMSO-d_6, 300 K)

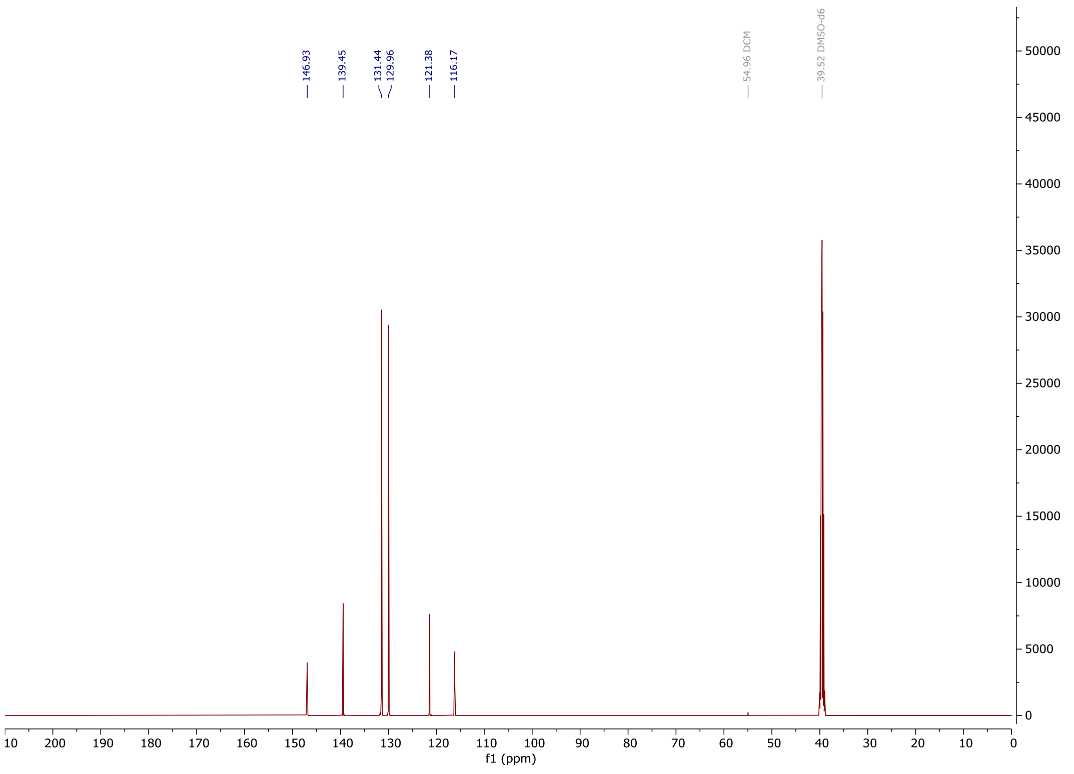

5,5'-(Ethene-1,1-diyl)bis(1,3-bis(trifluoromethyl)benzene) (9h)

^{1}H-NMR (500 MHz, C_6D_6, 300 K)

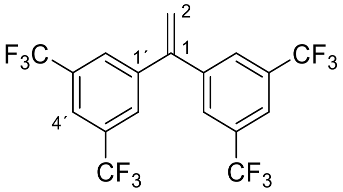

9h

$C_{18}H_8F_{12}$

M = 452.24 g/mol

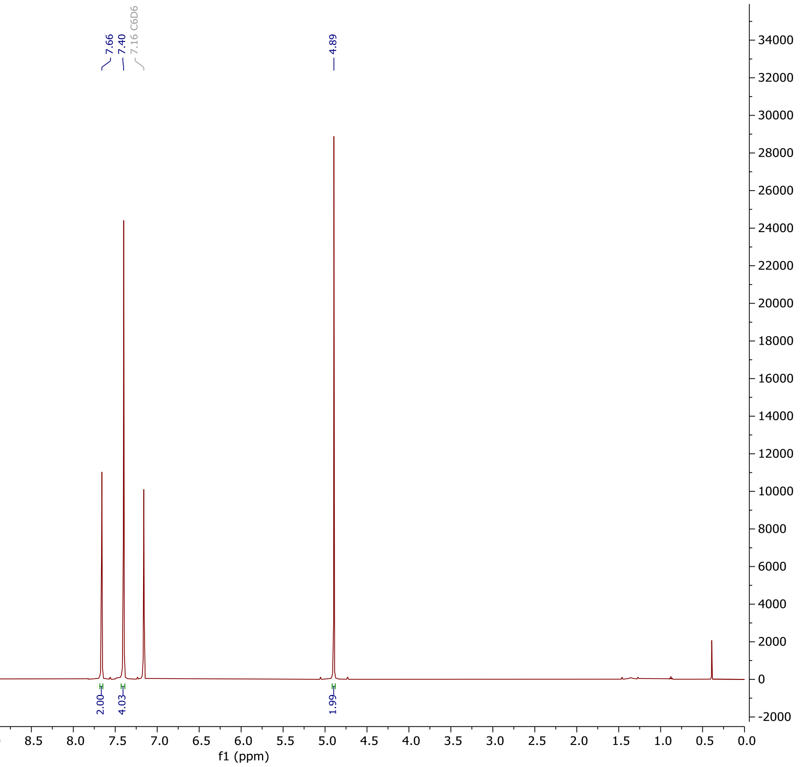

^{13}C-NMR (125 MHz, C_6D_6, 300 K)

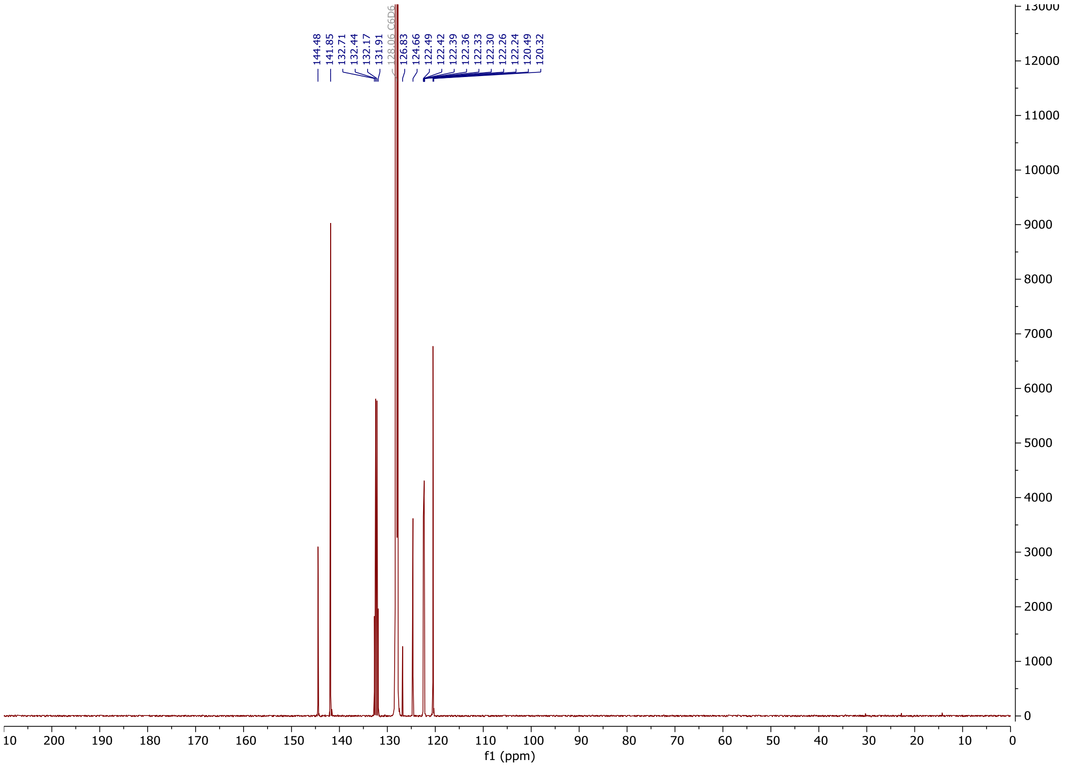

^{19}F-NMR (470 MHz, C_6D_6, 300 K)

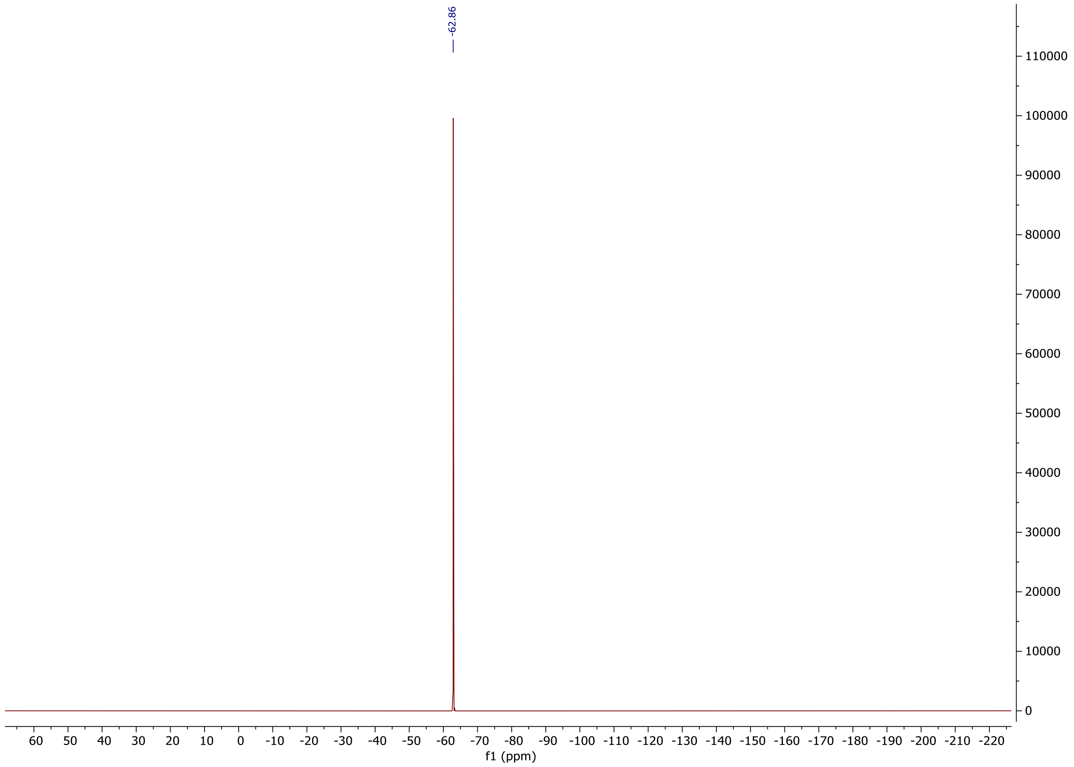

3,3'-(Ethene-1,1-diyl)bis(nitrobenzene) (9i)

^{1}H-NMR (500 MHz, $CDCl_3$, 300 K)

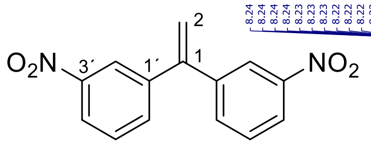

9i

$C_{14}H_{10}N_2O_4$

M = 270.24 g/mol

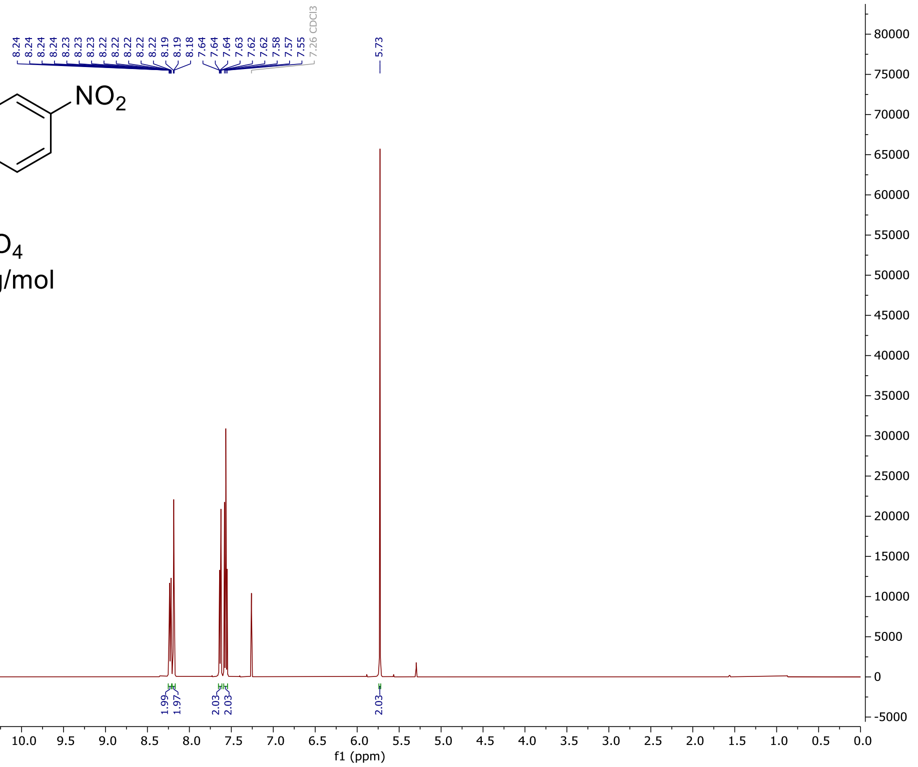

^{13}C-NMR (125 MHz, $CDCl_3$, 300 K)

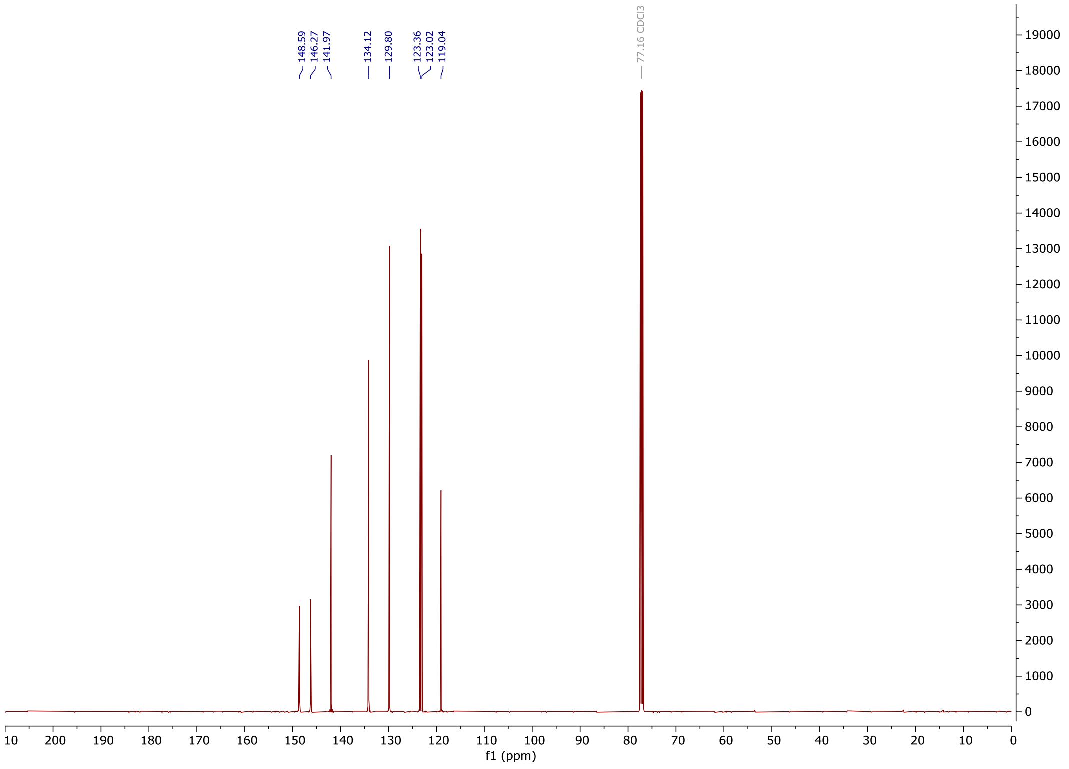

Ethene-1,1-diylbis(4,1-phenylene) bis(4-methylbenzenesulfonate) (9j)

[1]**H-NMR** (500 MHz, DMSO-d_6, 300 K)

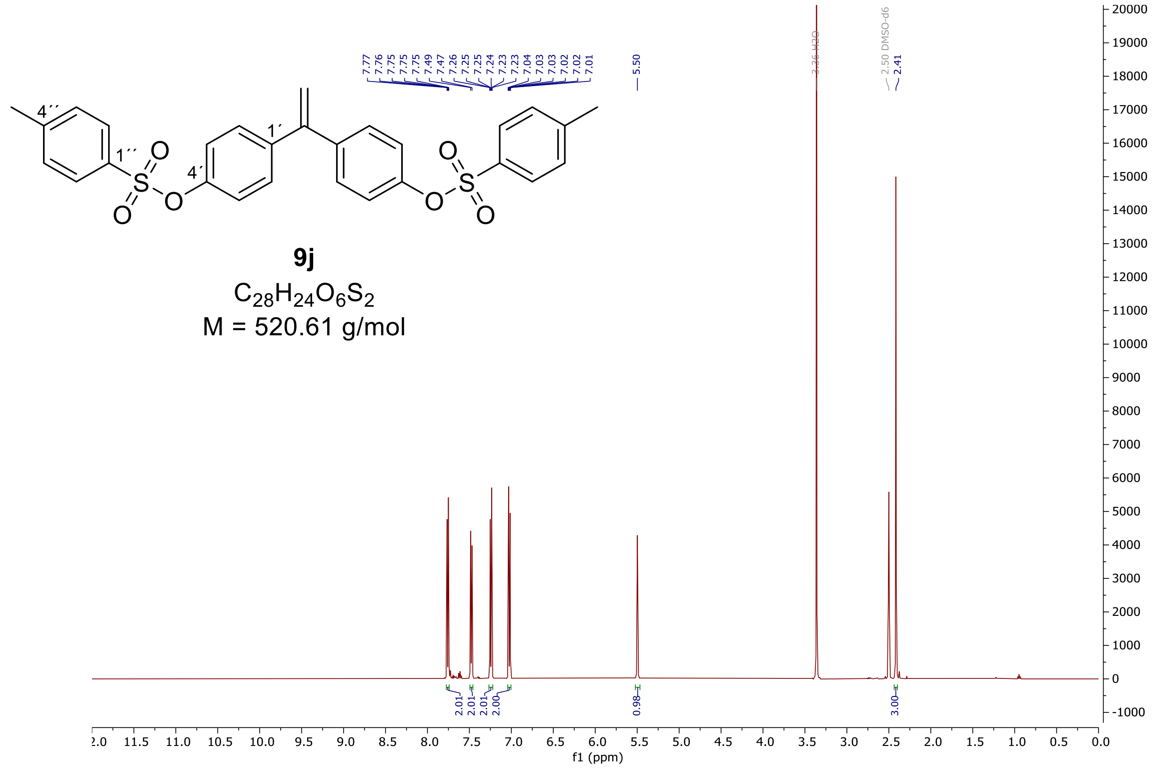

[13]**C-NMR** (125 MHz, DMSO-d_6, 300 K)

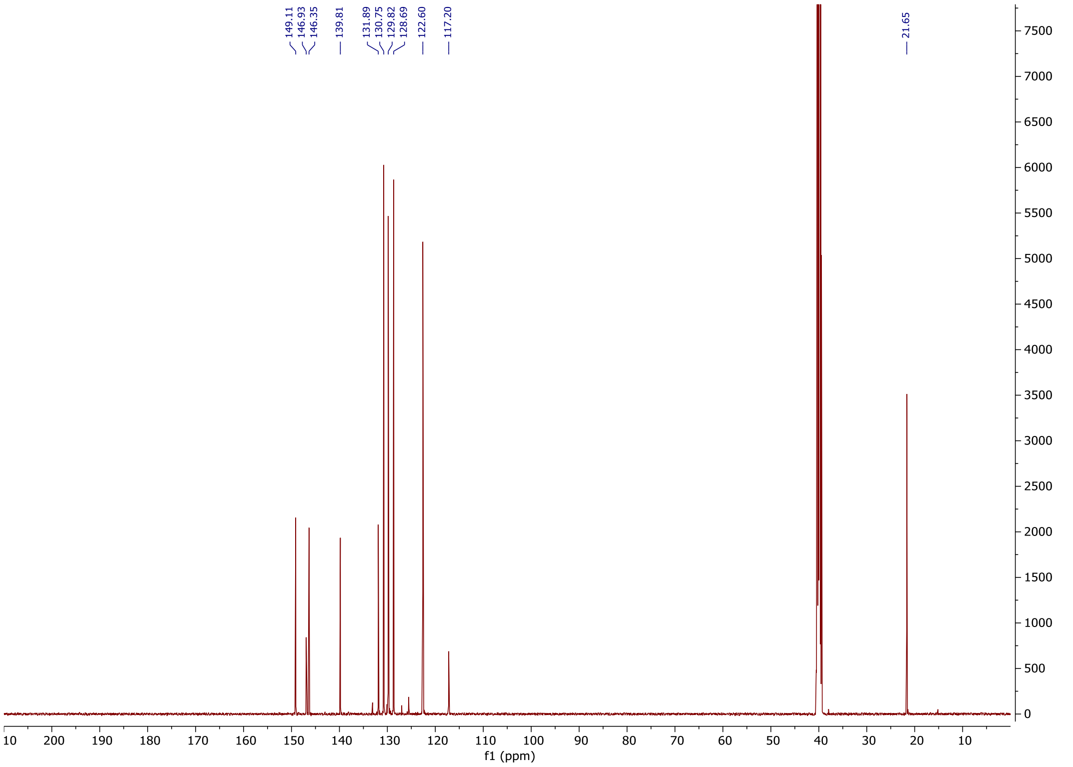

Ethene-1,1-diylbis(4,1-phenylene) bis(2,2-dimethylpropanoate) (9k)

^{1}H-NMR (500 MHz, $CDCl_3$, 300 K)

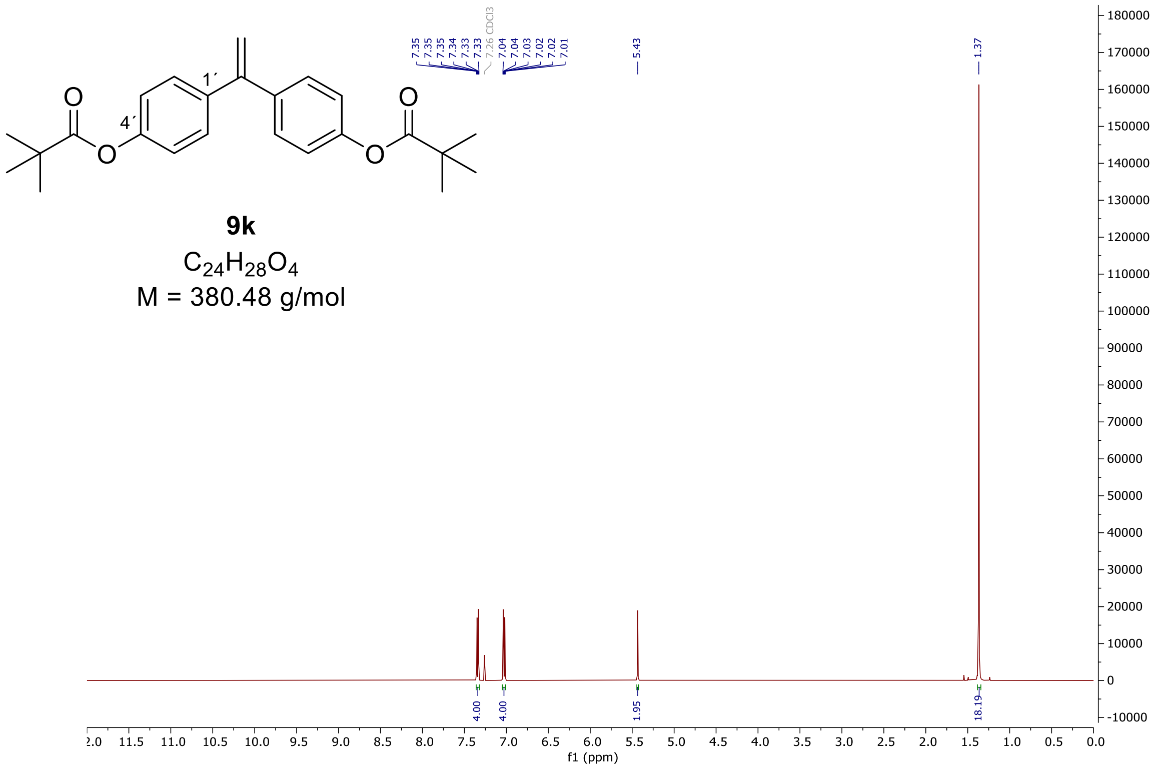

^{13}C-NMR (125 MHz, $CDCl_3$, 300 K)

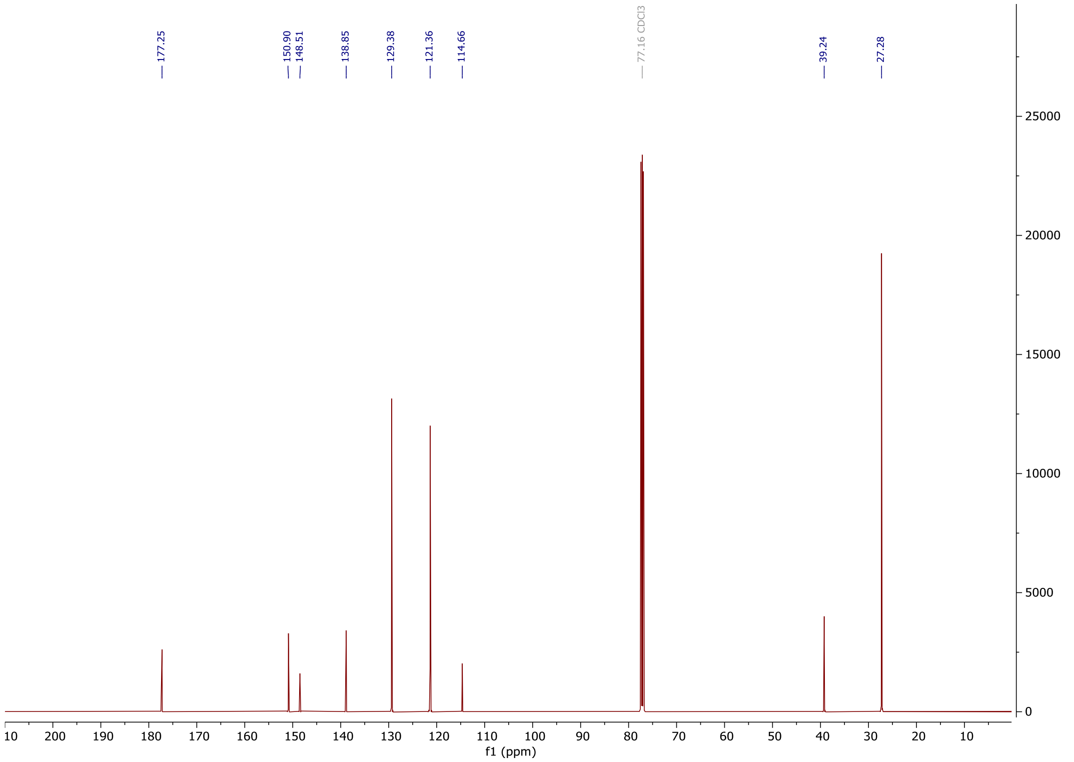

1-Acetylindoline-2,3-dione (S5a)

^{1}H-NMR (500 MHz, $CDCl_3$, 300 K)

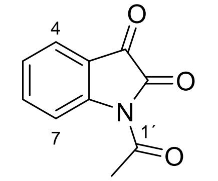

S5a

$C_{10}H_7NO_3$

M = 189.17 g/mol

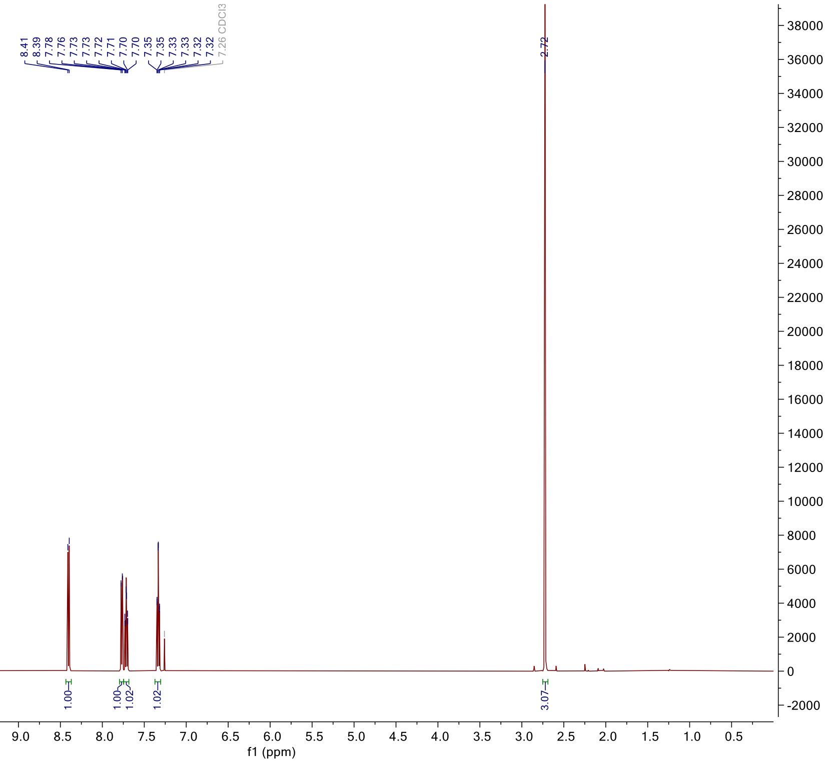

^{13}C-NMR (125 MHz, $CDCl_3$, 300 K)

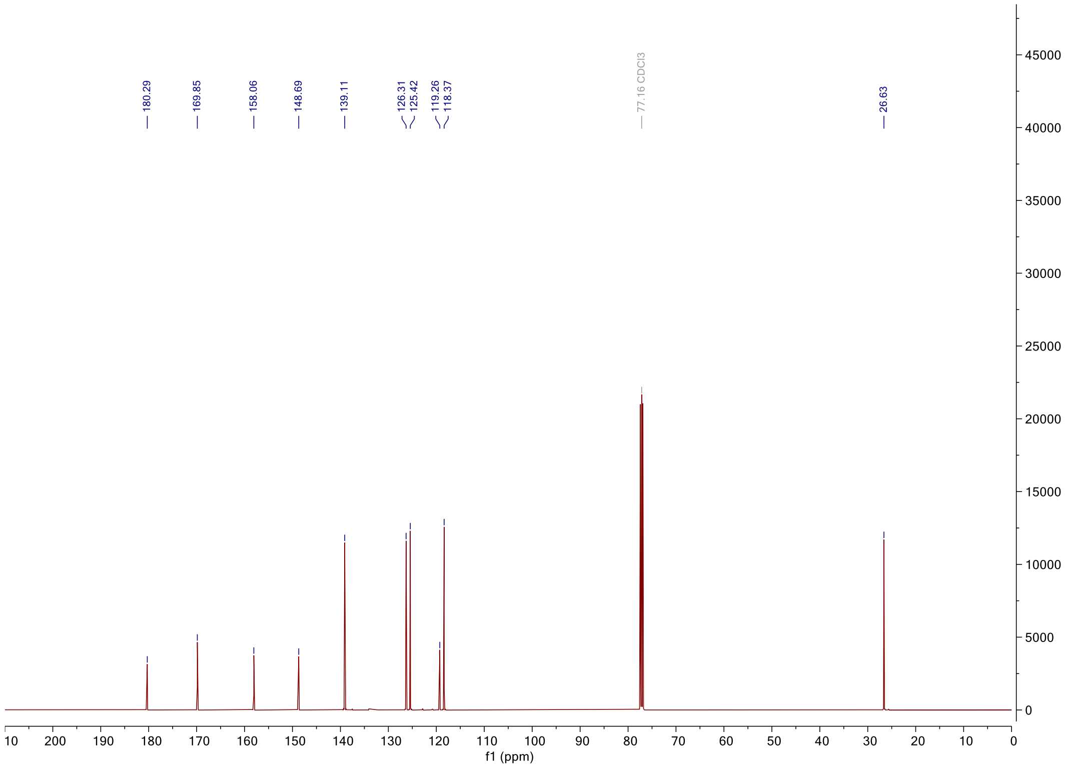

1-Acetyl-5-fluoroindoline-2,3-dione (S5b)

^{1}H-NMR (400 MHz, $CDCl_3$, 300 K)

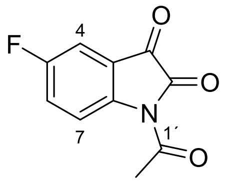

S5b

$C_{10}H_6FNO_3$

M = 207.16 g/mol

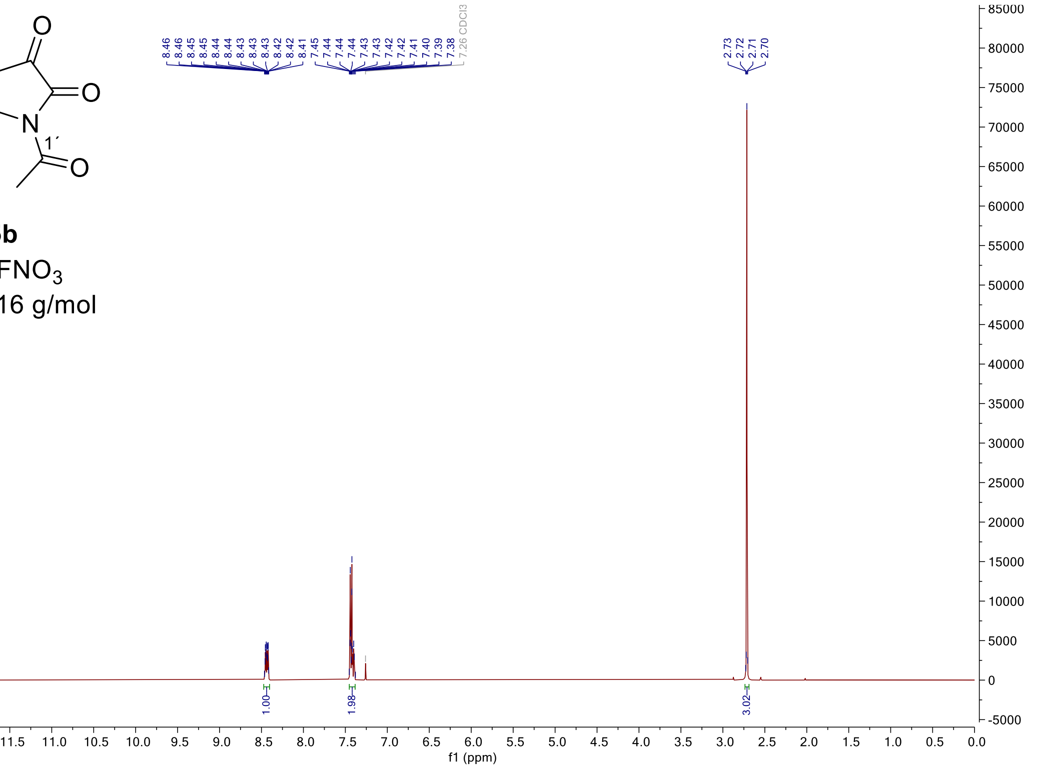

^{13}C-NMR (100 MHz, $CDCl_3$, 300 K)

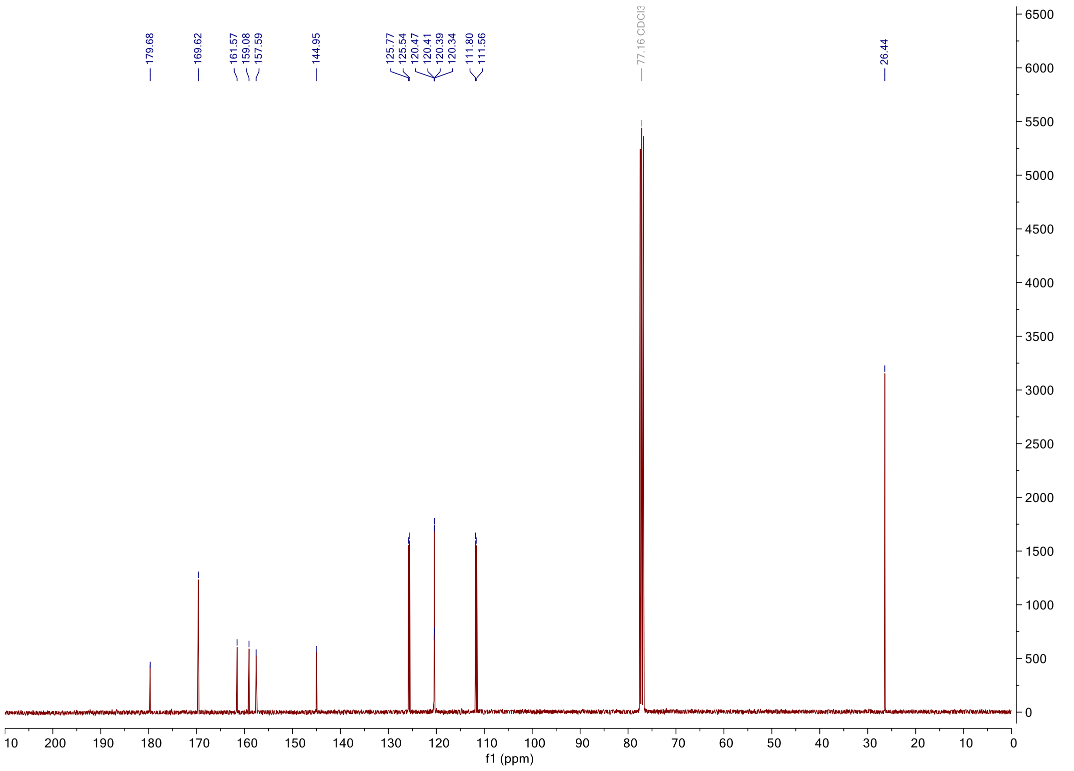

^{19}F-NMR (376 MHz, CDCl3, 300 K)

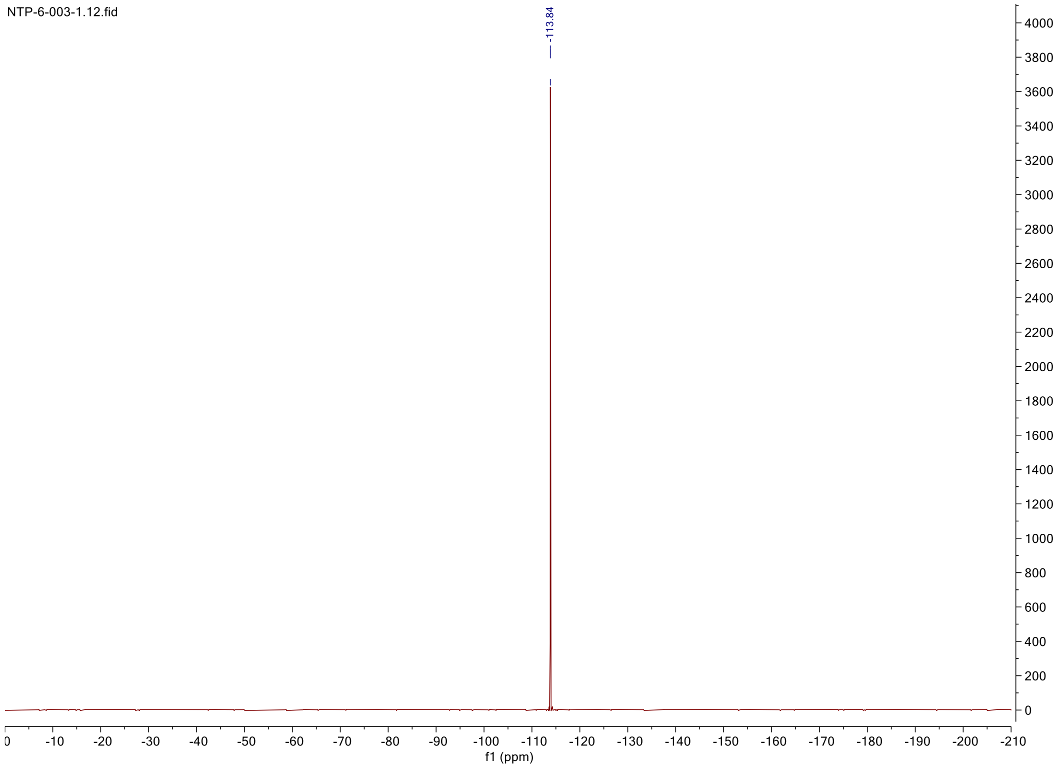

1-**Acetyl-6-chloroindoline-2,3-dione (S5c)**

^{1}H-NMR (500 MHz, $CDCl_3$, 300 K)

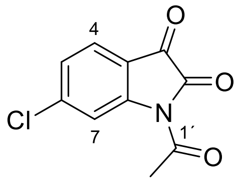

S5c

$C_{10}H_6ClNO_3$

M = 223.61 g/mol

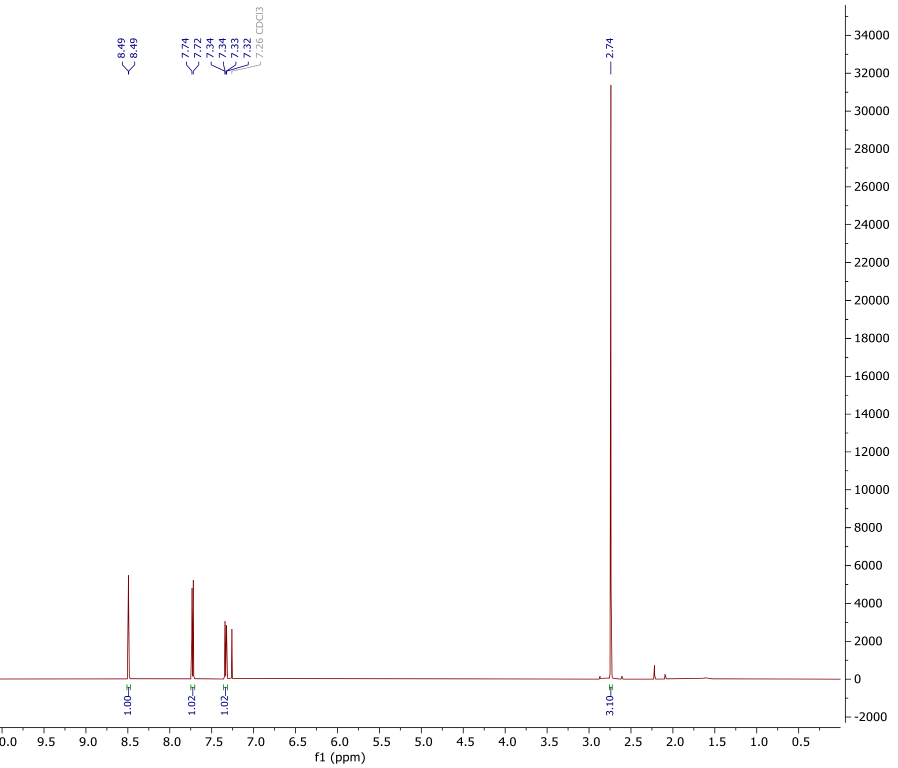

^{13}C-NMR (125 MHz, $CDCl_3$, 300 K)

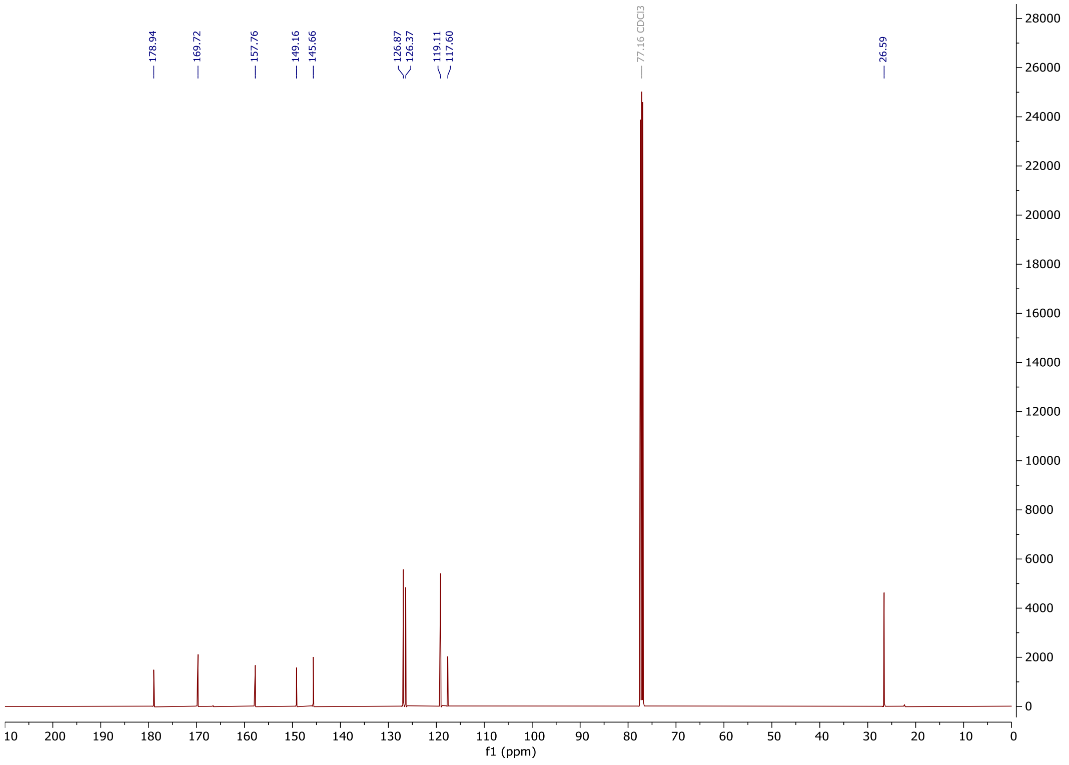

3',3'-Bis(4-chlorophenyl)spiro[indoline-3,2'-oxetan]-2-one (*rac*-5a)

^{1}H-NMR (500 MHz, DMSO-d_6, 300 K)

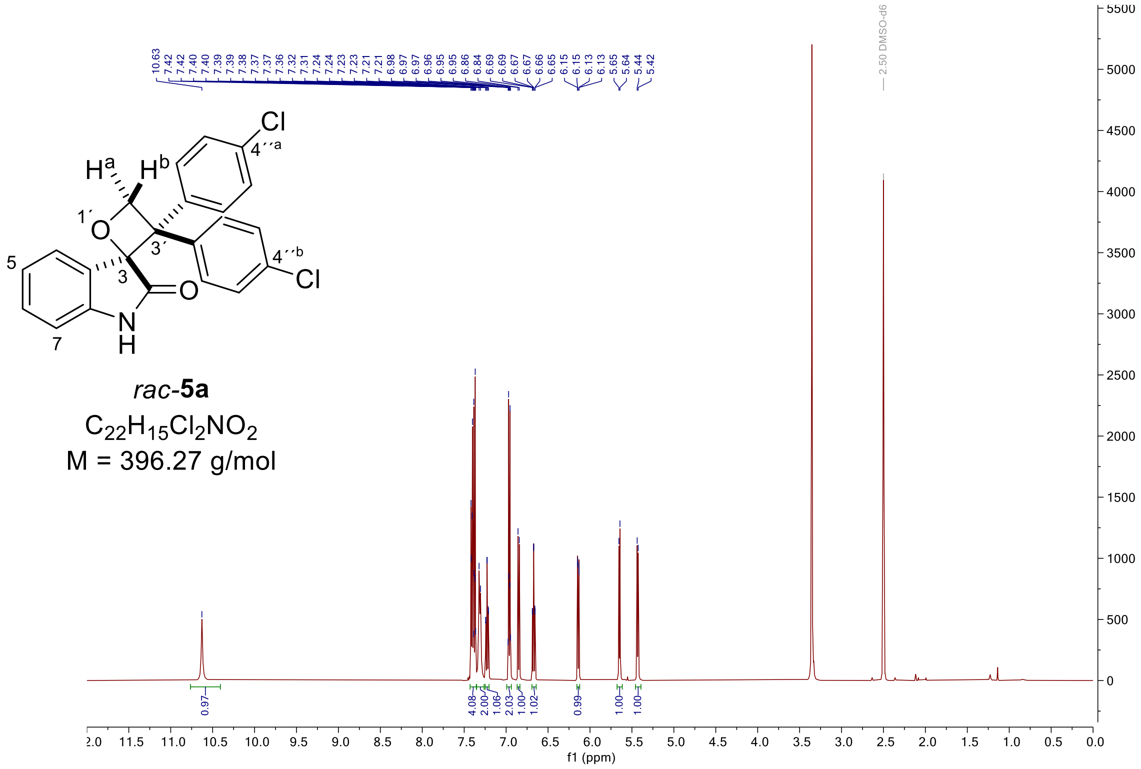

^{13}C-NMR (125 MHz, DMSO-d_6, 300 K)

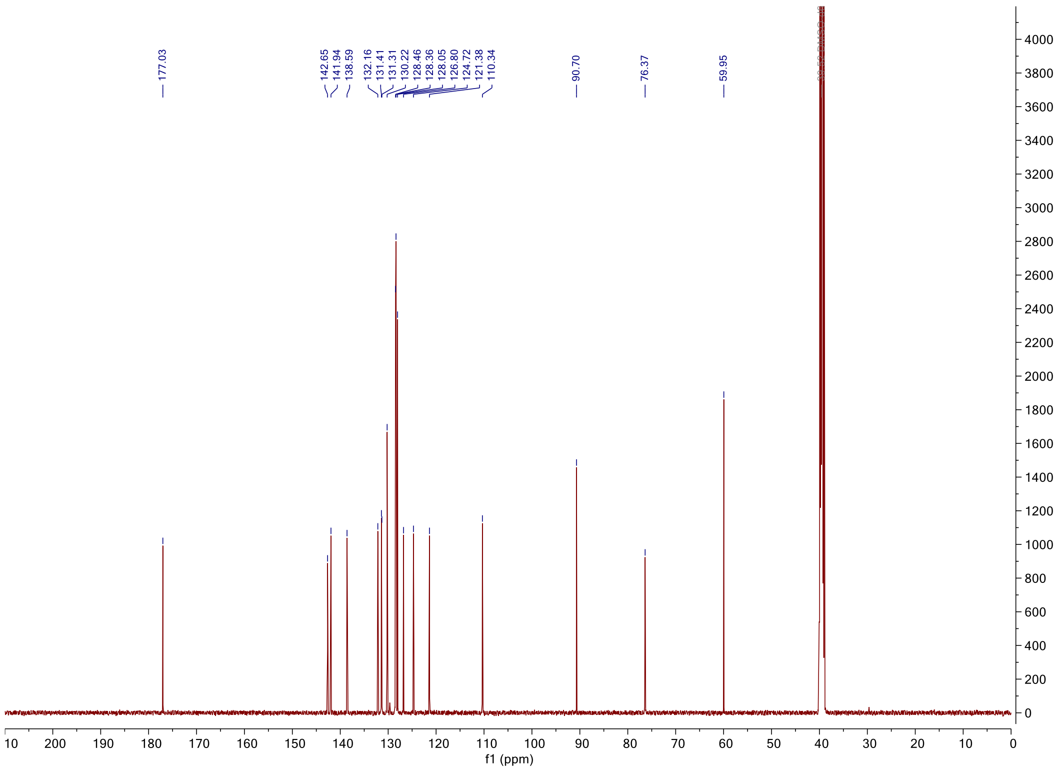

3',3'-Di-*p*-tolylspiro[indoline-3,2'-oxetan]-2-one (*rac*-5b)

^{1}H-NMR (500 MHz, DMSO-d_6, 300 K)

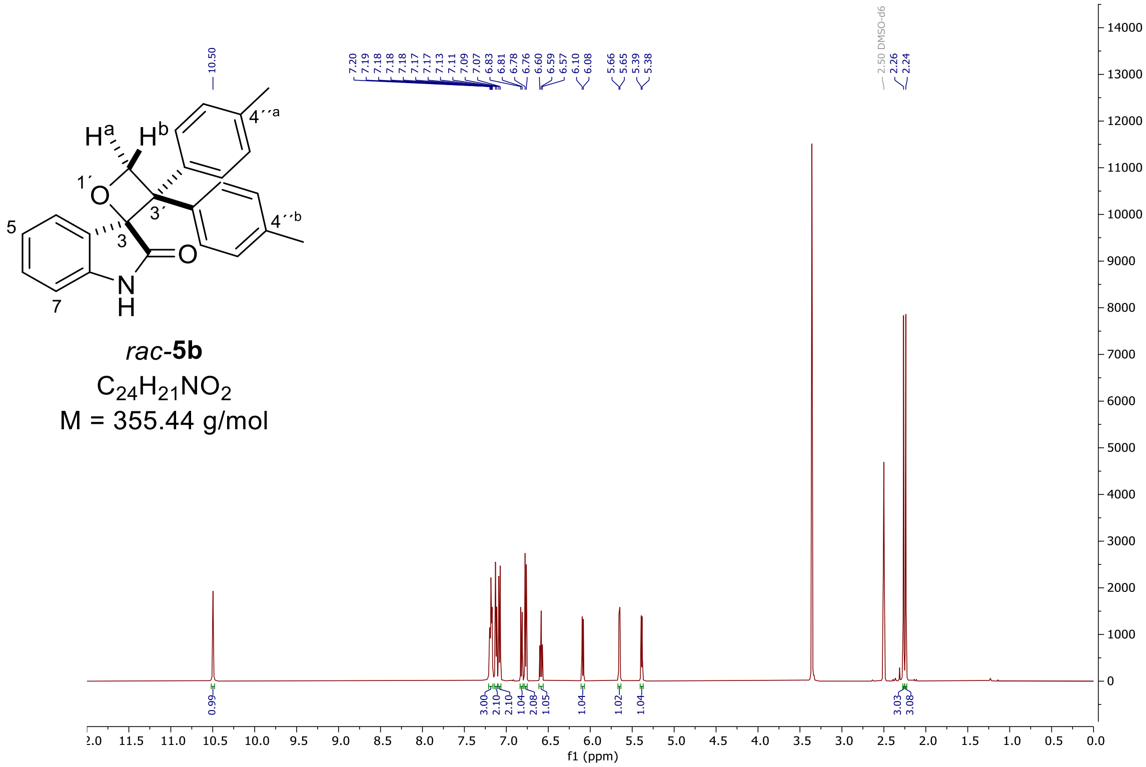

rac-**5b**
$C_{24}H_{21}NO_2$
M = 355.44 g/mol

^{13}C-NMR (125 MHz, DMSO-d_6, 300 K)

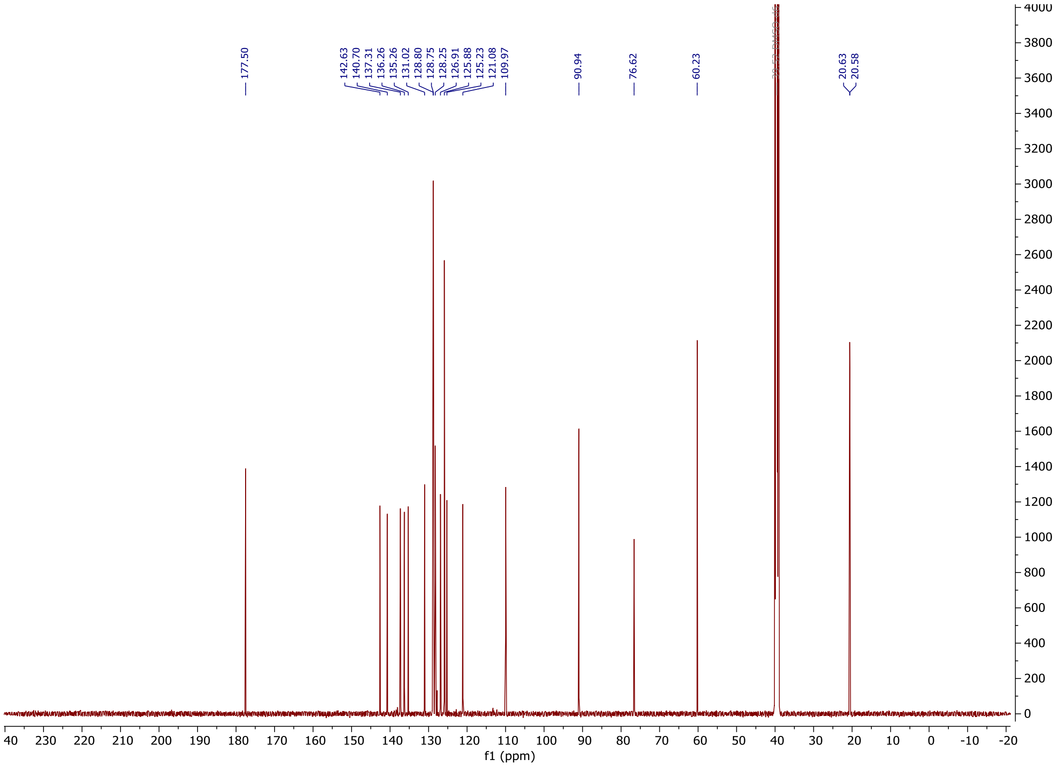

3',3'-Bis(4-(*tert*-butyl)phenyl)spiro[indoline-3,2'-oxetan]-2-one (*rac*-5c)

^{1}H-NMR (500 MHz, DMSO-d_6, 300 K)

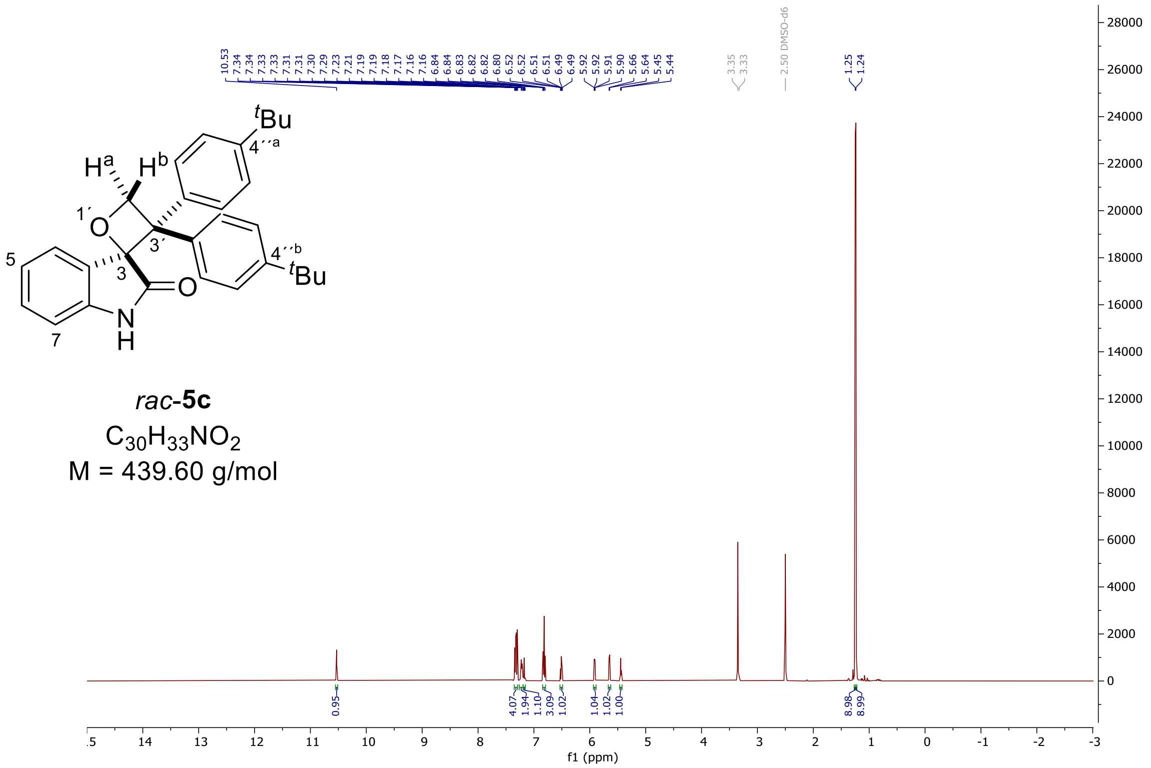

^{13}C-NMR (125 MHz, DMSO-d_6, 300 K)

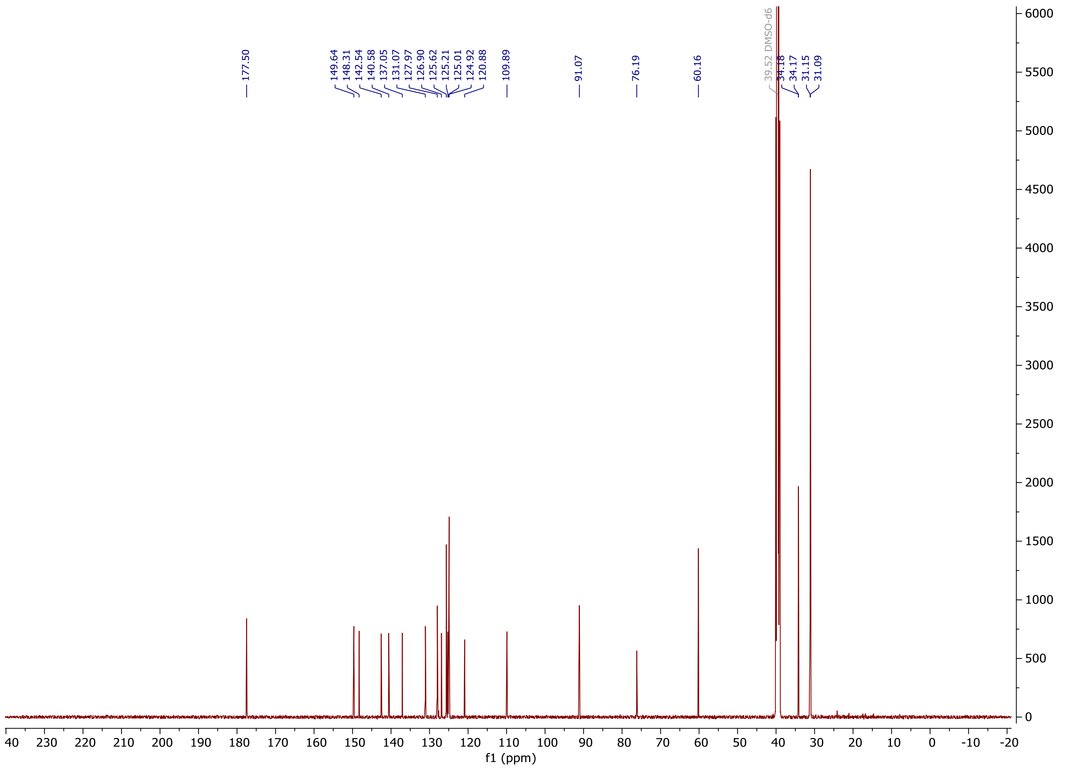

3',3'-Bis(3,5-di-*tert*-butylphenyl)spiro[indoline-3,2'-oxetan]-2-one (*rac*-5d)

^{1}H-NMR (500 MHz, DMSO-d_6, 300 K)

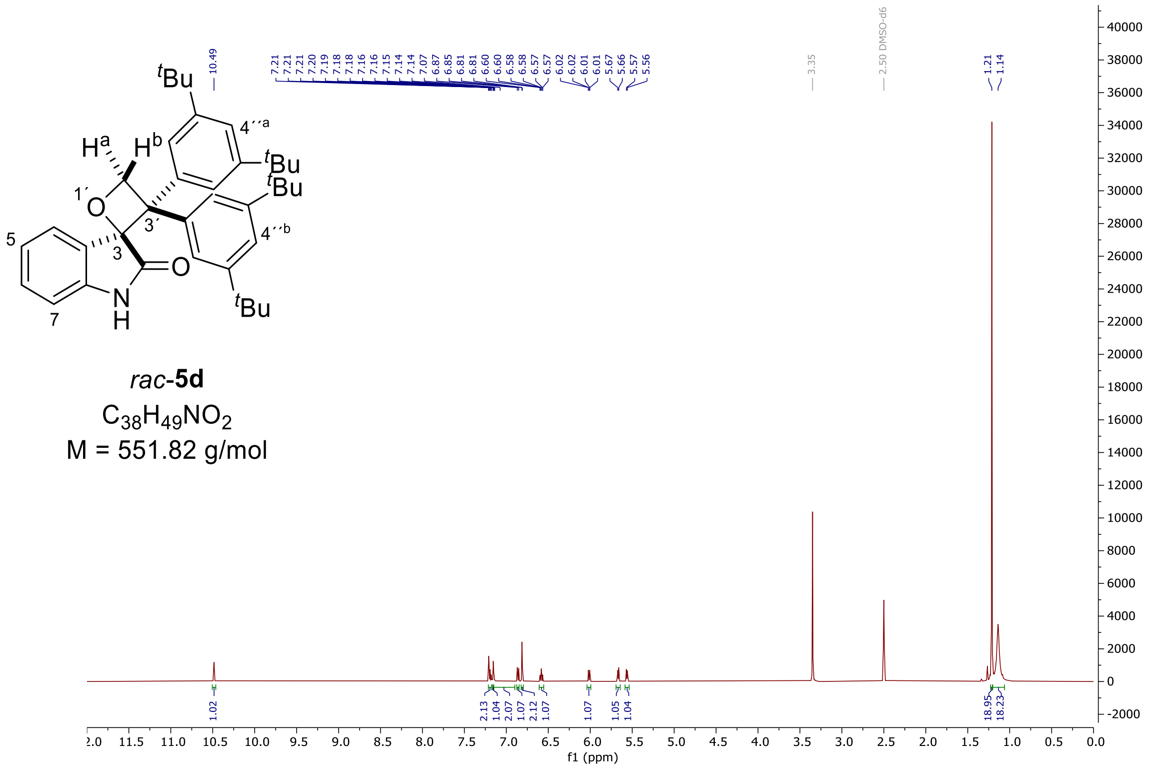

^{13}C-NMR (125 MHz, DMSO-d_6, 300 K)

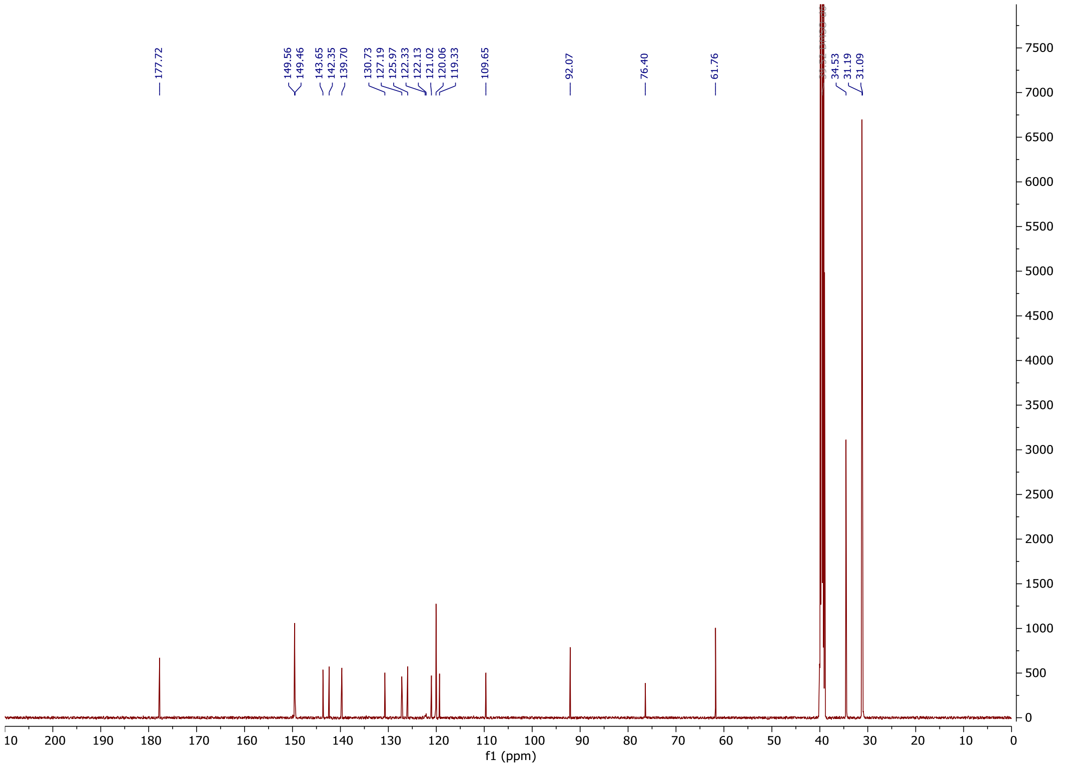

3',3'-Diphenylspiro[indoline-3,2'-oxetan]-2-one (*rac*-5e)

^{1}H-NMR (500 MHz, DMSO-*d*$_6$, 300 K)

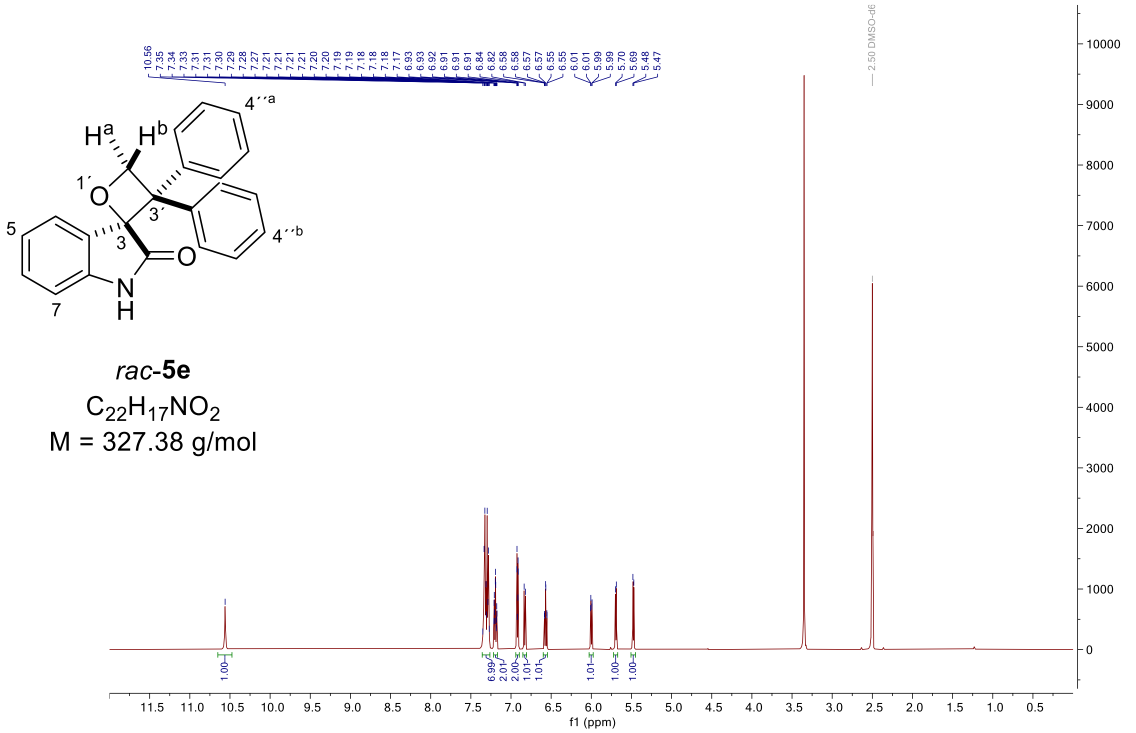

^{13}C-NMR (125 MHz, DMSO-*d*$_6$, 300 K)

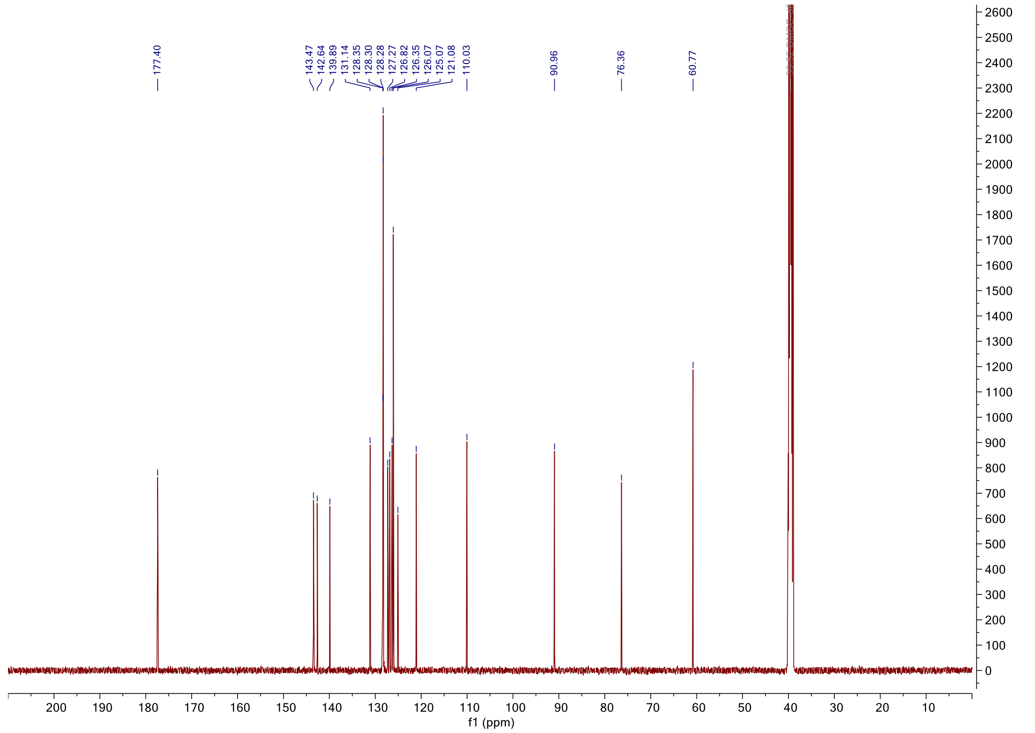

3',3'-Bis(4-fluorophenyl)spiro[indoline-3,2'-oxetan]-2-one (*rac*-5f)

^{1}H-NMR (500 MHz, DMSO-d_6, 300 K)

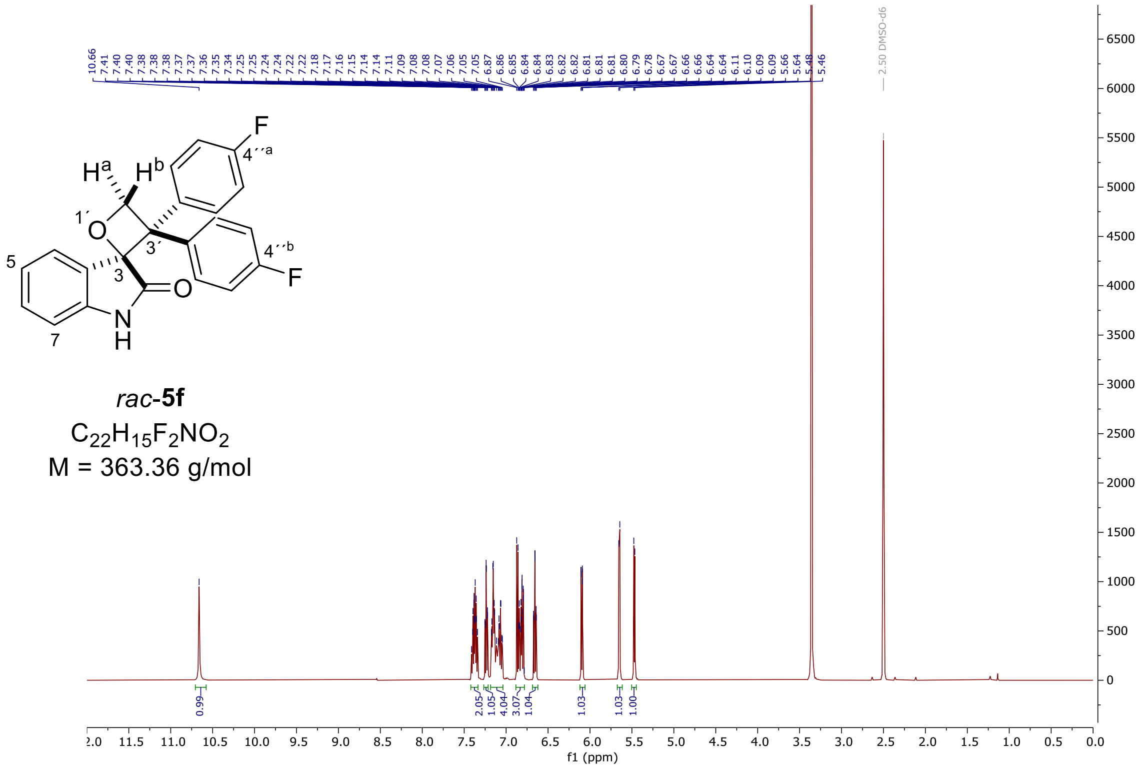

^{13}C-NMR (125 MHz, DMSO-d_6, 300 K)

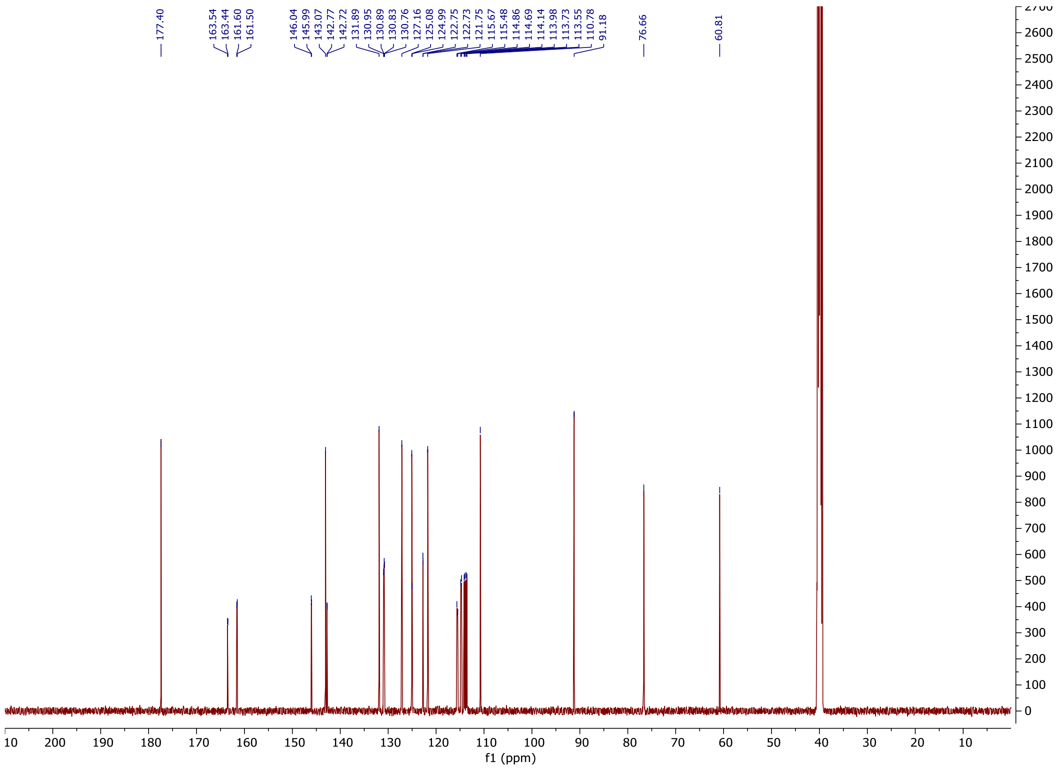

^{19}F-NMR (376 MHz, DMSO-d_6, 300 K)

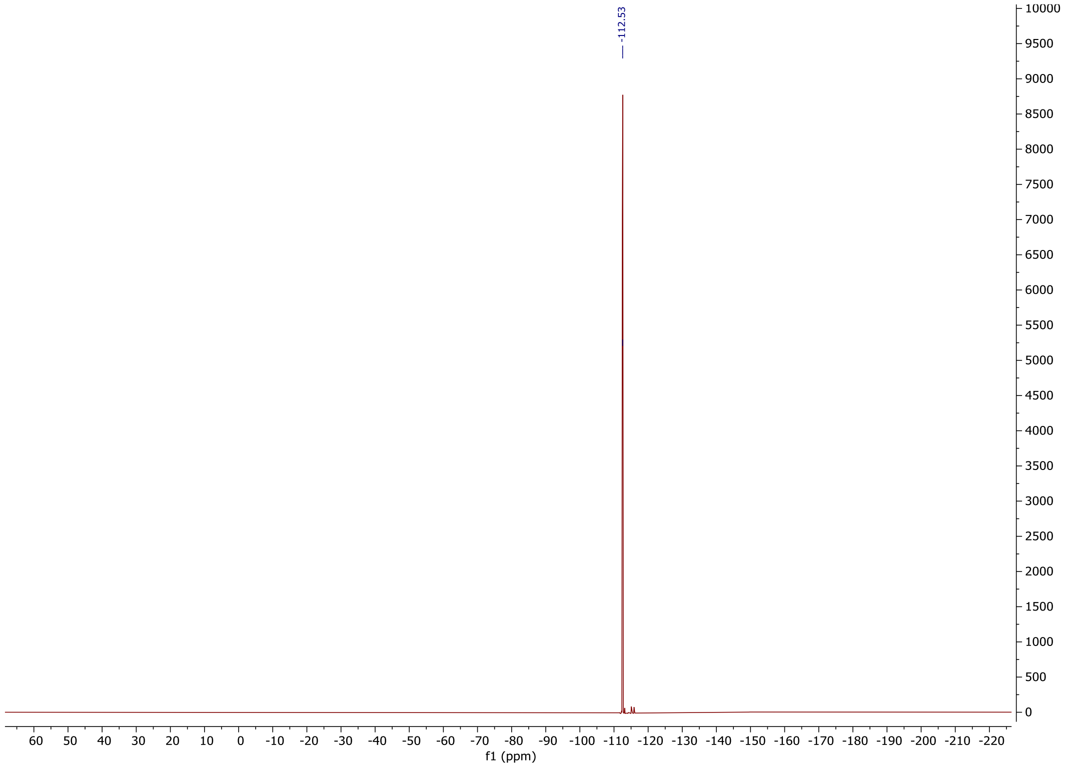

3',3'-Bis(4-bromophenyl)spiro[indoline-3,2'-oxetan]-2-one (*rac*-5g)

^{1}H-NMR (500 MHz, DMSO-d_6, 300 K)

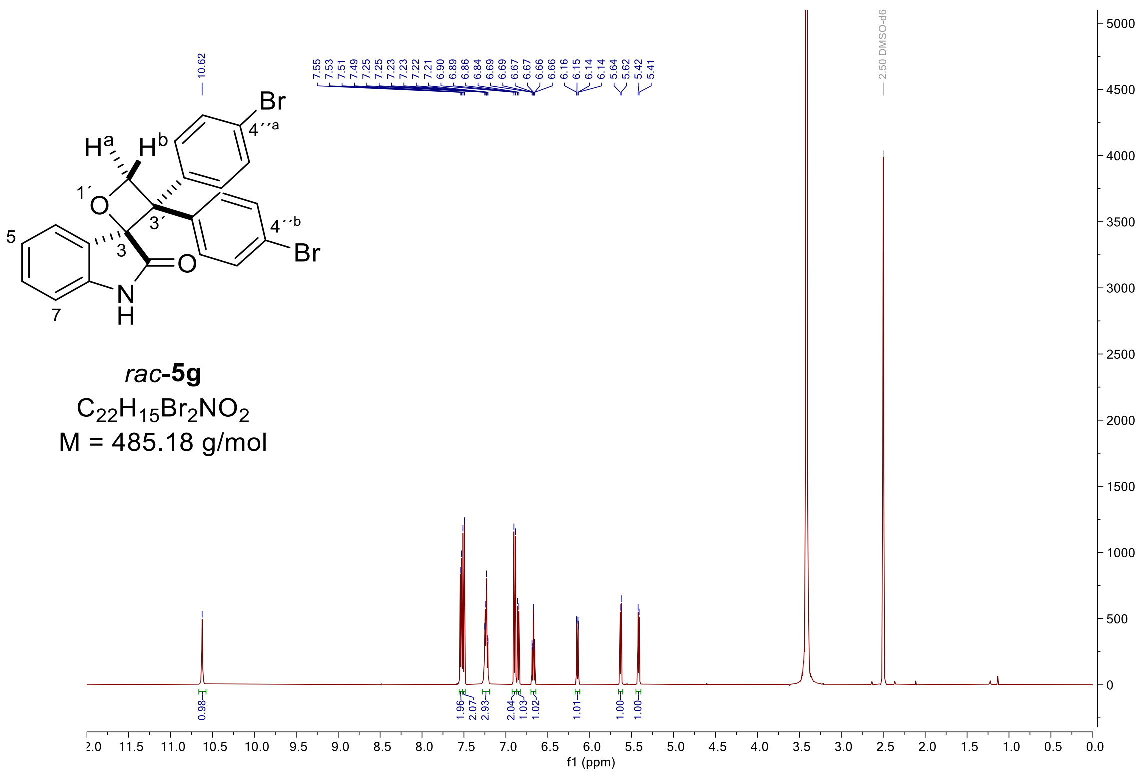

^{13}C-NMR (125 MHz, DMSO-d_6, 300 K)

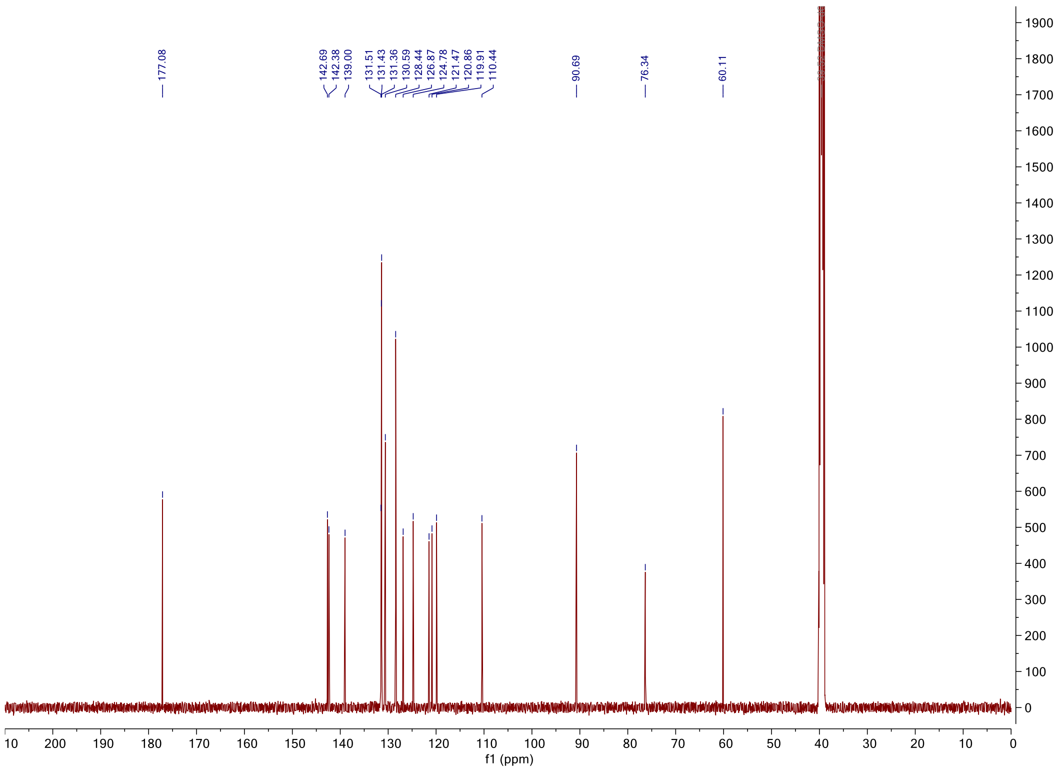

3',3'-bis(3,5-Bis(trifluoromethyl)phenyl)spiro[indoline-3,2'-oxetan]-2-one (*rac*-5h)

^{1}H-NMR (500 MHz, DMSO-d_6, 300 K)

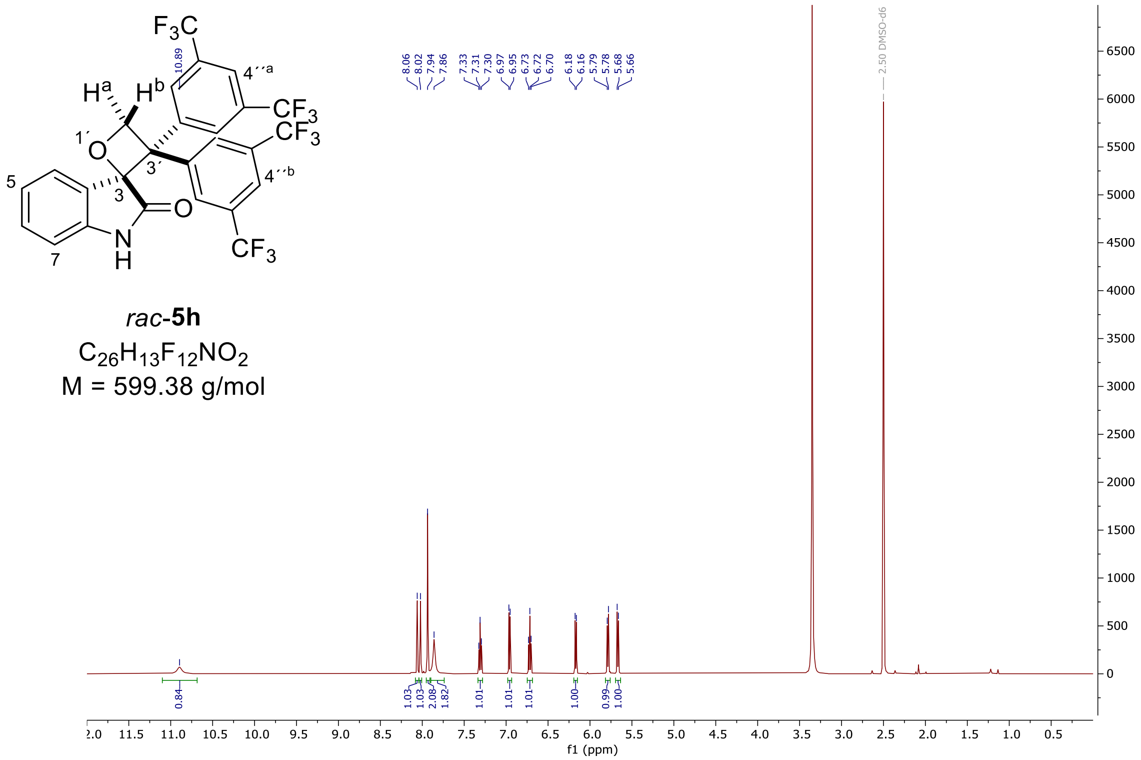

rac-**5h**

$C_{26}H_{13}F_{12}NO_2$

M = 599.38 g/mol

^{13}C-NMR (125 MHz, DMSO-d_6, 300 K)

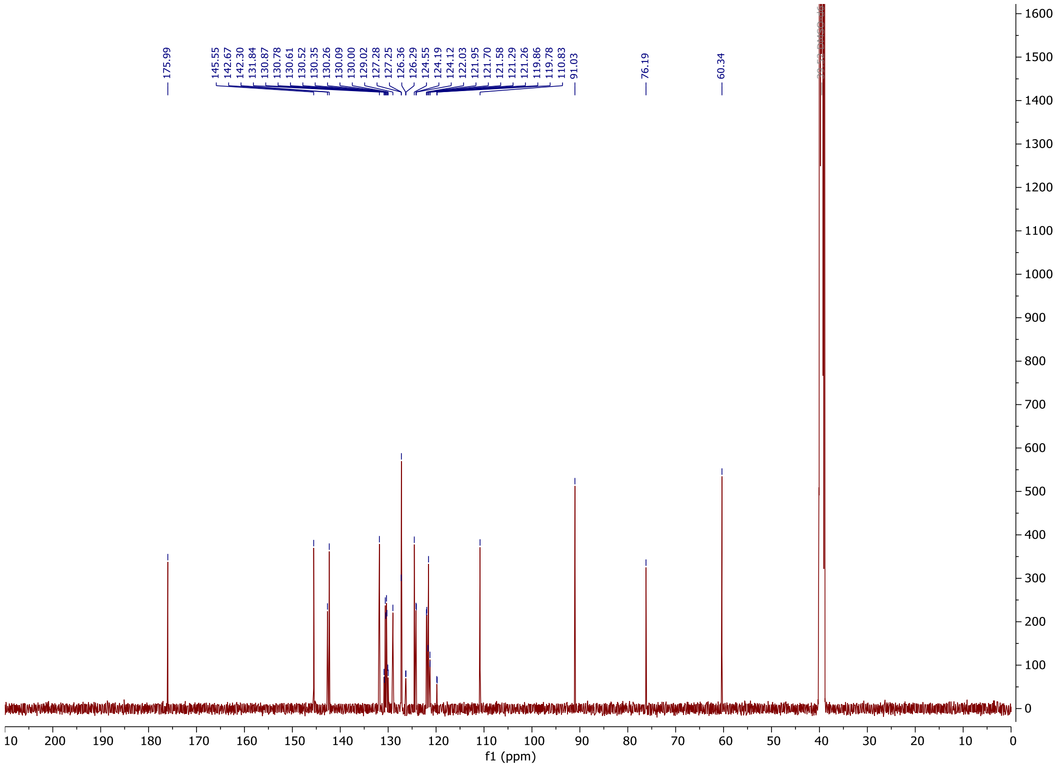

^{19}F-NMR (376 MHz, DMSO-d_6, 300 K)

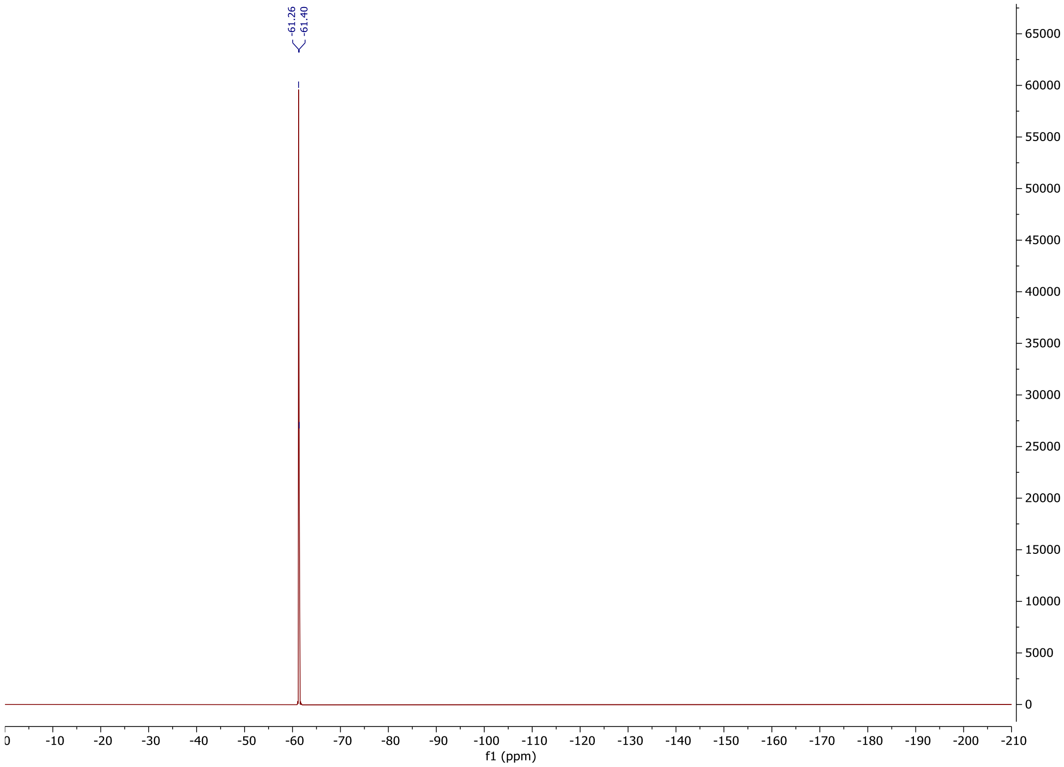

3',3'-Bis(3-nitrophenyl)spiro[indoline-3,2'-oxetan]-2-one (*rac*-5i)

^{1}H-NMR (500 MHz, DMSO-d_6, 300 K)

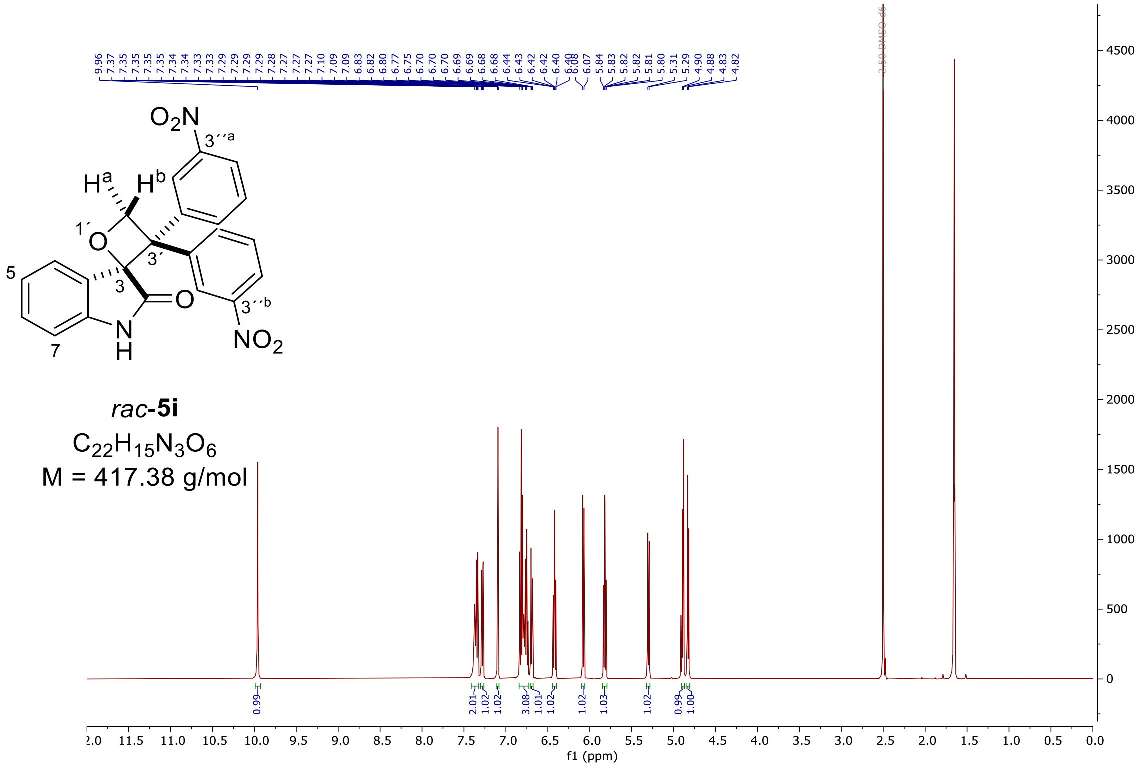

^{13}C-NMR (125 MHz, DMSO-d_6, 300 K)

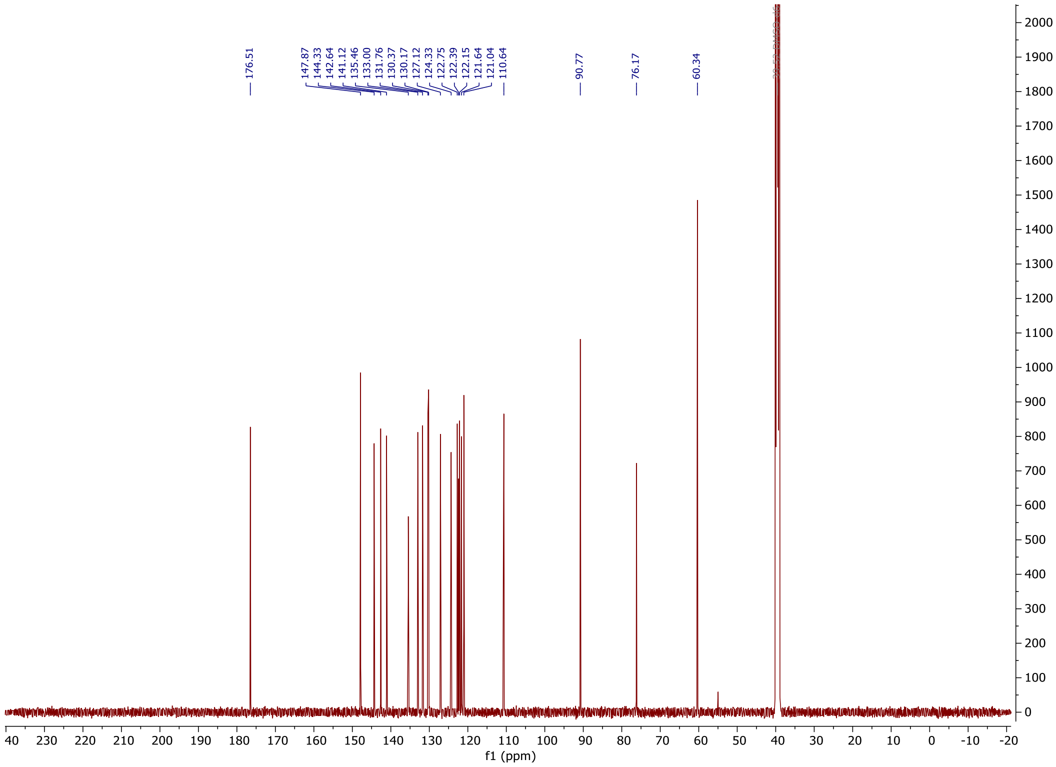

(2-Oxospiro[indoline-3,2'-oxetane]-3',3'-diyl)-bis(4,1-phenylene) bis(4-methylbenzene-sulfonate) (*rac*-5j)

^{1}H-NMR (500 MHz, DMSO-d_6, 300 K)

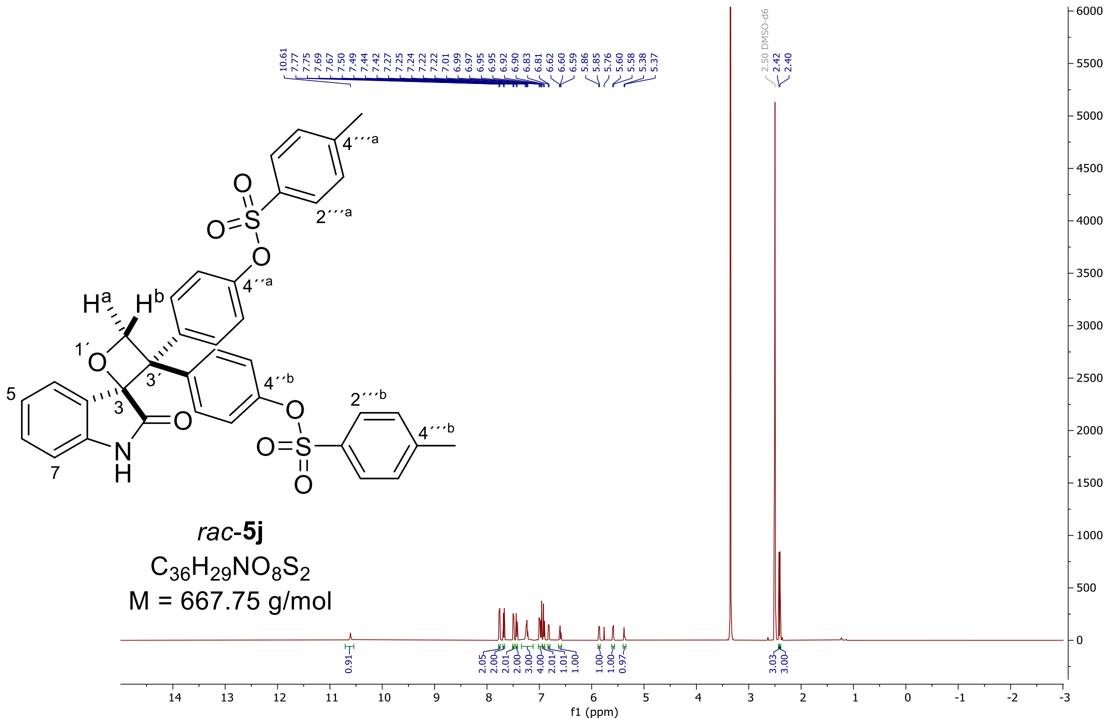

^{13}C-NMR (125 MHz, DMSO-d_6, 300 K)

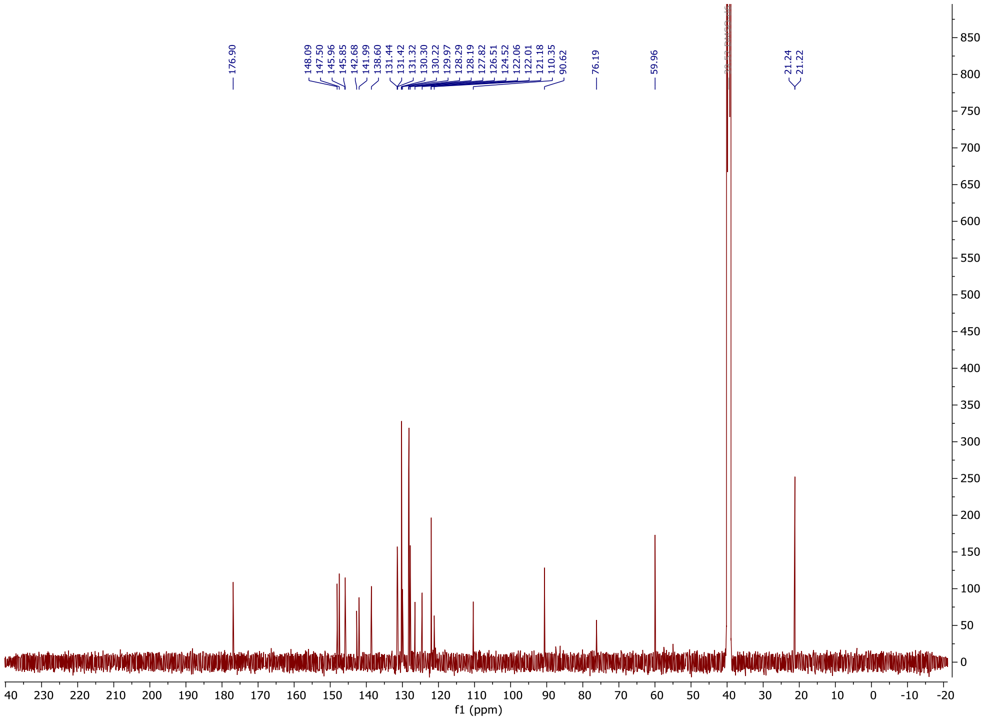

2-Oxospiro[indoline-3,2'-oxetane]-3',3'-diyl)bis(4,1-phenylene) bis(2,2-dimethylpropanoate (*rac*-5k)

^{1}H-NMR (500 MHz, DMSO-d_6, 300 K)

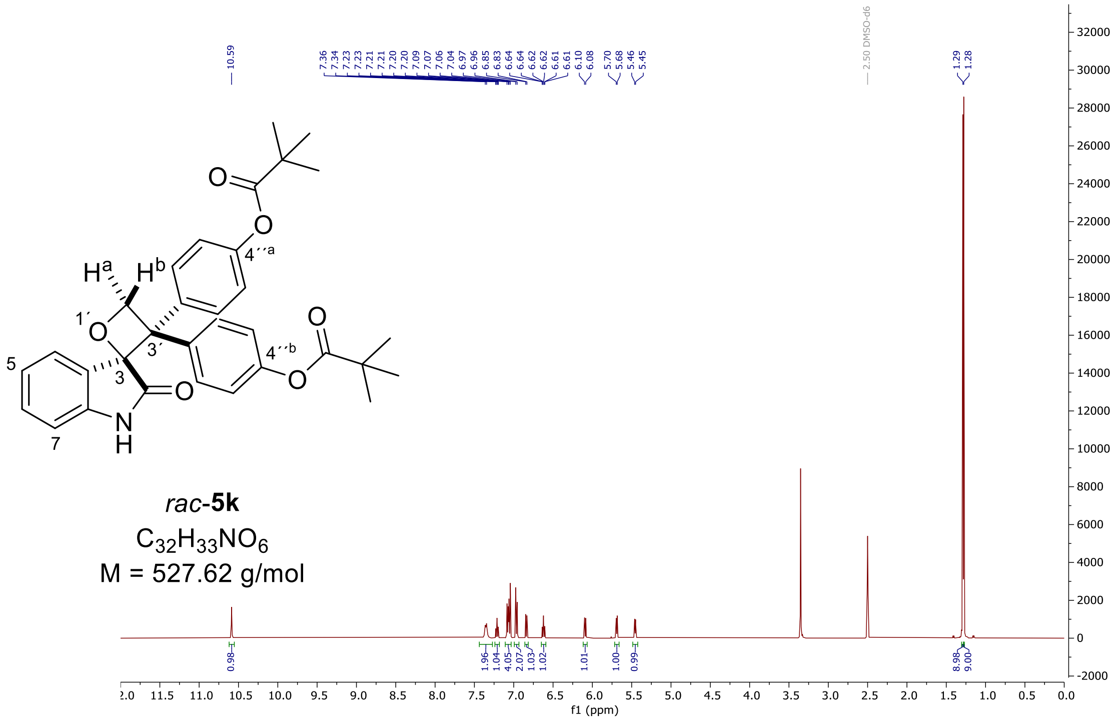

^{13}C-NMR (125 MHz, DMSO-d_6, 300 K)

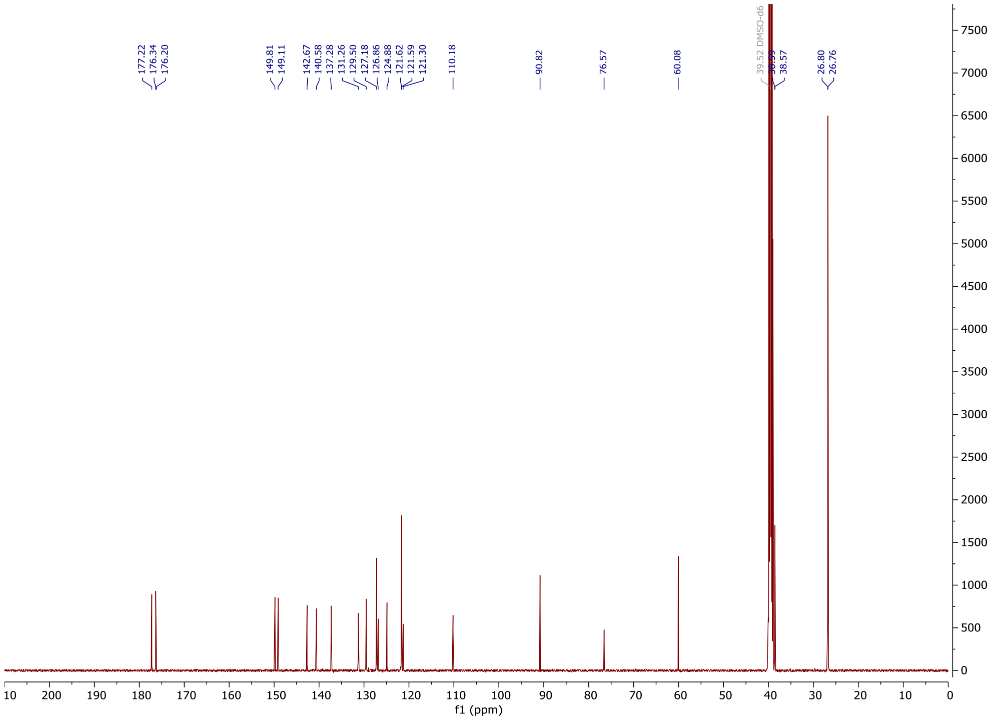

5-Fluoro-3',3'-bis(4-fluorophenyl)spiro[indoline-3,2'-oxetan]-2-one (*rac*-5l)

^{1}H-NMR (500 MHz, DMSO-d_6, 300 K)

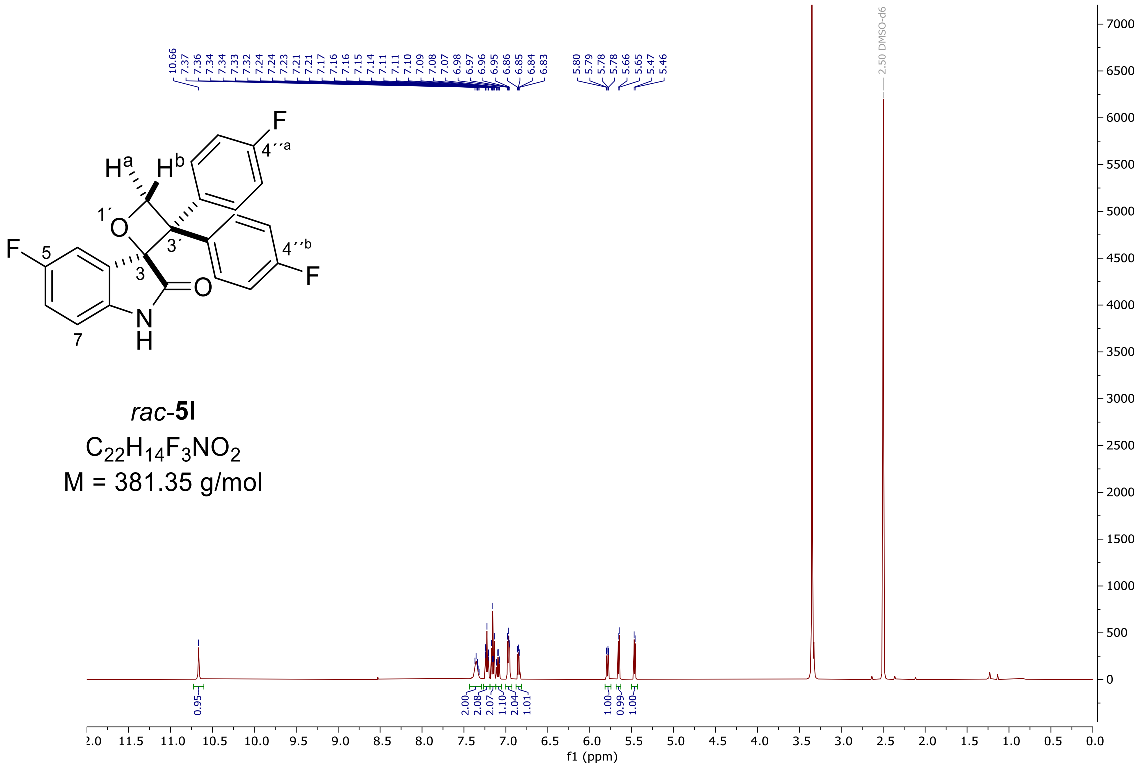

^{13}C-NMR (125 MHz, DMSO-d_6, 300 K)

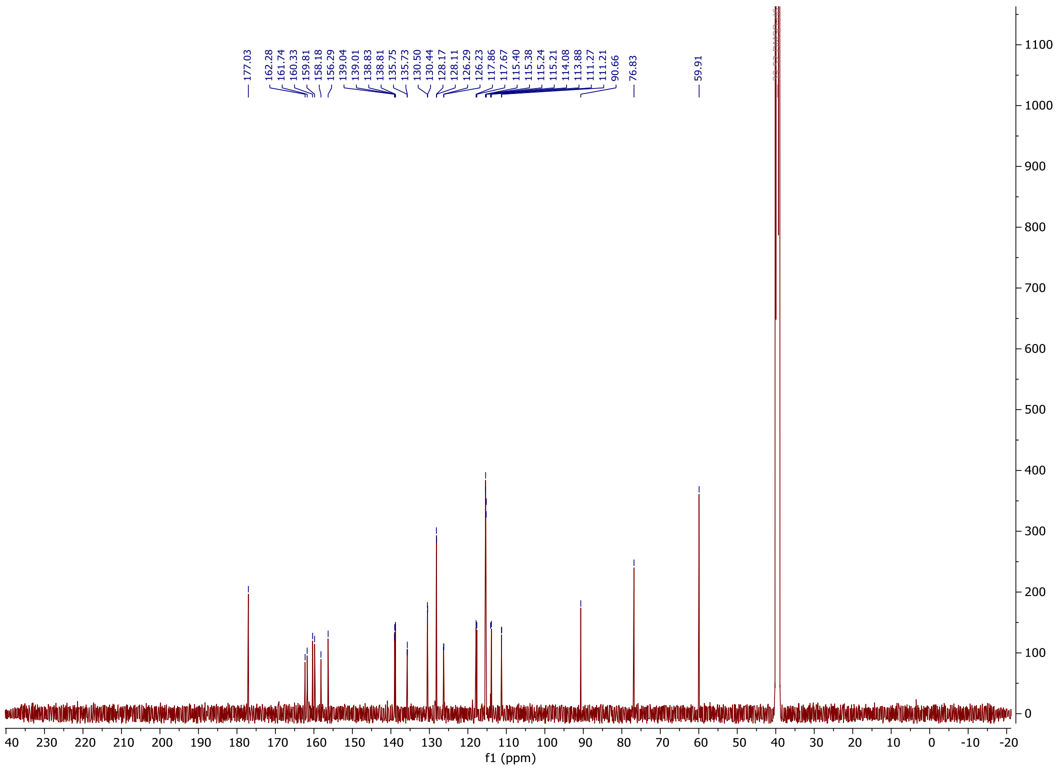

^{19}F-NMR (470 MHz, DMSO-d_6, 300 K)

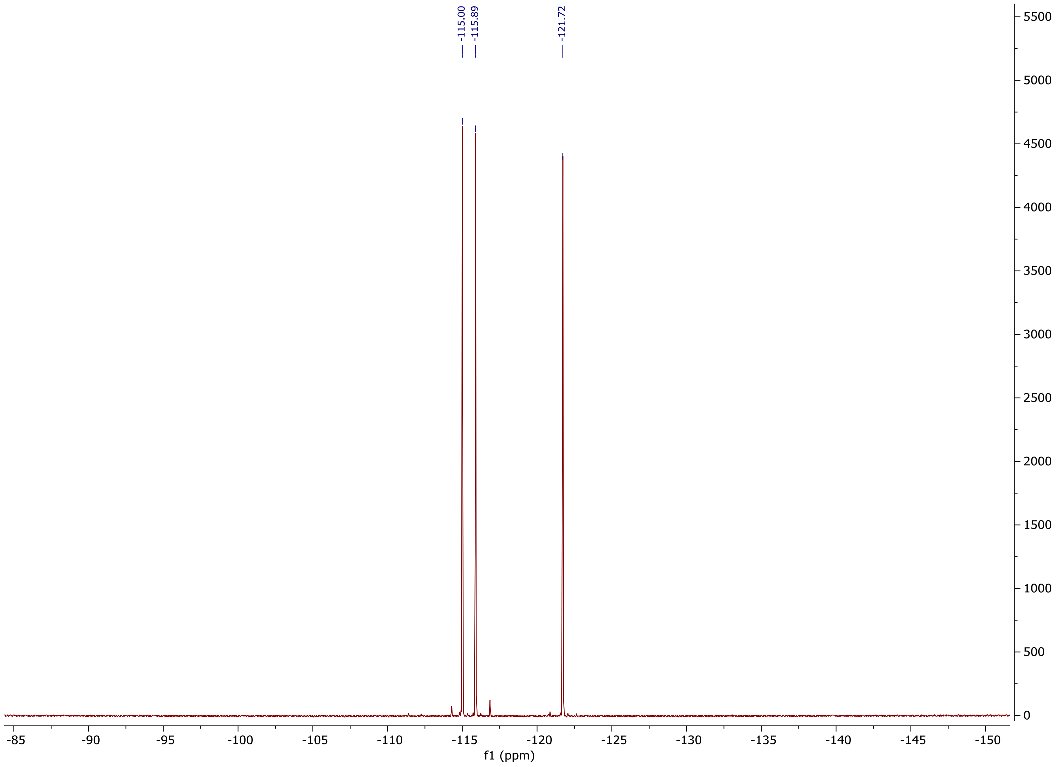

5-Fluoro-3',3'-di-*p*-tolylspiro[indoline-3,2'-oxetan]-2-one (5m)

^{1}H-NMR (500 MHz, DMSO-d_6, 300 K)

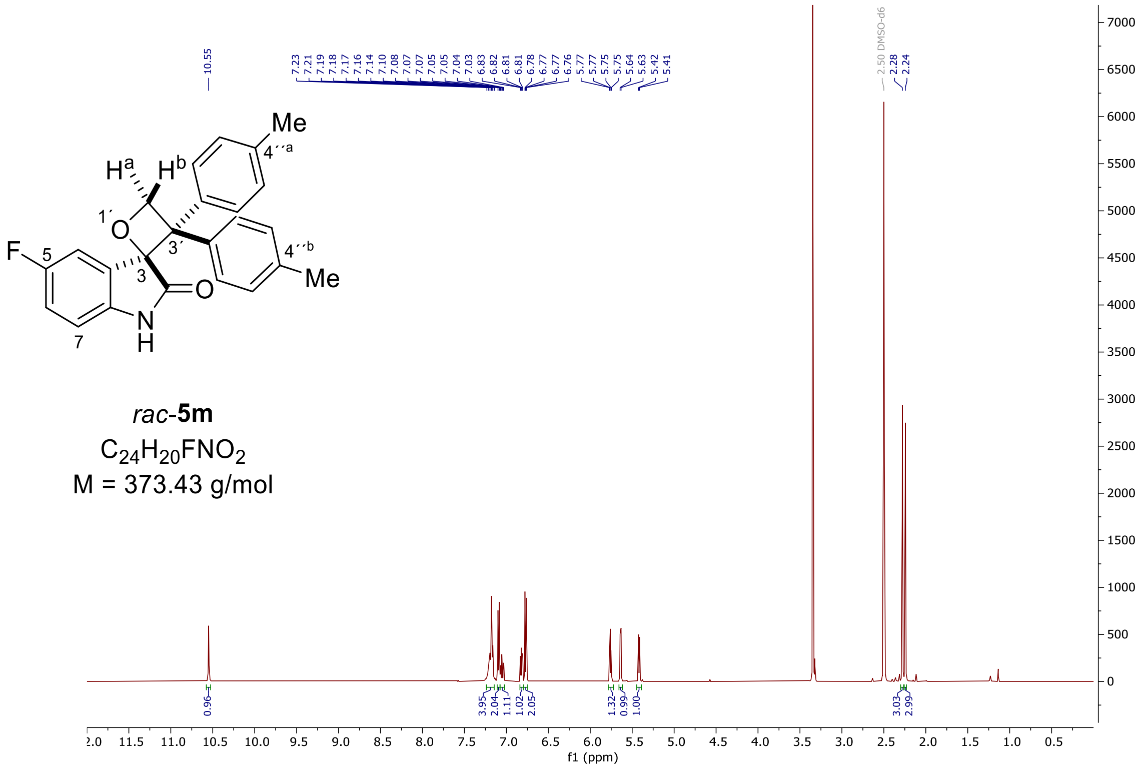

^{13}C-NMR (125 MHz, DMSO-d_6, 300 K)

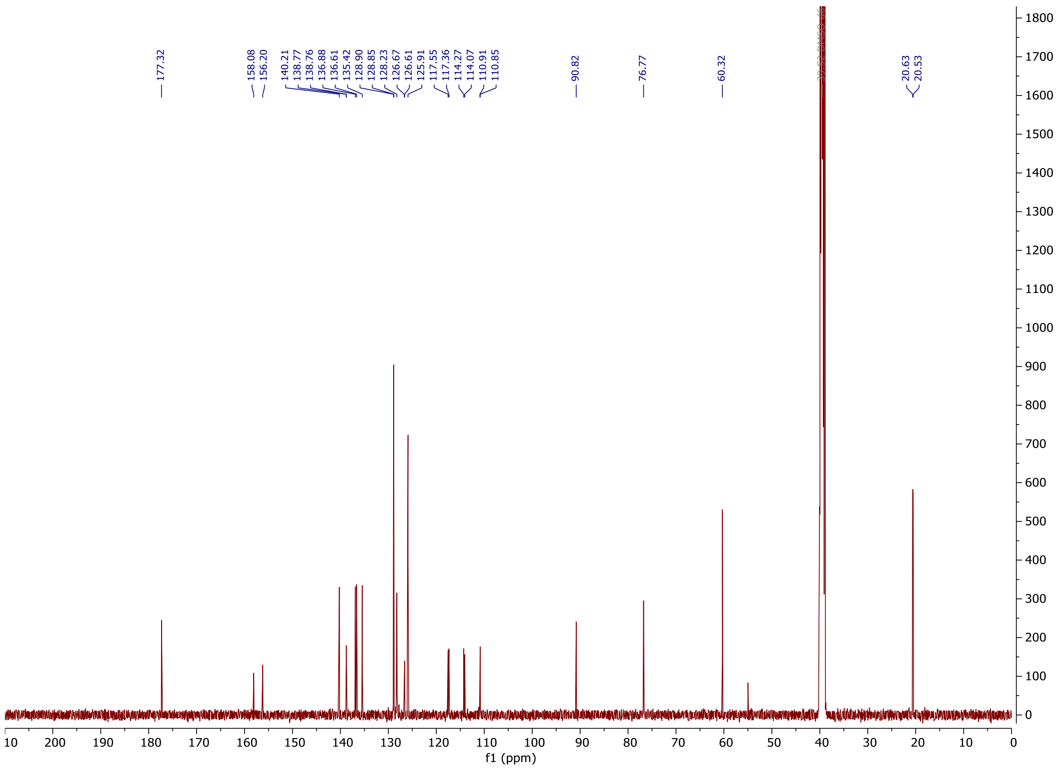

^{19}F-NMR (470 MHz, DMSO-d_6, 300 K)

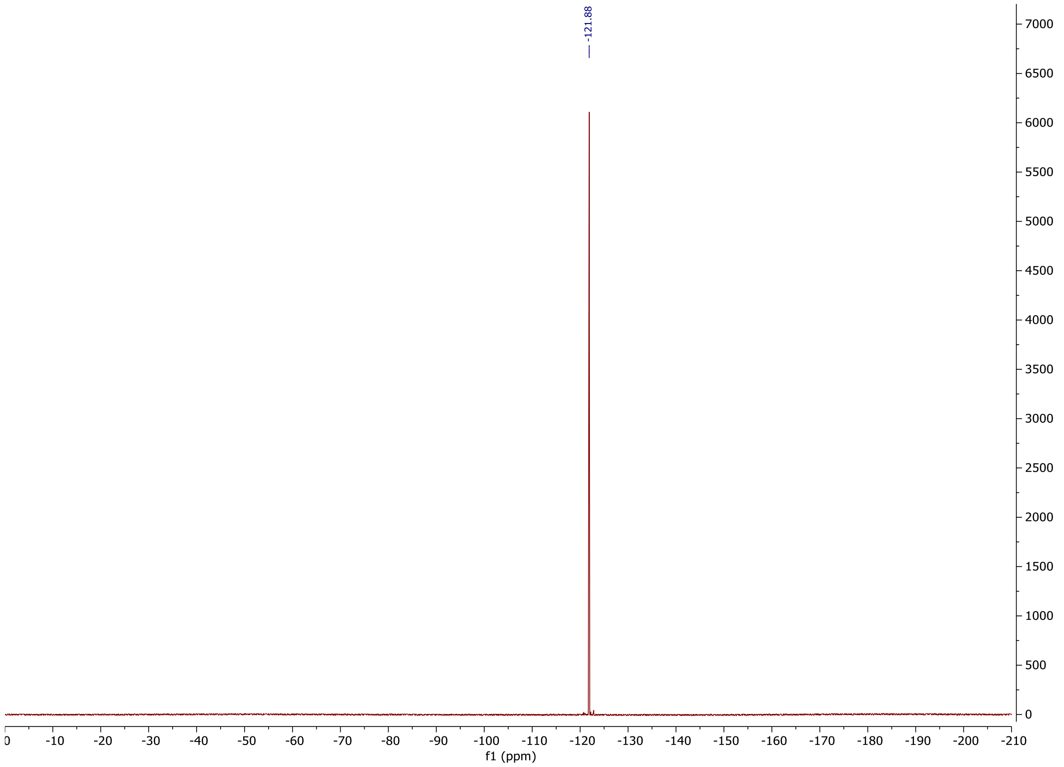

3',3'-Bis(4-chlorophenyl)-5-fluorospiro[indoline-3,2'-oxetan]-2-one (*rac*-5n)

^{1}H-NMR (500 MHz, DMSO-d_6, 300 K)

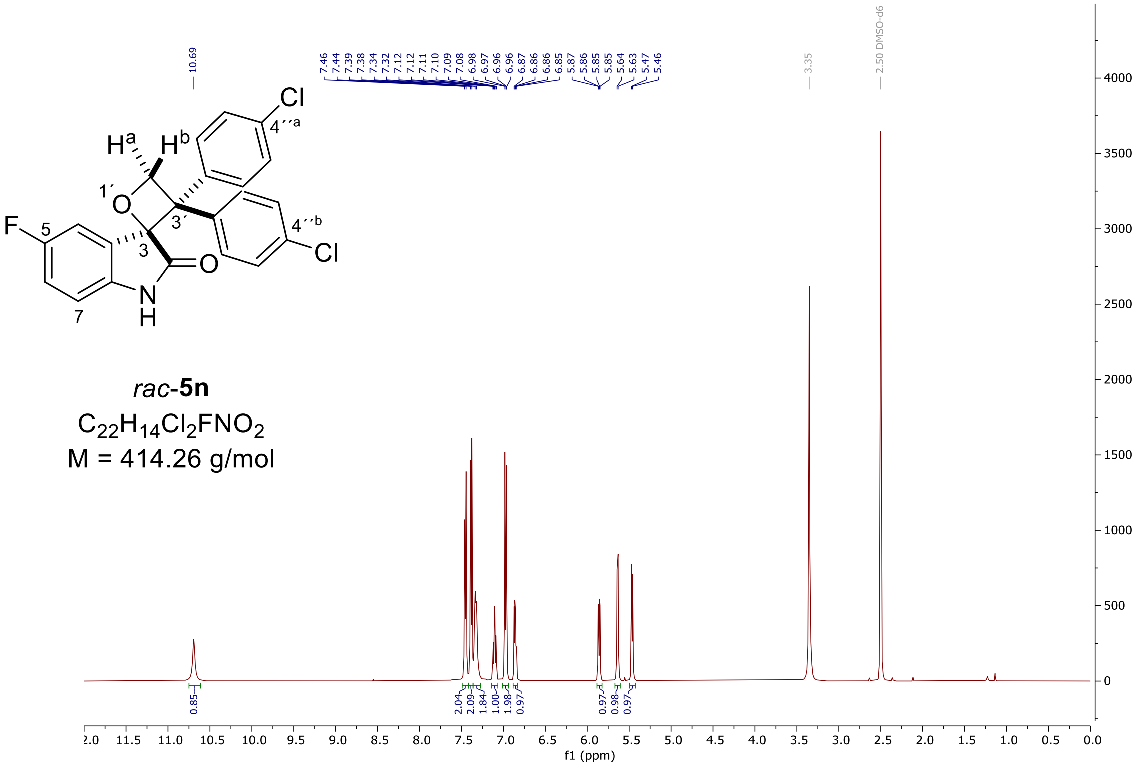

rac-**5n**

$C_{22}H_{14}Cl_2FNO_2$

M = 414.26 g/mol

^{13}C-NMR (125 MHz, DMSO-d_6, 300 K)

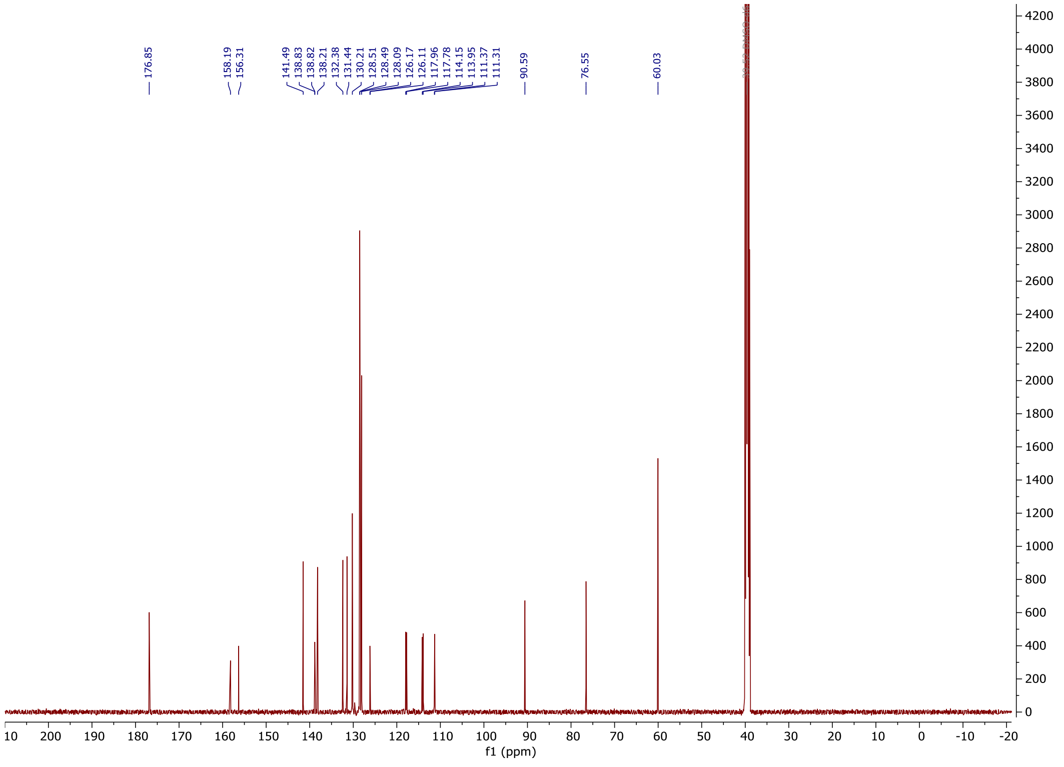

^{19}F-NMR (470 MHz, DMSO-d_6, 300 K)

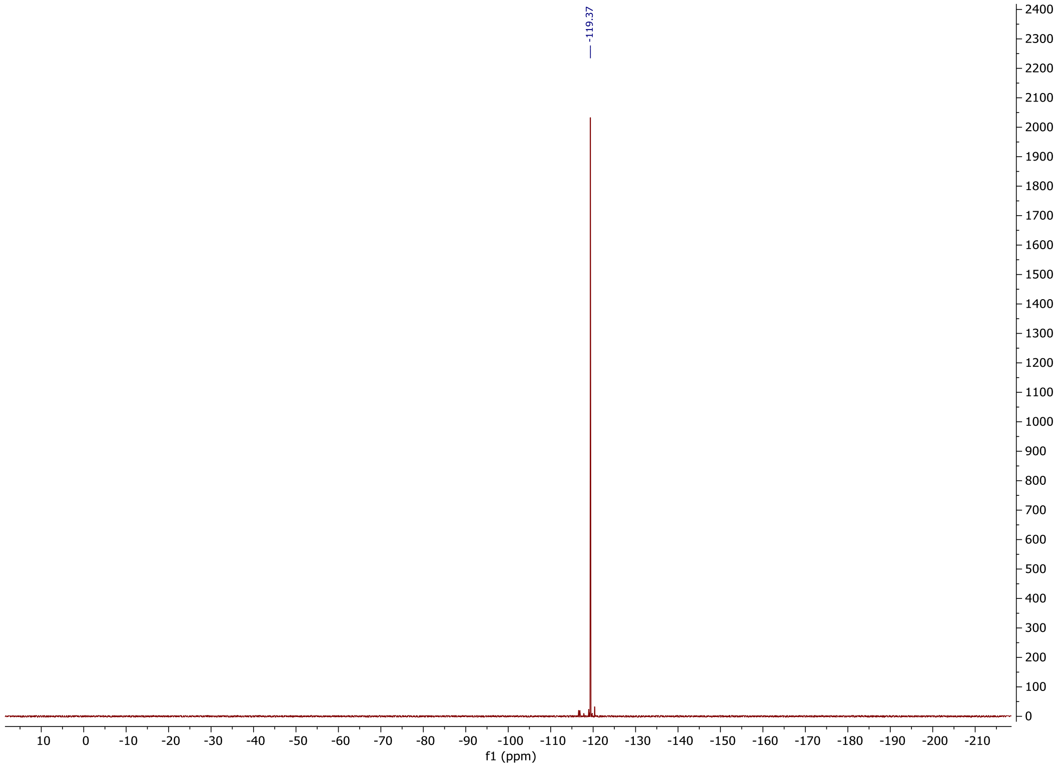

6-Chloro-3',3'-bis(4-chlorophenyl)spiro[indoline-3,2'-oxetan]-2-one (*rac*-5o)

^{1}H-NMR (500 MHz, DMSO-d_6, 300 K)

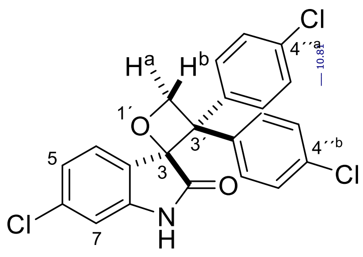

rac-**5o**

$C_{22}H_{14}Cl_3NO_2$

M = 430.71 g/mol

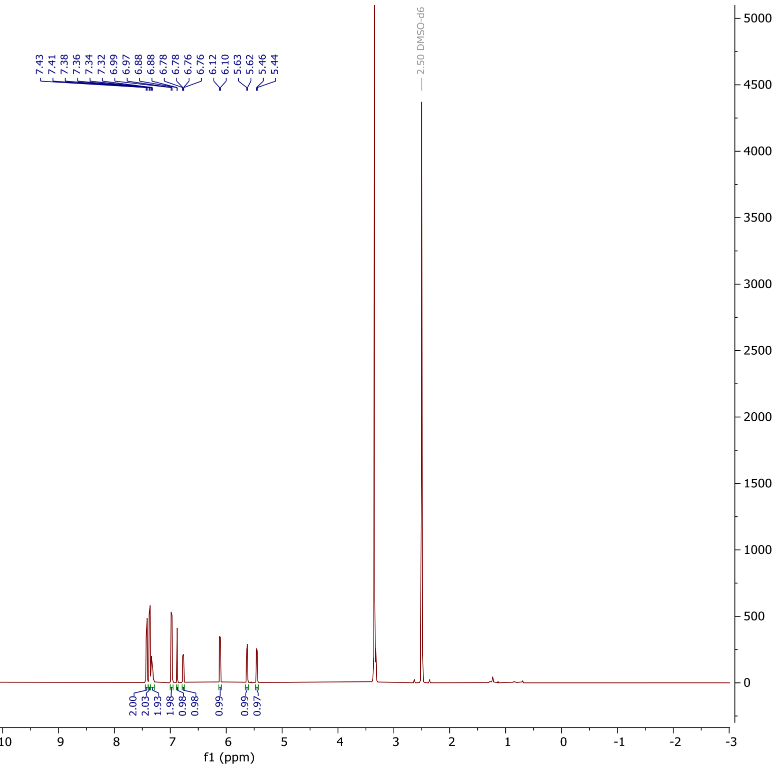

^{13}C-NMR (125 MHz, DMSO-d_6, 300 K)

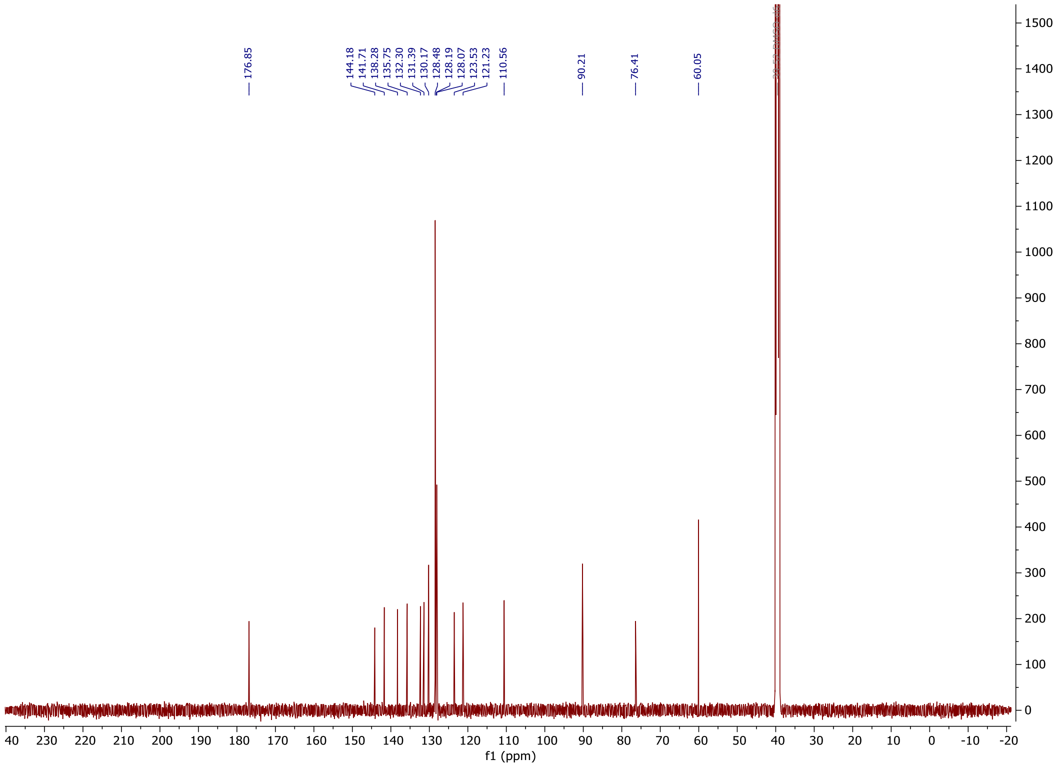

3-(Bis(4-chlorophenyl)methylene)indolin-2-one (7a)

^{1}H-NMR (500 MHz, DMSO-d_6, 300 K)

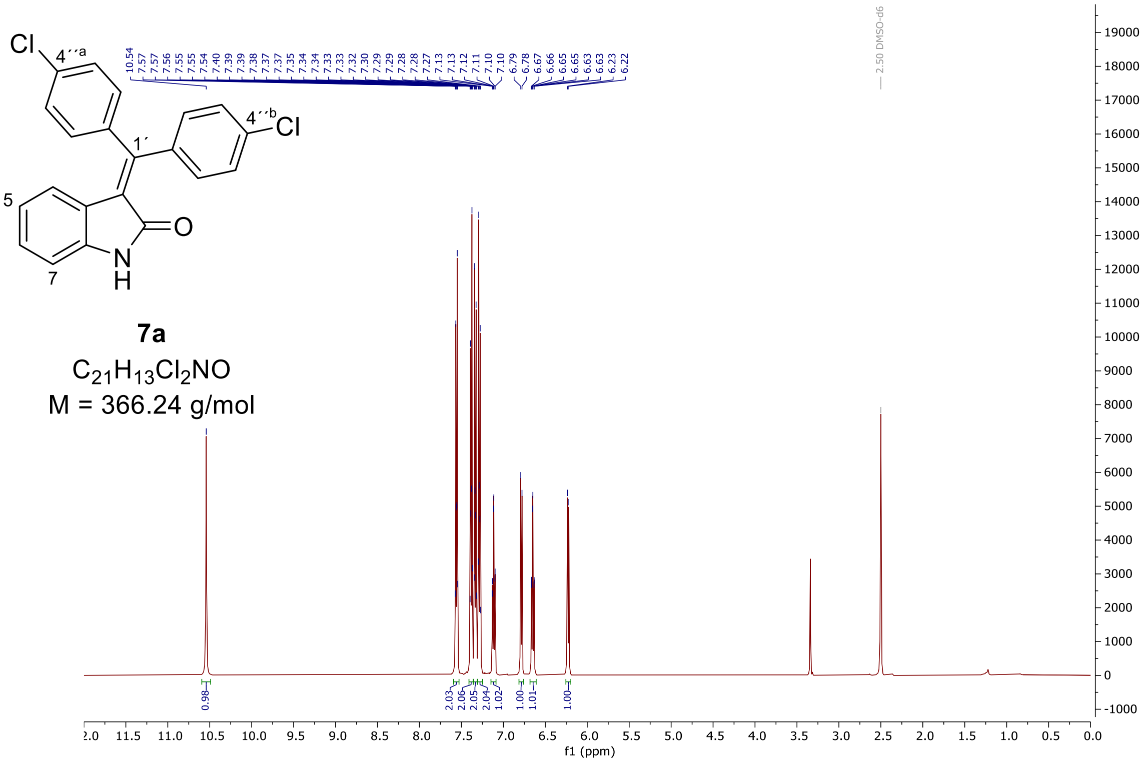

^{13}C-NMR (125 MHz, DMSO-d_6, 300 K)

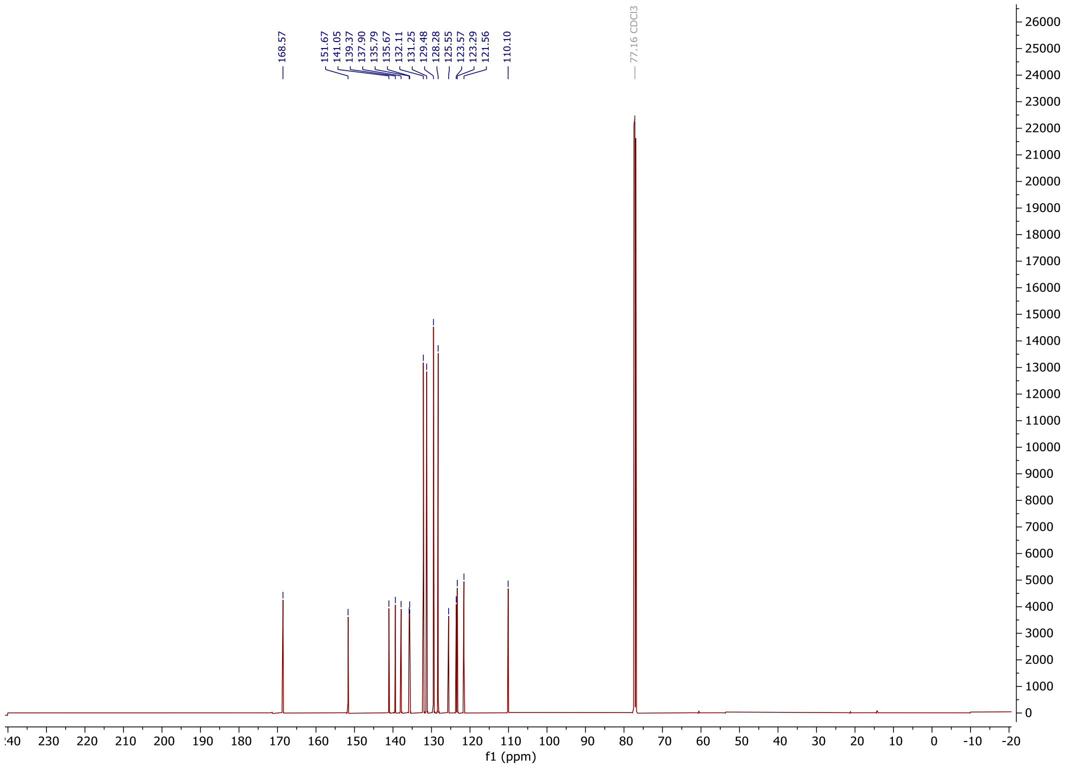

3-(Di-*p*-tolylmethylene)indolin-2-one (7b)

^{1}H-NMR (500 MHz, $CDCl_3$, 300 K)

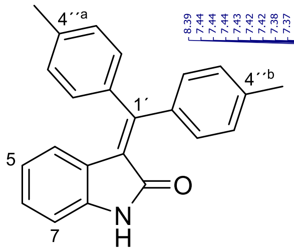

7b

$C_{23}H_{19}NO$

M = 325.4 g/mol

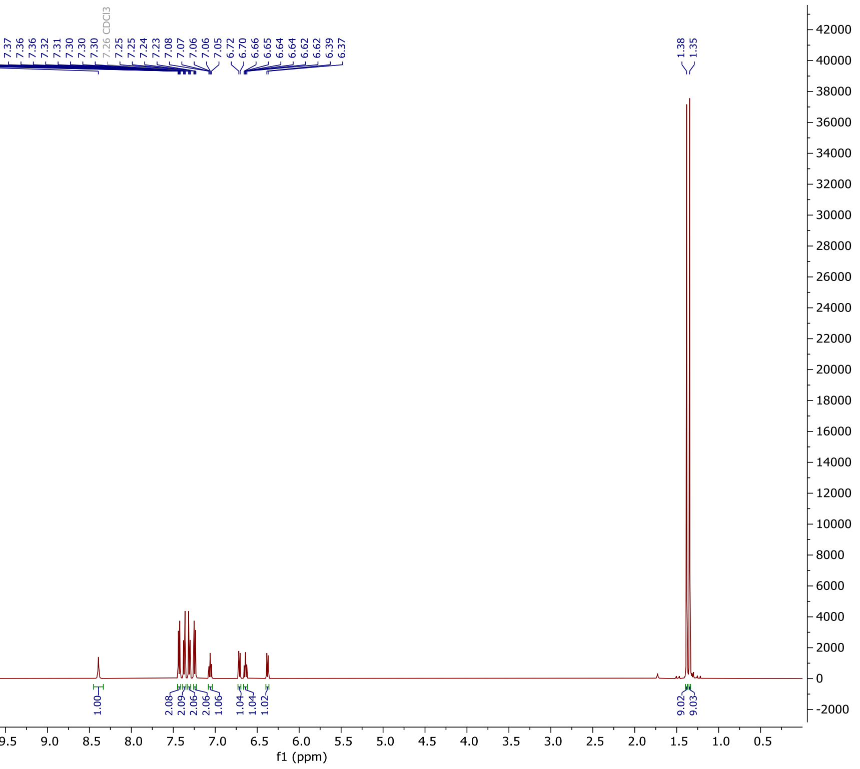

^{13}C-NMR (125 MHz, $CDCl_3$, 300 K)

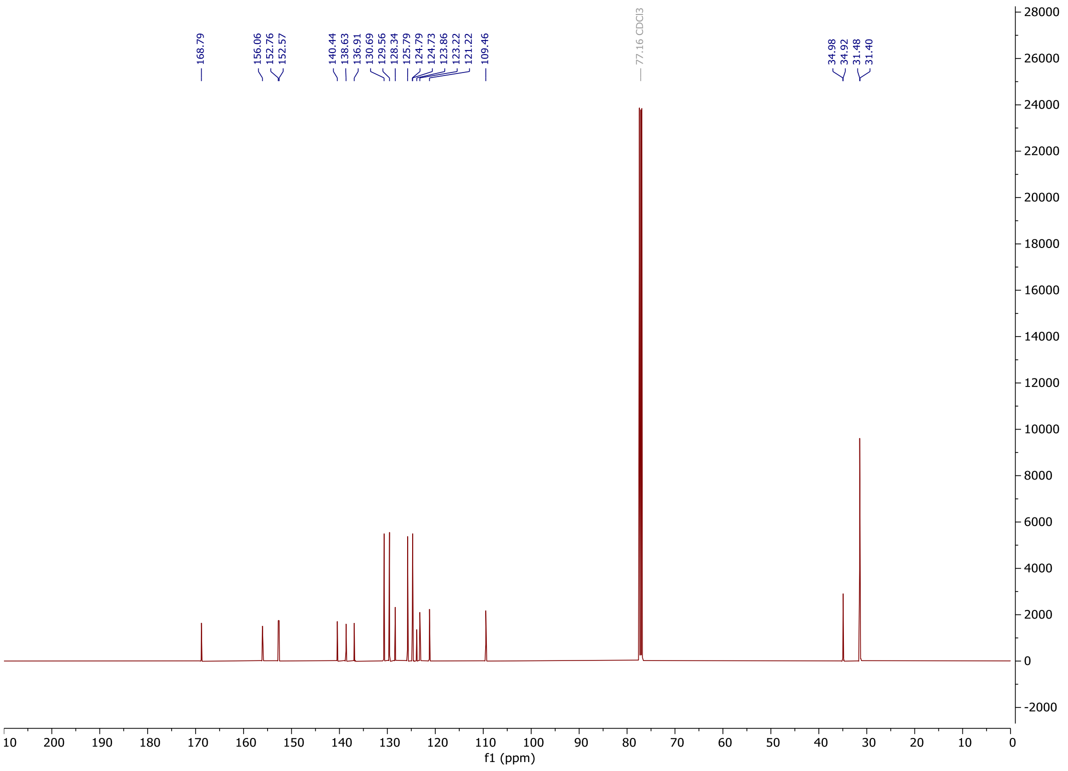

3-(Bis(4-(*tert*-butyl)phenyl)methylene)indolin-2-one (7c)

^{1}H-NMR (500 MHz, $CDCl_3$, 300 K)

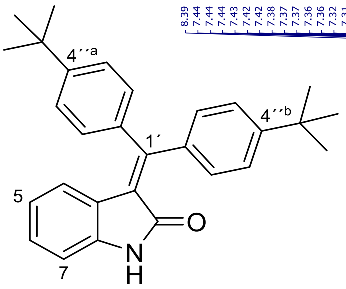

7c

$C_{29}H_{31}NO$

M = 409.57 g/mol

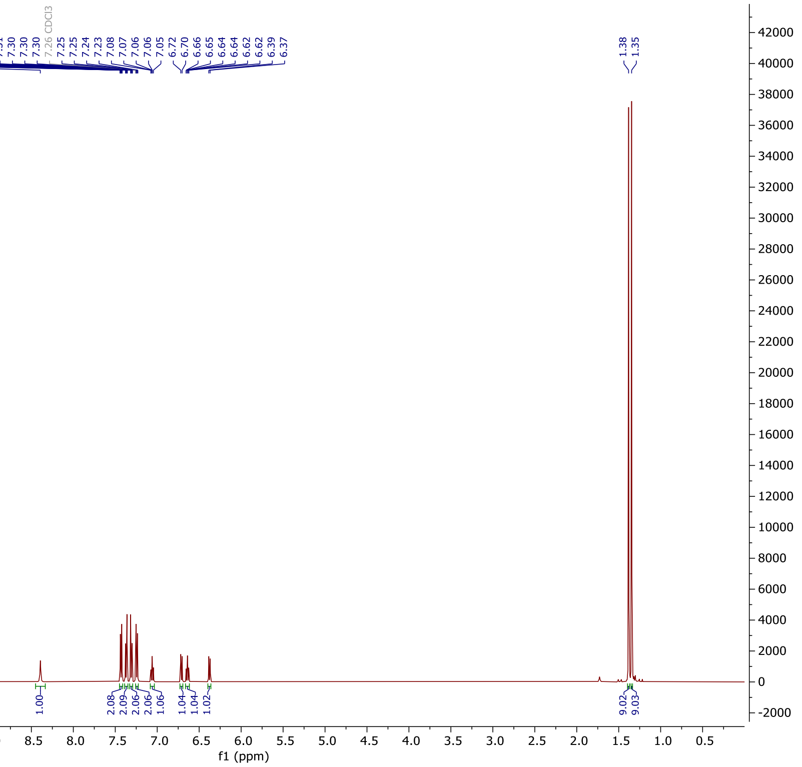

^{13}C-NMR (125 MHz, $CDCl_3$, 300 K)

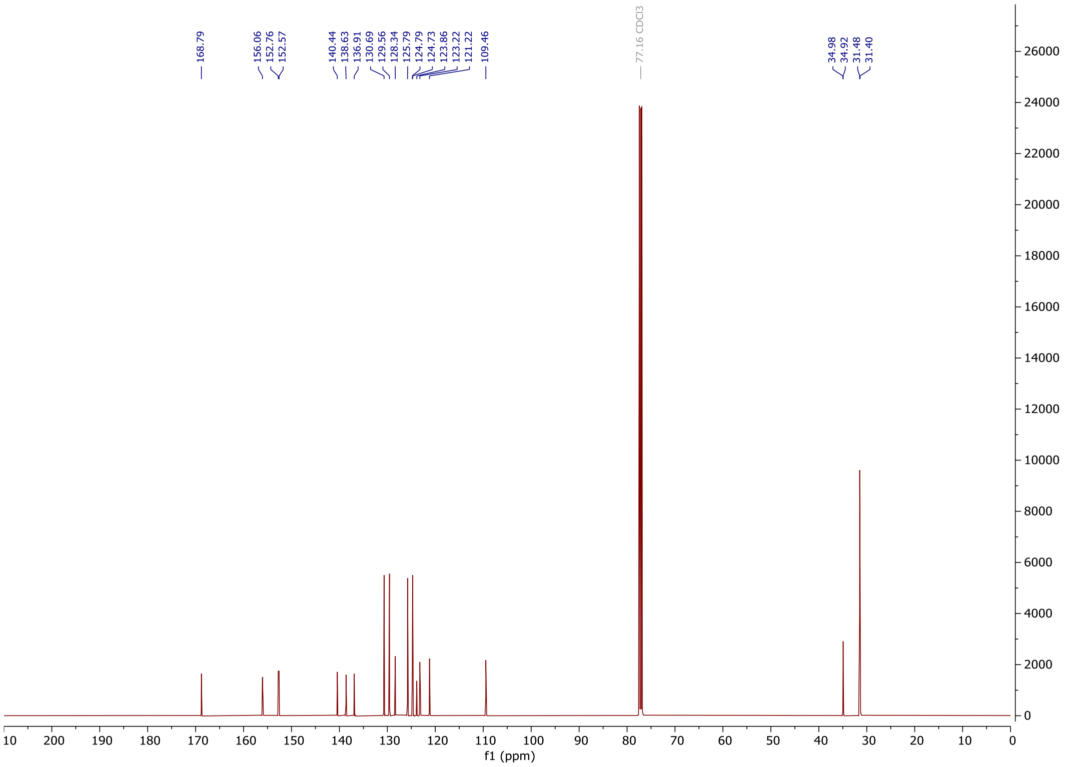

3-(Bis(3,5-di-*tert*-butylphenyl)methylene)indolin-2-one (7d)

^{1}H-NMR (500 MHz, $CDCl_3$, 300 K)

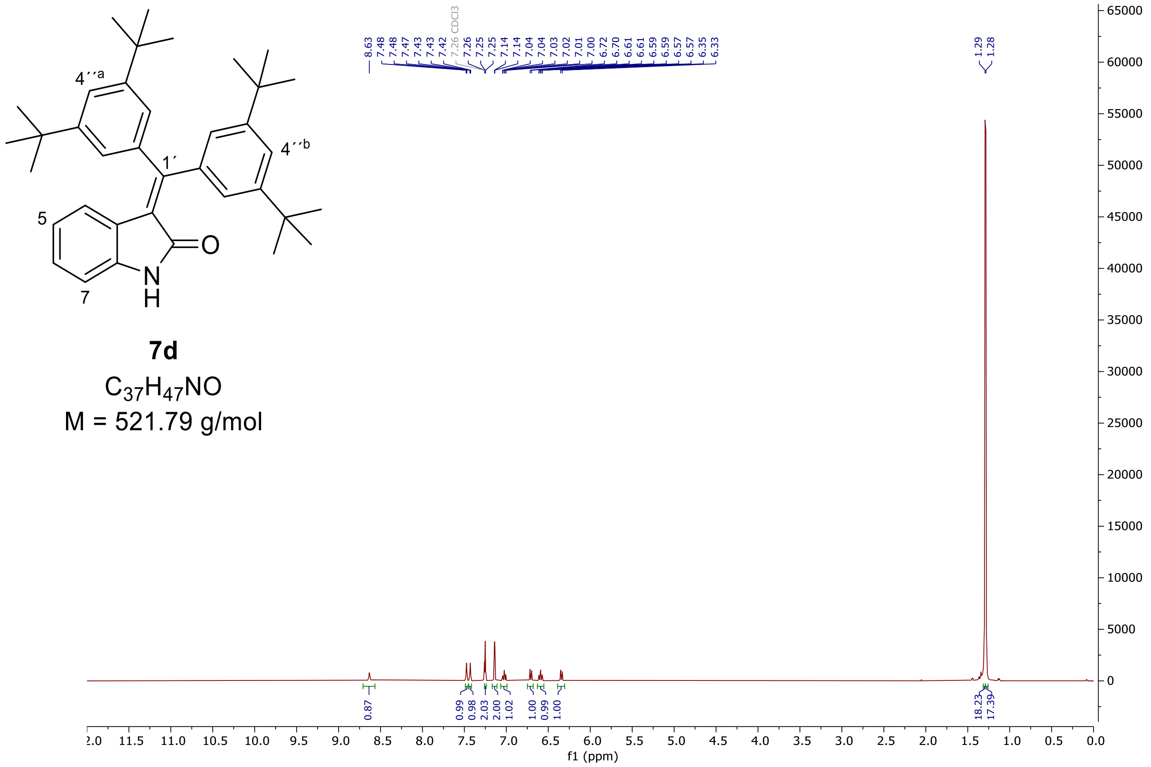

7d

$C_{37}H_{47}NO$

M = 521.79 g/mol

^{13}C-NMR (125 MHz, $CDCl_3$, 300 K)

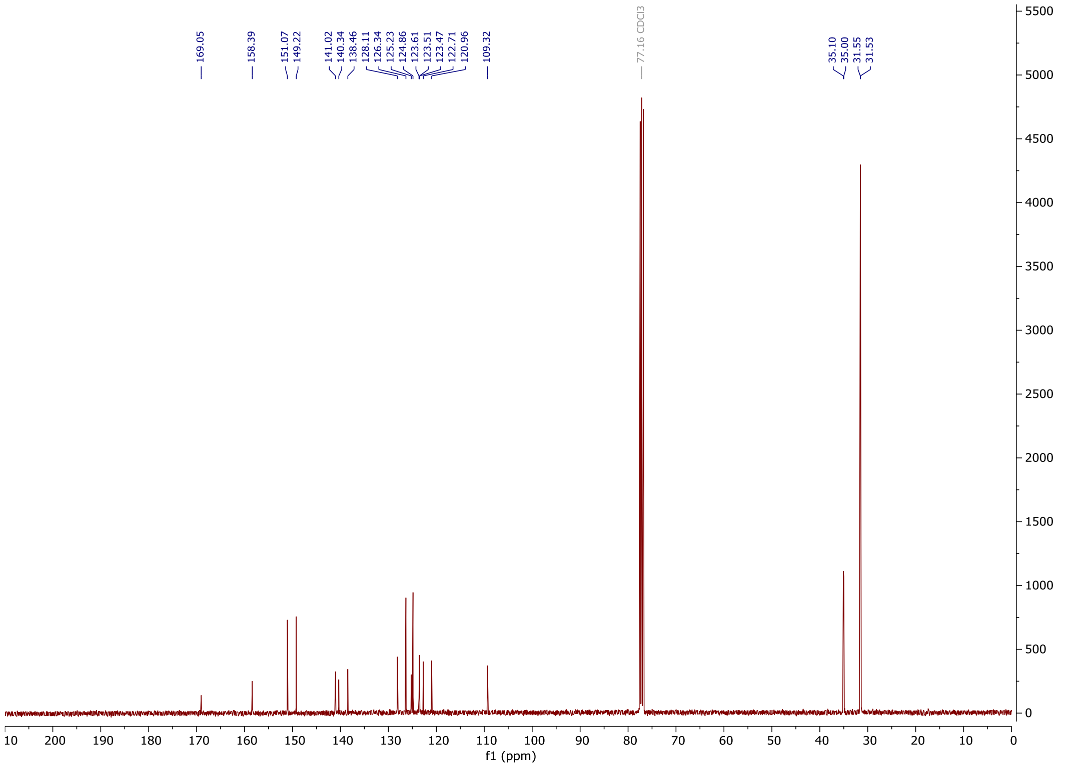

3-(Diphenylmethylene)indolin-2-one (7e)

^{1}H-NMR (500 MHz, DMSO-d_6, 300 K)

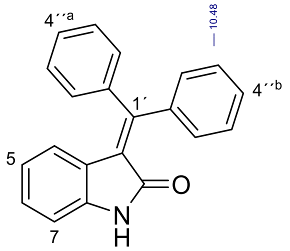

7e

$C_{21}H_{15}NO$

M = 297.36 g/mol

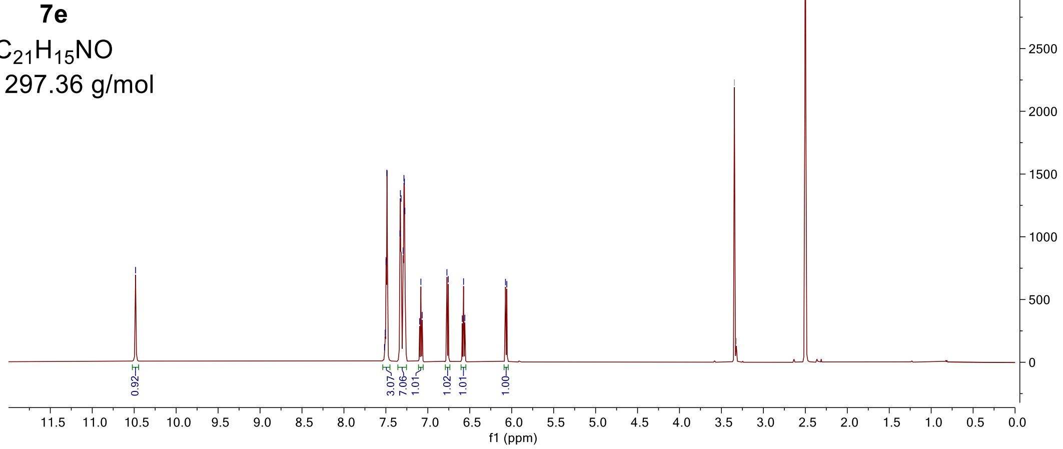

^{13}C-NMR (125 MHz, DMSO-d_6, 300 K)

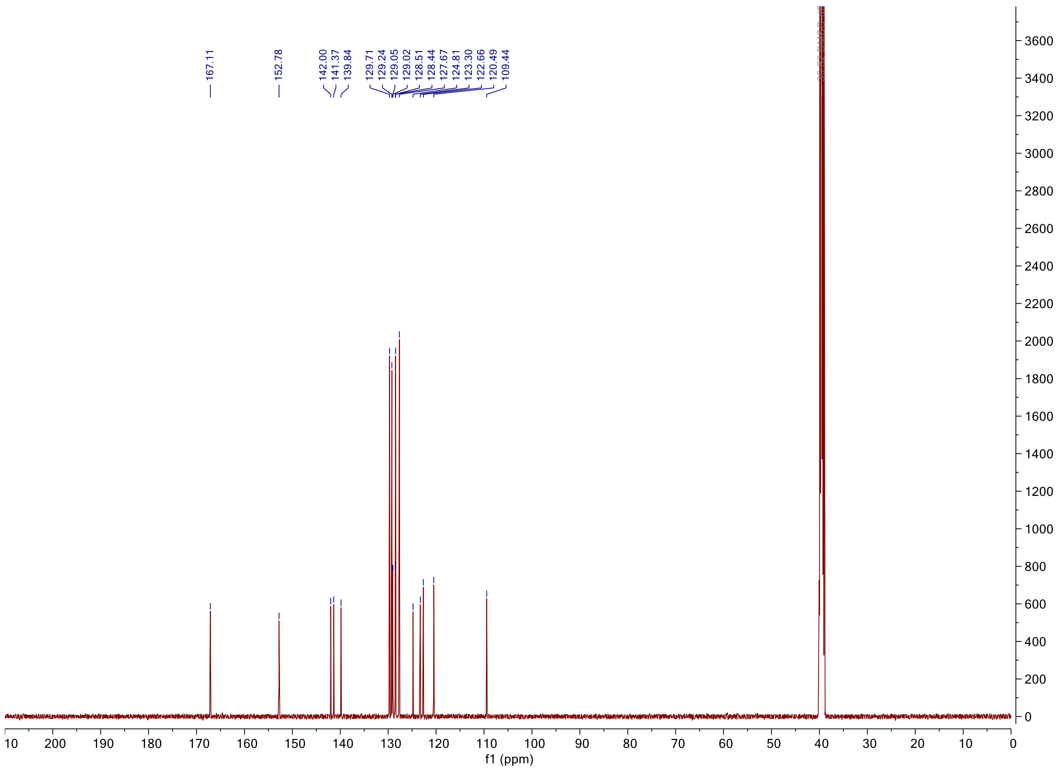

3-(Bis(4-fluorophenyl)methylene)indolin-2-one (7f)

^{1}H-NMR (500 MHz, DMSO-d_6, 300 K)

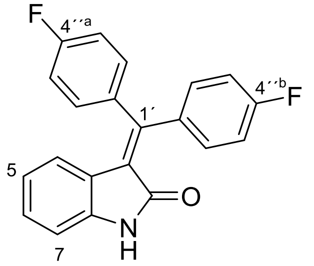

7f

$C_{21}H_{13}F_2NO$

M = 333.34 g/mol

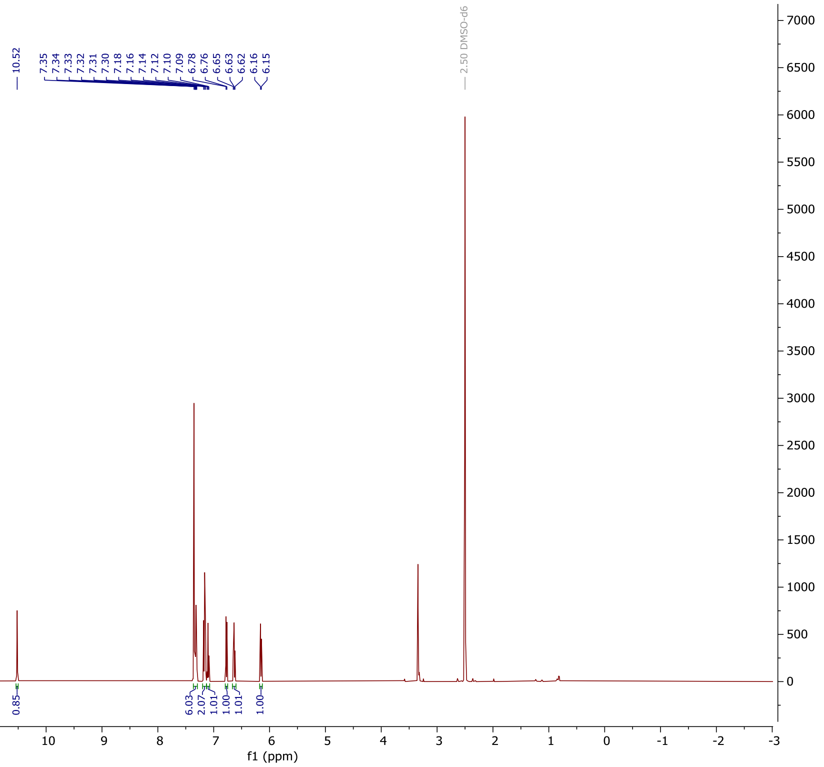

^{13}C-NMR (125 MHz, DMSO-d_6, 300 K)

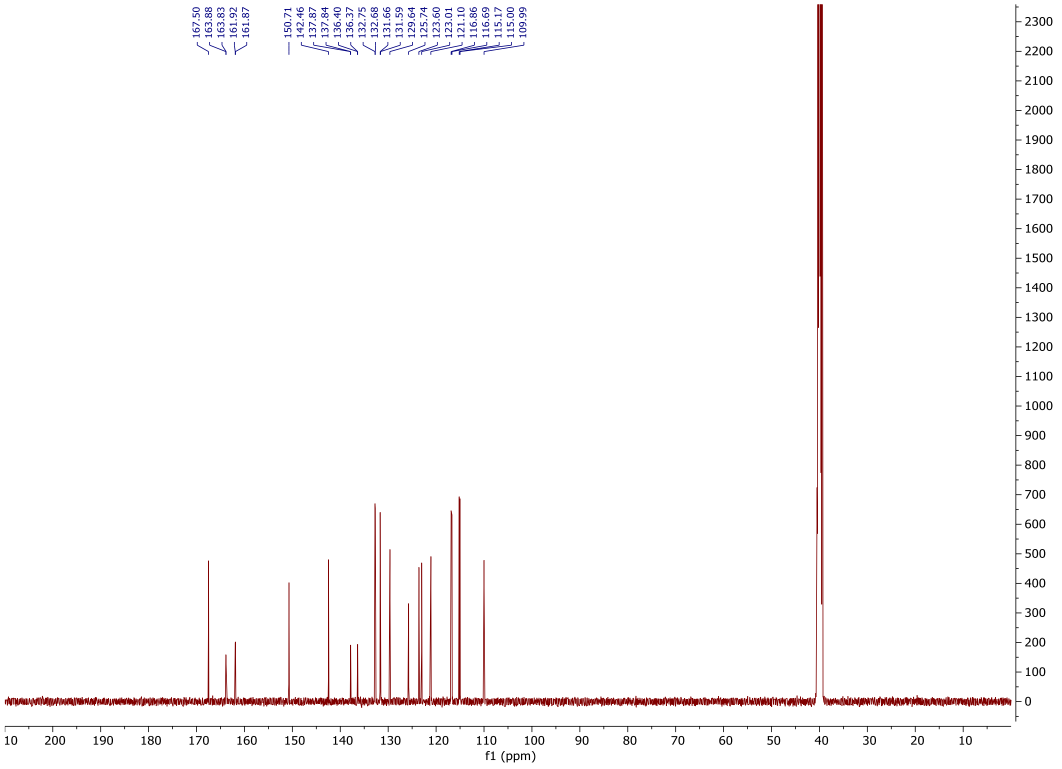

^{19}F-NMR (470 MHz, DMSO-d_6, 300 K)

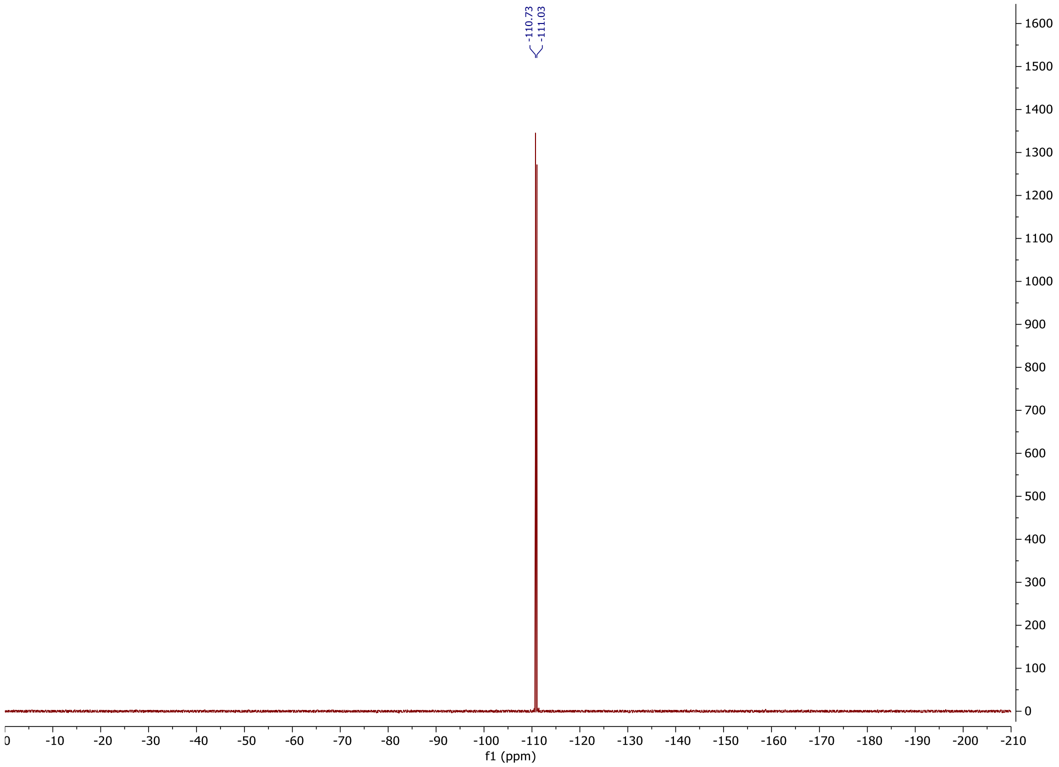

3-(Bis(4-bromophenyl)methylene)indolin-2-one (7g)

^{1}H-NMR (500 MHz, DMSO-d_6, 300 K)

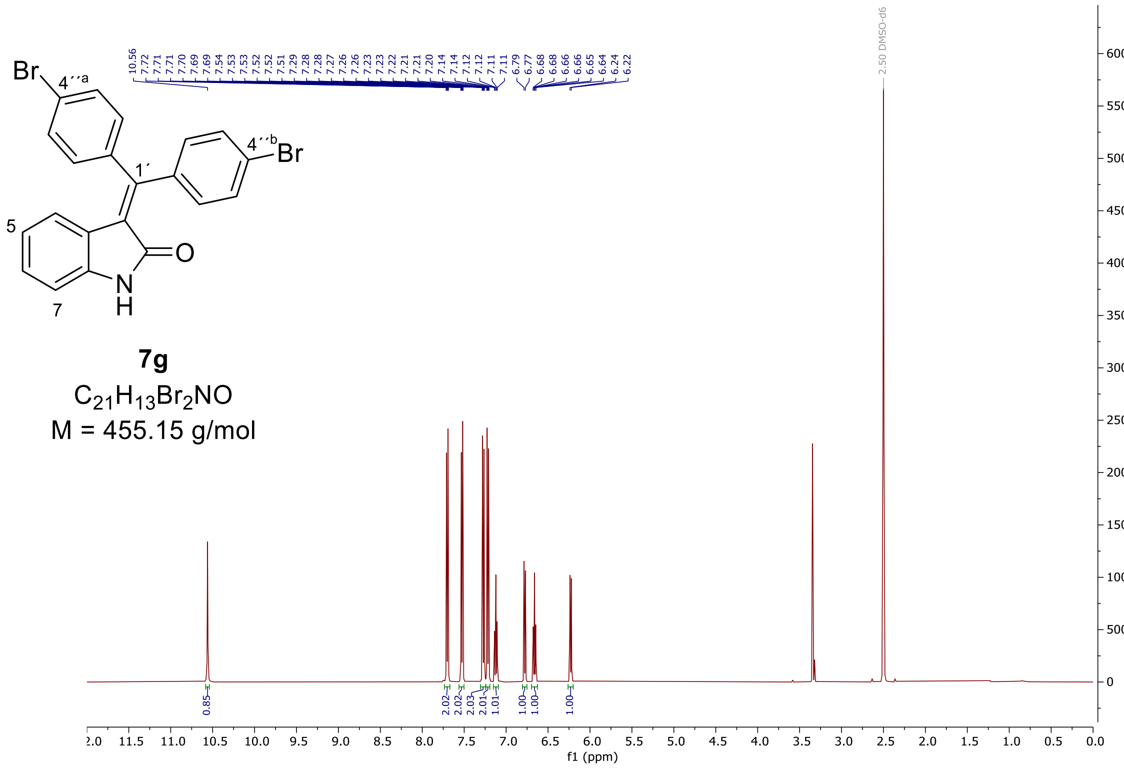

^{13}C-NMR (125 MHz, DMSO-d_6, 300 K)

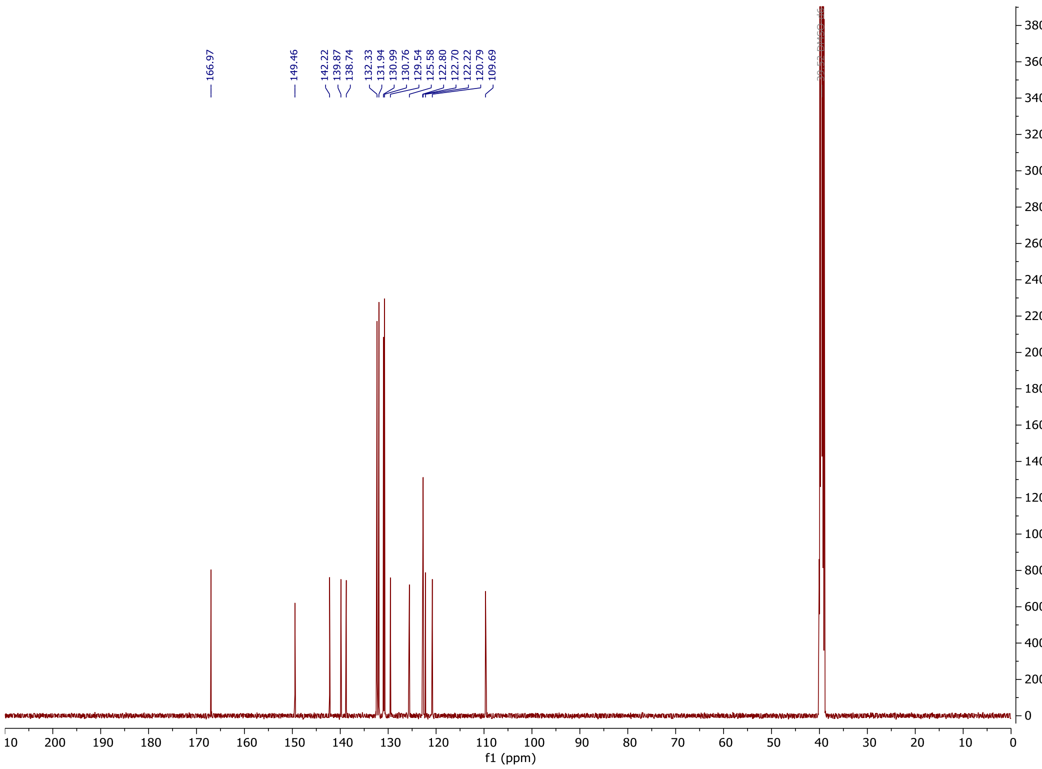

3-(Bis(3,5-bis(trifluoromethyl)phenyl)methylene)indolin-2-one (7h)

^{1}H-NMR (500 MHz, DMSO-d_6, 300 K)

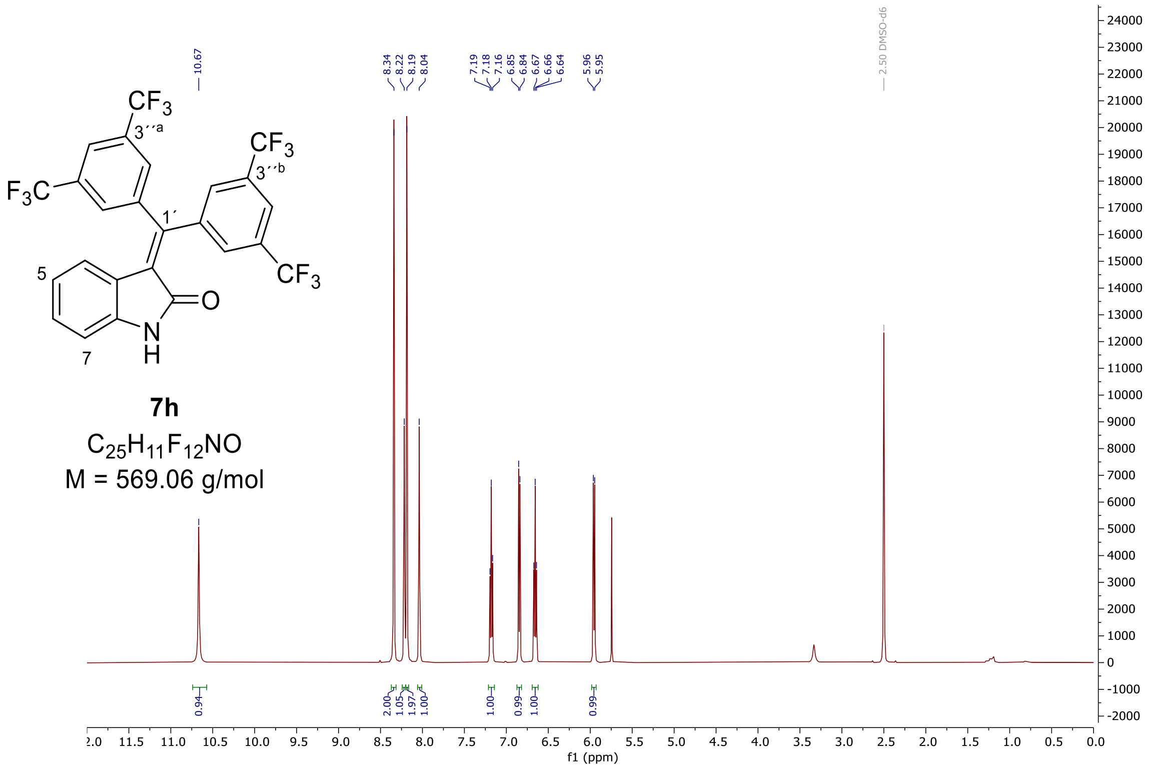

^{13}C-NMR (125 MHz, $CDCl_3$, 300 K)

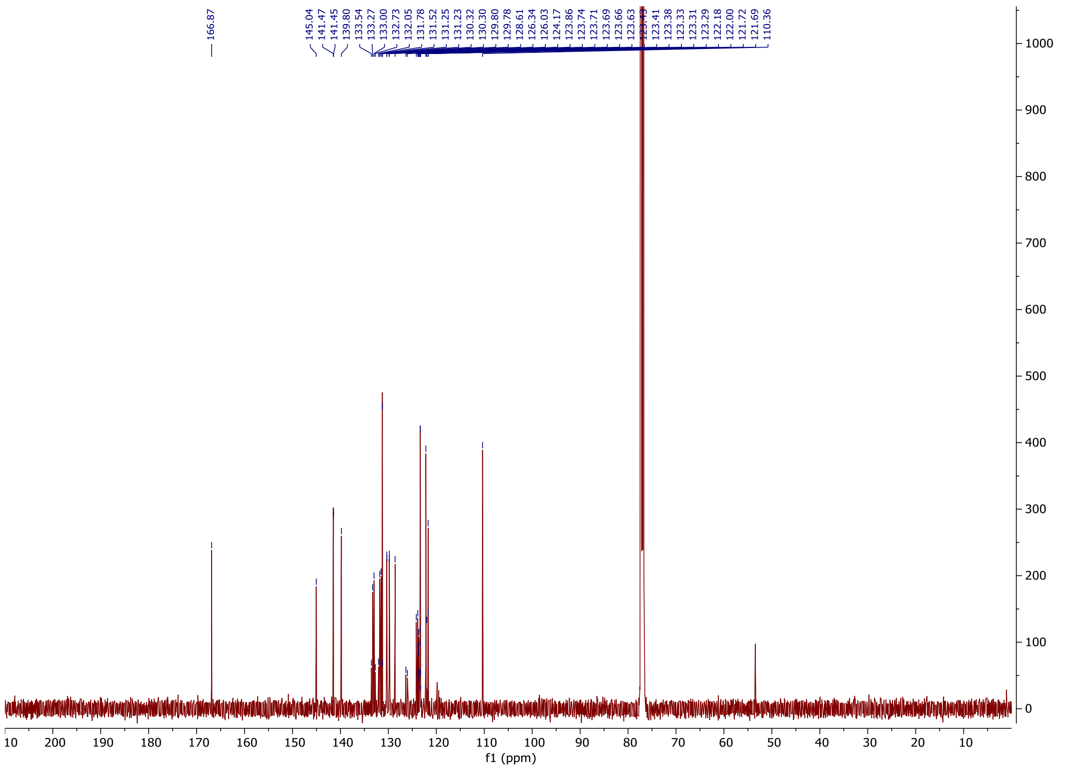

^{19}F-NMR (470 MHz, DMSO-d_6, 300 K)

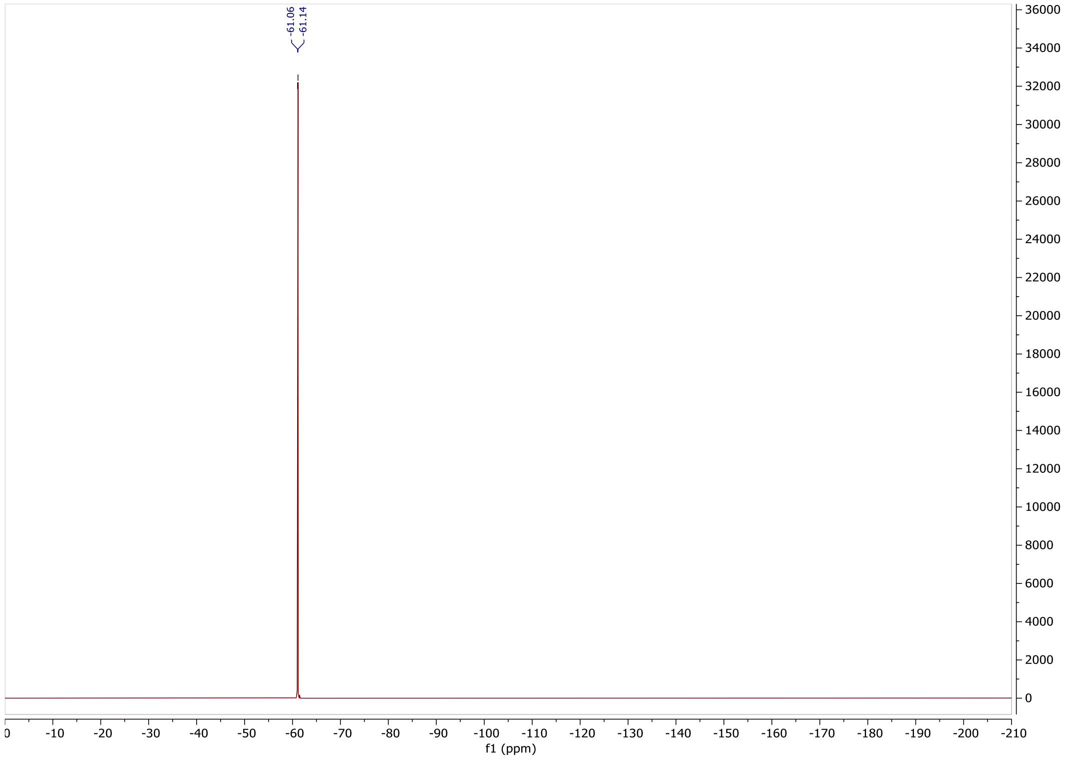

3-(Bis(3-nitrophenyl)methylene)indolin-2-one (7i)

^{1}H-NMR (500 MHz, DMSO-d_6, 300 K)

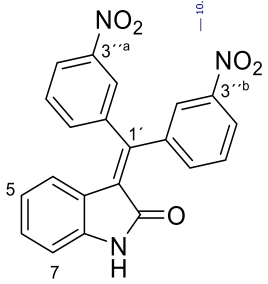

7i

$C_{21}H_{13}N_3O_5$

M = 387.09 g/mol

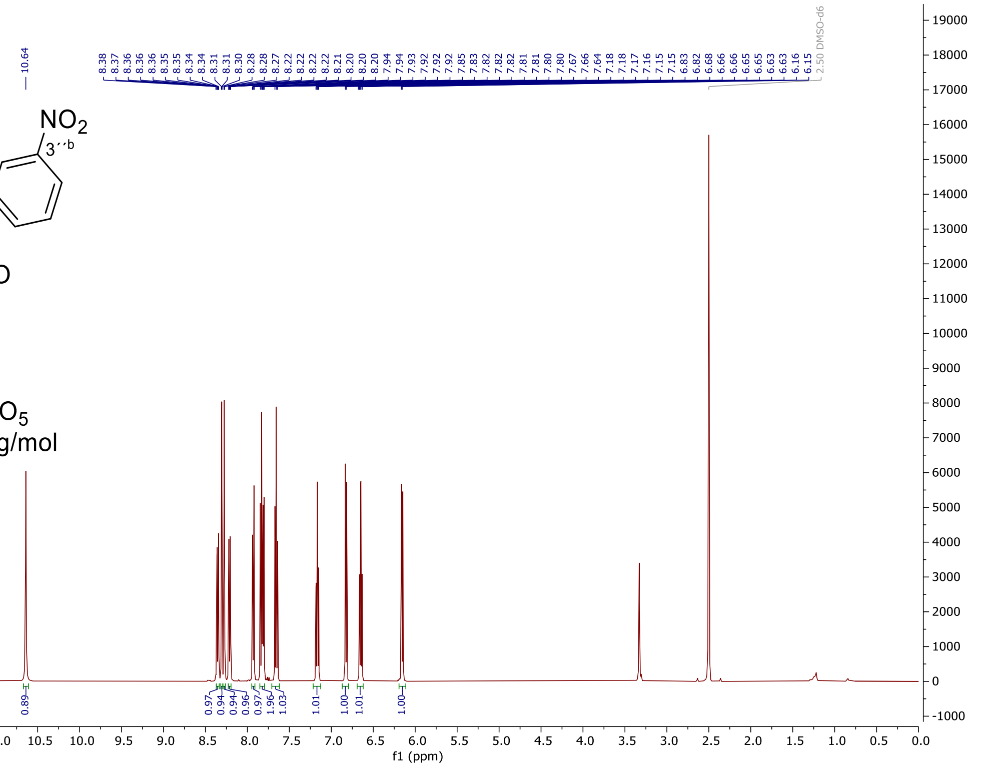

^{13}C-NMR (125 MHz, DMSO-d_6, 300 K)

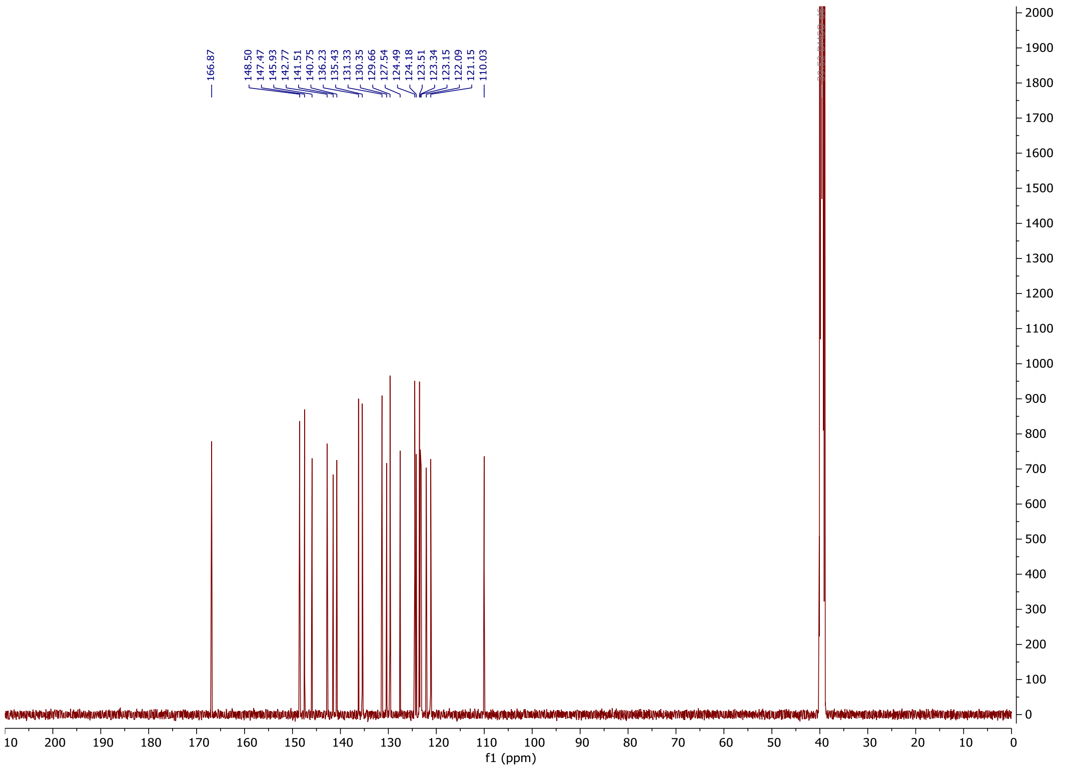

((2-Oxoindolin-3-ylidene)methylene)bis(4,1-phenylene) bis(4-methylbenzenesulfonate) 7j

^{1}H-NMR (500 MHz, DMSO-d_6, 300 K)

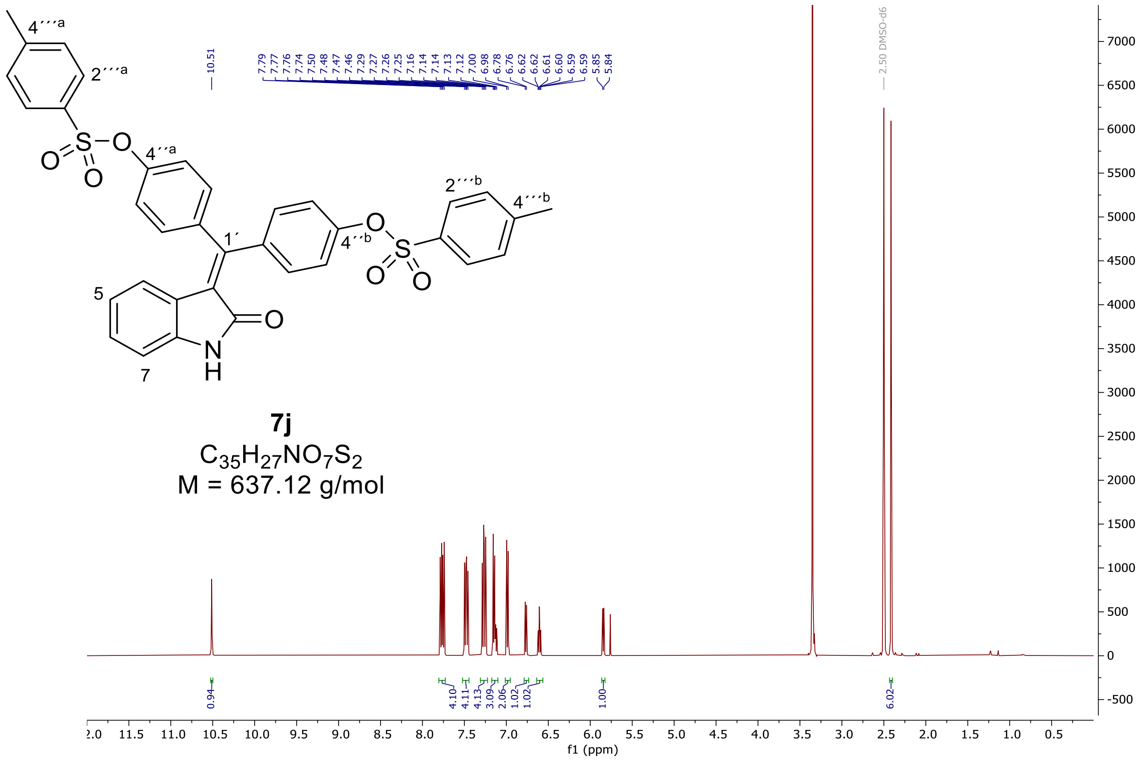

^{13}C-NMR (125 MHz, DMSO-d_6, 300 K)

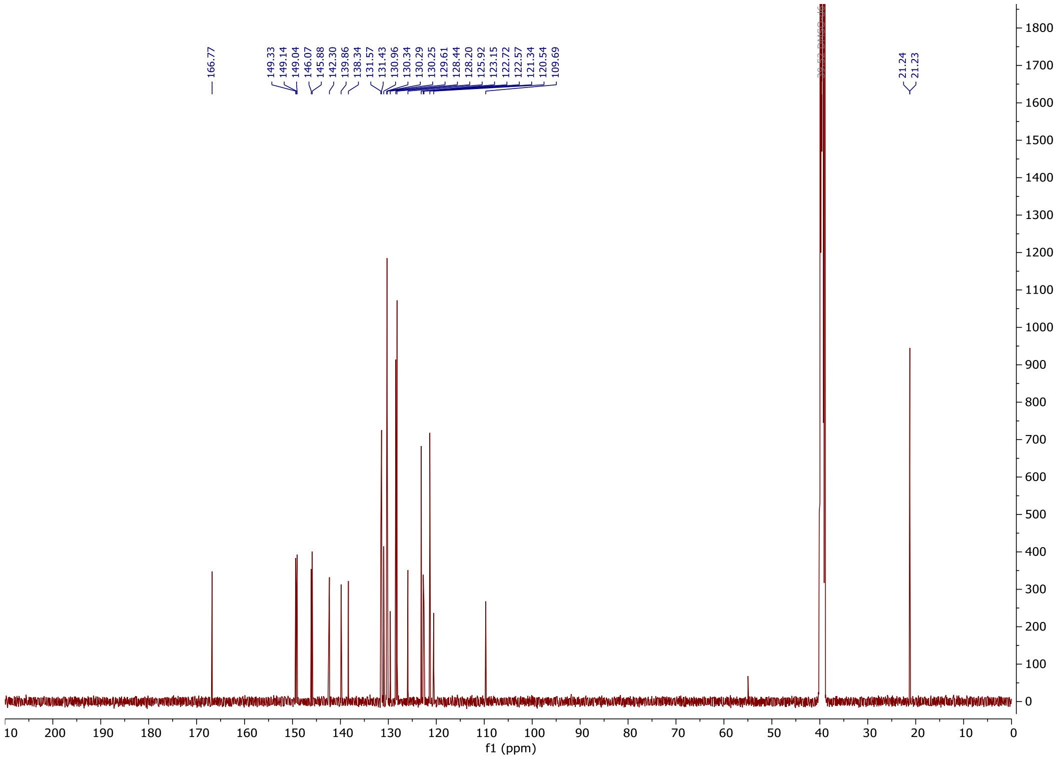

((2-Oxoindolin-3-ylidene)methylene)bis(4,1-phenylene) bis(2,2-dimethylpropanoate) (7k)

^{1}H-NMR (500 MHz, DMSO-d_6, 300 K)

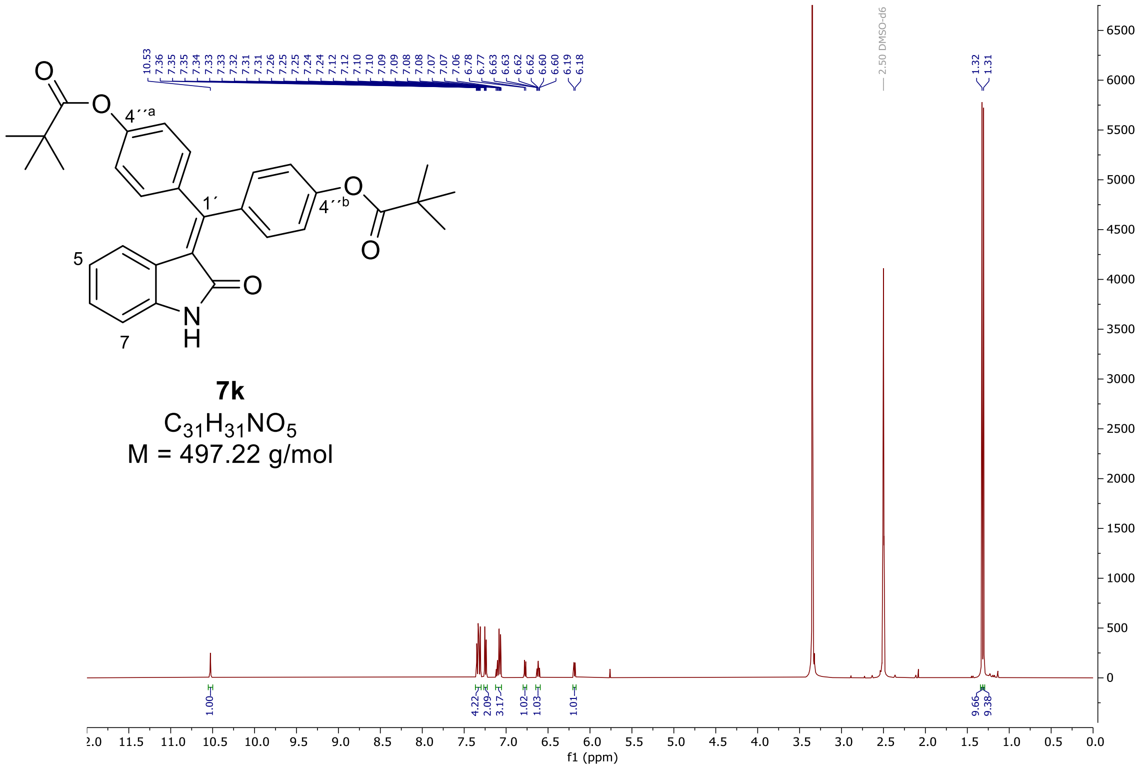

7k
$C_{31}H_{31}NO_5$
M = 497.22 g/mol

^{13}C-NMR (125 MHz, DMSO-d_6, 300 K)

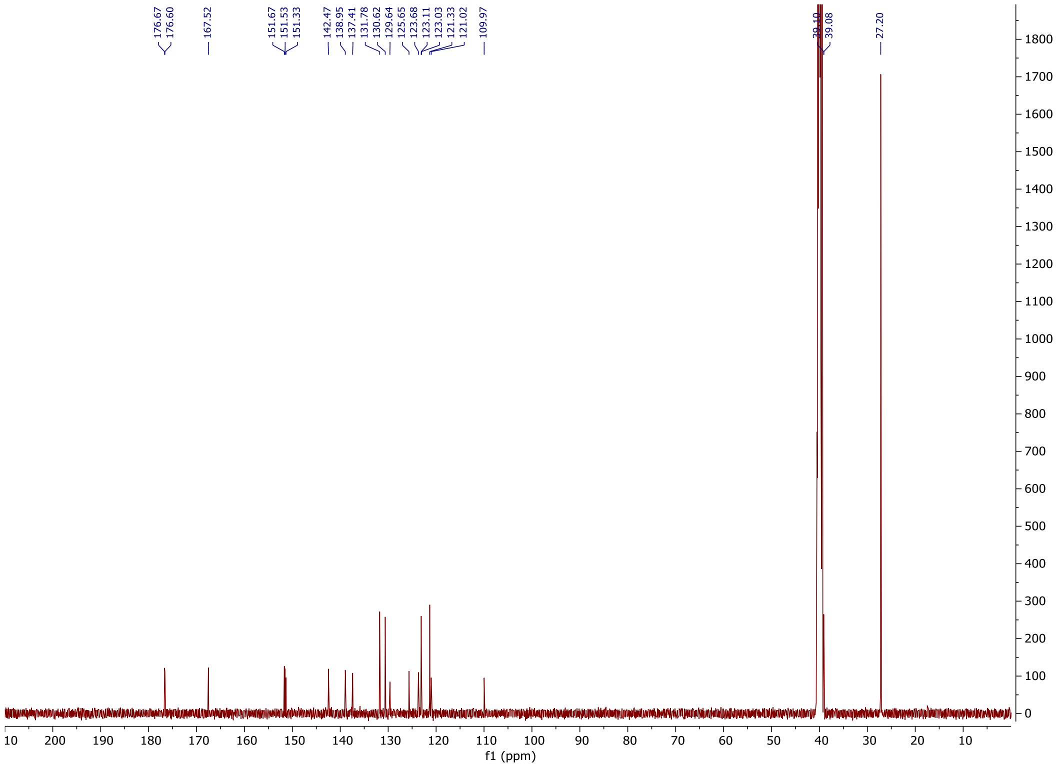

3-(Bis(4-fluorophenyl)methylene)-5-fluoroindolin-2-one (7l)

^{1}H-NMR (500 MHz, $CDCl_3$, 300 K)

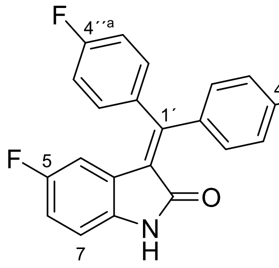

7l

$C_{21}H_{12}F_3NO$

M = 351.33 g/mol

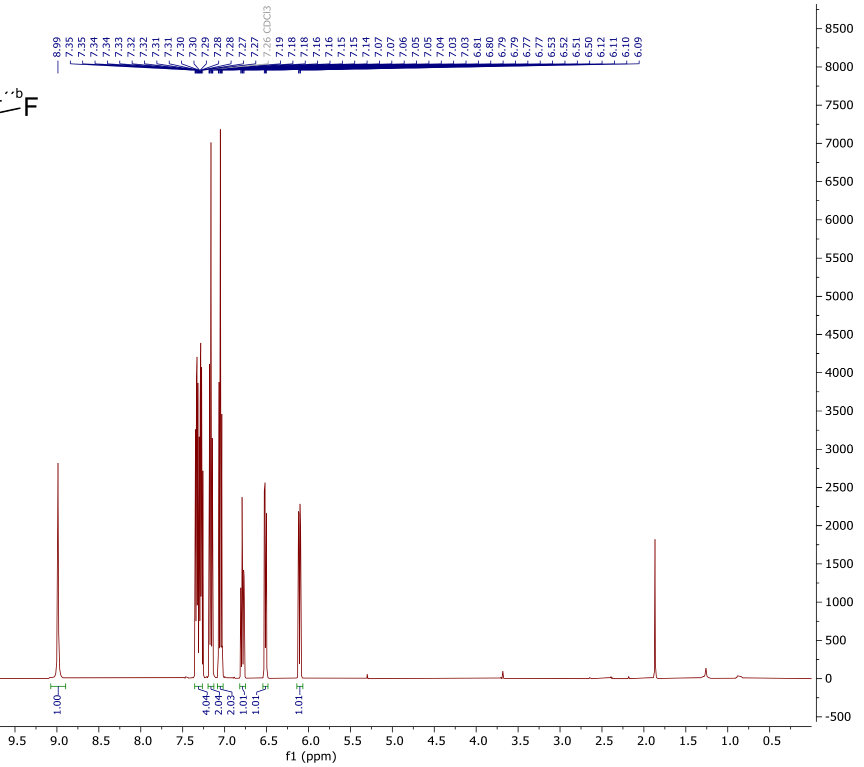

^{13}C-NMR (125 MHz, $CDCl_3$, 300 K)

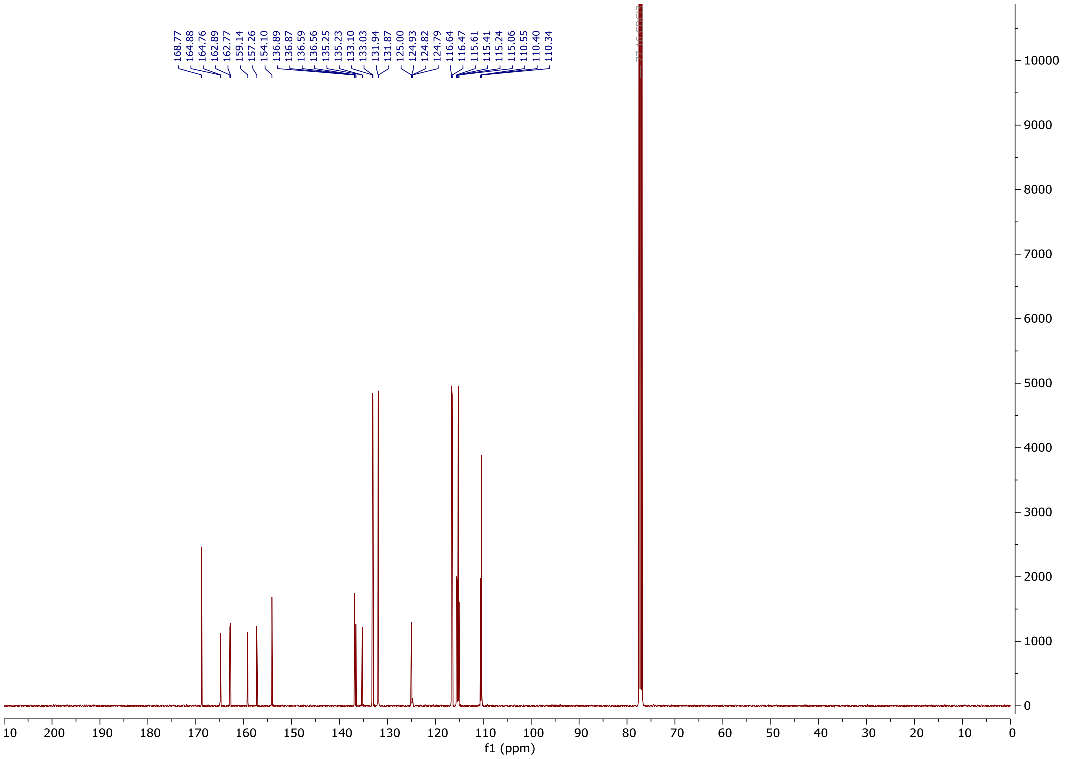

^{19}F-NMR (470 MHz, $CDCl_3$, 300 K)

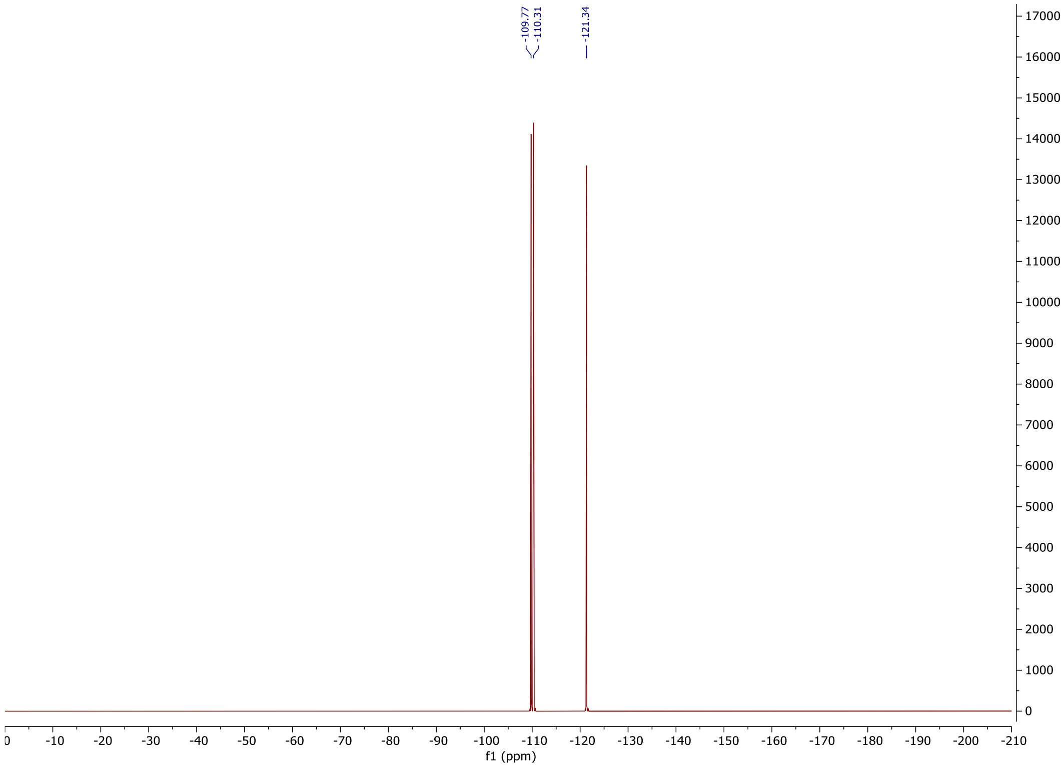

3-(Di-*p*-tolylmethylene)-5-fluoroindolin-2-one (7m)

^{1}H-NMR (500 MHz, $CDCl_3$, 300 K)

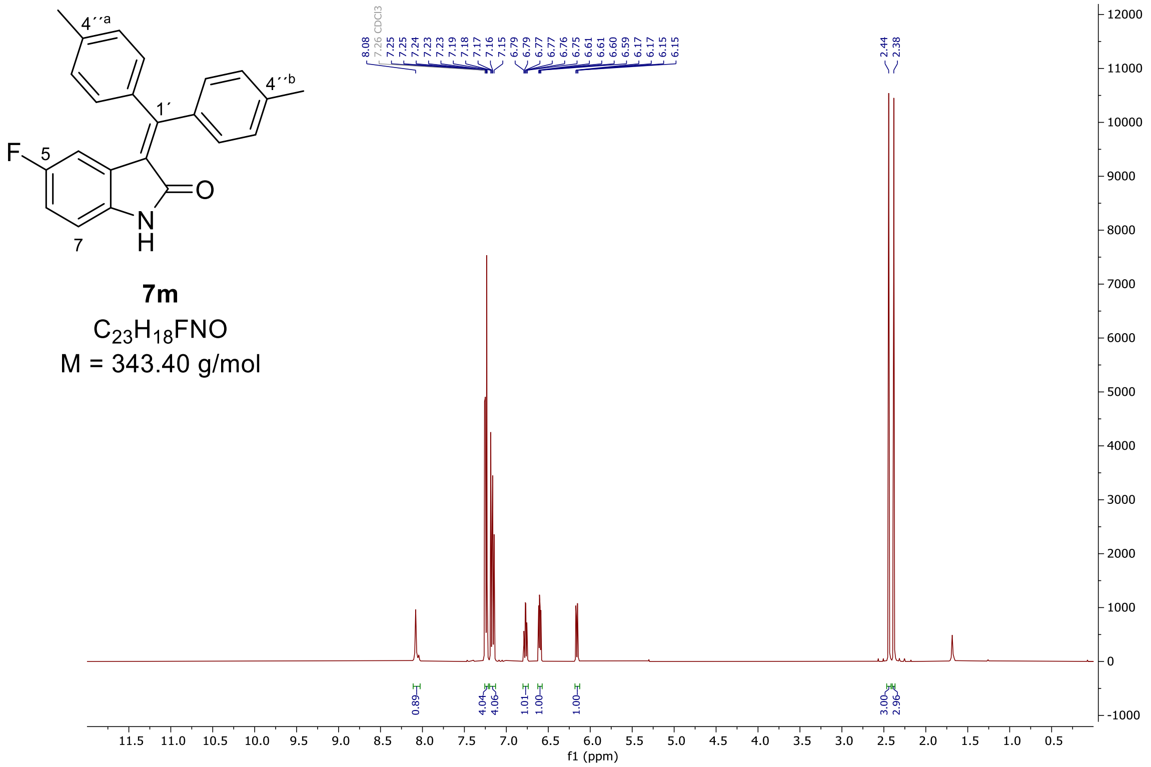

^{13}C-NMR (125 MHz, $CDCl_3$, 300 K)

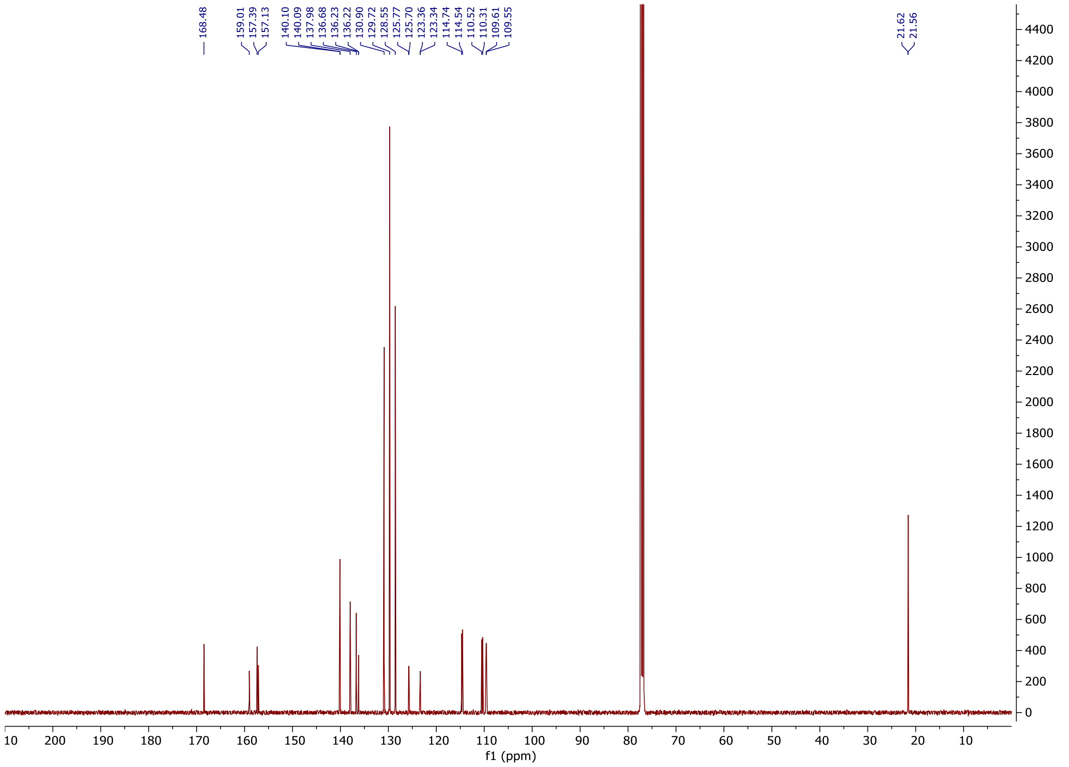

^{19}F-NMR (470 MHz, DMSO-d_6, 300 K)

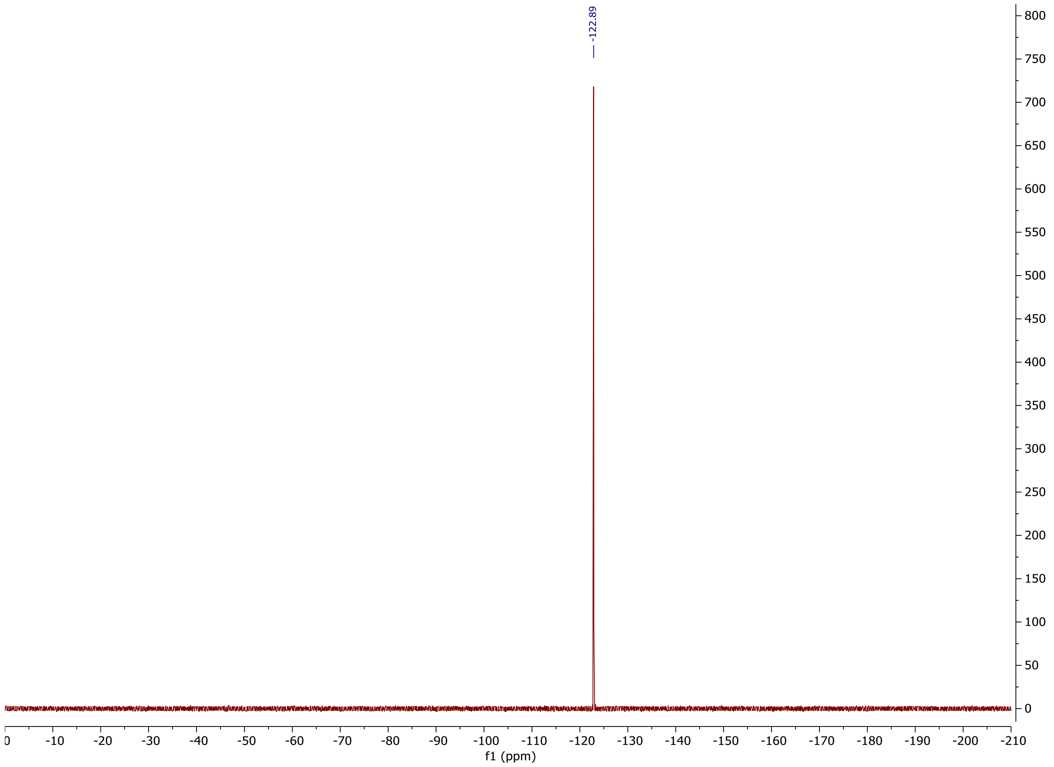

3-(Bis(4-chlorophenyl)methylene)-5-fluoroindolin-2-one (7n)

^{1}H-NMR (500 MHz, DMSO-d_6, 300 K)

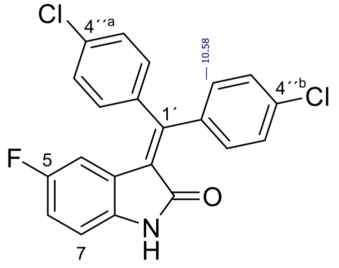

7n

$C_{21}H_{12}Cl_2FNO$

M = 383.03 g/mol

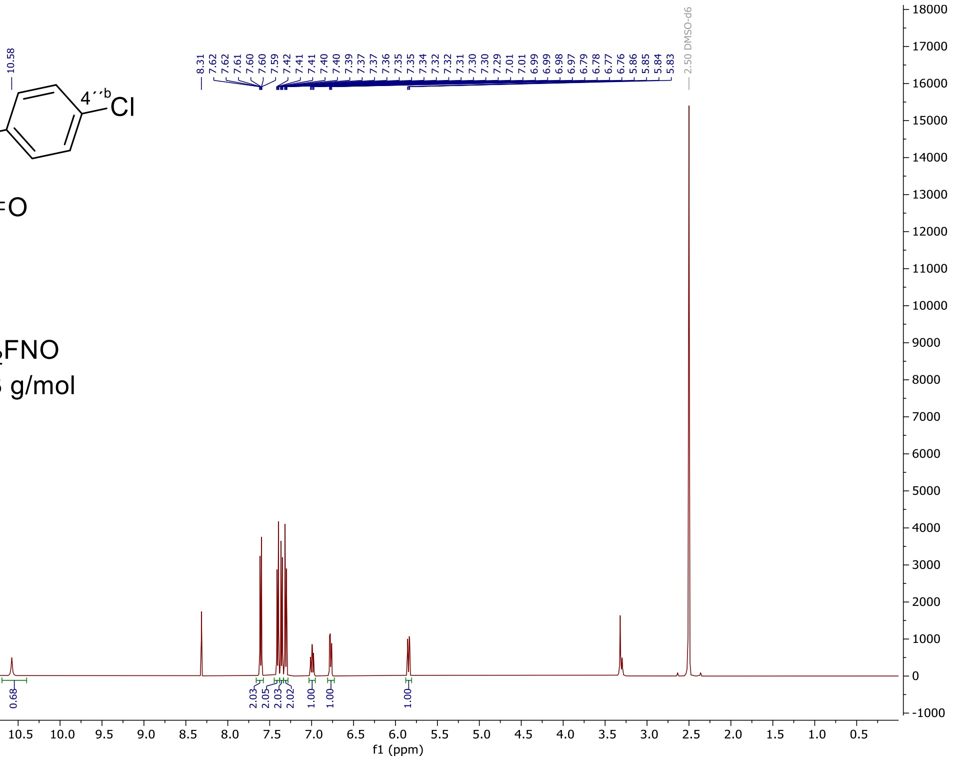

^{13}C-NMR (125 MHz, $CDCl_3$, 300 K)

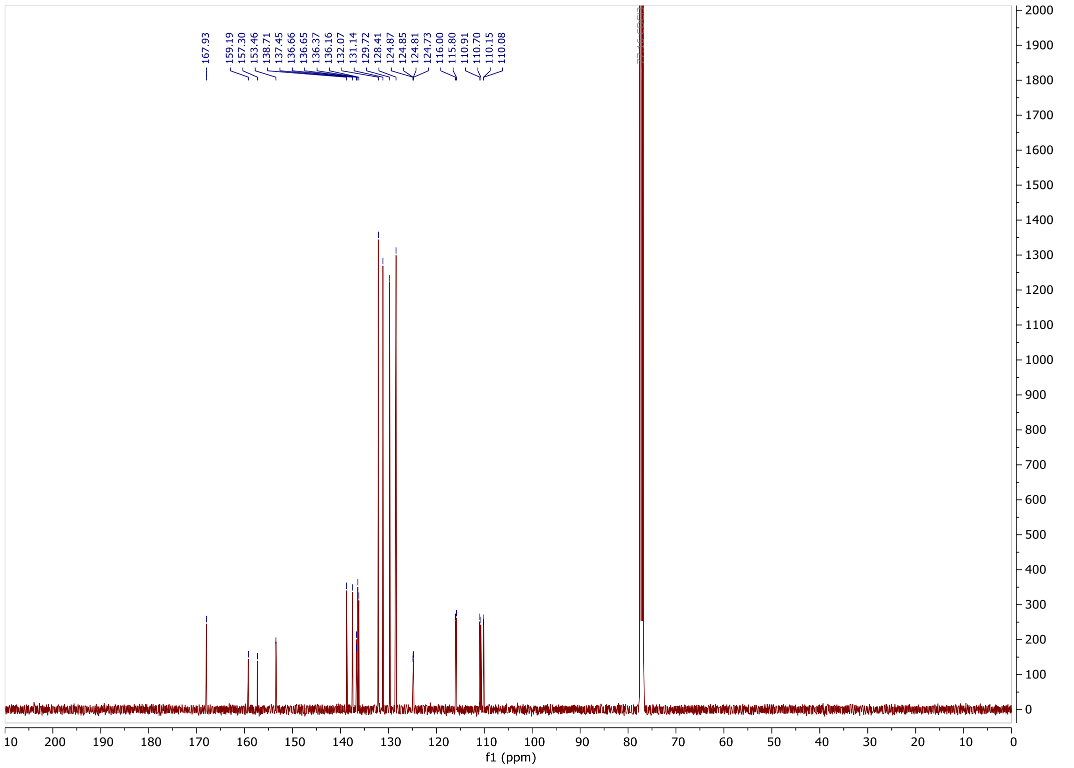

^{19}F-NMR (470 MHz, DMSO-d_6, 300 K)

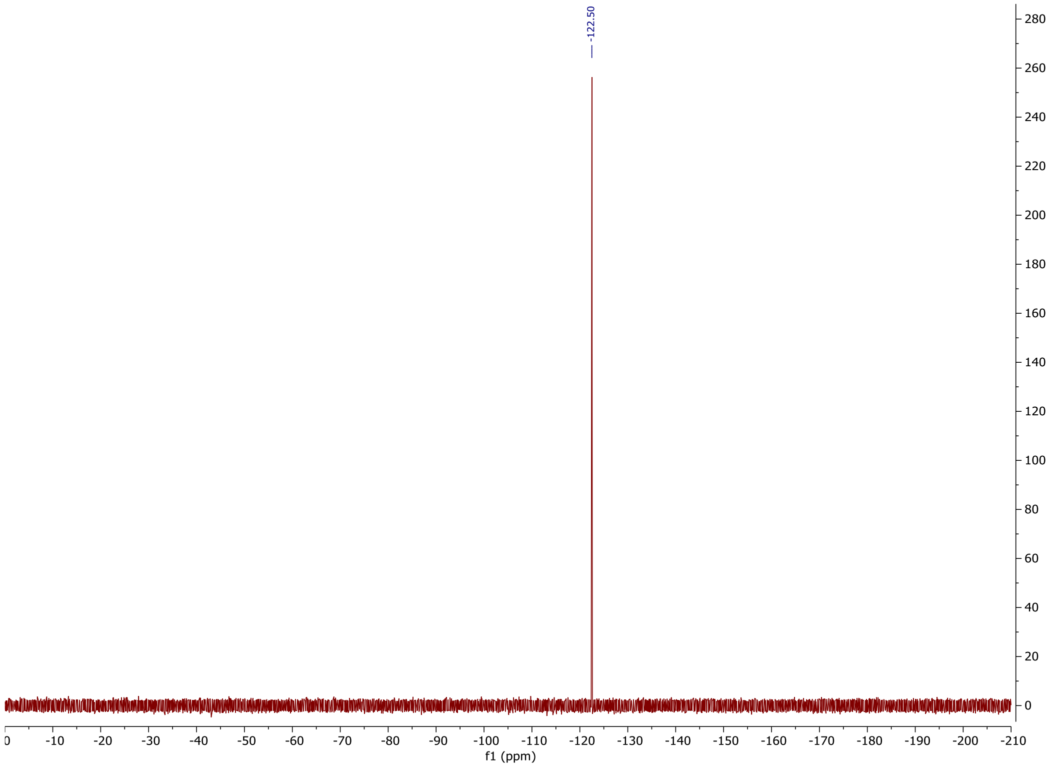

3-(Bis(4-chlorophenyl)methylene)-5-chloroindolin-2-one (7o)

^{1}H-NMR (500 MHz, DMSO-d_6, 300 K)

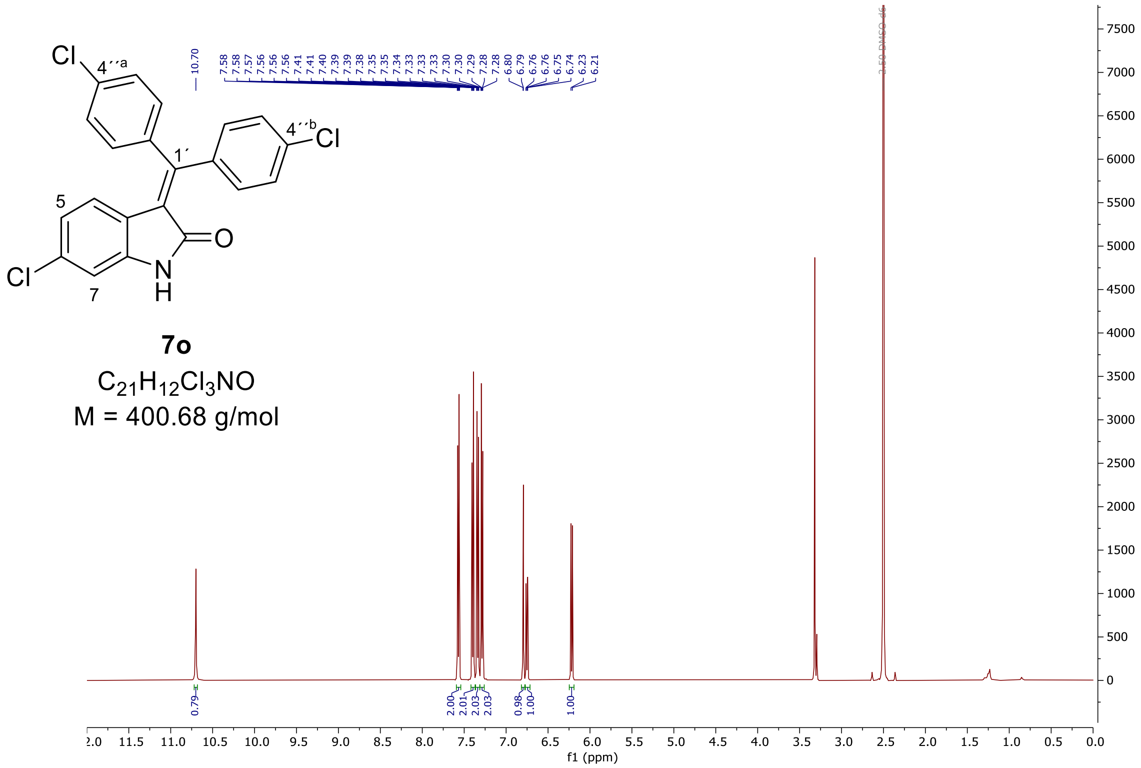

^{13}C-NMR (125 MHz, $CDCl_3$, 300 K)

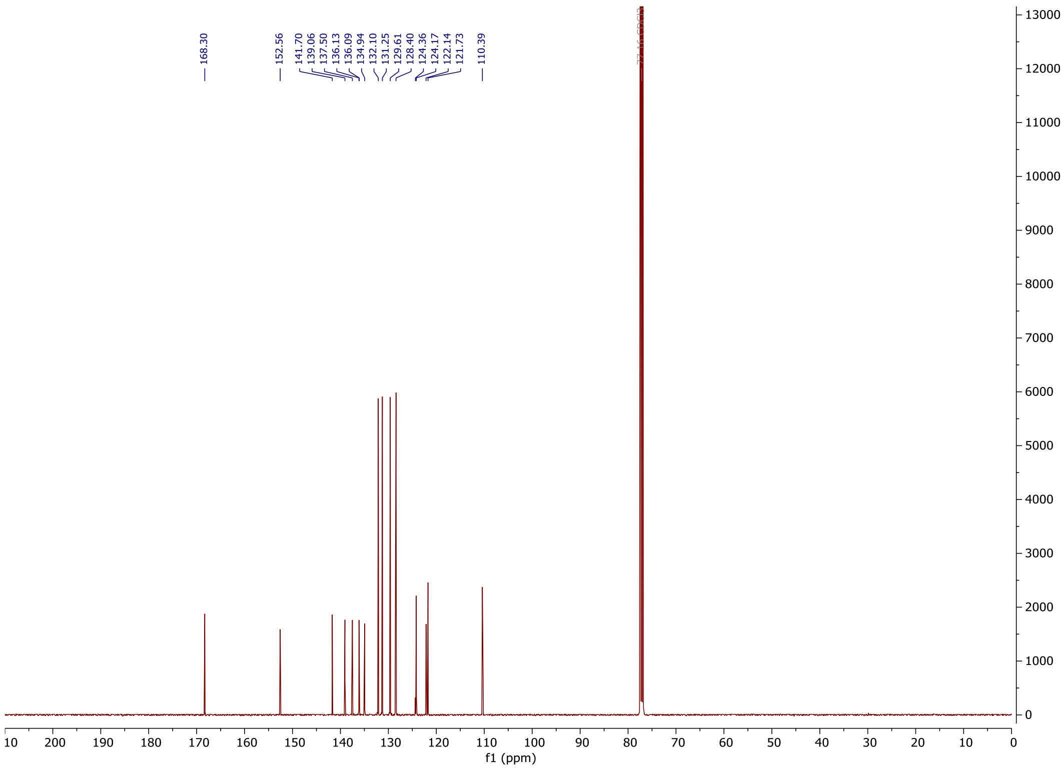

16. HPLC-Traces

(*R*)-3',3'-Bis(4-chlorophenyl)spiro[indoline-3,2'-oxetan]-2-one (5a)

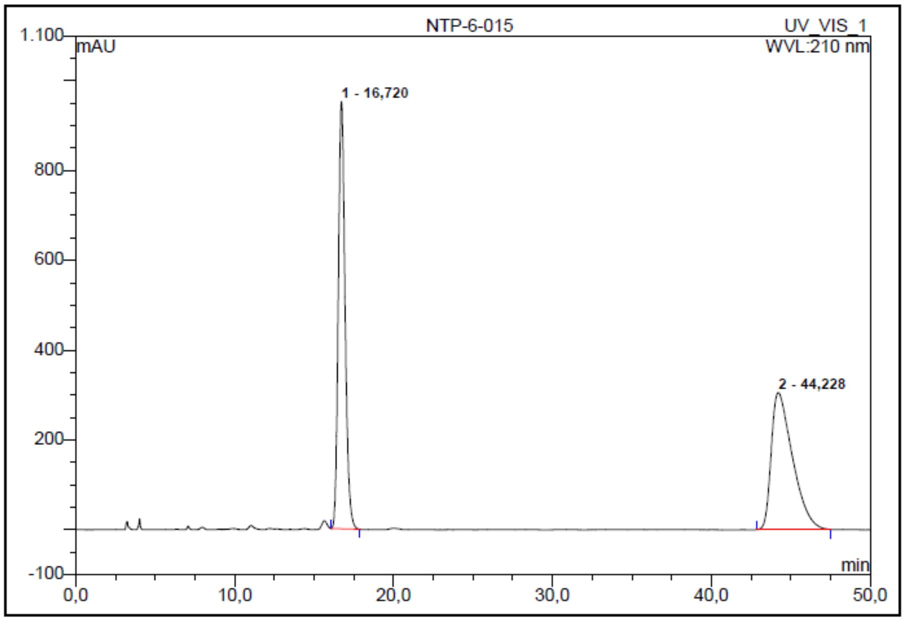

No.	Ret.Time min	Peak Name	Height mAU	Area mAU*min	Rel.Area %	Amount	Type
1	16,72	n.a.	951,762	471,519	49,95	n.a.	BMB*
2	44,23	n.a.	305,102	472,370	50,05	n.a.	BMB*
Total:			1256,864	943,890	100,00	0,000	

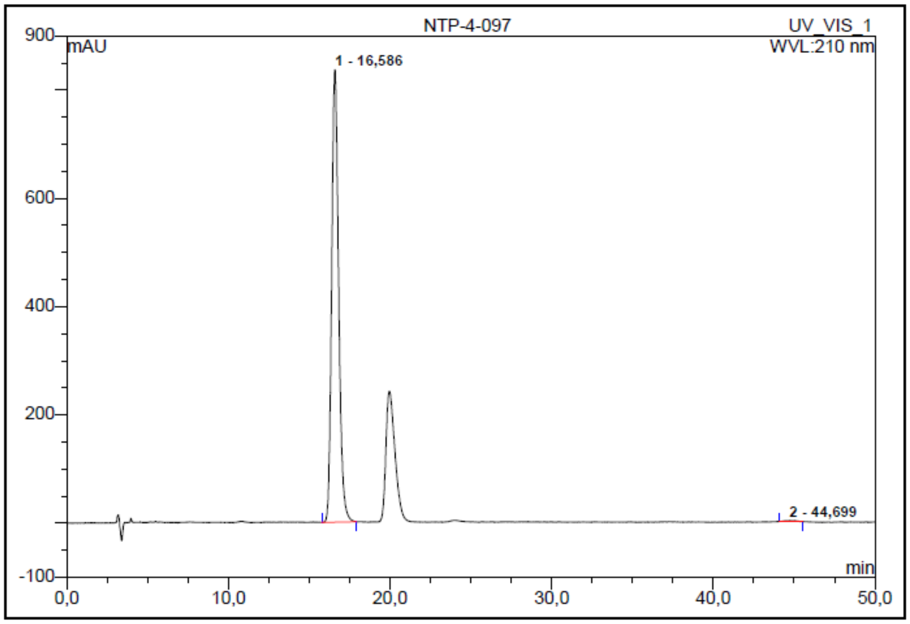

No.	Ret.Time min	Peak Name	Height mAU	Area mAU*min	Rel.Area %	Amount	Type
1	16,59	n.a.	835,860	417,766	99,62	n.a.	BMB*
2	44,70	n.a.	2,212	1,601	0,38	n.a.	BMB*
Total:			838,071	419,367	100,00	0,000	

(*R*)-3',3'-Di-*p*-tolylspiro[indoline-3,2'-oxetan]-2-one (5b)

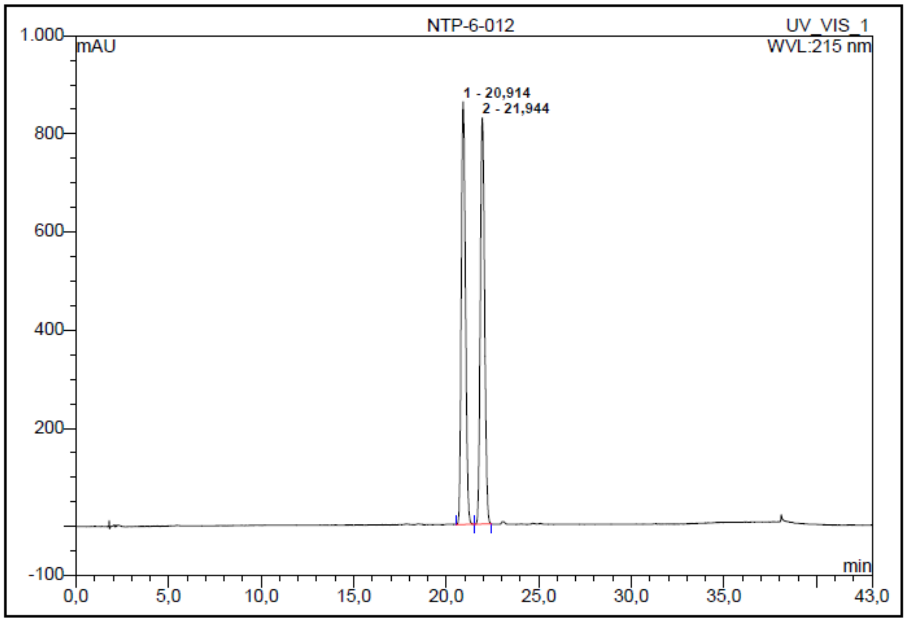

No.	Ret.Time min	Peak Name	Height mAU	Area mAU*min	Rel.Area %	Amount	Type
1	20,91	n.a.	861,200	225,485	50,05	n.a.	BM
2	21,94	n.a.	827,946	225,051	49,95	n.a.	MB
Total:			1689,145	450,536	100,00	0,000	

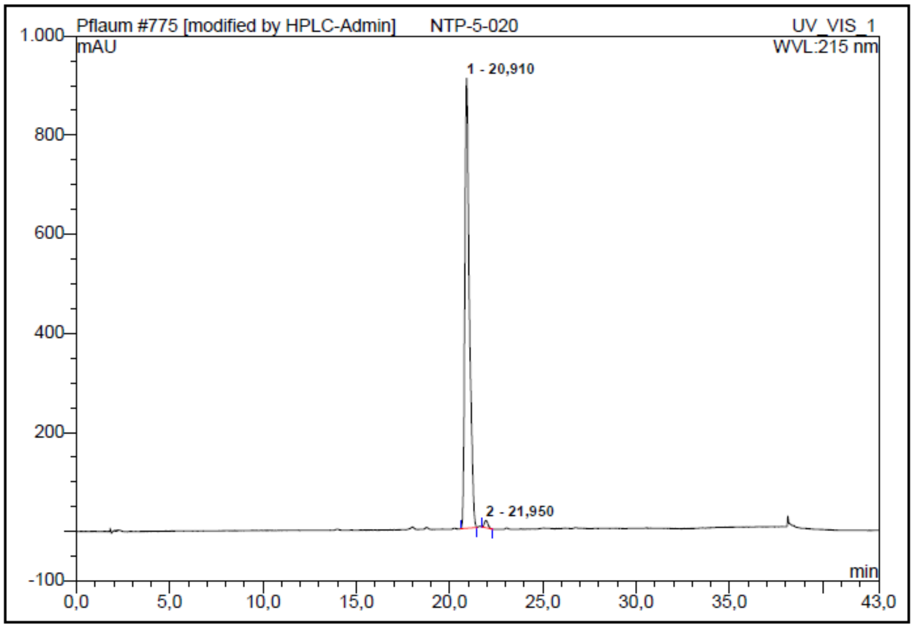

No.	Ret.Time min	Peak Name	Height mAU	Area mAU*min	Rel.Area %	Amount	Type
1	20,91	n.a.	908,414	256,190	98,75	n.a.	BMB*
2	21,95	n.a.	14,839	3,250	1,25	n.a.	BMB*
Total:			923,252	259,440	100,00	0,000	

(*R*)-3',3'-Bis(4-(*tert*-butyl)phenyl)spiro[indoline-3,2'-oxetan]-2-one (5c)

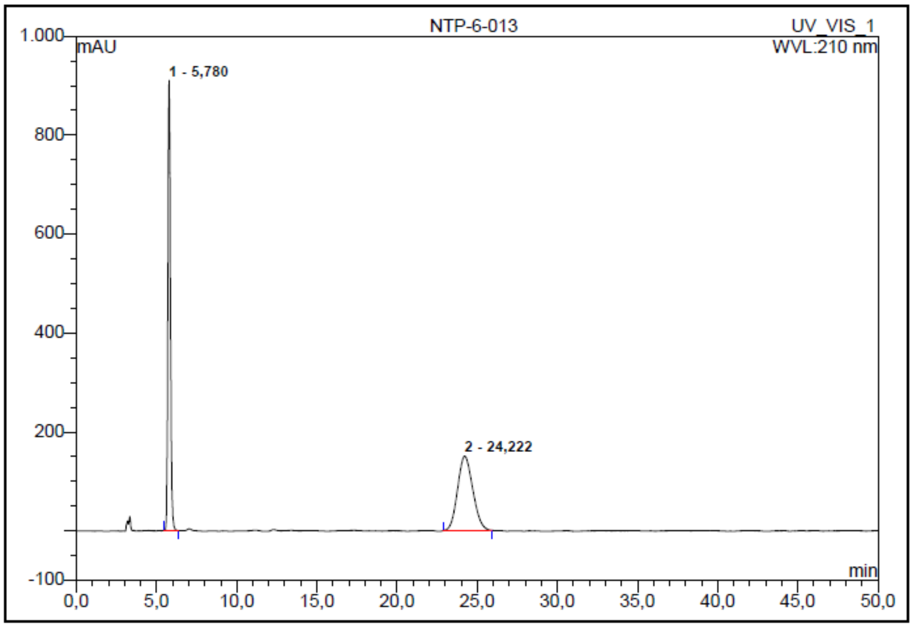

No.	Ret.Time min	Peak Name	Height mAU	Area mAU*min	Rel.Area %	Amount	Type
1	5,78	n.a.	910,257	167,780	50,25	n.a.	BMB
2	24,22	n.a.	150,618	166,094	49,75	n.a.	BMB
Total:			1060,875	333,874	100,00	0,000	

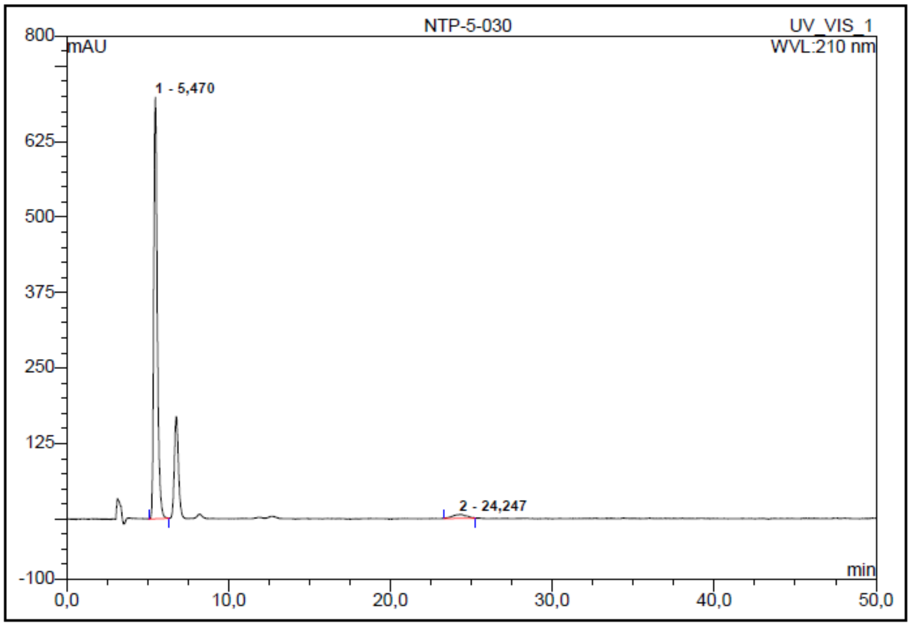

No.	Ret.Time min	Peak Name	Height mAU	Area mAU*min	Rel.Area %	Amount	Type
1	5,47	n.a.	698,138	176,746	96,55	n.a.	BMB*
2	24,25	n.a.	6,325	6,323	3,45	n.a.	BMB*
Total:			704,463	183,069	100,00	0,000	

(*R*)-3',3'-Bis(3,5-di-*tert*-butylphenyl)spiro[indoline-3,2'-oxetan]-2-one (5d)

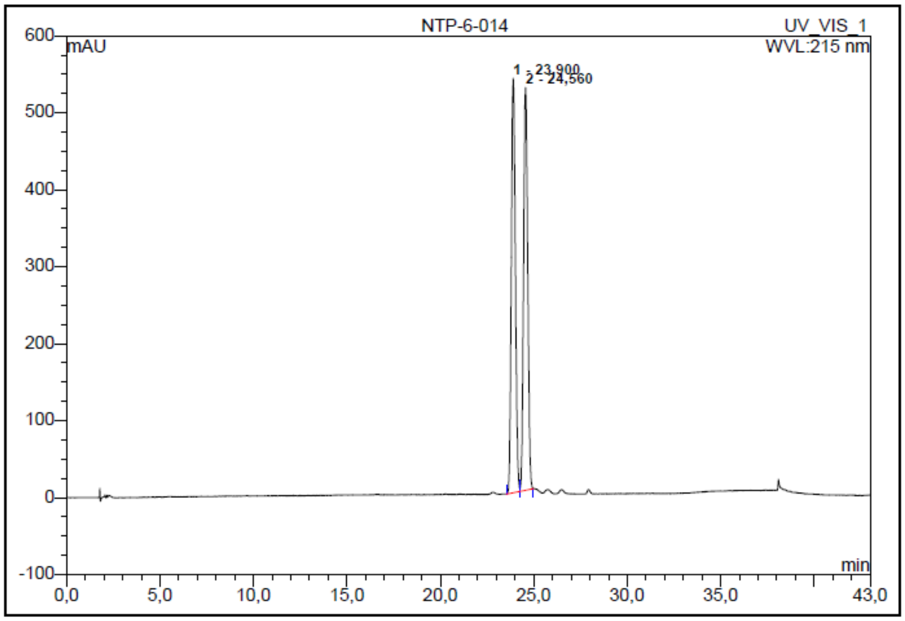

No.	Ret.Time min	Peak Name	Height mAU	Area mAU*min	Rel.Area %	Amount	Type
1	23,90	n.a.	538,404	135,225	50,16	n.a.	BM
2	24,56	n.a.	523,442	134,381	49,84	n.a.	MB
Total:			1061,846	269,607	100,00	0,000	

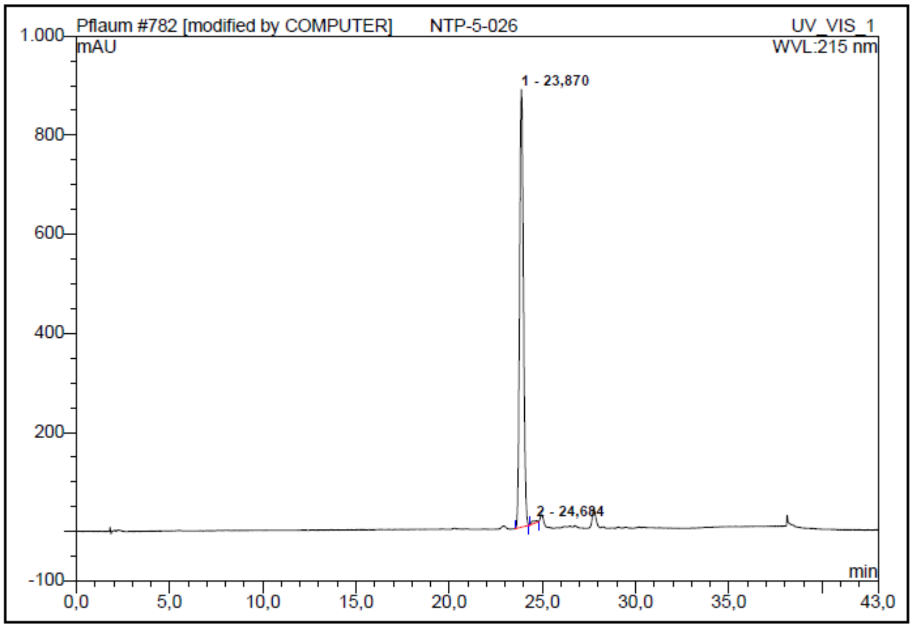

No.	Ret.Time min	Peak Name	Height mAU	Area mAU*min	Rel.Area %	Amount	Type
1	23,87	n.a.	883,577	213,655	99,22	n.a.	BMB
2	24,68	n.a.	3,698	1,674	0,78	n.a.	BMB*
Total:			887,275	215,330	100,00	0,000	

(*R*)-3',3'-Diphenylspiro[indoline-3,2'-oxetan]-2-one (5e)

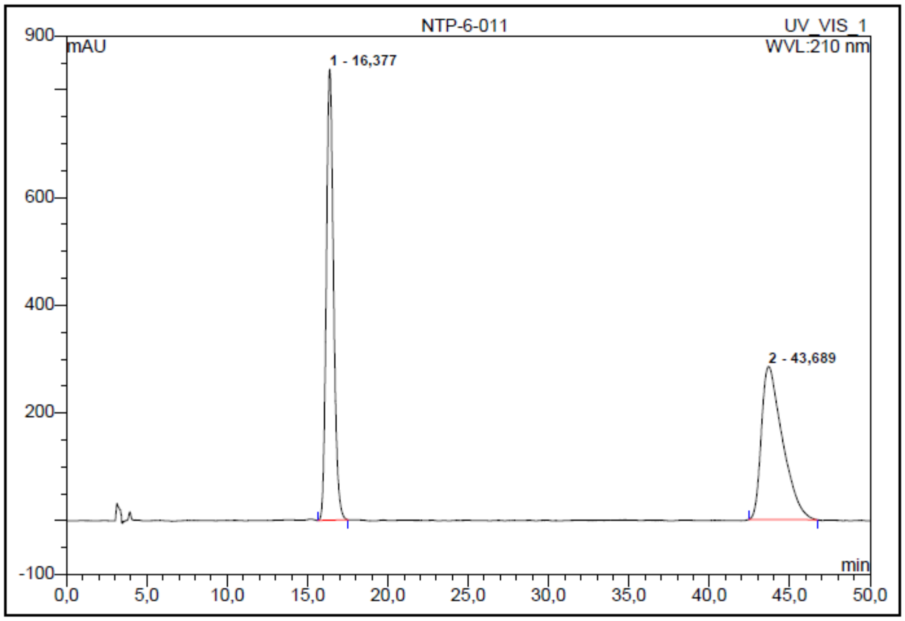

No.	Ret.Time min	Peak Name	Height mAU	Area mAU*min	Rel.Area %	Amount	Type
1	16,38	n.a.	837,081	438,058	50,34	n.a.	BMB
2	43,69	n.a.	283,888	432,205	49,66	n.a.	BMB*
Total:			1120,969	870,263	100,00	0,000	

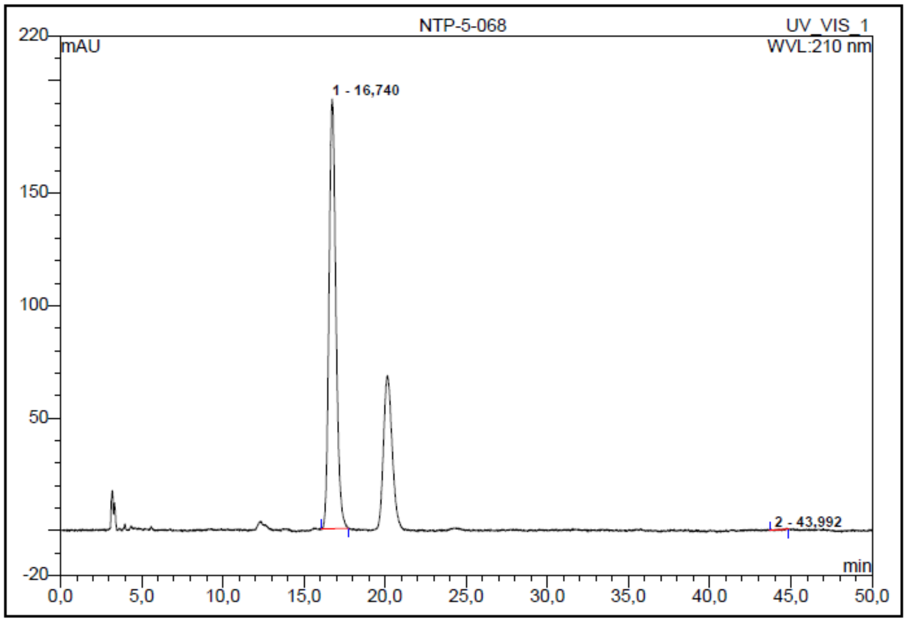

No.	Ret.Time min	Peak Name	Height mAU	Area mAU*min	Rel.Area %	Amount	Type
1	16,74	n.a.	190,997	94,524	99,93	n.a.	BMB*
2	43,99	n.a.	0,415	0,071	0,07	n.a.	BMB*
Total:			191,412	94,595	100,00	0,000	

(*R*)-3',3'-Bis(4-fluorophenyl)spiro[indoline-3,2'-oxetan]-2-one (5f)

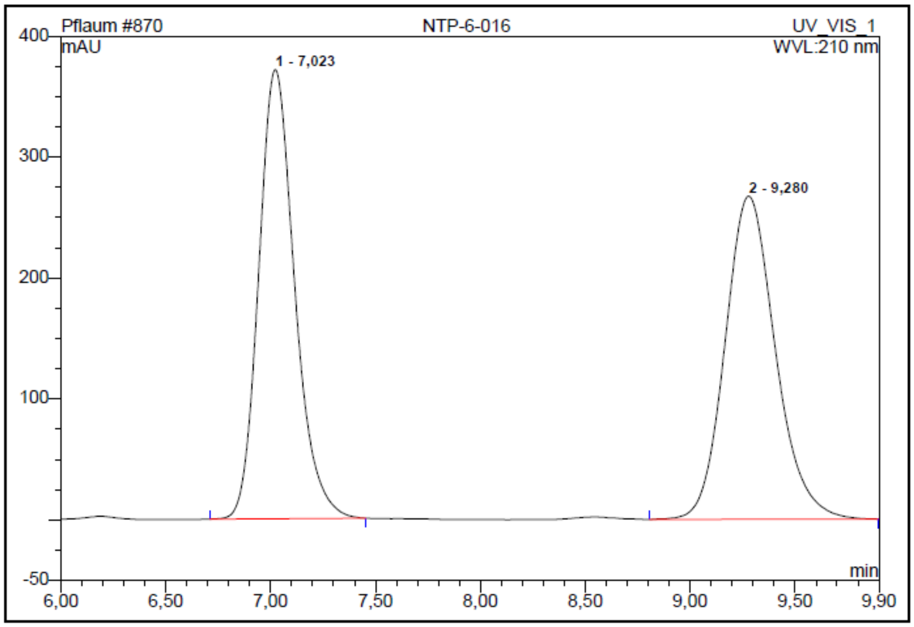

No.	Ret.Time min	Peak Name	Height mAU	Area mAU*min	Rel.Area %	Amount	Type
1	7,02	n.a.	371,428	73,825	49,82	n.a.	BMB
2	9,28	n.a.	267,152	74,360	50,18	n.a.	BMB
Total:			638,580	148,186	100,00	0,000	

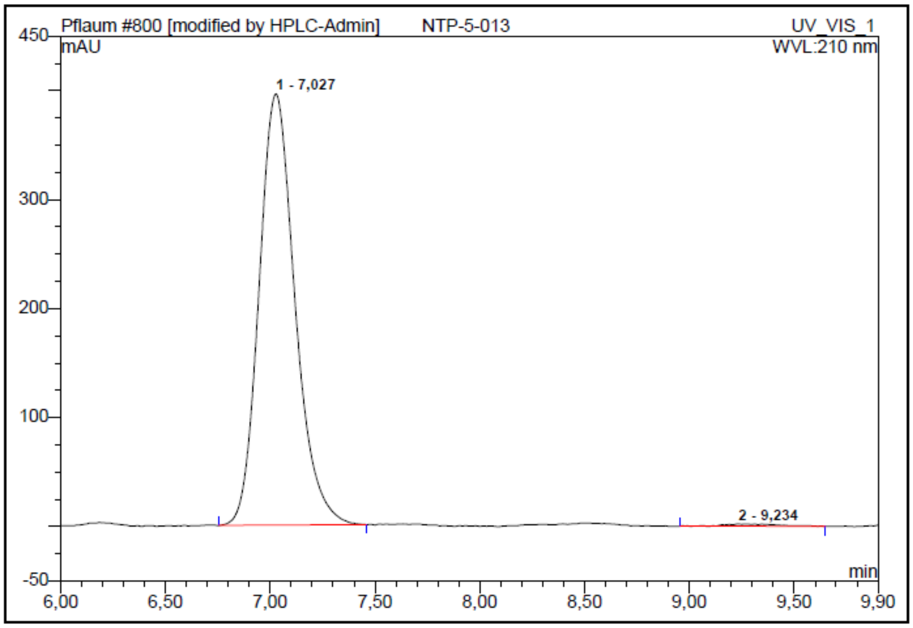

No.	Ret.Time min	Peak Name	Height mAU	Area mAU*min	Rel.Area %	Amount	Type
1	7,03	n.a.	395,599	78,632	99,27	n.a.	BMB
2	9,23	n.a.	2,052	0,578	0,73	n.a.	BMB*
Total:			397,651	79,210	100,00	0,000	

(*R*)-3',3'-Bis(4-bromophenyl)spiro[indoline-3,2'-oxetan]-2-one (5g)

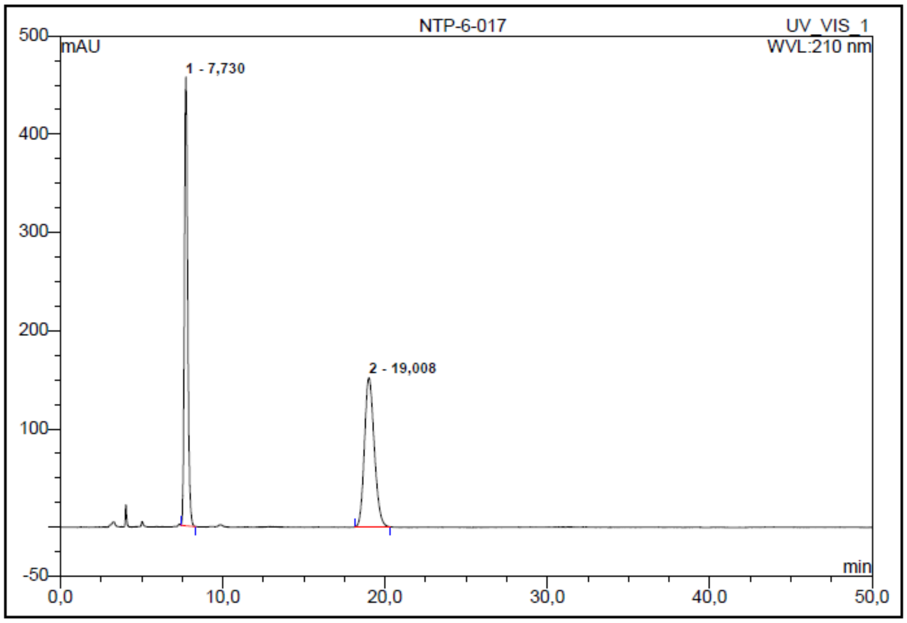

No.	Ret.Time min	Peak Name	Height mAU	Area mAU*min	Rel.Area %	Amount	Type
1	7,73	n.a.	457,066	106,574	49,97	n.a.	BMB
2	19,01	n.a.	151,908	106,705	50,03	n.a.	BMB
Total:			608,974	213,279	100,00	0,000	

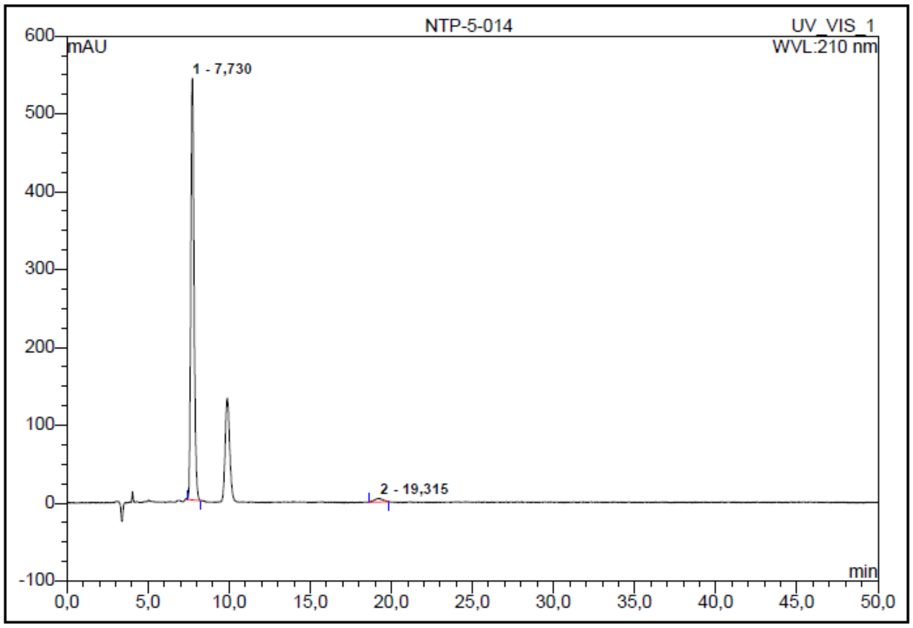

No.	Ret.Time min	Peak Name	Height mAU	Area mAU*min	Rel.Area %	Amount	Type
1	7,73	n.a.	541,915	125,936	97,75	n.a.	BMB
2	19,32	n.a.	4,732	2,895	2,25	n.a.	BMB*
Total:			546,647	128,832	100,00	0,000	

(*R*)-3',3'-Bis(3,5-bis(trifluoromethyl)phenyl)spiro[indoline-3,2'-oxetan]-2-one (5h)

Since Oxetane *ent*-**5h** and **7h** have a similar retention time on OD-RH column the *ee* determination was done using a second column (OJ-RH). Here both oxetane enantiomers have the same retention time but can be separated from **7h**. From the determined areas for the consecutive injections the *ee* was calculated to 98% *ee*

Rac-**5h** on OD-RH:

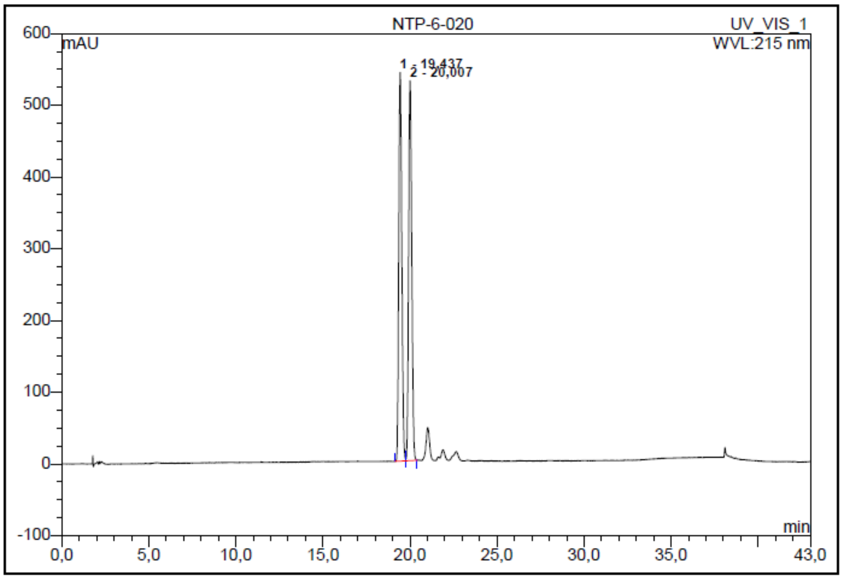

No.	Ret.Time min	Peak Name	Height mAU	Area mAU*min	Rel.Area %	Amount	Type
1	19,44	n.a.	541,952	109,388	49,85	n.a.	BM
2	20,01	n.a.	529,637	110,044	50,15	n.a.	MB
Total:			1071,589	219,433	100,00	0,000	

Rac-**5h** on OJ-RH:

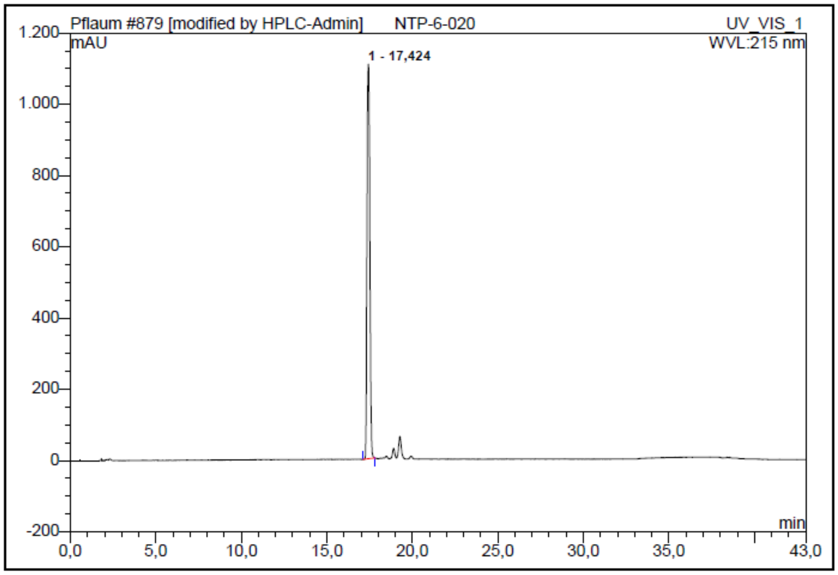

No.	Ret.Time min	Peak Name	Height mAU	Area mAU*min	Rel.Area %	Amount	Type
1	17,42	n.a.	1107,736	213,356	100,00	n.a.	BMB*
Total:			1107,736	213,356	100,00	0,000	

5h on OD-RH: (signal at 20.1 min shows specific absorption of **7h**)

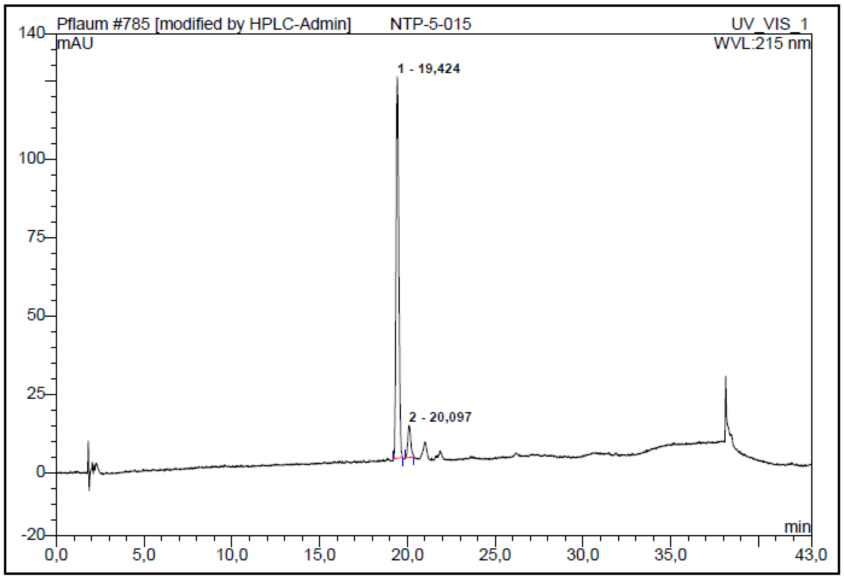

No.	Ret.Time min	Peak Name	Height mAU	Area mAU*min	Rel.Area %	Amount	Type
1	19,42	n.a.	121,531	22,136	91,71	n.a.	BMB*
2	20,10	n.a.	10,264	2,002	8,29	n.a.	BMB*
Total:			131,795	24,138	100,00	0,000	

5h on OJ-RH: (signal at 18.28 min shows specific absorption of **7h**)

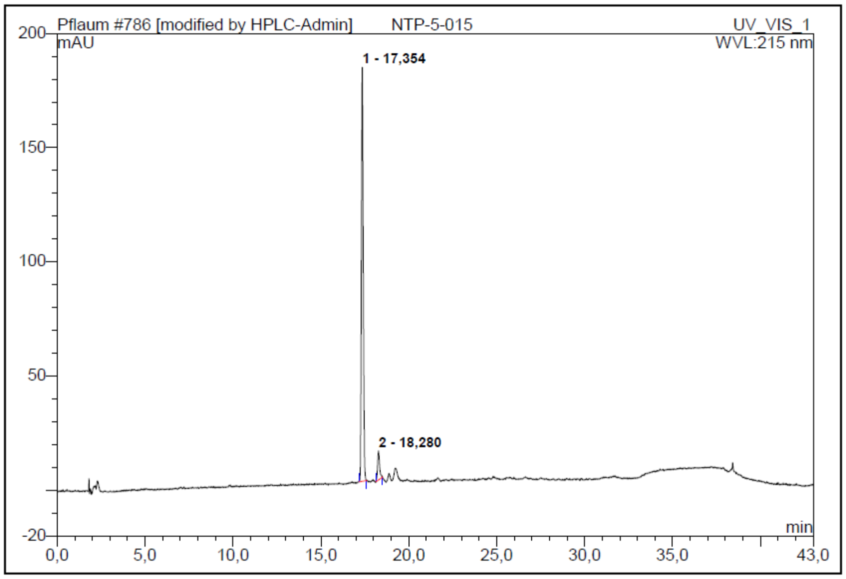

No.	Ret.Time min	Peak Name	Height mAU	Area mAU*min	Rel.Area %	Amount	Type
1	17,35	n.a.	181,324	22,113	92,85	n.a.	BMB*
2	18,28	n.a.	12,790	1,702	7,15	n.a.	BMB*
Total:			194,114	23,816	100,00	0,000	

(*R*)-3',3'-Bis(3-nitrophenyl)spiro[indoline-3,2'-oxetan]-2-one (5i)

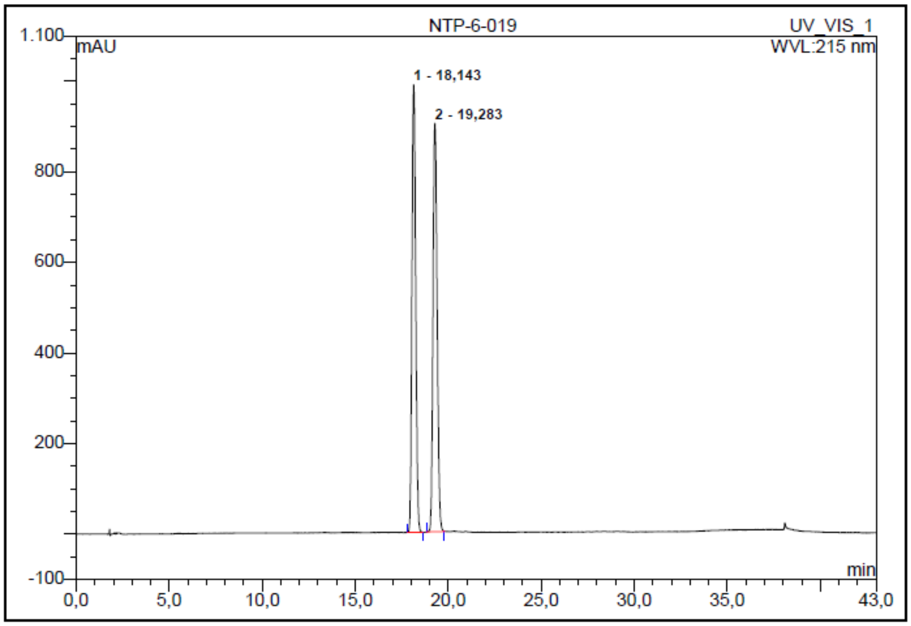

No.	Ret.Time min	Peak Name	Height mAU	Area mAU*min	Rel.Area %	Amount	Type
1	18,14	n.a.	988,435	231,175	49,95	n.a.	BMB
2	19,28	n.a.	902,074	231,663	50,05	n.a.	BMB
Total:			1890,509	462,838	100,00	0,000	

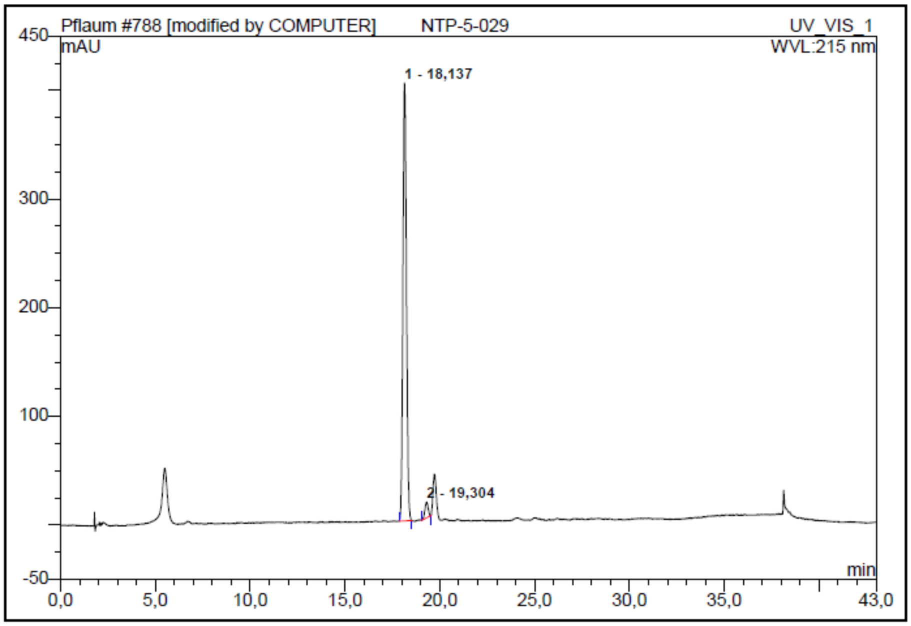

No.	Ret.Time min	Peak Name	Height mAU	Area mAU*min	Rel.Area %	Amount	Type
1	18,14	n.a.	403,007	87,207	96,86	n.a.	BMB
2	19,30	n.a.	14,380	2,824	3,14	n.a.	BMB*
Total:			417,387	90,031	100,00	0,000	

(*R*)-(2-Oxospiro[indoline-3,2'-oxetane]-3',3'-diyl)bis(4,1-phenylene) bis(4-methylbenzenesulfonate) (5j)

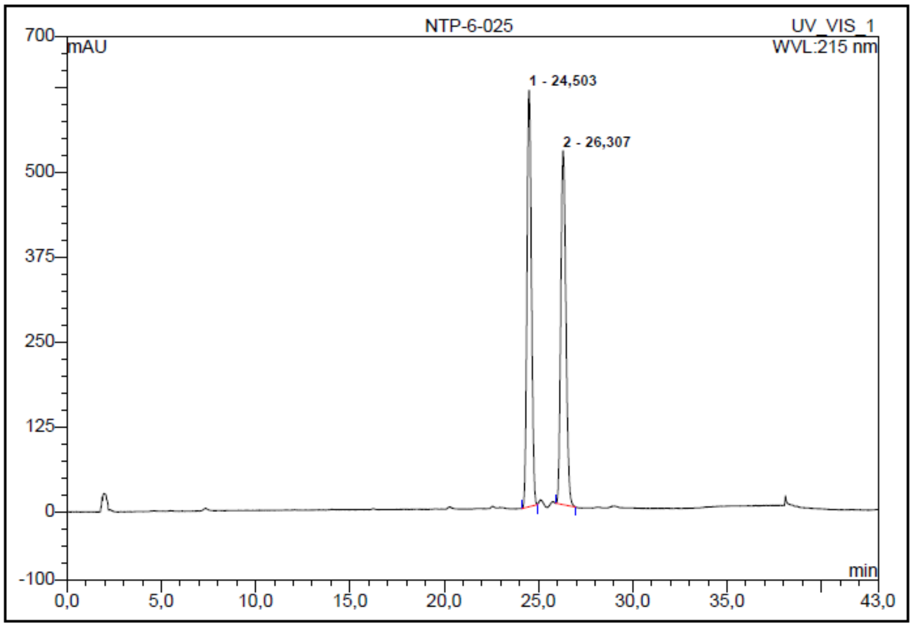

No.	Ret.Time min	Peak Name	Height mAU	Area mAU*min	Rel.Area %	Amount	Type
1	24,50	n.a.	612,938	168,080	50,65	n.a.	BMB
2	26,31	n.a.	520,525	163,739	49,35	n.a.	BMB
Total:			1133,463	331,819	100,00	0,000	

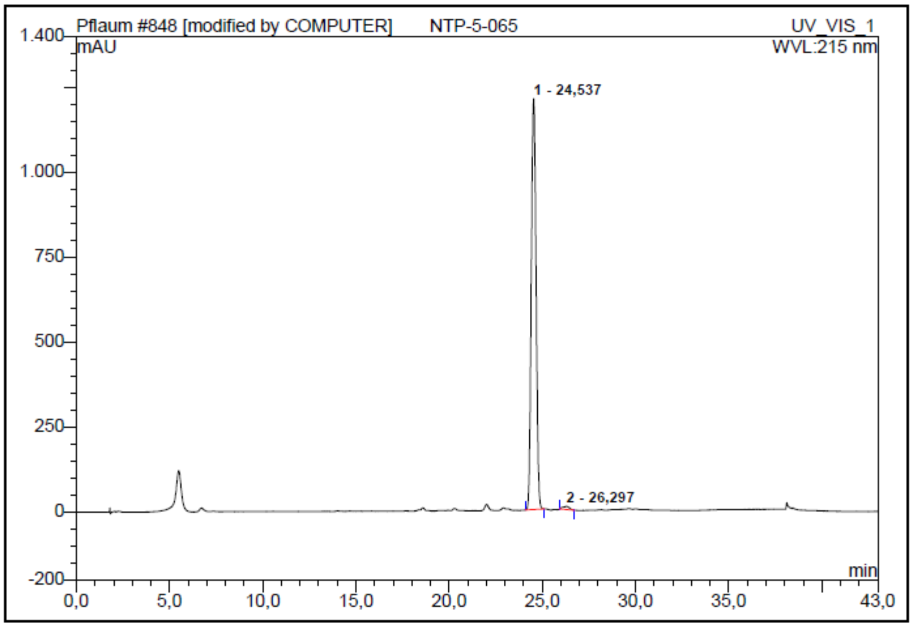

No.	Ret.Time min	Peak Name	Height mAU	Area mAU*min	Rel.Area %	Amount	Type
1	24,54	n.a.	1208,506	361,504	98,97	n.a.	BMB
2	26,30	n.a.	9,218	3,767	1,03	n.a.	BMB*
Total:			1217,724	365,271	100,00	0,000	

(*R*)-(2-Oxospiro[indoline-3,2'-oxetane]-3',3'-diyl)bis(4,1-phenylene) bis(2,2-dimethyl-propanoate) (5k)

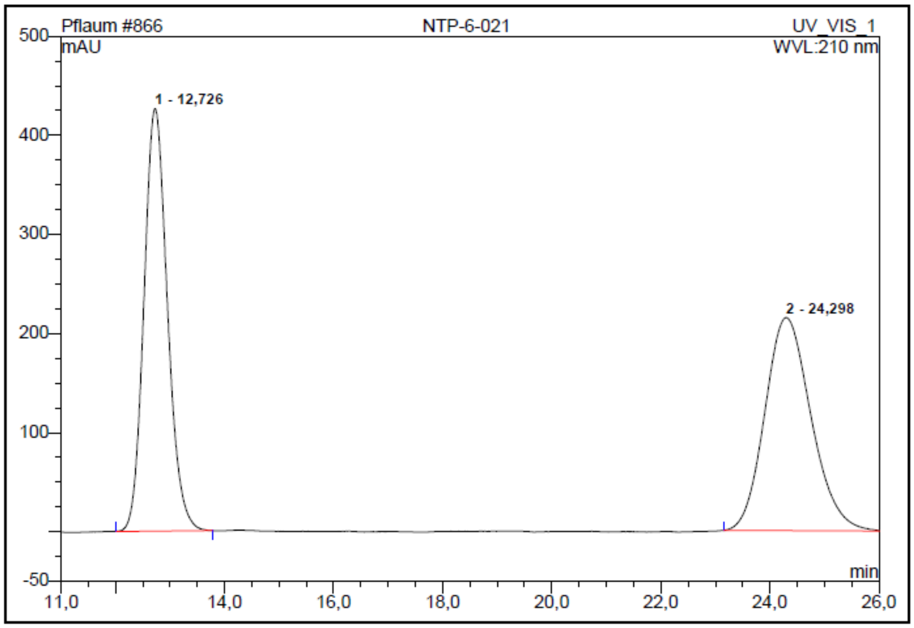

No.	Ret.Time min	Peak Name	Height mAU	Area mAU*min	Rel.Area %	Amount	Type
1	12,73	n.a.	426,445	212,231	50,12	n.a.	BMB
2	24,30	n.a.	215,059	211,195	49,88	n.a.	BMB
Total:			641,504	423,425	100,00	0,000	

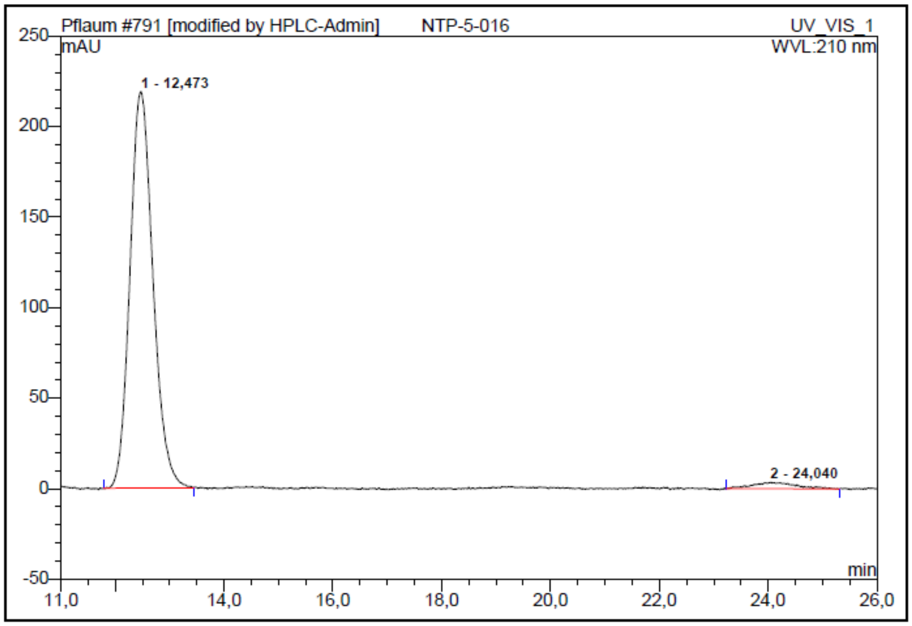

No.	Ret.Time min	Peak Name	Height mAU	Area mAU*min	Rel.Area %	Amount	Type
1	12,47	n.a.	218,948	107,146	96,99	n.a.	BMB*
2	24,04	n.a.	3,534	3,322	3,01	n.a.	BMB*
Total:			222,482	110,468	100,00	0,000	

(*R*)-5-Fluoro-3',3'-bis(4-fluorophenyl)spiro[indoline-3,2'-oxetan]-2-one (5l)

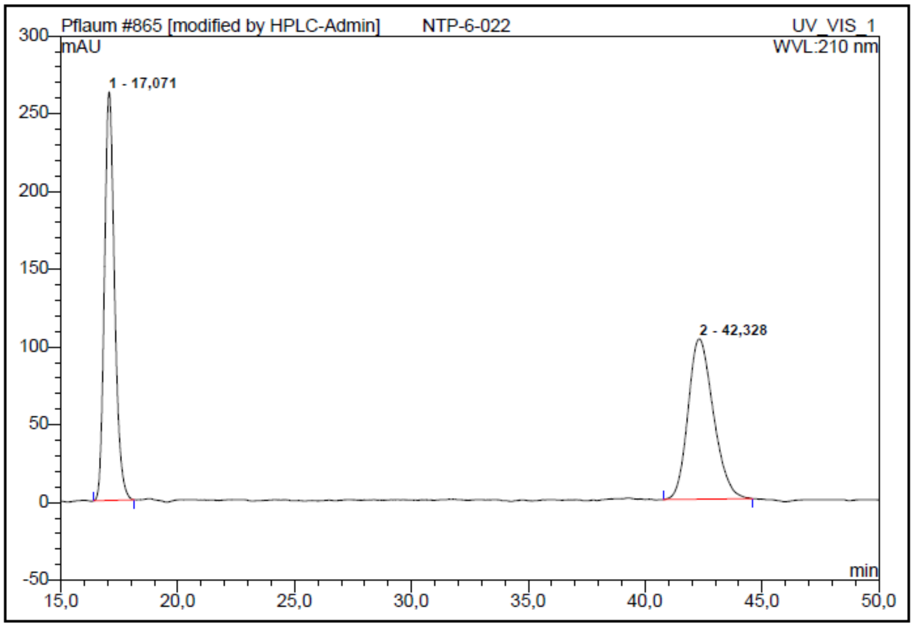

No.	Ret.Time min	Peak Name	Height mAU	Area mAU*min	Rel.Area %	Amount	Type
1	17,07	n.a.	262,764	131,980	50,43	n.a.	BMB
2	42,33	n.a.	103,165	129,737	49,57	n.a.	BMB*
Total:			365,928	261,717	100,00	0,000	

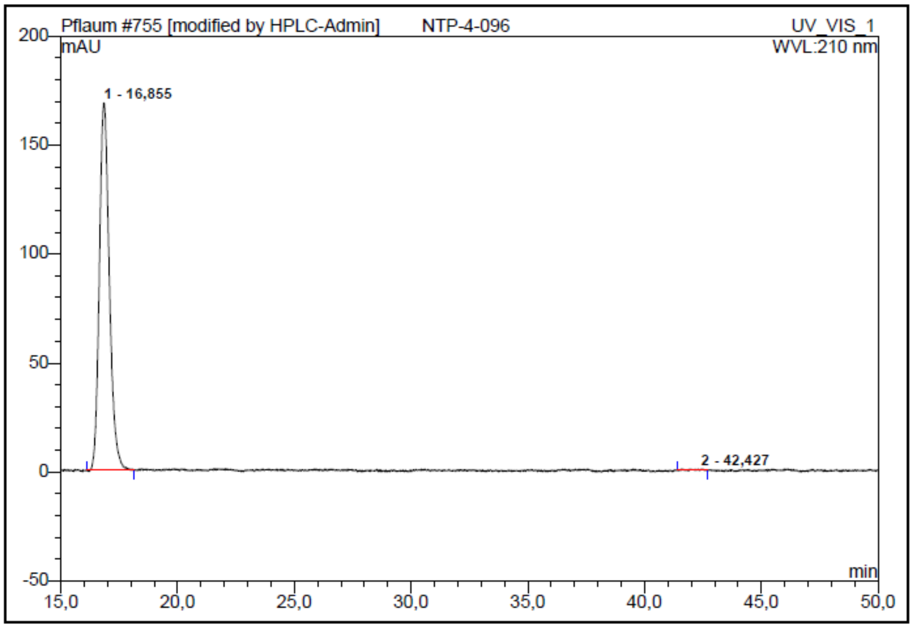

No.	Ret.Time min	Peak Name	Height mAU	Area mAU*min	Rel.Area %	Amount	Type
1	16,85	n.a.	168,650	83,727	99,61	n.a.	BMB*
2	42,43	n.a.	0,661	0,328	0,39	n.a.	BMB*
Total:			169,311	84,055	100,00	0,000	

(*R*)-5-Fluoro-3',3'-di-*p*-tolylspiro[indoline-3,2'-oxetan]-2-one (5m)

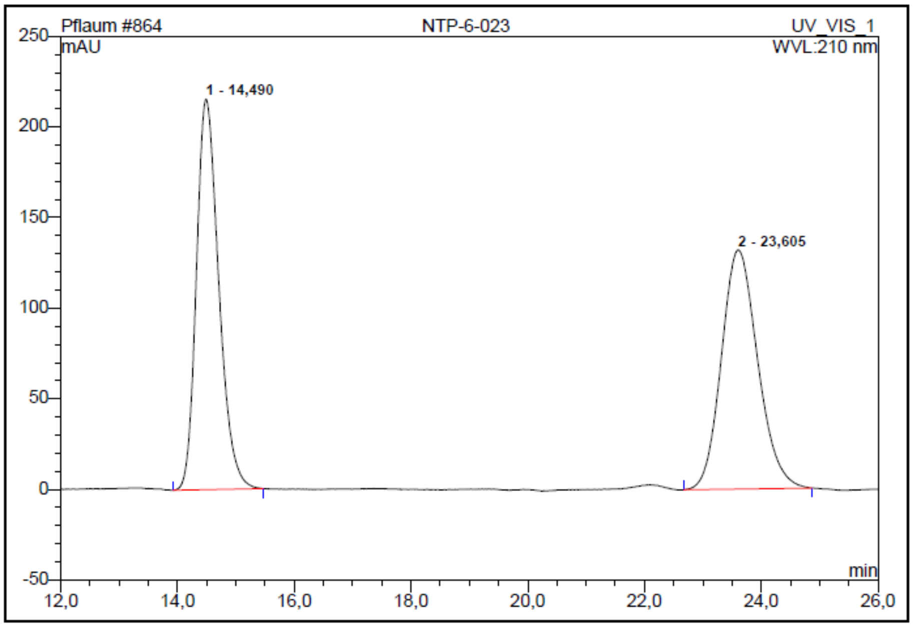

No.	Ret.Time min	Peak Name	Height mAU	Area mAU*min	Rel.Area %	Amount	Type
1	14,49	n.a.	215,400	95,644	50,12	n.a.	BMB
2	23,61	n.a.	131,944	95,198	49,88	n.a.	BMB
Total:			347,344	190,842	100,00	0,000	

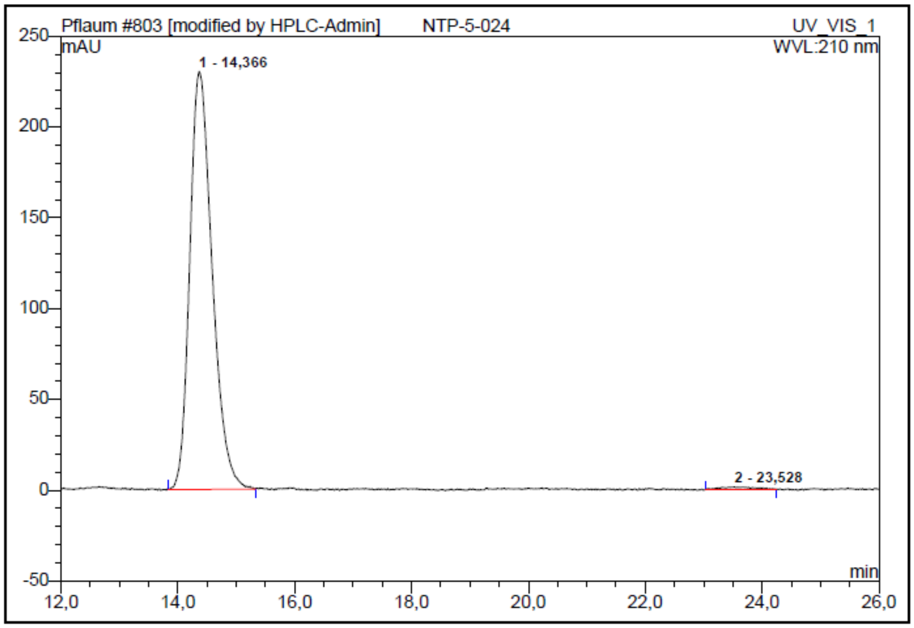

No.	Ret.Time min	Peak Name	Height mAU	Area mAU*min	Rel.Area %	Amount	Type
1	14,37	n.a.	229,840	102,098	99,07	n.a.	BMB*
2	23,53	n.a.	1,453	0,963	0,93	n.a.	BMB*
Total:			231,293	103,061	100,00	0,000	

(*R*)-3',3'-Bis(4-chlorophenyl)-5-fluorospiro[indoline-3,2'-oxetan]-2-one (5n)

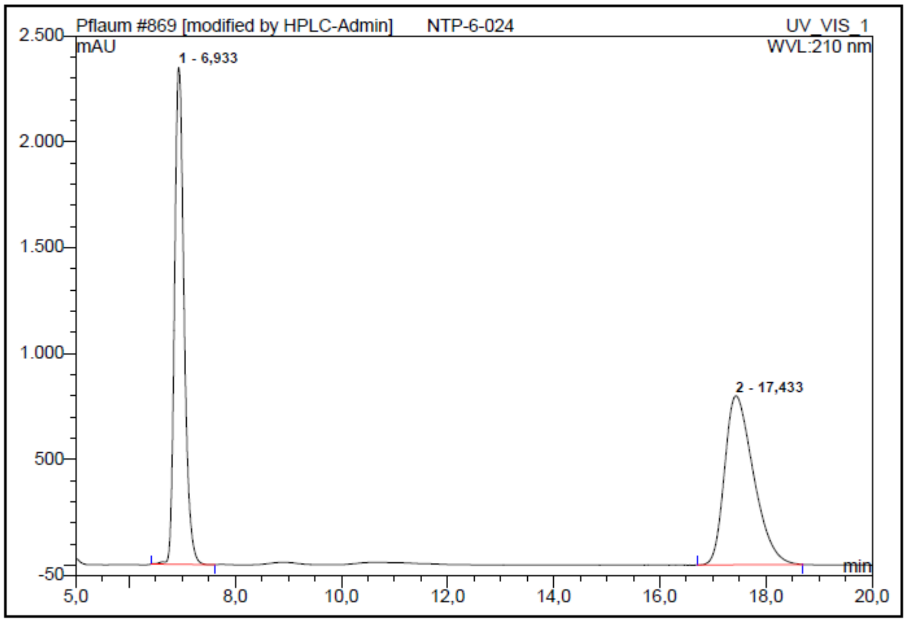

No.	Ret.Time min	Peak Name	Height mAU	Area mAU*min	Rel.Area %	Amount	Type
1	6,93	n.a.	2347,401	491,186	49,27	n.a.	BMB*
2	17,43	n.a.	798,763	505,703	50,73	n.a.	BMB*
Total:			3146,164	996,889	100,00	0,000	

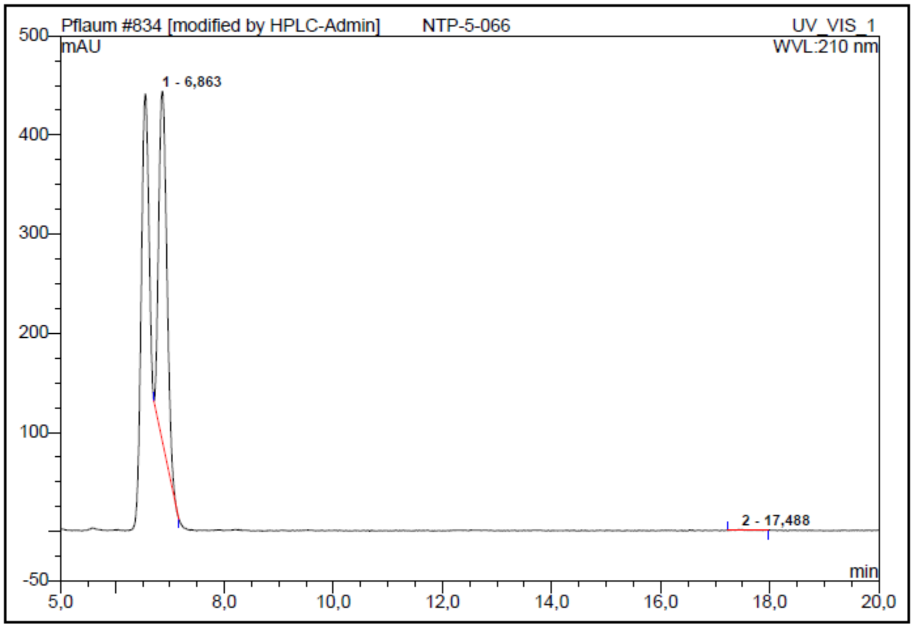

No.	Ret.Time min	Peak Name	Height mAU	Area mAU*min	Rel.Area %	Amount	Type
1	6,86	n.a.	352,638	60,731	99,61	n.a.	BMB*
2	17,49	n.a.	0,840	0,235	0,39	n.a.	BMB*
Total:			353,478	60,967	100,00	0,000	

(*R*)-6-chloro-3',3'-bis(4-chlorophenyl)spiro[indoline-3,2'-oxetan]-2-one (5o)

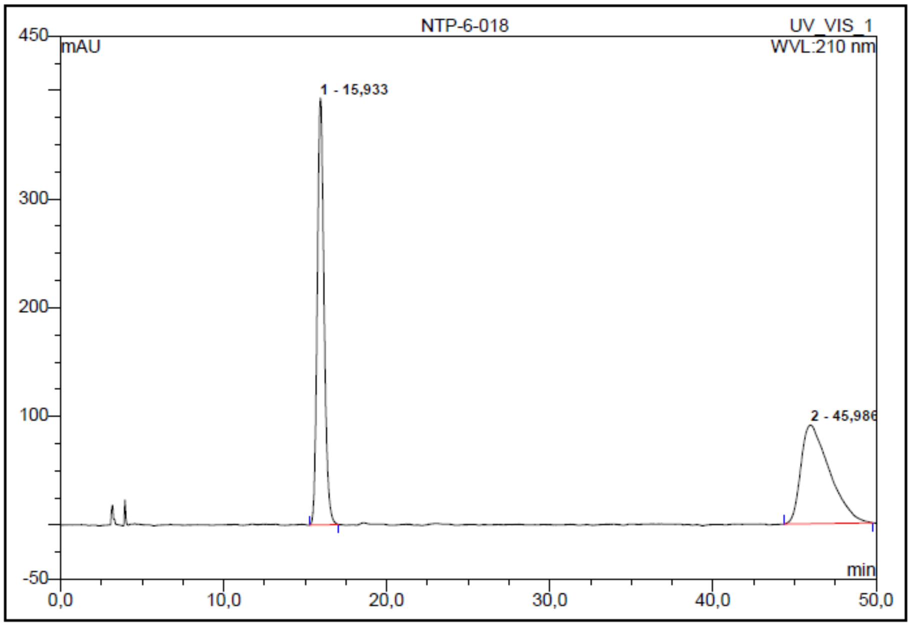

No.	Ret.Time min	Peak Name	Height mAU	Area mAU*min	Rel.Area %	Amount	Type
1	15,93	n.a.	392,493	190,352	50,72	n.a.	BMB
2	45,99	n.a.	90,678	184,929	49,28	n.a.	BMB*
Total:			483,171	375,281	100,00	0,000	

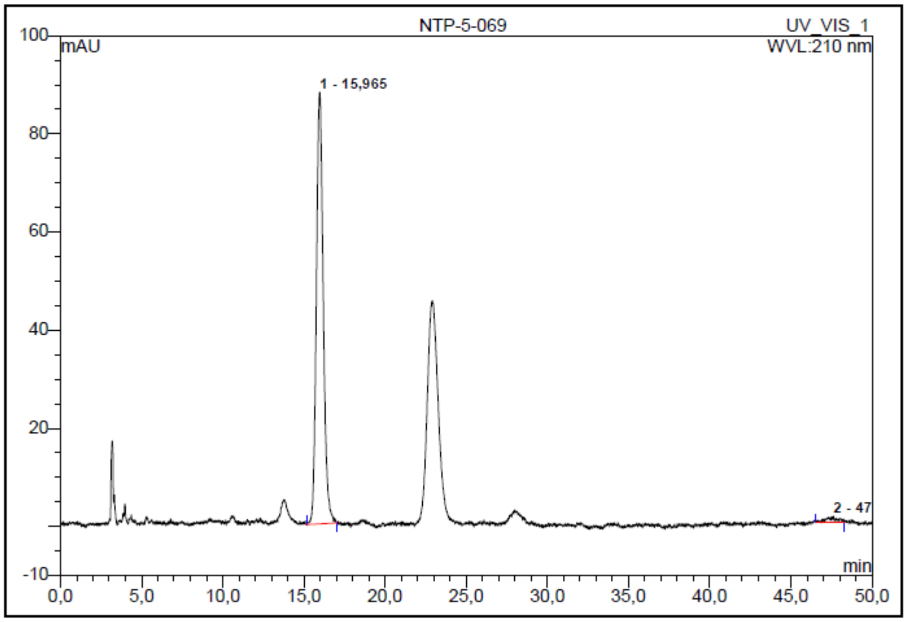

No.	Ret.Time min	Peak Name	Height mAU	Area mAU*min	Rel.Area %	Amount	Type
1	15,96	n.a.	87,985	43,243	97,92	n.a.	BMB*
2	47,62	n.a.	1,271	0,919	2,08	n.a.	BMB*
Total:			89,256	44,162	100,00	0,000	

3-(Bis(4-chlorophenyl)methylene)indolin-2-one (7a)

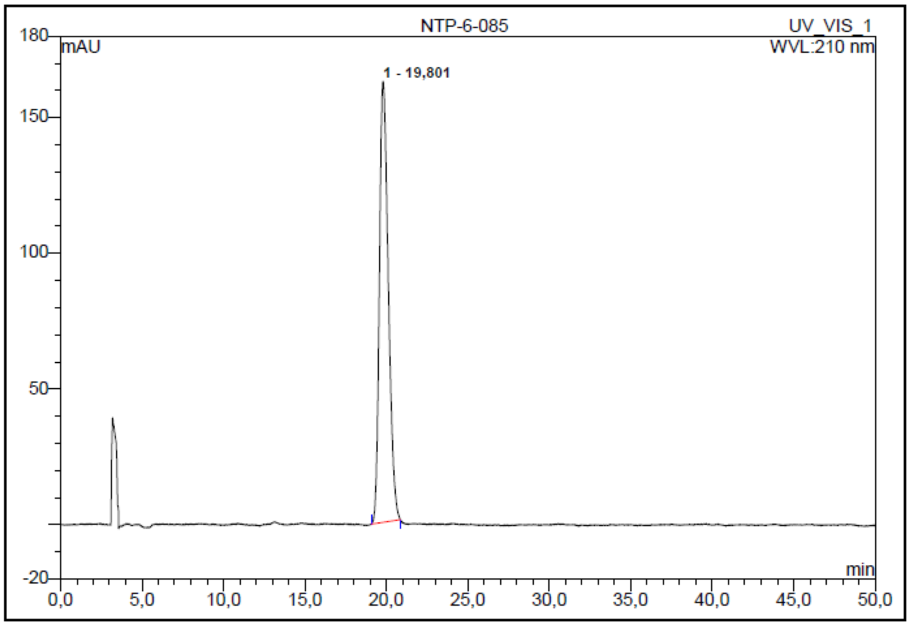

No.	Ret.Time min	Peak Name	Height mAU	Area mAU*min	Rel.Area %	Amount	Type
1	19,80	n.a.	162,419	103,096	100,00	n.a.	BMB
Total:			162,419	103,096	100,00	0,000	

3-(Di-*p*-tolylmethylene)indolin-2-one (7b)

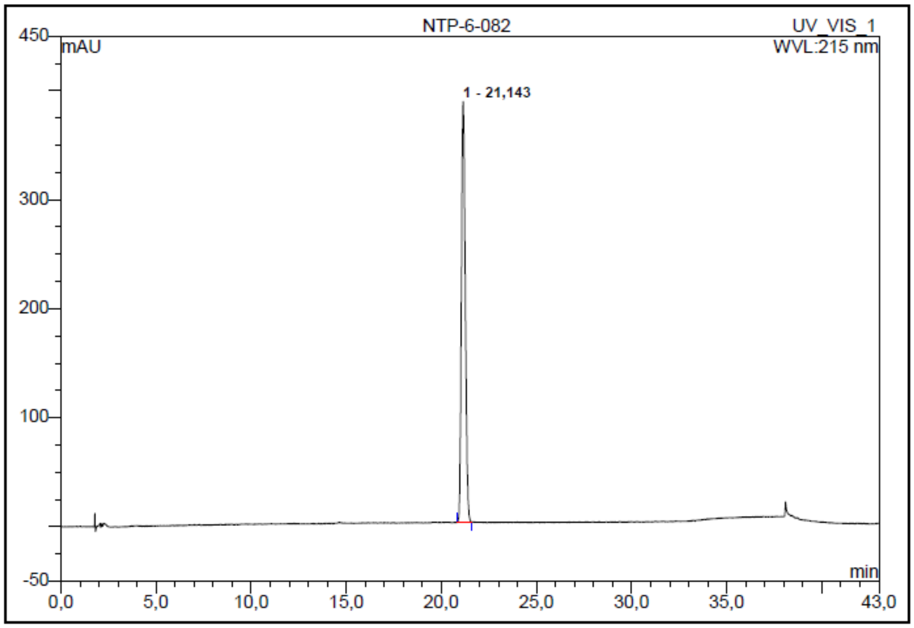

No.	Ret.Time min	Peak Name	Height mAU	Area mAU*min	Rel.Area %	Amount	Type
1	21,14	n.a.	385,672	92,100	100,00	n.a.	BMB
Total:			385,672	92,100	100,00	0,000	

3-(Bis(4-(*tert*-butyl)phenyl)methylene)indolin-2-one (7c)

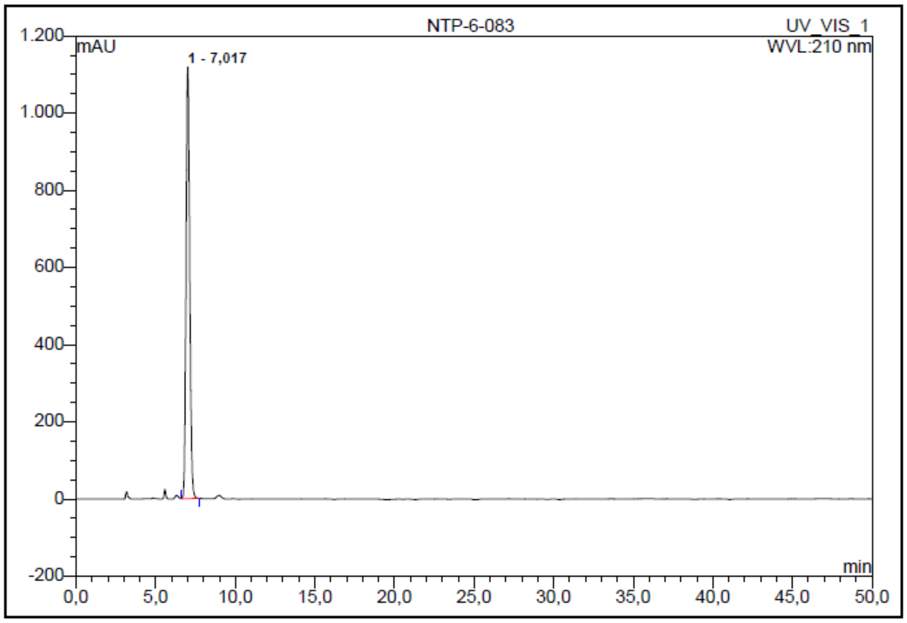

No.	Ret.Time min	Peak Name	Height mAU	Area mAU*min	Rel.Area %	Amount	Type
1	7,02	n.a.	1118,566	297,912	100,00	n.a.	BMB
Total:			1118,566	297,912	100,00	0,000	

3-(Bis(3,5-di-*tert*-butylphenyl)methylene)indolin-2-one (7d)

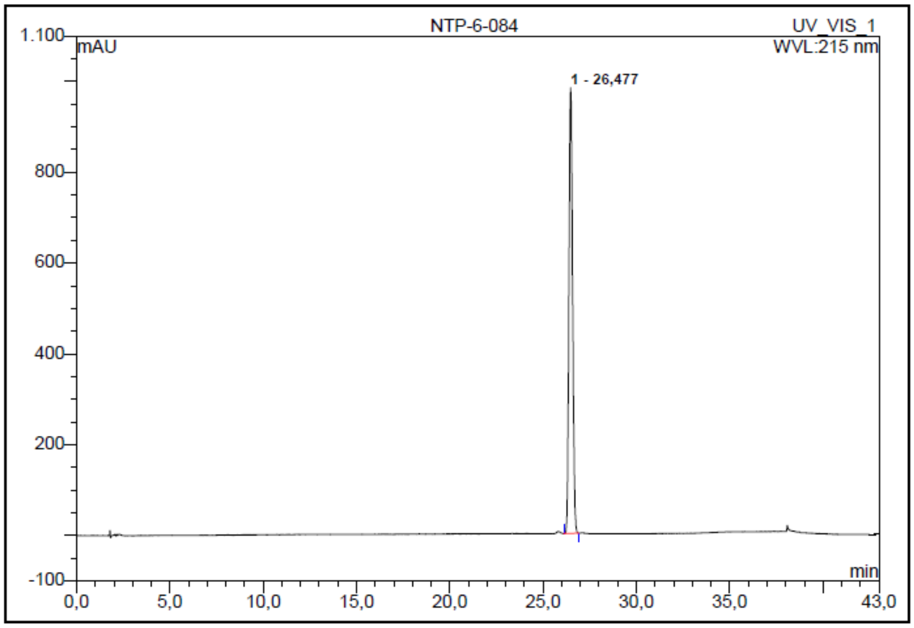

No.	Ret.Time min	Peak Name	Height mAU	Area mAU*min	Rel.Area %	Amount	Type
1	26,48	n.a.	981,376	224,046	100,00	n.a.	BMB
Total:			981,376	224,046	100,00	0,000	

3-(Diphenylmethylene)indolin-2-one (7e)

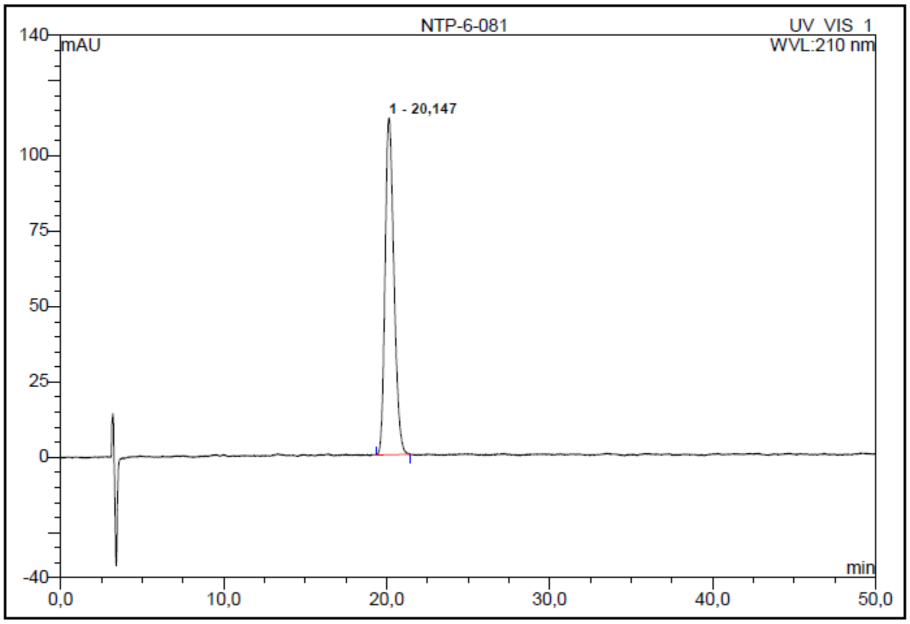

No.	Ret.Time min	Peak Name	Height mAU	Area mAU*min	Rel.Area %	Amount	Type
1	20,15	n.a.	111,754	70,365	100,00	n.a.	BMB*
Total:			111,754	70,365	100,00	0,000	

3-(Bis(4-fluorophenyl)methylene)indolin-2-one (7f)

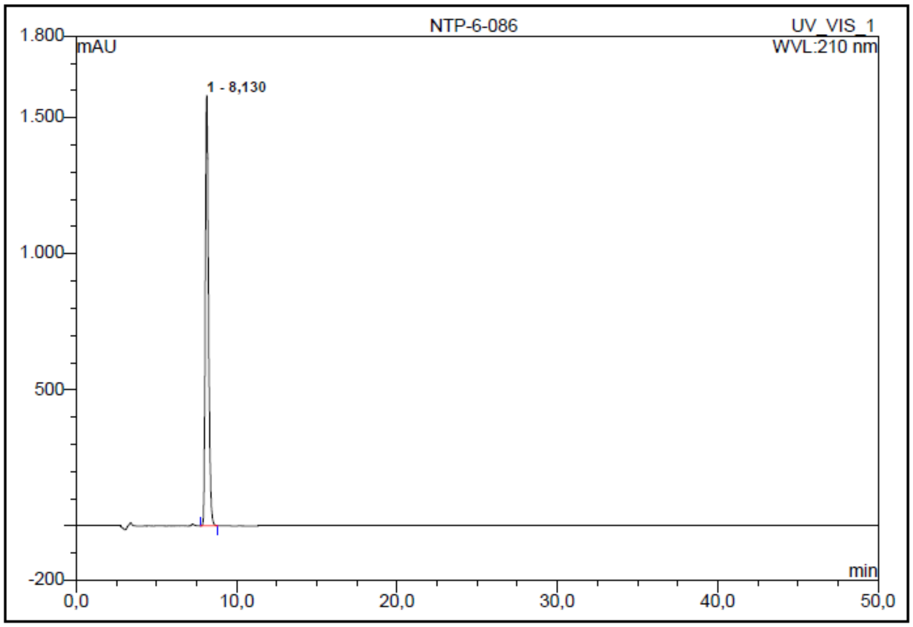

No.	Ret.Time min	Peak Name	Height mAU	Area mAU*min	Rel.Area %	Amount	Type
1	8,13	n.a.	1581,398	368,907	100,00	n.a.	BMB
Total:			1581,398	368,907	100,00	0,000	

3-(Bis(4-bromophenyl)methylene)indolin-2-one (7g)

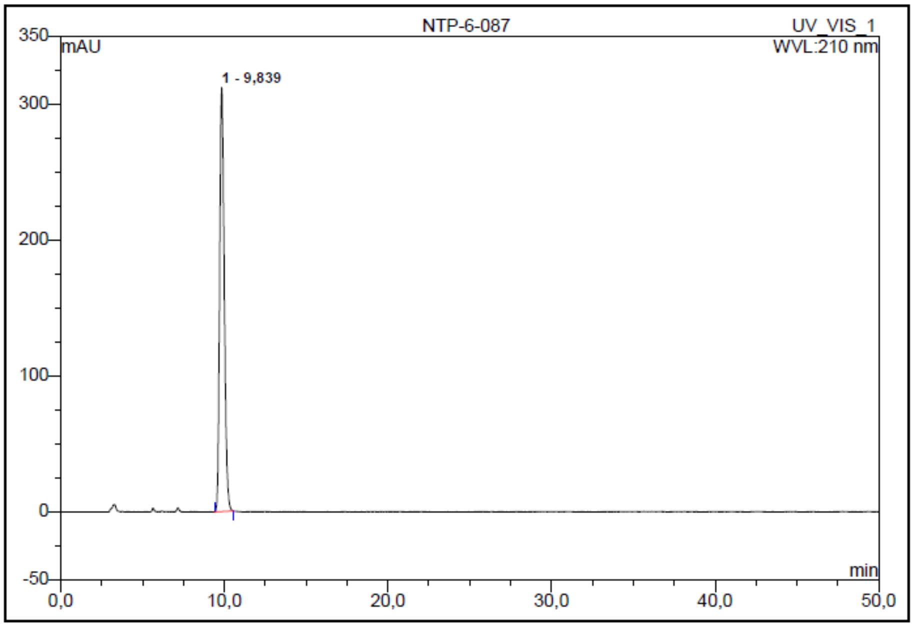

No.	Ret.Time min	Peak Name	Height mAU	Area mAU*min	Rel.Area %	Amount	Type
1	9,84	n.a.	311,741	101,558	100,00	n.a.	BMB
Total:			311,741	101,558	100,00	0,000	

3-(Bis(3,5-bis(trifluoromethyl)phenyl)methylene)indolin-2-one (7h)

3-(bis(3-nitrophenyl)methylene)indolin-2-one (7i)

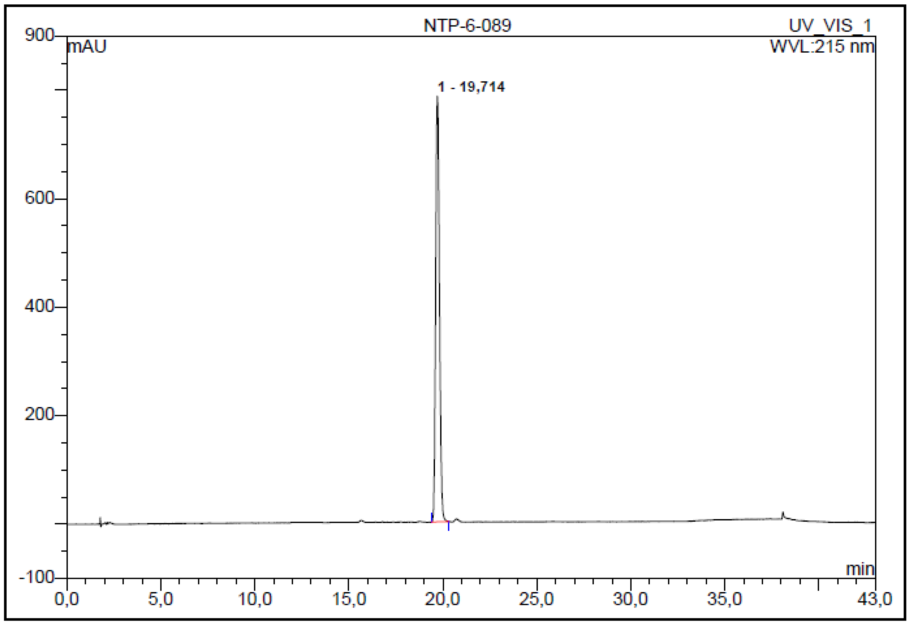

No.	Ret.Time min	Peak Name	Height mAU	Area mAU*min	Rel.Area %	Amount	Type
1	19,71	n.a.	785,008	184,798	100,00	n.a.	BMB
Total:			785,008	184,798	100,00	0,000	

((2-Oxoindolin-3-ylidene)methylene)bis(4,1-phenylene) bis(4-methylbenzenesulfonate) 7j

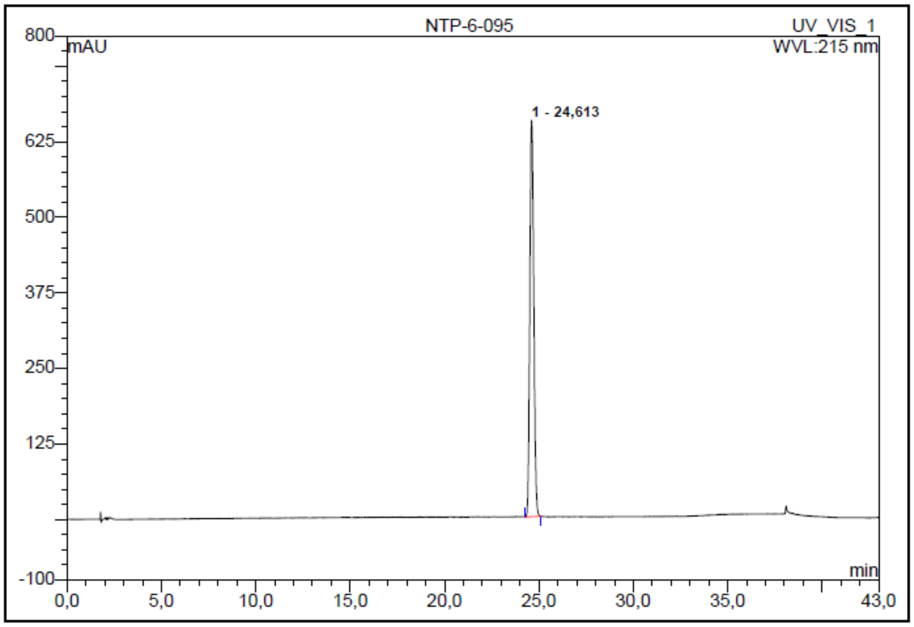

No.	Ret.Time min	Peak Name	Height mAU	Area mAU*min	Rel.Area %	Amount	Type
1	24,61	n.a.	655,613	159,746	100,00	n.a.	BMB
Total:			655,613	159,746	100,00	0,000	

((2-Oxoindolin-3-ylidene)methylene)bis(4,1-phenylene) bis(2,2-dimethylpropanoate) (7k)

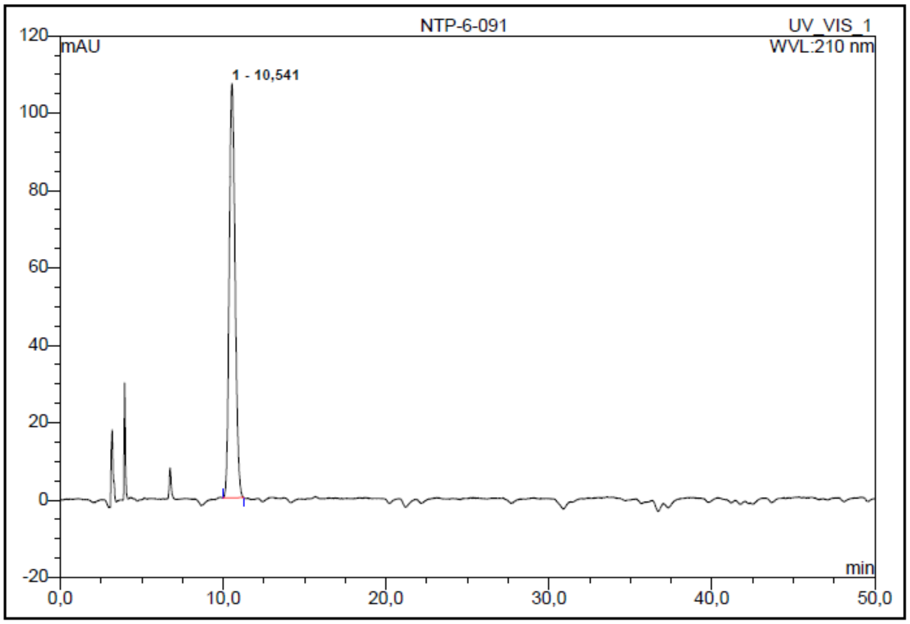

No.	Ret.Time min	Peak Name	Height mAU	Area mAU*min	Rel.Area %	Amount	Type
1	10,54	n.a.	107,085	44,678	100,00	n.a.	BMB
Total:			107,085	44,678	100,00	0,000	

3-(Bis(4-fluorophenyl)methylene)-5-fluoroindolin-2-one (7l)

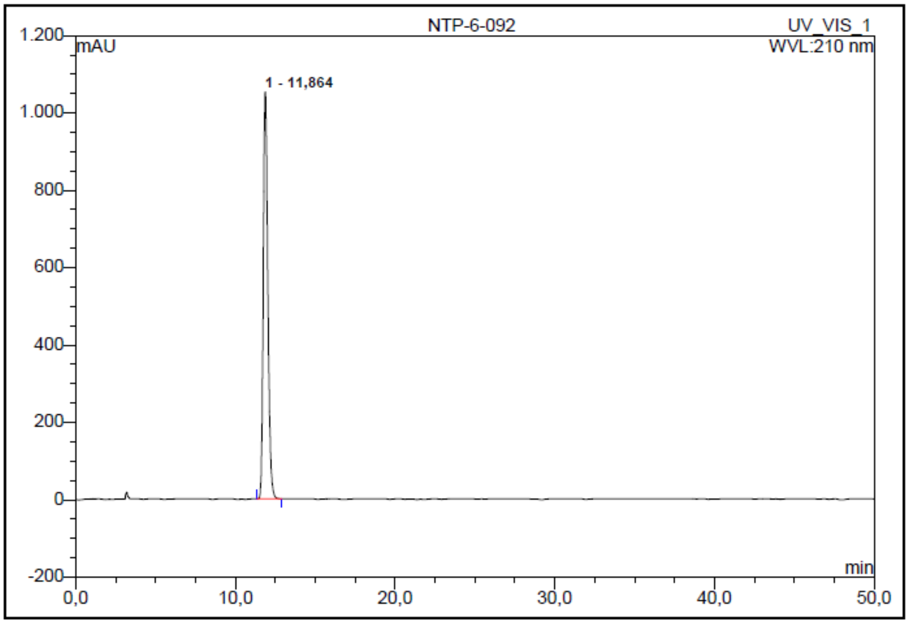

No.	Ret.Time min	Peak Name	Height mAU	Area mAU*min	Rel.Area %	Amount	Type
1	11,86	n.a.	1052,096	350,669	100,00	n.a.	BMB
Total:			1052,096	350,669	100,00	0,000	

3-(Di-*p*-tolylmethylene)-5-fluoroindolin-2-one (7m)

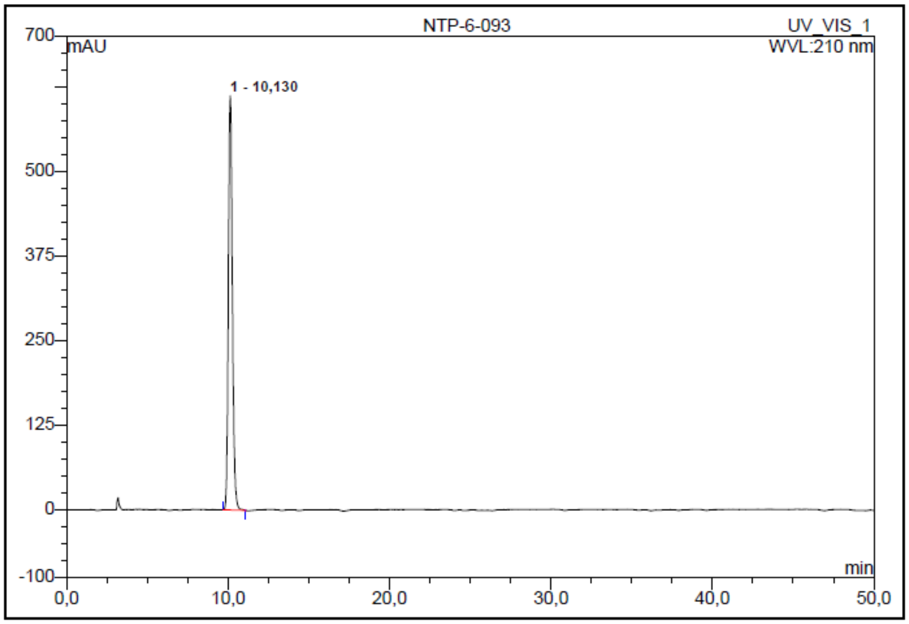

No.	Ret.Time min	Peak Name	Height mAU	Area mAU*min	Rel.Area %	Amount	Type
1	10,13	n.a.	611,896	178,050	100,00	n.a.	BMB
Total:			611,896	178,050	100,00	0,000	

3-(Bis(4-chlorophenyl)methylene)-5-fluoroindolin-2-one (7n)

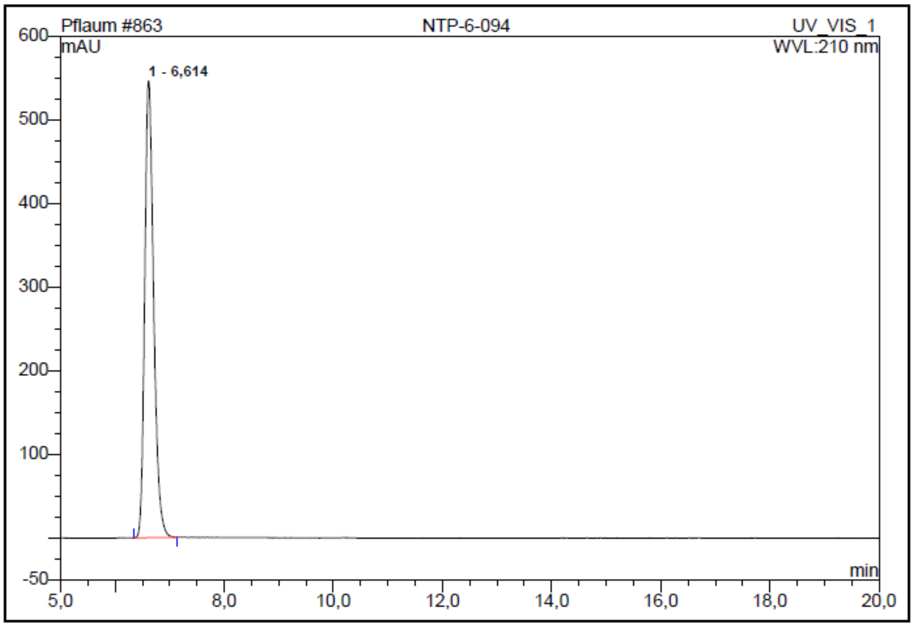

No.	Ret.Time min	Peak Name	Height mAU	Area mAU*min	Rel.Area %	Amount	Type
1	6,61	n.a.	545,890	103,863	100,00	n.a.	BMB
Total:			545,890	103,863	100,00	0,000	

3-(Bis(4-chlorophenyl)methylene)-5-chloroindolin-2-one (7o)

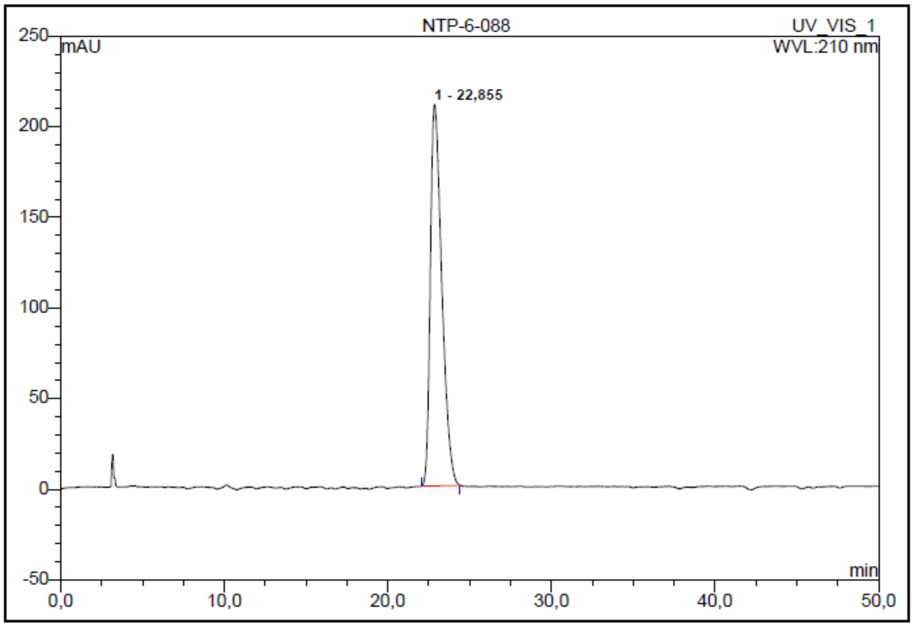

No.	Ret.Time min	Peak Name	Height mAU	Area mAU*min	Rel.Area %	Amount	Type
1	22,86	n.a.	210,371	170,583	100,00	n.a.	BMB
Total:			210,371	170,583	100,00	0,000	

17. Steady-state Absorption Spectra in the UV/Vis Spectral Range

All samples were measured in CH_2Cl_2 (if no further explanation) in a 0.1 cm cuvette at a concentration of c ≈ 10 mM (if no further explanation).

UV/Vis-Spectrum of **6** (measured in $PhCF_3$ with c = 0.5 mM):

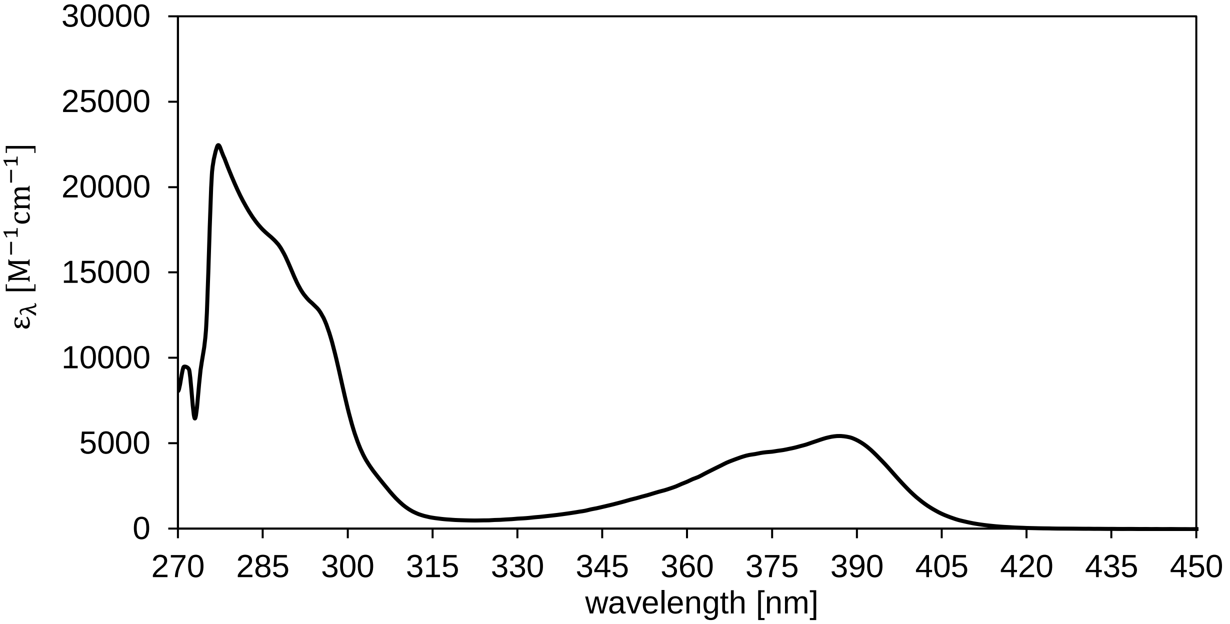

UV/Vis-Spectrum of **8** (measured in $PhCF_3$ with c = 0.5 mM):

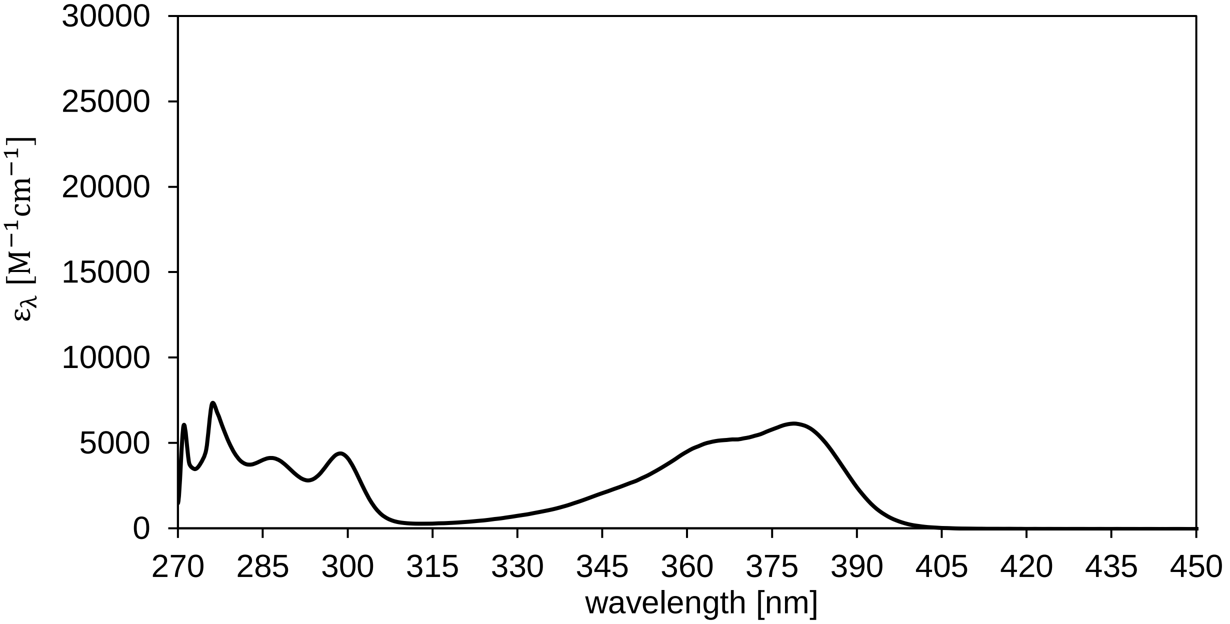

UV/Vis-Spectrum of **7a** (measured in $PhCF_3$ with c = 1.0 mM):

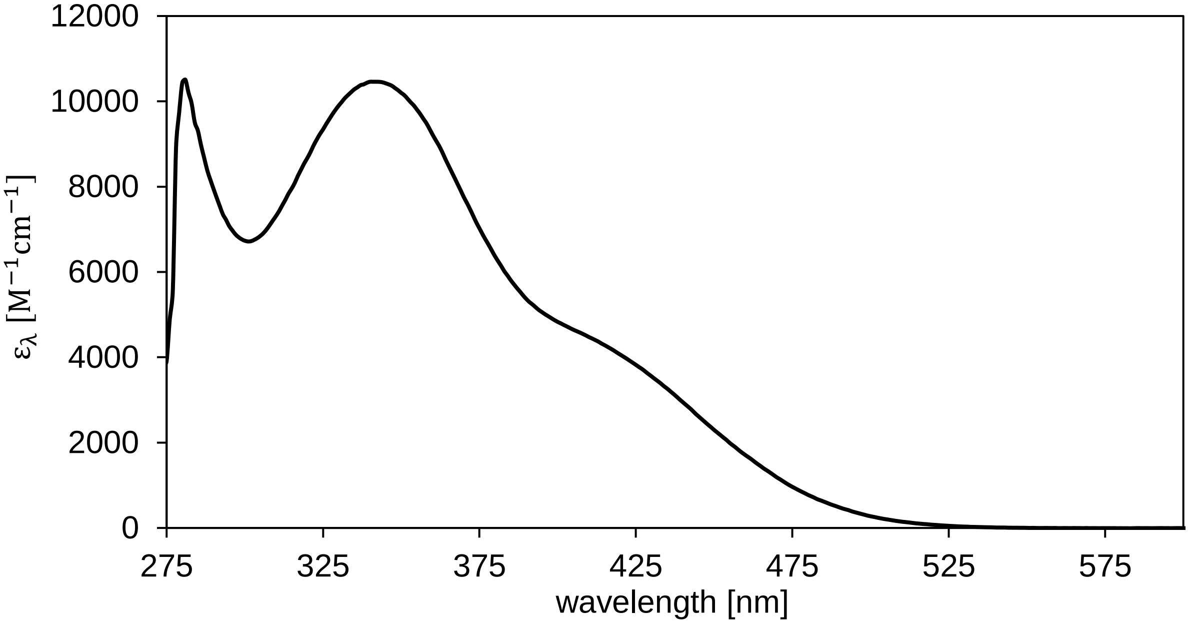

UV/Vis-Spectrum of **10a** (measured in $PhCF_3$ with c = 1.0 mM):

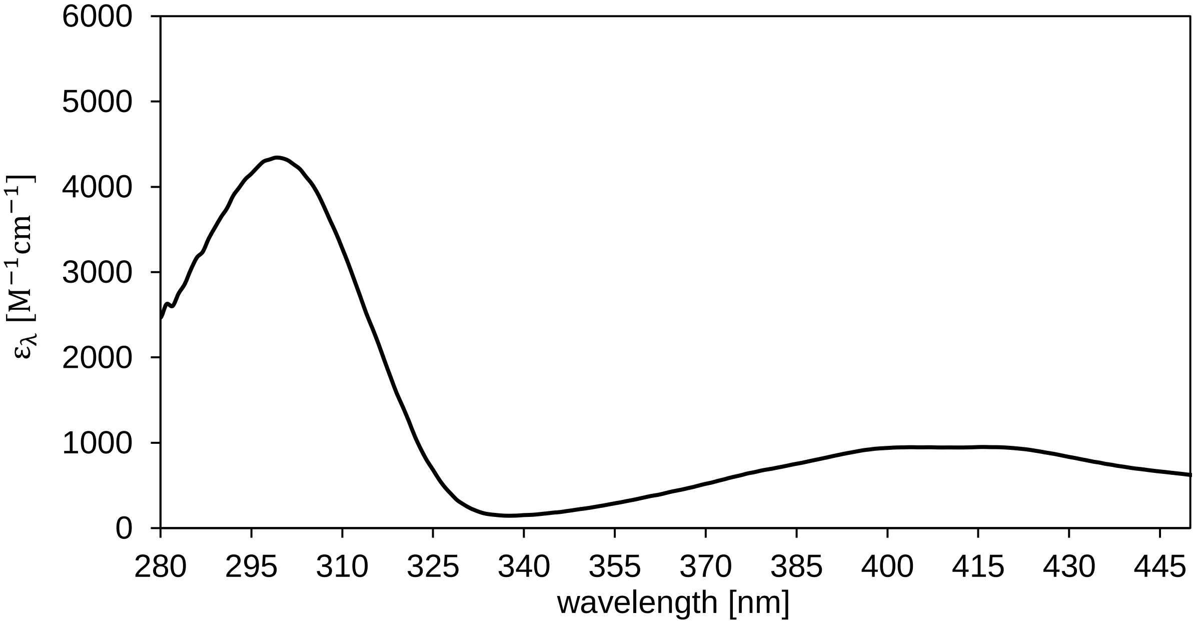

UV/Vis-Spectrum of *rac*-**5a**:

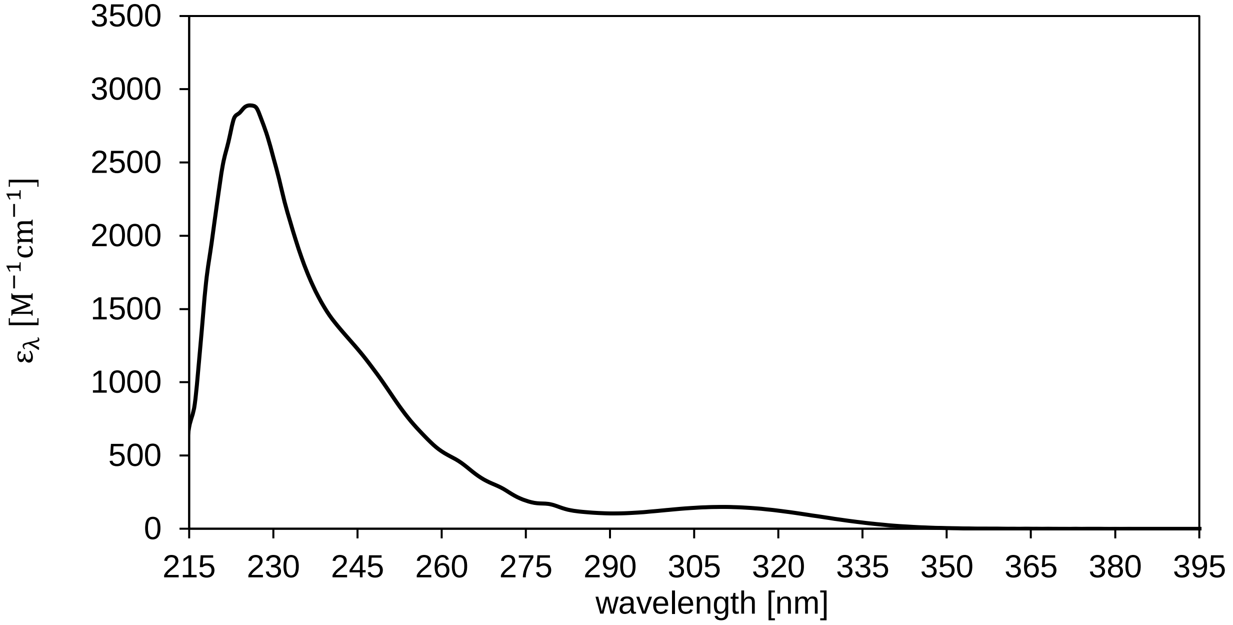

UV/Vis-Spectrum of *rac*-**5b**:

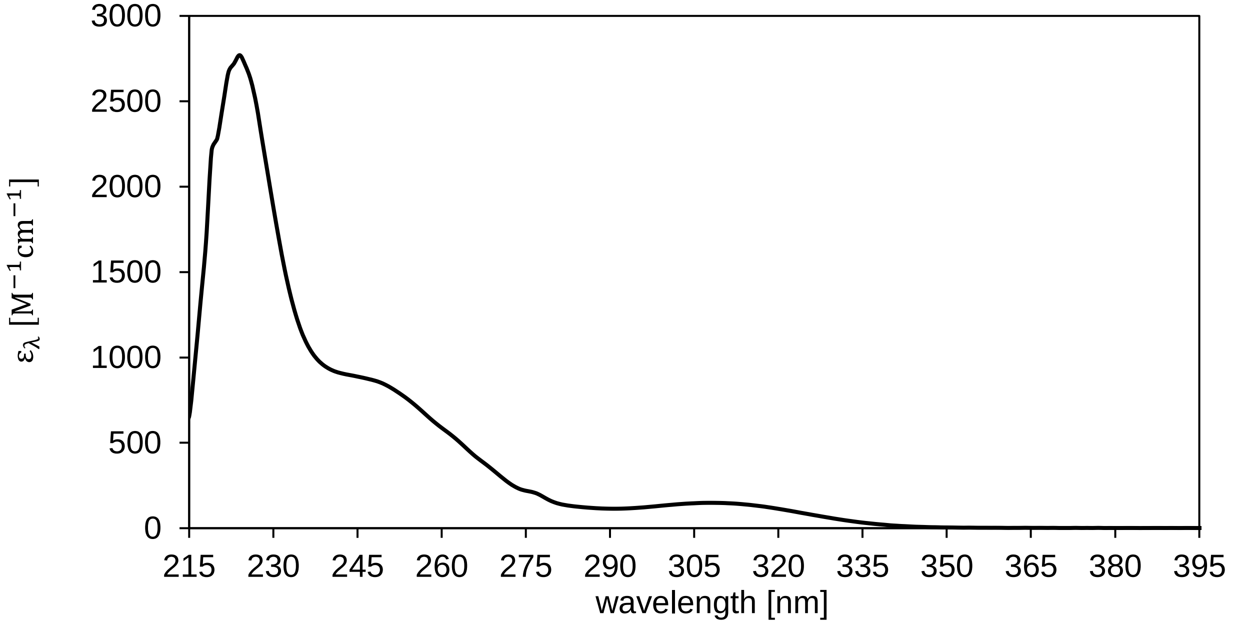

UV/Vis-Spectrum of *rac*-**5c**:

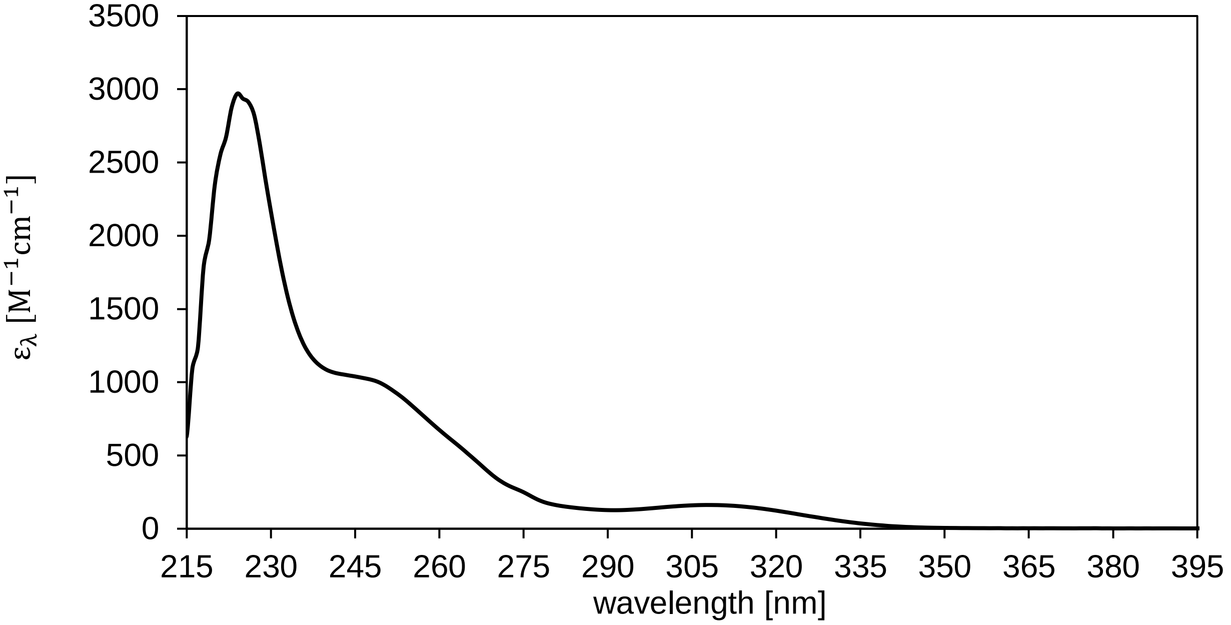

UV/Vis-Spectrum of *rac*-**5d**:

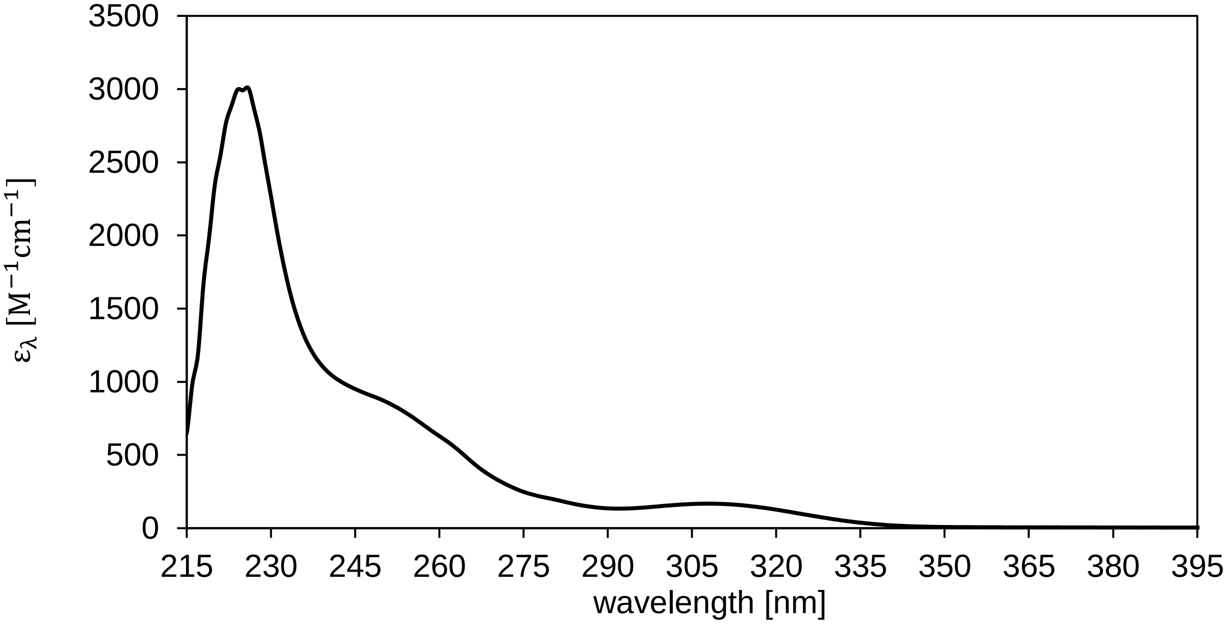

UV/Vis-Spectrum of *rac*-**5e**:

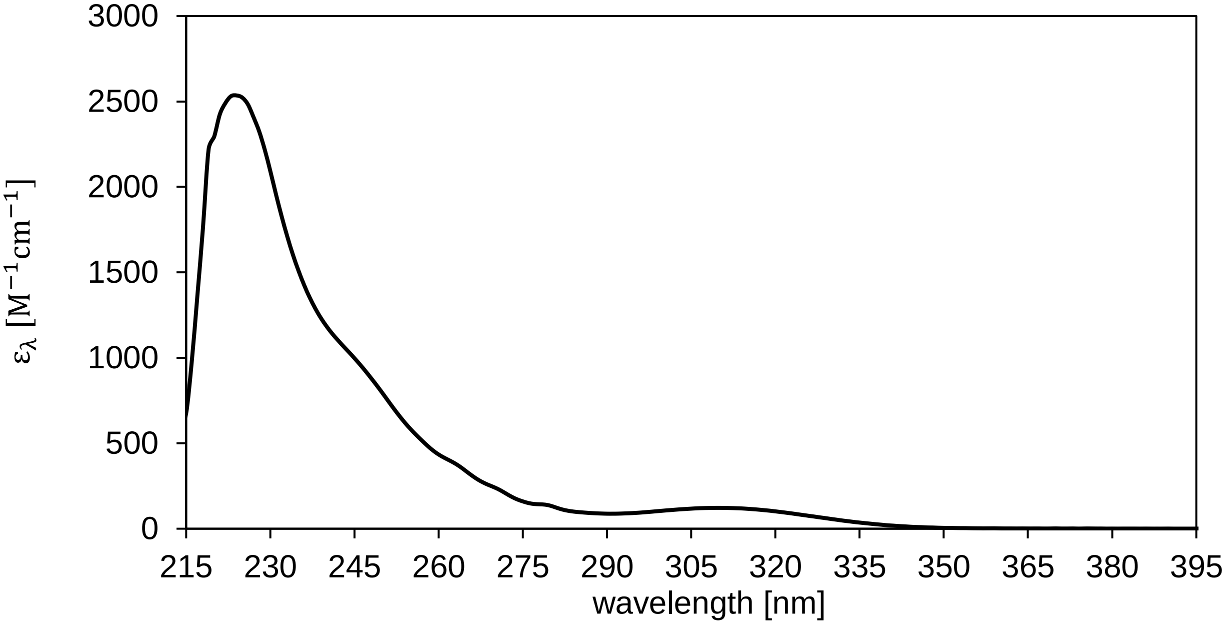

UV/Vis-Spectrum of *rac*-**5f**:

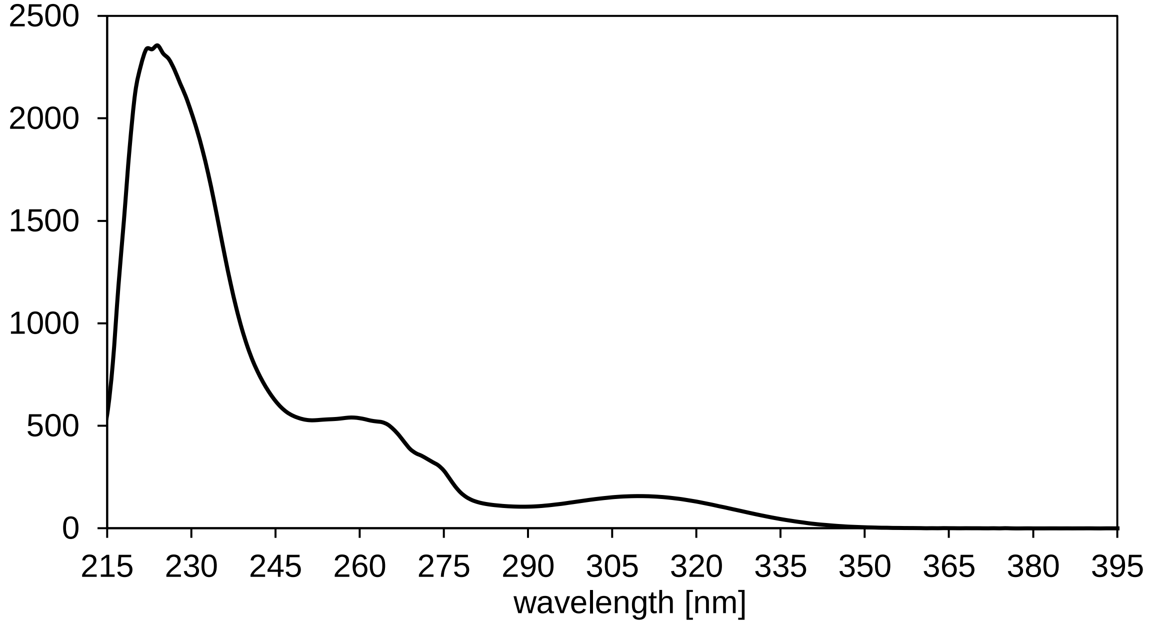

UV/Vis-Spectrum of *rac*-**5g**:

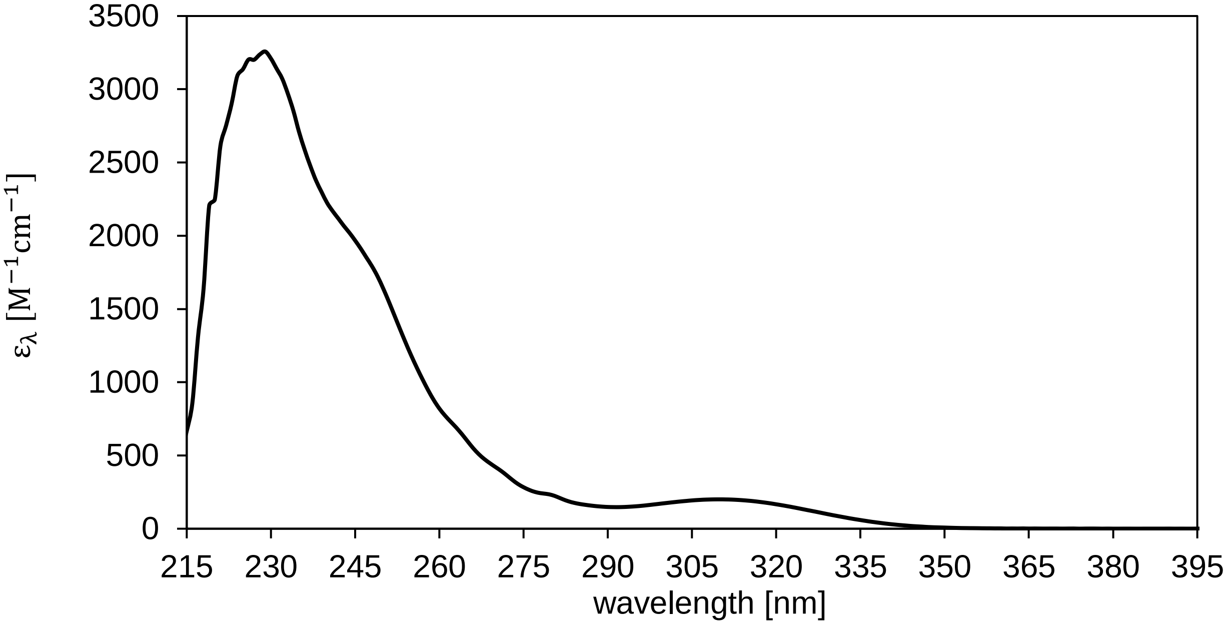

UV/Vis-Spectrum of *rac*-**5h**:

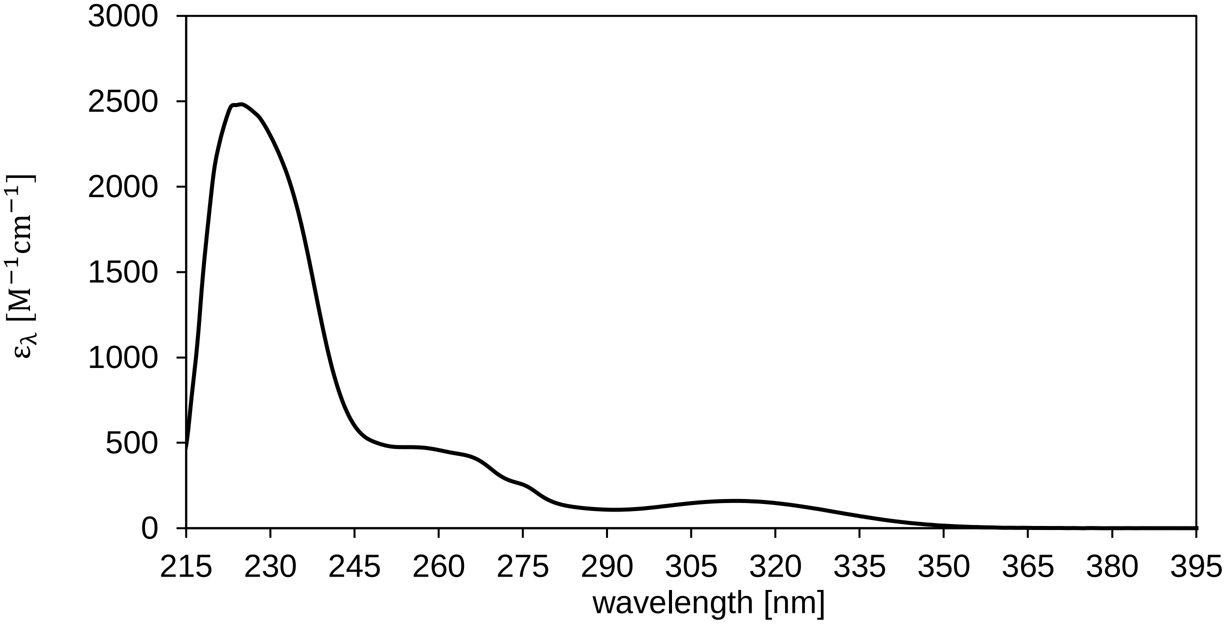

UV/Vis-Spectrum of *rac*-**5i** (c = 5.0 mM):

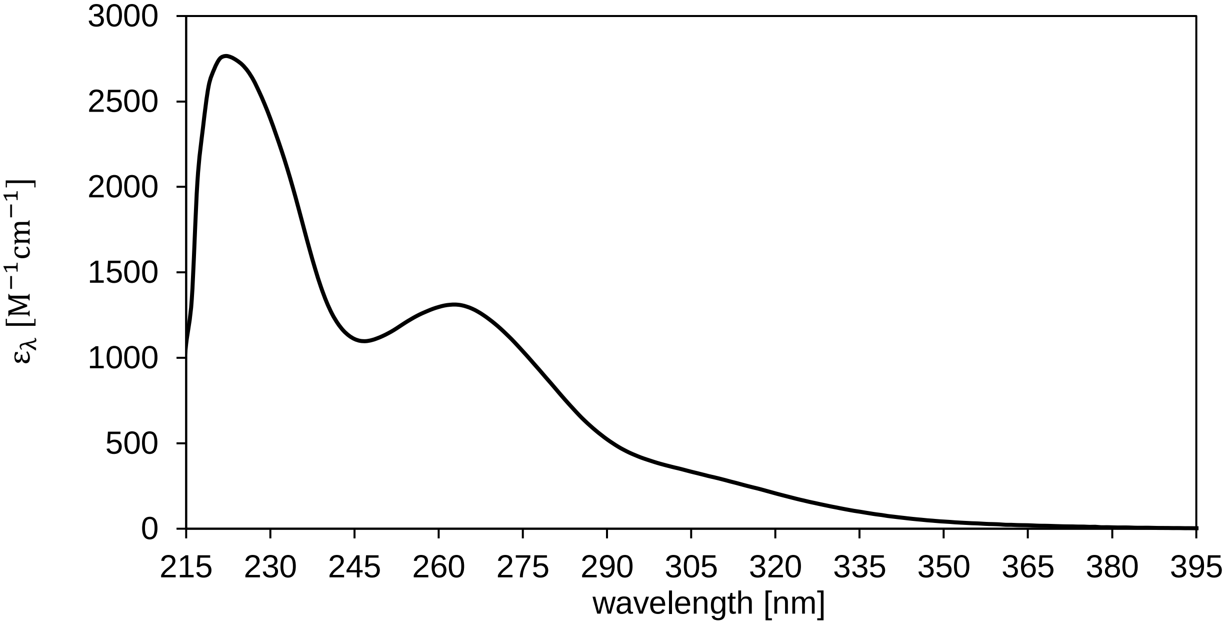

UV/Vis-Spectrum of *rac*-**5j**:

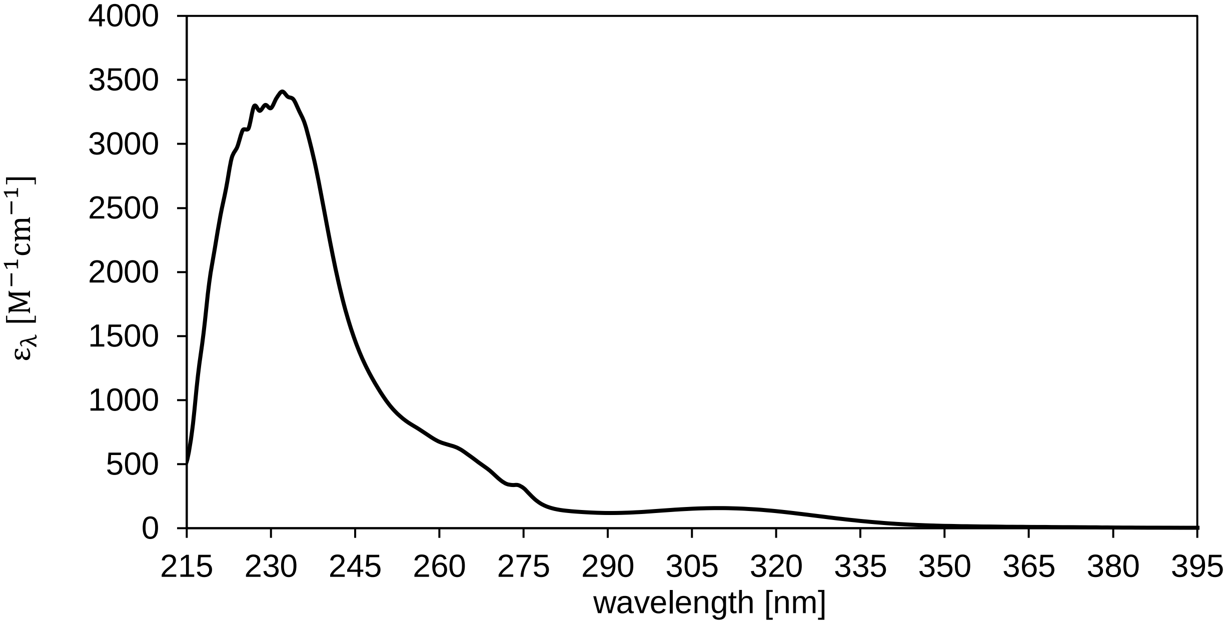

UV/Vis-Spectrum of *rac*-**5k**:

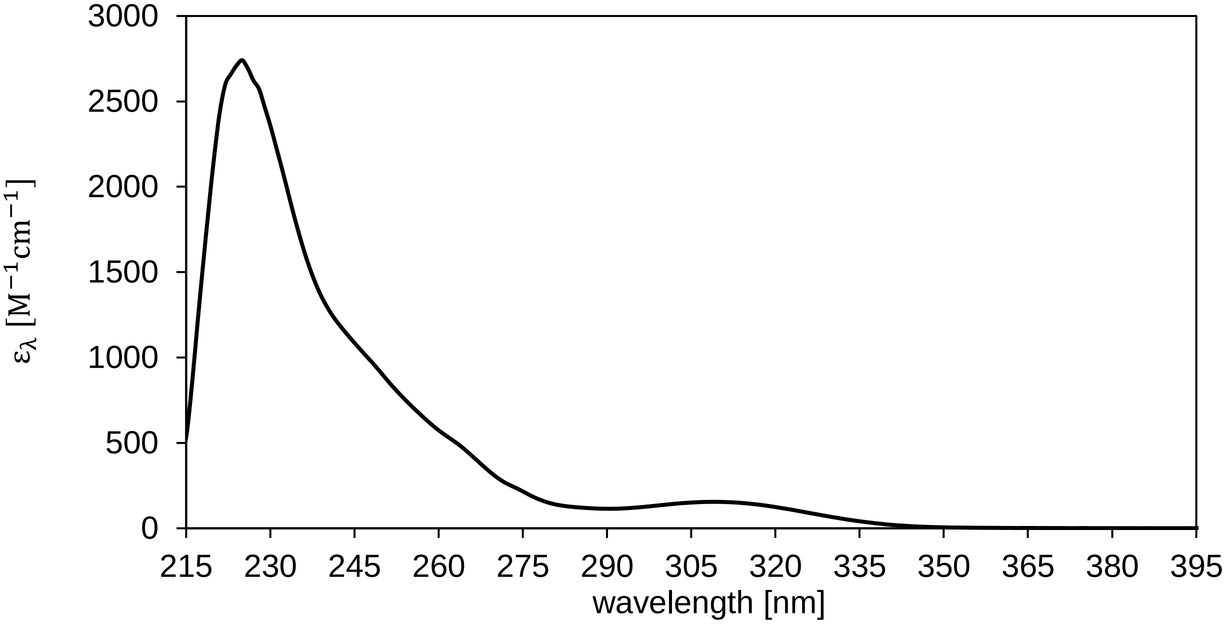

UV/Vis-Spectrum of *rac*-**5l**:

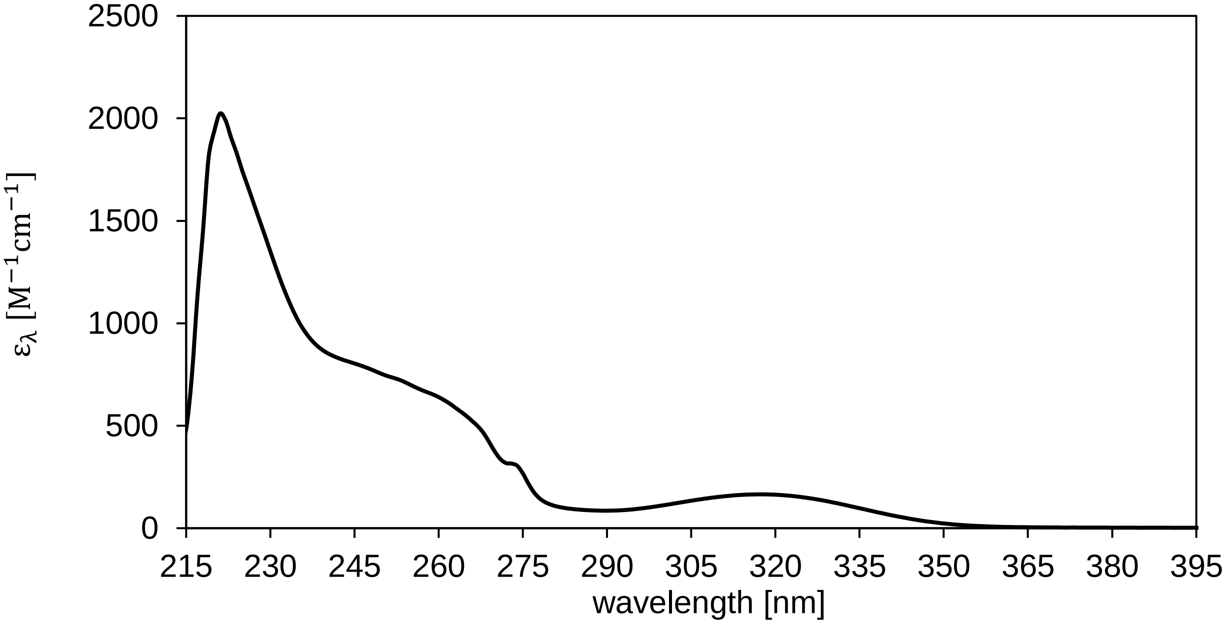

UV/Vis-Spectrum of *rac*-**5m**:

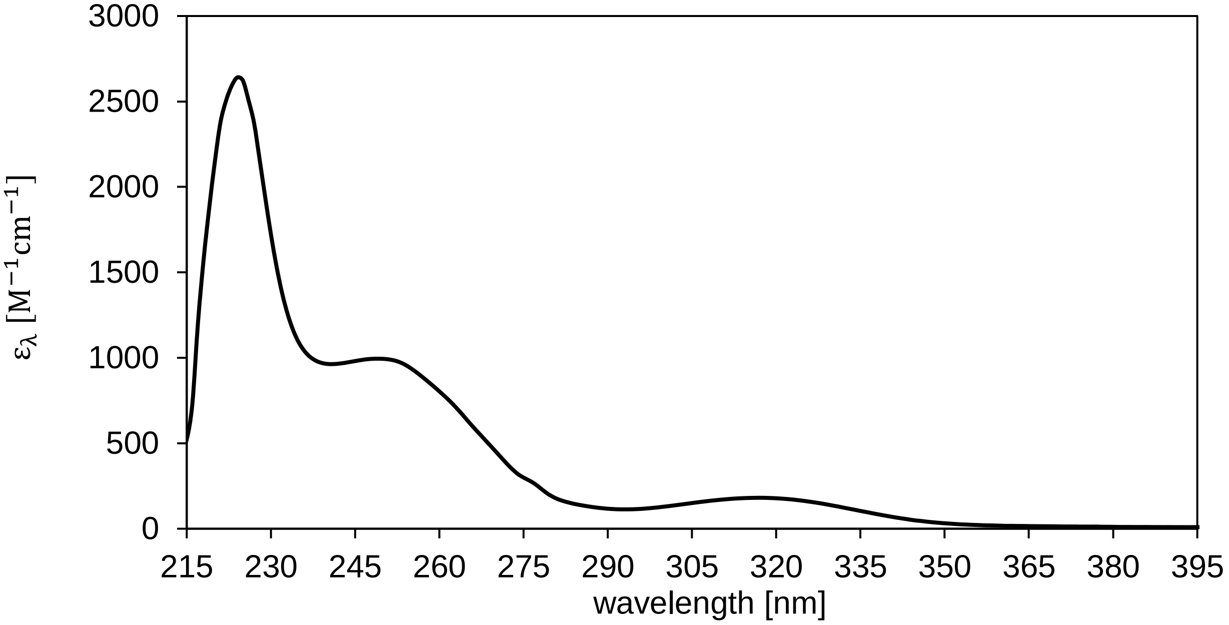

UV/Vis-Spectrum of *rac*-**5n**:

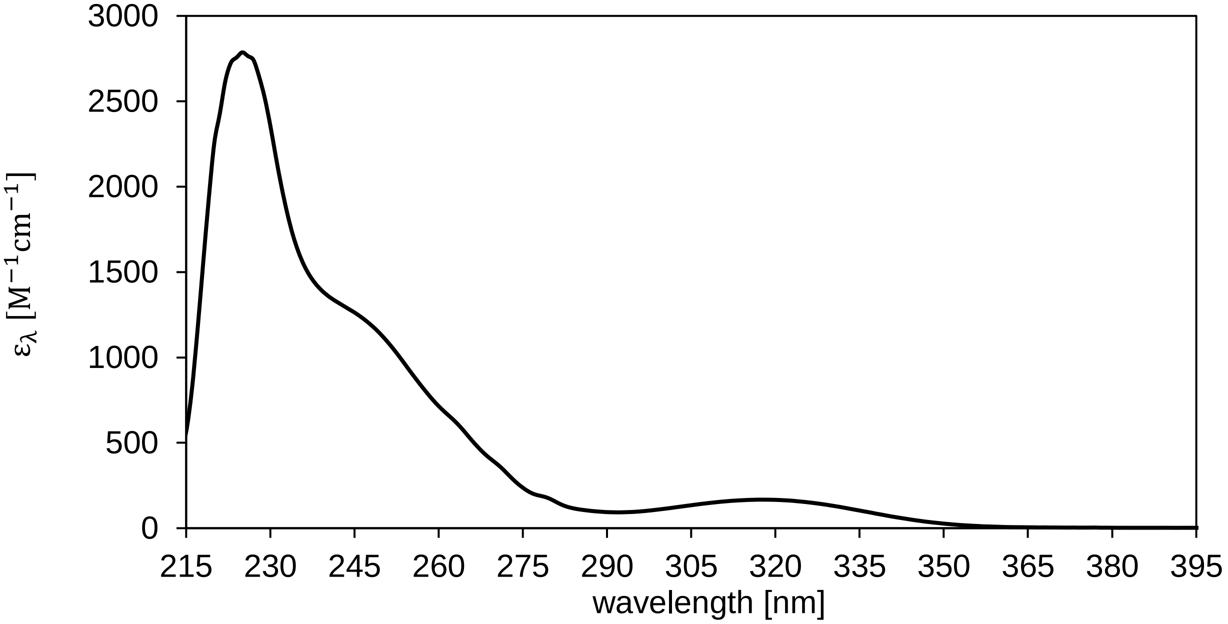

UV/Vis-Spectrum of *rac*-**5o**:

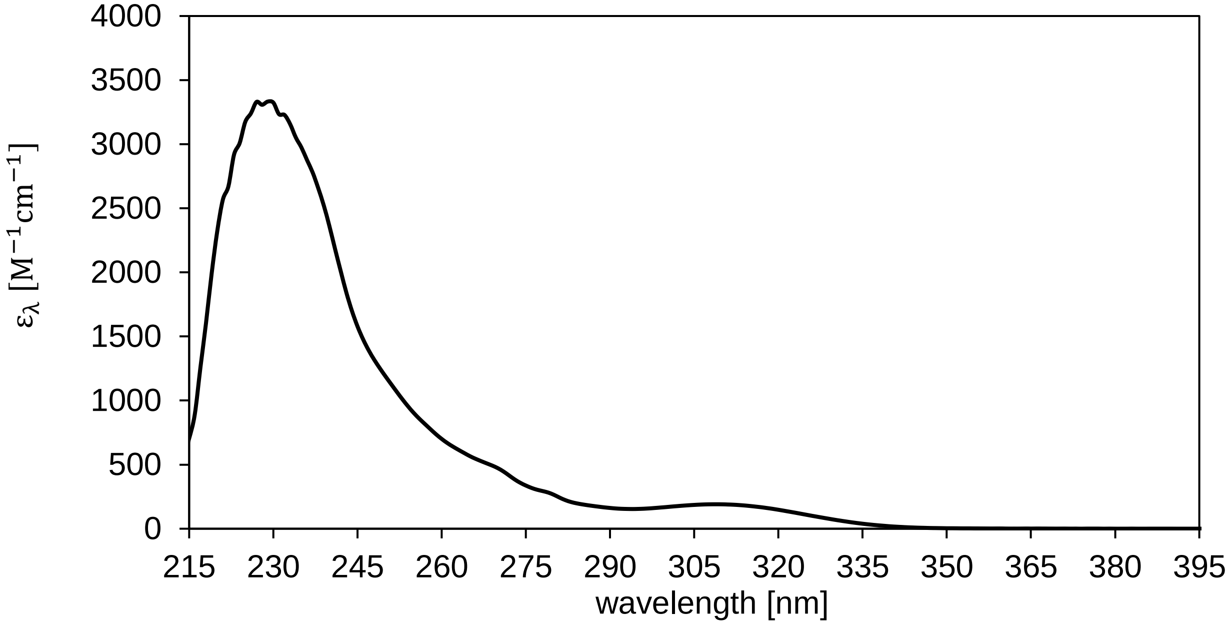

Supplement: Supplementary file 1 — ja5c02483_si_001.pdf [file ja5c02483_si_001.pdf]
